# Catalytic enantioselective oxidative coupling of saturated ethers with carboxylic acid derivatives

**DOI:** 10.1038/s41467-019-08473-x

**Published:** 2019-02-04

**Authors:** Gang Wang, Xiaodong Xin, Zehua Wang, Gang Lu, Yudao Ma, Lei Liu

**Affiliations:** 1 School of Pharmaceutical Sciences, Shandong University, 250100 Jinan, China; 2 School of Chemistry and Chemical Engineering, Shandong University, 250100 Jinan, China

## Abstract

Catalytic enantioselective C–C bond forming process through cross-dehydrogenative coupling represents a promising synthetic strategy, but it remains a long-standing challenge in chemistry. Here, we report a formal catalytic enantioselective cross-dehydrogenative coupling of saturated ethers with diverse carboxylic acid derivatives involving an initial oxidative acetal formation, followed by nickel(II)-catalyzed asymmetric alkylation. The one-pot, general, and modular method exhibits wide compatibility of a broad range of saturated ethers not only including prevalent tetrahydrofuran and tetrahydropyran, but also including medium- and large-sized cyclic moieties and acyclic ones with excellent enantioselectivity and functional group tolerance. The application in the rapid preparation of biologically active molecules that are difficult to access with existing methods is also demonstrated.

## Abstract (web summary)

Cross-dehydrogenative coupling (CDC) is a powerful method for C-C bond formation however the enantioselective variant is underdeveloped. Here, the authors show a formal enantioselective CDC method involving unactivated ethers and carboxylic acid derivatives allowing for the rapid preparation of biologically active molecules.

## Introduction

The cross-dehydrogenative coupling (CDC) of two readily available C–H components has emerged as a powerful approach for C–C bond construction whereby the only loss is H_2_ formally^1–3^. The design of the catalytic asymmetric variant is particularly attractive, but remains a challenging task^4–6^. Although impressive progress has been made in enantioselective CDC during the last decade, current studies predominantly focused on *N*-arylated amine^7–19^ and xanthese substrates^20–25^. In contrast, enantioselective CDC of ethers has remained scarce, and the unique existing two examples still focused on specific, activated benzylic and allylic ethers^26,27^. Our group described an enantioselective bimolecular CDC of cyclic benzylic ethers and aldehydes with high enantioselectivity^26^. Scheidt reported a delicate asymmetric intramolecular CDC of allylic ethers with appended β-keto esters, providing substituted tetrahydropyran-4-ones with excellent diastereo- and enantioselectivities^27^. On the other hand, optically pure saturated ethers with diverse α-alkyl substitutions represent ubiquitous structural motifs in numerous bioactive natural products and synthetic pharmaceuticals^28–30^. However, each catalytic asymmetric method is typically suitable for a single class of tetrahydrofuran (THF), tetrahydropyran (THP), or acyclic ether skeleton with a specific α-alkyl substitution pattern^31–46^. A general and modular catalytic enantioselective method to rapidly access saturated ethers with diverse skeletons and α-alkyl substituent patterns from readily available starting materials has remained elusive. Diverse saturated ether skeletons are basic feedstocks, and many of which, such as THF, THP, and diethyl ether, are daily used solvents in both academia and industry. Therefore, the development of catalytic asymmetric synthetic method starting from such abundant, low value chemical resources and sp^3^ C–H components is highly desirable. Herein, we report a formal catalytic enantioselective CDC of saturated ethers and carboxylic acid derivatives. The one-pot method exhibits wide compatibility of a broad range of saturated cyclic and acyclic ethers with excellent enantioselectivity. The application in the rapid preparation of biologically active molecules that are difficult to access with existing methods has also been demonstrated.

## Results

### Reaction design

Presumably, two major challenges hamper the design of catalytic asymmetric oxidative coupling starting from saturated ethers. First, due to the low reactivity of saturated ethers, the C–H cleavage process requires strongly oxidative conditions, making the compatibility with delicate asymmetric catalysis system difficult to achieve^47^. Second, while catalytic asymmetric addition to aromatic oxocarbenium ions has received considerable attentions^48–53^, the lack of any site on non-aromatic oxocarbenium ions for substrate–catalyst interactions makes asymmetric synthesis of α-substituted saturated ethers via such intermediates remains elusive^54–56^. Herein, we communicate a one-pot catalytic asymmetric synthetic method involving a broad range of saturated ethers and diverse carboxylic acid derivatives as starting materials. The one-pot synthetic method was designed as follow: saturated ether reacting with a combination of a protic additive and peroxide furnished a racemic acetal, which then ionized to corresponding oxocarbenium intermediate for enantioselective C–C bond forging process.

### Reaction condition optimization

The reaction of THF (**1a**) and acetyloxazolidinone **2a** was selected for optimization using bisoxazoline **L1**/Mg(ClO_4_)_2_ as chiral catalyst (Table 1)^57–61^. An initial survey revealed that oxidation of THF with hexanoic acid proceeded smoothly in the presence of ^t^BuOOH and a suitable additive, including TBAI, Fe(acac)_3_, Cu(acac)_2_, Cu(OAc)_2_, and CuOAc^62^. However, these catalytic amount of additive exhibited a dramatic effect on the subsequent enantioselective C–C bond forming reaction in the presence of BF_3_·OEt_2_/2,4,6-collidine, and CuOAc was identified to be optimal, providing expected **3a** in 14% yield with 17% ee as a separable mixture of diastereomers (d.r. = 50:50) (entries 1–5, Table 1). Lowing the loading of CuOAc afforded an improved reaction efficiency and ee (entry 6, Table 1). Acetyloxazolidinethione **2aa** proved to be a better component with regard to ee and yield (entry 7, Table 1). An extensive investigation of the combination of Lewis acid with chiral ligands **L1**-**L6** revealed that diphosphine **L6** and Ni(OTf)_2_ provided the highest yields and enantiocontrol (entries 8–17, Table 1). By using pre-prepared **L6**·Ni(OTf)_2_ as catalyst, the yield increased from 55% to 61% without detriment to ee (entry 18, Table 1). By increasing the loading of BF_3_·OEt_2_, the enantioselectivity and yield were improved to 90% and 86%, respectively (entry 19, Table 1). The reaction was also highly dependent on the solvent choice, and the nucleophilic addition stage worked the best in a mixed THF/CH_2_Cl_2_ (entry 20, Table 1). The effect of protic additives was further explored, and reaction with PhCOOH furnished the product in 81% yield and 98% ee as a 67:33 mixture of diastereomers, which were separable through silica gel chromatography (entry 21, Table 1). The combination of BF_3_·OEt_2_/2,4,6-collidine proved to be crucial to the expected reactivity (See Supplementary Table 1). No desired product was detected when BF_3_·OEt_2_ was replaced with TMSOTf or 2,4,6-collidine with diisopropylethylamine. Lowering the reaction temperature for the asymmetric C–C bond forming process did not provide any improvement on the diastereoselectivity (entry 22, Table 1).

**Table 1:** Reaction condition optimization^a^


Entry	L/Lewis acid	Additive	Yield (%)^b^	d.r.^c^	ee (%)^d^
1	**L1**/Mg(ClO_4_)_2_	TBAI	<5	n.d.	n.d.
2	**L1**/Mg(ClO_4_)_2_	Fe(acac)_3_	7	50:50	6/9
3	**L1**/Mg(ClO_4_)_2_	Cu(acac)_2_	9	50:50	10/12
4	**L1**/Mg(ClO_4_)_2_	Cu(OAc)_2_	11	50:50	13/14
5	**L1**/Mg(ClO_4_)_2_	CuOAc	14	50:50	17/16
6^e^	**L1**/Mg(ClO_4_)_2_	CuOAc	19	50:50	23/21
7^e,f^	**L1**/Mg(ClO_4_)_2_	CuOAc	27	51:49	28/26
8^e,f^	**L1**/Ni(ClO_4_)_2_	CuOAc	25	55:45	34/35
9^e,f^	**L1**/Cu(ClO_4_)_2_	CuOAc	<5	n.d.	n.d.
10^e,f^	**L2**/Ni(ClO_4_)_2_	CuOAc	21	54:46	26/23
11^e,f^	**L3**/Ni(ClO_4_)_2_	CuOAc	<5	n.d.	n.d.
12^e,f^	**L4**/Ni(ClO_4_)_2_	CuOAc	40	53:47	49/46
13^e,f^	**L5**/Ni(ClO_4_)_2_	CuOAc	38	55:45	60/61
14^e,f^	**L6**/Ni(ClO_4_)_2_	CuOAc	50	62:38	79/78
15^e,f^	**L6**/NiBr_2_	CuOAc	<5	n.d.	n.d.
16^e,f^	**L6**/Ni(OTf)_2_	CuOAc	54	64:36	82/81
17^e,f^	**L6**/Ni(SbF_6_)_2_	CuOAc	55	63:37	80/80
18^e,f,g^	**L6**·Ni(OTf)_2_	CuOAc	61	64:36	82/81
19^e,f,g,h^	**L6**·Ni(OTf)_2_	CuOAc	86	66:34	90/88
20^e,f,g,h,i^	**L6**·Ni(OTf)_2_	CuOAc	80	68:32	96/95
21^e,f,g,h,i,j^	**L6**·Ni(OTf)_2_	CuOAc	81	67:33	98/98
22^e,f,g,h,i,j,k^	**L6**·Ni(OTf)_2_	CuOAc	12	66:34	96/97

n.d., not determined

^a^Reaction condition: hexanoic acid (0.5 mmol, 2.5 equiv), additive (0.02 mmol, 10 mol%), and ^t^BuOOH in decane (0.5 mmol, 2.5 equiv) in THF (1 mL) at 80 °C for 2 h, followed by addition of **2a** (0.2 mmol, 1.0 equiv), **L** (0.024 mmol, 12 mol%), Lewis acid (0.02 mmol, 10 mol%), 2,4,6-collidine (0.6 mmol, 3.0 equiv), and BF_3_·OEt_2_ (0.6 mmol, 3.0 equiv) in CH_2_Cl_2_ (0.4 mL) at rt for 2 h, unless otherwise noted

^b^Isolated yield of the two diastereomers

^c^Determined by ^1^H NMR spectroscopy

^d^Determined by chiral HPLC analysis

^e^CuOAc (0.005 mmol, 2.5 mol%) used

^f^Acetyloxazolidinethione **2aa** used

^g^Pre-prepared **L6**·Ni(OTf)_2_ used

^h^BF_3_·OEt_2_ (0.8 mmol, 4.0 equiv) used

^i^THF/CH_2_Cl_2_ (3:1, v/v) as solvent for nucleophilic addition step

^j^PhCOOH instead of hexanoic acid as protic additive

^k^Asymmetric nucleophilic addition reaction performed at 0 °C

### Scope of acetic acid derivatives

**Fig. 1:**
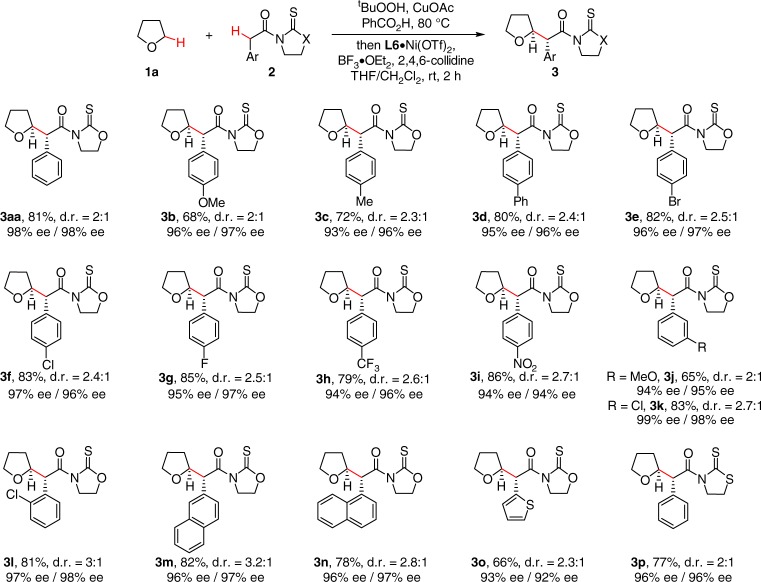
The scope of α-aryl acetic acid derivatives. Conditions: PhCOOH (0.5 mmol, 2.5 equiv), CuOAc (2.5 mol%), ^t^BuOOH in decane (2.5 equiv) in THF (1.0 mL) at 80 °C for 2 h, followed by 2 (1.0 equiv), L6·Ni(OTf)_2_ (10 mol%), 2,4,6-collidine (3.0 equiv) and BF_3_·OEt_2_ (4.0 equiv) in THF/CH_2_Cl_2_ (0.3 mL/0.1 mL) at rt for 2 h

The scope of acetic acid derivatives was then explored (Fig. 1). A variety of electron-donating and -withdrawing substituents at the *ortho*-, *meta*-, and *para*-positions of the aryl ring were well compatible with the reaction conditions, providing respective α-aryl acetamide substituted THF **3a**–**3l** in good yields with moderate diastereoselectivity and excellent enantioselectivity (93–98% ee) for both isomers. (α-Heteroaryl) **3o**, as well as (α-nathphyl)acyl oxazolidinethiones **3m** and **3n** were suitable components in high enantioselectivities. *N*-phenylacyl thiazolidinethione was also well tolerated, as demonstrated by the generation of **3p** in 96% ee.

**Fig. 2:**
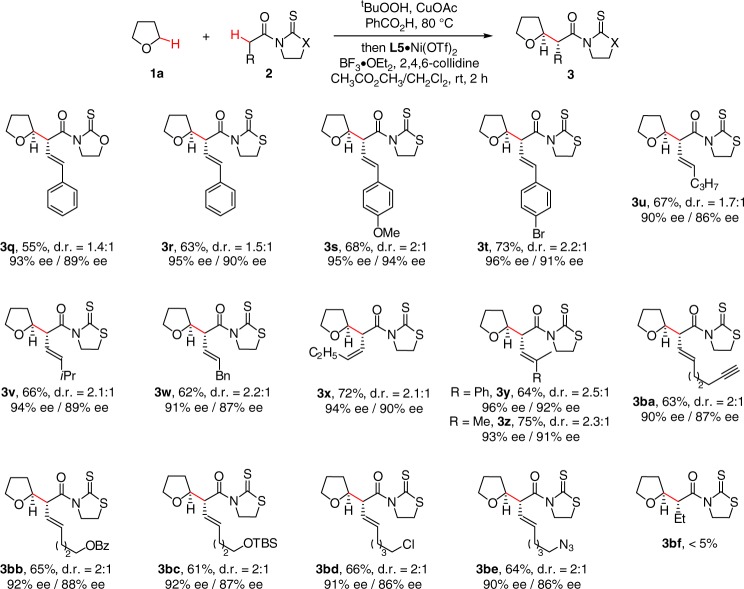
The scope of α-alkenyl acetic acid derivatives. Conditions: PhCOOH (0.5 mmol, 2.5 equiv), CuOAc (2.5 mol%), ^t^BuOOH in decane (2.5 equiv) in THF (1.0 mL) at 80 °C for 2 h, followed by 2 (1.0 equiv), L5·Ni(OTf)_2_ (10 mol%), 2,4,6-collidine (3.0 equiv), and BF_3_·OEt_2_ (4.0 equiv) in CH_3_CO_2_CH_3_/CH_2_Cl_2_ (0.3 mL/0.1 mL) at rt for 2 h

To further expand the synthetic utility of the method, we next investigated the possibility of α-alkenyl acetic acid derivatives as coupling components (Fig. 2). Thiazolidinethione **3r** was superior to oxazolidinethione **3q** in terms of ee and yield. In general, the reaction exhibited excellent regioselectivity, and the alkylation occurred exclusively at the α-position of 3-butenoyl derivatives with olefin geometry highly conserved. Electronically varied γ-aryl substituted 3-butenoyl **2s** and **2t** together with γ-alkyl substituted **2u**–**2x** were competent components, providing corresponding **3s**–**3w** in good yields with 90–96% ee. γ-Disubstituted **2y** and **2z** were also well tolerated in excellent enantiocontrol. The method had an excellent functional group tolerance, with common functionalities including terminal alkyne (**3ba**), benzoate (**3bb**), silyl ether (**3bc**), halide (**3bd**), and azide (**3be**) well tolerated in 90–92% ee for further manipulation. No expected product **3bf** was observed when α-alkyl acetic acid derivative **2bf** was used as the coupling partner.

### Scope of saturated ethers

**Fig. 3:**
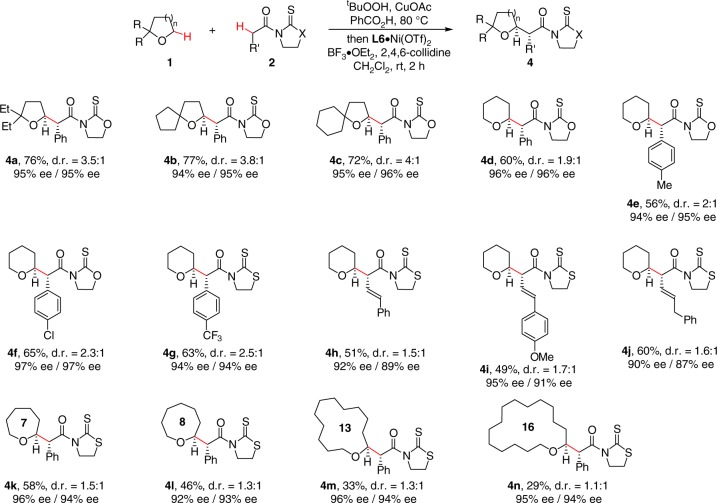
The scope of cyclic saturated ethers. Conditions: PhCOOH (0.5 mmol, 2.5 equiv), CuOAc (2.5 mol%), ^t^BuOOH in decane (3.0 equiv) in ether (1.0 mL) at 80 °C for 6–12 h, followed by 2 (1.0 equiv), L5·Ni(OTf)_2_ or L6·Ni(OTf)_2_ (10 mol%), 2,4,6-collidine (3.0 equiv), and BF_3_·OEt_2_ (4.0 equiv) in CH_2_Cl_2_ (0.4 mL) at rt for 2 h

The scope of saturated cyclic ethers was next examined (Fig. 3). Under the standard conditions, 1,1-disubstituted THF proved to be competent substrates, furnishing trisubstituted THF **4a**–**4c** in good yields with 94–96% ee and moderate d.r.. Enantioselective reaction of THP with α-aryl and α-vinyl substituted acetic acid derivatives also proceeded smoothly, giving corresponding **4d**–**4j** with excellent enantiocontrol. The success in the prevalent THF and THP frameworks encouraged us to further investigate the feasibility of enantioselective functionalization of larger cyclic ethers. Delightedly, medium-sized cyclic, aliphatic ethers, such as oxepane and oxocane, were tolerated, providing **4k** and **4l** in 96% ee and 92% ee, respectively. Albeit slightly decreased efficiency, large-sized cyclic, saturated ethers were also identified as competent substrates, as demonstrated by the generation of 13-membered **4m** and 16-membered **4n** in 96% ee and 95% ee. Although the scope of saturated cyclic ethers was not exclusively investigated, these results provide a proof-of-concept for the generality and modularity of the asymmetric synthetic method.

**Fig. 4:**
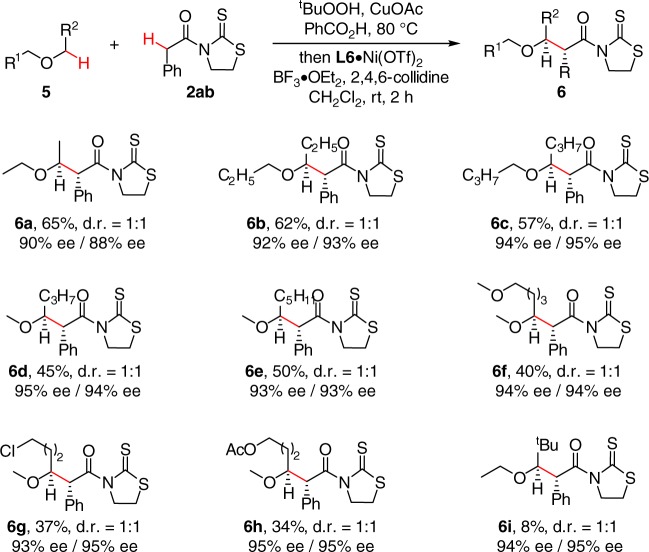
The scope of acyclic saturated ethers. Conditions: PhCOOH (0.5 mmol, 2.5 equiv), CuOAc (2.5 mol%), ^t^BuOOH in decane (3.0 equiv) in ether (1.0 mL) at 80 °C for 6–12 h, followed by 2 (1.0 equiv), L6·Ni(OTf)_2_ (10 mol%), 2,4,6-collidine (3.0 equiv), and BF_3_·OEt_2_ (4.0 equiv) in CH_2_Cl_2_ (0.4 mL) at rt for 2 h

The tolerance of large-sized, cyclic ether prompted us to further explore the generality of the method for acyclic saturated ethers (Fig. 4). Pleasingly, enantioselective reaction of commonly used solvents, such as diethyl ether, dipropyl ether, and dibutyl ether, with **2aa** proceeded smoothly, providing **6a**–**6c** in good yields with 90–94% ee. Aside from symmetric acyclic ethers, unsymmetric ones also proved to be suitable substrates, as demonstrated by the highly enantioselective reaction of methyl n-butyl ether (**6d**) and methyl n-hexyl ether (**6e**). The reaction exhibited excellent regioselectivity, and no substitution at primary C–H bond was observed. Acyclic ethers bearing a variety of commonly encountered functional groups, such as ether (**6f**), halide (**6g**), and acetate (**6h**), were also well tolerated for further manipulation. No improvement on the diastereoselectivity was observed when bulky *tert*-butyl ethyl ether (**6i**) was employed.

### Synthetic applications

**Fig. 5:**
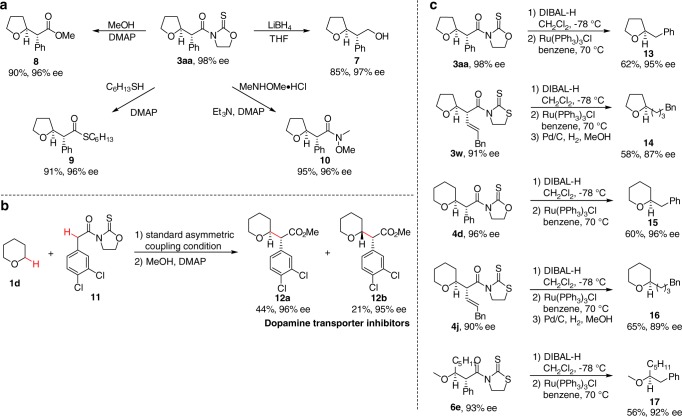
Synthetic utilities. **a** Transformations of oxa- and thiazolidinethiones to other synthetically valuable functional groups. **b** Rapid synthesis of two dopamine transporter inhibitors. **c** Removal of the oxa- and thiazolidinethione moieties

The synthetic utilities of the method was next explored (Fig. 5). First, the oxa- and thiazolidinethione moieties can be readily converted to other synthetically valuable functional groups. For example, the adduct **3a** was converted to corresponding alcohol **7**, ester **8**, thioester **9**, and Weinreb amide **10** in high efficiency with the ee highly conserved (Fig. 5a). Enantiopure diastereomers **12a** and **12b** are potent and selective dopamine transporter inhibitors (Fig. 5b)^63^. However, traditional synthetic method, which relied on menthol or 1-indanol mediated resolution technology, required seven steps for each isomer preparation starting from 3,4-dihydro-2*H*-pyran. Our method exhibited moderate diastereoselectivity, which afforded an opportunity to rapidly access both **12a** and **12b** in excellent ee through a two-step process consisting of asymmetric reaction of THP (**1d**) with **11** and esterification. Second, given the tolerance of a broad range of carboxylic acid derivatives, we envisioned that removing the exocyclic stereocenter would further enhance the synthetic utility of the protocol. Therefore, we designed a concise two-step sequence involving DIBAL-H mediated reduction of oxa- or thiazolidinethione to aldehyde followed by Ru(PPh_3_)_3_Cl catalyzed decarbonylation, providing α-alkyl substituted saturated ethers **13**–**17** that are difficult to prepare by other methods (Fig. 5c).

## Discussion

**Fig. 6:**
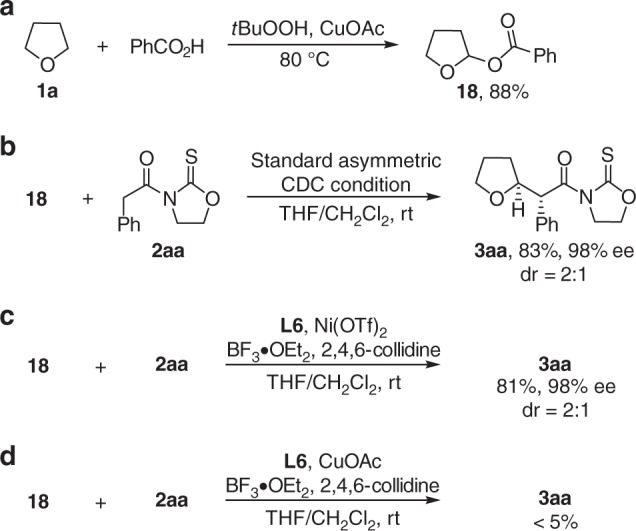
Mechanistic studies. **a** Reaction intermediate identification. **b** Asymmetric coupling of 18 with 2aa. **c** Asymmetric coupling of 18 with 2aa in the absence of CuOAc. **d** Identification of the real metal catalyst for the asymmetric coupling

Control experiments were conducted to gain a preliminary understanding of the reaction mechanism (Fig. 6). First, in the presence of CuOAc and ^t^BuOOH, THF (**1a**) reacted with PhCO_2_H giving acetate **18** in 88% yield (Fig. 6a). Subjecting **18** to the standard condition furnished expected **3aa** with comparable results to that observed in the reaction starting from THF (**1a**) (Fig. 6b). The observations implied the intermediacy of **18** in the process. Second, the alkylation of **18** with **2aa** proceeded smoothly under the standard reaction condition in the absence of oxidation elements (Fig. 6c). No expected **3aa** was observed when the combination of **L6** and CuOAc was used as the catalyst (Fig. 6d). These results suggested that the species binding to diphosphine ligand should be Ni(OTf)_2_ but not CuOAc.

**Fig. 7:**
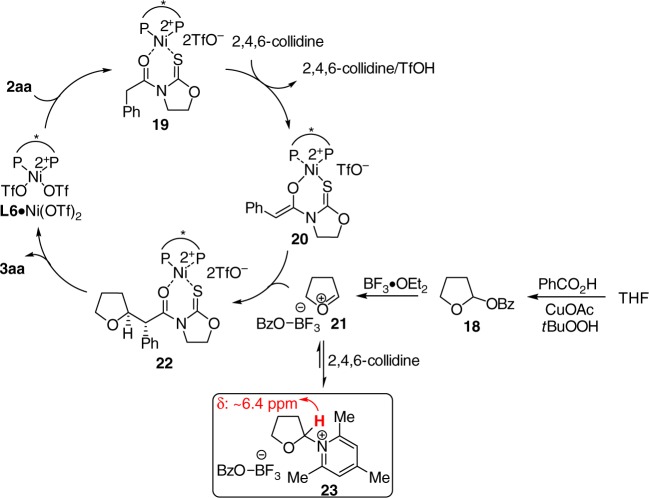
A proposed catalytic cycle. The possible reaction pathway based on our studies and previous literature

Based on the above observations, a plausible catalytic cycle is outlined in Fig. 7. Carboxylic acid derivative **2aa** coordinates with **L6**·Ni(OTf)_2_ complex giving rise to **19**, in which the acidity of the hydrogen α to the carbonyl moiety increases. 2,4,6-Collidine promotes the enolization of **19** providing chiral Ni-bound *Z*-enolate **20**^64^. **20** Reacts with cyclic oxocarbenium **21** generated in situ from acetal **18** and BF_3_·OEt_2_ yielding **22**. Product dissociation completes the catalytic cycle. Notably, no reactivity was detected when diisopropylethylamine was used instead of 2,4,6-collidine, indicating that the role of latter might not simply act as a base. Fujioka and Kita reported that in the presence of a suitable Lewis acid, the acetal reacted with 2,4,6-collidine furnishing corresponding pyridinium-type salt^65^. During our in situ ^1^H NMR study of the mixture of acetal **18**, BF_3_·OEt_2_, and 2,4,6-collidine, a peak at δ ~ 6.4 ppm was observed, which is characteristic for such type of pyridinium species^65,66^. Accordingly, we envisioned that 2,4,6-collidine might react with oxocarbenium **21** reversibly furnishing pyridinium **23**. Adduct **23** possessing weak electrophilicity might be considered as a reservoir of **21** to prevent the decomposition before the capture by enolate **20**.

**Fig. 8:**
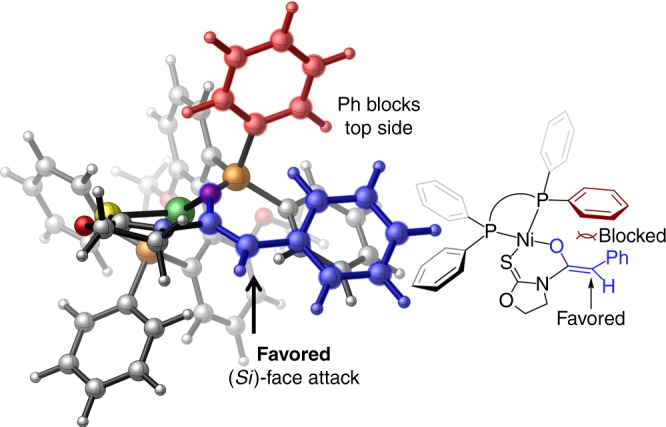
Stereochemical induction model with chiral Ni-bound *Z*-enolate. The geometry was optimized at the B3LYP/LANL2DZ–6–31G(d) level

The stereochemical induction model based on the Ni-bound *Z*-enolate with chiral bidentate phosphine ligand **L6** (MeO-BIPHEP) was proposed in Fig. 8. The *P*-bound phenyl group on the ligand (in red) shields the top face (*Re* face) of the enolate substrate (in blue), which disfavors the electrophilic attack by the oxocarbenium intermediate. In contrast, the addition of the oxocarbenium ion to the *Si* face of the enolate substrate is favored due to less repulsive interactions with the ligand. This model is consistent with the experimentally observed stereochemistry. The low to moderate diastereoselectivity observed for saturated ethers might be ascribed to the lack of Lewis basic site on corresponding oxocarbenium ion intermediates for substrate–catalyst interactions.

In summary, a one-pot catalytic enantioselective reaction of saturated ethers with diverse carboxylic acid derivatives is described. The general and modular method exhibits wide compatibility of a broad range of saturated cyclic ethers not only including prevalent THF and THP, but also including medium- and large-sized cyclic moieties with excellent enantioselectivity and functional group tolerance. The generality of the method is further demonstrated by application in saturated acyclic ethers. The synthetic application in the rapid preparation of biologically active molecules that are difficult to access with existing methods is demonstrated. We envision that the general, modular, and highly enantioselective reaction of abundant, low value saturated ethers outlined herein will provide a topologically straightforward synthetic planning for both complex target molecules and a plethora of analogs for lead discovery and optimization.

## Methods

### General procedure for the reaction of THF (1a) with 2aa

In an oven-dried Teflon septum screw-capped tube, PhCOOH (61 mg, 0.5 mmol), CuOAc (0.6 mg, 0.005 mmol, 2.5 mol%), and ^t^BuOOH in decane (0.5 mmol) were added to THF (1.0 mL). The solution was stirred at 80 °C for 2 h before the solvent was evaporated. Then carboxylic acid derivative **2aa** (44.3 mg, 0.2 mmol), **L6·**Ni(OTf)_2_ (18.7 mg, 0.02 mmol), 2,4,6-collidine (79.3 μL, 0.6 mmol), and BF_3_·OEt_2_ (98.7 μL, 0.8 mmol) in THF/CH_2_Cl_2_ (0.3 mL/0.1 mL, v-v) was added. After stirring at rt for 2 h, solvents were removed and the residue was purified by silica gel column chromatography (CH_2_Cl_2_/ethyl acetate = 99:1) giving the expected **3aa** in 81% yield (47.2 mg) with 98% ee as a separable mixture of diastereomers (d.r. = 2:1).

## Supplementary Information

Supplementary Information

## Data Availability

The authors declare that the data supporting the findings of this study are available within the article and its Supplementary Information files. The X-ray crystallographic coordinates for structures reported in this article have been deposited at the Cambridge Crystallographic Data Center (**3p**: CCDC 1858037, **3p**’: CCDC 1858065, **3s**: CCDC 1858049, **4e**: CCDC 1858043, **4l**: CCDC 1890776). These data could be obtained free of charge from The Cambridge Crystallographic Data Center via www.ccdc.cam.ac.uk/data_request/cif.
