# Supplementary material for: Catalytic enantioselective oxidative coupling of saturated ethers with carboxylic acid derivatives
Source: Nat Commun. 2019 Feb 4;10:559. doi: 10.1038/s41467-019-08473-x (PMC6362111; doi:10.1038/s41467-019-08473-x)
Supplement: Supplementary file 1 — Supplementary Information [file 41467_2019_8473_MOESM1_ESM.pdf]

Supplementary Information

Catalytic Enantioselective Oxidative Coupling of Saturated Ethers with Carboxylic Acid Derivatives

Wang et al.

Supplementary Methods

General Information

Proton (^{1}H NMR) and carbon (^{13}C NMR) nuclear magnetic resonance spectra were recorded at 500 MHz and 126 MHz, respectively. The chemical shifts are given in parts per million (ppm) on the delta (δ) scale. The solvent peak was used as a reference value, for ^{1}H NMR: $CDCl_3$ = 7.27 ppm, for ^{13}C NMR: $CDCl_3$ = 77.23. Analytical TLC was performed on precoated silica gel GF254 plates. Column chromatography was carried out on silica gel (200–300 mesh). IR spectra were recorded on an ALPHA-T spectrometer in the frequency range of 400-4000 cm^{-1}. HRMS were measured on an Orbitrap analyzer. The X-ray single-crystal determination was performed on Bruker APEX-II CCD diffractometer. Optical rotations were measured using a 2.5 mL cell with a 10 cm path length on Hanon P850 Automatic Polarimeter and concentrations (c) were reported in g×(100 mL)$^{-1}$. Enantiomeric excesses were determined by HPLC using a Daicel Chiralpak and Chiralcel column with hexane/*i*-PrOH as the eluent on Dionex instrument.

Synthesis of Carboxylic Acid Derivative

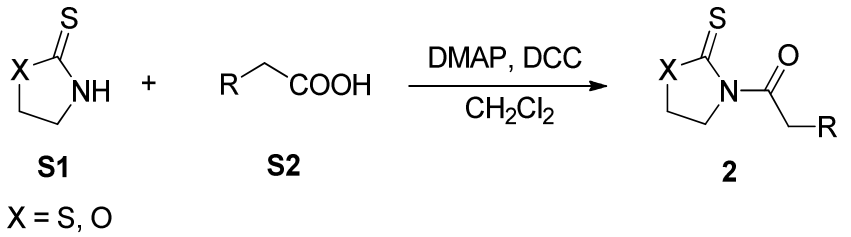

General Procedure A: To a suspension of **S1** (5 mmol), DMAP (0.5 mmol), and **S2** (7 mmol) in CH_2Cl_2 (10 mL) at 0 °C was added DCC (7 mmol) in one portion. The mixture was stirred at rt until all the starting material was consumed monitored by TLC. The dicyclohexylurea formed was filtered and the precipitate washed with CH_2Cl_2. The filtrate was washed with sat. $NaHCO_3$, dried with $MgSO_4$, concentrated at reduced pressure, and the residue was purified by silica gel chromatography (ethyl acetate/petroleum ether 10:90 to 20:80) to give the desired **2**.

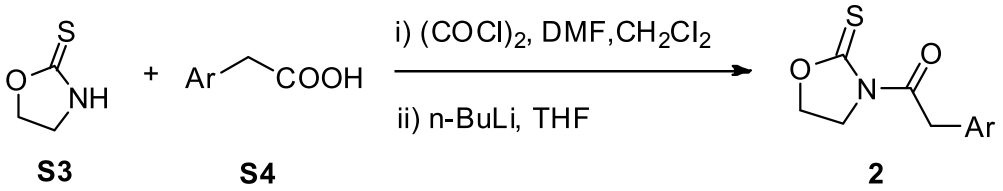

General Procedure B: To a solution of **S4** (6 mmol) in CH_2Cl_2 (20 mL) was added $(COCl)_2$ (15 mmol) and a few drops of dry DMF at 0 °C. The mixture was stirred for 2 h before all the volatiles were removed under vacuum and the residue was taken up in THF (5 mL). To a solution of oxazolidinone (5 mmol) in THF (20 mL) was slowly added n-BuLi (5 mmol) at -78 °C, and the mixture was stirred at the same temperature for 30 min. Then a solution of freshly prepared acid chloride in THF was slowly added at -78 °C. The resulting mixture was stirred for 2 h at -78 °C and allowed to room temperature. The reaction was quenched with aqueous NH_4Cl (20 mL) and the mixture was extracted with EtOAc. The organic layers were washed with brine, dried over $MgSO_4$, concentrated at reduced pressure and the residue was purified by silica gel chromatography (ethyl acetate/petroleum ether 20:80) to give the desired **2**.

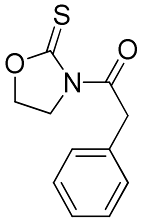

2-Phenyl-1-(2-thioxooxazolidin-3-yl)ethanone (2aa)

It was prepared following the general procedure A using 2-phenylacetic acid as starting material on a 10 mmol scale to afford **2aa** in 76% yield (1.68 g). ^{1}H NMR (500 MHz, $CDCl_3$) δ 7.40–7.32 (m, 2H), 7.32–7.27 (m, 3H), 4.74 (s, 2H), 4.54 (t, J = 8.5 Hz, 2H), 4.24 (t, J = 8.5 Hz, 2H); ^{13}C NMR (126 MHz, $CDCl_3$) δ 185.8, 172.6, 133.7, 130.0, 128.7, 127.4, 66.6, 47.5, 43.2; IR (KBr): 3331, 3021, 2953, 2871, 1695, 1512, 1375, 1154, 1017, 975 cm^{-1}; HRMS (EI) m/z $[M + H]^+$ calculated for $C_{11}H_{12}NO_2S$: 222.0583, found 222.0581.

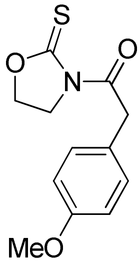

2-(4-Methoxyphenyl)-1-(2-thioxooxazolidin-3-yl)ethanone (2b)

It was prepared following the general procedure A using 2-(4-methoxyphenyl)acetic acid as starting material on a 5 mmol scale to afford **2b** in 71% yield (890 mg). ^{1}H NMR (500 MHz, $CDCl_3$) δ 7.20 (d, *J* = 8.7 Hz, 2H), 6.95–6.82 (m, 2H), 4.67 (s, 2H), 4.55 (t, *J* = 8.5 Hz, 2H), 4.24 (t, *J* = 8.5 Hz, 2H), 3.81 (s, 3H); ^{13}C NMR (126 MHz, $CDCl_3$) δ 185.8, 173.0, 159.0, 131.1, 125.7, 114.3, 66.6, 55.5, 47.5, 42.3; IR (KBr): 3326, 2957, 2916, 2838, 1713, 1610, 1513, 1386, 1154, 1017, 978, 813 cm^{-1}; HRMS (EI) m/z $[M + H]^+$ calculated for $C_{12}H_{14}NO_3S$: 252.0689, found 252.0688.

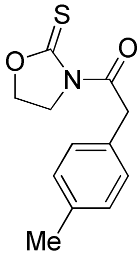

1-(2-Thioxooxazolidin-3-yl)-2-(p-tolyl)ethanone (2c)

It was prepared following the general procedure A using 2-(*p*-tolyl)acetic acid as starting material on a 5 mmol scale to afford **2c** in 74% yield (870 mg). ^{1}H NMR (500 MHz, $CDCl_3$) δ 7.24–7.06 (m, 4H), 4.69 (s, 2H), 4.54 (t, J = 8.5 Hz, 2H), 4.24 (t, J = 8.5 Hz, 2H), 2.35 (s, 3H); ^{13}C NMR (126 MHz, CDCl3) δ 185.8, 172.8, 137.1, 130.5, 129.8, 129.5, 66.6, 47.5, 42.8, 21.3; IR (KBr): 3328, 3001, 2933, 2844, 1695, 1513, 1377, 1154, 1015, 977 cm^{-1}; HRMS (EI) m/z $[M + H]^+$ calculated for $C_{12}H_{14}NO_2S$: 236.0740, found 236.0742.

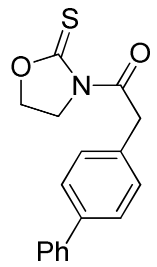

2-([1,1'-Biphenyl]-4-yl)-1-(2-thioxooxazolidin-3-yl)ethanone (2d)

It was prepared following the general procedure A using 2-([1,1'-biphenyl]-4-yl)acetic acid as starting material on a 5 mmol scale to afford **2d** in 81% yield (1.20 g). ^{1}H NMR (500 MHz, $CDCl_3$) δ 7.65–7.54 (m, 4H), 7.45 (t, J = 7.7 Hz, 2H), 7.42–7.30 (m, 3H), 4.79 (s, 2H), 4.57 (t, J = 8.5 Hz, 2H), 4.27 (t, J = 8.5 Hz, 2H); ^{13}C NMR (126 MHz, $CDCl_3$) δ 185.8, 172.6, 141.0, 140.4, 132.7, 130.4, 129.0, 127.5, 127.5, 127.3, 66.6, 47.5, 42.9; IR (KBr): 3327, 3005, 2914, 2852, 1693, 1515, 1380, 1339, 1242, 1183, 1020 cm^{-1}; HRMS (EI) m/z [M + H]$^{+}$ calculated for $C_{17}H_{16}NO_2S$: 298.0896, found 298.0899.

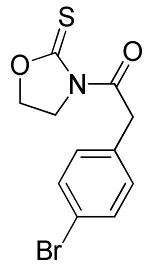

2-(4-Bromophenyl)-1-(2-thioxooxazolidin-3-yl)ethanone (2e)

It was prepared following the general procedure B using 2-(4-bromophenyl)acetic acid as starting material on a 5 mmol scale to afford **2e** in 71% yield (1.06 g). ^{1}H NMR (500 MHz, $CDCl_3$) δ 7.47 (d, J = 8.4 Hz, 2H), 7.16 (d, J = 8.3 Hz, 2H), 4.69 (s, 2H), 4.57 (t, J = 8.5 Hz, 2H), 4.25 (t, J = 8.5 Hz, 2H); ^{13}C NMR (126 MHz, $CDCl_3$) δ 185.7, 172.1, 132.6, 131.9, 131.8, 121.5, 66.7, 47.5, 42.7; IR (KBr): 3354, 3031, 2953, 2911, 1695, 1511, 1402, 1365, 1220, 1018, 976, 794 cm^{-1}; HRMS (EI) m/z [M + H]$^{+}$ calculated for $C_{11}H_{11}BrNO_2S$: 299.9688, found 299.9690.

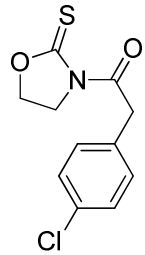

2-(4-Chlorophenyl)-1-(2-thioxooxazolidin-3-yl)ethanone (2f)

It was prepared following the general procedure B using 2-(4-chlorophenyl)acetic acid as starting material on a 5 mmol scale to afford **2f** in 75% yield (0.96 g). ^{1}H NMR (500 MHz, $CDCl_3$) δ 7.32 (d, *J* = 8.4 Hz, 2H), 7.21 (d, *J* = 8.5 Hz, 2H), 4.70 (s, 2H), 4.56 (t, *J* = 8.5 Hz, 2H), 4.25 (t, *J* = 8.5 Hz, 2H); ^{13}C NMR (126 MHz, $CDCl_3$) δ 185.7, 172.2, 133.4, 132.1, 131.4, 128.9, 66.6, 47.5, 42.6; IR (KBr): 3361, 3029, 2954, 2921, 1694, 1510, 1402, 1365, 1015, 977, 795 cm^{-1}; HRMS (EI) m/z $[M + H]^+$ calculated for $C_{11}H_{11}ClNO_2S$: 256.0194, found 256.0195.

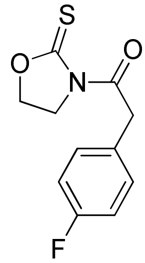

2-(4-Fluorophenyl)-1-(2-thioxooxazolidin-3-yl)ethanone (2g)

It was prepared following the general procedure B using 2-(4-fluorophenyl)acetic acid as starting material on a 5 mmol scale to afford **2g** in 65% yield (777 mg). ^{1}H NMR (500 MHz, $CDCl_3$) δ 7.27–7.21 (m, 2H), 7.04 (t, *J* = 8.7 Hz, 2H), 4.70 (s, 2H), 4.57 (t, *J* = 8.5 Hz, 2H), 4.26 (t, *J* = 8.5 Hz, 2H); ^{13}C NMR (126 MHz, $CDCl_3$) δ 185.7, 172.5, 162.27 (d, *J* = 245.7 Hz), 131.60 (d, *J* = 8.1 Hz), 129.29 (d, *J* = 3.3 Hz), 115.64 (d, *J* = 21.4 Hz), 66.6, 47.5, 42.5; IR (KBr): 3372, 3048, 2974, 2918, 1695, 1511, 1405, 1361, 1191, 1020, 978, 793 cm^{-1}; HRMS (EI) m/z $[M + H]^+$ calculated for $C_{11}H_{11}FNO_2S$: 240.0489, found 240.0487.

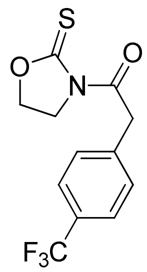

1-(2-Thioxooxazolidin-3-yl)-2-(4-(trifluoromethyl)phenyl)ethanone (2h)

It was prepared following the general procedure B using 2-(4-(trifluoromethyl) phenyl)acetic acid as starting material on a 5 mmol scale to afford **2h** in 68% yield (982 mg). ^{1}H NMR (500 MHz, $CDCl_3$) δ 7.61 (d, *J* = 8.1 Hz, 2H), 7.40 (d, *J* = 8.0 Hz, 2H), 4.80 (s, 2H), 4.57 (t, *J* = 8.5 Hz, 2H), 4.26 (t, *J* = 8.5 Hz, 2H); ^{13}C NMR (126 MHz, $CDCl_3$) δ 185.7, 171.7, 137.7, 130.4, 129.8 (q, *J* = 32.5 Hz), 125.6 (q, *J* = 3.7 Hz), 124.3 (q, *J* = 272.0 Hz), 66.7, 47.5, 43.0; IR (KBr): 3368, 3054, 2977, 2915, 1688, 1521, 1391, 1375, 1248, 1017, 897 cm^{-1}; HRMS (EI) m/z $[M + H]^+$ calculated for $C_{12}H_{11}F_3NO_2S$: 290.0457, found 290.0454.

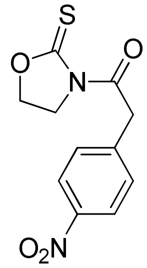

2-(4-Nitrophenyl)-1-(2-thioxooxazolidin-3-yl)ethanone (2i)

It was prepared following the general procedure B using 2-(4-nitrophenyl)acetic acid as starting material on a 5 mmol scale to afford **2i** in 66% yield (875 mg). ^{1}H NMR (500 MHz, $CDCl_3$) δ 8.22 (d, *J* = 8.6 Hz, 2H), 7.45 (d, *J* = 8.6 Hz, 2H), 4.85 (s, 2H), 4.61 (t, *J* = 8.5 Hz, 2H), 4.28 (t, *J* = 8.5 Hz, 2H); ^{13}C NMR (126 MHz, $CDCl_3$) δ 185.6, 171.2, 145.6, 141.1, 131.1, 123.9, 66.8, 47.5, 43.2; IR (KBr): 3771, 3351, 3081, 2962, 2907, 1683, 1528, 1387, 1369, 1223, 1018, 976, 818, 729 cm^{-1}; HRMS (EI) m/z $[M + H]^+$ calculated for $C_{11}H_{11}N_2O_4S$: 267.0434, found 267.0434.

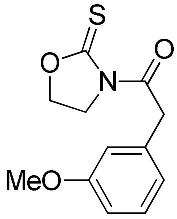

2-(3-Methoxyphenyl)-1-(2-thioxooxazolidin-3-yl)ethanone (2j)

It was prepared following the general procedure A using 2-(3-methoxyphenyl)acetic acid as starting material on a 2 mmol scale to afford **2j** in 75% yield (375 mg). ^{1}H NMR (500 MHz, $CDCl_3$) δ 7.28–7.25 (m, 1H), 6.87 (d, *J* = 7.7 Hz, 1H), 6.86–6.80 (m, 2H), 4.71 (s, 2H), 4.55 (t, *J* = 8.5 Hz, 2H), 4.25 (t, *J* = 8.5 Hz, 2H), 3.81 (s, 3H); ^{13}C NMR (126 MHz, $CDCl_3$) δ 185.8, 172.5, 159.9, 135.1, 129.7, 122.3, 115.7, 112.9, 66.6, 55.4, 47.5, 43.2; IR (KBr): 3324, 2955, 2915, 2836, 1710, 1512, 1389, 1244, 1153, 1017, 705 cm^{-1}; HRMS (EI) m/z $[M + H]^+$ calculated for $C_{12}H_{14}NO_3S$: 252.0689, found 252.0684.

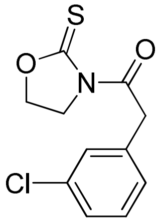

2-(3-Chlorophenyl)-1-(2-thioxooxazolidin-3-yl)ethanone (2k)

It was prepared following the general procedure B using 2-(3-chlorophenyl)acetic acid as starting material on a 2 mmol scale to afford **2k** in 69% yield (352 mg). ^{1}H NMR (500 MHz, $CDCl_3$) δ 7.31–7.25 (m, 3H), 7.22–7.12 (m, 1H), 4.72 (s, 2H), 4.57 (t, *J* = 8.5 Hz, 2H), 4.26 (t, *J* = 8.5 Hz, 2H); ^{13}C NMR (126 MHz, $CDCl_3$) δ 185.7, 171.9, 135.6, 134.5, 130.1, 129.9, 128.3, 127.7, 66.7, 47.5, 42.8; IR (KBr): 3358, 3027, 2954, 2918, 1694, 1511, 1411, 1366, 1016, 717 cm^{-1}; HRMS (EI) m/z $[M + H]^+$ calculated for $C_{11}H_{11}ClNO_2S$: 256.0194, found 256.0193.

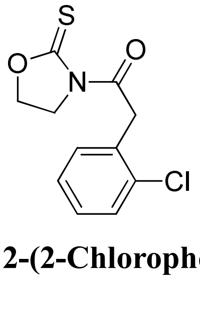

2-(2-Chlorophenyl)-1-(2-thioxooxazolidin-3-yl)ethanone (2l)

It was prepared following the general procedure B using 2-(2-chlorophenyl)acetic acid as starting material on a 2 mmol scale to afford **2l** in 65% yield (331 mg). ^{1}H NMR (500 MHz, $CDCl_3$) δ 7.46–7.37 (m, 1H), 7.28–7.24 (m, 3H), 4.78 (s, 2H), 4.60 (t, J = 8.5 Hz, 2H), 4.28 (t, J = 8.5 Hz, 2H); ^{13}C NMR (126 MHz, $CDCl_3$) δ 185.8, 171.3, 134.9, 132.6, 131.9, 129.7, 129.1, 127.2, 66.8, 47.4, 42.9; IR (KBr): 3364, 3031, 2953, 2920, 1695, 1511, 1409, 1365, 1015, 758 cm^{-1}; HRMS (EI) m/z [M + H]$^+$ calculated for $C_{11}H_{11}ClNO_2S$: 256.0194, found 256.0196.

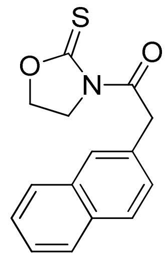

2-(Naphthalen-2-yl)-1-(2-thioxooxazolidin-3-yl)ethanone (2m)

It was prepared following the general procedure A using 2-(naphthalen-2-yl)acetic acid as starting material on a 2 mmol scale to afford **2m** in 85% yield (460 mg). ^{1}H NMR (500 MHz, $CDCl_3$) δ 7.86–7.78 (m, 3H), 7.75 (s, 1H), 7.52–7.44 (m, 2H), 7.41 (dd, J = 8.4, 1.6 Hz, 1H), 4.90 (s, 2H), 4.53 (t, J = 8.5 Hz, 2H), 4.25 (t, J = 8.5 Hz, 2H); ^{13}C NMR (126 MHz, $CDCl_3$) δ 185.8, 172.6, 133.6, 132.8, 131.2, 128.7, 128.3, 128.1, 127.9, 127.9, 126.3, 126.1, 66.6, 47.5, 43.3; IR (KBr): 3367, 3035, 2913, 1693, 1381, 1365, 1209, 1015, 975 cm^{-1}; HRMS (EI) m/z [M + H]$^+$ calculated for $C_{15}H_{14}NO_2S$: 272.0740, found 272.0741.

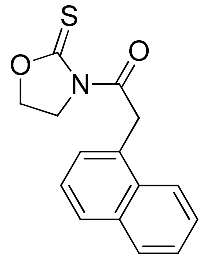

2-(Naphthalen-1-yl)-1-(2-thioxooxazolidin-3-yl)ethanone (2n)

It was prepared following the general procedure A using 2-(naphthalen-1-yl)acetic acid as starting material on a 2 mmol scale to afford **2n** in 82% yield (444 mg). ^{1}H NMR (500 MHz, $CDCl_3$) δ 7.92–7.86 (m, 1H), 7.86–7.79 (m, 2H), 7.57–7.48 (m, 2H),

7.48–7.43 (m, 1H), 7.38 (d, *J* = 6.9 Hz, 1H), 5.16 (s, 2H), 4.62–4.48 (m, 2H), 4.31–4.17 (m, 2H); ^{13}C NMR (126 MHz, $CDCl_3$) δ 185.9, 172.3, 134.0, 132.4, 130.6, 129.0, 128.4, 128.2, 126.5, 125.9, 125.6, 123.9, 66.7, 47.5, 41.5; IR (KBr): 3370, 3038, 2909, 1692, 1379, 1355, 1202, 1172, 1017, 942, 788 cm^{-1}; HRMS (EI) m/z $[M + H]^+$ calculated for $C_{15}H_{14}NO_2S$: 272.0740, found 272.0738.

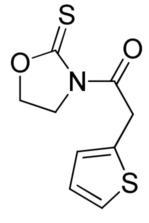

2-(Thiophen-2-yl)-1-(2-thioxooxazolidin-3-yl)ethanone (2o)

It was prepared following the general procedure B using 2-(thiophen-2-yl)acetic acid as starting material on a 5 mmol scale to afford **2o** in 60% yield (680 mg). ^{1}H NMR (500 MHz, $CDCl_3$) δ 7.28–7.27 (m, 1H), 7.07–6.95 (m, 2H), 4.97 (s, 2H), 4.58 (t, *J* = 8.5 Hz, 2H), 4.27 (t, *J* = 8.5 Hz, 2H); ^{13}C NMR (126 MHz, $CDCl_3$) δ 185.6, 171.5, 134.4, 127.7, 126.9, 125.7, 66.7, 47.5, 37.6; IR (KBr): 3372, 3039, 2964, 1696, 1510, 1368, 1274, 1017, 698 cm^{-1}; HRMS (EI) m/z $[M + H]^+$ calculated for $C_9H_{10}NO_2S_2$: 228.0147, found 228.0149.

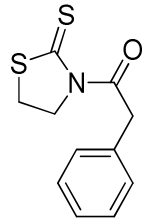

2-Phenyl-1-(2-thioxothiazolidin-3-yl)ethanone (2p)

It was prepared following the general procedure A using 2-phenylacetic acid as starting material on a 5 mmol scale to afford **2p** in 78% yield (924 mg). ^{1}H NMR (500 MHz, $CDCl_3$) δ 7.39–7.31 (m, 2H), 7.31–7.27 (m, 1H), 7.27–7.22 (m, 2H), 4.67 (s, 2H), 4.59 (t, *J* = 7.5 Hz, 2H), 3.29 (t, *J* = 7.5 Hz, 2H); ^{13}C NMR (126 MHz, $CDCl_3$) δ 202.0, 173.0, 134.0, 129.9, 128.7, 127.3, 56.5, 44.6, 28.5; IR (KBr): 3388, 3026, 2935, 1703, 1344, 1222, 1162, 1145, 1037, 765, 712 cm^{-1}; HRMS (EI) m/z $[M + H]^+$ calculated for $C_{11}H_{12}NOS_2$: 238.0355, found 238.0356.

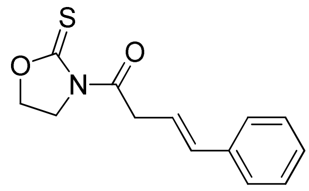

(*E*)-4-Phenyl-1-(2-thioxooxazolidin-3-yl)but-3-en-1-one (2q)

It was prepared following the general procedure A using (*E*)-4-phenylbut-3-enoic acid as starting material on a 5 mmol scale to afford **2q** in 65% yield (801 mg). ^{1}H NMR (500 MHz, $CDCl_3$) δ 7.44–7.37 (m, 2H), 7.36–7.29 (m, 2H), 7.27–7.21 (m, 1H), 6.57 (d, *J* = 16.0 Hz, 1H), 6.41 (dt, *J* = 15.9, 6.8 Hz, 1H), 4.57 (t, *J* = 8.5 Hz, 2H), 4.30 (dd, *J* = 6.8, 1.1 Hz, 2H), 4.26 (t, *J* = 8.5 Hz, 2H); ^{13}C NMR (126 MHz, $CDCl_3$) δ 185.7, 172.6, 137.0, 134.3, 128.8, 127.8, 126.6, 121.3, 66.7, 47.3, 41.2; IR (KBr): 3307, 3012, 2941, 1691, 1368, 1261, 1148, 1044, 757 cm^{-1}; HRMS (EI) m/z $[M + H]^+$ calculated for $C_{13}H_{14}NO_2S$: 248.0740, found 248.0744.

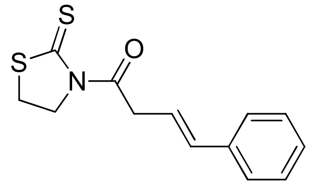

(*E*)-4-Phenyl-1-(2-thioxothiazolidin-3-yl)but-3-en-1-one (2r)

It was prepared following the general procedure A using (*E*)-4-phenylbut-3-enoic acid as starting material on a 5 mmol scale to afford **2r** in 71% yield (933 mg). ^{1}H NMR (500 MHz, $CDCl_3$) δ 7.43–7.37 (m, 2H), 7.36–7.29 (m, 2H), 7.27–7.22 (m, 1H), 6.53 (d, *J* = 16.0 Hz, 1H), 6.48–6.31 (m, 1H), 4.62 (t, *J* = 7.5 Hz, 2H), 4.24 (d, *J* = 6.8 Hz, 2H), 3.32 (t, *J* = 7.5 Hz, 2H); ^{13}C NMR (126 MHz, $CDCl_3$) δ 201.9, 173.0, 137.1, 134.0, 128.7, 127.8, 126.5, 121.8, 56.2, 42.5, 28.5; IR (KBr): 3328, 2974, 2931, 1695, 1373, 1255, 1159, 1044, 749 cm^{-1}; HRMS (EI) m/z $[M + H]^+$ calculated for $C_{13}H_{14}NOS_2$: 264.0511, found 264.0512.

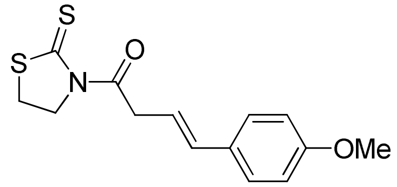

(*E*)-4-(4-Methoxyphenyl)-1-(2-thioxothiazolidin-3-yl)but-3-en-1-one (2s)

It was prepared following the general procedure A using (*E*)-4-(4-methoxyphenyl)

but-3-enoic acid as starting material on a 5 mmol scale to afford **2s** in 78% yield (1.14 g). ^{1}H NMR (500 MHz, $CDCl_3$) δ 7.33 (d, *J* = 8.7 Hz, 2H), 6.85 (d, *J* = 8.7 Hz, 2H), 6.47 (d, *J* = 15.9 Hz, 1H), 6.24 (dt, *J* = 15.9, 6.9 Hz, 1H), 4.61 (t, *J* = 7.5 Hz, 2H), 4.21 (dd, *J* = 6.9, 1.1 Hz, 2H), 3.81 (s, 3H), 3.31 (t, *J* = 7.5 Hz, 2H); ^{13}C NMR (126 9MHz, $CDCl_3$) δ 201.8, 173.2, 159.4, 133.4, 129.9, 127.71, 119.5, 114.1, 56.3, 55.5, 42.5, 28.5; IR (KBr): 3311, 2953, 2912, 1708, 1381, 1255, 1221, 1148, 1043, 788 cm^{-1}; HRMS (EI) m/z $[M + H]^+$ calculated for $C_{14}H_{16}NO_2S_2$: 294.0617, found 294.0616.

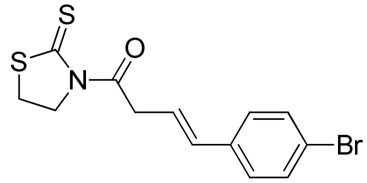

(*E*)-4-(4-Bromophenyl)-1-(2-thioxothiazolidin-3-yl)but-3-en-1-one (2t)

It was prepared following the general procedure A using (*E*)-4-(4-bromophenyl) but-3-enoic acid as starting material on a 5 mmol scale to afford **2t** in 64% yield (1.08 g). ^{1}H NMR (500 MHz, $CDCl_3$) δ 7.44 (d, *J* = 8.4 Hz, 2H), 7.25 (d, *J* = 8.4 Hz, 2H), 6.50–6.32 (m, 2H), 4.62 (t, *J* = 7.5 Hz, 2H), 4.23 (d, *J* = 6.4 Hz, 2H), 3.32 (t, *J* = 7.5 Hz, 2H); ^{13}C NMR (126 MHz, $CDCl_3$) δ 201.9, 172.7, 136.0, 132.8, 131.8, 128.1, 122.7, 121.5, 56.2, 42.5, 28.5; IR (KBr): 3330, 2978, 2941, 1693, 1369, 1265, 1162, 1041, 1011, 791 cm^{-1}; HRMS (EI) m/z $[M + H]^+$ calculated for $C_{13}H_{13}BrNOS_2$: 341.9616, found 341.9620.

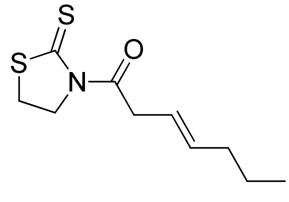

(*E*)-1-(2-Thioxothiazolidin-3-yl)hept-3-en-1-one (2u)

It was prepared following the general procedure A using (*E*)-hept-3-enoic acid as starting material on a 5 mmol scale to afford **2u** in 72% yield (824 mg). ^{1}H NMR (500 MHz, $CDCl_3$) δ 5.63–5.54 (m, 2H), 4.59 (t, *J* = 7.5 Hz, 2H), 4.00 (d, *J* = 4.8 Hz, 2H), 3.30 (t, *J* = 7.5 Hz, 2H), 2.04 (dd, *J* = 12.5, 6.8 Hz, 2H), 1.51–1.29 (m, 2H), 0.90 (t, *J* = 7.4 Hz, 3H); ^{13}C NMR (126 MHz, $CDCl_3$) δ 201.7, 173.7, 135.4, 121.6, 56.3, 42.3,

34.9, 28.5, 22.5, 13.95; IR (KBr): 3370, 2956, 2869, 1699, 1365, 1281, 1155, 1052 cm^{-1}; HRMS (EI) m/z $[M + H]^+$ calculated for $C_{10}H_{16}NOS_2$: 230.0668, found 230.0666.

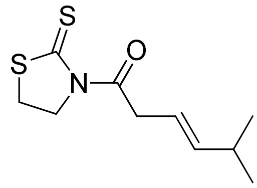

(*E*)-5-Methyl-1-(2-thioxothiazolidin-3-yl)hex-3-en-1-one (2v)

It was prepared following the general procedure A using (*E*)-5-methylhex-3-enoic acid as starting material on a 5 mmol scale to afford **2v** in 68% yield (778 mg). ^1H NMR (500 MHz, $CDCl_3$) δ 5.55–5.41 (m, 2H), 4.52 (t, J = 7.5 Hz, 2H), 3.97–3.86 (m, 2H), 3.22 (t, J = 7.5 Hz, 2H), 2.32–2.18 (m, 1H), 0.92 (d, J = 6.8 Hz, 6H); ^{13}C NMR (126 MHz, $CDCl_3$) δ 201.7, 173.7, 142.4, 118.6, 56.2, 42.3, 31.3, 28.5, 22.5; IR (KBr): 3374, 3309, 2958, 2868, 1700, 1464, 1364, 1280, 1154, 1051, 1004, 882, 713 cm^{-1}; HRMS (EI) m/z $[M + H]^+$ calculated for $C_{10}H_{16}NOS_2$: 230.0668, found 230.0669.

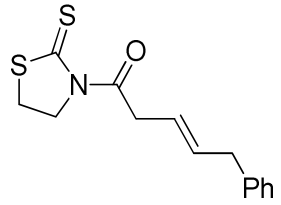

(*E*)-5-Phenyl-1-(2-thioxothiazolidin-3-yl)pent-3-en-1-one (2w)

It was prepared following the general procedure A using (*E*)-5-phenylpent-3-enoic acid as starting material on a 5 mmol scale to afford **2w** in 71% yield (980 mg). ^1H NMR (500 MHz, $CDCl_3$) δ 7.34–7.28 (m, 2H), 7.24–7.17 (m, 3H), 5.83–5.64 (m, 2H), 4.59 (t, J = 7.5 Hz, 2H), 4.05 (d, J = 4.8 Hz, 2H), 3.42 (d, J = 4.7 Hz, 2H), 3.29 (t, J = 7.5 Hz, 2H); ^{13}C NMR (126 MHz, $CDCl_3$) δ 201.7, 173.3, 140.3, 133.7, 128.7, 128.6, 126.3, 123.2, 56.2, 42.1, 39.2, 28.5; IR (KBr): 3374, 3025, 2894, 1699, 1362, 1280, 1152, 1051, 700 cm^{-1}; HRMS (EI) m/z $[M + H]^+$ calculated for $C_{14}H_{16}NOS_2$: 278.0668, found 278.0665.

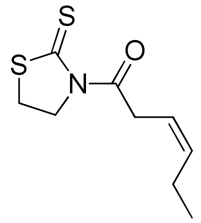

(*Z*)-1-(2-Thioxothiazolidin-3-yl)hex-3-en-1-one (2x)

It was prepared following the general procedure A using (*Z*)-hex-3-enoic acid as starting material on a 5 mmol scale to afford **2x** in 65% yield (700 mg). ^{1}H NMR (500 MHz, $CDCl_3$) δ 5.69–5.55 (m, 2H), 4.59 (t, *J* = 7.5 Hz, 2H), 4.06 (d, *J* = 5.4 Hz, 2H), 3.30 (t, *J* = 7.5 Hz, 2H), 2.12–2.02 (m, 2H), 0.98 (td, *J* = 7.5, 2.0 Hz, 3H); ^{13}C NMR (126 MHz, $CDCl_3$) δ 201.8, 173.3, 135.7, 119.9, 56.3, 37.2, 28.5, 21.3, 14.1; IR (KBr): 3380, 2963, 2933, 2874, 1701, 1368, 1280, 1155, 1051, 885 cm^{-1}; HRMS (EI) m/z [M + H]$^+$ calculated for $C_9H_{14}NOS_2$: 216.0511, found 216.0512.

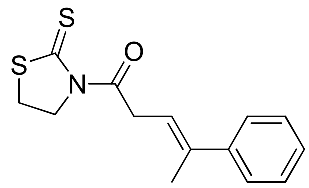

(*E*)-4-Phenyl-1-(2-thioxothiazolidin-3-yl)pent-3-en-1-one (2y)

It was prepared following the general procedure A using (*E*)-4-phenylpent-3-enoic acid as starting material on a 2.5 mmol scale to afford **2y** in 80% yield (550 mg). ^{1}H NMR (500 MHz, $CDCl_3$) δ 7.43 (d, *J* = 7.5 Hz, 2H), 7.33 (t, *J* = 7.6 Hz, 2H), 7.27–7.24 (m, 1H), 6.06 (t, *J* = 6.7 Hz, 1H), 4.63 (t, *J* = 7.5 Hz, 2H), 4.26 (d, *J* = 6.7 Hz, 2H), 3.32 (t, *J* = 7.5 Hz, 2H), 2.09 (s, 3H); ^{13}C NMR (126 MHz, $CDCl_3$) δ 201.9, 173.1, 143.3, 138.6, 128.4, 127.3, 126.1, 119.1, 56.4, 38.9, 28.5, 16.9; IR (KBr): 3345, 2944, 2915, 1698, 1371, 1277, 1151, 1048, 721 cm^{-1}; HRMS (EI) m/z [M + H]$^+$ calculated for $C_{14}H_{16}NOS_2$: 278.0668, found 278.0664.

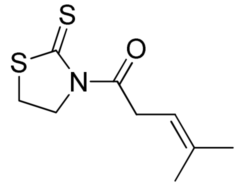

4-Methyl-1-(2-thioxothiazolidin-3-yl)pent-3-en-1-one (2z)

It was prepared following the general procedure A using 4-methylpent-3-enoic acid as

starting material on a 5 mmol scale to afford **2z** in 72% yield (774 mg). ^{1}H NMR (500 MHz, $CDCl_3$) δ 5.47–5.32 (m, 1H), 4.59 (t, *J* = 7.5 Hz, 2H), 4.02 (d, *J* = 6.8 Hz, 2H), 3.30 (t, *J* = 7.5 Hz, 2H), 1.77 (s, 3H), 1.65 (s, 3H); ^{13}C NMR (126 MHz, $CDCl_3$) δ 201.8, 173.8, 136.2, 115.6, 56.4, 38.3, 28.5, 25.9, 18.6; IR (KBr): 3352, 2967, 2931, 2856, 1700, 1366, 1281, 1153, 1051, 710 cm^{-1}; HRMS (EI) m/z $[M + H]^+$ calculated for $C_9H_{14}NOS_2$: 216.0511, found 216.0509.

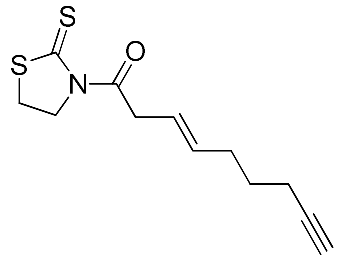

(*E*)-1-(2-Thioxothiazolidin-3-yl)non-3-en-8-yn-1-one (3ba)

It was prepared following the general procedure A using (*E*)-non-3-en-8-ynoic acid as starting material on a 2.5 mmol scale to afford **3aa** in 66% yield (415 mg). ^{1}H NMR (500 MHz, $CDCl_3$) δ 5.72–5.51 (m, 2H), 4.59 (t, *J* = 7.5 Hz, 2H), 4.01 (d, *J* = 6.3 Hz, 2H), 3.30 (t, *J* = 7.5 Hz, 2H), 2.23–2.15 (m, 4H), 1.95 (t, *J* = 2.6 Hz, 1H), 1.68–1.60 (m, 2H); ^{13}C NMR (126 MHz, $CDCl_3$) δ 201.8, 173.5, 134.1, 122.6, 84.5, 68.7, 56.2, 42.2, 31.6, 28.5, 28.1, 18.0; IR (KBr): 3289, 3260, 2941, 2859, 1698, 1364, 1280, 1149, 1051, 639 cm^{-1}; HRMS (EI) m/z $[M + H]^+$ calculated for $C_{12}H_{16}NOS_2$: 254.0668, found 254.0668.

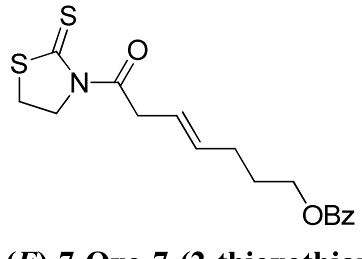

(*E*)-7-Oxo-7-(2-thioxothiazolidin-3-yl)hept-4-en-1-yl benzoate (3bb)

It was prepared following the general procedure A using (*E*)-7-(benzoyloxy)hept-3-enoic acid as starting material on a 2.5 mmol scale to afford **3ab** in 74% yield (645 mg). ^{1}H NMR (500 MHz, $CDCl_3$) δ 8.09–8.01 (m, 2H), 7.56 (t, *J* = 7.4 Hz, 1H), 7.45 (t, *J* = 7.7 Hz, 2H), 5.73–5.57 (m, 2H), 4.58 (t, *J* = 7.5 Hz, 2H), 4.34 (t, *J* = 6.5 Hz, 2H), 4.01 (d, *J* = 5.8 Hz, 2H), 3.29 (t, *J* = 7.5 Hz, 2H), 2.28–2.21 (m, 2H), 1.92–1.83

(m, 2H); ^{13}C NMR (126 MHz, $CDCl_3$) δ 201.8, 173.4, 166.8, 133.9, 133.1, 130.6, 129.7, 128.5, 122.7, 64.5, 56.2, 42.2, 29.3, 28.5, 28.4; IR (KBr): 3381, 2941, 1714, 1366, 1276, 1154, 1051, 713 cm^{-1}; HRMS (EI) m/z $[M + H]^+$ calculated for $C_{17}H_{20}NO_3S_2$: 350.0879, found 350.0876.

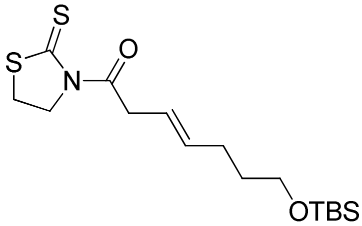

(*E*)-7-((*tert*-Butyldimethylsilyl)oxy)-1-(2-thioxothiazolidin-3-yl)hept-3-en-1-one (3bc)

It was prepared following the general procedure A using (*E*)-7-((*tert*-butyldimethyl-silyl)oxy)hept-3-enoic acid as starting material on a 2.5 mmol scale to afford **3ac** in 50% yield (450 mg). ^{1}H NMR (500 MHz, $CDCl_3$) δ 5.69–5.51 (m, 2H), 4.59 (t, *J* = 7.5 Hz, 2H), 4.00 (d, *J* = 5.0 Hz, 2H), 3.62 (t, *J* = 6.4 Hz, 2H), 3.30 (t, *J* = 7.5 Hz, 2H), 2.17–2.07 (m, 2H), 1.65–1.58 (m, 2H), 0.90 (s, 9H), 0.05 (s, 6H); ^{13}C NMR (126 MHz, $CDCl_3$) δ 201.7, 173.6, 135.0, 121.8, 62.7, 56.2, 42.2, 32.5, 29.1, 28.5, 28.5, 26.2, -5.1; IR (KBr): 3376, 2930, 2856, 1701, 1364, 1281, 1153, 1100, 1051, 836 cm^{-1}; HRMS (EI) m/z $[M + H]^+$ calculated for $C_{16}H_{30}NO_2S_2Si$: 360.1482, found 360.1481.

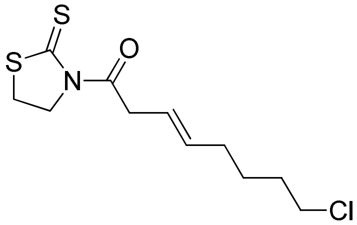

(*E*)-8-Chloro-1-(2-thioxothiazolidin-3-yl)oct-3-en-1-one (3bd)

It was prepared following the general procedure A using (*E*)-8-chlorooct-3-enoic acid as starting material on a 2.5 mmol scale to afford **3ad** in 70% yield (485 mg). ^{1}H NMR (500 MHz, $CDCl_3$) δ 5.66–5.53 (m, 2H), 4.59 (t, *J* = 7.5 Hz, 2H), 4.01 (d, *J* = 6.1 Hz, 2H), 3.54 (t, *J* = 6.7 Hz, 2H), 3.30 (t, *J* = 7.5 Hz, 2H), 2.15–2.05 (m, 2H), 1.82–1.76 (m, 2H), 1.58–1.51 (m, 2H); ^{13}C NMR (126 MHz, $CDCl_3$) δ 201.8, 173.5, 134.6, 122.3, 56.2, 45.2, 42.2, 32.2, 31.9, 28.5, 26.5; IR (KBr): 3377, 2933, 2854, 1701, 1364, 1281, 1156, 1051, 712 cm^{-1}; HRMS (EI) m/z $[M + H]^+$ calculated for

$C_{11}H_{17}ClNOS_2$: 278.0435, found 278.0433.

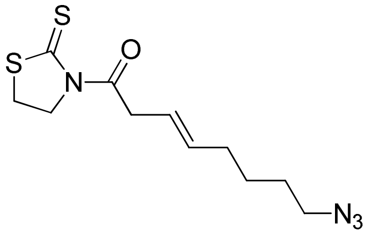

(*E*)-8-Azido-1-(2-thiox othiazolidin-3-yl)oct-3-en-1-one (3be)

It was prepared following the general procedure A using (*E*)-8-azidooct-3-enoic acid as starting material on a 2.5 mmol scale to afford **3ae** in 76% yield (540 mg). ^{1}H NMR (500 MHz, $CDCl_3$) δ 5.72–5.47 (m, 2H), 4.59 (t, *J* = 7.5 Hz, 2H), 4.06–3.96 (m, 2H), 3.32–3.26 (m, 4H), 2.10 (q, *J* = 7.0 Hz, 2H), 1.64–1.59 (m, 2H), 1.51–1.44 (m, 2H); ^{13}C NMR (126 MHz, $CDCl_3$) δ 201.8, 173.5, 134.5, 122.3, 56.2, 51.5, 42.2, 32.2, 28.5, 28.4, 26.4; IR (KBr): 3374, 2934, 2857, 2095, 1700, 1365, 1281, 1231, 1152, 1051 cm^{-1}; HRMS (EI) m/z $[M + H]^+$ calculated for $C_{11}H_{17}N_4OS_2$: 285.0838, found 285.0832.

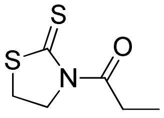

1-(2-Thioxothiazolidin-3-yl)propan-1-one (3bf)

It was prepared following the general procedure A using propionic acid as starting material on a 2.5 mmol scale to afford **3bf** in 85% yield (372 mg). ^{1}H NMR (500 MHz, $CDCl_3$) δ 4.59 (dt, *J* = 13.4, 7.6 Hz, 2H), 3.37–3.14 (m, 4H), 1.25–1.11 (m, 3H); ^{13}C NMR (126 MHz, $CDCl_3$) δ 201.8, 175.8, 56.3, 32.5, 28.6, 9.0. These data are consistent with reported literature values.[1]

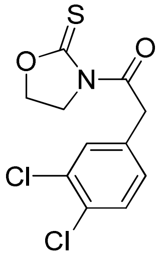

2-(3,4-Dichlorophenyl)-1-(2-thioxooxazolidin-3-yl)ethanone (11)

It was prepared following the general procedure B using 2-(3,4-dichlorophenyl)acetic acid as starting material on a 5 mmol scale to afford **11** in 71% yield (1.02 g). ^{1}H

NMR (500 MHz, $CDCl_3$) δ 7.42 (d, J = 8.2 Hz, 1H), 7.38 (d, J = 2.0 Hz, 1H), 7.12 (dd, J = 8.2, 2.1 Hz, 1H), 4.69 (s, 2H), 4.58 (t, J = 8.5 Hz, 2H), 4.26 (t, J = 8.5 Hz, 2H); ^{13}C NMR (126 MHz, $CDCl_3$) δ 185.7, 171.6, 133.7, 132.7, 132.0, 131.7, 130.6, 129.5, 66.7, 47.5, 42.3; IR (KBr): 3367, 3031, 2949, 2911, 1693, 1509, 1408, 1364, 1016, 811 cm^{-1}; HRMS (EI) m/z $[M + H]^+$ calculated for $C_{11}H_{10}Cl_2NOS_2$: 305.9575, found 305.9570.

Preparation of L5·Ni(OTf)$_2$ or L6·Ni(OTf)$_2$

To a suspension of **L5** or **L6** (0.25 mmol) in acetonitrile (5 mL) was added anhydrous $NiBr_2$ (54.5 mg, 0.25 mmol) and one drop of water. The mixture was stirred at reflux overnight before the solvent was removed. The residue was triturated with toluene, followed by filtration to afford a solid. To a stirred suspension of the solid (0.2 mmol) in CH_2Cl_2 (2 mL) was added anhydrous AgOTf (102 mg, 0.4 mmol). After filtration through celite, the solvent was removed under reduced pressure to afford the expected **L5·Ni(OTf)$_2$ or L6·Ni(OTf)$_2$**.

Supplementary Table 1. Optimization of Lewis acid and Lewis base[a]

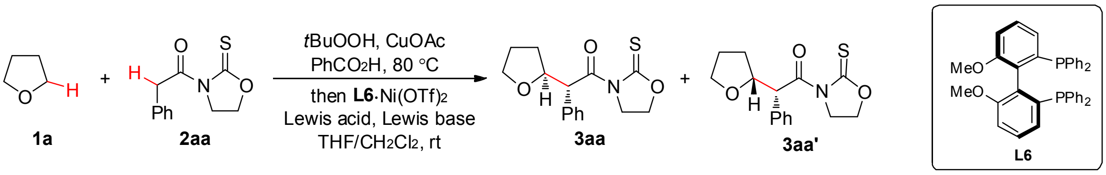

entry	Lewis acid	Lewis base	yield (%)[b]	d.r.[c]	ee (%)[d]
1	$BF_3{\cdot}OEt_2$	2,4,6-collidine	81	67:33.	98/98
2	TMSOTf	2,4,6-collidine	< 5	n.d.	n.d.
3	$BF_3{\cdot}OEt_2$	DIPEA	< 5	n.d.	n.d.
4	$BF_3{\cdot}OEt_2$	Et_3N	< 5	n.d.	n.d.
5	$BF_3{\cdot}OEt_2$	pyridine	< 5	n.d.	n.d.
6	$BF_3{\cdot}OEt_2$	2,6-lutidine	75	65:35	97/96

[a]Reaction condition: PhCOOH (0.5 mmol, 2.5 equiv), CuOAc (0.005 mmol, 2.5 mol%), and ^{t}BuOOH in decane (0.5 mmol, 2.5 equiv) in THF (1 mL) at 80 °C for 2 h, followed by addition of **2aa** (0.2 mmol, 1.0 equiv), **L6**·Ni(OTf)$_2$ (0.024 mmol, 12 mol%), Lewis base (0.6 mmol, 3.0 equiv), and Lewis acid (0.8 mmol, 4.0 equiv) in CH_2Cl_2 (0.4 mL) at rt for 2 h. [b]Isolated yield of the two diastereomers. [c]Determined by ^{1}H NMR spectroscopy. [d]Determined by chiral HPLC analysis. DIPEA: *N*,*N*-Diisopropylethylamine.

General Procedure C for Enantioselective CDC of THF with α-Aryl Acetic Acid Derivative

A solution of $PhCO_2H$ (0.5 mmol, 2.5 eq), CuOAc (0.005 mmol, 2.5 mol%), and ^{t}BuOOH in decane (0.5 mmol, 2.5 equiv) in THF (1.0 mL) was stirred at 80 ºC for 2 h. After the solvent was evaporated, **2** (0.2 mmol, 1.0 equiv), **L6**·Ni(OTf)$_2$ (0.02 mmol, 10 mol%), 2,4,6-collidine (0.6 mmol, 3.0 equiv), and $BF_3{\cdot}OEt_2$ (0.8 mmol, 4.0 equiv) in THF/CH_2Cl_2 (0.3 mL/0.1 mL) was added to the mixture at rt for 2 h. The solvent was removed and the residue was purified by silica gel chromatography to give the desired product.

General Procedure D for Enantioselective CDC of THF with α-Alkenyl Acetic

Acid Derivative

A solution of $PhCO_2H$ (0.5 mmol, 2.5 eq), CuOAc (0.005 mmol, 2.5 mol%), and ^{t}BuOOH in decane (0.5 mmol, 2.5 equiv) in THF (1.0 mL) was stired at 80 ºC for 2 h. After the solvent was evaporated, **2** (0.2 mmol, 1.0 equiv), **L5**·Ni(OTf)$_2$ (0.02 mmol, 10 mol%), 2,4,6-collidine (0.6 mmol, 3.0 equiv), and BF_3·OEt_2 (0.8 mmol, 4.0 equiv) in $CH_3CO_2CH_3/CH_2Cl_2$ (0.3 mL/0.1 mL) was added to the mixture at rt for 2 h. The solvent was removed and the residue was purified by silica gel chromatography to give the desired product.

General Procedure E for Enantioselective CDC of Other Saturated Ethers

A solution of $PhCO_2H$ (0.5 mmol, 2.5 eq), CuOAc (0.005 mmol, 2.5 mol%), and ^{t}BuOOH in decane (0.6 mmol, 3.0 equiv) in ether (1.0 mL) was stired at 80 ºC. Upon $PhCO_2H$ consumption, the solvent was evaporated. Then **2** (0.2 mmol, 1.0 equiv), **L5**·Ni(OTf)$_2$ or **L6**·Ni(OTf)$_2$ (0.02 mmol, 10 mol%), 2,4,6-collidine (0.6 mmol, 3.0 equiv), and BF_3·OEt_2 (0.8 mmol, 4.0 equiv) in CH_2Cl_2 (0.4 mL) was added to the mixture at rt for 2 h. The solvent was removed and the residue was purified by silica gel chromatography to give the desired product.

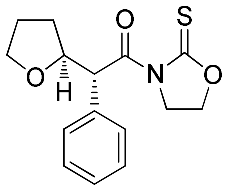

(*R*)-2-Phenyl-2-((*S*)-tetrahydrofuran-2-yl)-1-(2-thioxooxazolidin-3-yl)ethanone (3aa)

Prepared according to general procedure C and purified by silica gel chromatography (CH_2Cl_2/EtOAc 100:0 to 99:1). Yield: 81% (47.2 mg), **3aa**/**3aa'** = 2:1. ^{1}H NMR (500 MHz, $CDCl_3$) δ 7.53–7.43 (m, 2H), 7.36–7.30 (m, 2H), 7.29–7.25 (m, 1H), 6.29 (d, J = 8.3 Hz, 1H), 4.57–4.50 (m, 1H), 4.47 (td, J = 9.3, 6.5 Hz, 1H), 4.39–4.32 (m, 1H), 4.22 (ddd, J = 11.2, 9.5, 8.6 Hz, 1H), 4.11 (ddd, J = 11.3, 9.3, 6.5 Hz, 1H), 3.87–3.80 (m, 1H), 3.72 (td, J = 7.8, 6.2 Hz, 1H), 2.17–2.08 (m, 1H), 1.97–1.82 (m, 2H), 1.76–1.69 (m, 1H); ^{13}C NMR (126 MHz, $CDCl_3$) δ 185.3, 173.6, 136.1, 129.8, 128.6,

127.8, 81.1, 68.4, 66.2, 52.9, 47.6, 30.5, 25.7; IR (KBr): 3376, 2972, 2951, 2870, 1690, 1378, 1208, 1155, 1071, 1019, 700 cm^{-1}; HRMS (EI) m/z $[M + H]^+$ calculated for $C_{15}H_{18}NO_3S$: 292.1002, found 292.1006; HPLC: the ee value was determined by HPLC analysis (Chiralpak AD-H, *i*-PrOH/Hexane = 10/90, 1.0 mL/min, 273 nm), retention time: t_{major} = 16.570 min, t_{minor} = 17.733 min, ee = 98%; $[\alpha]_D^{25}$ = + 27.9 (c = 2.72, THF).

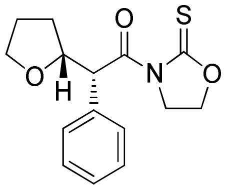

(*R*)-2-Phenyl-2-((*R*)-tetrahydrofuran-2-yl)-1-(2-thioxooxazolidin-3-yl)ethanone (3aa')

^{1}H NMR (500 MHz, $CDCl_3$) δ 7.49–7.42 (m, 2H), 7.35–7.26 (m, 3H), 5.97 (d, *J* = 9.7 Hz, 1H), 4.75–4.56 (m, 2H), 4.54–4.47 (m, 1H), 3.95–3.86 (m, 1H), 3.87–3.78 (m, 1H), 3.32–3.21 (m, 1H), 3.14 (dt, *J* = 10.9, 7.4 Hz, 1H), 1.94–1.87 (m, 1H), 1.86–1.79 (m, 1H), 1.70–1.61 (m, 1H), 1.57–1.48 (m, 1H); ^{13}C NMR (126 MHz, $CDCl_3$) δ 202.0, 174.9, 135.4, 129.6, 128.7, 127.9, 82.4, 68.6, 56.9, 54.9, 29.6, 28.3, 25.5; IR (KBr): 3377, 2973, 2952, 2872, 1695, 1377, 1210, 1157, 1065, 1018, 701cm^{-1}; HRMS (EI) m/z $[M + H]^+$ calculated for $C_{15}H_{18}NO_2S$: 292.1002, found 292.1000; HPLC: the ee value was determined by HPLC analysis (Chiralpak AD-H, *i*-PrOH/Hexane = 25/75, 1.0 mL/min, 308 nm), retention time: t_{minor} = 6.613 min, t_{major} = 8.957 min, ee = 96%; $[\alpha]_D^{27}$ = – 32.3 (c = 1.16, THF).

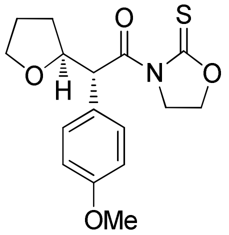

(*R*)-2-(4-Methoxyphenyl)-2-((*R*)-tetrahydrofuran-2-yl)-1-(2-thioxooxazolidin-3-yl) ethanone (3b)

Prepared according to general procedure C and purified by silica gel chromatography

(CH_2Cl_2/EtOAc 100:0 to 99:1). Yield: 68% (43.5 mg), **3b**/**3b'** = 2:1. ^{1}H NMR (500 MHz, $CDCl_3$) δ 7.45–7.37 (m, 2H), 6.92–6.83 (m, 2H), 6.22 (d, *J* = 8.2 Hz, 1H), 4.59–4.46 (m, 2H), 4.40 (q, *J* = 8.9 Hz, 1H), 4.29–4.23 (m, 1H), 4.13 (ddd, *J* = 11.3, 9.3, 6.4 Hz, 1H), 3.86–3.81 (m, 1H), 3.79 (s, 3H), 3.73 (td, *J* = 7.8, 6.3 Hz, 1H), 2.16–2.07 (m, 1H), 1.96–1.82 (m, 2H), 1.74–1.67 (m, 1H); ^{13}C NMR (126 MHz, $CDCl_3$) δ 185.4, 174.0, 159.3, 130.9, 128.1, 114.1, 81.1, 68.5, 66.2, 55.4, 52.2, 47.7, 30.5, 25.0; IR (KBr): 3376, 2938, 2915, 2837, 1708, 1611, 1389, 1155, 1072, 1018, 819 cm^{-1}; HRMS (EI) m/z $[M + H]^+$ calculated for $C_{16}H_{20}NO_4S$: 322.1108, found 292.1114; HPLC: the ee value was determined by HPLC analysis (Chiralpak AD-H, *i*-PrOH/Hexane = 25/75, 1.0 mL/min, 272 nm), retention time: t_{minor} = 9.937 min, t_{major} = 13.340 min, ee = 96%; $[\alpha]_D^{25}$ = + 11.5 (c = 0.32, THF).

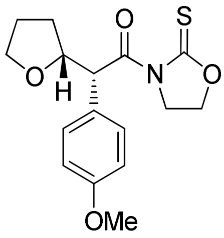

(*R*)-2-(4-Methoxyphenyl)-2-((*S*)-tetrahydrofuran-2-yl)-1-(2-thioxooxazolidin-3-yl)ethanone (3b')

^{1}H NMR (500 MHz, $CDCl_3$) δ 7.41 (d, *J* = 8.7 Hz, 2H), 6.86 (d, *J* = 8.7 Hz, 2H), 6.20 (d, *J* = 9.9 Hz, 1H), 4.63 (dt, *J* = 9.8, 6.5 Hz, 1H), 4.49 (dd, *J* = 17.0, 8.8 Hz, 1H), 4.42 (dd, *J* = 17.2, 8.3 Hz, 1H), 4.34 (ddd, *J* = 17.2, 9.5, 7.7 Hz, 1H), 4.18–4.10 (m, 1H), 3.93 (dd, *J* = 14.8, 7.2 Hz, 1H), 3.86–3.78 (m, 4H), 1.99–1.91 (m, 1H), 1.88–1.80 (m, 1H), 1.73–1.65 (m, 1H), 1.60–1.53 (m, 1H); ^{13}C NMR (126 MHz, $CDCl_3$) δ 185.6, 174.5, 159.4, 130.7, 127.2, 114.2, 81.9, 68.7, 66.2, 55.4, 52.2, 47.6, 29.5, 25.6; IR (KBr): 3377, 2933, 2914, 2839, 1713, 1613, 1387, 1156, 1066, 1017, 809 cm^{-1}; HRMS (EI) m/z $[M + H]^+$ calculated for $C_{16}H_{20}NO_4S$: 322.1108, found 292.1110; HPLC: the ee value was determined by HPLC analysis (Chiralpak AD-H, *i*-PrOH/Hexane = 25/75, 1.0 mL/min, 273 nm), retention time: t_{major} = 12.337 min, t_{minor} = 13.590 min, ee = 97%; $[\alpha]_D^{25}$ = – 37.0 (c = 0.84, THF).

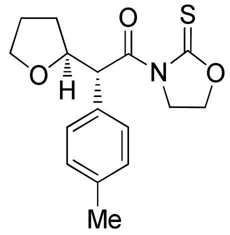

(*R*)-2-((*S*)-Tetrahydrofuran-2-yl)-1-(2-thioxooxazolidin-3-yl)-2-(*p*-tolyl)ethanone (3c)

Prepared according to general procedure C and purified by silica gel chromatography (CH_2Cl_2/EtOAc 100:0 to 99:1). Yield: 72% (43.8 mg), **3c**/**3c'** = 2.3:1. ^{1}H NMR (500 MHz, $CDCl_3$) δ 7.36 (d, *J* = 8.0 Hz, 2H), 7.15 (d, *J* = 7.9 Hz, 2H), 6.24 (d, *J* = 8.4 Hz, 1H), 4.56–4.47 (m, 2H), 4.41–4.35 (m, 1H), 4.28–4.21 (m, 1H), 4.12 (ddd, *J* = 11.2, 9.4, 6.3 Hz, 1H), 3.84 (dd, *J* = 14.9, 7.1 Hz, 1H), 3.72 (dd, *J* = 14.3, 7.7 Hz, 1H), 2.32 (s, 3H), 2.18–2.08 (m, 1H), 1.99–1.83 (m, 2H), 1.77–1.66 (m, 1H); ^{13}C NMR (126 MHz, $CDCl_3$) δ 185.3, 173.8, 137.6, 133.1, 129.6, 129.4, 81.1, 68.4, 66.2, 52.6, 47.6, 30.5, 25.7, 21.3; IR (KBr): 3367, 2982, 2931, 2843, 1691, 1378, 1210, 1155, 1068, 1019 cm^{-1}; HRMS (EI) m/z $[M + H]^+$ calculated for $C_{16}H_{20}NO_3S$: 306.1158, found 306.1158; HPLC: the ee value was determined by HPLC analysis (Chiralcel OD-H, *i*-PrOH/Hexane = 25/75, 1.0 mL/min, 265 nm), retention time: t_{minor} = 11.877 min, t_{major} = 25.847 min, ee = 93%; $[\alpha]_D^{25}$ = + 20.8 (c = 0.7, THF).

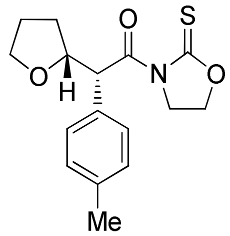

(*R*)-2-((*R*)-Tetrahydrofuran-2-yl)-1-(2-thioxooxazolidin-3-yl)-2-(*p*-tolyl)ethanone (3c')

^{1}H NMR (500 MHz, $CDCl_3$) δ 7.38 (d, *J* = 8.0 Hz, 2H), 7.14 (d, *J* = 7.9 Hz, 2H), 6.23 (d, *J* = 9.9 Hz, 1H), 4.65 (dt, *J* = 9.8, 6.5 Hz, 1H), 4.54–4.46 (m, 1H), 4.42 (dt, *J* = 16.4, 8.3 Hz, 1H), 4.38–4.30 (m, 1H), 4.18–4.10 (m, 1H), 3.93 (dd, *J* = 14.8, 7.2 Hz, 1H), 3.83 (dd, *J* = 13.9, 7.8 Hz, 1H), 2.33 (s, 3H), 2.00 – 1.91 (m, 1H), 1.88–1.80 (m, 1H), 1.73–1.67 (m, 1H), 1.61–1.54 (m, 1H); ^{13}C NMR (126 MHz, $CDCl_3$) δ 185.6,

174.4, 137.8, 132.2, 129.6, 129.5, 81.9, 68.7, 66.2, 52.7, 47.6, 29.5, 25.6, 21.3; IR (KBr): 3368, 2983, 2930, 2845, 1695, 1376, 1213, 1155, 1065, 1018 cm^{-1}; HRMS (EI) m/z $[M + H]^+$ calculated for $C_{16}H_{20}NO_3S$: 306.1158, found 306.1161; HPLC: the ee value was determined by HPLC analysis (Chiralpak AD-H, *i*-PrOH/Hexane = 25/75, 1.0 mL/min, 266 nm), retention time: t_{minor} = 7.387 min, t_{major} = 9.340 min, ee = 96%; $[\alpha]_D^{25}$ = – 35.7 (c = 0.46, THF).

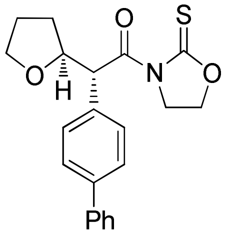

(*R*)-2-([1,1'-Biphenyl]-4-yl)-2-((*S*)-tetrahydrofuran-2-yl)-1-(2-thioxooxazolidin-3-yl)ethanone (3d)

Prepared according to general procedure C and purified by silica gel chromatography (CH_2Cl_2/EtOAc 100:0 to 99:1). Yield: 80% (58.8 mg), **3d/3d'** = 2.4:1. ^{1}H NMR (500 MHz, $CDCl_3$) δ 7.64–7.51 (m, 6H), 7.47–7.40 (m, 2H), 7.34 (t, *J* = 7.4 Hz, 1H), 6.36 (d, *J* = 8.4 Hz, 1H), 4.60 (dt, *J* = 8.1, 6.7 Hz, 1H), 4.52 (td, *J* = 9.3, 6.5 Hz, 1H), 4.41 (q, *J* = 9.0 Hz, 1H), 4.32–4.24 (m, 1H), 4.16 (ddd, *J* = 11.3, 9.3, 6.5 Hz, 1H), 3.88 (dt, *J* = 13.9, 6.9 Hz, 1H), 3.77 (td, *J* = 7.8, 6.3 Hz, 1H), 2.21–2.12 (m, 1H), 2.01–1.87 (m, 2H), 1.75 (ddt, *J* = 12.5, 8.5, 7.0 Hz, 1H); ^{13}C NMR (126 MHz, $CDCl_3$) δ 185.4, 173.7, 141.0, 140.7, 135.2, 130.2, 128.9, 127.4, 127.3, 81.2, 68.5, 66.2, 52.7, 47.7, 30.6, 25.8; IR (KBr): 3369, 2987, 2913, 2855, 1689, 1383, 1247, 1182, 1022 cm^{-1}; HRMS (EI) m/z $[M + H]^+$ calculated for $C_{21}H_{22}NO_3S$: 368.1315, found 368.1318; HPLC: the ee value was determined by HPLC analysis (Chiralcel OD-H, *i*-PrOH/Hexane = 35/65, 1.0 mL/min, 262 nm), retention time: t_{minor} = 19.340 min, t_{major} = 26.113 min, ee = 95%; $[\alpha]_D^{28}$ = + 32.4 (c = 0.82, THF).

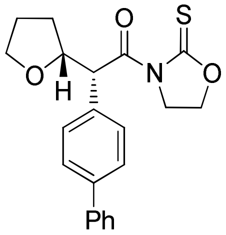

(*R*)-2-([1,1'-Biphenyl]-4-yl)-2-((*R*)-tetrahydrofuran-2-yl)-1-(2-thioxooxazolidin-3-yl)ethanone (3d')

^{1}H NMR (500 MHz, $CDCl_3$) δ 7.64–7.51 (m, 6H), 7.44 (t, J = 7.6 Hz, 2H), 7.35 (t, J = 7.4 Hz, 1H), 6.34 (d, J = 9.8 Hz, 1H), 4.72 (dt, J = 9.8, 6.5 Hz, 1H), 4.55–4.33 (m, 3H), 4.18 (ddd, J = 11.2, 9.3, 7.6 Hz, 1H), 3.96 (dd, J = 14.9, 7.1 Hz, 1H), 3.86 (dt, J = 14.0, 7.0 Hz, 1H), 2.03–1.95 (m, 1H), 1.92–1.83 (m, 1H), 1.79–1.71 (m, 1H), 1.66–1.59 (m, 1H); ^{13}C NMR (126 MHz, $CDCl_3$) δ 185.7, 174.2, 140.9, 140.7, 134.2, 130.1, 129.0, 127.6, 127.5, 127.2, 82.0, 68.8, 66.3, 52.7, 47.7, 29.6, 25.7; IR (KBr): 3368, 2986, 2911, 2850, 1694, 1382, 1245, 1180, 1021 cm^{-1}; HRMS (EI) m/z $[M + H]^+$ calculated for $C_{21}H_{22}NO_3S$: 368.1315, found 368.1322; HPLC: the ee value was determined by HPLC analysis (Chiralpak AD-H, *i*-PrOH/Hexane = 5/95, 1.0 mL/min, 262 nm), retention time: t_{minor} = 44.183 min, t_{major} = 49.360 min, ee = 96%; $[\alpha]_D^{28}$ = –22.2 (c = 0.56, THF).

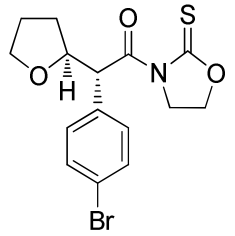

(*R*)-2-(4-Bromophenyl)-2-((*S*)-tetrahydrofuran-2-yl)-1-(2-thioxooxazolidin-3-yl) ethanone (3e)

Prepared according to general procedure C and purified by silica gel chromatography (CH_2Cl_2/EtOAc 100:0 to 99:1). Yield: 82% (60.5 mg), **3e**/**3e'** = 2.5:1. ^{1}H NMR (500 MHz, $CDCl_3$) δ 7.46 (d, J = 8.5 Hz, 2H), 7.36 (d, J = 8.5 Hz, 2H), 6.25 (d, J = 8.1 Hz, 1H), 4.58–4.48 (m, 2H), 4.44 (dd, J = 17.5, 9.1 Hz, 1H), 4.27 (ddd, J = 11.3, 9.5, 8.4 Hz, 1H), 4.15 (ddd, J = 11.3, 9.3, 6.7 Hz, 1H), 3.86–3.80 (m, 1H), 3.73 (dd, J = 14.3, 7.6 Hz, 1H), 2.18–2.09 (m, 1H), 1.95–1.87 (m, 2H), 1.72–1.66 (m, 1H); ^{13}C NMR (126 MHz, $CDCl_3$) δ 185.3, 173.3, 135.1, 131.8, 131.6, 122.2, 81.0, 68.6, 66.3, 52.5, 47.7, 30.5, 25.8; IR (KBr): 3386, 2991, 2951, 2909, 1692, 1404, 1368, 1221, 1017, 790 cm^{-1}; HRMS (EI) m/z $[M + H]^+$ calculated for $C_{15}H_{17}BrNO_3S$: 370.0107, found 370.1110; HPLC: the ee value was determined by HPLC analysis (Chiralpak AD-H,

i-PrOH/Hexane = 25/75, 1.0 mL/min, 267 nm), retention time: t_{major} = 9.680 min, t_{minor} = 10.383 min, ee = 96%; $[\alpha]_D^{25}$ = + 21.6 (c = 0.12, THF).

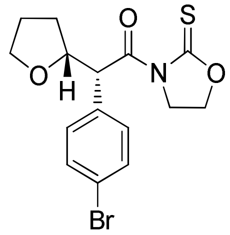

(*R*)-2-(4-Bromophenyl)-2-((*R*)-tetrahydrofuran-2-yl)-1-(2-thioxooxazolidin-3-yl) ethanone (3e')

^{1}H NMR (500 MHz, $CDCl_3$) δ 7.46 (d, *J* = 8.5 Hz, 2H), 7.39 (d, *J* = 8.5 Hz, 2H), 6.24 (d, *J* = 9.8 Hz, 1H), 4.62 (dt, *J* = 9.7, 6.5 Hz, 1H), 4.56–4.43 (m, 2H), 4.36 (ddd, *J* = 11.2, 9.6, 7.1 Hz, 1H), 4.16 (ddd, *J* = 11.3, 9.4, 8.0 Hz, 1H), 3.92 (dd, *J* = 15.0, 7.1 Hz, 1H), 3.83 (td, *J* = 7.8, 6.1 Hz, 1H), 1.99–1.90 (m, 1H), 1.90–1.75 (m, 1H), 1.75–1.67 (m, 1H), 1.57–1.49 (m, 1H); ^{13}C NMR (126 MHz, $CDCl_3$) δ 185.7, 173.9, 134.2, 132.0, 131.4, 122.3, 81.9, 68.9, 66.4, 52.5, 47.6, 29.6, 25.6; IR (KBr): 3385, 2993, 2952, 2910, 1695, 1403, 1365, 1222, 1019, 787 cm^{-1}; HRMS (EI) m/z $[M + H]^+$ calculated for $C_{15}H_{17}BrNO_3S$: 370.0107, found 370.1109; HPLC: the ee value was determined by HPLC analysis (Chiralpak AD-H, *i*-PrOH/Hexane = 25/75, 1.0 mL/min, 266 nm), retention time: t_{minor} = 8.700 min, t_{major} = 10.000 min, ee = 97%; $[\alpha]_D^{25}$ = – 17.2 (c = 0.18, THF).

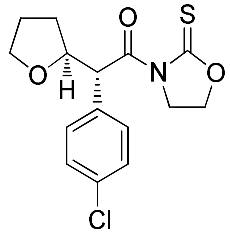

(*R*)-2-(4-Chlorophenyl)-2-((*S*)-tetrahydrofuran-2-yl)-1-(2-thioxooxazolidin-3-yl) ethanone (3f)

Prepared according to general procedure C and purified by silica gel chromatography (CH_2Cl_2/EtOAc 100:0 to 99:1). Yield: 83% (54.0 mg), **3f**/**3f'** = 2.4:1. ^{1}H NMR (500 MHz, $CDCl_3$) δ 7.42 (d, *J* = 8.5 Hz, 2H), 7.31 (d, *J* = 8.5 Hz, 2H), 6.26 (d, *J* = 8.1 Hz, 1H), 4.59–4.48 (m, 2H), 4.43 (dd, *J* = 17.5, 9.1 Hz, 1H), 4.26 (ddd, *J* = 11.3, 9.5, 8.3

Hz, 1H), 4.15 (ddd, J = 11.4, 9.4, 6.8 Hz, 1H), 3.87–3.80 (m, 1H), 3.73 (dd, J = 14.4, 7.6 Hz, 1H), 2.17–2.08 (m, 1H), 1.95–1.84 (m, 2H), 1.72–1.64 (m, 1H); ^{13}C NMR (126 MHz, $CDCl_3$) δ 185.3, 173.4, 134.5, 133.9, 131.2, 128.8, 81.0, 68.5, 66.3, 52.4, 47.6, 30.5, 25.8; IR (KBr): 3398, 2977, 2955, 2920, 1690, 1403, 1363, 1016, 799 cm^{-1}; HRMS (EI) m/z [M + H]$^+$ calculated for $C_{15}H_{17}ClNO_3S$: 326.0621, found 326.0621; HPLC: the ee value was determined by HPLC analysis (Chiralpak AS-H, *i*-PrOH/Hexane = 25/75, 1.0 mL/min, 266 nm), retention time: t_{minor} = 14.480 min, t_{major} = 15.520 min, ee = 97%; $[\alpha]_D^{26}$ = + 35.8 (c = 0.48, THF).

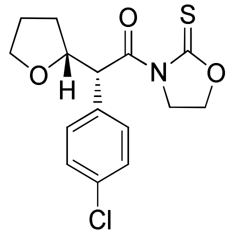

(*R*)-2-(4-Chlorophenyl)-2-((*S*)-tetrahydrofuran-2-yl)-1-(2-thioxooxazolidin-3-yl) ethanone (3f')

^{1}H NMR (500 MHz, $CDCl_3$) δ 7.45 (d, J = 8.5 Hz, 2H), 7.30 (d, J = 8.4 Hz, 2H), 6.25 (d, J = 9.8 Hz, 1H), 4.62 (dt, J = 9.7, 6.5 Hz, 1H), 4.55–4.42 (m, 2H), 4.40–4.31 (m, 1H), 4.16 (ddd, J = 11.3, 9.3, 8.0 Hz, 1H), 3.92 (dd, J = 15.0, 7.1 Hz, 1H), 3.83 (dd, J = 13.8, 7.8 Hz, 1H), 1.99–1.91 (m, 1H), 1.89–1.80 (m, 1H), 1.74–1.67 (m, 1H), 1.58–1.51 (m, 1H); ^{13}C NMR (126 MHz, $CDCl_3$) δ 185.7, 174.0, 134.0, 133.7, 131.0, 129.0, 81.9, 68.8, 66.4, 52.4, 47.6, 29.6, 25.6; IR (KBr): 3399, 2975, 2953, 2923, 1694, 1405, 1364, 1018, 794 cm^{-1}; HRMS (EI) m/z [M + H]$^+$ calculated for $C_{15}H_{17}ClNO_3S$: 326.0621, found 326.0620; HPLC: the ee value was determined by HPLC analysis (Chiralpak AD-H, *i*-PrOH/Hexane = 25/75, 1.0 mL/min, 268 nm), retention time: t_{minor} = 7.967 min, t_{major} = 9.340 min, ee = 96%; $[\alpha]_D^{26}$ = – 57.1 (c = 0.52, THF).

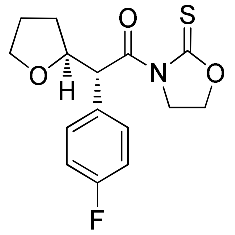

(*R*)-2-(4-Fluorophenyl)-2-((*S*)-tetrahydrofuran-2-yl)-1-(2-thioxooxazolidin-3-yl)ethanone (3g)

Prepared according to general procedure C and purified by silica gel chromatography (CH_2Cl_2/EtOAc 100:0 to 99:1). Yield: 85% (52.0 mg), **3g**/**3g'** = 2.6:1. ^{1}H NMR (500 MHz, $CDCl_3$) δ 7.46 (dd, *J* = 8.6, 5.5 Hz, 2H), 7.02 (t, *J* = 8.7 Hz, 2H), 6.27 (d, *J* = 8.1 Hz, 1H), 4.57–4.48 (m, 2H), 4.43 (dd, *J* = 17.6, 9.0 Hz, 1H), 4.32–4.23 (m, 1H), 4.15 (ddd, *J* = 11.3, 9.4, 6.8 Hz, 1H), 3.83 (dd, *J* = 15.1, 6.9 Hz, 1H), 3.73 (dd, *J* = 14.5, 7.4 Hz, 1H), 2.17–2.08 (m, 1H), 1.95–1.83 (m, 2H), 1.73–1.63 (m, 1H); ^{13}C NMR (126 MHz, $CDCl_3$) δ 185.4, 173.6, 162.5 (d, *J* = 246.3 Hz), 131.8 (d, *J* = 3.2 Hz), 131.5 (d, *J* = 8.0 Hz), 115.5 (d, *J* = 21.3 Hz), 81.1, 68.5, 66.2, 52.1, 47.6, 30.5, 25.8; IR (KBr): 3415, 3001, 2972, 2916, 1691, 1409, 1368, 1193, 1085, 1021, 790 cm^{-1}; HRMS (EI) m/z $[M + H]^+$ calculated for $C_{15}H_{17}FNO_3S$: 310.0908, found 3310.0908; HPLC: the ee value was determined by HPLC analysis (Chiralpak AD-H, *i*-PrOH/Hexane = 25/75, 1.0 mL/min, 266 nm), retention time: t_{minor} = 8.360 min, t_{major} = 8.887 min, ee = 95%; $[\alpha]_D^{28}$ = + 29.1 (c = 0.64, THF).

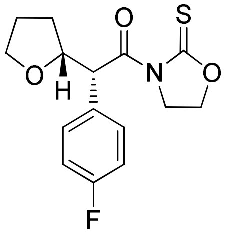

(*R*)-2-(4-Fluorophenyl)-2-((*R*)-tetrahydrofuran-2-yl)-1-(2-thioxooxazolidin-3-yl)ethanone (3g')

^{1}H NMR (500 MHz, $CDCl_3$) δ 7.48 (dd, *J* = 8.6, 5.4 Hz, 2H), 7.02 (t, *J* = 8.7 Hz, 2H), 6.26 (d, *J* = 9.8 Hz, 1H), 4.62 (dt, *J* = 9.8, 6.5 Hz, 1H), 4.55–4.42 (m, 2H), 4.36 (ddd, *J* = 11.1, 9.6, 7.2 Hz, 1H), 4.16 (ddd, *J* = 11.3, 9.4, 7.9 Hz, 1H), 3.93 (dd, *J* = 15.0, 7.1 Hz, 1H), 3.86–3.79 (m, 1H), 1.99–1.90 (m, 1H), 1.90–1.81 (m, 1H), 1.73–1.66 (m, 1H), 1.58–1.50 (m, 1H); ^{13}C NMR (126 MHz, $CDCl_3$) δ 185.7, 174.3, 162.6 (d, *J* = 246.8 Hz), 131.3 (d, *J* = 8.0 Hz), 131.0 (d, *J* = 3.2 Hz), 115.7 (d, *J* = 21.3 Hz), 82.0, 68.8, 66.3, 52.2, 47.6, 29.6, 25.6; IR (KBr): 3372, 3003, 2973, 2917, 1696, 1408, 1366, 1194, 1086, 1023, 788 cm^{-1}; HRMS (EI) m/z $[M + H]^+$ calculated for

$C_{15}H_{17}FNO_3S$: 310.0908, found 3310.0914; HPLC: the ee value was determined by HPLC analysis (Chiralpak AD-H, *i*-PrOH/Hexane = 25/75, 1.0 mL/min, 266 nm), retention time: t_{minor} = 7.317 min, t_{major} = 9.417 min, ee = 97%; $[\alpha]_D^{28}$ = – 33.0 (c = 0.59, THF).

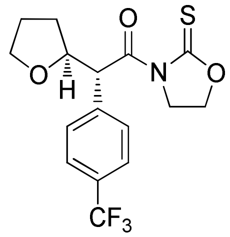

(*R*)-2-((*S*)-Tetrahydrofuran-2-yl)-1-(2-thioxooxazolidin-3-yl)-2-(4-(trifluoromethy l)phenyl)ethan-one (3h)

Prepared according to general procedure C and purified by silica gel chromatography (CH_2Cl_2/EtOAc 100:0 to 99:1). Yield: 79% (56.7 mg), **3h**/**3h'** = 2.8:1. ^{1}H NMR (500 MHz, $CDCl_3$) δ 7.70–7.52 (m, 4H), 6.37 (d, *J* = 8.2 Hz, 1H), 4.60–4.50 (m, 2H), 4.45 (dd, *J* = 17.6, 8.9 Hz, 1H), 4.33–4.24 (m, 1H), 4.17 (ddd, *J* = 11.3, 9.4, 6.9 Hz, 1H), 3.84 (dd, *J* = 15.1, 6.9 Hz, 1H), 3.74 (dd, *J* = 14.4, 7.5 Hz, 1H), 2.20–2.11 (m, 1H), 1.96–1.85 (m, 2H), 1.74–1.66 (m, 1H); ^{13}C NMR (126 MHz, $CDCl_3$) δ 185.4, 173.1, 140.1, 130.3, 130.1 (q, *J* = 32.4 Hz), 125.6 (q, *J* = 3.7 Hz), 124.4 (q, *J* = 272.1 Hz), 81.1, 68.6, 66.3, 52.9, 47.6, 30.6, 25.8; IR (KBr): 3405, 3008, 2975, 2914, 1683, 1395, 1377, 1251, 1019 cm^{-1}; HRMS (EI) m/z $[M + H]^+$ calculated for $C_{15}H_{17}F_3NO_3S$: 360.0876, found 360.0876; HPLC: the ee value was determined by HPLC analysis (Chiralpak AD-H, *i*-PrOH/Hexane = 25/75, 1.0 mL/min, 266 nm), retention time: t_{minor} = 6.660 min, t_{major} = 7.117 min, ee = 94%; $[\alpha]_D^{28}$ = + 38.1 (c = 0.46, THF).

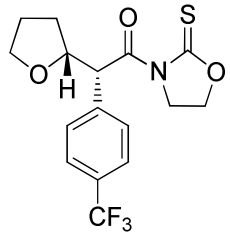

(*R*)-2-((*R*)-Tetrahydrofuran-2-yl)-1-(2-thioxooxazolidin-3-yl)-2-(4-(trifluorometh yl)phenyl)ethan-one (3h')

^{1}H NMR (500 MHz, $CDCl_3$) δ 7.65 (d, *J* = 8.2 Hz, 2H), 7.59 (d, *J* = 8.2 Hz, 2H), 6.35

(d, *J* = 9.7 Hz, 1H), 4.65 (dt, *J* = 9.7, 6.5 Hz, 1H), 4.57–4.43 (m, 2H), 4.37 (ddd, *J* = 11.2, 9.6, 7.0 Hz, 1H), 4.17 (ddd, *J* = 11.3, 9.3, 8.2 Hz, 1H), 3.93 (dd, *J* = 15.0, 7.1 Hz, 1H), 3.84 (td, *J* = 7.8, 6.1 Hz, 1H), 2.01–1.92 (m, 1H), 1.92–1.83 (m, 1H), 1.74–1.67 (m, 1H), 1.58–1.49 (m, 1H); ^{13}C NMR (126 MHz, $CDCl_3$) δ 185.7, 173.7, 139.2, 130.3 (q, *J* = 32.4 Hz), 130.1, 125.7 (q, *J* = 3.7 Hz), 124.2 (q, *J* = 272.2 Hz), 82.1, 68.9, 66.4, 52.9, 47.7, 29.7, 25.6; IR (KBr): 3408, 3007, 2976, 2913, 1688, 1399, 1378, 1252, 1018 cm^{-1}; HRMS (EI) m/z $[M + H]^+$ calculated for $C_{15}H_{17}F_3NO_3S$: 360.0876, found 360.0879; HPLC: the ee value was determined by HPLC analysis (Chiralpak AD-H, *i*-PrOH/Hexane = 25/75, 1.0 mL/min, 266 nm), retention time: t_{minor} = 6.087 min, t_{major} = 7.373 min, ee = 96%; $[\alpha]_D^{28}$ = – 15.5 (c = 0.68, THF).

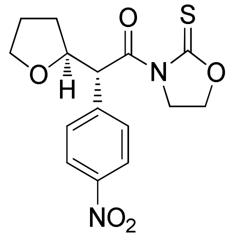

(*R*)-2-(4-Nitrophenyl)-2-((*S*)-tetrahydrofuran-2-yl)-1-(2-thioxooxazolidin-3-yl)ethanone (3i)

Prepared according to general procedure C and purified by silica gel chromatography (CH_2Cl_2/EtOAc 100:0 to 99:1). Yield: 86% (57.8 mg), **3i**/**3i'** = 3:1. ^{1}H NMR (500 MHz, $CDCl_3$) δ 8.20 (d, *J* = 8.8 Hz, 2H), 7.67 (d, *J* = 8.8 Hz, 2H), 6.40 (d, *J* = 8.2 Hz, 1H), 4.67–4.39 (m, 3H), 4.30 (ddd, *J* = 11.4, 9.5, 7.8 Hz, 1H), 4.19 (ddd, *J* = 11.4, 9.4, 7.2 Hz, 1H), 3.84 (dt, *J* = 8.2, 6.8 Hz, 1H), 3.78–3.69 (m, 1H), 2.22–2.11 (m, 1H), 1.97–1.83 (m, 2H), 1.72–1.64 (m, 1H); ^{13}C NMR (126 MHz, $CDCl_3$) δ 185.3, 172.4, 147.5, 143.5, 130.9, 123.6, 81.1, 68.6, 66.4, 53.0, 47.6, 30.6, 25.7; IR (KBr): 3775, 3389, 3078, 2959, 2911, 1680, 1389, 1371, 1081, 1017, 732 cm^{-1}; HRMS (EI) m/z $[M + H]^+$ calculated for $C_{15}H_{17}N_2O_5S$: 337.0853, found 337.0851; HPLC: the ee value was determined by HPLC analysis (Chiralpak AS-H, *i*-PrOH/Hexane = 25/75, 1.0 mL/min, 257 nm), retention time: t_{major} = 34.950 min, t_{minor} = 39.743 min, ee = 94%; $[\alpha]_D^{26}$ = + 58.0 (c = 0.72, THF).

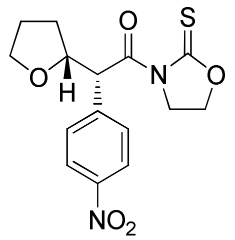

(*R*)-2-(4-Nitrophenyl)-2-((*R*)-tetrahydrofuran-2-yl)-1-(2-thioxooxazolidin-3-yl)ethanone (3i')

^{1}H NMR (500 MHz, $CDCl_3$) δ 8.20 (d, *J* = 8.8 Hz, 2H), 7.71 (d, *J* = 8.8 Hz, 2H), 6.41 (d, *J* = 9.6 Hz, 1H), 4.65 (dt, *J* = 9.6, 6.6 Hz, 1H), 4.58–4.46 (m, 2H), 4.38 (ddd, *J* = 11.3, 9.5, 6.7 Hz, 1H), 4.19 (ddd, *J* = 11.4, 9.3, 8.5 Hz, 1H), 3.98–3.90 (m, 1H), 3.84 (td, *J* = 7.9, 5.9 Hz, 1H), 2.00–1.92 (m, 1H), 1.92–1.83 (m, 1H), 1.76–1.68 (m, 1H), 1.56–1.49 (m, 1H); ^{13}C NMR (126 MHz, $CDCl_3$) δ 185.7, 173.2, 147.7, 142.4, 130.7, 123.9, 82.1, 68.9, 66.5, 53.0, 47.7, 29.7, 25.7; IR (KBr): 3774, 3391, 3080, 2958, 2910, 1684, 1388, 1372, 1083, 1016, 737 cm^{-1}; HRMS (EI) m/z $[M + H]^+$ calculated for $C_{15}H_{17}N_2O_5S$: 337.0853, found 337.0852; HPLC: the ee value was determined by HPLC analysis (Chiralpak AD-H, *i*-PrOH/Hexane = 25/75, 1.0 mL/min, 267 nm), retention time: t_{minor} = 14.037 min, t_{major} = 18.397 min, ee = 94%; $[\alpha]_D^{26}$ = – 2.5 (c = 0.68, THF).

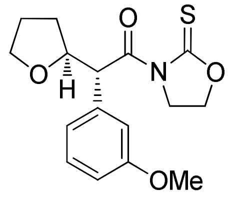

(*R*)-2-(3-Methoxyphenyl)-2-((*S*)-tetrahydrofuran-2-yl)-1-(2-thioxooxazolidin-3-yl)ethanone (3j)

Prepared according to general procedure C and purified by silica gel chromatography (CH_2Cl_2/EtOAc 100:0 to 99:1). Yield: 65% (41.7 mg), **3j**/**3j'** = 2:1. ^{1}H NMR (500 MHz, $CDCl_3$) δ 7.24 (t, *J* = 7.9 Hz, 1H), 7.10–7.00 (m, 2H), 6.86–6.78 (m, 1H), 6.28 (d, *J* = 8.4 Hz, 1H), 4.61–4.45 (m, 2H), 4.39 (q, *J* = 8.9 Hz, 1H), 4.27–4.20 (m, 1H), 4.12 (ddd, *J* = 11.3, 9.4, 6.4 Hz, 1H), 3.88–3.82 (m, 1H), 3.80 (s, 3H), 3.74 (td, *J* = 7.9, 6.2 Hz, 1H), 2.17–2.08 (m, 1H), 1.99–1.83 (m, 2H), 1.77–1.69 (m, 1H); ^{13}C NMR (126 MHz, $CDCl_3$) δ 185.4, 173.5, 159.7, 137.6, 129.5, 122.1, 115.4, 113.3, 81.1, 68.5,

66.2, 55.4, 52.8, 47.6, 30.5, 25.7; IR (KBr): 3371, 2912, 2913, 2837, 1705, 1391, 1248, 1083, 1017, 710 cm^{-1}; HRMS (EI) m/z $[M + H]^+$ calculated for $C_{16}H_{20}NO_4S$: 322.1108, found 292.1107; HPLC: the ee value was determined by HPLC analysis (Chiralpak AD-H, *i*-PrOH/Hexane = 25/75, 1.0 mL/min, 266 nm), retention time: t_{major} = 9.340 min, t_{minor} = 12.963 min, ee = 94%; $[\alpha]_D^{25}$ = + 17.5 (c = 1.20, THF).

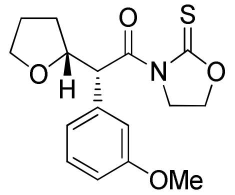

(*R*)-2-(3-Methoxyphenyl)-2-((*R*)-tetrahydrofuran-2-yl)-1-(2-thioxooxazolidin-3-yl) ethanone (3j')

^{1}H NMR (500 MHz, $CDCl_3$) δ 7.23 (t, *J* = 8.1 Hz, 1H), 7.11–7.06 (m, 2H), 6.84–6.81 (m, 1H), 6.25 (d, *J* = 9.8 Hz, 1H), 4.66 (dt, *J* = 9.8, 6.4 Hz, 1H), 4.53–4.46 (m, 1H), 4.42 (dt, *J* = 16.4, 8.3 Hz, 1H), 4.33 (ddd, *J* = 11.1, 9.6, 7.5 Hz, 1H), 4.15 (ddd, *J* = 11.2, 9.3, 7.5 Hz, 1H), 3.93 (dd, *J* = 15.0, 7.0 Hz, 1H), 3.84–3.79 (m, 4H), 1.99–1.92 (m, 1H), 1.87–1.81 (m, 1H), 1.74–1.68 (m, 1H), 1.61–1.56 (m, 1H); ^{13}C NMR (126 MHz, $CDCl_3$) δ 185.6, 174.1, 159.8, 136.6, 129.7, 122.0, 115.3, 113.4, 81.9, 68.7, 66.2, 55.4, 52.9, 47.6, 29.5, 25.6; IR (KBr): 3372, 2914, 2914, 2838, 1711, 1391, 1247, 1084, 1016, 709 cm^{-1}; HRMS (EI) m/z $[M + H]^+$ calculated for $C_{16}H_{20}NO_4S$: 322.1108, found 292.1105; HPLC: the ee value was determined by HPLC analysis (Chiralpak AD-H, *i*-PrOH/Hexane = 25/75, 1.0 mL/min, 266 nm), retention time: t_{minor} = 8.730 min, t_{major} = 15.307 min, ee = 95%; $[\alpha]_D^{25}$ = – 45.4 (c = 1.12, THF).

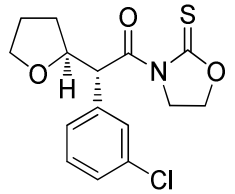

(*R*)-2-(3-Chlorophenyl)-2-((*S*)-tetrahydrofuran-2-yl)-1-(2-thioxooxazolidin-3-yl) ethanone (3k)

Prepared according to general procedure C and purified by silica gel chromatography (CH_2Cl_2/EtOAc 100:0 to 99:1). Yield: 83% (54.0 mg), **3k**/**3k'** = 2.7:1. ^{1}H NMR (500

MHz, $CDCl_3$) δ 7.47 (s, 1H), 7.41–7.34 (m, 1H), 7.28–7.24 (m, 2H), 6.29 (d, *J* = 8.2 Hz, 1H), 4.58–4.48 (m, 2H), 4.43 (dd, *J* = 17.5, 9.1 Hz, 1H), 4.26 (ddd, *J* = 11.2, 9.4, 8.4 Hz, 1H), 4.16 (ddd, *J* = 11.4, 9.4, 6.8 Hz, 1H), 3.84 (dd, *J* = 15.1, 6.9 Hz, 1H), 3.74 (dd, *J* = 14.2, 7.7 Hz, 1H), 2.18–2.08 (m, 1H), 1.97–1.85 (m, 2H), 1.75–1.65 (m, 1H); ^{13}C NMR (126 MHz, $CDCl_3$) δ 185.4, 173.1, 138.0, 134.4, 129.8, 129.7, 128.4, 128.1, 81.0, 68.5, 66.3, 52.5, 47.6, 30.5, 25.7; IR (KBr): 3395, 2975, 2955, 2917, 1690, 1413, 1368, 1017, 719 cm^{-1}; HRMS (EI) m/z $[M + H]^+$ calculated for $C_{15}H_{17}ClNO_3S$: 326.0621, found 326.0618; HPLC: the ee value was determined by HPLC analysis (Chiralpak AD-H, *i*-PrOH/Hexane = 25/75, 1.0 mL/min, 276 nm), retention time: t_{major} = 7.957 min, t_{minor} = 8.493 min, ee = 99%; $[\alpha]_D^{26}$ = + 33.5 (c = 0.97, THF).

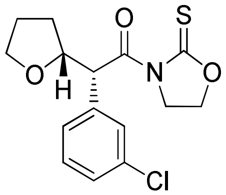

(*R*)-2-(3-Chlorophenyl)-2-((*R*)-tetrahydrofuran-2-yl)-1-(2-thioxooxazolidin-3-yl) ethanone (3k')

^1H NMR (500 MHz, $CDCl_3$) δ 7.51 (s, 1H), 7.44–7.37 (m, 1H), 7.29–7.24 (m, 2H), 6.27 (d, *J* = 9.8 Hz, 1H), 4.63 (dt, *J* = 9.8, 6.5 Hz, 1H), 4.55–4.44 (m, 2H), 4.36 (ddd, *J* = 11.2, 9.6, 7.2 Hz, 1H), 4.18 (ddd, *J* = 11.3, 9.3, 8.0 Hz, 1H), 3.93 (dd, *J* = 14.9, 7.1 Hz, 1H), 3.87–3.80 (m, 1H), 2.01–1.91 (m, 1H), 1.91–1.81 (m, 1H), 1.77–1.69 (m, 1H), 1.60–1.51 (m, 1H); ^{13}C NMR (126 MHz, $CDCl_3$) δ 185.7, 173.7, 137.1, 134.6, 130.0, 129.5, 128.3, 128.1, 82.0, 68.8, 66.4, 52.6, 47.6, 29.6, 25.6; IR (KBr): 3397, 2976, 2956, 2919, 1694, 1412, 1369, 1017, 721 cm^{-1}; HRMS (EI) m/z $[M + H]^+$ calculated for $C_{15}H_{17}ClNO_3S$: 326.0621, found 326.0621; HPLC: the ee value was determined by HPLC analysis (Chiralpak AD-H, *i*-PrOH/Hexane = 25/75, 1.0 mL/min, 276 nm), retention time: t_{minor} = 7.313 min, t_{major} = 11.153 min, ee = 98%; $[\alpha]_D^{26}$ = – 28.1 (c = 0.76, THF).

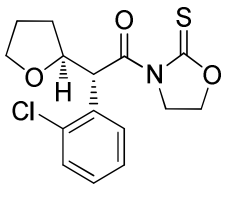

(*R*)-2-(2-Chlorophenyl)-2-((*S*)-tetrahydrofuran-2-yl)-1-(2-thioxooxazolidin-3-yl) ethanone (3l)

Prepared according to general procedure C and purified by silica gel chromatography (CH_2Cl_2/EtOAc 100:0 to 99:1). Yield: 81% (52.6 mg), **3l/3l'** = 3:1. ^{1}H NMR (500 MHz, $CDCl_3$) δ 7.69 (dd, *J* = 7.6, 1.9 Hz, 1H), 7.39 (dd, *J* = 7.7, 1.6 Hz, 1H), 7.27–7.20 (m, 2H), 6.58 (d, *J* = 6.3 Hz, 1H), 4.65 (q, *J* = 6.6 Hz, 1H), 4.57–4.43 (m, 2H), 4.30–4.18 (m, 2H), 3.85 (dd, *J* = 14.7, 6.7 Hz, 1H), 3.78–3.72 (m, 1H), 2.08–2.02 (m, 1H), 1.90–1.75 (m, 3H); ^{13}C NMR (126 MHz, $CDCl_3$) δ 185.1, 172.9, 135.7, 133.5, 131.3, 129.8, 128.9, 126.9, 80.5, 69.0, 66.4, 49.5, 47.9, 29.7, 25.9; IR (KBr): 3411, 2979, 2955, 2922, 1691, 1407, 1370, 1016, 760 cm^{-1}; HRMS (EI) m/z $[M + H]^+$ calculated for $C_{15}H_{17}ClNO_3S$: 326.0621, found 326.0625; HPLC: the ee value was determined by HPLC analysis (Chiralpak AS-H, *i*-PrOH/Hexane = 25/75, 1.0 mL/min, 266 nm), retention time: t_{minor} = 15.583 min, t_{major} = 19.533 min, ee = 97%; $[\alpha]_D^{26}$ = + 18.7 (c = 0.66, THF).

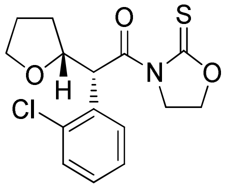

(*R*)-2-(2-Chlorophenyl)-2-((*R*)-tetrahydrofuran-2-yl)-1-(2-thioxooxazolidin-3-yl) ethanone (3l')

^{1}H NMR (500 MHz, $CDCl_3$) δ 7.69 (dd, *J* = 7.7, 1.9 Hz, 1H), 7.40 (dd, *J* = 7.7, 1.6 Hz, 1H), 7.27–7.20 (m, 2H), 6.60 (d, *J* = 9.6 Hz, 1H), 4.64 (dt, *J* = 9.6, 6.0 Hz, 1H), 4.53 (td, *J* = 9.2, 6.7 Hz, 1H), 4.44 (dd, *J* = 17.2, 9.0 Hz, 1H), 4.33 (ddd, *J* = 11.1, 9.4, 8.2 Hz, 1H), 4.19 (ddd, *J* = 11.2, 9.2, 6.7 Hz, 1H), 4.03 (td, *J* = 7.7, 5.7 Hz, 1H), 3.83–3.79 (m, 1H), 2.14–2.06 (m, 1H), 1.92–1.84 (m, 1H), 1.83–1.74 (m, 2H); ^{13}C NMR (126 MHz, $CDCl_3$) δ 185.1, 174.0, 135.2, 133.6, 130.4, 130.3, 129.0, 127.2,

83.1, 69.3, 66.2, 49.7, 47.9, 29.3, 25.8; IR (KBr): 3413, 2980, 2955, 2921, 1695, 1406, 1369, 1017, 762 cm^{-1}; HRMS (EI) m/z $[M + H]^+$ calculated for $C_{15}H_{17}ClNO_3S$: 326.0621, found 326.0618; HPLC: the ee value was determined by HPLC analysis (Chiralpak AD-H, *i*-PrOH/Hexane = 25/75, 1.0 mL/min, 266 nm), retention time: t_{minor} = 9.697 min, t_{major} = 14.817 min, ee = 98%; $[\alpha]_D^{26}$ = – 52.5 (c = 0.42, THF).

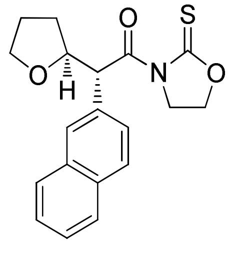

(*R*)-2-(Naphthalen-2-yl)-2-((*S*)-tetrahydrofuran-2-yl)-1-(2-thioxooxazolidin-3-yl)ethanone (3m)

Prepared according to general procedure C and purified by silica gel chromatography (CH_2Cl_2/EtOAc 100:0 to 99:1). Yield: 78% (53.2 mg), **3m/3m'** = 2.8:1. ^1H NMR (500 MHz, $CDCl_3$) δ 7.92 (s, 1H), 7.89–7.76 (m, 3H), 7.65 (dd, *J* = 8.5, 1.7 Hz, 1H), 7.52–7.42 (m, 2H), 6.46 (d, *J* = 8.2 Hz, 1H), 4.66 (dd, *J* = 14.9, 6.8 Hz, 1H), 4.50 (td, *J* = 9.2, 6.3 Hz, 1H), 4.36 (q, *J* = 8.9 Hz, 1H), 4.27 (dt, *J* = 11.1, 9.1 Hz, 1H), 4.14 (ddd, *J* = 11.2, 9.3, 6.3 Hz, 1H), 3.86 (dd, *J* = 15.1, 6.9 Hz, 1H), 3.74 (dd, *J* = 14.1, 7.8 Hz, 1H), 2.22–2.14 (m, 1H), 1.99–1.86 (m, 2H), 1.83–1.73 (m, 1H); ^{13}C NMR (126 MHz, $CDCl_3$) δ 185.4, 173.7, 133.7, 133.5, 133.1, 128.7, 128.2, 127.8, 127.8, 126.2, 81.1, 68.5, 66.2, 53.1, 47.7, 30.6, 25.8; IR (KBr): 3413, 2975, 2914, 1689, 1382, 1368, 1087, 1016 cm^{-1}; HRMS (EI) m/z $[M + H]^+$ calculated for $C_{19}H_{20}NO_3S$: 342.1158, found 342.1161; HPLC: the ee value was determined by HPLC analysis (Chiralpak AD-H, *i*-PrOH/Hexane = 25/75, 1.0 mL/min, 276 nm), retention time: t_{major} = 11.530 min, t_{minor} = 16.163 min, ee = 96%; $[\alpha]_D^{25}$ = + 46.1 (c = 0.56, THF).

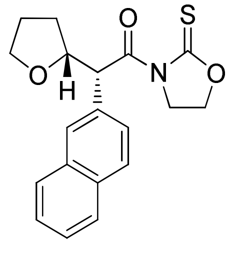

(*R*)-2-(Naphthalen-2-yl)-2-((*R*)-tetrahydrofuran-2-yl)-1-(2-thioxooxazolidin-3-yl)

ethanone (3m')

^{1}H NMR (500 MHz, $CDCl_3$) δ 7.96 (s, 1H), 7.91–7.75 (m, 3H), 7.64 (dd, *J* = 8.5, 1.6 Hz, 1H), 7.52–7.44 (m, 2H), 6.44 (d, *J* = 9.8 Hz, 1H), 4.79 (dt, *J* = 9.8, 6.5 Hz, 1H), 4.55–4.46 (m, 1H), 4.44–4.34 (m, 2H), 4.21–4.13 (m, 1H), 3.98 (dd, *J* = 15.0, 7.1 Hz, 1H), 3.87 (td, *J* = 7.8, 6.0 Hz, 1H), 2.03–1.93 (m, 1H), 1.91–1.81 (m, 1H), 1.71–1.62 (m, 2H); ^{13}C NMR (126 MHz, $CDCl_3$) δ 185.6, 174.2, 133.5, 133.0, 132.7, 128.7, 128.5, 128.2, 127.8, 127.6, 126.4, 126.4, 82.0, 68.8, 66.3, 53.2, 47.7, 29.6, 25.7; IR (KBr): 3414, 2976, 2915, 1694, 1384, 1367, 1088, 1017 cm^{-1}; HRMS (EI) m/z $[M + H]^+$ calculated for $C_{19}H_{20}NO_3S$: 342.1158, found 342.1158; HPLC: the ee value was determined by HPLC analysis (Chiralpak AD-H, *i*-PrOH/Hexane = 25/75, 1.0 mL/min, 276 nm), retention time: t_{minor} = 12.373 min, t_{major} = 14.113 min, ee = 97%; $[\alpha]_D^{25}$ = –22.2 (c = 0.48, THF).

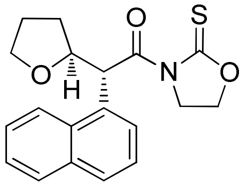

(*R*)-2-(Naphthalen-1-yl)-2-((*S*)-tetrahydrofuran-2-yl)-1-(2-thioxooxazolidin-3-yl)ethanone (3n)

Prepared according to general procedure C and purified by silica gel chromatography (CH_2Cl_2/EtOAc 100:0 to 99:1). Yield: 82% (56.0 mg), **3n**/**3n'** = 3.2:1. ^{1}H NMR (500 MHz, $CDCl_3$) δ 8.34 (d, *J* = 8.6 Hz, 1H), 7.84 (d, *J* = 8.1 Hz, 1H), 7.80 (d, *J* = 8.2 Hz, 1H), 7.70 (d, *J* = 7.2 Hz, 1H), 7.56 (t, *J* = 7.3 Hz, 1H), 7.47 (dt, *J* = 15.3, 7.6 Hz, 2H), 7.08 (d, *J* = 7.4 Hz, 1H), 4.68 (q, *J* = 6.8 Hz, 1H), 4.48 (td, *J* = 9.0, 6.3 Hz, 1H), 4.41–4.20 (m, 2H), 4.13 (ddd, *J* = 11.1, 9.1, 6.3 Hz, 1H), 3.94–3.80 (m, 1H), 3.78–3.63 (m, 1H), 2.10–1.93 (m, 3H), 1.93–1.83 (m, 1H); ^{13}C NMR (126 MHz, $CDCl_3$) δ 185.4, 173.9, 134.2, 132.9, 132.5, 128.9, 128.5, 126.4, 126.3, 125.8, 125.5, 124.8, 81.8, 68.9, 66.3, 48.3, 47.9, 30.2, 26.0; IR (KBr): 3421, 2991, 2911, 1689, 1381, 1356, 1172, 1081, 1017, 777 cm^{-1}; HRMS (EI) m/z $[M + H]^+$ calculated for $C_{19}H_{20}NO_3S$: 342.1158, found 342.1160; HPLC: the ee value was determined by HPLC analysis (Chiralpak AD-H, *i*-PrOH/Hexane = 25/75, 1.0 mL/min, 274 nm),

retention time: t_{major} = 9.860 min, t_{minor} = 12.000 min, ee = 96%; $[\alpha]_D^{26}$ = + 131.5 (c = 0.62, THF).

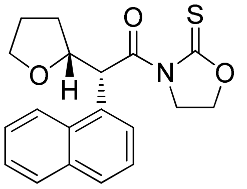

(*R*)-2-(Naphthalen-1-yl)-2-((*R*)-tetrahydrofuran-2-yl)-1-(2-thioxooxazolidin-3-yl) ethanone (3n')

^{1}H NMR (500 MHz, $CDCl_3$) δ 8.55 (d, *J* = 8.6 Hz, 1H), 7.86 (d, *J* = 8.1 Hz, 1H), 7.84–7.74 (m, 2H), 7.59 (t, *J* = 7.2 Hz, 1H), 7.50 (t, *J* = 7.4 Hz, 1H), 7.46 (t, *J* = 7.7 Hz, 1H), 7.08 (t, *J* = 10.6 Hz, 1H), 4.82 (dt, *J* = 9.4, 6.3 Hz, 1H), 4.56–4.46 (m, 1H), 4.41–4.31 (m, 2H), 4.19–4.09 (m, 1H), 4.09–4.03 (m, 1H), 3.85 (dd, *J* = 14.6, 7.4 Hz, 1H), 2.04–1.98 (m, 1H), 1.89–1.79 (m, 1H), 1.71–1.64 (m, 1H), 1.61–1.54 (m, 1H); ^{13}C NMR (126 MHz, $CDCl_3$) δ 185.8, 174.8, 134.3, 132.6, 132.0, 129.0, 128.6, 126.8, 126.6, 125.9, 125.6, 125.0, 83.4, 69.1, 66.2, 48.3, 48.0, 29.6, 25.8; IR (KBr): 3422, 2990, 2914, 1694, 1382, 1357, 1172, 1083, 1019, 775 cm^{-1}; HRMS (EI) m/z $[M + H]^+$ calculated for $C_{19}H_{20}NO_3S$: 342.1158, found 342.1163; HPLC: the ee value was determined by HPLC analysis (Chiralpak AD-H, *i*-PrOH/Hexane = 25/75, 1.0 mL/min, 275 nm), retention time: t_{minor} = 9.397 min, t_{major} = 16.050 min, ee = 97%; $[\alpha]_D^{26}$ = – 14.6 (c = 0.56, THF).

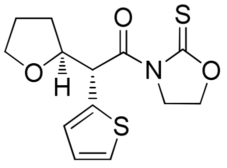

(*R*)-2-((*S*)-Tetrahydrofuran-2-yl)-2-(thiophen-2-yl)-1-(2-thioxooxazolidin-3-yl) ethanone (3o)

Prepared according to general procedure C and purified by silica gel chromatography (CH_2Cl_2/EtOAc 100:0 to 99:1). Yield: 66% (39.2 mg), **3o**/**3o'** = 2.3:1. ^{1}H NMR (500 MHz, $CDCl_3$) δ 7.29 (d, *J* = 5.0 Hz, 1H), 7.10 (d, *J* = 3.2 Hz, 1H), 6.98 (dd, *J* = 5.0, 3.7 Hz, 1H), 6.73 (d, *J* = 6.2 Hz, 1H), 4.59–4.46 (m, 3H), 4.32–4.18 (m, 2H), 3.87 (dd, *J* = 14.9, 7.1 Hz, 1H), 3.78 (dd, *J* = 13.9, 7.7 Hz, 1H), 2.13–2.05 (m, 1H), 1.90–1.77

(m, 2H), 1.74–1.65 (m, 1H); ^{13}C NMR (126 MHz, $CDCl_3$) δ 185.5, 172.8, 137.0, 127.8, 126.4, 126.3, 80.7, 68.9, 66.4, 48.4, 47.6, 29.9, 25.7; IR (KBr): 3415, 2987, 2965, 1693, 1369, 1276, 1018, 701 cm^{-1}; HRMS (EI) m/z $[M + H]^+$ calculated for $C_{13}H_{16}NO_3S_2$: 298.0566, found 298.0570; HPLC: the ee value was determined by HPLC analysis (Chiralpak AD-H, *i*-PrOH/Hexane = 25/75, 1.0 mL/min, 267 nm), retention time: t_{minor} = 8.623 min, t_{major} = 9.130 min, ee = 93%; $[\alpha]_D^{26}$ = + 19.5 (c = 0.49, THF).

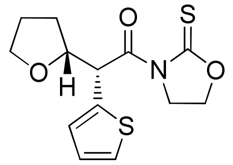

(*R*)-2-((*R*)-Tetrahydrofuran-2-yl)-2-(thiophen-2-yl)-1-(2-thioxooxazolidin-3-yl) ethanone (3o')

^{1}H NMR (500 MHz, $CDCl_3$) δ 7.26 (d, *J* = 5.2 Hz, 1H), 7.14 (d, *J* = 3.2 Hz, 1H), 7.01–6.94 (m, 1H), 6.69 (d, *J* = 9.7 Hz, 1H), 4.69–4.62 (m, 1H), 4.56–4.46 (m, 2H), 4.40–4.32 (m, 1H), 4.24–4.16 (m, 1H), 3.93 (dd, *J* = 13.9, 7.3 Hz, 1H), 3.83 (dd, *J* = 14.2, 7.1 Hz, 1H), 2.02–1.94 (m, 1H), 1.91–1.81 (m, 2H), 1.71–1.64 (m, 1H); ^{13}C NMR (126 MHz, $CDCl_3$) δ 185.8, 173.9, 137.2, 127.3, 126.8, 125.9, 82.5, 69.0, 66.4, 48.7, 47.5, 29.1, 25.6; IR (KBr): 3414, 3989, 2965, 1697, 1370, 1277, 1018, 702 cm^{-1}; HRMS (EI) m/z $[M + H]^+$ calculated for $C_{13}H_{16}NO_3S_2$: 298.0566, found 298.0570; HPLC: the ee value was determined by HPLC analysis (Chiralpak AD-H, *i*-PrOH/Hexane = 25/75, 1.0 mL/min, 267 nm), retention time: t_{minor} = 8.557 min, t_{major} = 11.437 min, ee = 92%; $[\alpha]_D^{26}$ = – 38.0 (c = 0.56, THF).

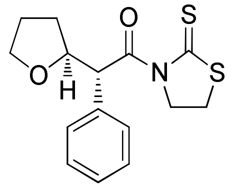

(*R*)-2-Phenyl-2-((*S*)-tetrahydrofuran-2-yl)-1-(2-thioxothiazolidin-3-yl)ethanone (3p)

Prepared according to general procedure C and purified by silica gel chromatography (CH_2Cl_2/EtOAc 100:0 to 99:1). Yield: 77% (47.3 mg), **3p**/**3p'** = 2:1. ^{1}H NMR (500

MHz, $CDCl_3$) δ 7.45–7.37 (m, 2H), 7.36–7.29 (m, 2H), 7.29–7.25 (m, 1H), 6.07 (d, *J* = 8.2 Hz, 1H), 4.61–4.41 (m, 3H), 3.83–3.76 (m, 1H), 3.75–3.66 (m, 1H), 3.17–3.08 (m, 2H), 2.18–2.08 (m, 1H), 1.92–1.82 (m, 2H), 1.76 (ddt, *J* = 12.3, 8.5, 6.9 Hz, 1H); ^{13}C NMR (126 MHz, $CDCl_3$) δ 201.8, 174.4, 136.3, 129.6, 128.6, 127.8, 81.0, 68.4, 56.9, 54.8, 30.5, 28.2, 25.7; IR (KBr): 3429, 2988, 2937, 1700, 1346, 1165, 1141, 1038, 718 cm^{-1}; HRMS (EI) m/z $[M + H]^+$ calculated for $C_{15}H_{18}NO_2S_2$: 308.0773, found 308.0776; HPLC: the ee value was determined by HPLC analysis (Chiralpak AD-H, *i*-PrOH/Hexane = 5/95, 1.0 mL/min, 307 nm), retention time: t_{major} = 15.683 min, t_{minor} = 17.537 min, ee = 96%; $[\alpha]_D^{27}$ = + 46.9 (c = 0.35, THF).

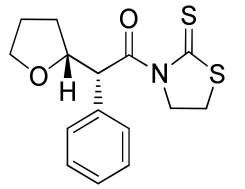

(*R*)-2-Phenyl-2-((*R*)-tetrahydrofuran-2-yl)-1-(2-thioxothiazolidin-3-yl)ethanone (3p')

^1H NMR (500 MHz, $CDCl_3$) δ 7.49–7.42 (m, 2H), 7.35–7.26 (m, 3H), 5.97 (d, *J* = 9.7 Hz, 1H), 4.75–4.56 (m, 2H), 4.54–4.47 (m, 1H), 3.95–3.86 (m, 1H), 3.87–3.78 (m, 1H), 3.32–3.21 (m, 1H), 3.14 (dt, *J* = 10.9, 7.4 Hz, 1H), 1.94–1.87 (m, 1H), 1.86–1.79 (m, 1H), 1.70–1.61 (m, 1H), 1.57–1.48 (m, 1H); ^{13}C NMR (126 MHz, $CDCl_3$) δ 202.0, 174.9, 135.4, 129.6, 128.7, 127.9, 82.4, 68.6, 56.9, 54.9, 29.6, 28.3, 25.5; IR (KBr): 3430, 2986, 2935, 1704, 1345, 1164, 1142, 1038, 715 cm^{-1}; HRMS (EI) m/z $[M + H]^+$ calculated for $C_{15}H_{18}NO_2S_2$: 308.0773, found 308.0770; HPLC: the ee value was determined by HPLC analysis (Chiralpak AD-H, *i*-PrOH/Hexane = 25/75, 1.0 mL/min, 308 nm), retention time: t_{minor} = 6.613 min, t_{major} = 8.957 min, ee = 96%; $[\alpha]_D^{27}$ = – 32.3 (c = 1.16, THF).

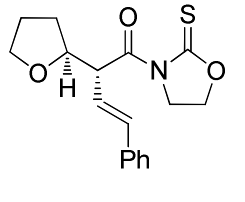

(*R*,*E*)-4-Phenyl-2-((*S*)-tetrahydrofuran-2-yl)-1-(2-thioxooxazolidin-3-yl)but-3-en-

1-one (3q)

Prepared according to general procedure D and purified by silica gel chromatography (CH_2Cl_2/EtOAc 99:1). Yield: 55% (34.9 mg), **3q/3q'** = 1.4:1. ^{1}H NMR (500 MHz, $CDCl_3$) δ 7.45–7.39 (m, 2H), 7.31 (t, *J* = 7.5 Hz, 2H), 7.26–7.22 (m, 1H), 6.61 (d, *J* = 16.1 Hz, 1H), 6.44 (dd, *J* = 16.1, 9.1 Hz, 1H), 5.79 (dd, *J* = 9.1, 5.2 Hz, 1H), 4.59–4.51 (m, 2H), 4.46 (td, *J* = 7.0, 5.3 Hz, 1H), 4.26 (ddd, *J* = 16.2, 8.1, 3.7 Hz, 2H), 3.88 (dt, *J* = 8.1, 6.8 Hz, 1H), 3.81–3.76 (m, 1H), 2.14–2.06 (m, 1H), 1.93–1.85 (m, 2H), 1.82–1.75 (m, 1H); ^{13}C NMR (126 MHz, $CDCl_3$) δ 185.6, 173.8, 136.9, 134.8, 128.7, 128.0, 126.8, 124.2, 80.0, 69.1, 66.4, 51.2, 47.6, 29.7, 25.9; IR (KBr): 3338, 3010, 2943, 1692, 1361, 1253, 1141, 1039, 742 cm^{-1}; HRMS (EI) m/z $[M + H]^+$ calculated for $C_{17}H_{20}NO_3S$: 318.1158, found 318.1160; HPLC: the ee value was determined by HPLC analysis (Chiralpak AD-H, *i*-PrOH/Hexane = 30/70, 1.0 mL/min, 260 nm), retention time: t_{minor} = 11.240 min, t_{major} = 15.497 min, ee = 93%; $[\alpha]_D^{27}$ = + 13.4 (c = 1.16, THF).

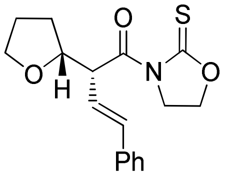

(*R*,*E*)-4-Phenyl-2-((*R*)-tetrahydrofuran-2-yl)-1-(2-thioxooxazolidin-3-yl)but-3-en-1-one (3q')

^{1}H NMR (500 MHz, $CDCl_3$) δ 7.41–7.35 (m, 2H), 7.35–7.29 (m, 2H), 7.27–7.22 (m, 1H), 6.70 (d, *J* = 16.0 Hz, 1H), 6.21 (dd, *J* = 16.0, 9.1 Hz, 1H), 5.88 (t, *J* = 9.3 Hz, 1H), 4.52 (t, *J* = 8.5 Hz, 2H), 4.48 (dt, *J* = 9.4, 6.2 Hz, 1H), 4.34 (dt, *J* = 11.3, 8.0 Hz, 1H), 4.23 (dt, *J* = 11.3, 8.8 Hz, 1H), 3.95 – 3.88 (m, 1H), 3.81 (dt, *J* = 14.0, 7.1 Hz, 1H), 2.05–1.96 (m, 2H), 1.94–1.86 (m, 1H), 1.84–1.76 (m, 1H); ^{13}C NMR (126 MHz, $CDCl_3$) δ 185.8, 174.4, 136.7, 135.0, 128.8, 128.2, 126.7, 124.1, 80.9, 68.8, 66.4, 51.6, 47.5, 29.6, 25.7; IR (KBr): 3339, 3011, 2945, 1693, 1362, 1254, 1143, 1038, 747 cm^{-1}; HRMS (EI) m/z $[M + H]^+$ calculated for $C_{17}H_{20}NO_3S$: 318.1158, found 318.1159; HPLC: the ee value was determined by HPLC analysis (Chiralpak AD-H, *i*-PrOH/Hexane = 20/80, 1.0 mL/min, 256 nm), retention time: t_{minor} = 10.377 min,

t_{major} = 12.267 min, ee = 89%; $[\alpha]_D^{25}$ = – 20.8 (c = 1.16, THF).

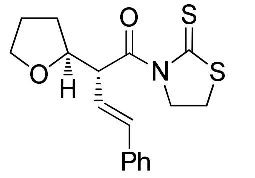

(*R*,*E*)-4-Phenyl-2-((*S*)-tetrahydrofuran-2-yl)-1-(2-thioxothiazolidin-3-yl)but-3-en-1-one (3r)

Prepared according to general procedure D and purified by silica gel chromatography (CH_2Cl_2/EtOAc 99:1). Yield: 63% (42.0 mg), **3r**/**3r'** = 1.5:1. ^{1}H NMR (500 MHz, $CDCl_3$) δ 7.47–7.38 (m, 2H), 7.36–7.28 (m, 2H), 7.27–7.20 (m, 1H), 6.57 (d, *J* = 16.1 Hz, 1H), 6.41 (dd, *J* = 16.1, 9.0 Hz, 1H), 5.63 (dd, *J* = 9.0, 5.3 Hz, 1H), 4.68–4.52 (m, 2H), 4.43 (td, *J* = 7.0, 5.4 Hz, 1H), 3.91–3.83 (m, 1H), 3.83–3.75 (m, 1H), 3.28 (qt, *J* = 11.1, 7.5 Hz, 2H), 2.11–2.01 (m, 1H), 1.94–1.83 (m, 2H), 1.81–1.73 (m, 1H); ^{13}C NMR (126 MHz, $CDCl_3$) δ 202.1, 174.4, 136.9, 134.7, 128.7, 127.9, 126.7, 124.4, 80.0, 69.0, 56.8, 52.8, 29.6, 28.4, 25.9; IR (KBr): 3369, 2972, 2946, 2868, 1696, 1366, 1278, 1149, 1054, 759 cm^{-1}; HRMS (EI) m/z $[M + H]^+$ calculated for $C_{17}H_{20}NO_2S_2$: 334.0930, found 334.0932; HPLC: the ee value was determined by HPLC analysis (Chiralpak AD-H, *i*-PrOH/Hexane = 25/75, 1.0 mL/min, 270 nm), retention time: t_{minor} = 10.300 min, t_{major} = 14.880 min, ee = 95%; $[\alpha]_D^{25}$ = + 58.0 (c = 0.72, THF).

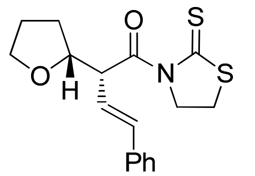

(*R*,*E*)-4-Phenyl-2-((*R*)-tetrahydrofuran-2-yl)-1-(2-thioxothiazolidin-3-yl)but-3-en-1-one (3r')

^{1}H NMR (500 MHz, $CDCl_3$) δ 7.40–7.36 (m, 2H), 7.32 (t, *J* = 7.5 Hz, 2H), 7.27–7.22 (m, 1H), 6.64 (d, *J* = 16.1 Hz, 1H), 6.21 (dd, *J* = 16.0, 9.0 Hz, 1H), 5.53 (t, *J* = 9.1 Hz, 1H), 4.70 (ddd, *J* = 12.2, 7.8, 5.5 Hz, 1H), 4.51 (ddd, *J* = 12.1, 8.9, 7.8 Hz, 1H), 4.44 (dt, *J* = 9.3, 6.3 Hz, 1H), 3.93–3.85 (m, 1H), 3.80 (td, *J* = 7.6, 6.2 Hz, 1H), 3.34 (ddd, *J* = 11.0, 8.9, 7.9 Hz, 1H), 3.21 (ddd, *J* = 11.0, 7.7, 5.5 Hz, 1H), 2.05–1.92 (m, 2H),

1.91–1.85 (m, 1H), 1.80–1.72 (m, 1H); ^{13}C NMR (126 MHz, $CDCl_3$) δ 202.1, 175.0, 136.7, 134.6, 128.7, 128.0, 126.5, 124.2, 81.4, 68.6, 56.7, 53.2, 29.5, 28.6, 25.6; IR (KBr): 3371, 2972, 2945, 2868, 1696, 1363, 1278, 1154, 1059, 751 cm^{-1}; HRMS (EI) m/z [M + H]$^+$ calculated for $C_{17}H_{20}NO_2S_2$: 334.0930, found 334.0932; HPLC: the ee value was determined by HPLC analysis (Chiralpak AD-H, *i*-PrOH/Hexane = 25/75, 1.0 mL/min, 305 nm), retention time: t_{minor} = 9.670 min, t_{major} = 10.957 min, ee = 90%; $[\alpha]_D^{25}$ = – 30.1 (c = 1.05, THF).

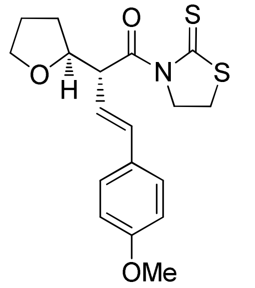

(*R*,*E*)-4-(4-Methoxyphenyl)-2-((*S*)-tetrahydrofuran-2-yl)-1-(2-thioxothiazolidin-3-yl)but-3-en-1-one (3s)

Prepared according to general procedure D and purified by silica gel chromatography (CH_2Cl_2/EtOAc 99:1). Yield: 68% (49.3 mg), **3s**/**3s'** = 2:1. ^{1}H NMR (500 MHz, $CDCl_3$) δ 7.34 (d, *J* = 8.7 Hz, 2H), 6.85 (d, *J* = 8.7 Hz, 2H), 6.52 (d, *J* = 16.0 Hz, 1H), 6.25 (dd, *J* = 16.0, 9.1 Hz, 1H), 5.60 (dd, *J* = 9.1, 5.3 Hz, 1H), 4.68–4.51 (m, 2H), 4.42 (dd, *J* = 12.3, 7.0 Hz, 1H), 3.89–3.75 (m, 5H), 3.36–3.19 (m, 2H), 2.09–1.98 (m, 1H), 1.95–1.82 (m, 2H), 1.82–1.74 (m, 1H); ^{13}C NMR (126 MHz, $CDCl_3$) δ 202.1, 174.6, 159.5, 134.2, 129.7, 127.9, 122.0, 114.1, 80.0, 69.0, 56.9, 55.5, 52.8, 29.6, 28.4, 25.9; IR (KBr): 3356, 2955, 2925, 2871, 1709, 1377, 1277, 1223, 1148, 1050, 779 cm^{-1}; HRMS (EI) m/z [M + H]$^+$ calculated for $C_{18}H_{22}NO_3S_2$: 364.1036, found 364.1035; HPLC: the ee value was determined by HPLC analysis (Chiralpak AD-H, *i*-PrOH/Hexane = 25/75, 1.0 mL/min, 270 nm), retention time: t_{minor} = 17.343 min, t_{major} = 24.587 min, ee = 95%; $[\alpha]_D^{28}$ = + 48.1 (c = 0.44, THF).

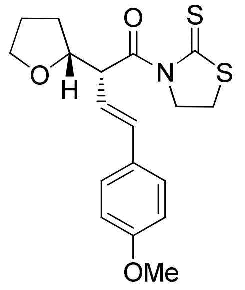

(*R*,*E*)-4-(4-Methoxyphenyl)-2-((*R*)-tetrahydrofuran-2-yl)-1-(2-thioxothiazolidin-3-yl)but-3-en-1-one (3s')

^{1}H NMR (500 MHz, $CDCl_3$) δ 7.31 (d, *J* = 8.7 Hz, 2H), 6.85 (d, *J* = 8.7 Hz, 2H), 6.58 (d, *J* = 16.0 Hz, 1H), 6.04 (dd, *J* = 16.0, 9.0 Hz, 1H), 5.50 (t, *J* = 9.2 Hz, 1H), 4.70 (ddd, *J* = 12.3, 7.8, 5.7 Hz, 1H), 4.55–4.48 (m, 1H), 4.43 (dt, *J* = 9.3, 6.3 Hz, 1H), 3.88 (dd, *J* = 14.6, 6.9 Hz, 1H), 3.83–3.77 (m, 4H), 3.40–3.30 (m, 1H), 3.22 (ddd, *J* = 11.0, 7.7, 5.7 Hz, 1H), 2.02–1.91 (m, 2H), 1.90–1.84 (m, 1H), 1.78–1.72 (m, 1H); ^{13}C NMR (126 MHz, $CDCl_3$) δ 202.1, 175.3, 159.6, 134.1, 129.6, 127.8, 121.9, 114.1, 81.4, 68.7, 56.7, 55.5, 53.2, 29.8, 28.6, 25.6; IR (KBr): 3354, 2954, 2927, 2872, 1708, 1376, 1276, 1224, 1147, 1049, 781 cm^{-1}; HRMS (EI) m/z $[M + H]^+$ calculated for $C_{18}H_{22}NO_3S_2$: 364.1036, found 364.1033; HPLC: the ee value was determined by HPLC analysis (Chiralpak AD-H, *i*-PrOH/Hexane = 25/75, 1.0 mL/min, 265 nm), retention time: t_{minor} = 15.880 min, t_{major} = 16.890 min, ee = 94%; $[\alpha]_D^{28}$ = – 29.5 (c = 0.86, THF).

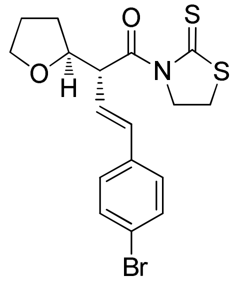

(*R*,*E*)-4-(4-Bromophenyl)-2-((*S*)-tetrahydrofuran-2-yl)-1-(2-thioxothiazolidin-3-yl) but-3-en-1-one (3t)

Prepared according to general procedure D and purified by silica gel chromatography (CH_2Cl_2/EtOAc 99:1). Yield: 73% (60.0 mg), **3t**/**3t'** = 2.2:1. ^{1}H NMR (500 MHz, $CDCl_3$) δ 7.43 (d, *J* = 8.4 Hz, 2H), 7.27 (d, *J* = 8.4 Hz, 2H), 6.51 (d, *J* = 16.1 Hz, 1H), 6.40 (dd, *J* = 16.1, 8.9 Hz, 1H), 5.62 (dd, *J* = 8.8, 5.4 Hz, 1H), 4.68–4.51 (m, 2H),

4.41 (dd, J = 12.5, 7.0 Hz, 1H), 3.85 (dd, J = 15.0, 6.9 Hz, 1H), 3.82–3.74 (m, 1H), 3.36–3.20 (m, 2H), 2.10–2.02 (m, 1H), 1.92–1.82 (m, 2H), 1.78–1.71 (m, 1H); ^{13}C NMR (126 MHz, $CDCl_3$) δ 202.1, 174.2, 135.8, 133.5, 131.8, 128.2, 125.3, 121.7, 80.0, 69.0, 56.8, 52.7, 29.7, 28.4, 25.9; IR (KBr): 3372, 2977, 2875, 1694, 1371, 1281, 1153, 1042, 1012, 780 cm^{-1}; HRMS (EI) m/z [M + H]$^+$ calculated for $C_{17}H_{19}BrNO_2S_2$: 412.0035, found 412.0031; HPLC: the ee value was determined by HPLC analysis (Chiralpak AD-H, *i*-PrOH/Hexane = 25/75, 1.0 mL/min, 268 nm), retention time: t_{minor} = 15.050 min, t_{major} = 24.970 min, ee = 96%; $[\alpha]_D^{28}$ = + 51.1 (c = 0.78, THF).

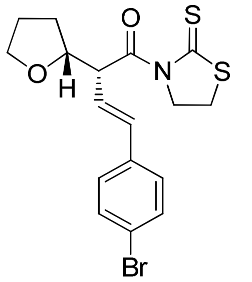

(*R*,*E*)-4-(4-Bromophenyl)-2-((*R*)-tetrahydrofuran-2-yl)-1-(2-thioxothiazolidin-3-yl)but-3-en-1-one (3t')

^{1}H NMR (500 MHz, $CDCl_3$) δ 7.43 (d, J = 8.5 Hz, 2H), 7.23 (d, J = 8.5 Hz, 2H), 6.57 (d, J = 16.1 Hz, 1H), 6.20 (dd, J = 16.0, 9.0 Hz, 1H), 5.50 (t, J = 9.1 Hz, 1H), 4.72 (ddd, J = 12.7, 7.8, 5.1 Hz, 1H), 4.51 (ddd, J = 12.1, 9.4, 7.7 Hz, 1H), 4.42 (dt, J = 9.3, 6.3 Hz, 1H), 3.94–3.84 (m, 1H), 3.83–3.76 (m, 1H), 3.37 (ddd, J = 10.9, 9.4, 7.8 Hz, 1H), 3.22 (ddd, J = 11.0, 7.7, 5.1 Hz, 1H), 2.09–1.85 (m, 3H), 1.76–1.70 (m, 1H); ^{13}C NMR (126 MHz, $CDCl_3$) δ 202.3, 174.9, 135.7, 133.5, 131.8, 128.1, 125.0, 121.8, 81.4, 68.7, 56.7, 53.2, 29.6, 28.7, 25.6; IR (KBr): 3372, 2976, 2877, 1693, 1367, 1281, 1153, 1047, 1013, 786 cm^{-1}; HRMS (EI) m/z [M + H]$^+$ calculated for $C_{17}H_{19}BrNO_2S_2$: 412.0035, found 412.0033; HPLC: the ee value was determined by HPLC analysis (Chiralpak AS-H, *i*-PrOH/Hexane = 25/75, 1.0 mL/min, 268 nm), retention time: t_{minor} = 16.823 min, t_{major} = 25.837 min, ee = 91%; $[\alpha]_D^{28}$ = – 20.1 (c = 0.94, THF).

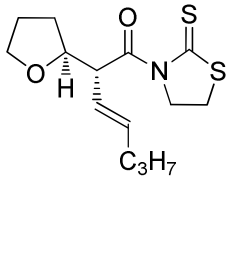

(*R*,*E*)-2-((*S*)-Tetrahydrofuran-2-yl)-1-(2-thioxothiazolidin-3-yl)hept-3-en-1-one (3u)

Prepared according to general procedure D and purified by silica gel chromatography (CH_2Cl_2/petroleum ether 75:25 to 100:0). Yield: 67% (40.0 mg), **3u**/**3u'** = 1.7:1. ^1H NMR (500 MHz, $CDCl_3$) δ 5.74–5.55 (m, 2H), 5.37 (dd, *J* = 7.9, 5.4 Hz, 1H), 4.64–4.48 (m, 2H), 4.30 (dd, *J* = 12.5, 7.0 Hz, 1H), 3.82 (dd, *J* = 14.9, 6.9 Hz, 1H), 3.74 (dd, *J* = 14.1, 7.5 Hz, 1H), 3.27 (qt, *J* = 11.1, 7.5 Hz, 2H), 2.10–1.94 (m, 3H), 1.90–1.79 (m, 2H), 1.77–1.68 (m, 1H), 1.44–1.34 (m, 2H), 0.88 (t, *J* = 7.4 Hz, 3H); ^{13}C NMR (126 MHz, $CDCl_3$) δ 201.9, 175.0, 136.4, 124.4, 79.9, 68.9, 56.9, 52.6, 34.9, 29.5, 28.4, 25.9, 22.4, 13.8; IR (KBr): 3401, 2958, 2877, 1699, 1375, 1270, 1157, 1051 cm^{-1}; HRMS (EI) m/z $[M + H]^+$ calculated for $C_{14}H_{22}NO_2S_2$: 300.1086, found 300.1087; HPLC: the ee value was determined by HPLC analysis (Chiralpak AD-H, *i*-PrOH/Hexane = 10/90, 1.0 mL/min, 310 nm), retention time: t_{minor} = 7.510 min, t_{major} = 8.363 min, ee = 90%;$[\alpha]_D^{25}$ = + 41.5 (c = 1.12, THF).

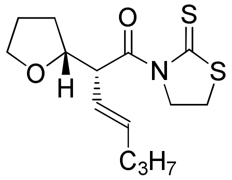

(*R*,*E*)-2-((*R*)-Tetrahydrofuran-2-yl)-1-(2-thioxothiazolidin-3-yl)hept-3-en-1-one (3u')

^1H NMR (500 MHz, $CDCl_3$) δ 5.70 (dt, *J* = 15.2, 6.8 Hz, 1H), 5.40 (dd, *J* = 15.4, 8.8 Hz, 1H), 5.27 (t, *J* = 9.1 Hz, 1H), 4.67 (ddd, *J* = 12.2, 7.8, 5.6 Hz, 1H), 4.57–4.49 (m, 1H), 4.30 (dt, *J* = 9.3, 6.3 Hz, 1H), 3.84 (dd, *J* = 14.6, 6.9 Hz, 1H), 3.76 (dd, *J* = 13.9, 7.6 Hz, 1H), 3.37–3.31 (m, 1H), 3.21 (ddd, *J* = 11.0, 7.7, 5.6 Hz, 1H), 2.03–1.83 (m, 5H), 1.72–1.65 (m, 1H), 1.42–1.35 (m, 2H), 0.87 (t, *J* = 7.4 Hz, 3H); ^{13}C NMR (126 MHz, $CDCl_3$) δ 202.0, 175.7, 136.5, 124.5, 81.4, 68.6, 56.7, 53.1, 34.9, 29.5, 28.7, 25.6, 22.4, 13.8; IR (KBr): 3403, 2958, 2882, 1700, 1377, 1275, 1159, 1051 cm^{-1}; HRMS (EI) m/z $[M + H]^+$ calculated for $C_{14}H_{22}NO_2S_2$: 300.1086, found 300.1082; HPLC: the ee value was determined by HPLC analysis (Chiralpak AD-H, *i*-PrOH/Hexane = 10/90, 1.0 mL/min, 309 nm), retention time: t_{minor} = 6.900 min,

t_{major} = 8.457 min, ee = 86%; $[\alpha]_D^{25}$ = – 48.5 (c = 3.72, THF).

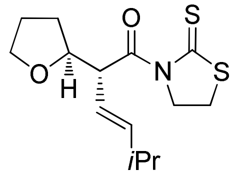

(*R*,*E*)-5-Methyl-2-((*S*)-tetrahydrofuran-2-yl)-1-(2-thioxothiazolidin-3-yl)hex-3-en-1-one (3v)

Prepared according to general procedure D and purified by silica gel chromatography (CH_2Cl_2/petroleum ether 75:25 to 100:0). Yield: 66% (39.5 mg), **3v**/**3v'** = 2.1:1. ^{1}H NMR (500 MHz, $CDCl_3$) δ 5.71–5.49 (m, 2H), 5.34 (dd, *J* = 7.9, 5.0 Hz, 1H), 4.61–4.51 (m, 2H), 4.32 (td, *J* = 7.0, 5.1 Hz, 1H), 3.86–3.80 (m, 1H), 3.78–3.72 (m, 1H), 3.32–3.21 (m, 2H), 2.33 (dq, *J* = 13.4, 6.7 Hz, 1H), 2.03–1.96 (m, 1H), 1.88–1.81 (m, 2H), 1.77–1.68 (m, 1H), 1.00 (dd, *J* = 6.8, 1.2 Hz, 6H); ^{13}C NMR (126 MHz, $CDCl_3$) δ 201.9, 175.1, 143.4, 121.2, 79.9, 69.0, 56.9, 52.5, 31.4, 29.4, 28.5, 25.9, 22.6, 22.4; IR (KBr): 3410, 3352, 2960, 2871, 1701, 1354, 1278, 1160, 1057, 1009, 731 cm^{-1}; HRMS (EI) m/z $[M + H]^+$ calculated for $C_{14}H_{22}NO_2S_2$: 300.1086, found 300.1084; HPLC: the ee value was determined by HPLC analysis (Chiralpak AD-H, *i*-PrOH/Hexane = 25/75, 1.0 mL/min, 307 nm), retention time: t_{minor} = 4.513 min, t_{major} = 4.807 min, ee = 94%; $[\alpha]_D^{25}$ = + 44.2 (c = 1.12, THF).

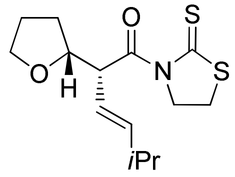

(*R*,*E*)-5-Methyl-2-((*R*)-tetrahydrofuran-2-yl)-1-(2-thioxothiazolidin-3-yl)hex-3-en-1-one (3v')

^{1}H NMR (500 MHz, $CDCl_3$) δ 5.68 (dd, *J* = 15.5, 6.6 Hz, 1H), 5.36 (ddd, *J* = 15.5, 8.8, 1.2 Hz, 1H), 5.24 (t, *J* = 9.1 Hz, 1H), 4.73–4.63 (m, 1H), 4.55–4.47 (m, 1H), 4.29 (dt, *J* = 9.3, 6.2 Hz, 1H), 3.87–3.81 (m, 1H), 3.79–3.73 (m, 1H), 3.33 (ddd, *J* = 11.2, 8.9, 7.8 Hz, 1H), 3.21 (ddd, *J* = 11.0, 7.7, 5.5 Hz, 1H), 2.27 (dqd, *J* = 13.4, 6.7, 1.0 Hz, 1H), 1.96–1.81 (m, 3H), 1.71–1.63 (m, 1H), 0.96 (d, *J* = 6.8 Hz, 6H); ^{13}C NMR (126

MHz, $CDCl_3$) δ 202.0, 175.8, 143.5, 121.5, 81.5, 68.6, 56.7, 53.0, 31.4, 29.4, 28.7, 25.6, 22.4, 22.3; IR (KBr): 3412, 3353, 2960, 2872, 1700, 1356, 1276, 1164, 1059, 1007, 729 cm^{-1}; HRMS (EI) m/z $[M + H]^+$ calculated for $C_{14}H_{22}NO_2S_2$: 300.1086, found 300.1085; HPLC: the ee value was determined by HPLC analysis (Chiralpak AD-H, *i*-PrOH/Hexane = 25/75, 1.0 mL/min, 310 nm), retention time: t_{minor} = 4.573 min, t_{major} = 5.450 min, ee = 89%; $[\alpha]_D^{25}$ = – 56.9 (c = 0.94, THF).

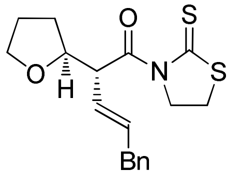

(*R*,*E*)-5-Phenyl-2-((*S*)-tetrahydrofuran-2-yl)-1-(2-thioxothiazolidin-3-yl)pent-3-en-1-one (3w)

Prepared according to general procedure D and purified by silica gel chromatography (CH_2Cl_2/petroleum ether 75:25 to 100:0). Yield: 62% (43.0 mg), **5h**/**5h'** = 2.2:1. ^1H NMR (500 MHz, $CDCl_3$) δ 7.31–7.27 (m, 2H), 7.26–7.09 (m, 3H), 5.87–5.69 (m, 2H), 5.46–5.37 (m, 1H), 4.59–4.47 (m, 2H), 4.34 (dd, *J* = 12.9, 6.6 Hz, 1H), 3.84 (q, *J* = 7.3 Hz, 1H), 3.76 (dd, *J* = 14.4, 7.2 Hz, 1H), 3.47–3.38 (m, 2H), 3.24 (dt, *J* = 10.8, 7.5 Hz, 1H), 3.15 (dt, *J* = 11.0, 7.5 Hz, 1H), 2.09–1.96 (m, 1H), 1.93–1.80 (m, 2H), 1.79–1.68 (m, 1H); ^{13}C NMR (126 MHz, $CDCl_3$) δ 201.9, 174.8, 140.3, 134.7, 128.7, 128.6, 126.3, 126.0, 79.9, 68.9, 56.9, 52.5, 39.3, 29.6, 28.4, 25.9; IR (KBr): 3408, 3021, 2988, 2891, 1700, 1360, 1271, 1161, 1059, 715 cm^{-1}; HRMS (EI) m/z $[M + H]^+$ calculated for $C_{18}H_{22}NO_2S_2$: 448.1086, found 448.1089; HPLC: the ee value was determined by HPLC analysis (Chiralpak AD-H, *i*-PrOH/Hexane = 10/90, 1.0 mL/min, 307 nm), retention time: t_{minor} = 10.903 min, t_{major} = 12.510 min, ee = 91%; $[\alpha]_D^{25}$ = + 48.4 (c = 0.48, THF).

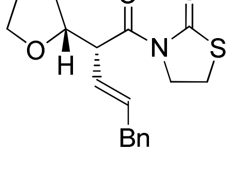

(*R*,*E*)-5-Phenyl-2-((*R*)-tetrahydrofuran-2-yl)-1-(2-thioxothiazolidin-3-yl)pent-3-e

n-1-one (3w')

^{1}H NMR (500 MHz, $CDCl_3$) δ 7.29 (t, *J* = 7.5 Hz, 2H), 7.20 (t, *J* = 7.3 Hz, 1H), 7.15 (d, *J* = 7.6 Hz, 2H), 5.94–5.79 (m, 1H), 5.55 (dd, *J* = 15.4, 8.7 Hz, 1H), 5.32 (t, *J* = 9.0 Hz, 1H), 4.72–4.60 (m, 1H), 4.51–4.45 (m, 1H), 4.38–4.27 (m, 1H), 3.83 (q, *J* = 7.2 Hz, 1H), 3.76 (dd, *J* = 14.2, 7.3 Hz, 1H), 3.37 (d, *J* = 6.9 Hz, 2H), 3.31 (dd, *J* = 19.0, 8.7 Hz, 1H), 3.20–3.12 (m, 1H), 1.99–1.83 (m, 3H), 1.73–1.65 (m, 1H); ^{13}C NMR (126 MHz, $CDCl_3$) δ 202.0, 175.3, 140.0, 134.7, 128.6, 128.6, 126.3, 125.9, 81.3, 68.5, 56.7, 53.0, 39.2, 29.5, 28.6, 25.6; IR (KBr): 3407, 3022, 2987, 2890, 1699, 1360, 1271, 1163, 1061, 718 cm^{-1}; HRMS (EI) m/z [M + H]$^+$ calculated for $C_{18}H_{22}NO_2S_2$: 448.1086, found 448.1086; HPLC: the ee value was determined by HPLC analysis (Chiralpak AD-H, *i*-PrOH/Hexane = 25/75, 1.0 mL/min, 273 nm), retention time: t_{minor} = 6.703 min, t_{major} = 8.317 min, ee = 87%; $[\alpha]_D^{25}$ = – 53.8 (c = 1.52, THF).

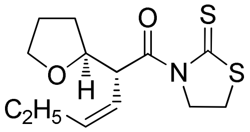

(*R*,*Z*)-2-((*S*)-Tetrahydrofuran-2-yl)-1-(2-thioxothiazolidin-3-yl)hex-3-en-1-one (3x)

Prepared according to general procedure D and purified by silica gel chromatography (CH_2Cl_2/petroleum ether 75:25 to 100:0). Yield: 72% (41.0 mg), **3x/3x'** = 2.1:1. ^{1}H NMR (500 MHz, $CDCl_3$) δ 5.82 (dd, *J* = 9.7, 5.2 Hz, 1H), 5.70 (dt, *J* = 10.6, 7.3 Hz, 1H), 5.54 (t, *J* = 10.2 Hz, 1H), 4.55 (qt, *J* = 12.1, 7.5 Hz, 2H), 4.35–4.29 (m, 1H), 3.85–3.80 (m, 1H), 3.76 (td, *J* = 7.8, 5.9 Hz, 1H), 3.34–3.22 (m, 2H), 2.09 (p, *J* = 7.4 Hz, 2H), 2.01–1.94 (m, 1H), 1.93–1.81 (m, 2H), 1.79–1.70 (m, 1H), 0.98 (t, *J* = 7.5 Hz, 3H); ^{13}C NMR (126 MHz, $CDCl_3$) δ 202.2, 175.2, 137.3, 123.1, 80.0, 68.9, 57.1, 47.8, 29.3, 28.5, 26.0, 22.2, 14.4; IR (KBr): 3418, 2962, 2946, 2868, 1700, 1363, 1271, 1144, 1060, 891 cm^{-1}; HRMS (EI) m/z [M + H]$^+$ calculated for $C_{13}H_{20}NO_2S_2$: 286.0930, found 286.0933; HPLC: the ee value was determined by HPLC analysis (Chiralcel OJ-H, *i*-PrOH/Hexane = 10/90, 1.0 mL/min, 309 nm), retention time: t_{minor}

= 8.383 min, t_{major} = 9.150 min, ee = 94%; $[\alpha]_D^{25}$ = + 31.2 (c = 0.28, THF).

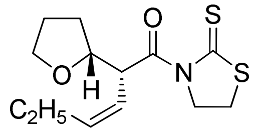

(*R*,*Z*)-2-((*R*)-Tetrahydrofuran-2-yl)-1-(2-thioxothiazolidin-3-yl)hex-3-en-1-one (3x')

^{1}H NMR (500 MHz, $CDCl_3$) δ 5.67 (t, *J* = 9.6 Hz, 1H), 5.51 (dt, *J* = 10.6, 7.4 Hz, 1H), 5.27 (t, *J* = 10.3 Hz, 1H), 4.59–4.52 (m, 1H), 4.44 (dt, *J* = 12.1, 7.8 Hz, 1H), 4.20 (dt, *J* = 9.3, 5.8 Hz, 1H), 3.82 (dt, *J* = 13.3, 6.6 Hz, 1H), 3.71–3.67 (m, 1H), 3.27–3.21 (m, 1H), 3.19–3.14 (m, 1H), 2.16–2.01 (m, 2H), 1.90–1.83 (m, 2H), 1.81–1.77 (m, 1H), 1.62–1.56 (m, 1H), 0.91 (t, *J* = 7.5 Hz, 3H); ^{13}C NMR (126 MHz, $CDCl_3$) δ 202.3, 176.1, 137.3, 123.6, 82.0, 68.8, 56.9, 49.2, 29.6, 28.7, 25.5, 22.6, 14.3; IR (KBr): 3417, 2960, 2948, 2867, 1700, 1363, 1271, 1146, 1059, 893 cm^{-1}; HRMS (EI) m/z [M + H]$^+$ calculated for $C_{13}H_{20}NO_2S_2$: 286.0930, found 286.0929; HPLC: the ee value was determined by HPLC analysis (Chiralcel OJ-H, *i*-PrOH/Hexane = 10/90, 1.0 mL/min, 309 nm), retention time: t_{major} = 10.947 min, t_{minor} = 12.313 min, ee = 90%; $[\alpha]_D^{25}$ = – 72.8 (c = 0.72, THF).

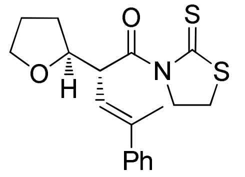

(*R*,*E*)-4-Phenyl-2-((*S*)-tetrahydrofuran-2-yl)-1-(2-thioxothiazolidin-3-yl)pent-3-en-1-one (3y)

Prepared according to general procedure D and purified by silica gel chromatography (CH_2Cl_2/EtOAc 99:1). Yield: 64% (44.4 mg), **3y/3y'** = 2.5:1. ^{1}H NMR (500 MHz, $CDCl_3$) δ 7.49–7.42 (m, 2H), 7.36–7.30 (m, 2H), 7.27–7.22 (m, 1H), 6.02 (d, *J* = 2.1 Hz, 2H), 4.66–4.52 (m, 2H), 4.46–4.38 (m, 1H), 3.86 (dd, *J* = 15.0, 6.9 Hz, 1H), 3.82–3.76 (m, 1H), 3.33 (dt, *J* = 11.0, 8.0 Hz, 1H), 3.25 (ddd, *J* = 11.1, 7.6, 6.3 Hz, 1H), 2.11 (s, 3H), 2.03 (dtd, *J* = 12.0, 7.0, 4.8 Hz, 1H), 1.93–1.83 (m, 2H), 1.82–1.75 (m, 1H); ^{13}C NMR (126 MHz, $CDCl_3$) δ 202.3, 174.9, 143.1, 139.9, 128.4, 127.4,

126.2, 122.2, 80.3, 69.1, 57.1, 48.9, 29.3, 28.5, 26.1, 17.5; IR (KBr): 3381, 2941, 2923, 1699, 1367, 1271, 1139, 1042, 732 cm^{-1}; HRMS (EI) m/z $[M + H]^+$ calculated for $C_{18}H_{22}NO_2S_2$: 348.1086, found 348.1084; HPLC: the ee value was determined by HPLC analysis (Chiralpak AD-H, *i*-PrOH/Hexane = 25/75, 1.0 mL/min, 271 nm), retention time: t_{minor} = 7.140 min, t_{major} = 26.967 min, ee = 96%; $[\alpha]_D^{28}$ = +3.5 (c = 0.60, THF).

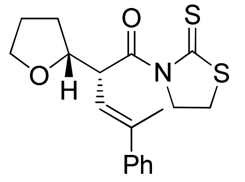

(*R*,*E*)-4-Phenyl-2-((*R*)-tetrahydrofuran-2-yl)-1-(2-thioxothiazolidin-3-yl)pent-3-en-1-one (3y')

^{1}H NMR (500 MHz, $CDCl_3$) δ 7.41 (d, *J* = 7.4 Hz, 2H), 7.33 (t, *J* = 7.6 Hz, 2H), 7.28–7.24 (m, 1H), 5.93 (t, *J* = 9.5 Hz, 1H), 5.80 (d, *J* = 10.0 Hz, 1H), 4.73–4.62 (m, 1H), 4.55 (dt, *J* = 12.1, 7.8 Hz, 1H), 4.38 (dt, *J* = 9.1, 5.7 Hz, 1H), 3.98–3.90 (m, 1H), 3.80 (dd, *J* = 14.3, 7.3 Hz, 1H), 3.33 (dt, *J* = 10.9, 7.9 Hz, 1H), 3.25 (dt, *J* = 11.0, 7.1 Hz, 1H), 2.18 (s, 3H), 2.03–1.93 (m, 2H), 1.93–1.86 (m, 1H), 1.79–1.70 (m, 1H); ^{13}C NMR (126 MHz, $CDCl_3$) δ 202.4, 175.9, 143.0, 140.0, 128.4, 127.6, 126.1, 123.0, 82.5, 68.9, 57.0, 50.3, 29.6, 28.7, 25.6, 18.1; IR (KBr): 3380, 2942, 2924, 1699, 1368, 1270, 1139, 1043, 728 cm^{-1}; HRMS (EI) m/z $[M + H]^+$ calculated for $C_{18}H_{22}NO_2S_2$: 348.1086, found 348.1084; HPLC: the ee value was determined by HPLC analysis (Chiralpak AD-H, *i*-PrOH/Hexane = 25/75, 1.0 mL/min, 310 nm), retention time: t_{minor} = 6.623 min, t_{major} = 13.103 min, ee = 92%; $[\alpha]_D^{28}$ = – 73.8 (c = 0.64, THF).

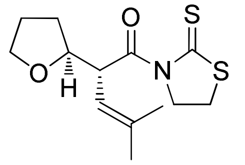

(*R*)-4-Methyl-2-((*S*)-tetrahydrofuran-2-yl)-1-(2-thioxothiazolidin-3-yl)pent-3-en-1-one (3z)

Prepared according to general procedure D and purified by silica gel chromatography (CH_2Cl_2/petroleum ether 75:25 to 100:0). Yield: 75% (42.7 mg), **3z/3z'** = 2.3:1. ^{1}H

NMR (500 MHz, $CDCl_3$) δ 5.68 (dd, *J* = 9.6, 5.1 Hz, 1H), 5.27 (d, *J* = 9.6 Hz, 1H), 4.53–4.42 (m, 2H), 4.23 (dd, *J* = 12.2, 7.0 Hz, 1H), 3.75 (dd, *J* = 14.9, 7.0 Hz, 1H), 3.71–3.66 (m, 1H), 3.25–3.14 (m, 2H), 1.94–1.86 (m, 1H), 1.83–1.75 (m, 2H), 1.71 (s, 3H), 1.69–1.64 (m, 1H), 1.61 (s, 3H); ^{13}C NMR (126 MHz, $CDCl_3$) δ 202.2, 175.5, 137.9, 119.1, 80.3, 69.0, 57.1, 48.5, 29.2, 28.5, 26.1, 26.0, 19.5; IR (KBr): 3431, 3256, 2937, 2871, 1699, 1357, 1271, 1144, 1048, 648 cm^{-1}; HRMS (EI) m/z $[M + H]^+$ calculated for $C_{13}H_{20}NO_2S_2$: 286.0930, found 286.0928; HPLC: the ee value was determined by HPLC analysis (Chiralcel OJ-H, *i*-PrOH/Hexane = 10/90, 1.0 mL/min, 309 nm), retention time: t_{major} = 13.187 min, t_{minor} = 15.117 min, ee = 93%; $[\alpha]_D^{25}$ = + 27.3 (c = 0.42, THF).

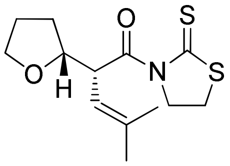

(*R*)-4-Methyl-2-((*R*)-tetrahydrofuran-2-yl)-1-(2-thioxothiazolidin-3-yl)pent-3-en-1-one (3z')

^1H NMR (500 MHz, $CDCl_3$) δ 5.60 (t, *J* = 9.6 Hz, 1H), 5.08 (d, *J* = 9.8 Hz, 1H), 4.60–4.52 (m, 1H), 4.48–4.42 (m, 1H), 4.21–4.14 (m, 1H), 3.87–3.80 (m, 1H), 3.68 (dd, *J* = 14.5, 7.0 Hz, 1H), 3.25–3.14 (m, 2H), 1.89–1.77 (m, 3H), 1.66 (d, *J* = 0.8 Hz, 6H), 1.61–1.58 (m, 1H); ^{13}C NMR (126 MHz, $CDCl_3$) δ 202.3, 176.5, 137.9, 119.9, 82.4, 68.8, 57.0, 49.9, 29.5, 28.6, 26.1, 25.5, 20.0; IR (KBr): 3433, 3257, 2936, 2870, 1699, 1355, 1273, 1146, 1046, 651 cm^{-1}; HRMS (EI) m/z $[M + H]^+$ calculated for $C_{13}H_{20}NO_2S_2$: 286.0930, found 286.0932; HPLC: the ee value was determined by HPLC analysis (Chiralcel OJ-H, *i*-PrOH/Hexane = 10/90, 1.0 mL/min, 313 nm), retention time: t_{major} = 10.793 min, t_{minor} = 13.947 min, ee = 91%; $[\alpha]_D^{25}$ = – 67.5 (c = 0.35, THF).

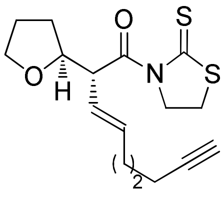

(*R*,*E*)-2-((*S*)-Tetrahydrofuran-2-yl)-1-(2-thioxothiazolidin-3-yl)non-3-en-8-yn-1-o

ne (3ba)

Prepared according to general procedure D and purified by silica gel chromatography (CH_2Cl_2/petroleum ether 75:25 to 100:0). Yield: 63% (40.7 mg), **3ba/3ba'** = 2:1. ^1H NMR (500 MHz, $CDCl_3$) δ 5.71–5.58 (m, 2H), 5.44–5.37 (m, 1H), 4.63–4.50 (m, 2H), 4.31 (dd, J = 12.6, 7.0 Hz, 1H), 3.83 (dd, J = 15.0, 6.8 Hz, 1H), 3.76 (dd, J = 14.0, 7.6 Hz, 1H), 3.35–3.22 (m, 2H), 2.29–2.11 (m, 4H), 2.05–1.99 (m, 1H), 1.95 (t, J = 2.6 Hz, 1H), 1.91–1.82 (m, 2H), 1.75–1.69 (m, 1H), 1.65–1.60 (m, 2H); ^{13}C NMR (126 MHz, $CDCl_3$) δ 201.9, 174.9, 135.1, 125.5, 84.4, 79.9, 68.9, 68.7, 56.9, 52.6, 31.7, 29.6, 28.5, 28.0, 25.9, 17.9; IR (KBr): 3413, 2936, 2876, 1715, 1699, 1358, 1271, 1147, 1044, 723 cm^{-1}; HRMS (EI) m/z $[M + H]^+$ calculated for $C_{16}H_{22}NO_2S_2$: 324.1086, found 324.1086; HPLC: the ee value was determined by HPLC analysis (Chiralpak AD-H, *i*-PrOH/Hexane = 25/75, 1.0 mL/min, 310 nm), retention time: t_{minor} = 7.197 min, t_{major} = 7.657 min, ee = 90%; $[\alpha]_D^{28}$ = + 31.2 (c = 0.24, THF).

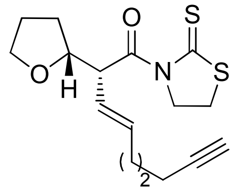

(*R*,*E*)-2-((*R*)-Tetrahydrofuran-2-yl)-1-(2-thioxothiazolidin-3-yl)non-3-en-8-yn-1-one (3ba')

^1H NMR (500 MHz, $CDCl_3$) δ 5.70 (dt, J = 15.2, 6.8 Hz, 1H), 5.47 (dd, J = 15.5, 8.7 Hz, 1H), 5.28 (t, J = 9.0 Hz, 1H), 4.73–4.63 (m, 1H), 4.50 (ddd, J = 12.1, 9.0, 7.7 Hz, 1H), 4.30 (dt, J = 9.4, 6.4 Hz, 1H), 3.87–3.82 (m, 1H), 3.81–3.74 (m, 1H), 3.34 (ddd, J = 10.9, 9.1, 7.8 Hz, 1H), 3.22 (ddd, J = 11.0, 7.7, 5.4 Hz, 1H), 2.21–2.13 (m, 4H), 1.98–1.85 (m, 4H), 1.69–1.59 (m, 3H); ^{13}C NMR (126 MHz, $CDCl_3$) δ 202.0, 175.6, 135.2, 125.5, 84.3, 81.4, 68.8, 68.6, 56.8, 53.2, 31.7, 29.6, 28.7, 27.9, 25.6, 17.9; IR (KBr): 3415, 2935, 2877, 1715, 1700, 1356, 1269, 1146, 1043, 720 cm^{-1}; HRMS (EI) m/z $[M + H]^+$ calculated for $C_{16}H_{22}NO_2S_2$: 324.1086, found 324.1088; HPLC: the ee value was determined by HPLC analysis (Chiralpak AD-H, *i*-PrOH/Hexane = 25/75, 1.0 mL/min, 309 nm), retention time: t_{minor} = 6.900 min, t_{major} = 8.723 min, ee = 87%; $[\alpha]_D^{28}$ = – 35.3 (c = 0.20, THF).

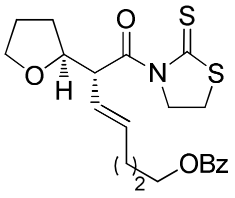

(*R*,*E*)-7-Oxo-6-((*S*)-tetrahydrofuran-2-yl)-7-(2-thioxothiazolidin-3-yl)hept-4-en-1-yl benzoate (3bb)

Prepared according to general procedure D and purified by silica gel chromatography (CH_2Cl_2/petroleum ether 75:25 to 100:0). Yield: 65% (54.5 mg), **3bb**/**3bb'** = 2:1. ^{1}H NMR (500 MHz, $CDCl_3$) δ 8.06–8.01 (m, 2H), 7.59–7.54 (m, 1H), 7.45 (t, *J* = 7.7 Hz, 2H), 5.76–5.63 (m, 2H), 5.42 (dd, *J* = 7.7, 5.6 Hz, 1H), 4.56 (t, *J* = 7.5 Hz, 2H), 4.36–4.28 (m, 3H), 3.84–3.78 (m, 1H), 3.74 (dd, *J* = 14.0, 7.7 Hz, 1H), 3.28 (t, *J* = 7.5 Hz, 2H), 2.33–2.22 (m, 2H), 2.04–1.97 (m, 1H), 1.91–1.83 (m, 4H), 1.74–1.67 (m, 1H); ^{13}C NMR (126 MHz, $CDCl_3$) δ 202.0, 174.8, 166.8, 134.9, 133.1, 130.5, 129.7, 128.6, 125.6, 79.9, 68.9, 64.4, 56.9, 52.5, 29.6, 29.3, 28.4, 28.3, 25.9; IR (KBr): 3413, 2924, 2851, 1700, 1360, 1275, 1150, 1101, 1048 cm^{-1}; HRMS (EI) m/z [M + H]$^+$ calculated for $C_{21}H_{26}NO_4S_2$: 420.1298, found 420.1299; HPLC: the ee value was determined by HPLC analysis (Chiralpak IB-H, *i*-PrOH/Hexane = 25/75, 1.0 mL/min, 31 nm), retention time: t_{minor} = 8.590 min, t_{major} = 9.410 min, ee = 92%; $[\alpha]_D^{27}$ = + 21.4 (c = 0.35, THF).

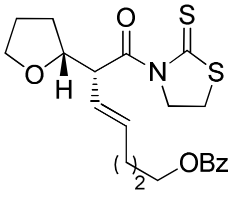

(*R*,*E*)-7-Oxo-6-((*R*)-tetrahydrofuran-2-yl)-7-(2-thioxothiazolidin-3-yl)hept-4-en-1-yl benzoate (3bb')

^{1}H NMR (500 MHz, $CDCl_3$) δ 8.04 (d, *J* = 7.2 Hz, 2H), 7.57 (t, *J* = 7.4 Hz, 1H), 7.45 (t, *J* = 7.7 Hz, 2H), 5.77 (dt, *J* = 15.1, 6.8 Hz, 1H), 5.49 (dd, *J* = 15.5, 8.8 Hz, 1H), 5.30 (t, *J* = 9.1 Hz, 1H), 4.79–4.62 (m, 1H), 4.55–4.48 (m, 1H), 4.36–4.27 (m, 3H), 3.84 (dd, *J* = 14.3, 7.2 Hz, 1H), 3.76 (dd, *J* = 14.0, 7.6 Hz, 1H), 3.38–3.31 (m, 1H), 3.23 (ddd, *J* = 11.0, 7.7, 5.4 Hz, 1H), 2.29–2.17 (m, 2H), 1.98–1.83 (m, 5H),

1.70–1.64 (m, 1H); ^{13}C NMR (126 MHz, $CDCl_3$) δ 202.1, 175.5, 166.8, 135.0, 133.1, 130.5, 129.7, 128.6, 125.7, 81.3, 68.6, 64.4, 56.8, 53.1, 29.6, 29.4, 28.7, 28.3, 25.6; IR (KBr): 3414, 2923, 2849, 1701, 1359, 1274, 1149, 1103, 1044 cm^{-1}; HRMS (EI) m/z [M + H]$^+$ calculated for $C_{21}H_{26}NO_4S_2$: 420.1298, found 420.1296; HPLC: the ee value was determined by HPLC analysis (Chiralpak AD-H, *i*-PrOH/Hexane = 25/75, 1.0 mL/min, 310 nm), retention time: t_{minor} = 8.990 min, t_{major} = 1.653 min, ee = 88%; $[\alpha]_D^{27}$ = – 24.5 (c = 0.28, THF).

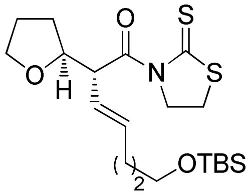

(*R*,*E*)-7-((*tert*-Butyldimethylsilyl)oxy)-2-((*S*)-tetrahydrofuran-2-yl)-1-(2-thioxothiazolidin-3-yl)hept-3-en-1-one (3bc)

Prepared according to general procedure D and purified by silica gel chromatography (CH_2Cl_2/petroleum ether 75:25). Yield: 61% (52.3 mg), **3bc/3bc'** = 2:1. ^{1}H NMR (500 MHz, $CDCl_3$) δ 5.72–5.56 (m, 2H), 5.39 (dd, *J* = 7.9, 5.5 Hz, 1H), 4.64–4.48 (m, 2H), 4.30 (dd, *J* = 12.6, 7.0 Hz, 1H), 3.86–3.80 (m, 1H), 3.75 (dd, *J* = 14.0, 7.6 Hz, 1H), 3.60 (t, *J* = 6.5 Hz, 2H), 3.27 (qt, *J* = 11.0, 7.5 Hz, 2H), 2.13 (dd, *J* = 14.3, 6.3 Hz, 2H), 2.04–1.97 (m, 1H), 1.90–1.82 (m, 2H), 1.76–1.68 (m, 1H), 1.64–1.57 (m, 2H), 0.89 (d, *J* = 2.8 Hz, 9H), 0.05 (s, 6H); ^{13}C NMR (126 MHz, $CDCl_3$) δ 201.9, 175.0, 136.0, 124.6, 80.0, 68.9, 62.7, 56.9, 52.5, 32.5, 29.5, 29.2, 28.4, 26.2, 25.9, 18.6, -5.1; IR (KBr): 3415, 2928, 2856, 1701, 1368, 1285, 1161, 1052, 699 cm^{-1}; HRMS (EI) m/z [M + H]$^+$ calculated for $C_{20}H_{36}NO_3S_2Si$: 430.1900, found 430.1901; HPLC: the ee value was determined by HPLC analysis (Chiralpak AD-H, *i*-PrOH/Hexane = 5/95, 1.0 mL/min, 310 nm), retention time: t_{minor} = 5.393 min, t_{major} = 5.873 min, ee = 92%; $[\alpha]_D^{25}$ = + 27.5 (c = 0.15, THF).

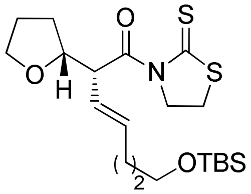

(*R*,*E*)-7-((*tert*-Butyldimethylsilyl)oxy)-2-((*S*)-tetrahydrofuran-2-yl)-1-(2-thioxothiazolidin-3-yl)hept-3-en-1-one (3bc')

^{1}H NMR (500 MHz, $CDCl_3$) δ 5.72 (dt, *J* = 15.2, 6.8 Hz, 1H), 5.42 (dd, *J* = 15.5, 8.8 Hz, 1H), 5.27 (t, *J* = 9.1 Hz, 1H), 4.72–4.63 (m, 1H), 4.49 (ddd, *J* = 12.1, 9.0, 7.8 Hz, 1H), 4.30 (dt, *J* = 9.3, 6.3 Hz, 1H), 3.84 (dd, *J* = 14.6, 6.9 Hz, 1H), 3.76 (dd, *J* = 14.0, 7.5 Hz, 1H), 3.58 (t, *J* = 6.5 Hz, 2H), 3.34 (ddd, *J* = 10.9, 9.0, 8.0 Hz, 1H), 3.21 (ddd, *J* = 11.0, 7.7, 5.4 Hz, 1H), 2.08 (dd, *J* = 14.3, 7.3 Hz, 2H), 1.97–1.84 (m, 3H), 1.71–1.63 (m, 1H), 1.62–1.55 (m, 2H), 0.89 (s, 9H), 0.04 (s, 6H); ^{13}C NMR (126 MHz, $CDCl_3$) δ 202.0, 175.6, 136.1, 124.7, 81.4, 68.6, 62.6, 56.7, 53.1, 32.3, 29.5, 29.2, 28.7, 26.2, 25.6, 18.5, -5.1; IR (KBr): 3416, 2931, 2858, 1701, 1369, 1289, 1162, 1053, 697 cm^{-1}; HRMS (EI) m/z [M + H]$^+$ calculated for $C_{20}H_{36}NO_3S_2Si$: 430.1900, found 430.1901; HPLC: the ee value was determined by HPLC analysis (Chiralpak AD-H, *i*-PrOH/Hexane = 5/95, 1.0 mL/min, 310 nm), retention time: t_{minor} = 5.520 min, t_{major} = 6.840 min, ee = 87%; $[\alpha]_D^{25}$ = – 51.5 (c = 0.10, THF).

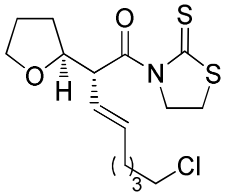

(*R*,*E*)-8-Chloro-2-((*S*)-tetrahydrofuran-2-yl)-1-(2-thioxothiazolidin-3-yl)oct-3-en-1-one (3bd)

Prepared according to general procedure D and purified by silica gel chromatography (CH_2Cl_2/petroleum ether 75:25 to 100:0). Yield: 66% (45.8 mg), **3bd**/**3bd'** = 2:1. ^{1}H NMR (500 MHz, $CDCl_3$) δ 5.73–5.58 (m, 2H), 5.46–5.35 (m, 1H), 4.62–4.51 (m, 2H), 4.30 (dd, *J* = 12.7, 7.0 Hz, 1H), 3.86–3.79 (m, 1H), 3.75 (dd, *J* = 14.1, 7.6 Hz, 1H), 3.53 (t, *J* = 6.7 Hz, 2H), 3.35–3.20 (m, 2H), 2.18–2.08 (m, 2H), 2.04–1.97 (m, 1H), 1.91–1.83 (m, 2H), 1.81–1.74 (m, 2H), 1.73–1.67 (m, 1H), 1.58–1.50 (m, 2H); ^{13}C NMR (126 MHz, $CDCl_3$) δ 201.9, 174.9, 135.5, 125.2, 80.0, 68.9, 56.9, 52.5, 45.1, 32.2, 32.0, 29.6, 28.4, 26.5, 25.9; IR (KBr): 3415, 2933, 2846, 2098, 1701, 1368, 1279, 1228, 1150, 1060 cm^{-1}; HRMS (EI) m/z [M + H]$^+$ calculated for $C_{15}H_{23}ClNO_2S_2$: 348.0853, found 348.0851; HPLC: the ee value was determined by

HPLC analysis (Chiralpak AS-H, *i*-PrOH/Hexane = 25/75, 1.0 mL/min, 310 nm), retention time: t_{minor} = 10.343 min, t_{major} = 11.120 min, ee = 91%; $[\alpha]_D^{28}$ = + 15.1 (c = 0.090, THF).

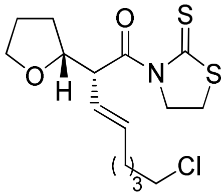

(*R*,*E*)-8-Chloro-2-((*R*)-tetrahydrofuran-2-yl)-1-(2-thioxothiazolidin-3-yl)oct-3-en-1-one (3bd')

^{1}H NMR (500 MHz, $CDCl_3$) δ 5.70 (dt, *J* = 15.2, 6.8 Hz, 1H), 5.45 (dd, *J* = 15.5, 8.7 Hz, 1H), 5.27 (t, *J* = 9.0 Hz, 1H), 4.68 (ddd, *J* = 13.0, 7.8, 5.4 Hz, 1H), 4.50 (ddd, *J* = 12.1, 9.0, 7.8 Hz, 1H), 4.30 (dt, *J* = 9.3, 6.3 Hz, 1H), 3.89–3.81 (m, 1H), 3.80–3.71 (m, 1H), 3.53 (t, *J* = 6.6 Hz, 2H), 3.34 (ddd, *J* = 10.9, 9.0, 7.9 Hz, 1H), 3.22 (ddd, *J* = 11.0, 7.7, 5.4 Hz, 1H), 2.14–2.01 (m, 2H), 1.99–1.82 (m, 3H), 1.80–1.71 (m, 2H), 1.71–1.63 (m, 1H), 1.58–1.48 (m, 2H); ^{13}C NMR (126 MHz, $CDCl_3$) δ 202.0, 175.6, 135.71, 125.2, 81.4, 68.6, 56.7, 53.1, 45.1, 32.1, 32.1, 29.6, 28.7, 26.4, 25.6; IR (KBr): 3416, 2931, 2849, 2097, 1701, 1368, 1278, 1226, 1149, 1061 cm^{-1}; HRMS (EI) m/z $[M + H]^+$ calculated for $C_{15}H_{23}ClNO_2S_2$: 348.0853, found 348.0853; HPLC: the ee value was determined by HPLC analysis (Chiralpak AS-H, *i*-PrOH/Hexane = 25/75, 1.0 mL/min, 309 nm), retention time: t_{minor} = 11.923 min, t_{major} = 16.460 min, ee = 86%; $[\alpha]_D^{28}$ = – 38.8 (c = 0.25, THF).

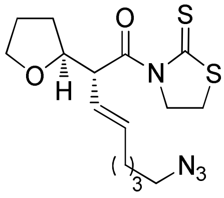

(*R*,*E*)-8-Azido-2-((*S*)-tetrahydrofuran-2-yl)-1-(2-thioxothiazolidin-3-yl)oct-3-en-1-one (3be)

Prepared according to general procedure D and purified by silica gel chromatography (CH_2Cl_2/petroleum ether 75:25 to 100:0). Yield: 64% (45.3 mg), **3be**/**3be'** = 2:1. ^{1}H NMR (500 MHz, $CDCl_3$) δ 5.75–5.58 (m, 2H), 5.46–5.33 (m, 1H), 4.63–4.49 (m, 2H),

4.30 (dd, J = 12.7, 6.9 Hz, 1H), 3.83 (dd, J = 15.0, 6.9 Hz, 1H), 3.76 (dd, J = 14.3, 7.4 Hz, 1H), 3.38–3.19 (m, 4H), 2.20–2.07 (m, 2H), 2.01 (dt, J = 12.2, 7.1 Hz, 1H), 1.91–1.83 (m, 2H), 1.74–1.67 (m, 1H), 1.62–1.58 (m, 2H), 1.52–1.44 (m, 2H); ^{13}C NMR (126 MHz, $CDCl_3$) δ 201.9, 174.9, 135.5, 125.2, 80.0, 68.9, 56.9, 52.5, 51.5, 32.3, 29.6, 28.4, 26.3, 25.9; IR (KBr): 3415, 3028, 2951, 2902, 1690, 1411, 1365, 1017, 820 cm^{-1}; HRMS (EI) m/z $[M + H]^+$ calculated for $C_{15}H_{23}N_4O_2S_2$: 355.1257, found 355.1258; HPLC: the ee value was determined by HPLC analysis (Chiralpak AD-H, *i*-PrOH/Hexane = 10/90, 1.0 mL/min, 313 nm), retention time: t_{minor} = 12.050 min, t_{major} = 13.383 min, ee = 90%; $[\alpha]_D^{28}$ = + 6.8 (c = 0.20, THF).

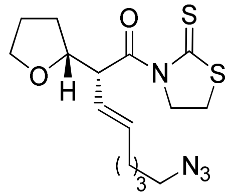

(*R*,*E*)-8-Azido-2-((*S*)-tetrahydrofuran-2-yl)-1-(2-thioxothiazolidin-3-yl)oct-3-en-1-one (3be')

^{1}H NMR (500 MHz, $CDCl_3$) δ 5.70 (dt, J = 15.3, 6.8 Hz, 1H), 5.44 (dd, J = 15.5, 8.7 Hz, 1H), 5.27 (t, J = 9.0 Hz, 1H), 4.78–4.63 (m, 1H), 4.50 (ddd, J = 12.1, 9.2, 7.7 Hz, 1H), 4.30 (dt, J = 9.3, 6.3 Hz, 1H), 3.89–3.81 (m, 1H), 3.77 (dd, J = 13.9, 7.6 Hz, 1H), 3.35 (ddd, J = 10.9, 9.1, 7.9 Hz, 1H), 3.31–3.08 (m, 3H), 2.17–2.00 (m, 2H), 1.99–1.82 (m, 3H), 1.69–1.65 (m, 1H), 1.61–1.55 (m, 2H), 1.50–1.42 (m, 2H); ^{13}C NMR (126 MHz, $CDCl_3$) δ 202.1, 175.6, 135.7, 125.2, 81.4, 68.6, 56.7, 53.1, 51.5, 32.3, 29.6, 28.7, 28.4, 26.3, 25.6; IR (KBr): 3416, 3027, 2952, 2905, 1694, 1412, 1366, 1016, 825 cm^{-1}; HRMS (EI) m/z $[M + H]^+$ calculated for $C_{15}H_{23}N_4O_2S_2$: 355.1257, found 355.1255; HPLC: the ee value was determined by HPLC analysis (Chiralpak AD-H, *i*-PrOH/Hexane = 25/75, 1.0 mL/min, 307 nm), retention time: t_{minor} = 7.227 min, t_{major} = 8.637 min, ee = 86%; $[\alpha]_D^{28}$ = – 52.8 (c = 0.18, THF).

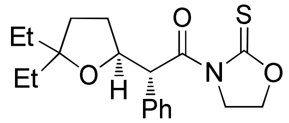

(*R*)-2-((*S*)-5,5-Diethyltetrahydrofuran-2-yl)-2-phenyl-1-(2-thioxooxazolidin-3-yl)ethanone (4a)

Prepared according to general procedure E using THF/CH_2Cl_2 (3:1) as solvent and purified by silica gel chromatography (CH_2Cl_2/petroleum ether 75:25 to 100:0). Yield: 76% (52.7 mg), **4a**/**4a'** = 3.5:1. ^{1}H NMR (500 MHz, $CDCl_3$) δ 7.48 (d, *J* = 7.1 Hz, 2H), 7.31 (t, *J* = 7.3 Hz, 2H), 7.28–7.24 (m, 1H), 6.35 (d, *J* = 8.3 Hz, 1H), 4.58 (dt, *J* = 8.2, 6.6 Hz, 1H), 4.49 (td, *J* = 9.2, 6.8 Hz, 1H), 4.39 (dd, *J* = 17.5, 9.0 Hz, 1H), 4.26–4.19 (m, 1H), 4.12 (ddd, *J* = 11.3, 9.3, 6.7 Hz, 1H), 2.10 (ddt, *J* = 11.9, 7.8, 6.0 Hz, 1H), 1.89–1.69 (m, 3H), 1.56–1.36 (m, 4H), 0.83 (t, *J* = 7.5 Hz, 3H), 0.78 (t, *J* = 7.5 Hz, 3H); ^{13}C NMR (126 MHz, $CDCl_3$) δ 185.5, 174.0, 136.4, 129.9, 128.5, 127.6, 86.9, 81.0, 66.1, 53.3, 47.6, 33.95, 31.5, 30.7, 30.7, 8.9, 8.8; IR (KBr): 3381, 2978, 2932, 2874, 1691, 1379, 1156, 1018, 725 cm^{-1}; HRMS (EI) m/z $[M + H]^+$ calculated for $C_{19}H_{26}NO_3S$: 348.1628, found 348.1630; HPLC: the ee value was determined by HPLC analysis (Chiralpak AD-H, *i*-PrOH/Hexane = 10/90, 1.0 mL/min, 262 nm), retention time: t_{minor} = 7.140 min, t_{major} = 8.250 min, ee = 96%; $[\alpha]_D^{28}$ = + 16.5 (c = 0.54, THF).

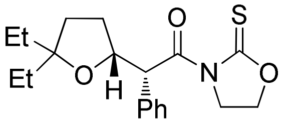

(*R*)-2-((*R*)-5,5-Diethyltetrahydrofuran-2-yl)-2-phenyl-1-(2-thioxooxazolidin-3-yl) ethanone (4a')

^{1}H NMR (500 MHz, $CDCl_3$) δ 7.55–7.47 (m, 2H), 7.34–7.29 (m, 2H), 7.29–7.26 (m, 1H), 6.21 (d, *J* = 9.6 Hz, 1H), 4.73–4.61 (m, 1H), 4.50–4.40 (m, 2H), 4.34 (ddd, *J* = 11.1, 9.6, 6.8 Hz, 1H), 4.15 (ddd, *J* = 11.2, 9.2, 8.3 Hz, 1H), 1.72–1.45 (m, 8H), 0.86 (dt, *J* = 10.7, 7.5 Hz, 6H); ^{13}C NMR (126 MHz, $CDCl_3$) δ 185.6, 174.4, 135.4, 129.7, 128.7, 127.9, 87.2, 82.0, 66.3, 54.0, 47.7, 33.7, 31.7, 30.9, 30.0, 8.9, 8.9; IR (KBr): 3382, 2977, 2931, 2873, 1695, 1378, 1157, 1017, 726 cm^{-1}; HRMS (EI) m/z $[M + H]^+$ calculated for $C_{19}H_{26}NO_3S$: 348.1628, found 348.1631; HPLC: the ee value was determined by HPLC analysis (Chiralpak AD-H, *i*-PrOH/Hexane = 10/90, 1.0 mL/min, 263 nm), retention time: t_{minor} = 6.630 min, t_{major} = 7.637 min, ee = 95%; $[\alpha]_D^{28}$ = + 10.1 (c = 0.70, THF).

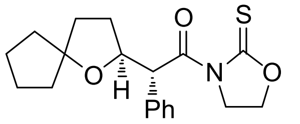

(*R*)-2-Phenyl-2-((*S*)-1-oxaspiro[4.4]nonan-2-yl)-1-(2-thioxooxazolidin-3-yl)ethanone (4b)

Prepared according to general procedure E using THF/CH_2Cl_2 (3:1) as solvent and purified by silica gel chromatography (CH_2Cl_2/petroleum ether 75:25 to 100:0). Yield: 77% (53.0 mg), **4b**/**4b'** = 3.8:1. ^{1}H NMR (500 MHz, $CDCl_3$) δ 7.57–7.40 (m, 2H), 7.35–7.29 (m, 2H), 7.29–7.27 (m, 1H), 6.30 (d, *J* = 8.2 Hz, 1H), 4.66–4.54 (m, 1H), 4.50 (td, *J* = 9.3, 6.7 Hz, 1H), 4.40 (dd, *J* = 17.5, 9.1 Hz, 1H), 4.25 (ddd, *J* = 11.3, 9.5, 8.4 Hz, 1H), 4.14 (ddd, *J* = 11.3, 9.3, 6.7 Hz, 1H), 2.21–2.11 (m, 1H), 1.93–1.74 (m, 4H), 1.74–1.63 (m, 3H), 1.60–1.44 (m, 4H); ^{13}C NMR (126 MHz, $CDCl_3$) δ 185.5, 173.9, 136.2, 130.1, 128.5, 127.7, 92.2, 80.6, 66.2, 53.7, 47.7, 39.1, 38.4, 36.5, 31.0, 24.2, 23.9; IR (KBr): 3376, 2973, 2931, 2863, 1690, 1378, 1208, 1155, 1019, 912, 735 cm^{-1}; HRMS (EI) m/z [M + H]$^+$ calculated for $C_{19}H_{24}NO_3S$: 346.1471, found 346.1480; HPLC: the ee value was determined by HPLC analysis (Chiralpak IB-H, *i*-PrOH/Hexane = 10/90, 1.0 mL/min, 273 nm), retention time: t_{minor} = 11.397 min, t_{major} = 13.467 min, ee = 94%; $[\alpha]_D^{28}$ = + 25.4 (c = 0.24, THF).

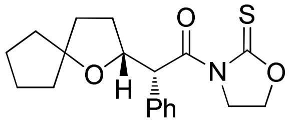

(*R*)-2-Phenyl-2-((*R*)-1-oxaspiro[4.4]nonan-2-yl)-1-(2-thioxooxazolidin-3-yl)ethanone (4b')

^{1}H NMR (500 MHz, $CDCl_3$) δ 7.62–7.44 (m, 2H), 7.36–7.30 (m, 2H), 7.30–7.27 (m, 1H), 6.29 (d, *J* = 9.7 Hz, 1H), 4.69 (dt, *J* = 9.7, 6.2 Hz, 1H), 4.52–4.41 (m, 2H), 4.35 (ddd, *J* = 11.2, 9.5, 7.0 Hz, 1H), 4.16 (ddd, *J* = 11.2, 9.2, 8.0 Hz, 1H), 1.91–1.48 (m, 12H); ^{13}C NMR (126 MHz, $CDCl_3$) δ 185.7, 174.6, 135.4, 129.8, 128.8, 127.9, 92.5, 81.6, 66.3, 53.8, 47.7, 39.4, 38.7, 36.3, 29.9, 24.3, 24.1; IR (KBr): 3377, 2973, 2932, 2864, 1695, 1377, 1210, 1157, 1018, 913, 737cm^{-1}; HRMS (EI) m/z [M + H]$^+$ calculated for $C_{19}H_{24}NO_3S$: 346.1471, found 346.1475; HPLC: the ee value was determined by HPLC analysis (Chiralpak IB-H, *i*-PrOH/Hexane = 10/90, 1.0 mL/min,

274 nm), retention time: t_{major} = 8.823 min, t_{minor} = 12.187 min, ee = 95%; $[\alpha]_D^{28}$ = + 2.4 (c = 0.30, THF).

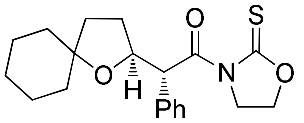

(*R*)-2-Phenyl-2-((*S*)-1-oxaspiro[4.5]decan-2-yl)-1-(2-thioxooxazolidin-3-yl)ethanone (4c)

Prepared according to general procedure E using THF/CH_2Cl_2 (3:1) as solvent and purified by silica gel chromatography (CH_2Cl_2/petroleum ether 75:25 to 100:0). Yield: 72% (51.7 mg), **4c**/**4c'** = 4:1. ^{1}H NMR (500 MHz, $CDCl_3$) δ 7.48 (d, *J* = 7.2 Hz, 2H), 7.32 (t, *J* = 7.3 Hz, 2H), 7.30–7.25 (m, 1H), 6.32 (d, *J* = 8.2 Hz, 1H), 4.60 (dd, *J* = 14.5, 6.4 Hz, 1H), 4.48 (td, *J* = 9.2, 6.9 Hz, 1H), 4.37 (dd, *J* = 17.5, 9.0 Hz, 1H), 4.26–4.18 (m, 1H), 4.13 (ddd, *J* = 11.3, 9.4, 6.9 Hz, 1H), 2.20–2.09 (m, 1H), 1.90–1.71 (m, 3H), 1.65–1.29 (m, 10H); ^{13}C NMR (126 MHz, $CDCl_3$) δ 185.5, 173.9, 136.2, 130.0, 128.4, 127.5, 83.8, 80.4, 66.1, 53.5, 47.6, 38.6, 37.7, 35.6, 30.2, 25.8, 24.1, 24.0; IR (KBr): 3378, 2972, 2933, 2860, 1690, 1379, 1209, 1156, 1018, 913, 738 cm^{-1}; HRMS (EI) m/z $[M + H]^+$ calculated for $C_{20}H_{26}NO_3S$: 360.1628, found 360.1630; HPLC: the ee value was determined by HPLC analysis (Chiralpak IB-H, *i*-PrOH/Hexane = 10/90, 1.0 mL/min, 274 nm), retention time: t_{minor} = 10.310 min, t_{major} = 11.313 min, ee = 95%; $[\alpha]_D^{28}$ = + 28.3 (c = 1.24, THF).

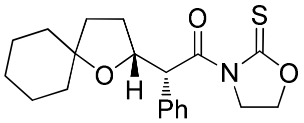

(*R*)-2-Phenyl-2-((*R*)-1-oxaspiro[4.5]decan-2-yl)-1-(2-thioxooxazolidin-3-yl)ethanone (4c')

^{1}H NMR (500 MHz, $CDCl_3$) δ 7.51 (d, *J* = 7.2 Hz, 2H), 7.31 (t, *J* = 7.3 Hz, 2H), 7.29–7.26 (m, 1H), 6.23 (d, *J* = 9.7 Hz, 1H), 4.75–4.65 (m, 1H), 4.52–4.40 (m, 2H), 4.39–4.30 (m, 1H), 4.16 (dt, *J* = 11.1, 8.8 Hz, 1H), 1.79–1.32 (m, 14H); ^{13}C NMR (126 MHz, $CDCl_3$) δ 185.7, 174.6, 135.4, 129.7, 128.7, 127.8, 84.1, 81.5, 66.3, 54.1, 47.7, 38.8, 37.6, 35.2, 29.2, 25.8, 24.3, 24.1; IR (KBr): 3378, 2973, 2932, 2859, 1694, 1378, 1210, 1156, 1017, 913, 736cm^{-1}; HRMS (EI) m/z $[M + H]^+$ calculated for

$C_{20}H_{26}NO_3S$: 360.1628, found 360.1627; HPLC: the ee value was determined by HPLC analysis (Chiralpak IB-H, *i*-PrOH/Hexane = 10/90, 1.0 mL/min, 272 nm), retention time: t_{major} = 8.233 min, t_{minor} = 12.833 min, ee = 96%; $[\alpha]_D^{28}$ = + 1.2 (c = 1.24, THF).

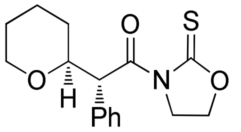

(*R*)-2-Phenyl-2-((*S*)-tetrahydro-2*H*-pyran-2-yl)-1-(2-thioxooxazolidin-3-yl)ethanone (4d)

Prepared according to general procedure E and purified by silica gel chromatography (CH_2Cl_2/EtOAc 99:1). Yield: 60% (36.6 mg), **4d**/**4d'** = 1.9:1. ^1H NMR (500 MHz, $CDCl_3$) δ 7.50 (d, *J* = 7.8 Hz, 2H), 7.32 (t, *J* = 7.4 Hz, 2H), 7.30–7.26 (m, 1H), 6.39 (d, *J* = 8.3 Hz, 1H), 4.47 (dd, *J* = 17.0, 8.2 Hz, 1H), 4.38 (q, *J* = 8.8 Hz, 1H), 4.22 (dd, *J* = 19.0, 9.6 Hz, 1H), 4.11 (dd, *J* = 18.2, 9.3 Hz, 1H), 4.00 (t, *J* = 9.0 Hz, 1H), 3.90 (d, *J* = 11.1 Hz, 1H), 3.32 (t, *J* = 10.9 Hz, 1H), 1.89–1.79 (m, 1H), 1.75–1.66 (m, 1H), 1.61–1.44 (m, 4H); ^{13}C NMR (126 MHz, $CDCl_3$) δ 185.4, 173.5, 135.9, 130.0, 128.4, 127.7, 79.6, 69.0, 66.1, 53.3, 47.6, 29.8, 25.9, 23.4; IR (KBr): 3361, 2968, 2949, 2871, 1692, 1378, 1209, 1151, 1071, 1019, 710 cm^{-1}; HRMS (EI) m/z $[M + H]^+$ calculated for $C_{16}H_{20}NO_3S$: 306.1158, found 306.1160; HPLC: the ee value was determined by HPLC analysis (Chiralpak AD-H, *i*-PrOH/Hexane = 10/90, 1.0 mL/min, 277 nm), retention time: t_{major} = 13.793 min, t_{minor} = 16.960 min, ee = 96%; $[\alpha]_D^{20}$ = – 30.0 (c = 1.12, THF).

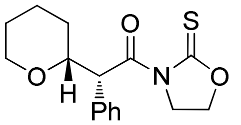

(*R*)-2-Phenyl-2-((*R*)-tetrahydro-2*H*-pyran-2-yl)-1-(2-thioxooxazolidin-3-yl)ethanone (4d')

^1H NMR (500 MHz, $CDCl_3$) δ 7.49 (d, *J* = 7.6 Hz, 2H), 7.31 (t, *J* = 7.4 Hz, 2H), 7.29–7.25 (m, 1H), 6.19 (d, *J* = 9.9 Hz, 1H), 4.53–4.37 (m, 2H), 4.36–4.26 (m, 1H), 4.15 (dd, *J* = 19.0, 9.7 Hz, 1H), 4.03 (td, *J* = 9.8, 3.2 Hz, 1H), 4.00–3.91 (m, 1H),

3.53–3.37 (m, 1H), 1.79–1.72 (m, 1H), 1.60–1.52 (m, 1H), 1.50–1.45 (m, 1H), 1.41–1.32 (m, 1H), 1.25–1.13 (m, 2H); ^{13}C NMR (126 MHz, $CDCl_3$) δ 185.4, 174.2, 134.5, 130.0, 128.7, 127.9, 80.6, 68.8, 66.2, 53.8, 47.6, 29.1, 26.0, 23.3; IR (KBr): 3363, 2967, 2948, 2872, 1694, 1377, 1208, 1152, 1065, 1017, 712 cm^{-1}; HRMS (EI) m/z $[M + H]^+$ calculated for $C_{16}H_{20}NO_3S$: 306.1158, found 306.1161; HPLC: the ee value was determined by HPLC analysis (Chiralpak AD-H, *i*-PrOH/Hexane = 20/80, 1.0 mL/min, 264 nm), retention time: t_{minor} = 6.540 min, t_{major} = 7.067 min, ee = 96%; $[\alpha]_D^{20}$ = – 7.0 (c = 1.16, THF).

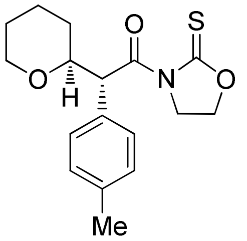

(*R*)-2-((*S*)-Tetrahydro-2*H*-pyran-2-yl)-1-(2-thioxooxazolidin-3-yl)-2-(p-tolyl)ethanone (4e)

Prepared according to general procedure E and purified by silica gel chromatography (CH_2Cl_2/EtOAc 99:1). Yield: 56% (35.7 mg), **4e**/**4e’** = 2:1. ^{1}H NMR (500 MHz, $CDCl_3$) δ 7.39 (d, *J* = 8.1 Hz, 2H), 7.14 (d, *J* = 8.0 Hz, 2H), 6.34 (d, *J* = 8.4 Hz, 1H), 4.49 (td, *J* = 9.2, 6.8 Hz, 1H), 4.39 (dd, *J* = 17.4, 9.1 Hz, 1H), 4.23 (ddd, *J* = 11.3, 9.5, 8.2 Hz, 1H), 4.12 (ddd, *J* = 11.3, 9.4, 6.8 Hz, 1H), 4.01–3.96 (m, 1H), 3.94–3.88 (m, 1H), 3.33 (td, *J* = 11.5, 2.3 Hz, 1H), 2.33 (s, 3H), 1.87–1.81 (m, 1H), 1.73–1.68 (m, 1H), 1.59–1.43 (m, 4H); ^{13}C NMR (126 MHz, $CDCl_3$) δ 185.4, 173.7, 137.4, 132.9, 129.9, 129.3, 79.6, 69.1, 66.1, 53.0, 47.7, 29.9, 26.0, 23.5, 21.4; IR (KBr): 3357, 2977, 2927, 2854, 1692, 1375, 1212, 1069, 1020 cm^{-1}; HRMS (EI) m/z $[M + H]^+$ calculated for $C_{17}H_{22}NO_3S$: 320.1315, found 320.1311; HPLC: the ee value was determined by HPLC analysis (Chiralpak IB-H, *i*-PrOH/Hexane = 10/90, 1.0 mL/min, 264 nm), retention time: t_{minor} = 14.050 min, t_{major} = 15.080 min, ee = 94%; $[\alpha]_D^{28}$ = – 28.4 (c = 0.26, THF).

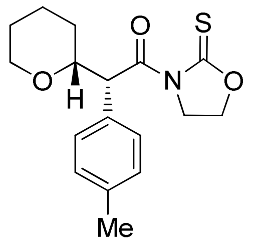

(*R*)-2-((*R*)-Tetrahydro-2*H*-pyran-2-yl)-1-(2-thioxooxazolidin-3-yl)-2-(p-tolyl)ethanone (4e')

^{1}H NMR (500 MHz, $CDCl_3$) δ 7.37 (d, *J* = 8.1 Hz, 2H), 7.13 (d, *J* = 8.0 Hz, 2H), 6.14 (d, *J* = 9.9 Hz, 1H), 4.52–4.38 (m, 2H), 4.31 (ddd, *J* = 11.1, 9.6, 7.0 Hz, 1H), 4.14 (ddd, *J* = 11.1, 9.4, 8.0 Hz, 1H), 4.01 (ddd, *J* = 9.9, 7.8, 5.3 Hz, 1H), 3.98–3.93 (m, 1H), 3.46 (td, *J* = 11.7, 2.4 Hz, 1H), 2.32 (s, 3H), 1.78–1.72 (m, 1H), 1.60–1.53 (m, 1H), 1.50–1.45 (m, 1H), 1.40–1.31 (m, 1H), 1.24–1.14 (m, 2H); ^{13}C NMR (126 MHz, $CDCl_3$) δ 185.4, 174.4, 137.7, 131.5, 129.8, 129.4, 80.6, 68.8, 66.2, 53.5, 47.7, 29.1, 26.0, 23.4, 21.3; IR (KBr): 3358, 2978, 2927, 2855, 1695, 1376, 1213, 1071, 1019 cm^{-1}; HRMS (EI) m/z $[M + H]^+$ calculated for $C_{17}H_{22}NO_3S$: 320.1315, found 320.1314; HPLC: the ee value was determined by HPLC analysis (Chiralpak IB-H, *i*-PrOH/Hexane = 20/80, 1.0 mL/min, 262 nm), retention time: t_{major} = 7.283 min, t_{minor} = 12.220 min, ee = 95%; $[\alpha]_D^{28}$ = – 20.5 (c = 0.48, THF).

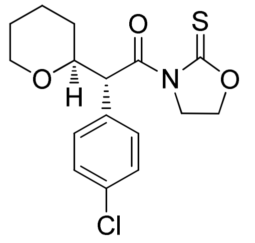

(*R*)-2-(4-Chlorophenyl)-2-((*S*)-tetrahydro-2*H*-pyran-2-yl)-1-(2-thioxooxazolidin-3-yl)ethanone (4f)

Prepared according to general procedure E and purified by silica gel chromatography (CH_2Cl_2/EtOAc 99:1). Yield: 65% (44.0 mg), **4f/4f'** = 2.3:1. ^{1}H NMR (500 MHz, $CDCl_3$) δ 7.44 (d, *J* = 8.5 Hz, 2H), 7.29 (d, *J* = 8.5 Hz, 2H), 6.35 (d, *J* = 8.1 Hz, 1H), 4.51 (td, *J* = 9.2, 7.3 Hz, 1H), 4.44 (dt, *J* = 16.9, 8.5 Hz, 1H), 4.24 (ddd, *J* = 11.3, 9.5, 7.8 Hz, 1H), 4.14 (ddd, *J* = 11.4, 9.4, 7.3 Hz, 1H), 3.96 (ddd, *J* = 10.1, 8.2, 1.9 Hz, 1H), 3.93–3.87 (m, 1H), 3.32 (td, *J* = 11.3, 2.5 Hz, 1H), 1.87–1.79 (m, 1H), 1.70–1.67

(m, 1H), 1.57–1.39 (m, 4H); ^{13}C NMR (126 MHz, $CDCl_3$) δ 185.4, 173.2, 134.4, 133.7, 131.5, 128.6, 79.5, 69.1, 66.2, 52.8, 47.7, 29.9, 25.9, 23.4; IR (KBr): 3389, 2979, 2948, 2921, 1692, 1401, 1365, 1017, 781 cm^{-1}; HRMS (EI) m/z $[M + H]^+$ calculated for $C_{16}H_{19}ClNO_3S$: 340.0769, found 340.0766; HPLC: the ee value was determined by HPLC analysis (Chiralpak IB-H, *i*-PrOH/Hexane = 10/90, 1.0 mL/min, 262 nm), retention time: t_{major} = 12.160 min, t_{minor} = 13.667 min, ee = 97%; $[\alpha]_D^{28}$ = – 49.2 (c = 0.34, THF).

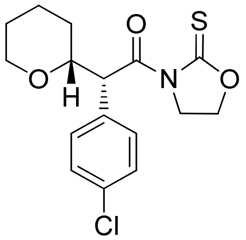

(*R*)-2-(4-Chlorophenyl)-2-((*R*)-tetrahydro-2*H*-pyran-2-yl)-1-(2-thioxooxazolidin-3-yl)ethanone (4f')

^{1}H NMR (500 MHz, $CDCl_3$) δ 7.36 (d, *J* = 8.5 Hz, 2H), 7.22 (d, *J* = 8.5 Hz, 2H), 6.08 (d, *J* = 9.9 Hz, 1H), 4.46–4.34 (m, 2H), 4.24 (ddd, *J* = 11.2, 9.4, 6.7 Hz, 1H), 4.12–4.06 (m, 1H), 3.97–3.83 (m, 2H), 3.37 (td, *J* = 11.7, 2.5 Hz, 1H), 1.72–1.65 (m, 1H), 1.52–1.46 (m, 1H), 1.42–1.38 (m, 1H), 1.32–1.28 (m, 1H), 1.15–1.09 (m, 2H); ^{13}C NMR (126 MHz, $CDCl_3$) δ 185.5, 174.1, 134.0, 133.0, 131.3, 128.9, 80.7, 68.8, 66.3, 53.2, 47.7, 29.1, 25.9, 23.3; IR (KBr): 3391, 2978, 2950, 2923, 1694, 1402, 1366, 1017, 784 cm^{-1}; HRMS (EI) m/z $[M + H]^+$ calculated for $C_{16}H_{19}ClNO_3S$: 340.0769, found 340.0767; HPLC: the ee value was determined by HPLC analysis (Chiralpak IB-H, *i*-PrOH/Hexane = 20/80, 1.0 mL/min, 274 nm), retention time: t_{major} = 7.417 min, t_{minor} = 12.580 min, ee = 97%; $[\alpha]_D^{28}$ = – 13.6 (c = 0.42, THF).

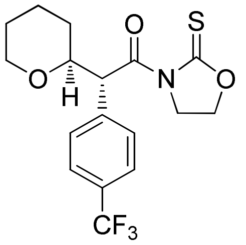

(*R*)-2-((*S*)-Tetrahydro-2*H*-pyran-2-yl)-1-(2-thioxooxazolidin-3-yl)-2-(4-(trifluoro methyl)phenyl)e-thanone (4g)

Prepared according to general procedure E and purified by silica gel chromatography (CH_2Cl_2/EtOAc 99:1). Yield: 63% (47.0 mg), **4g/4g'** = 2.5:1. ^{1}H NMR (500 MHz, $CDCl_3$) δ 7.63 (d, *J* = 8.2 Hz, 2H), 7.58 (d, *J* = 8.3 Hz, 2H), 6.45 (d, *J* = 8.1 Hz, 1H), 4.55–4.49 (m, 1H), 4.49–4.41 (m, 1H), 4.25 (ddd, *J* = 11.2, 9.4, 7.8 Hz, 1H), 4.15 (ddd, *J* = 11.3, 9.4, 7.5 Hz, 1H), 4.01 (ddd, *J* = 10.1, 8.2, 1.8 Hz, 1H), 3.94–3.86 (m, 1H), 3.32 (td, *J* = 11.3, 2.5 Hz, 1H), 1.89–1.80 (m, 1H), 1.73–1.67 (m, 1H), 1.58–1.38 (m, 4H); ^{13}C NMR (126 MHz, $CDCl_3$) δ 185.4, 172.8, 140.0, 130.5, 129.8 (q, *J* = 32.3 Hz), 125.3 (q, *J* = 3.7 Hz), 124.4 (q, *J* = 272.1 Hz), 79.5, 69.1, 66.2, 53.2, 47.7, 29.9, 25.9, 23.4; IR (KBr): 3398, 3001, 2977, 2916, 1686, 1390, 1379, 1246, 1019 cm^{-1}; HRMS (EI) m/z $[M + H]^+$ calculated for $C_{17}H_{19}F_3NO_3S$: 374.1032, found 374.1033; HPLC: the ee value was determined by HPLC analysis (Chiralpak IB-H, *i*-PrOH/Hexane = 10/90, 1.0 mL/min, 272 nm), retention time: t_{minor} = 11.553 min, t_{major} = 12.530 min, ee = 94%; $[\alpha]_D^{27}$ = – 40.9 (c = 0.80, THF).

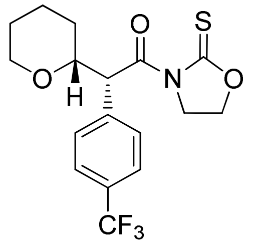

(*R*)-2-((*R*)-Tetrahydro-2*H*-pyran-2-yl)-1-(2-thioxooxazolidin-3-yl)-2-(4-(trifluoro methyl)phenyl)e-thanone (4g')

^{1}H NMR (500 MHz, $CDCl_3$) δ 7.64 (d, *J* = 8.2 Hz, 2H), 7.58 (d, *J* = 8.3 Hz, 2H), 6.26 (d, *J* = 9.9 Hz, 1H), 4.55–4.43 (m, 2H), 4.33 (ddd, *J* = 11.1, 9.4, 6.6 Hz, 1H), 4.17 (dt, *J* = 11.2, 9.1 Hz, 1H), 4.03 (td, *J* = 10.2, 2.5 Hz, 1H), 3.99–3.92 (m, 1H), 3.46 (td, *J* = 11.8, 2.4 Hz, 1H), 1.80–1.73 (m, 1H), 1.61–1.52 (m, 1H), 1.52–1.45 (m, 1H), 1.42–1.33 (m, 1H), 1.24–1.14 (m, 2H); ^{13}C NMR (126 MHz, $CDCl_3$) δ 185.5, 173.8, 138.6, 130.4, 130.2 (q, *J* = 32.4 Hz), 125.6 (q, *J* = 3.7 Hz), 124.2 (q, *J* = 272.1 Hz), 80.7, 68.9, 66.4, 53.7, 47.7, 29.2, 25.9, 23.2; IR (KBr): 3399, 3001, 2978, 2917, 1688, 1389, 1380, 1247, 1019 cm^{-1}; HRMS (EI) m/z $[M + H]^+$ calculated for $C_{17}H_{19}F_3NO_3S$: 374.1032, found 374.1033; HPLC: the ee value was determined by HPLC analysis (Chiralpak IB-H, *i*-PrOH/Hexane = 10/90, 1.0 mL/min, 264 nm), retention time: t_{major}

= 9.480 min, t_{minor} = 15.620 min, ee = 94%; $[\alpha]_D^{27}$ = – 12.8 (c = 0.92, THF).

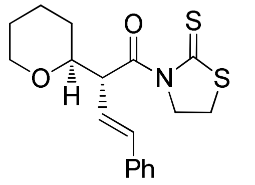

(*R*,*E*)-4-Phenyl-2-((*S*)-tetrahydro-2*H*-pyran-2-yl)-1-(2-thioxothiazolidin-3-yl)but-3-en-1-one (4h)

Prepared according to general procedure E and purified by silica gel chromatography (CH_2Cl_2/petroleum ether 75:25 to 100:0). Yield: 51% (35.4 mg), **4h**/**4h'** = 1.5:1. ^{1}H NMR (500 MHz, $CDCl_3$) δ 7.42 (d, *J* = 7.2 Hz, 2H), 7.31 (t, *J* = 7.6 Hz, 2H), 7.23 (t, *J* = 7.3 Hz, 1H), 6.52 (d, *J* = 16.1 Hz, 1H), 6.43 (dd, *J* = 16.0, 8.9 Hz, 1H), 5.54 (dd, *J* = 8.9, 5.1 Hz, 1H), 4.71–4.46 (m, 2H), 4.06–3.94 (m, 1H), 3.94–3.82 (m, 1H), 3.42 (td, *J* = 11.3, 4.5 Hz, 1H), 3.31 (dt, *J* = 11.0, 8.1 Hz, 1H), 3.25 (ddd, *J* = 11.0, 7.5, 6.3 Hz, 1H), 1.88–1.78 (m, 1H), 1.62–1.43 (m, 5H); ^{13}C NMR (126 MHz, $CDCl_3$) δ 202.0, 174.3, 137.1, 134.4, 128.7, 127.8, 126.8, 124.9, 78.9, 69.2, 57.0, 53.7, 29.4, 28.5, 25.9, 23.5; IR (KBr): 3365, 2970, 2941, 2862, 1694, 1351, 1275, 1150, 1056, 761 cm^{-1}; HRMS (EI) m/z $[M + H]^+$ calculated for $C_{18}H_{22}NO_2S_2$: 348.1086, found 348.1088; HPLC: the ee value was determined by HPLC analysis (Chiralpak IB-H, *i*-PrOH/Hexane = 5/95, 1.0 mL/min, 310 nm), retention time: t_{minor} = 13.627 min, t_{major} = 14.623 min, ee = 92%; $[\alpha]_D^{27}$ = – 21.8 (c = 0.76, THF).

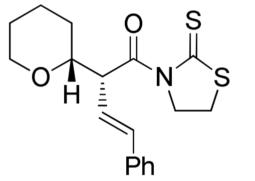

(*R*,*E*)-4-Phenyl-2-((*R*)-tetrahydro-2*H*-pyran-2-yl)-1-(2-thioxothiazolidin-3-yl)but-3-en-1-one (4h')

^{1}H NMR (500 MHz, $CDCl_3$) δ 7.40–7.35 (m, 2H), 7.33–7.29 (m, 2H), 7.26–7.21 (m, 1H), 6.65 (d, *J* = 16.0 Hz, 1H), 6.15 (dd, *J* = 16.0, 9.2 Hz, 1H), 5.39 (t, *J* = 9.3 Hz, 1H), 4.66 (ddd, *J* = 12.0, 7.8, 4.3 Hz, 1H), 4.48 (ddd, *J* = 11.9, 10.1, 7.6 Hz, 1H), 3.98–3.90 (m, 1H), 3.84–3.77 (m, 1H), 3.43 (td, *J* = 11.7, 2.4 Hz, 1H), 3.40–3.34 (m,

1H), 3.19 (ddd, J = 11.0, 7.6, 4.3 Hz, 1H), 1.85–1.73 (m, 3H), 1.60–1.54 (m, 1H), 1.51–1.43 (m, 2H); ^{13}C NMR (126 MHz, $CDCl_3$) δ 201.8, 175.6, 136.9, 135.0, 130.4, 128.7, 128.0, 126.6, 123.9, 80.7, 68.9, 56.7, 53.8, 29.5, 28.9, 26.0, 23.4; IR (KBr): 3367, 2970, 2942, 2863, 1694, 1352, 1275, 1155, 1059, 764 cm^{-1}; HRMS (EI) m/z $[M + H]^+$ calculated for $C_{18}H_{22}NO_2S_2$: 348.1086, found 348.1089; HPLC: the ee value was determined by HPLC analysis (Chiralpak IB-H, *i*-PrOH/Hexane = 10/90, 1.0 mL/min, 307 nm), retention time: t_{major} = 7.497 min, t_{minor} = 9.330 min, ee = 89%; $[\alpha]_D^{27}$ = – 39.2 (c = 0.72, THF).

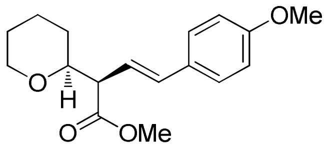

(*R*,*E*)-Methyl 4-(4-methoxyphenyl)-2-((*S*)-tetrahydro-2*H*-pyran-2-yl)but-3-enoate (4i-a)

Product **4i** and **4i'** were converted to **4i-a** and **4i'-a** for separation. Prepared according to general procedure E and purified by silica gel chromatography (CH_2Cl_2/petroleum ether 70:30 to 100:0), a mixture was obtained. Then, to a solution of the mixture in MeOH (1.0 mL) was added a catalytic amount of DMAP. The mixture was stirred at room temperature for 24 h. The solvent was removed in vacuo and the residue was purified by silica gel chromatography (EtOAc/petroleum ether 10:90). Yield: 49% (28.4 mg), **4i-a**/**4i'-a** = 1.7:1. ^{1}H NMR (500 MHz, $CDCl_3$) δ 7.34 (d, J = 8.7 Hz, 2H), 6.85 (d, J = 8.7 Hz, 2H), 6.41 (d, J = 15.9 Hz, 1H), 6.18 (dd, J = 15.9, 9.4 Hz, 1H), 4.02–3.98 (m, 1H), 3.81 (s, 3H), 3.75–3.70 (m, 4H), 3.43 (td, J = 11.3, 2.6 Hz, 1H), 3.22 (dd, J = 9.3, 6.2 Hz, 1H), 1.88–1.82 (m, 1H), 1.58–1.44 (m, 5H); ^{13}C NMR (126 MHz, $CDCl_3$) δ 173.1, 159.4, 133.2, 129.9, 127.8, 122.6, 114.1, 78.7, 69.2, 55.8, 55.5, 52.2, 29.6, 25.9, 23.5; IR (KBr): 2958, 2927, 2873, 1738, 1247, 1149, 1039, 765 cm^{-1}; HRMS (EI) m/z $[M + H]^+$ calculated for $C_{17}H_{23}O_4$: 291.1591, found 291.1592; HPLC: the ee value was determined by HPLC analysis (Chiralpak AD-H, *i*-PrOH/Hexane = 5/95, 1.0 mL/min, 257 nm), retention time: t_{minor} = 8.690 min, t_{major} = 12.737 min, ee = 95%; $[\alpha]_D^{27}$ = + 21.3 (c = 0.56, THF).

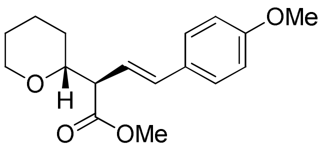

(*R*,*E*)-Methyl 4-(4-methoxyphenyl)-2-((*R*)-tetrahydro-2*H*-pyran-2-yl)but-3-enoate (4i'-a)

^{1}H NMR (500 MHz, $CDCl_3$) δ 7.30 (d, *J* = 8.7 Hz, 2H), 6.85 (d, *J* = 8.7 Hz, 2H), 6.47 (d, *J* = 15.8 Hz, 1H), 5.96 (dd, *J* = 15.8, 9.6 Hz, 1H), 4.02–3.96 (m, 1H), 3.81 (s, 3H), 3.73 (s, 3H), 3.69–3.62 (m, 1H), 3.45 (td, *J* = 11.7, 2.3 Hz, 1H), 3.19 (t, *J* = 9.6 Hz, 1H), 1.87–1.81 (m, 1H), 1.76–1.70 (m, 1H), 1.60–1.46 (m, 3H), 1.25–1.20 (m, 1H); ^{13}C NMR (126 MHz, $CDCl_3$) δ 173.6, 159.6, 133.8, 129.5, 127.8, 121.6, 114.2, 78.7, 69.0, 56.9, 55.5, 52.2, 29.7, 26.0, 23.4; IR (KBr): 2958, 2928, 2874, 1739, 1246, 1150, 1038, 764 cm^{-1}; HRMS (EI) m/z [M + H]$^+$ calculated for $C_{17}H_{23}O_4$: 291.1591, found 291.1595; HPLC: the ee value was determined by HPLC analysis (Chiralpak AD-H, *i*-PrOH/Hexane = 5/95, 1.0 mL/min, 256 nm), retention time: t_{minor} = 9.357 min, t_{major} = 11.003 min, ee = 91%; $[\alpha]_D^{27}$ = + 63.9 (c = 0.52, THF).

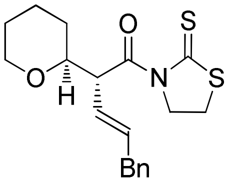

(*R*,*E*)-5-Phenyl-2-((*S*)-tetrahydro-2*H*-pyran-2-yl)-1-(2-thioxothiazolidin-3-yl)pent-3-en-1-one (4j)

Prepared according to general procedure E and purified by silica gel chromatography (CH_2Cl_2/petroleum ether 75:25 to 100:0). Yield: 60% (43.3 mg), **6j**/**6j'** = 1.6:1. ^{1}H NMR (500 MHz, $CDCl_3$) δ 7.31–7.26 (m, 2H), 7.26–7.14 (m, 3H), 5.87–5.68 (m, 2H), 5.35 (dd, *J* = 8.4, 5.1 Hz, 1H), 4.61–4.45 (m, 2H), 4.05–3.94 (m, 1H), 3.88–3.75 (m, 1H), 3.48–3.38 (m, 3H), 3.26 (dt, *J* = 11.0, 8.0 Hz, 1H), 3.15 (ddd, *J* = 11.1, 7.5, 6.5 Hz, 1H), 1.90–1.78 (m, 1H), 1.60–1.43 (m, 5H); ^{13}C NMR (126 MHz, $CDCl_3$) δ 201.8, 174.6, 140.5, 134.2, 128.7, 128.6, 126.2, 126.2, 78.7, 69.1, 57.0, 53.3, 39.3, 29.3, 28.5, 26.0, 23.5; IR (KBr): 3400, 3012, 2982, 2881, 1698, 1361, 1269, 1158, 1055, 719 cm^{-1}; HRMS (EI) m/z [M + H]$^+$ calculated for $C_{19}H_{24}NO_2S_2$: 362.1243, found

362.1240; HPLC: the ee value was determined by HPLC analysis (Chiralpak IB-H, *i*-PrOH/Hexane = 5/95, 1.0 mL/min, 307 nm), retention time: t_{minor} = 11.947 min, t_{major} = 12.547 min, ee = 90%; $[\alpha]_D^{27}$ = – 38.2 (c = 0.39, THF).

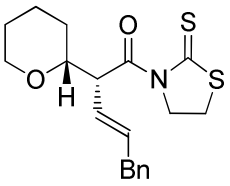

(*R*,*E*)-5-Phenyl-2-((*S*)-tetrahydro-2*H*-pyran-2-yl)-1-(2-thioxothiazolidin-3-yl)pent-3-en-1-one (4j')

^{1}H NMR (500 MHz, $CDCl_3$) δ 7.35–7.27 (m, 2H), 7.23–7.18 (m, 1H), 7.18–7.09 (m, 2H), 5.89 (dt, *J* = 15.2, 6.9 Hz, 1H), 5.48 (ddt, *J* = 15.4, 9.0, 1.4 Hz, 1H), 5.20 (t, *J* = 9.2 Hz, 1H), 4.61 (ddd, *J* = 12.1, 7.7, 4.5 Hz, 1H), 4.45 (ddd, *J* = 11.9, 10.0, 7.6 Hz, 1H), 3.95–3.85 (m, 1H), 3.77–3.62 (m, 1H), 3.45–3.30 (m, 4H), 3.15 (ddd, *J* = 11.0, 7.6, 4.5 Hz, 1H), 1.87–1.80 (m, 1H), 1.79–1.71 (m, 1H), 1.60–1.45 (m, 3H), 1.26–1.18 (m, 1H); ^{13}C NMR (126 MHz, $CDCl_3$) δ 201.6, 175.9, 140.1, 135.0, 128.7, 128.6, 126.3, 125.5, 80.5, 68.8, 56.7, 53.7, 39.2, 29.3, 29.0, 26.0, 23.4; IR (KBr): 3401, 3014, 2983, 2879, 1697, 1363, 1270, 1157, 1059, 721 cm^{-1}; HRMS (EI) m/z [M + H]$^+$ calculated for $C_{19}H_{24}NO_2S_2$: 362.1243, found 362.1243; HPLC: the ee value was determined by HPLC analysis (Chiralpak IB-H, *i*-PrOH/Hexane = 10/90, 1.0 mL/min, 309 nm), retention time: t_{major} = 7.203 min, t_{minor} = 10.197 min, ee = 87%; $[\alpha]_D^{27}$ = – 44.5 (c = 0.47, THF).

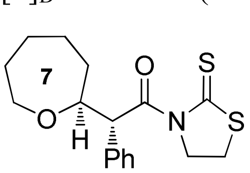

(*R*)-2-((*S*)-Oxepan-2-yl)-2-phenyl-1-(2-thioxothiazolidin-3-yl)ethanone (4k)

Prepared according to general procedure E using THF/CH_2Cl_2 (3:1) as solvent and purified by silica gel chromatography (CH_2Cl_2/EtOAc 100:0 to 99:1). Yield: 58% (38.9 mg), **4k/4k'** = 1.5:1. ^{1}H NMR (500 MHz, $CDCl_3$) δ 7.42 (d, *J* = 7.2 Hz, 2H), 7.32 (t, *J* = 7.3 Hz, 2H), 7.29–7.25 (m, 1H), 6.15 (d, *J* = 8.6 Hz, 1H), 4.66–4.40 (m, 2H), 4.19 (ddd, *J* = 10.0, 8.9, 3.5 Hz, 1H), 3.65 (ddd, *J* = 12.1, 7.9, 4.1 Hz, 1H),

3.41–3.29 (m, 1H), 3.25–3.08 (m, 2H), 1.94 (dtd, *J* = 9.8, 6.5, 3.2 Hz, 1H), 1.81 – 1.57 (m, 5H), 1.54–1.45 (m, 2H); ^{13}C NMR (126 MHz, $CDCl_3$) δ 201.8, 174.7, 136.6, 129.7, 128.4, 127.6, 81.1, 68.6, 57.0, 55.4, 34.1, 31.0, 28.2, 26.8, 26.5; IR (KBr): 3412, 2977, 2931, 2867, 1703, 1454, 1342, 1161, 1142, 1035, 721 cm^{-1}; HRMS (EI) m/z $[M + H]^+$ calculated for $C_{17}H_{22}NO_2S_2$: 336.1086, found 336.1088; HPLC: the ee value was determined by HPLC analysis (Chiralpak AD-H, *i*-PrOH/Hexane = 10/90, 1.0 mL/min, 310 nm), retention time: t_{minor} = 7.360 min, t_{major} = 7.943 min, ee = 96%; $[\alpha]_D^{25}$ = – 22.9 (c = 0.94, THF).

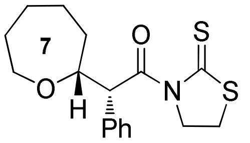

(*R*)-2-((*R*)-Oxepan-2-yl)-2-phenyl-1-(2-thioxothiazolidin-3-yl)ethanone (4k')

^{1}H NMR (500 MHz, $CDCl_3$) δ 7.48 (d, *J* = 7.1 Hz, 2H), 7.31 (t, *J* = 7.3 Hz, 2H), 7.28–7.26 (m, 1H), 5.90 (d, *J* = 10.1 Hz, 1H), 4.64 (ddd, *J* = 12.1, 7.8, 5.7 Hz, 1H), 4.47 (ddd, *J* = 12.0, 8.5, 7.9 Hz, 1H), 4.19 (td, *J* = 9.9, 3.6 Hz, 1H), 3.84 (ddd, *J* = 12.3, 7.6, 4.8 Hz, 1H), 3.66–3.60 (m, 1H), 3.30 (dt, *J* = 10.9, 8.3 Hz, 1H), 3.16 (ddd, *J* = 11.0, 7.7, 5.7 Hz, 1H), 1.82–1.74 (m, 1H), 1.71–1.61 (m, 3H), 1.57–1.47 (m, 1H), 1.47–1.39 (m, 1H), 1.38–1.31 (m, 2H); ^{13}C NMR (126 MHz, $CDCl_3$) δ 201.7, 175.5, 135.7, 129.8, 128.7, 127.8, 83.1, 68.8, 56.9, 55.9, 32.9, 31.2, 28.7, 26.5, 26.3; IR (KBr): 3411, 2976, 2930, 2865, 1704, 1453, 1343, 1160, 1141, 1036, 720 cm^{-1}; HRMS (EI) m/z $[M + H]^+$ calculated for $C_{17}H_{22}NO_2S_2$: 336.1086, found 336.1089; HPLC: the ee value was determined by HPLC analysis (Chiralpak AD-H, *i*-PrOH/Hexane = 10/90, 1.0 mL/min, 309 nm), retention time: t_{minor} = 6.997 min, t_{major} = 8.077 min, ee = 94%; $[\alpha]_D^{25}$ = – 61.9 (c = 0.54, THF).

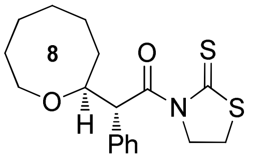

(*R*)-2-((*S*)-Oxocan-2-yl)-2-phenyl-1-(2-thioxothiazolidin-3-yl)ethanone (4l)

Prepared according to general procedure E and purified by silica gel chromatography (CH_2Cl_2/EtOAc 100:0 to 99:1). Yield: 46% (32.1 mg), **4l**/**4l'** = 1.3:1. ^{1}H NMR (500

MHz, $CDCl_3$) δ 7.51–7.38 (m, 2H), 7.31 (t, *J* = 7.3 Hz, 2H), 7.28–7.24 (m, 1H), 5.99 (d, *J* = 8.9 Hz, 1H), 4.57–4.44 (m, 2H), 4.16–4.09 (m, 1H), 3.47 (ddd, *J* = 12.2, 9.0, 3.4 Hz, 1H), 3.26–3.08 (m, 2H), 2.91 (ddd, *J* = 12.0, 5.5, 3.6 Hz, 1H), 1.81–1.57 (m, 7H), 1.55–1.46 (m, 2H), 1.42–1.34 (m, 1H); ^{13}C NMR (126 MHz, $CDCl_3$) δ 201.6, 174.7, 136.5, 129.9, 128.4, 127.5, 81.4, 71.2, 56.8, 56.3, 33.8, 28.2, 27.9, 27.2, 26.9, 25.2; IR (KBr): 3410, 2975, 2930, 2858, 1703, 1455, 1224, 1157, 1142, 1035, 721 cm^{-1}; HRMS (EI) m/z $[M + H]^+$ calculated for $C_{18}H_{24}NO_2S_2$: 350.1243, found 350.1243; HPLC: the ee value was determined by HPLC analysis (Chiralpak AD-H, *i*-PrOH/Hexane = 10/90, 1.0 mL/min, 310 nm), retention time: t_{minor} = 6.443 min, t_{major} = 8.313 min, ee = 92%; $[\alpha]_D^{20}$ = – 10.2 (c = 1.04, THF).

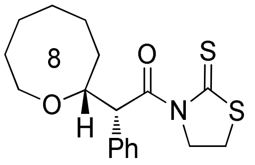

(*R*)-2-((*R*)-Oxocan-2-yl)-2-phenyl-1-(2-thioxothiazolidin-3-yl)ethanone (4l')

^1H NMR (500 MHz, $CDCl_3$) δ 7.48 (d, *J* = 7.1 Hz, 2H), 7.31 (t, *J* = 7.3 Hz, 2H), 7.27–7.23 (m, 1H), 5.85 (d, *J* = 9.9 Hz, 1H), 4.64 (ddd, *J* = 13.1, 7.7, 5.6 Hz, 1H), 4.47 (dt, *J* = 12.0, 8.3 Hz, 1H), 4.27–4.13 (m, 1H), 3.85 (ddd, *J* = 11.8, 8.3, 3.1 Hz, 1H), 3.63 (ddd, *J* = 11.9, 6.6, 3.4 Hz, 1H), 3.37–3.24 (m, 1H), 3.19–3.12 (m, 1H), 1.82–1.72 (m, 1H), 1.72–1.60 (m, 4H), 1.57–1.48 (m, 2H), 1.35–1.28 (m, 3H); ^{13}C NMR (126 MHz, $CDCl_3$) δ 201.6, 175.6, 135.8, 129.8, 128.7, 127.7, 83.2, 71.3, 56.9, 56.4, 31.7, 28.6, 28.3, 27.0, 26.5, 24.3; IR (KBr): 3409, 2974, 2930, 2857, 1703, 1456, 1226, 1158, 1141, 1036, 720 cm^{-1}; HRMS (EI) m/z $[M + H]^+$ calculated for $C_{18}H_{24}NO_2S_2$: 350.1243, found 350.1242; HPLC: the ee value was determined by HPLC analysis (Chiralpak AD-H, *i*-PrOH/Hexane = 10/90, 1.0 mL/min, 310 nm), retention time: t_{minor} = 6.757 min, t_{major} = 7.483 min, ee = 93%; $[\alpha]_D^{20}$ = – 67.5 (c = 1.04, THF).

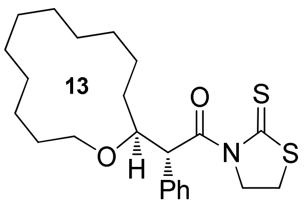

(*R*)-2-((*S*)-Oxacyclotridecan-2-yl)-2-phenyl-1-(2-thioxothiazolidin-3-yl)ethanone (4m)

Prepared according to general procedure E and purified by silica gel chromatography (CH_2Cl_2/petroleum ether 75:25). Yield: 33% (27.5 mg), **4m/4m'** = 1.3:1. ^{1}H NMR (500 MHz, $CDCl_3$) δ 7.51–7.37 (m, 2H), 7.37–7.28 (m, 2H), 7.28–7.27 (m, 1H), 5.96 (d, *J* = 8.7 Hz, 1H), 4.58 (dt, *J* = 12.1, 7.6 Hz, 1H), 4.54–4.44 (m, 1H), 3.74 (td, *J* = 9.0, 1.7 Hz, 1H), 3.29–3.01 (m, 3H), 2.84–2.71 (m, 1H), 1.72–1.62 (m, 2H), 1.51–1.30 (m, 14H), 1.24–1.17 (m, 2H), 1.15–1.07 (m, 2H); ^{13}C NMR (126 MHz, $CDCl_3$) δ 201.3, 174.5, 136.3, 130.0, 128.4, 127.6, 83.8, 70.5, 56.7, 55.5, 34.7, 29.4, 28.3, 26.9, 26.8, 25.9, 24.6, 24.3, 23.7, 23.0, 22.9; IR (KBr): 3409, 2974, 2929, 2856, 1703, 1455, 1225, 1156, 1142, 1030, 720 cm^{-1}; HRMS (EI) m/z $[M + H]^+$ calculated for $C_{23}H_{34}NO_2S_2$: 420.2520, found 420.2525; HPLC: the ee value was determined by HPLC analysis (Chiralpak IB-H, *i*-PrOH/Hexane = 3/97, 1.0 mL/min, 309 nm), retention time: t_{minor} = 7.170 min, t_{major} = 7.687 min, ee = 96%; $[\alpha]_D^{22}$ = + 52.3 (c = 0.20, THF).

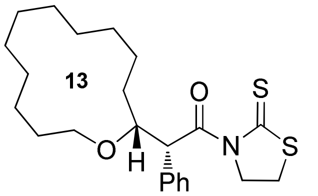

(*R*)-2-((*R*)-Oxacyclotridecan-2-yl)-2-phenyl-1-(2-thioxothiazolidin-3-yl)ethanone (4m')

^{1}H NMR (500 MHz, $CDCl_3$) δ 7.53–7.45 (m, 2H), 7.35–7.29 (m, 2H), 7.27–7.24 (m, 1H), 5.82 (d, *J* = 9.8 Hz, 1H), 4.60 (ddd, *J* = 12.1, 7.7, 5.2 Hz, 1H), 4.46 (ddd, *J* = 11.9, 9.3, 7.6 Hz, 1H), 3.94 (ddd, *J* = 9.6, 7.2, 2.1 Hz, 1H), 3.65 (dt, *J* = 8.8, 3.7 Hz, 1H), 3.53 (ddd, *J* = 11.1, 9.2, 2.1 Hz, 1H), 3.32 (ddd, *J* = 10.9, 9.3, 7.7 Hz, 1H), 3.17 (ddd, *J* = 11.0, 7.5, 5.2 Hz, 1H), 1.73–1.60 (m, 2H), 1.44–1.24 (m, 17H), 1.14–1.06 (m, 1H); ^{13}C NMR (126 MHz, $CDCl_3$) δ 201.5, 176.0, 135.9, 129.9, 128.6, 127.7, 83.9, 70.0, 56.9, 54.6, 31.6, 30.0, 28.9, 27.1, 26.8, 25.7, 25.1, 24.4, 23.9, 23.7, 21.8; IR (KBr): 3408, 2973, 2929, 2857, 1703, 1455, 1227, 1157, 1141, 1031, 720 cm^{-1}; HRMS (EI) m/z $[M + H]^+$ calculated for $C_{23}H_{34}NO_2S_2$: 420.2520, found 420.2521;

HPLC: the ee value was determined by HPLC analysis (Chiralpak IB-H, *i*-PrOH/Hexane = 3/97, 1.0 mL/min, 307 nm), retention time: t_{minor} = 6.753 min, t_{major} = 7.603 min, ee = 94%; $[\alpha]_D^{22}$ = – 66.7 (c = 0.40, THF).

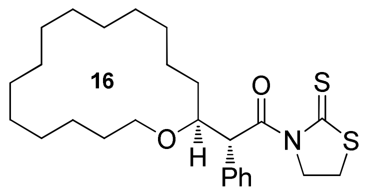

(*R*)-2-((*S*)-Oxacyclohexadecan-2-yl)-2-phenyl-1-(2-thioxothiazolidin-3-yl)ethanone (4n)

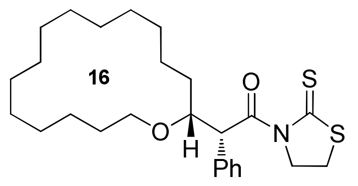

(*R*)-2-((*R*)-Oxacyclohexadecan-2-yl)-2-phenyl-1-(2-thioxothiazolidin-3-yl)ethanone (4n')

Prepared according to general procedure E and purified by silica gel chromatography (CH_2Cl_2/petroleum ether 75:25). Yield: 29% (26.7 mg), **4n**/**4n'** = 1.1:1 (**4n** and **4n'** are inseparable). ^1H NMR (500 MHz, $CDCl_3$) δ 7.48 (d, *J* = 7.1 Hz, 3H), 7.44 (d, *J* = 7.0 Hz, 2H), 7.31 (t, *J* = 7.3 Hz, 5H), 7.27–7.25 (m, 2.5H), 6.07 (d, *J* = 8.1 Hz, 1H), 5.92 (d, *J* = 9.7 Hz, 1.5H), 4.70–4.54 (m, 2.5H), 4.54–4.42 (m, 2.5H), 4.08–3.97 (m, 1.5H), 3.82 (t, *J* = 8.3 Hz, 1H), 3.53 (dt, *J* = 8.9, 5.5 Hz, 1.5H), 3.45 (td, *J* = 8.5, 5.2 Hz, 1.5H), 3.30 (ddd, *J* = 10.8, 9.1, 7.8 Hz, 1.5H), 3.25–3.12 (m, 3.5H), 3.04 (td, *J* = 8.5, 4.5 Hz, 1H), 2.99–2.88 (m, 1H), 1.64–1.52 (m, 5H), 1.44–1.19 (m, 60H); ^{13}C NMR (126 MHz, $CDCl_3$) δ 201.5, 201.4, 176.1, 174.5, 136.2, 135.8, 130.1, 129.9, 128.6, 128.4, 127.7, 127.6, 83.6, 83.4, 71.5, 69.8, 56.9, 56.8, 55.1, 53.9, 34.3, 30.7, 29.8, 28.8, 28.4, 28.1, 28.1, 28.0, 27.9, 27.6, 27.5, 27.3, 27.1, 26.8, 26.7, 26.7, 26.6, 26.5, 26.5, 26.5, 26.3, 26.2, 26.2, 25.3, 25.3, 25.0, 23.1; IR (KBr): 3408, 2973, 2928, 2856, 1703, 1454, 1226, 1158, 1140, 1030, 722 cm^{-1}; HRMS (EI) m/z $[M + H]^+$ calculated for $C_{26}H_{40}NO_2S_2$: 462.2495, found 462.2498.

Characteristic peaks of **4n**. ^1H NMR (500 MHz, $CDCl_3$) δ7.44 (d, *J* = 7.0 Hz, 2H),

6.07 (d, *J* = 8.1 Hz, 1H), 3.82 (t, *J* = 8.3 Hz, 1H), 3.04 (td, *J* = 8.5, 4.5 Hz, 1H), 2.99–2.88 (m, 1H); ^{13}C NMR (126 MHz, $CDCl_3$) δ 201.4, 174.5, 136.2, 130.1, 128.4, 127.6, 83.6, 71.5, 56.8, 55.1; HPLC: the ee value was determined by HPLC analysis (Chiralpak IB-H, *i*-PrOH/Hexane = 1/99, 1.0 mL/min, 309 nm), retention time: t_{minor} = 9.223 min, t_{major} = 10.367 min, ee = 95%.

Characteristic peaks of **4n'**. ^{1}H NMR (500 MHz, $CDCl_3$) δ 7.48 (d, *J* = 7.1 Hz, 2H), 5.92 (d, *J* = 9.7 Hz, 1H), 4.08–3.97 (m, 1H), 3.53 (dt, *J* = 8.9, 5.5 Hz, 1H), 3.45 (td, *J* = 8.5, 5.2 Hz, 1H), 3.30 (ddd, *J* = 10.8, 9.1, 7.8 Hz, 1H); ^{13}C NMR (126 MHz, $CDCl_3$) δ 201.5, 176.1, 135.8, 129.9, 128.6, 127.7, 83.4, 69.8, 56.9, 53.9; HPLC: the ee value was determined by HPLC analysis (Chiralpak IB-H, *i*-PrOH/Hexane = 1/99, 1.0 mL/min, 308 nm), retention time: t_{minor} = 8.580 min, t_{major} = 10.890 min, ee = 94%.

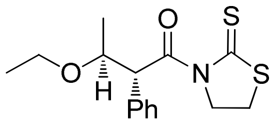

(2*R*,3*S*)-3-Ethoxy-2-phenyl-1-(2-thioxothiazolidin-3-yl)butan-1-one (6a)

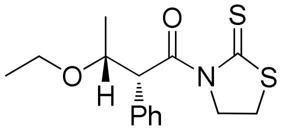

(2*R*,3*R*)-3-Ethoxy-2-phenyl-1-(2-thioxothiazolidin-3-yl)butan-1-one (6a')

Prepared according to general procedure E and purified by silica gel chromatography (CH_2Cl_2/petroleum ether 75:25 to 100:0). Yield: 65% (40.2 mg), **6a**/**6a'** = 1:1 (**6a** and **6a'** are inseparable). ^{1}H NMR (500 MHz, $CDCl_3$) δ 7.48 (d, *J* = 7.2 Hz, 3H), 7.43 (d, *J* = 7.2 Hz, 2H), 7.32 (t, *J* = 7.3 Hz, 5H), 7.29–7.26 (m, 2.5H), 6.04 (d, *J* = 7.9 Hz, 1H), 5.66 (d, *J* = 9.8 Hz, 1.5H), 4.62 (ddd, *J* = 12.4, 7.7, 5.0 Hz, 1.5H), 4.55 (dt, *J* = 12.1, 7.6 Hz, 1H), 4.50–4.43 (m, 2.5H), 4.12–4.06 (m, 1.5H), 4.05–3.99 (m, 1H), 3.66 (dq, *J* = 9.0, 7.0 Hz, 1.5H), 3.47–3.39 (m, 2.5H), 3.35–3.30 (m, 1.5H), 3.22–3.08 (m, 4.5H), 1.27 (d, *J* = 6.1 Hz, 3H), 1.21–1.15 (m, 4.5H), 0.99–0.92 (m, 7.5H); ^{13}C NMR (126 MHz, $CDCl_3$) δ 201.5, 201.4, 175.8, 174.5, 136.1, 135.5, 130.0, 129.8, 128.6, 128.3, 127.7, 127.5, 79.9, 78.1, 65.3, 64.7, 57.1, 56.9, 56.9, 56.2, 28.9, 28.4, 18.9, 17.4, 15.8, 15.4; IR (KBr): 3398, 2977, 2929, 2861, 1703, 1456, 1155, 1028, 718 cm^{-1};

HRMS (EI) m/z $[M + H]^+$ calculated for $C_{15}H_{20}NO_2S_2$: 310.0930, found 310.0933.

Characteristic peaks of **6a**. ^{1}H NMR (500 MHz, $CDCl_3$) δ7.43 (d, *J* = 7.2 Hz, 2H), 6.04 (d, *J* = 7.9 Hz, 1H), 4.55 (dt, *J* = 12.1, 7.6 Hz, 1H), 4.05–3.99 (m, 1H), 1.27 (d, *J* = 6.1 Hz, 3H); ^{13}C NMR (126 MHz, $CDCl_3$) δ 201.5, 174.5, 136.1, 130.0, 128.3, 127.5, 78.1, 65.3, 56.9, 56.2, 28.4, 18.9, 15.4; HPLC: the ee value was determined by HPLC analysis (Chiralpak IB-H, *i*-PrOH/Hexane = 5/95, 1.0 mL/min, 306 nm), retention time: t_{minor} = 7.407 min, t_{major} = 7.673 min, ee = 90%.

Characteristic peaks of **6a'**. ^{1}H NMR (500 MHz, $CDCl_3$) δ 7.48 (d, *J* = 7.2 Hz, 2H), 5.66 (d, *J* = 9.8 Hz, 1H), 4.62 (ddd, *J* = 12.4, 7.7, 5.0 Hz, 1H), 4.12–4.06 (m, 1H), 3.66 (dq, *J* = 9.0, 7.0 Hz, 1H), 3.35–3.30 (m, 1H), 1.21–1.15 (m, 3H); ^{13}C NMR (126 MHz, $CDCl_3$) δ 201.4, 175.8, 135.5, 129.8, 128.6, 127.7, 79.9, 64.7, 57.1, 56.9, 28.9, 17.4, 15.8; HPLC: the ee value was determined by HPLC analysis (Chiralpak IB-H, *i*-PrOH/Hexane = 5/95, 1.0 mL/min, 305 nm), retention time: t_{minor} = 6.260 min, t_{major} = 7.067 min, ee = 88%.

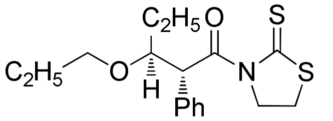

(2*R*,3*S*)-2-Phenyl-3-propoxy-1-(2-thioxothiazolidin-3-yl)pentan-1-one (6b)

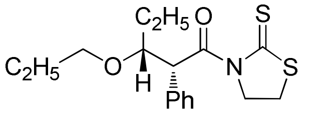

(2*R*,3*R*)-2-phenyl-3-propoxy-1-(2-thioxothiazolidin-3-yl)pentan-1-one (6b')

Prepared according to general procedure E and purified by silica gel chromatography (CH_2Cl_2/petroleum ether 75:25 to 100:0). Yield: 62% (41.8 mg), **6b**/**6b'** = 1:1 (**6b** and **6b'** are inseparable, and a part of **6b** decomposed after purification). ^{1}H NMR (500 MHz, $CDCl_3$) δ 7.54–7.48 (m, 2.8H), 7.47–7.41 (m, 2H), 7.37–7.29 (m, 4.8H), 7.28–7.24 (m, 2.4H), 6.08 (d, *J* = 8.0 Hz, 1H), 5.96 (d, *J* = 10.0 Hz, 1.4H), 4.65 (ddd, *J* = 12.6, 7.7, 5.1 Hz, 1.4H), 4.56 (dt, *J* = 12.1, 7.5 Hz, 1H), 4.52–4.40 (m, 2.4H), 4.04–3.97 (m, 1.4H), 3.88–3.80 (m, 1H), 3.52 (dt, *J* = 8.8, 6.4 Hz, 1.4H), 3.35 (dt, *J* = 7.3, 5.9 Hz, 1.4H), 3.32–3.13 (m, 5.8H), 2.91 (dt, *J* = 8.8, 6.7 Hz, 1H), 1.62–1.54 (m, 4.4H), 1.52–1.46 (m, 1.4H), 1.37–1.29 (m, 2.4H), 1.18–1.12 (m, 1.4H), 0.97–0.91 (m,

7.2H), 0.83 (t, J = 7.4 Hz, 4.2H), 0.69 (t, J = 7.4 Hz, 3H); ^{13}C NMR (126 MHz, $CDCl_3$) δ 201.7, 201.5, 175.9, 174.6, 136.0, 135.7, 130.2, 129.9, 128.6, 128.3, 127.7, 127.6, 84.2, 83.7, 73.2, 71.8, 56.9, 56.9, 54.6, 53.4, 28.7, 28.3, 26.4, 23.7, 23.3, 23.3, 10.9, 10.7, 10.2, 8.4; IR (KBr): 3395, 2976, 2927, 2859, 1704, 1458, 1156, 1029, 720 cm^{-1}; HRMS (EI) m/z $[M + H]^+$ calculated for $C_{17}H_{24}NO_2S_2$: 338.1243, found 338.1244.

Characteristic peaks of **6b**. ^{1}H NMR (500 MHz, $CDCl_3$) δ 7.47–7.41 (m, 2H), 6.08 (d, J = 8.0 Hz, 1H), 4.56 (dt, J = 12.1, 7.5 Hz, 1H), 3.88–3.80 (m, 1H), 2.91 (dt, J = 8.8, 6.7 Hz, 1H), 0.69 (t, J = 7.4 Hz, 3H); ^{13}C NMR (126 MHz, $CDCl_3$) δ 201.5, 174.6, 136.0, 130.2, 128.3, 127.6, 83.7, 73.2, 56.9, 54.6, 28.3, 26.4, 23.3, 10.7, 10.2; HPLC: the ee value was determined by HPLC analysis (Chiralpak IB-H, *i*-PrOH/Hexane = 2/98, 1.0 mL/min, 307 nm), retention time: t_{minor} = 7.803 min, t_{major} = 8.673 min, ee = 92%.

Characteristic peaks of **6b'**. ^{1}H NMR (500 MHz, $CDCl_3$) δ 7.54–7.48 (m, 2H), 5.96 (d, J = 10.0 Hz, 1H), 4.65 (ddd, J = 12.6, 7.7, 5.1 Hz, 1H), 4.04–3.97 (m, 1H), 3.52 (dt, J = 8.8, 6.4 Hz, 1H), 3.35 (dt, J = 7.3, 5.9 Hz, 1H), 1.52–1.46 (m, 1H), 1.18–1.12 (m, 1H), 0.83 (t, J = 7.4 Hz, 3H); ^{13}C NMR (126 MHz, $CDCl_3$) δ 201.7, 175.9, 135.7, 129.9, 128.6, 127.7, 84.2, 71.8, 56.9, 53.4, 28.7, 23.7, 23.3, 10.9, 8.4; HPLC: the ee value was determined by HPLC analysis (Chiralpak IB-H, *i*-PrOH/Hexane = 2/98, 1.0 mL/min, 308 nm), retention time: t_{minor} = 6.923 min, t_{major} = 8.107 min, ee = 93%.

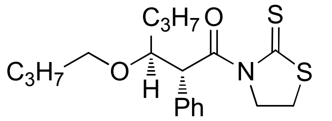

(2*R*,3*S*)-3-Butoxy-2-phenyl-1-(2-thioxothiazolidin-3-yl)hexan-1-one (6c)

Prepared according to general procedure E and purified by silica gel chromatography (CH_2Cl_2/petroleum ether 75:25 to 100:0). Yield: 57% (41.6 mg), **6c**/**6c'** = 1:1. ^{1}H NMR (500 MHz, $CDCl_3$) δ 7.43 (d, J = 7.2 Hz, 2H), 7.31 (t, J = 7.2 Hz, 2H), 7.27–7.24 (m, 1H), 6.07 (d, J = 7.9 Hz, 1H), 4.57 (dt, J = 12.1, 7.5 Hz, 1H), 4.49 (dt, J = 12.1, 7.4 Hz, 1H), 3.87 (td, J = 8.0, 2.9 Hz, 1H), 3.29–3.11 (m, 3H), 2.94 (dd, J = 15.4, 6.6 Hz, 1H), 1.63–1.56 (m, 1H), 1.54–1.46 (m, 2H), 1.38–1.34 (m, 1H),

1.31–1.27 (m, 2H), 1.18–1.08 (m, 2H), 0.91 (t, *J* = 7.0 Hz, 3H), 0.77 (t, *J* = 7.4 Hz, 3H); ^{13}C NMR (126 MHz, $CDCl_3$) δ 201.5, 174.6, 136.1, 130.2, 128.3, 127.6, 82.6, 71.4, 56.9, 55.0, 36.1, 32.3, 28.3, 19.4, 19.2, 14.4, 14.0; IR (KBr): 3390, 2971, 2927, 2857, 1703, 1457, 1150, 1027, 719 cm^{-1}; HRMS (EI) m/z $[M + H]^+$ calculated for $C_{19}H_{28}NO_2S_2$: 366.1556, found 366.1555; HPLC: the ee value was determined by HPLC analysis (Chiralcel OD-H, *i*-PrOH/Hexane = 2/98, 1.0 mL/min, 311 nm), retention time: t_{minor} = 8.120 min, t_{major} = 9.013 min, ee = 94%; $[\alpha]_D^{24}$ = + 4.8 (c = 0.20, THF).

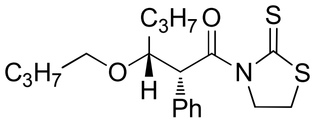

(2*R*,3*R*)-3-Butoxy-2-phenyl-1-(2-thioxothiazolidin-3-yl)hexan-1-one (6c')

^1H NMR (500 MHz, $CDCl_3$) δ 7.54–7.43 (m, 2H), 7.31 (t, *J* = 7.3 Hz, 2H), 7.27–7.23 (m, 1H), 5.95 (d, *J* = 9.9 Hz, 1H), 4.64 (ddd, *J* = 12.6, 7.7, 5.2 Hz, 1H), 4.45 (ddd, *J* = 12.0, 9.2, 7.7 Hz, 1H), 4.10–3.96 (m, 1H), 3.55 (dt, *J* = 8.8, 6.3 Hz, 1H), 3.42 (dt, *J* = 8.9, 6.6 Hz, 1H), 3.29 (ddd, *J* = 10.9, 9.2, 7.8 Hz, 1H), 3.16 (ddd, *J* = 11.0, 7.6, 5.3 Hz, 1H), 1.55–1.47 (m, 2H), 1.42–1.32 (m, 4H), 1.28–1.25 (m, 1H), 1.20–1.13 (m, 1H), 0.93 (t, *J* = 7.4 Hz, 3H), 0.79 (t, *J* = 7.0 Hz, 3H); ^{13}C NMR (126 MHz, $CDCl_3$) δ 201.6, 175.8, 135.7, 129.9, 128.6, 127.7, 83.4, 70.1, 56.9, 54.0, 33.4, 32.6, 28.7, 19.6, 17.7, 14.4, 14.2; IR (KBr): 3391, 2973, 2927, 2858, 1703, 1458, 1153, 1028, 720 cm^{-1}; HRMS (EI) m/z $[M + H]^+$ calculated for $C_{19}H_{28}NO_2S_2$: 366.1556, found 366.1555; HPLC: the ee value was determined by HPLC analysis (Chiralcel OD-H, *i*-PrOH/Hexane = 3/97, 1.0 mL/min, 307 nm), retention time: t_{minor} = 6.480 min, t_{major} = 8.147 min, ee = 95%; $[\alpha]_D^{24}$ = – 51.7 (c = 0.44, THF).

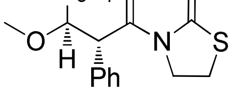

(2*R*,3*S*)-3-Methoxy-2-phenyl-1-(2-thioxothiazolidin-3-yl)hexan-1-one (6d)

Prepared according to general procedure E and purified by silica gel chromatography (CH_2Cl_2/petroleum ether 75:25 to 100:0). Yield: 45% (29.0 mg), **6d**/**6d'** = 1:1. ^1H NMR (500 MHz, $CDCl_3$) δ 7.43 (d, *J* = 7.0 Hz, 2H), 7.33 (t, *J* = 7.2 Hz, 2H),

7.30–7.27 (m, 1H), 6.11 (d, *J* = 7.8 Hz, 1H), 4.60–4.46 (m, 2H), 3.81 (td, *J* = 7.9, 3.2 Hz, 1H), 3.22–3.15 (m, 2H), 3.07 (s, 3H), 1.63–1.55 (m, 1H), 1.54–1.47 (m, 2H), 1.42–1.33 (m, 1H), 0.91 (t, *J* = 7.1 Hz, 3H); ^{13}C NMR (126 MHz, $CDCl_3$) δ 201.6, 174.5, 135.9, 130.1, 128.5, 127.7, 83.9, 59.0, 56.9, 54.6, 35.6, 28.3, 19.1, 14.4; IR (KBr): 3402, 2981, 2932, 2859, 1704, 1460, 1216, 1150, 1028, 719 cm^{-1}; HRMS (EI) m/z [M + H]$^+$ calculated for $C_{16}H_{22}NO_2S_2$: 324.1086, found 324.1088; HPLC: the ee value was determined by HPLC analysis (Chiralpak IB-H, *i*-PrOH/Hexane = 5/95, 1.0 mL/min, 310 nm), retention time: t_{minor} = 6.957 min, t_{major} = 8.623 min, ee = 95%; $[\alpha]_D^{24}$ = + 3.6 (c = 0.30, THF).

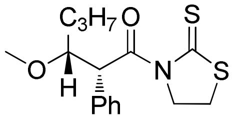

(2*R*,3*R*)-3-Methoxy-2-phenyl-1-(2-thioxothiazolidin-3-yl)hexan-1-one (6d')

^{1}H NMR (500 MHz, $CDCl_3$) δ 7.46 (d, *J* = 7.1 Hz, 2H), 7.32 (t, *J* = 7.3 Hz, 2H), 7.29–7.26 (m, 1H), 6.05 (d, *J* = 9.9 Hz, 1H), 4.74–4.56 (m, 1H), 4.49 (dt, *J* = 12.1, 7.9 Hz, 1H), 3.97 (ddd, *J* = 9.6, 6.1, 3.1 Hz, 1H), 3.39 (s, 3H), 3.28 (dt, *J* = 11.0, 8.1 Hz, 1H), 3.20–3.11 (m, 1H), 1.42–1.32 (m, 2H), 1.29–1.25 (m, 1H), 1.20–1.12 (m, 1H), 0.79 (t, *J* = 7.0 Hz, 3H); ^{13}C NMR (126 MHz, $CDCl_3$) δ 201.8, 175.5, 135.6, 129.8, 128.7, 127.8, 84.4, 58.2, 56.9, 53.9, 32.9, 28.5, 17.7, 14.4; IR (KBr): 3405, 2983, 2934, 2859, 1703, 1461, 1216, 1153, 1028, 721 cm^{-1}; HRMS (EI) m/z [M + H]$^+$ calculated for $C_{16}H_{22}NO_2S_2$: 324.1086, found 324.1089; HPLC: the ee value was determined by HPLC analysis (Chiralpak IB-H, *i*-PrOH/Hexane = 5/95, 1.0 mL/min, 307 nm), retention time: t_{minor} = 7.227 min, t_{major} = 7.787 min, ee = 94%; $[\alpha]_D^{24}$ = – 52.2 (c = 0.40, THF).

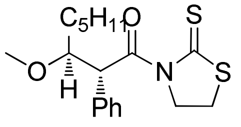

(2*R*,3*S*)-3-Methoxy-2-phenyl-1-(2-thioxothiazolidin-3-yl)octan-1-one (6e)

Prepared according to general procedure E and purified by silica gel chromatography (CH_2Cl_2/petroleum ether 75:25 to 100:0). Yield: 50% (35.2 mg), **6e**/**6e'** = 1:1. ^{1}H NMR (500 MHz, $CDCl_3$) δ 7.48–7.40 (m, 2H), 7.37–7.30 (m, 2H), 7.30–7.27 (m, 1H),

6.11 (d, J = 7.8 Hz, 1H), 4.53 (dtd, J = 14.4, 12.1, 7.3 Hz, 2H), 3.81 (td, J = 7.7, 3.7 Hz, 1H), 3.25–3.13 (m, 2H), 3.07 (s, 3H), 1.62–1.45 (m, 3H), 1.38–1.27 (m, 5H), 0.88 (t, J = 7.0 Hz, 3H); ^{13}C NMR (126 MHz, $CDCl_3$) δ 201.5, 174.5, 135.9, 130.1, 128.5, 127.7, 84.1, 59.0, 56.9, 54.6, 33.3, 32.1, 28.3, 25.5, 22.8, 14.3; IR (KBr): 3400, 2979, 2931, 2858, 1703, 1459, 1215, 1151, 1025, 719 cm^{-1}; HRMS (EI) m/z $[M + H]^+$ calculated for $C_{18}H_{26}NO_2S_2$: 352.1399, found 352.1340; HPLC: the ee value was determined by HPLC analysis (Chiralpak IB-H, *i*-PrOH/Hexane = 5/95, 1.0 mL/min, 311 nm), retention time: t_{minor} = 6.970 min, t_{major} = 9.377 min, ee = 93%; $[\alpha]_D^{20}$ = + 5.6 (c = 0.16, THF).

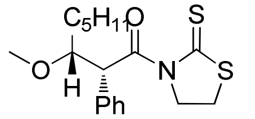

(2*R*,3*R*)-3-Methoxy-2-phenyl-1-(2-thioxothiazolidin-3-yl)octan-1-one (6e')

^{1}H NMR (500 MHz, $CDCl_3$) δ 7.54–7.38 (m, 2H), 7.36–7.29 (m, 2H), 7.29–7.27 (m, 1H), 6.05 (d, J = 9.9 Hz, 1H), 4.63 (ddd, J = 12.1, 7.7, 6.4 Hz, 1H), 4.49 (dt, J = 12.1, 7.9 Hz, 1H), 4.01–3.90 (m, 1H), 3.39 (s, 3H), 3.28 (dt, J = 11.0, 7.9 Hz, 1H), 3.16 (ddd, J = 11.0, 7.7, 6.4 Hz, 1H), 1.41–1.14 (m, 8H), 0.83 (t, J = 7.2 Hz, 3H); ^{13}C NMR (126 MHz, $CDCl_3$) δ 201.8, 175.5, 135.6, 129.8, 128.7, 127.9, 84.6, 58.1, 56.9, 53.9, 32.1, 30.5, 28.5, 23.9, 22.7, 14.2; IR (KBr): 3401, 2980, 2932, 2859, 1703, 1461, 1215, 1153, 1027, 720 cm^{-1}; HRMS (EI) m/z $[M + H]^+$ calculated for $C_{18}H_{26}NO_2S_2$: 352.1399, found 352.1341; HPLC: the ee value was determined by HPLC analysis (Chiralpak IB-H, *i*-PrOH/Hexane = 5/95, 1.0 mL/min, 309 nm), retention time: t_{minor} = 7.277 min, t_{major} = 8.040 min, ee = 93%; $[\alpha]_D^{20}$ = – 18.7 (c = 0.12, THF).

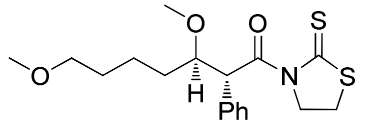

(2*R*,3*S*)-3,7-Dimethoxy-2-phenyl-1-(2-thioxothiazolidin-3-yl)heptan-1-one (6f)

Prepared according to general procedure E and purified by silica gel chromatography (CH_2Cl_2/EtOAc 95:5). Yield: 40% (29.4 mg), **6f/6f'** = 1:1. ^{1}H NMR (500 MHz, $CDCl_3$) δ 7.48–7.39 (m, 2H), 7.38–7.30 (m, 2H), 7.30–7.27 (m, 1H), 6.11 (d, J = 7.8 Hz, 1H), 4.63–4.42 (m, 2H), 3.80 (td, J = 7.8, 3.1 Hz, 1H), 3.36 (t, J = 6.4 Hz, 2H),

3.32 (s, 3H), 3.26–3.13 (m, 2H), 3.07 (s, 3H), 1.63–1.51 (m, 5H), 1.45–1.38 (m, 1H); ^{13}C NMR (126 MHz, $CDCl_3$) δ 201.6, 174.4, 135.8, 130.1, 128.5, 127.7, 84.0, 72.9, 59.1, 58.7, 56.9, 54.5, 33.2, 29.8, 28.3, 22.5; IR (KBr): 3402, 2978, 2930, 2856, 1703, 1462, 1213, 1150, 1023, 718 cm^{-1}; HRMS (EI) m/z $[M + H]^+$ calculated for $C_{18}H_{26}NO_3S_2$: 368.1349, found 368.1347; HPLC: the ee value was determined by HPLC analysis (Chiralpak IB-H, *i*-PrOH/Hexane = 20/80, 1.0 mL/min, 313 nm), retention time: t_{minor} = 6.470 min, t_{major} = 7.213 min, ee = 94%; $[\alpha]_D^{22}$ = + 8.3 (c = 0.22, THF).

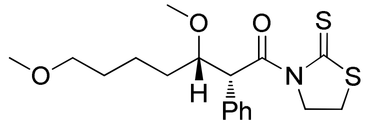

(2*R*,3*R*)-3,7-Dimethoxy-2-phenyl-1-(2-thioxothiazolidin-3-yl)heptan-1-one (6f')

^1H NMR (500 MHz, $CDCl_3$) δ 7.50–7.41 (m, 2H), 7.35–7.29 (m, 2H), 7.29–7.27 (m, 1H), 6.05 (d, *J* = 9.9 Hz, 1H), 4.62 (ddd, *J* = 12.1, 7.7, 6.6 Hz, 1H), 4.49 (dt, *J* = 12.1, 7.9 Hz, 1H), 4.03–3.90 (m, 1H), 3.39 (s, 3H), 3.35–3.21 (m, 6H), 3.16 (ddd, *J* = 11.0, 7.6, 6.5 Hz, 1H), 1.50–1.36 (m, 4H), 1.34–1.31 (m, 1H), 1.23–1.16 (m, 1H); ^{13}C NMR (126 MHz, $CDCl_3$) δ 201.8, 175.4, 135.5, 129.8, 128.7, 127.9, 84.4, 72.8, 58.7, 58.2, 56.9, 53.9, 30.4, 29.8, 28.5, 20.9; IR (KBr): 3403, 2981, 2931, 2857, 1703, 1463, 1213, 1149, 1025, 721 cm^{-1}; HRMS (EI) m/z $[M + H]^+$ calculated for $C_{18}H_{26}NO_3S_2$: 368.1349, found 368.1351; HPLC: the ee value was determined by HPLC analysis (Chiralpak IB-H, *i*-PrOH/Hexane = 20/80, 1.0 mL/min, 309 nm), retention time: t_{minor} = 6.247 min, t_{major} = 6.627 min, ee = 94%; $[\alpha]_D^{22}$ = – 30.3 (c = 0.24, THF).

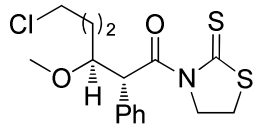

(2*R*,3*S*)-6-Chloro-3-methoxy-2-phenyl-1-(2-thioxothiazolidin-3-yl)hexan-1-one (6g)

Prepared according to general procedure E and purified by silica gel chromatography (CH_2Cl_2/petroleum ether 75:25 to 100:0). Yield: 37% (26.4 mg), **6g**/**6g'** = 1:1. ^1H NMR (500 MHz, $CDCl_3$) δ 7.49–7.40 (m, 2H), 7.36–7.31 (m, 2H), 7.31–7.28 (m, 1H),

6.14 (d, *J* = 8.2 Hz, 1H), 4.57 (dt, *J* = 12.1, 7.8 Hz, 1H), 4.50 (ddd, *J* = 12.1, 7.6, 6.7 Hz, 1H), 3.90–3.74 (m, 1H), 3.60–3.51 (m, 2H), 3.27–3.13 (m, 2H), 3.06 (s, 3H), 2.00–1.92 (m, 1H), 1.90–1.81 (m, 1H), 1.79–1.71 (m, 2H); ^{13}C NMR (126 MHz, $CDCl_3$) δ 201.7, 174.2, 135.7, 130.0, 128.6, 127.8, 83.5, 59.0, 56.9, 54.3, 45.4, 30.5, 28.9, 28.3; IR (KBr): 3403, 2965, 2930, 2854, 1703, 1459, 1220, 1152, 1034, 719 cm^{-1}; HRMS (EI) m/z $[M + H]^+$ calculated for $C_{16}H_{21}ClNO_2S_2$: 358.0697, found 358.0698; HPLC: the ee value was determined by HPLC analysis (Chiralpak IB-H, *i*-PrOH/Hexane = 20/80, 1.0 mL/min, 307 nm), retention time: t_{minor} = 6.530 min, t_{major} = 7.467 min, ee = 93%; $[\alpha]_D^{20}$ = + 1.2 (c = 0.24, THF).

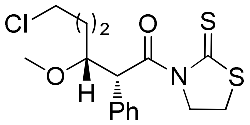

(2*R*,3*R*)-6-Chloro-3-methoxy-2-phenyl-1-(2-thioxothiazolidin-3-yl)hexan-1-one (6g')

^{1}H NMR (500 MHz, $CDCl_3$) δ 7.45 (d, *J* = 7.0 Hz, 2H), 7.33 (t, *J* = 7.2 Hz, 2H), 7.31–7.27 (m, 1H), 6.15 (d, *J* = 9.9 Hz, 1H), 4.63 (dt, *J* = 12.1, 7.4 Hz, 1H), 4.51 (dt, *J* = 12.1, 7.7 Hz, 1H), 4.08–3.98 (m, 1H), 3.44 (t, *J* = 6.5 Hz, 2H), 3.39 (s, 3H), 3.27 (dt, *J* = 11.0, 7.7 Hz, 1H), 3.17 (dt, *J* = 11.0, 7.4 Hz, 1H), 1.86–1.73 (m, 2H), 1.67–1.61 (m, 1H), 1.33–1.29 (m, 1H); ^{13}C NMR (126 MHz, $CDCl_3$) δ 201.9, 175.1, 135.1, 129.7, 128.9, 128.1, 83.6, 58.0, 56.9, 53.4, 45.4, 28.4, 27.4, 27.4; IR (KBr): 3403, 2967, 2931, 2855, 1703, 1460, 1218, 1153, 1033, 720 cm^{-1}; HRMS (EI) m/z $[M + H]^+$ calculated for $C_{16}H_{21}ClNO_2S_2$: 358.0697, found 358.0698; HPLC: the ee value was determined by HPLC analysis (Chiralpak IB-H, *i*-PrOH/Hexane = 20/80, 1.0 mL/min, 314 nm), retention time: t_{minor} = 6.360 min, t_{major} = 7.890 min, ee = 95%; $[\alpha]_D^{20}$ = – 38.9 (c = 0.24, THF).

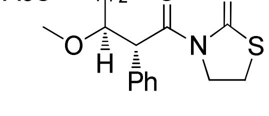

(4*S*,5*R*)-4-Methoxy-6-oxo-5-phenyl-6-(2-thioxothiazolidin-3-yl)hexyl acetate (6h)

Prepared according to general procedure E and purified by silica gel chromatography

(CH_2Cl_2/EtOAc 95:5). Yield: 34% (26.0 mg), **6h**/**6h'** = 1:1. ^{1}H NMR (500 MHz, $CDCl_3$) δ 7.47–7.39 (m, 2H), 7.38–7.31 (m, 2H), 7.31–7.27 (m, 1H), 6.14 (d, *J* = 7.9 Hz, 1H), 4.64–4.45 (m, 2H), 4.06 (t, *J* = 6.1 Hz, 2H), 3.81 (td, *J* = 7.5, 3.9 Hz, 1H), 3.26–3.13 (m, 2H), 3.07 (s, 3H), 2.04 (s, 3H), 1.89–1.77 (m, 1H), 1.74–1.59 (m, 3H); ^{13}C NMR (126 MHz, $CDCl_3$) δ 201.6, 174.2, 171.4, 135.6, 130.0, 128.6, 127.8, 83.6, 64.6, 59.1, 56.9, 54.4, 29.7, 28.3, 25.1, 21.2; IR (KBr): 3411, 2975, 2932, 2859, 1708, 1455, 1221, 1151, 1030, 716 cm^{-1}; HRMS (EI) m/z $[M + H]^+$ calculated for $C_{18}H_{24}NO_4S_2$: 382.1141, found 382.1143; HPLC: the ee value was determined by HPLC analysis (Chiralpak IB-H, *i*-PrOH/Hexane = 20/80, 1.0 mL/min, 311 nm), retention time: t_{minor} = 8.193 min, t_{major} = 9.160 min, ee = 95%; $[\alpha]_D^{23}$ = + 1.4 (c = 0.32, THF).

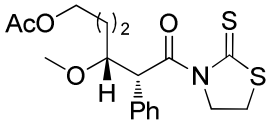

(4*R*,5*R*)-4-Methoxy-6-oxo-5-phenyl-6-(2-thioxothiazolidin-3-yl)hexyl acetate (6h')

^{1}H NMR (500 MHz, $CDCl_3$) δ 7.48–7.40 (m, 2H), 7.35–7.27 (m, 3H), 6.12 (d, *J* = 9.9 Hz, 1H), 4.62 (ddd, *J* = 12.1, 7.7, 6.9 Hz, 1H), 4.50 (dt, *J* = 12.1, 7.7 Hz, 1H), 4.08–3.85 (m, 3H), 3.39 (s, 3H), 3.27 (dt, *J* = 11.0, 7.7 Hz, 1H), 3.19–3.13 (m, 1H), 1.97 (s, 3H), 1.71–1.59 (m, 2H), 1.54–1.42 (m, 1H), 1.24–1.13 (m, 1H); ^{13}C NMR (126 MHz, $CDCl_3$) δ 201.9, 175.1, 171.3, 135.2, 129.7, 128.8, 128.0, 83.7, 64.5, 58.1, 56.9, 53.5, 28.4, 26.5, 23.4, 21.1; IR (KBr): 3410, 2974, 2933, 2859, 1708, 1456, 1222, 1153, 1031, 728 cm^{-1}; HRMS (EI) m/z $[M + H]^+$ calculated for $C_{18}H_{24}NO_4S_2$: 382.1141, found 382.1144; HPLC: the ee value was determined by HPLC analysis (Chiralpak IB-H, *i*-PrOH/Hexane = 20/80, 1.0 mL/min, 311 nm), retention time: t_{minor} = 7.683 min, t_{major} = 8.527 min, ee = 95%; $[\alpha]_D^{23}$ = – 34.1 (c = 0.44, THF).

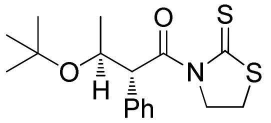

(2R,3S)-3-(*Tert*-butoxy)-2-phenyl-1-(2-thioxothiazolidin-3-yl)butan-1-one (6i)

Prepared according to general procedure E on a 0.5 mmol scale and purified by silica gel chromatography (CH_2Cl_2/petroleum ether 75:25). Yield: 8% (13.5 mg), **6i**/**6i'** =

1:1. ^{1}H NMR (500 MHz, $CDCl_3$) δ 7.44–7.35 (m, 2H), 7.32–7.28 (m, 2H), 7.26–7.23 (m, 1H), 5.85 (d, *J* = 8.0 Hz, 1H), 4.68–4.58 (m, 1H), 4.50 (dt, *J* = 12.1, 7.7 Hz, 1H), 4.12 (dq, *J* = 7.8, 6.0 Hz, 1H), 3.25 (dt, *J* = 11.0, 7.7 Hz, 1H), 3.20–3.13 (m, 1H), 1.26 (d, *J* = 6.0 Hz, 3H), 0.89 (s, 9H); ^{13}C NMR (126 MHz, $CDCl_3$) δ 201.2, 174.7, 136.4, 130.5, 128.1, 127.4, 74.0, 71.0, 57.4, 56.8, 28.4, 28.3, 22.1; HRMS (EI) m/z $[M + H]^+$ calculated for $C_{17}H_{24}NO_2S_2$: 338.1243, found 338.1246; HPLC: the ee value was determined by HPLC analysis (Chiralcel OD-H, *i*-PrOH/Hexane = 1/99, 1.0 mL/min, 305 nm), retention time: t_{minor} = 10.993 min, t_{major} = 12.897 min, ee = 94%; $[\alpha]_D^{27}$ = + 20.2 (c = 0.23, THF).

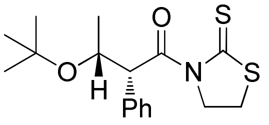

(2*R*,3*R*)-3-(*Tert*-butoxy)-2-phenyl-1-(2-thioxothiazolidin-3-yl)butan-1-one (6i')

^{1}H NMR (500 MHz, $CDCl_3$) δ 7.57–7.44 (m, 2H), 7.31 (t, *J* = 7.3 Hz, 2H), 7.27–7.23 (m, 1H), 5.63 (d, *J* = 9.2 Hz, 1H), 4.67 (ddd, *J* = 11.8, 7.5, 4.1 Hz, 1H), 4.42 (ddd, *J* = 11.9, 10.5, 7.4 Hz, 1H), 4.32 (dq, *J* = 9.2, 6.0 Hz, 1H), 3.34 (td, *J* = 10.6, 7.6 Hz, 1H), 3.16 (ddd, *J* = 11.2, 7.4, 4.1 Hz, 1H), 1.21 (s, 9H), 0.97 (d, *J* = 6.0 Hz, 3H); ^{13}C NMR (126 MHz, $CDCl_3$) δ 201.5, 176.2, 136.0, 130.2, 128.5, 127.6, 74.3, 73.2, 57.7, 56.9, 29.3, 29.0, 21.4; HRMS (EI) m/z $[M + H]^+$ calculated for $C_{17}H_{24}NO_2S_2$: 338.1243, found 338.1242; HPLC: the ee value was determined by HPLC analysis (Chiralcel AD-H, *i*-PrOH/Hexane = 2/98, 1.0 mL/min, 309 nm), retention time: t_{major} = 7.593 min, t_{minor} = 8.610 min, ee = 95%; $[\alpha]_D^{27}$ = – 84.4 (c = 0.32, THF).

Synthetic Applications

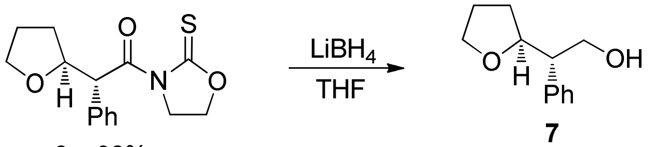

3a, 98% ee **7** 85%, 97% ee

(*S*)-2-Phenyl-2-((*S*)-tetrahydrofuran-2-yl)ethanol (7)

To a stirred solution of **3a** (29 mg, 0.1 mmol) in THF (1 mL) at 0 °C was added $LiBH_4$ (6.5 mg, 0.3 mmol). After 10 min the reaction was quenched by the addition of water (5 mL). The mixture was diluted with 1.0 M NaOH (5 mL) and extracted with EtOAc (3×10 mL). The combined organic layers were washed with brine (10 mL), dried ($MgSO_4$), filtered and concentrated. The residue was purified by flash chromatography on silica gel using MeOH/CH_2Cl_2 (5:95) as eluent to afford **7** (16.3 mg, 85% yield) as a colorless oil. ^{1}H NMR (500 MHz, $CDCl_3$) δ 7.37–7.23 (m, 5H), 4.30–4.18 (m, 1H), 4.03 (dd, *J* = 10.9, 6.7 Hz, 1H), 3.92 (ddd, *J* = 10.9, 6.7, 1.0 Hz, 1H), 3.78 (q, *J* = 7.3 Hz, 1H), 3.74–3.67 (m, 1H), 3.01 (q, *J* = 6.0 Hz, 1H), 2.41 (br s, 1H), 1.96–1.88 (m, 1H), 1.86–1.77 (m, 1H), 1.76–1.66 (m, 1H), 1.60 (dq, *J* = 11.8, 8.4 Hz, 1H); ^{13}C NMR (126 MHz, $CDCl_3$) δ 139.5, 129.3, 128.7, 127.2, 80.7, 68.3, 65.0, 52.2, 29.0, 25.9; IR (KBr): 3281, 2932, 2867, 1450, 1087, 1056, 702 cm^{-1}; HRMS (EI) m/z $[M + H]^+$ calculated for $C_{12}H_{17}O_2$: 193.1223, found 193.1229; HPLC: the ee value was determined by HPLC analysis (Chiralpak AS-H, *i*-PrOH/Hexane = 10/90, 1.0 mL/min, 205 nm), retention time: t_{major} = 9.157 min, t_{minor} = 10.300 min, ee = 97%; $[\alpha]_D^{18}$ = + 8.2 (c = 0.52, THF).

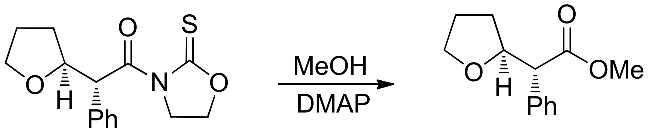

3a, 98% ee **8** 90%, 96% ee

(*R*)-Methyl 2-phenyl-2-((*S*)-tetrahydrofuran-2-yl)acetate (8)

A solution of **3a** (29 mg, 0.1 mmol) and DMAP (1 mg) in MeOH (2 mL) was stirred at room temperature under N_2 for 48 h. The reaction was quenched by addition of sat NH_4Cl (5 mL) and the mixture was extracted with EtOAc (3×10 mL). The combined

organic extracts were washed with brine (10 mL), dried over $MgSO_4$, filtered and concentrated. The residue was purified by flash chromatography on silica gel using EtOAc/petroleum ether (20:80) as eluent to afford **8** (19.8 mg, 90% yield) as a colorless oil. ^{1}H NMR (500 MHz, $CDCl_3$) δ 7.43–7.37 (m, 2H), 7.37–7.31 (m, 2H), 7.31–7.27 (m, 1H), 4.52–4.41 (m, 1H), 3.86–3.79 (m, 1H), 3.75–3.70 (m, 1H), 3.68 (s, 3H), 3.64 (d, *J* = 8.6 Hz, 1H), 2.14 (td, *J* = 12.6, 6.4 Hz, 1H), 1.95–1.83 (m, 2H), 1.69 (dt, *J* = 8.0, 5.7 Hz, 1H); ^{13}C NMR (126 MHz, $CDCl_3$) δ 172.8, 136.8, 128.8, 128.8, 127.7, 80.2, 68.5, 57.0, 52.2, 30.3, 25.8; IR (KBr): 2952, 2871, 1734, 1454, 1203, 1158, 1072, 1021, 700 cm^{-1}; HRMS (EI) m/z $[M + H]^+$ calculated for $C_{13}H_{17}O_3$: 221.1172, found 221.1177; HPLC: the ee value was determined by HPLC analysis (Chiralpak IB-H, *i*-PrOH/Hexane = 2/98, 1.0 mL/min, 208 nm), retention time: t_{minor} = 6.730 min, t_{major} = 7.120 min, ee = 96%; $[\alpha]_D^{28}$ = + 58.6 (c = 0.42, THF).

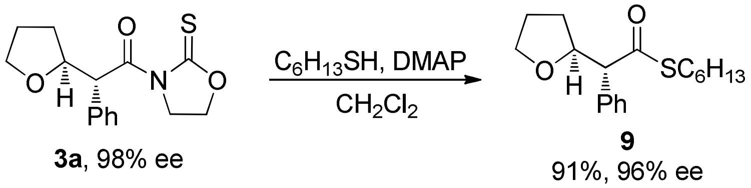

3a, 98% ee

9
91%, 96% ee

(*R*)-*S*-Hexyl 2-phenyl-2-((*S*)-tetrahydrofuran-2-yl)ethanethioate (9)

A solution of **3a** (29 mg, 0.1 mmol), 1-hexanethiol (35 μL, 0.25 mmol) and a catalytic amount of DMAP in CH_2Cl_2 (0.4 mL) was stirred at room temperature under N_2 for 48 h. The reaction mixture was diluted with EtOAc (10 mL), washed with brine (10 mL), dried over $MgSO_4$, filtered and concentrated. The residue was purified by flash chromatography on silica gel using EtOAc/petroleum ether (15:85) as eluent to afford **9** (27.8 mg, 91% yield) as a colorless oil. ^{1}H NMR (500 MHz, $CDCl_3$) δ 7.48–7.37 (m, 2H), 7.37–7.33 (m, 2H), 7.32–7.28 (m, 1H), 4.52 (dd, *J* = 15.4, 7.1 Hz, 1H), 3.86–3.76 (m, 2H), 3.75–3.70 (m, 1H), 2.90–2.77 (m, 2H), 2.12 (dq, *J* = 13.0, 6.6 Hz, 1H), 1.94–1.85 (m, 2H), 1.73 (ddd, *J* = 15.7, 12.3, 7.8 Hz, 1H), 1.56–1.48 (m, 2H), 1.34–1.24 (m, 6H), 0.87 (t, *J* = 6.9 Hz, 3H); ^{13}C NMR (126 MHz, $CDCl_3$) δ 198.9, 136.7, 128.9, 128.8, 127.8, 80.2, 68.4, 65.7, 31.4, 30.3, 29.5, 29.3, 28.6, 25.8, 22.7, 14.2; IR (KBr): 3640, 2927, 2856, 1683, 1455, 1075, 1011, 700 cm^{-1}; HRMS (EI) m/z $[M + H]^+$ calculated for $C_{18}H_{27}O_2S$: 307.1726, found 307.1726; HPLC: the ee value

was determined by HPLC analysis (Chiralpak IB-H, *i*-PrOH/Hexane = 1/99, 1.0 mL/min, 205 nm), retention time: t_{minor} = 5.650 min, t_{major} = 6.210 min, ee = 96%; $[\alpha]_D^{18}$ = + 57.0 (c = 0.46, THF).

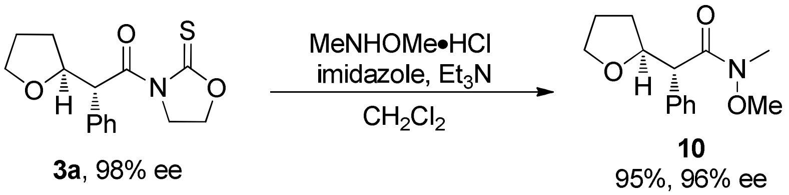

(*R*)-*N*-Methoxy-*N*-methyl-2-phenyl-2-((*S*)-tetrahydrofuran-2-yl)acetamide (10)

To a stirred solution of **3a** (29 mg, 0.1 mmol) in CH_2Cl_2 (2 mL) was added Me(OMe)NH•HCl (49 mg, 0.5 mmol), imidazole (34, 0.5 mmol) and Et_3N (69 μL, 0.5 mmol). The reaction stirred at rt for 2 days. The solvent was removed in vacuo and the residue purified by flash chromatography on silica gel using EtOAc/petroleum ether (30:70) as eluent to afford **10** (23.6 mg, 95% yield) as a colorless oil. ^{1}H NMR (500 MHz, $CDCl_3$) δ 7.42 (d, *J* = 7.3 Hz, 2H), 7.33 (t, *J* = 7.6 Hz, 2H), 7.27–7.23 (m, 1H), 4.44 (dt, *J* = 9.3, 7.0 Hz, 1H), 4.18–3.99 (m, 1H), 3.89–3.81 (m, 1H), 3.70 (dd, *J* = 15.1, 7.2 Hz, 1H), 3.48 (s, 3H), 3.15 (s, 3H), 2.27–2.18 (m, 1H), 2.00–1.88 (m, 2H), 1.66–1.61 (m, 1H); ^{13}C NMR (126 MHz, $CDCl_3$) δ 173.2, 138.1, 128.8, 128.7, 127.4, 81.4, 68.3, 61.5, 53.3, 32.2, 30.9, 25.8; IR (KBr): 2968, 2925, 2875, 1656, 1382, 1070 cm^{-1}; HRMS (EI) m/z $[M + H]^+$ calculated for $C_{14}H_{20}NO_3$: 250.1438, found 250.1439; HPLC: the ee value was determined by HPLC analysis (Chiralpak AD-H, *i*-PrOH/Hexane = 5/95, 1.0 mL/min, 209 nm), retention time: t_{minor} = 9.077 min, t_{major} = 11.130 min, ee = 96%; $[\alpha]_D^{18}$ = + 82.7 (c = 0.44, THF).

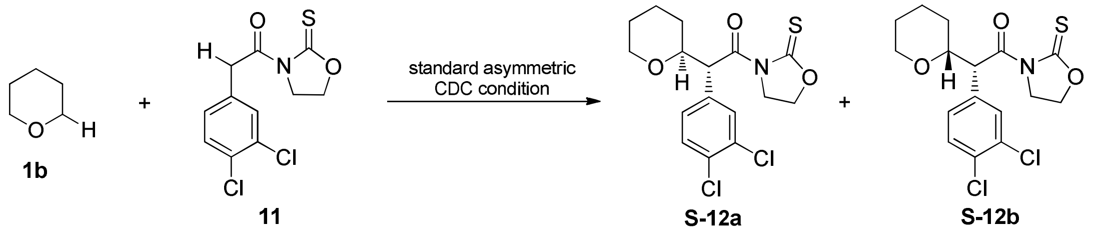

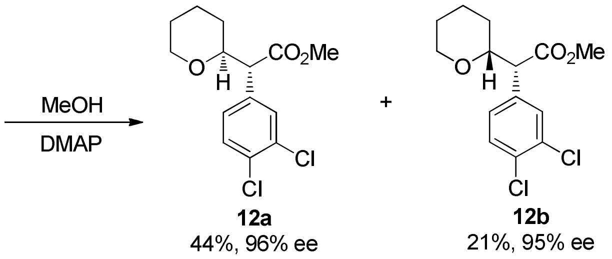

Synthesis of dopamine transporter inhibitors 12a and 12b

According to the general procedure E, **S-12a** and **S-12b** were prepared and separated by silica gel chromatography. Then a solution of **S-12a** or **S-12b** and DMAP (1 mg) in MeOH (1.0 mL) was stirred at room temperature for 24 h. The solvent was removed in vacuo and the residue purified by flash chromatography on silica gel using EtOAc/petroleum ether (20:80) as eluent to afford **12a** (26.6 mg, 44% yield) and **12b** (12.7 mg, 21% yield), respectively. The NMR spectral data is consistent with reported literature values.[2]

(*R*)-Methyl 2-(3,4-dichlorophenyl)-2-((*S*)-tetrahydro-2*H*-pyran-2-yl)acetate (12a)

^{1}H NMR (500 MHz, $CDCl_3$) δ 7.48 (d, *J* = 2.1 Hz, 1H), 7.39 (d, *J* = 8.3 Hz, 1H), 7.23 (dd, *J* = 8.3, 2.1 Hz, 1H), 3.94–3.83 (m, 2H), 3.68 (s, 3H), 3.57 (d, *J* = 8.6 Hz, 1H), 3.35 (td, *J* = 11.2, 3.3 Hz, 1H), 1.90–1.82 (m, 1H), 1.71–1.65 (m, 1H), 1.57–1.45 (m, 3H), 1.36–1.31 (m, 1H); ^{13}C NMR (126 MHz, $CDCl_3$) δ 171.9, 136.7, 132.5, 131.7, 131.1, 130.4, 128.6, 78.4, 69.1, 57.1, 52.5, 30.1, 25.9, 23.4; IR (KBr): 2961, 2873, 1718, 1450, 1208, 1150, 1069, 1009, 801 cm^{-1}; HRMS (EI) m/z $[M + H]^+$ calculated for $C_{14}H_{17}Cl_2O_3$: 303.0549, found 303.0550; HPLC: the ee value was determined by HPLC analysis (Chiralpak AD-H, *i*-PrOH/Hexane = 2/98, 1.0 mL/min, 228 nm), retention time: t_{minor} = 5.707 min, t_{major} = 6.210 min, ee = 96%; $[\alpha]_D^{22}$ = – 7.8 (c = 0.24, THF).

(*R*)-Methyl 2-(3,4-dichlorophenyl)-2-((*R*)-tetrahydro-2*H*-pyran-2-yl)acetate (12b)

^{1}H NMR (500 MHz, $CDCl_3$) δ 7.48 (d, J = 2.1 Hz, 1H), 7.40 (d, J = 8.3 Hz, 1H), 7.21 (dd, J = 8.3, 2.1 Hz, 1H), 4.03–3.99 (m, 1H), 3.88–3.81 (m, 1H), 3.71 (s, 3H), 3.55–3.42 (m, 2H), 1.82–1.74 (m, 1H), 1.57–1.36 (m, 4H), 1.16–1.07 (m, 1H); ^{13}C NMR (126 MHz, $CDCl_3$) δ 172.8, 135.6, 133.0, 132.2, 131.5, 130.8, 130.8, 128.3, 79.4, 69.1, 57.5, 52.5, 29.3, 25.9, 23.3; IR (KBr): 2962, 2873, 1718, 1451, 1211, 1151, 1068, 1010, 799 cm^{-1}; HRMS (EI) m/z $[M + H]^+$ calculated for $C_{14}H_{17}Cl_2O_3$: 303.0549, found 303.0553; HPLC: the ee value was deterfmined by HPLC analysis (Chiralpak IB-H, *i*-PrOH/Hexane = 0.5/99.5, 1.0 mL/min, 227 nm), retention time: t_{major} = 5.327 min, t_{minor} = 5.723 min, ee = 95%; $[\alpha]_D^{22}$ = + 5.1 (c = 0.18, THF).

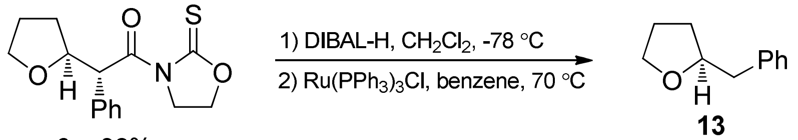

3a, 98% ee

13
62%, 95% ee

(*S*)-2-Benzyltetrahydrofuran (13)

To a solution of **3a** (58 mg, 0.2 mmol) in CH_2Cl_2 (2 mL) at -78 °C was added DIBAL-H (1.5 M in hexanes, 0.2 mL, 0.3 mmol) dropwisely. After 0.5 h, the reaction was quenched with MeOH. The resultant mixture was diluted with EtOAc (10 mL), washed with 1 M HCl (10 mL), dried ($MgSO_4$), filtered and concentrated. The residue was purified by flash chromatography on silica gel using EtOAc/petroleum ether (10:90) as eluent to afford aldehyde. To a solution of aldehyde in benzene (1.5 mL) was added $Ru(PPh_3)_3Cl$ (92.5mg, 1.0 mmol). The mixture was stirred at 70 °C overnight. The solvent was evaporated under reduced pressure and the reside was purified by a flash silica gel using EtOAc/petroleum ether (5:95) as eluent to afford **13** (20.0 mg, 62% yield) as a colorless oil. ^{1}H NMR (500 MHz, $CDCl_3$) δ 7.33–7.27 (m, 2H), 7.27–7.16 (m, 3H), 4.16–4.02 (m, 1H), 3.97–3.88 (m, 1H), 3.75 (td, J = 7.8, 6.2 Hz, 1H), 2.94 (dd, J = 13.6, 6.5 Hz, 1H), 2.76 (dd, J = 13.6, 6.5 Hz, 1H), 2.00–1.80 (m, 3H), 1.60–1.52 (m, 1H); ^{13}C NMR (126 MHz, $CDCl_3$) δ 139.2, 129.4, 128.5, 126.4, 80.3, 68.1, 42.1, 31.2, 25.8; IR (KBr): 2935, 2859, 1455, 1074, 1031, 701 cm^{-1}; HPLC: the ee value was determined by HPLC analysis (Chiralcel OJ-H, *i*-PrOH/Hexane = 0.1/99.9, 1.0 mL/min, 213 nm), retention time: t_{minor} = 23.328 min,

t_{major} = 24.757 min, ee = 95%; $[\alpha]_D^{27}$ = + 2.1 (c = 0.064, $CHCl_3$).

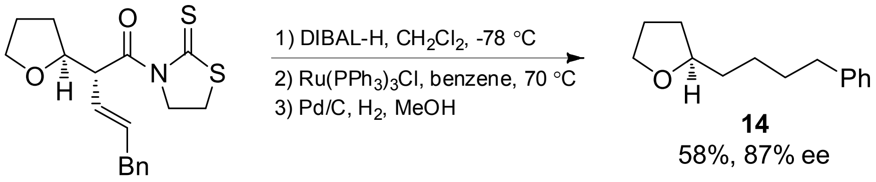

5j, 91% ee

14
58%, 87% ee

(*R*)-2-(4-Phenylbutyl)tetrahydrofuran (14)

To a solution of **5j** (70 mg, 0.2 mmol) in CH_2Cl_2 (2 mL) at -78 °Cwas added DIBAL-H (1.5 M in hexanes, 0.2 mL, 0.3 mmol) slowly. After 0.5 h, the reaction was quenched with MeOH. The resultant mixture was diluted with EtOAc (10 mL), washed with 1 M HCl (10 mL), dried ($MgSO_4$), filtered and concentrated. The residue was purified by flash chromatography on silica gel using EtOAc/petroleum ether (10:90) as eluent to afford aldehyde for next step. To a solution of aldehyde in benzene (1.5 mL) was added $Ru(PPh_3)_3Cl$ (83.2mg, 0.9 mmol). The mixture was stirred at 70 °C overnight. The solvent was evaporated under reduced pressure and the reside was was used as such for next step. A mixture of olefin and Pd/C (3.0 mg) in MeOH (2 mL) was stirred vigorously under a H_2 atmosphere for 5 h at rt. The mixture was filtered through a short celite pad, and the solvent was removed under reduced pressure to afford **14** (23.5 mg, 58% yield) as a colorless oil. ^1H NMR (500 MHz, $CDCl_3$) δ 7.30–7.27 (m, 2H), 7.24–7.14 (m, 3H), 3.95–3.84 (m, 1H), 3.84–3.76 (m, 1H), 3.72 (dd, *J* = 14.3, 7.9 Hz, 1H), 2.67–2.59 (m, 2H), 2.01–1.93 (m, 1H), 1.92–1.80 (m, 2H), 1.68–1.60 (m, 3H), 1.54–1.37 (m, 4H); ^{13}C NMR (126 MHz, $CDCl_3$) δ 142.9, 128.6, 128.4, 125.8, 79.5, 67.8, 36.2, 35.8, 31.8, 31.6, 26.3, 25.9; IR (KBr): 2932, 2857, 1456, 1072, 1033, 699 cm^{-1}; HPLC: the ee value was determined by HPLC analysis (Chiralpak IB-H, *i*-PrOH/Hexane = 0.1/99.9, 1.0 mL/min, 211 nm), retention time: t_{minor} = 10.413 min, t_{major} = 10.767 min, ee = 87%; $[\alpha]_D^{27}$ = + 1.2 (c = 0.070, $CHCl_3$).

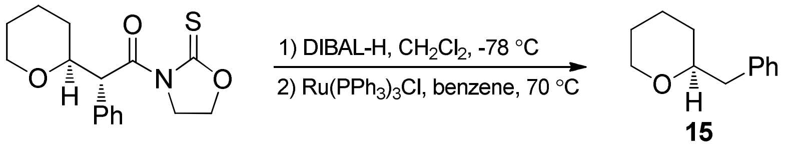

6a, 96% ee

15
60%, 96% ee

(*S*)-2-Benzyltetrahydro-2*H*-pyran (15)

To a solution of **6a** (61 mg, 0.2 mmol) in CH_2Cl_2 (2 mL) at -78 °Cwas added DIBAL-H (1.5 M in hexanes, 0.2 mL, 0.3 mmol) dropwise. After 0.5 h, the reaction was quenched with MeOH. The resultant mixture was diluted with EtOAc (10 mL), washed with 1 M HCl (10 mL), dried ($MgSO_4$), filtered and concentrated. The residue was purified by flash chromatography on silica gel using EtOAc/petroleum ether (10:90) as eluent to afford aldehyde. To a solution of aldehyde in benzene (1.5 mL) was added $Ru(PPh_3)_3Cl$ (92.5mg, 1.0 mmol). The mixture was stirred at 70 °C overnight. The solvent was evaporated under reduced pressure and the reside was purified by a flash silica gel using EtOAc/petroleum ether (5:95) as eluent to afford **15** (21.0 mg, 60% yield) as a colorless oil. ^{1}H NMR (500 MHz, $CDCl_3$) δ 7.34–7.27 (m, 2H), 7.27–7.13 (m, 3H), 4.06–3.93 (m, 1H), 3.50 (dtd, *J* = 10.7, 6.6, 2.0 Hz, 1H), 3.42 (td, *J* = 11.8, 2.4 Hz, 1H), 2.89 (dd, *J* = 13.6, 6.6 Hz, 1H), 2.65 (dd, *J* = 13.6, 6.5 Hz, 1H), 1.86–1.78 (m, 1H), 1.59 (dt, *J* = 12.2, 3.9 Hz, 2H), 1.52–1.32 (m, 3H); ^{13}C NMR (126 MHz, $CDCl_3$) δ 139.0, 129.6, 128.4, 126.3, 79.0, 68.9, 43.4, 31.6, 26.3, 23.7; IR (KBr): 2933, 2857, 1454, 1073, 1032, 701 cm^{-1}; HPLC: the ee value was determined by HPLC analysis (Chiralpak AD-H, *i*-PrOH/Hexane = 0.1/99.9, 1.0 mL/min, 206 nm), retention time: t_{minor} = 7.033 min, t_{major} = 7.997 min, ee = 96%; $[\alpha]_D^{27}$ = + 2.5 (c = 0.090, $CHCl_3$).

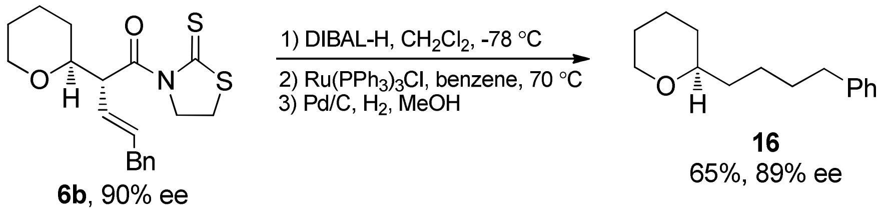

6b, 90% ee

16
65%, 89% ee

(*R*)-2-(4-Phenylbutyl)tetrahydro-2*H*-pyran (16)

To a solution of **6b** (72 mg, 0.2 mmol) in CH_2Cl_2 (2 mL) at -78 °C was added DIBAL-H (1.5 M in hexanes, 0.2 mL, 0.3 mmol) dropwise. After 0.5 h, the reaction was quenched with MeOH. The resultant mixture was diluted with EtOAc (10 mL), washed with 1 M HCl (10 mL), dried ($MgSO_4$), filtered and concentrated. The residue was purified by flash chromatography on silica gel using EtOAc/petroleum ether

(10:90) as eluent to afford aldehyde. To a solution of aldehyde in benzene (1.5 mL) was added $Ru(PPh_3)_3Cl$ (83.2mg, 0.9 mmol). The mixture was stirred at 70 °C overnight. The solvent was evaporated under reduced pressure and the reside was used as such for next step. A mixture of residue and Pd/C (3.0 mg) in MeOH (2 mL) was stirred vigorously under a H_2 atmosphere for 5 h at rt. The mixture was filtered through a short celite pad, and the solvent was removed under reduced pressure to afford **16** (28.0 mg, 65% yield) as a colorless oil. ^1H NMR (500 MHz, $CDCl_3$) δ 7.31–7.27 (m, 2H), 7.25–7.12 (m, 3H), 4.03–3.92 (m, 1H), 3.42 (td, J = 11.6, 2.4 Hz, 1H), 3.28–3.17 (m, 1H), 2.62 (t, J = 7.8 Hz, 2H), 1.88–1.77 (m, 1H), 1.67–1.38 (m, 10H), 1.27–1.20 (m, 1H); ^{13}C NMR (126 MHz, $CDCl_3$) δ 143.0, 128.6, 128.4, 125.8, 78.0, 68.7, 36.7, 36.1, 32.2, 31.8, 26.4, 25.5, 23.8; IR (KBr): 2932, 2856, 1455, 1073, 1035, 700 cm^{-1}; HPLC: the ee value was determined by HPLC analysis (Chiralpak AD-H, *i*-PrOH/Hexane = 1/99, 1.0 mL/min, 209 nm), retention time: t_{major} = 4.700 min, t_{minor} = 5.297 min, ee = 89%; $[\alpha]_D^{27}$ = + 1.4 (c = 0.075, $CHCl_3$).

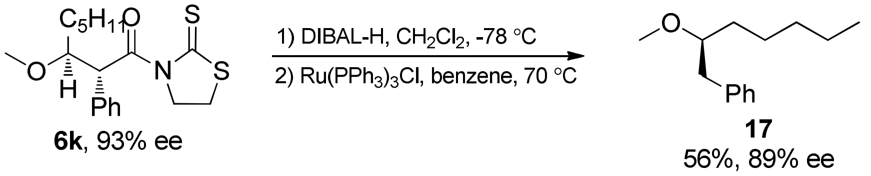

(*S*)-(2-Methoxyheptyl)benzene (17)

To a solution of **6k** (67 mg, 0.2 mmol) in CH_2Cl_2 (2 mL) at -78 °Cwas added DIBAL-H (1.5 M in hexanes, 0.2 mL, 0.3 mmol) dropwise. After 0.5 h, the reaction was quenched with MeOH. The resultant mixture was diluted with EtOAc (10 mL), washed with 1 M HCl (10 mL), dried ($MgSO_4$), filtered and concentrated. The residue was purified by flash chromatography on silica gel using EtOAc/petroleum ether (10:90) as eluent to afford aldehyde. To a solution of aldehyde in benzene (1.5 mL) was added $Ru(PPh_3)_3Cl$ (92.5mg, 1.0 mmol). The mixture was stirred at 70 °C overnight. The solvent was evaporated under reduced pressure and the reside was purified by a flash silica gel using EtOAc/petroleum ether (5:95) as eluent to afford **17** (23.0 mg, 56% yield) as a colorless oil. ^1H NMR (500 MHz, $CDCl_3$) δ 7.33–7.28 (m, 2H), 7.26–7.18 (m, 3H), 3.44–3.26 (m, 4H), 2.85 (dd, J = 13.7, 6.2 Hz, 1H), 2.71 (dd,

J = 13.7, 6.2 Hz, 1H), 1.48–1.41 (m, 2H), 1.35–1.25 (m, 6H), 0.88 (t, J = 7.1 Hz, 3H); ^{13}C NMR (126 MHz, $CDCl_3$) δ 139.5, 129.6, 128.4, 126.2, 82.6, 57.2, 40.4, 33.7, 32.2, 25.2, 22.9, 14.3; IR (KBr): 2694, 2933, 2865, 1461, 1069, 1032, 708 cm^{-1}; HPLC: the ee value was determined by HPLC analysis (Chiralpak IB-H, *i*-PrOH/Hexane = 0/100, 1.0 mL/min, 212 nm), retention time: t_{major} = 7.147 min, t_{minor} = 7.880 min, ee = 92%; $[\alpha]_D^{18}$ = + 10.0 (c = 0.12, THF).

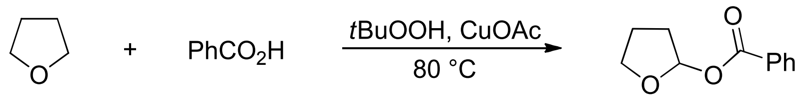

1a **18**, 88%

Tetrahydrofuran-2-yl benzoate (18)

To a solution of PhCOOH (0.5 mmol) in THF (1.0 mL) was added CuOAc (0.005 mmol), and tBuOOH in decane (0.5 mmol). The reaction mixture was heated at 80ºC for 2 h. Removal of the solvent under vacuum followed by purification with flash silica gel chromatography (PE/EA/Et_3N 100:5:1) affored **18** in 88% yield (84.5 mg). ^{1}H NMR (500 MHz, $CDCl_3$) δ 8.14–7.93 (m, 2H), 7.63–7.51 (m, 1H), 7.49–7.38 (m, 2H), 6.77–6.35 (m, 1H), 4.22–4.12 (m, 1H), 4.06–3.96 (m, 1H), 2.25–2.10 (m, 3H), 2.07–1.97 (m, 1H); ^{13}C NMR (126 MHz, $CDCl_3$) δ 166.1, 133.2, 130.6, 129.9, 128.5, 99.9, 69.2, 32.5, 23.2. These data are consistent with reported literature values.[3]

Absolute Configuration Determination

The absolute configuration of **3p** was determined by the X-ray diffraction. A suitable crystal was selected and analyzed on a Bruker APEX-II CCD diffractometer. Further information is contained in the CCDC file 1858037.

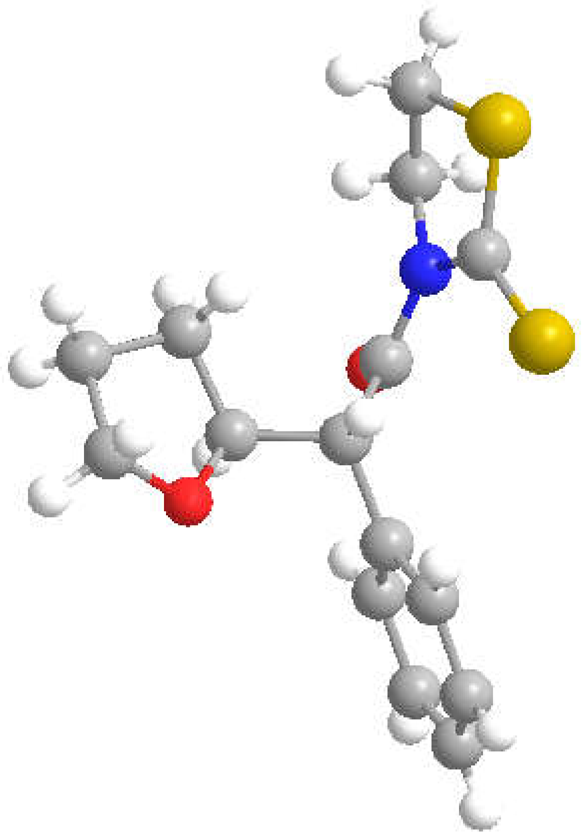

Supplementary figure 1. Absolute configuration determination of 3p

The absolute stereochemistry of **3p’** was determined by the X-ray diffraction. A suitable crystal was selected and analyzed on a Bruker APEX-II CCD diffractometer. Further information is contained in the CCDC file 1858065.

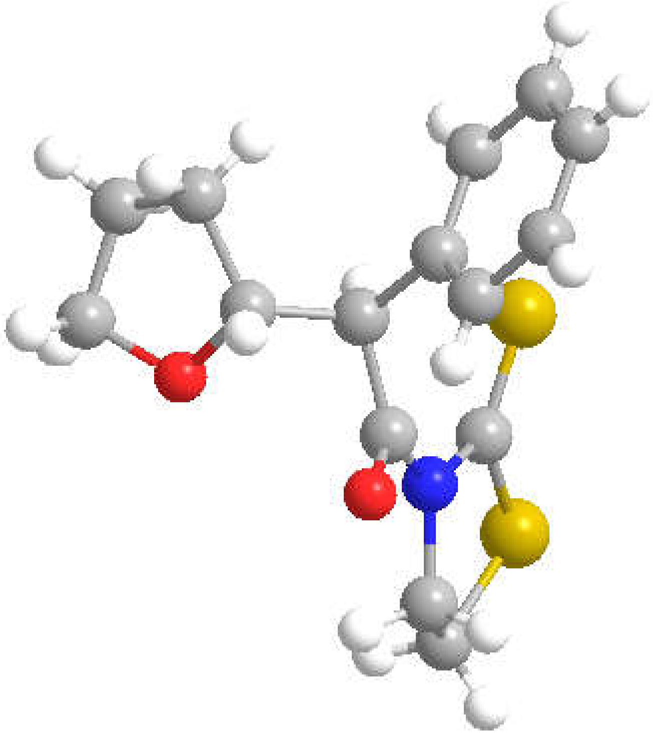

Supplementary figure 2. Absolute configuration determination of 3p’

The absolute stereochemistry of **3s** was determined by the X-ray diffraction. A suitable crystal was selected and analyzed on a Bruker APEX-II CCD diffractometer. Further information is contained in the CCDC file 1858049.

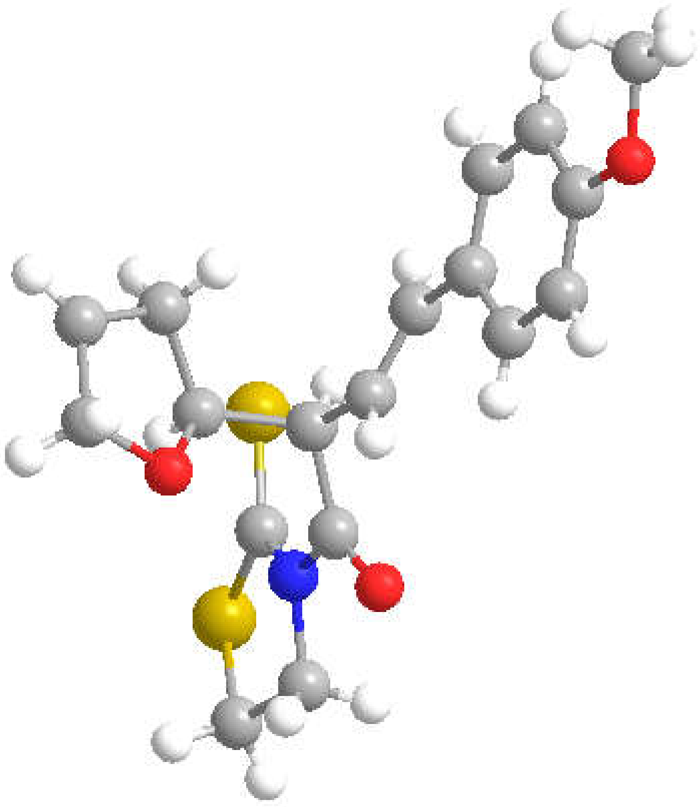

Supplementary figure 3. Absolute configuration determination of 3s

The absolute stereochemistry of **4e** was determined by the X-ray diffraction. A suitable crystal was selected and analyzed on a Bruker APEX-II CCD diffractometer. Further information is contained in the CCDC file 1858043.

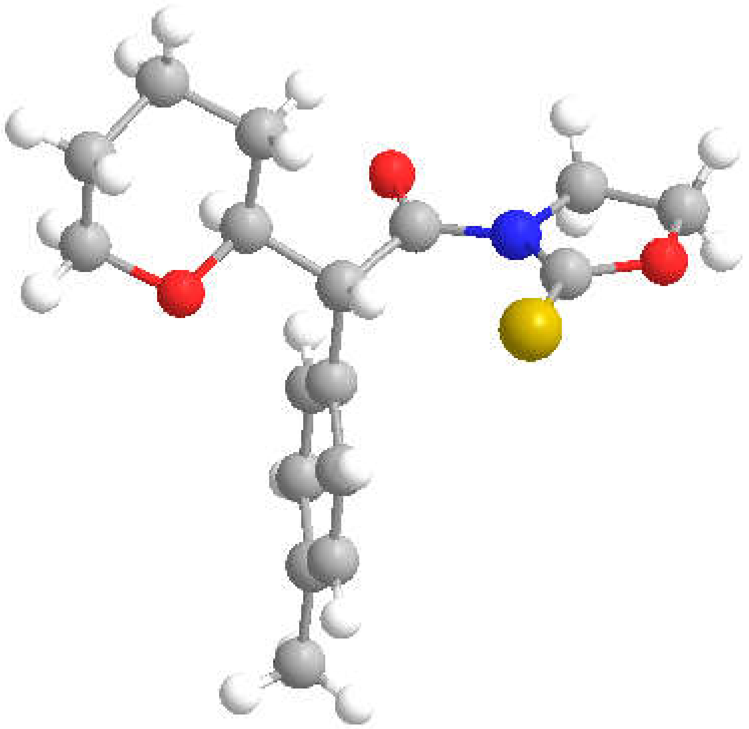

Supplementary figure 4. Absolute configuration determination of 4e

The absolute stereochemistry of **4l** was determined by the X-ray diffraction. A suitable crystal was selected and analyzed on a Bruker APEX-II CCD diffractometer. Further information is contained in the CCDC file 1890776.

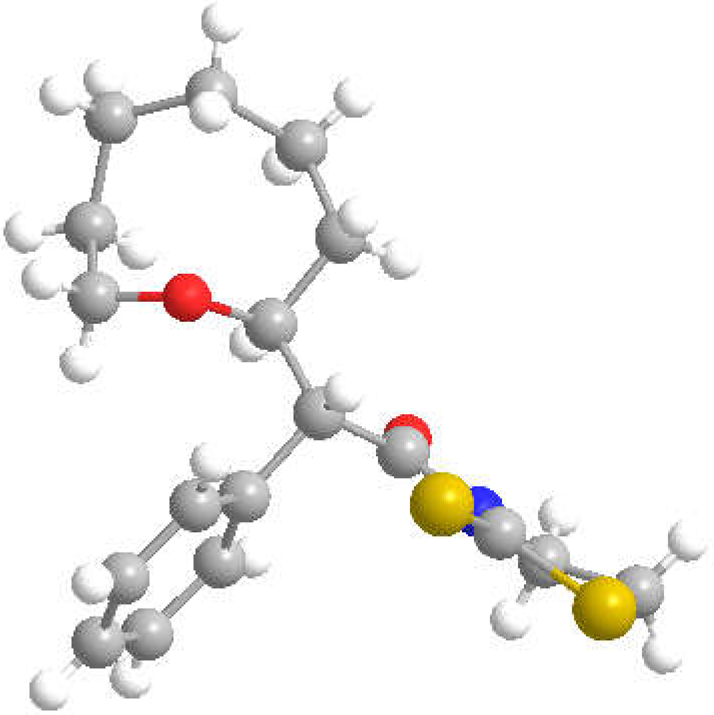

Supplementary figure 5. Absolute configuration determination of 4l

Absolute Configuration Determination of 6e

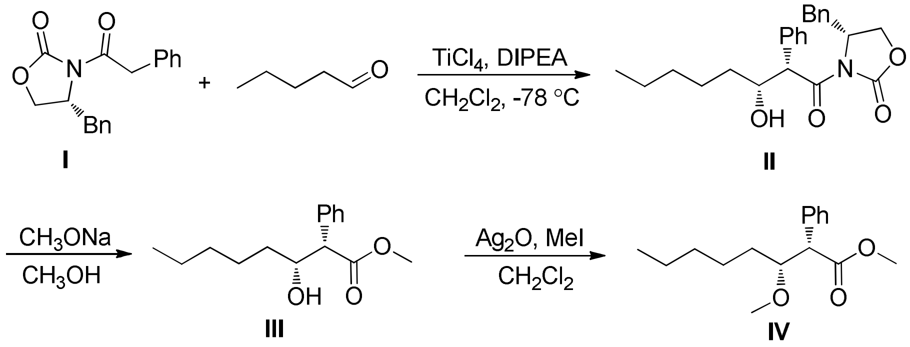

According to known methods,[4] **II** was obtained as the non-Evans syn aldol product. To a solution of **I** (147 mg, 0.5 mmol) in distilled CH_2Cl_2 (3.0 mL) was added $TiCl_4$ (110 uL, 1.0 mmol) at 0 °C followed by diisopropylethylamine (82 uL, 0.5 mmol). The dark red titanium enolate was stirred for 20 min, and then was cooled to -78 °C. Freshly distilled hexanal (67 uL, 0.55 mmol) was added dropwise. The resulting mixture was stirred for 1 h at -78 °C. The reaction was quenched with half-saturated ammonium chloride (6 mL). The resultant mixture was diluted with CH_2Cl_2 (10 mL), and the layers were separated. The organic layer was dried over $MgSO_4$, filtered and the solvent was evaporated under vacuum. The residue was purified by flash chromatography (CH_2Cl_2/petroleum ether 80:20) to afford **II** (128mg, 65% yield)**.**

To a solution of **II** (79 mg, 0.2 mmol) in CH_3OH (3.0 mL) was added a solution of sodium methoxide (16 mg, 0.3 mmol) in CH_3OH (2.0 mL) dropwise at 0 °C. The reaction mixture was stirred at 0 °C for 1.5 h and quenched with saturated aq NH_4Cl (10 mL). The resulting solution was extracted with CH_2Cl_2 (3 × 15 mL). The organic layer was dried over $MgSO_4$, filtered, and concentrated in vacuo. The crude mixture was purified by flash chromatography (EtOAc/petroleum ether 30:70) to give **III** (37.5mg, 75% yield).

To a solution of **III** (25 mg, 0.1 mmol) in Et_2O (1 mL) under argon atmosphere were added molecular sieves 3Å (200 mg), Ag_2O (120 mg, 1.0 mmol) and CH_3I (120 uL, 2.0 mmol) subsequently. The mixture was vigorously stirred at rt for 24 h in dark before filtered through a pad of celite. The solvent was evaporated and the crude product was purificated by flash chromatography (EtOAc/petroleum ether 10:1) to to

give **IV** (25.0 mg, 95% yield). ^{1}H NMR (500 MHz, $CDCl_3$) δ 7.39–7.25 (m, 5H), 3.90 (ddd, J = 9.9, 5.8, 3.7 Hz, 1H), 3.68 (s, 3H), 3.66 (d, J = 10.3 Hz, 1H), 3.43 (s, 3H), 1.43–1.36 (m, 1H), 1.32–1.11 (m, 7H), 0.82 (t, J = 7.2 Hz, 3H); ^{13}C NMR (126 MHz, $CDCl_3$) δ 173.8, 136.1, 128.9, 128.8, 127.9, 82.7, 58.4, 56.9, 52.2, 32.1, 30.5, 23.7, 22.7, 14.2; HRMS (EI) m/z $[M + H]^+$ calculated for $C_{16}H_{25}O_3$: 265.1798, found 265.1797; HPLC: the ee value was determined by HPLC analysis (Chiralpak AD-H, *i*-PrOH/Hexane = 1/99, 1.0 mL/min, 222 nm), retention time: t_{major} = 9.160 min, t_{minor} = 10.260 min, ee = 99.7%; $[\alpha]_D^{18}$ = - 46.7 (c = 0.46, THF).

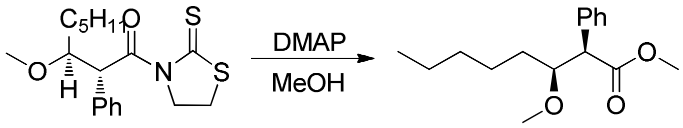

6e **V**

A solution of **6e** (35.0 mg, 0.1 mmol) and DMAP (1 mg) in MeOH (2 mL) was stirred at rt under N_2 for 24 h. The reaction was quenched by addition of sat NH_4Cl (5 mL) and the resultant mixture was extracted with EtOAc. The combined organic extracts were washed with brine (10 mL), dried ($MgSO_4$), filtered and concentrated. The residue was purified by flash chromatography on silica gel using EtOAc/petroleum ether (20:80) as eluent to afford **V** (24.0 mg, 91% yield). The ^{1}H NMR and ^{13}C NMR spectra of **V** are same as those of **IV**; HPLC for **V**: the ee value was determined by HPLC analysis (Chiralpak AD-H, *i*-PrOH/Hexane = 1/99, 1.0 mL/min, 218 nm), retention time: t_{minor} = 9.133 min, t_{major} = 10.310 min, ee = 92%; $[\alpha]_D^{18}$ = + 35.4 (c = 0.22, THF). The retention time of **V** is same as that of **IV**, thus confirming the absolute configuration of **6e**.

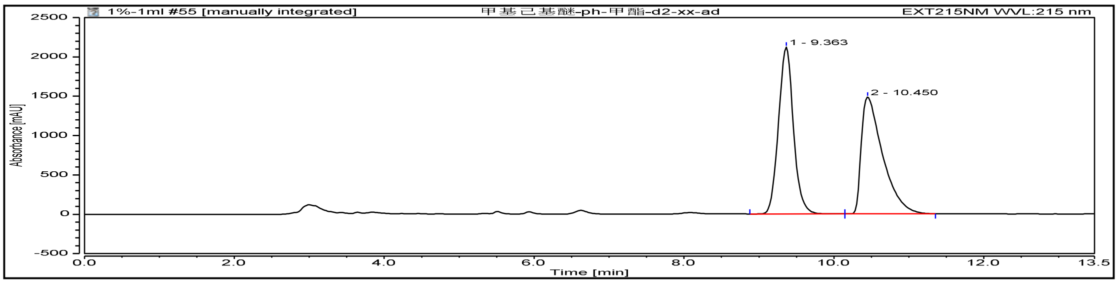

Integration Results					
No.	Peak Name	Retention Time<br>min	Area<br>mAU*min	Relative Area<br>%	Amount<br>n.a.
1		9.363	489.884	49.95	n.a.
2		10.450	490.888	50.05	n.a.
**Total:**			**980.772**	**100.00**	

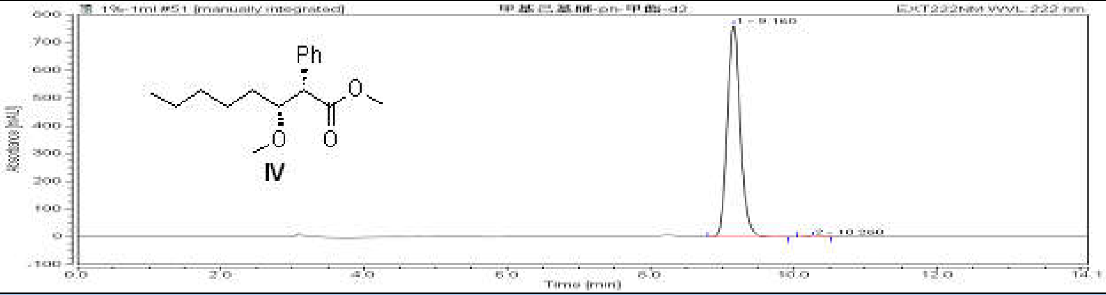

Integration Results					
No.	Peak Name	Retention Time<br>min	Area<br>mAU*min	Relative Area<br>%	Amount<br>n.a.
1		9.160	159.408	99.87	n.a.
2		10.260	0.214	0.13	n.a.
**Total:**			**159.622**	**100.00**	

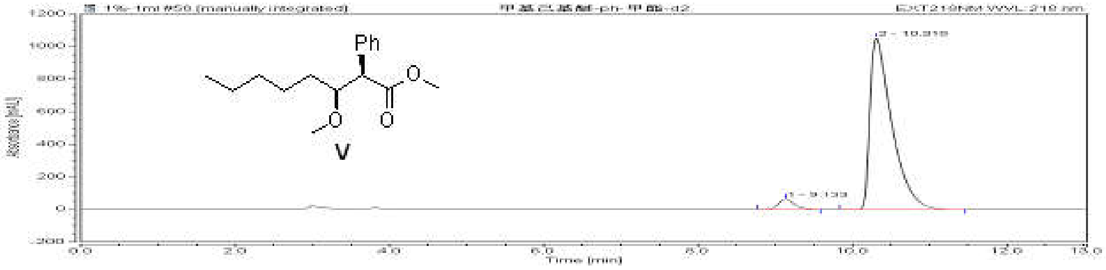

Integration Results					
No.	Peak Name	Retention Time<br>min	Area<br>mAU*min	Relative Area<br>%	Amount<br>n.a.
1		9.133	13.749	3.85	n.a.
2		10.310	343.304	96.15	n.a.
**Total:**			**357.054**	**100.00**	

Supplementary figure 6. Absolute configuration determination of **6e**

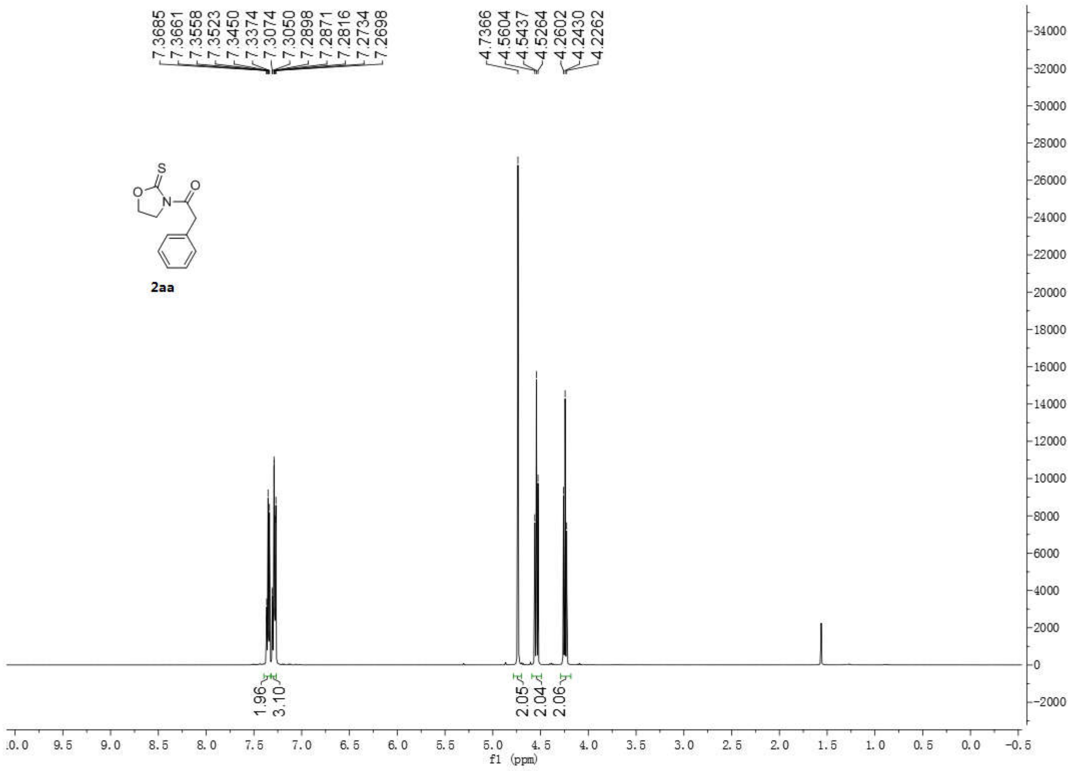

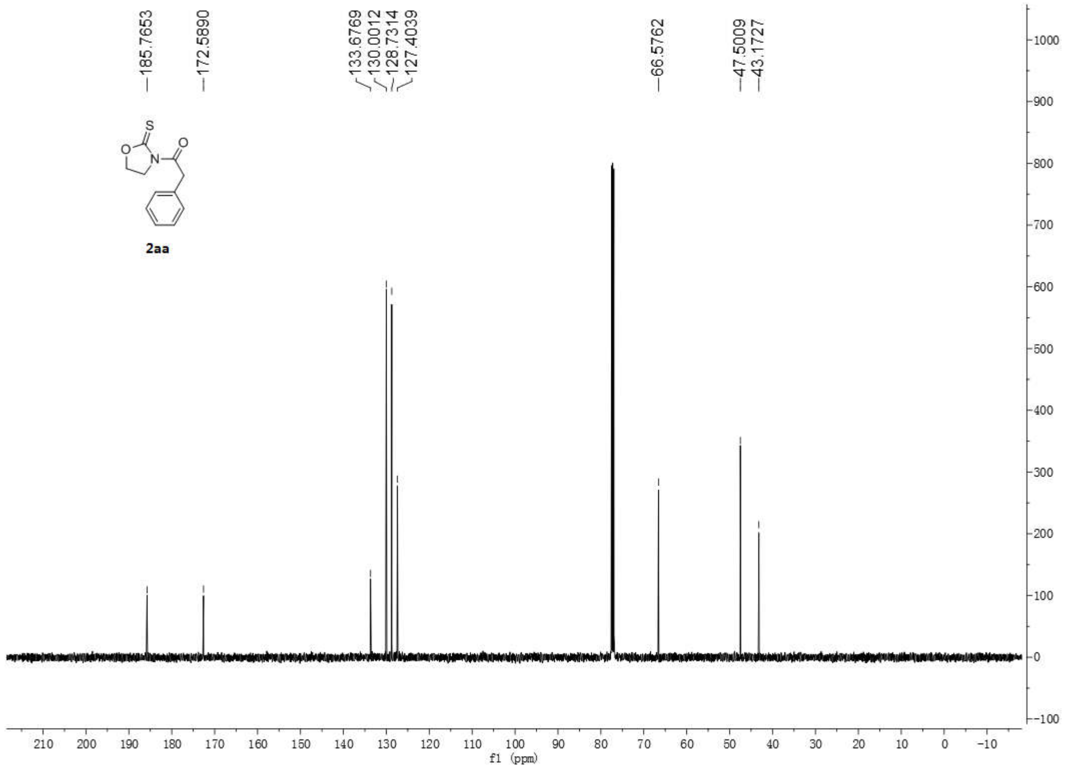

Supplementary figure 7. ^{1}H and ^{13}C NMR spectrum of compound **2aa**

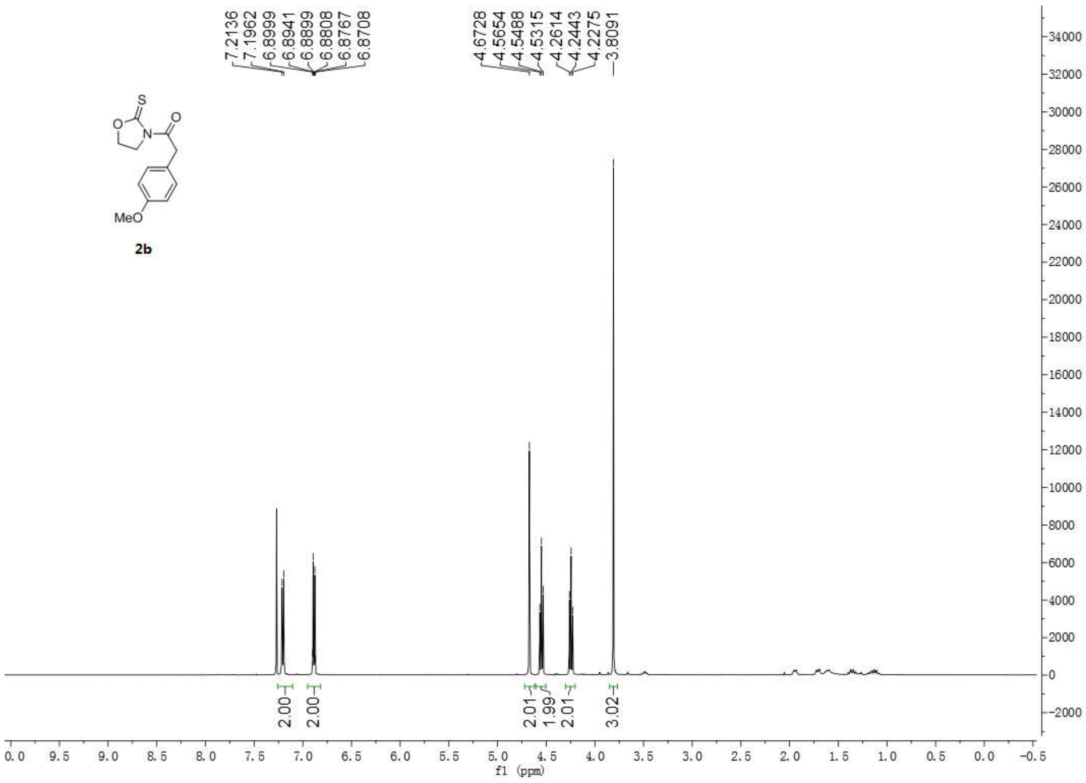

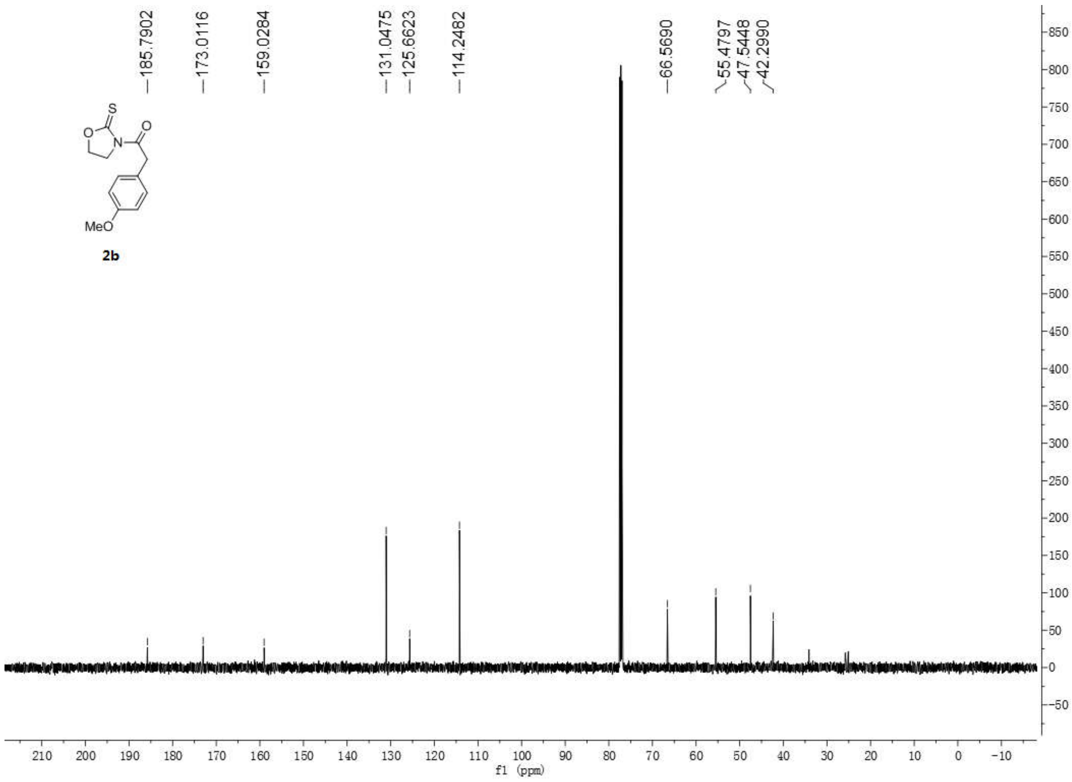

Supplementary figure 8. ^{1}H and ^{13}C NMR spectrum of compound **2b**

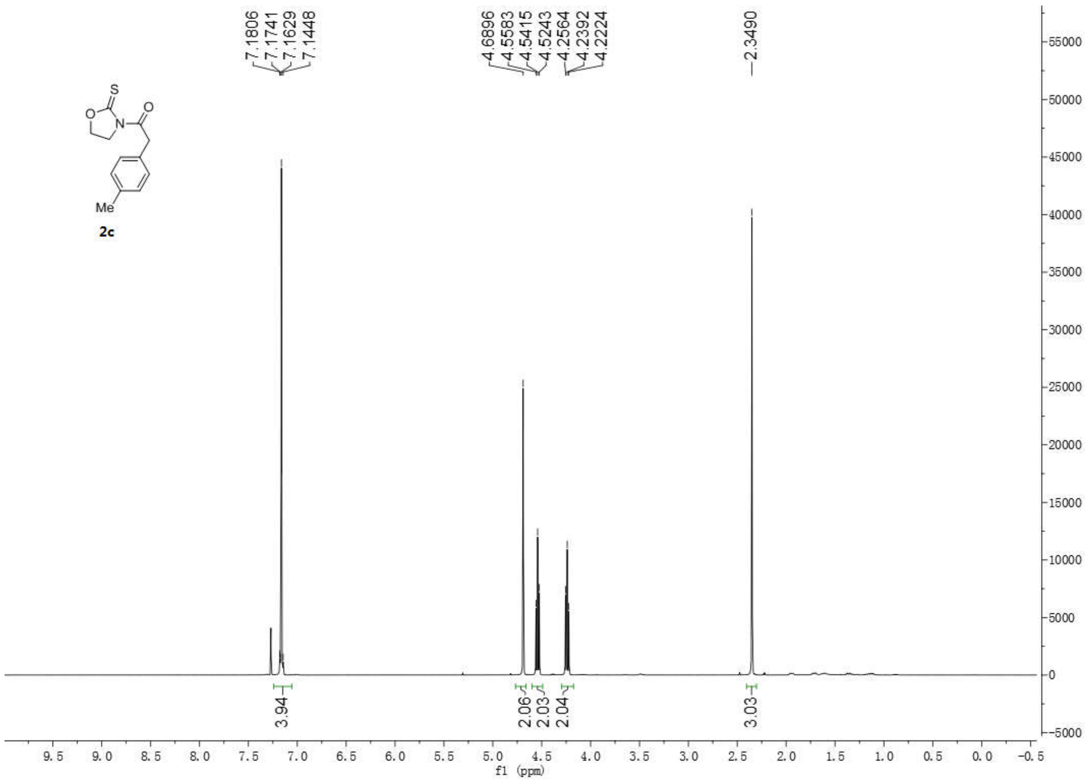

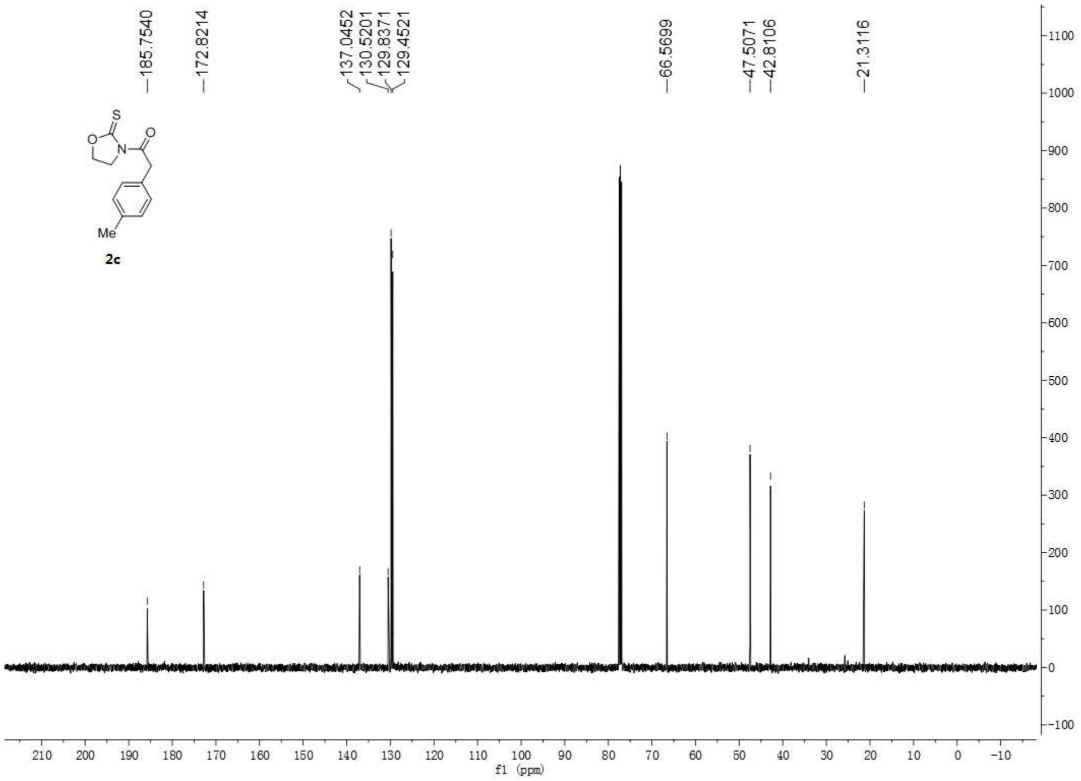

Supplementary figure 9. ^{1}H and ^{13}C NMR spectrum of compound **2c**

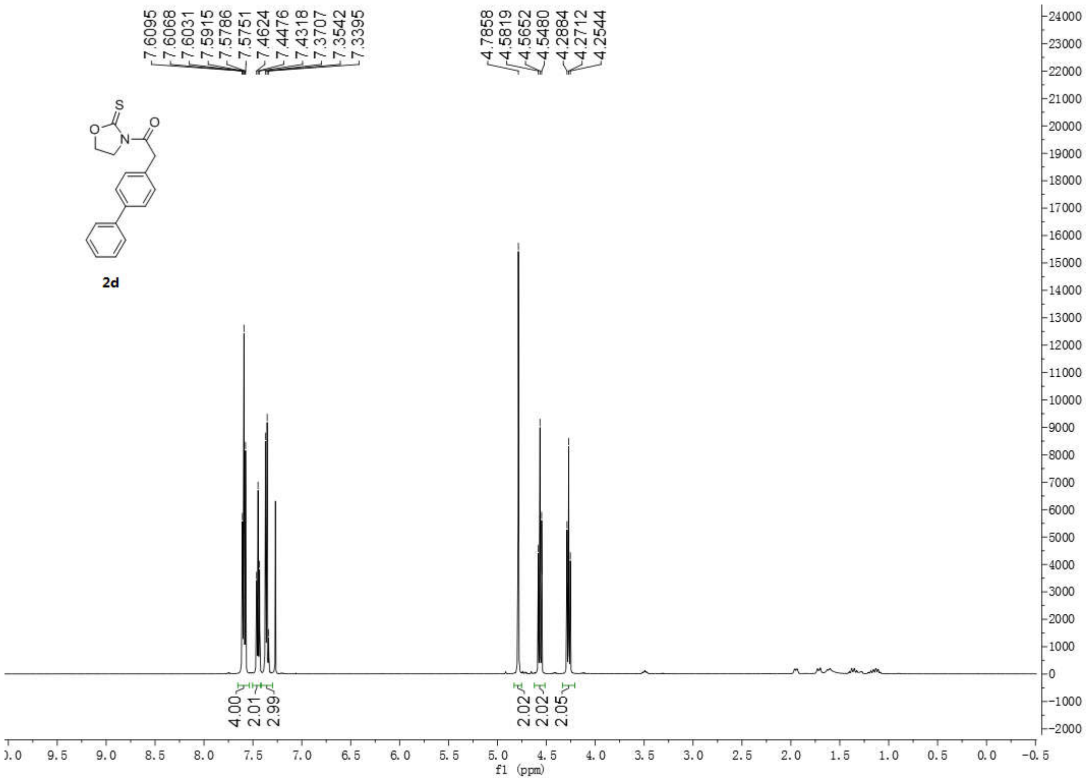

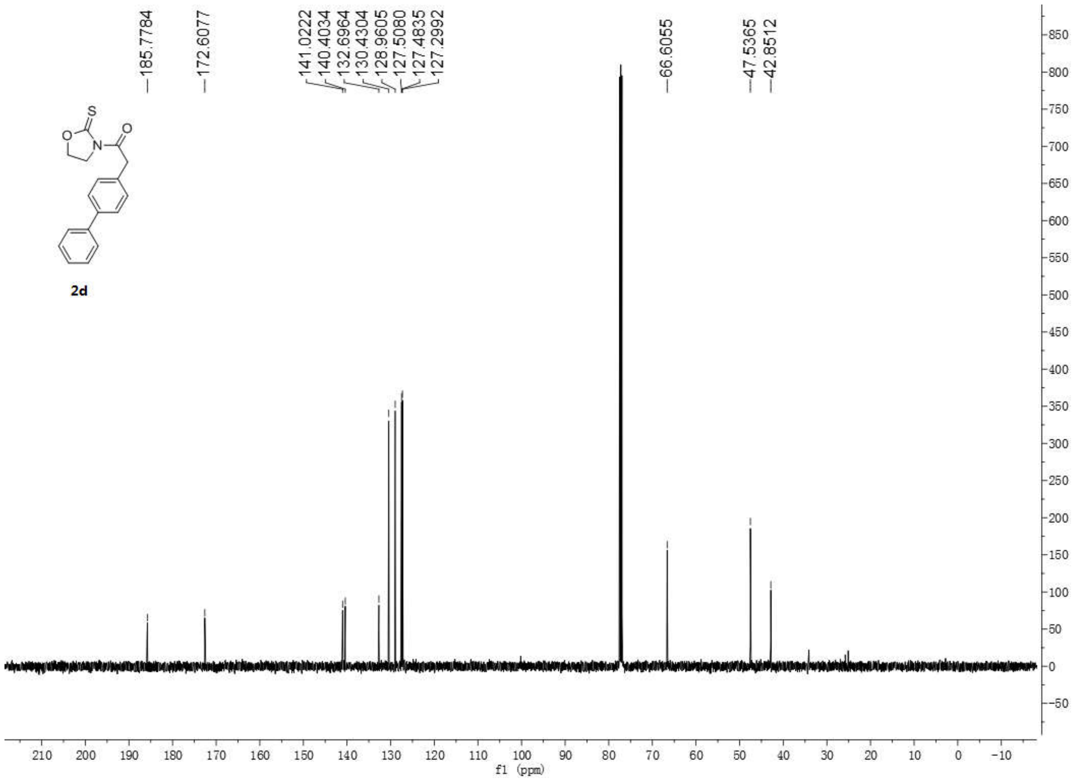

Supplementary figure 10. ^{1}H and ^{13}C NMR spectrum of compound **2d**

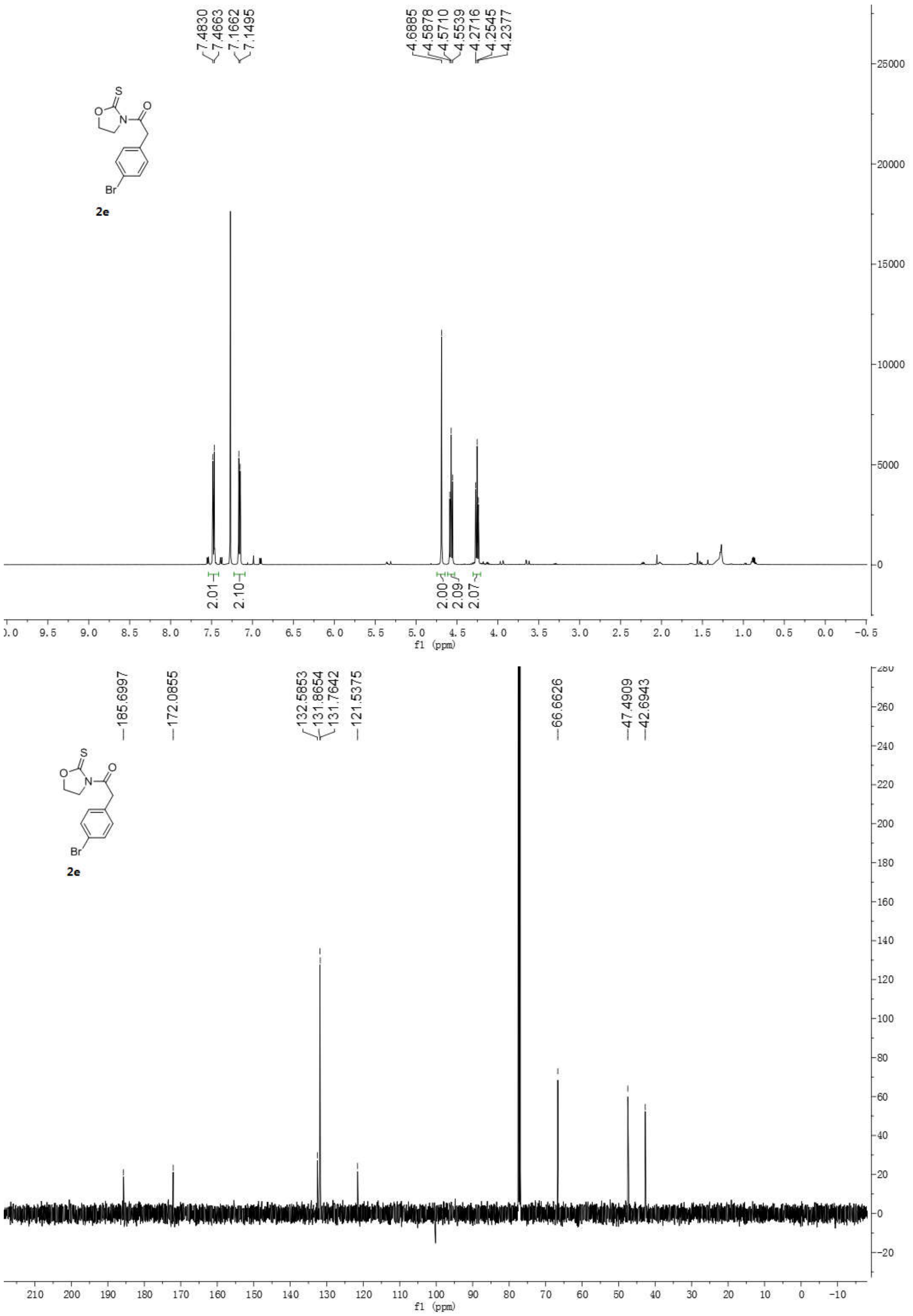

Supplementary figure 11. ^{1}H and ^{13}C NMR spectrum of compound **2e**

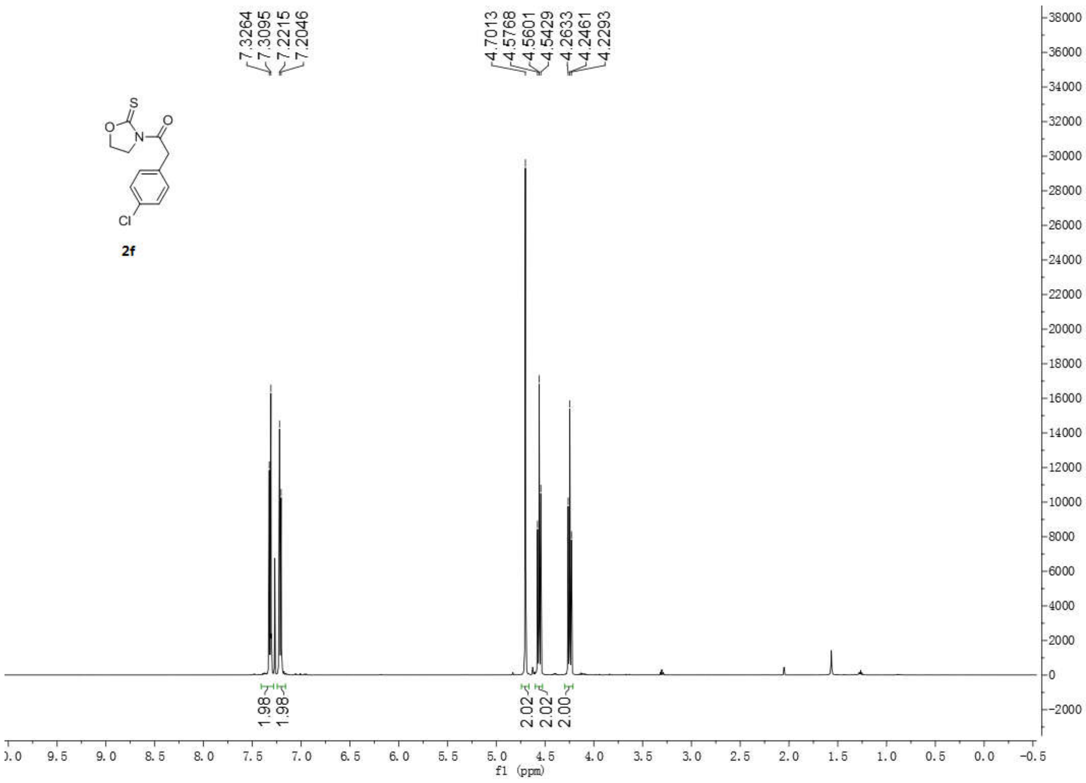

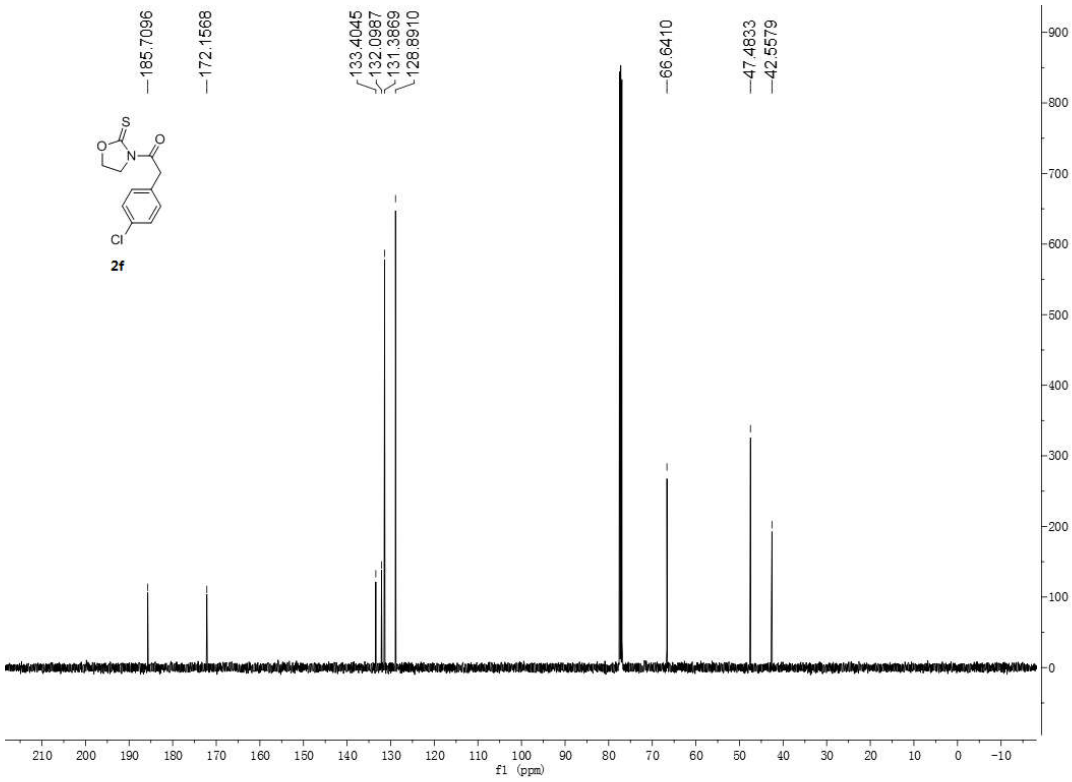

Supplementary figure 12. ^{1}H and ^{13}C NMR spectrum of compound **2f**

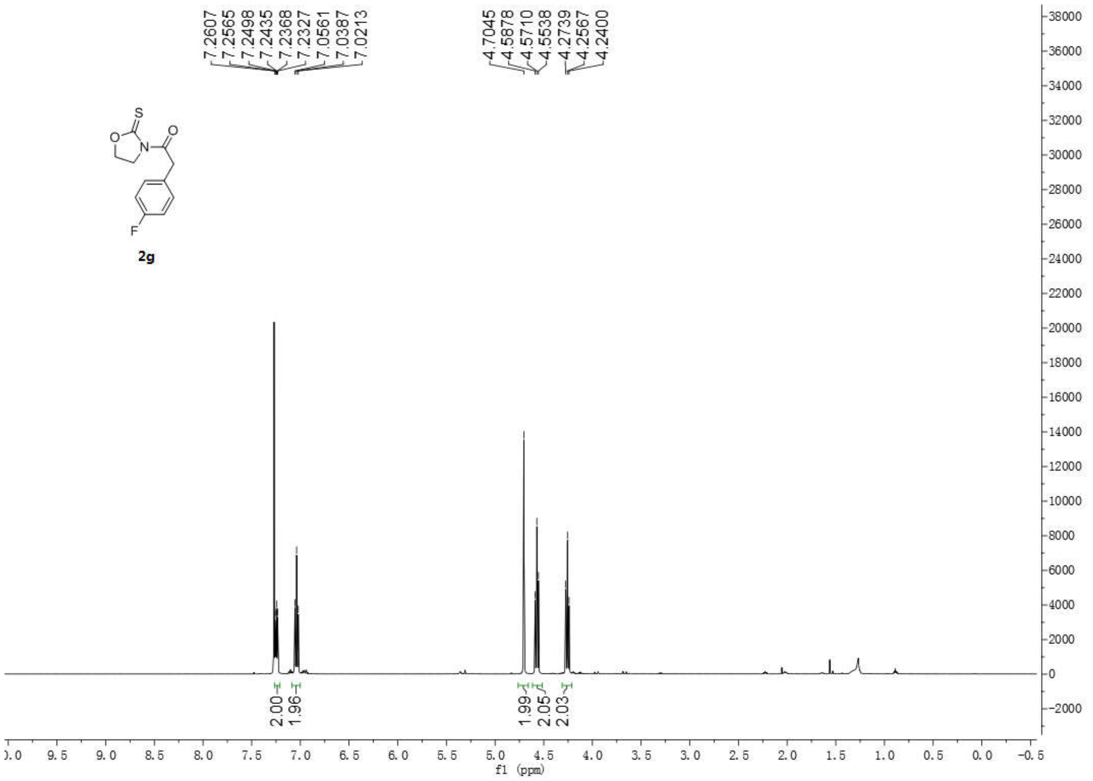

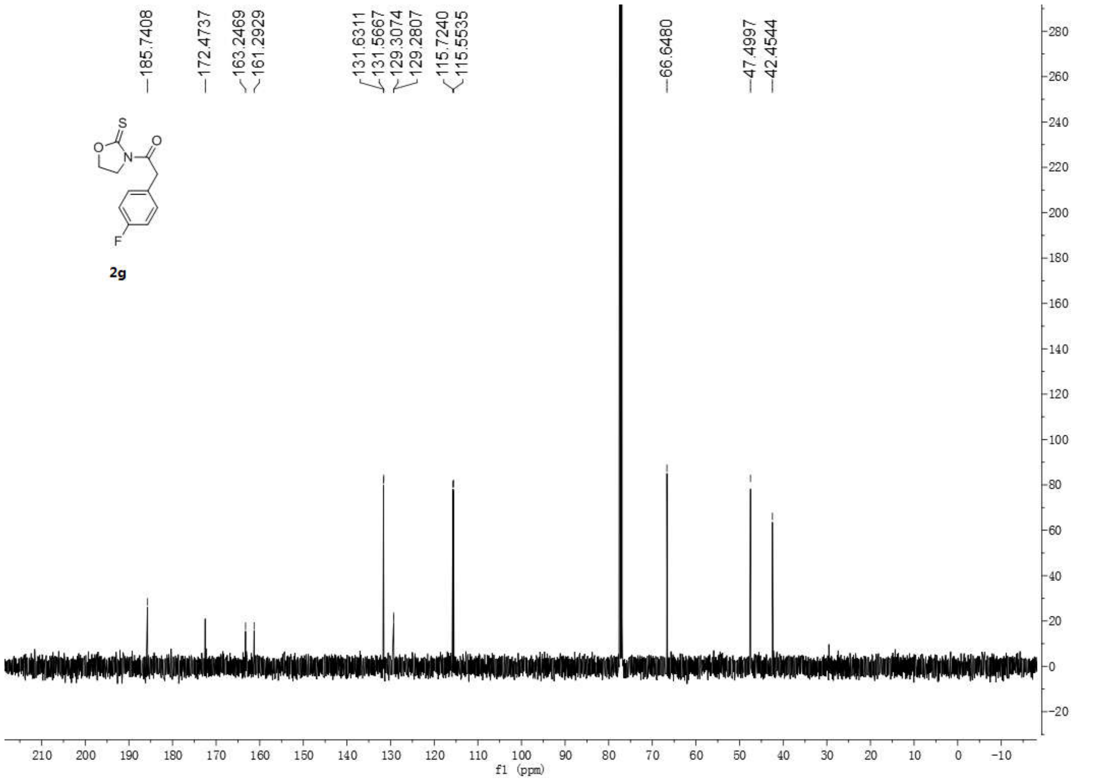

Supplementary figure 13. ^{1}H and ^{13}C NMR spectrum of compound **2g**

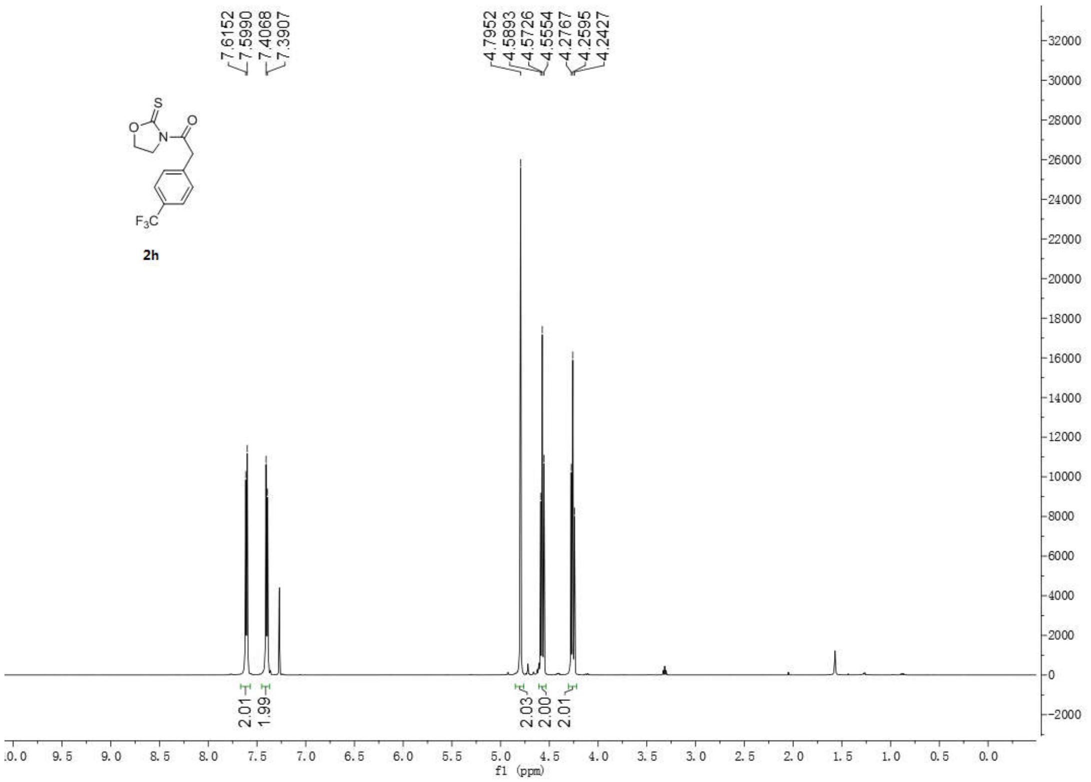

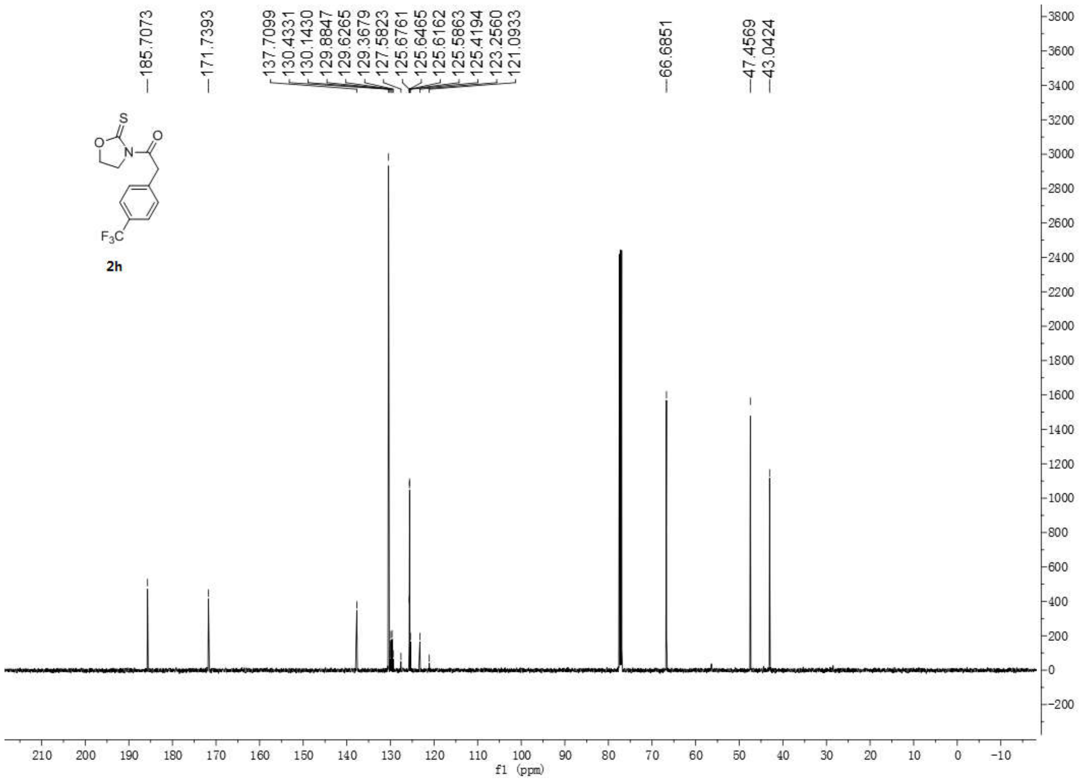

Supplementary figure 14. ^{1}H and ^{13}C NMR spectrum of compound **2h**

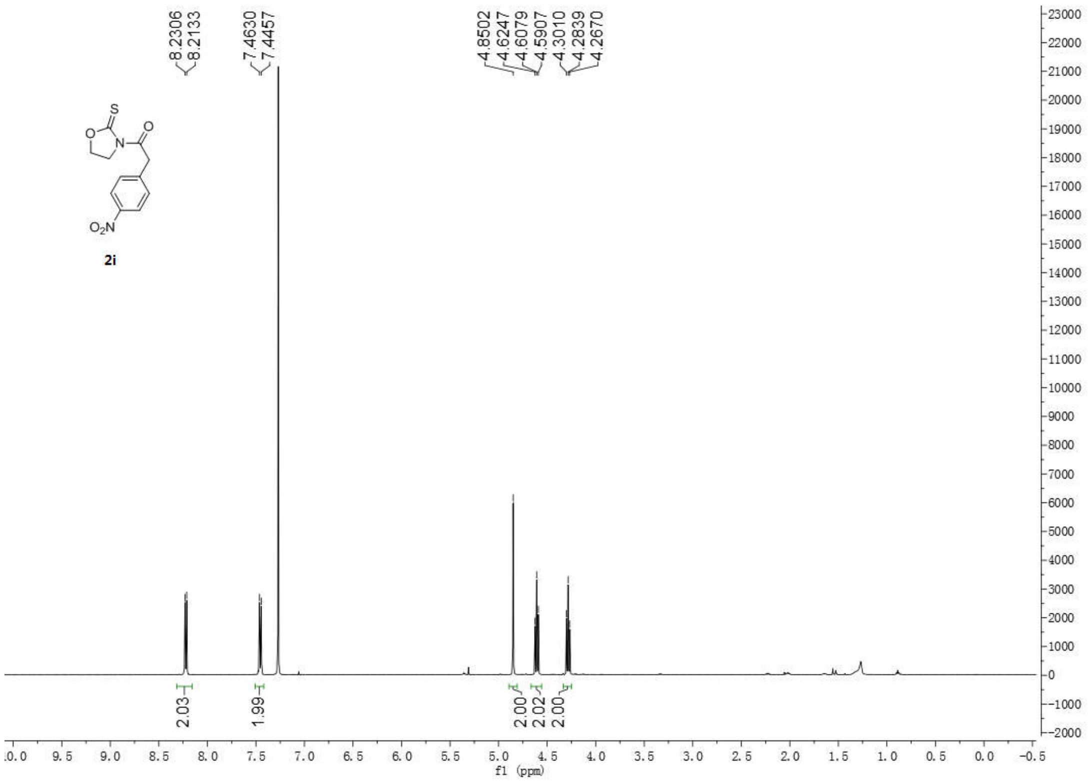

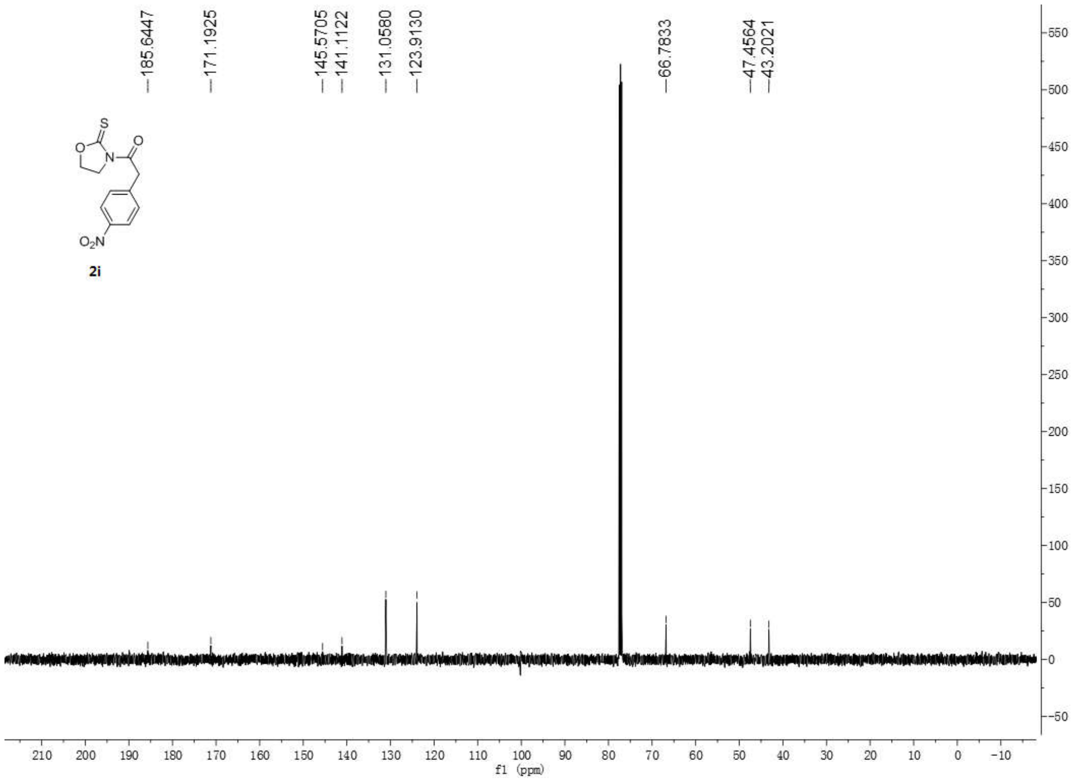

Supplementary figure 15. ^{1}H and ^{13}C NMR spectrum of compound **2i**

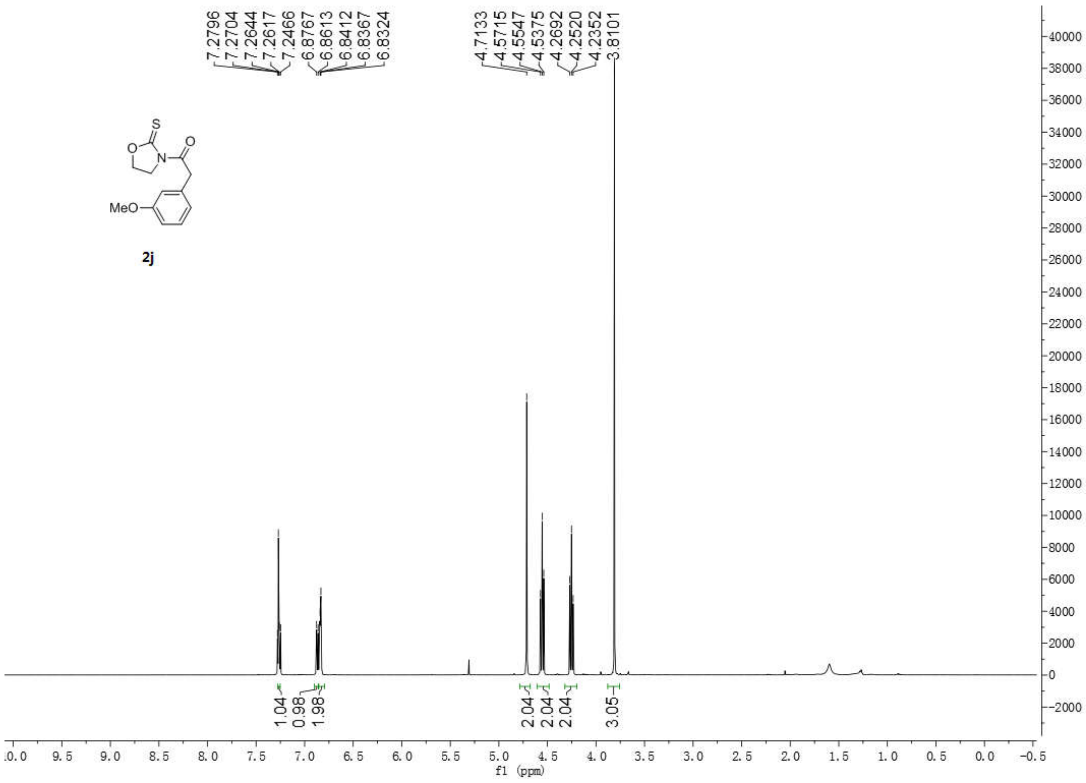

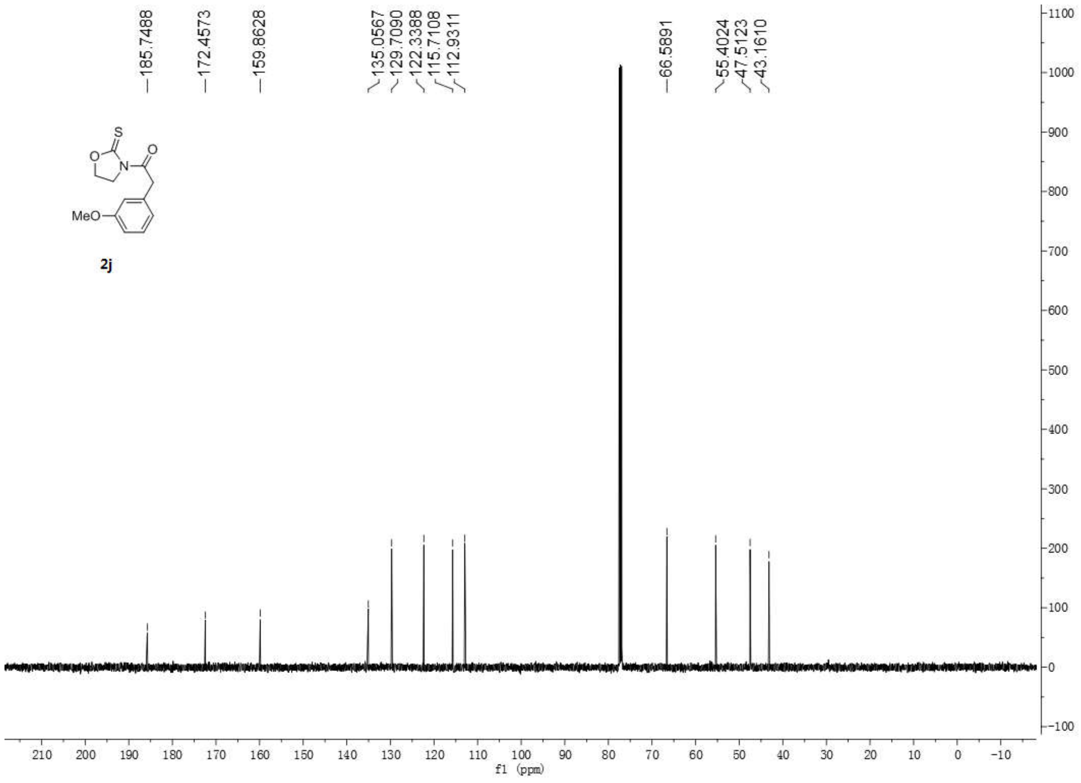

Supplementary figure 16. ^{1}H and ^{13}C NMR spectrum of compound **2j**

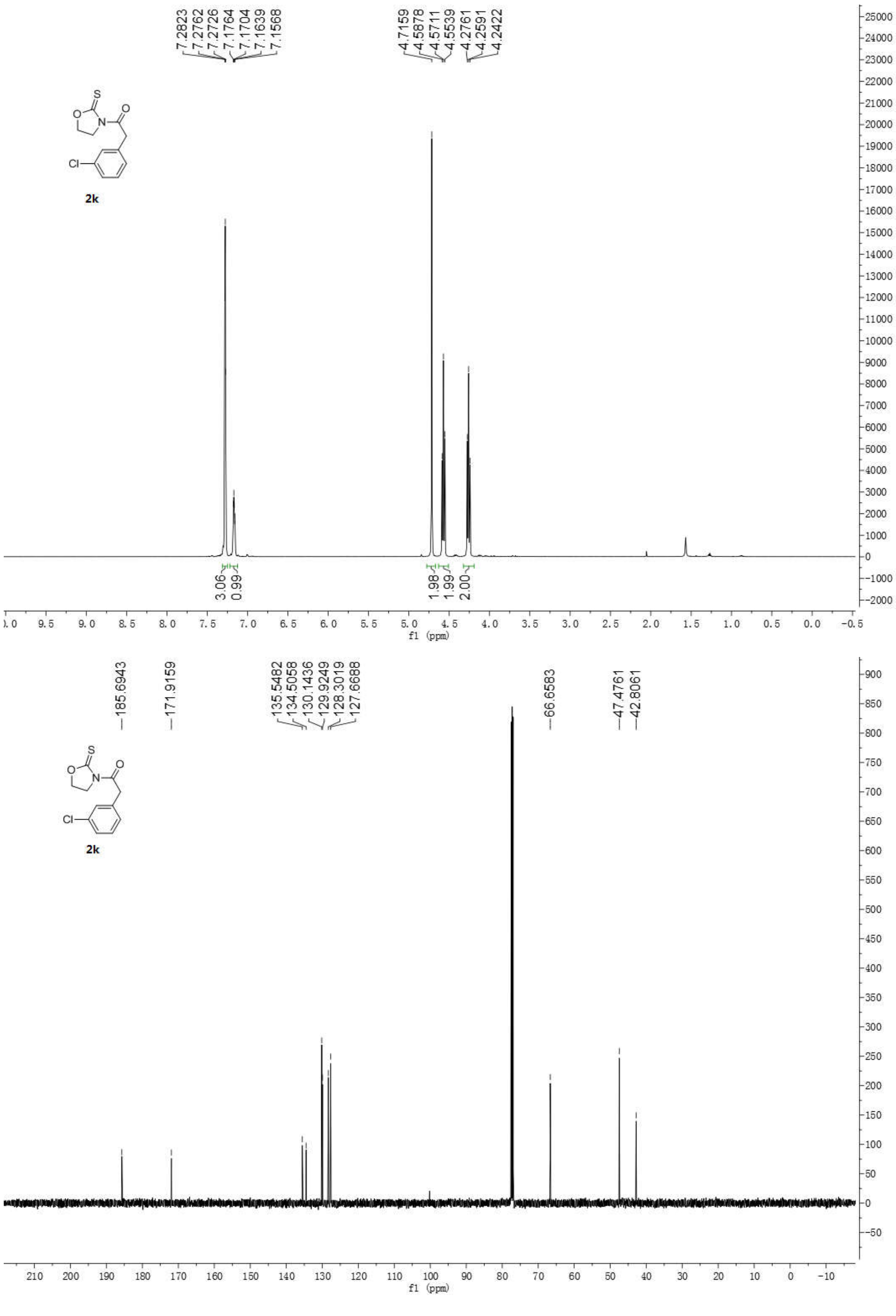

Supplementary figure 17. ^{1}H and ^{13}C NMR spectrum of compound **2k**

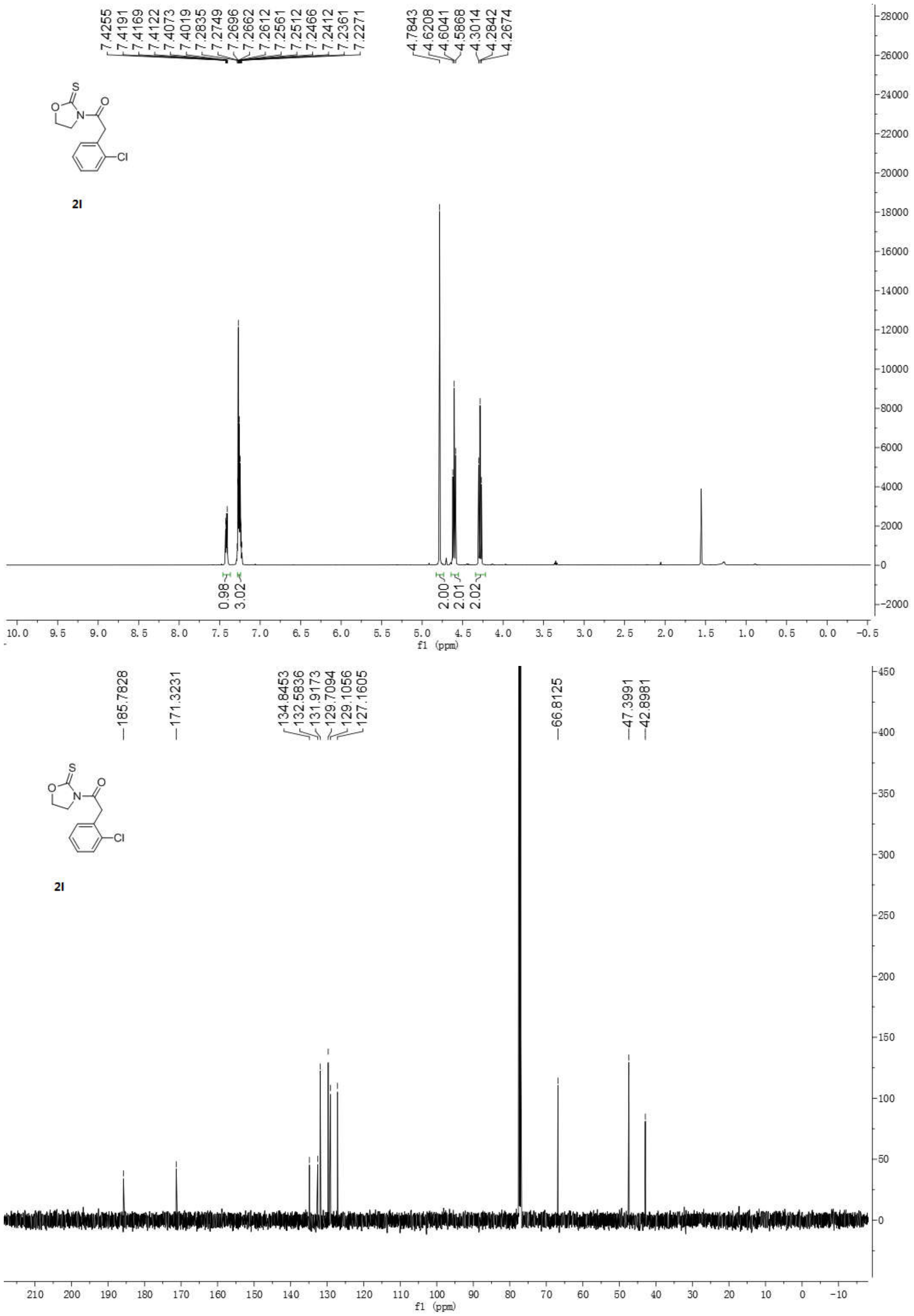

Supplementary figure 18. ^{1}H and ^{13}C NMR spectrum of compound **2l**

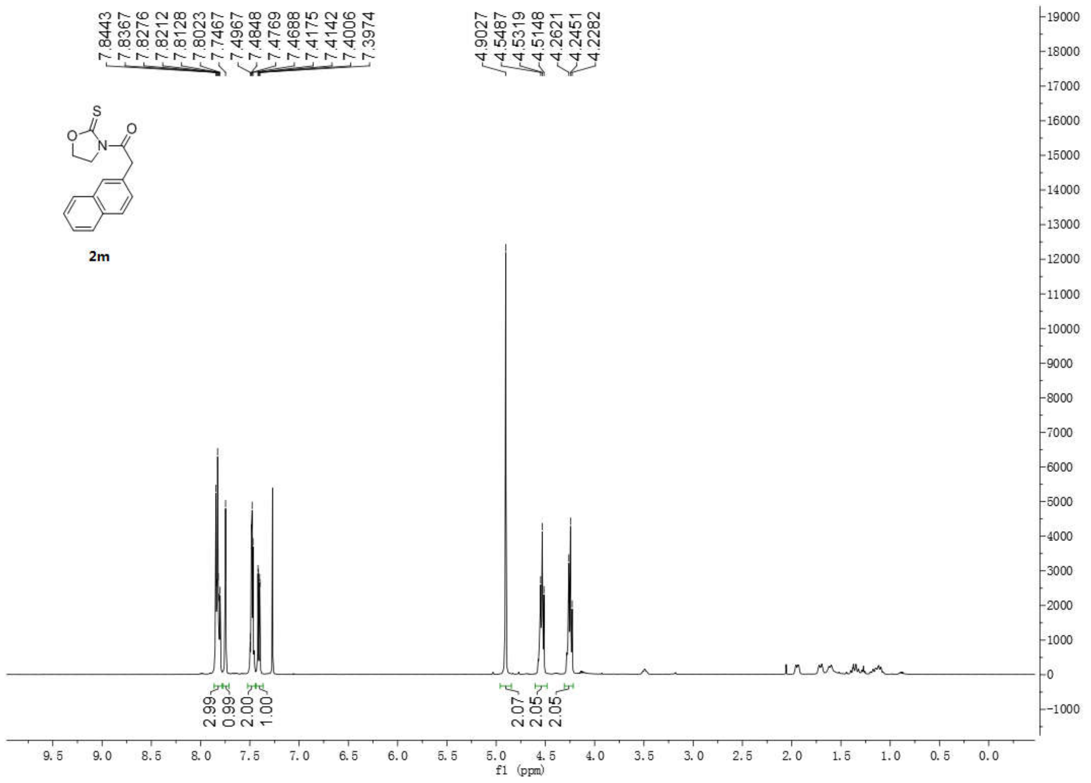

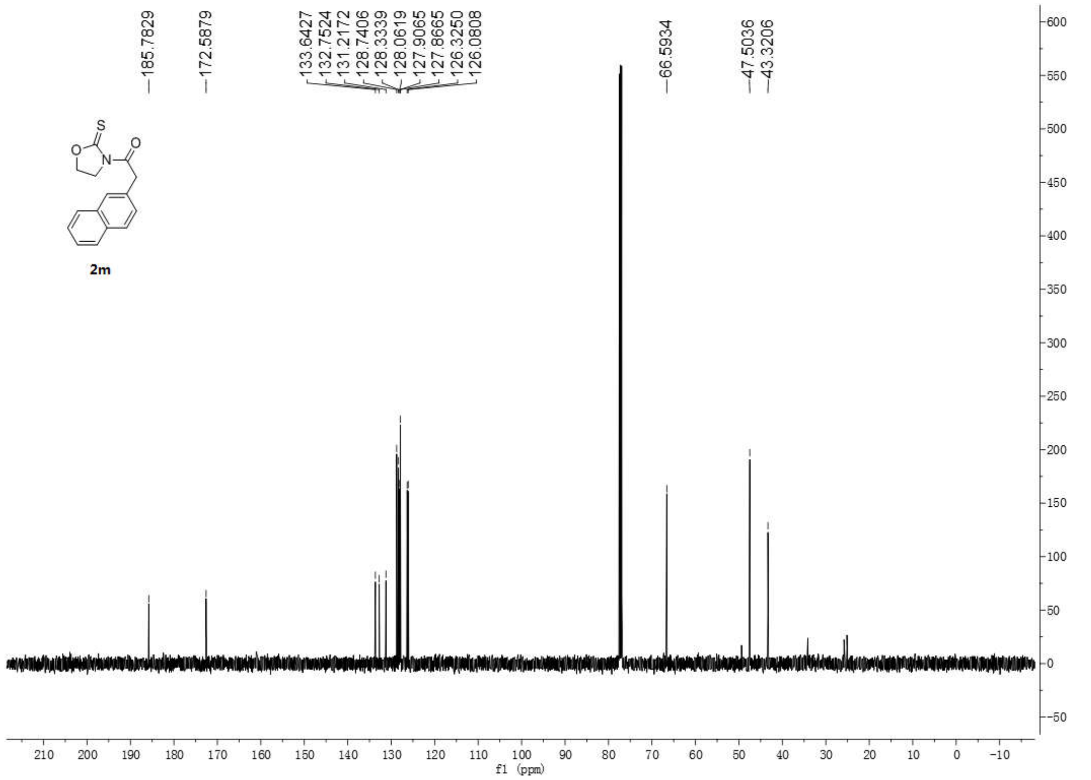

Supplementary figure 19. ^{1}H and ^{13}C NMR spectrum of compound **2m**

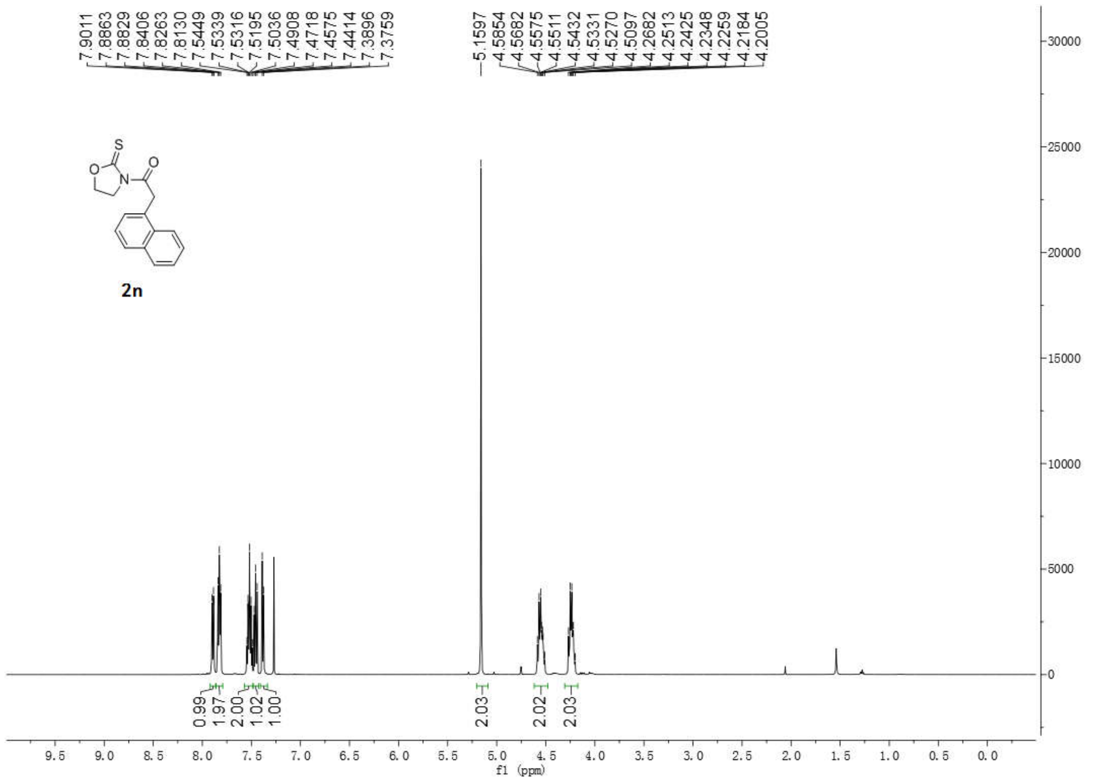

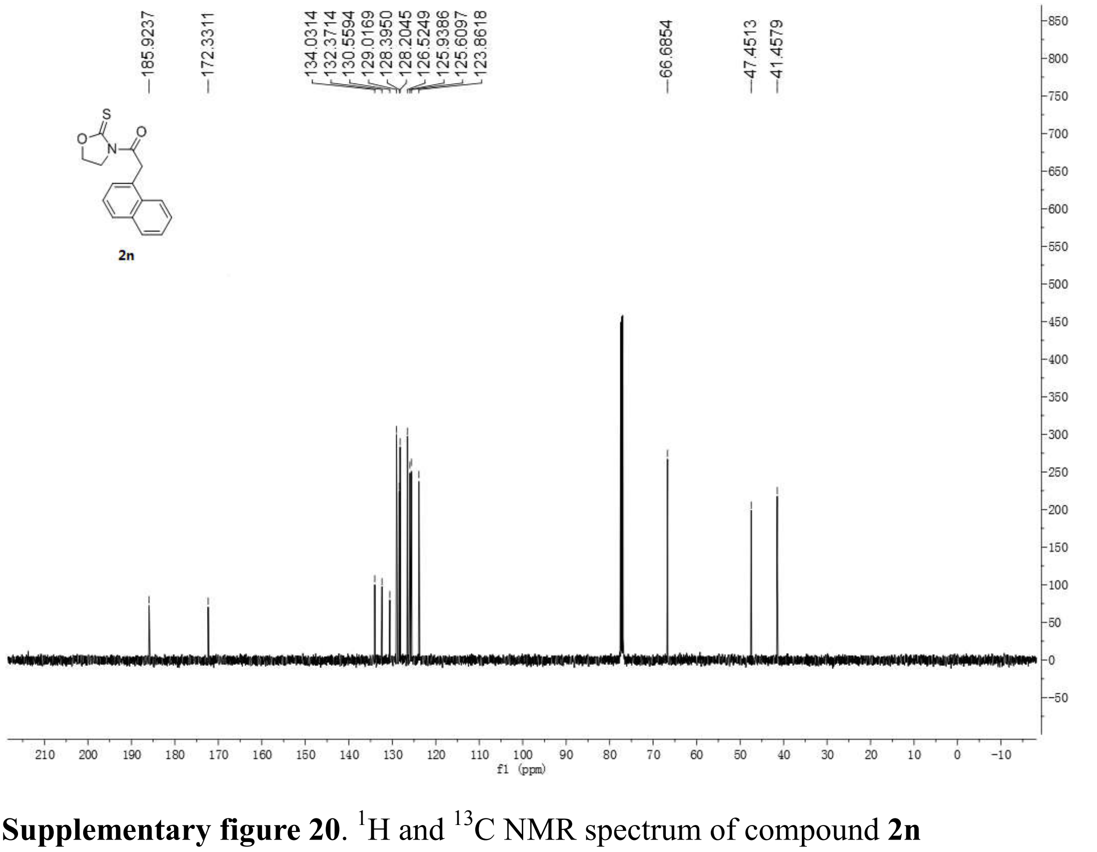

Supplementary figure 20. ^{1}H and ^{13}C NMR spectrum of compound **2n**

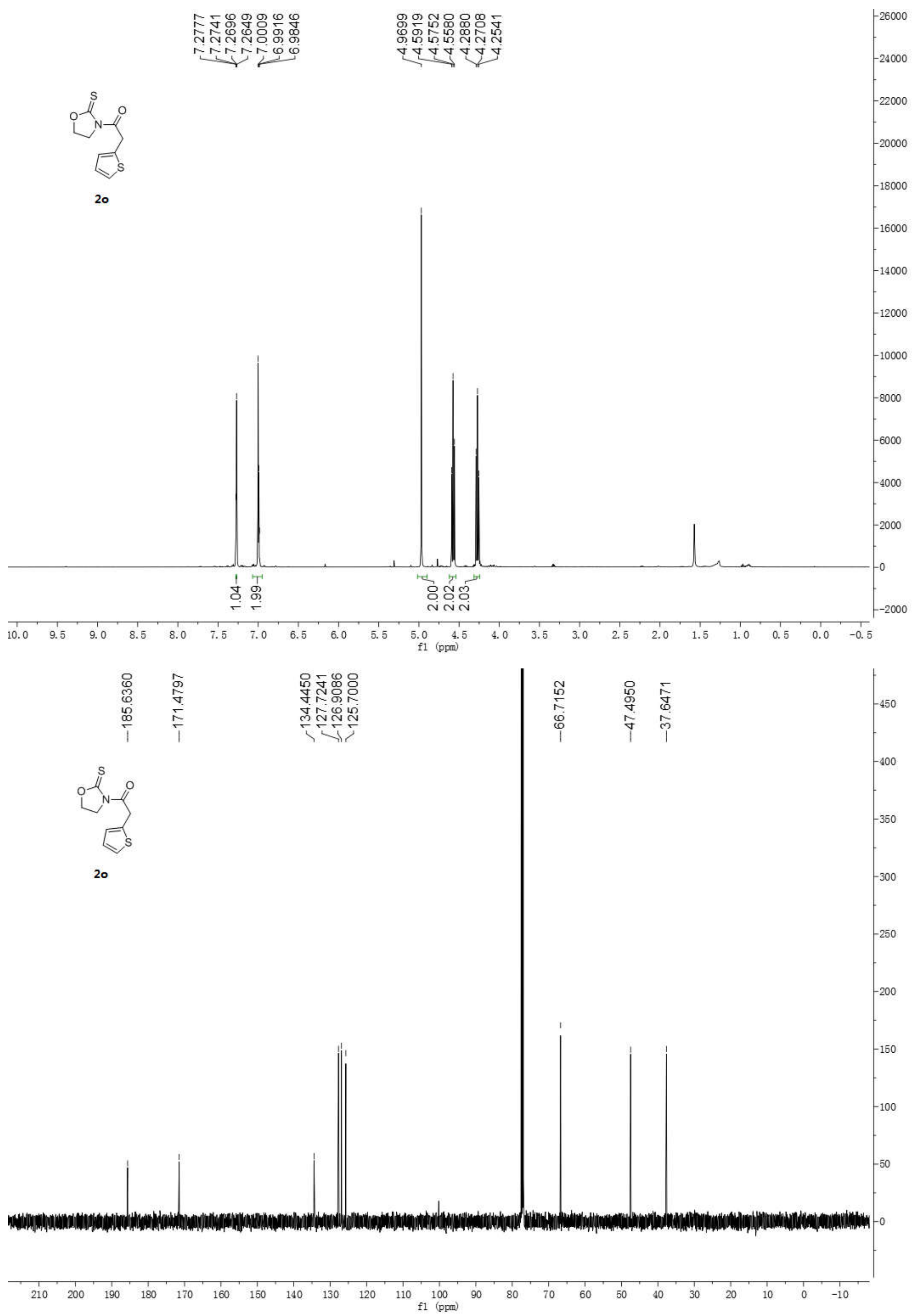

Supplementary figure 21. ^{1}H and ^{13}C NMR spectrum of compound **2o**

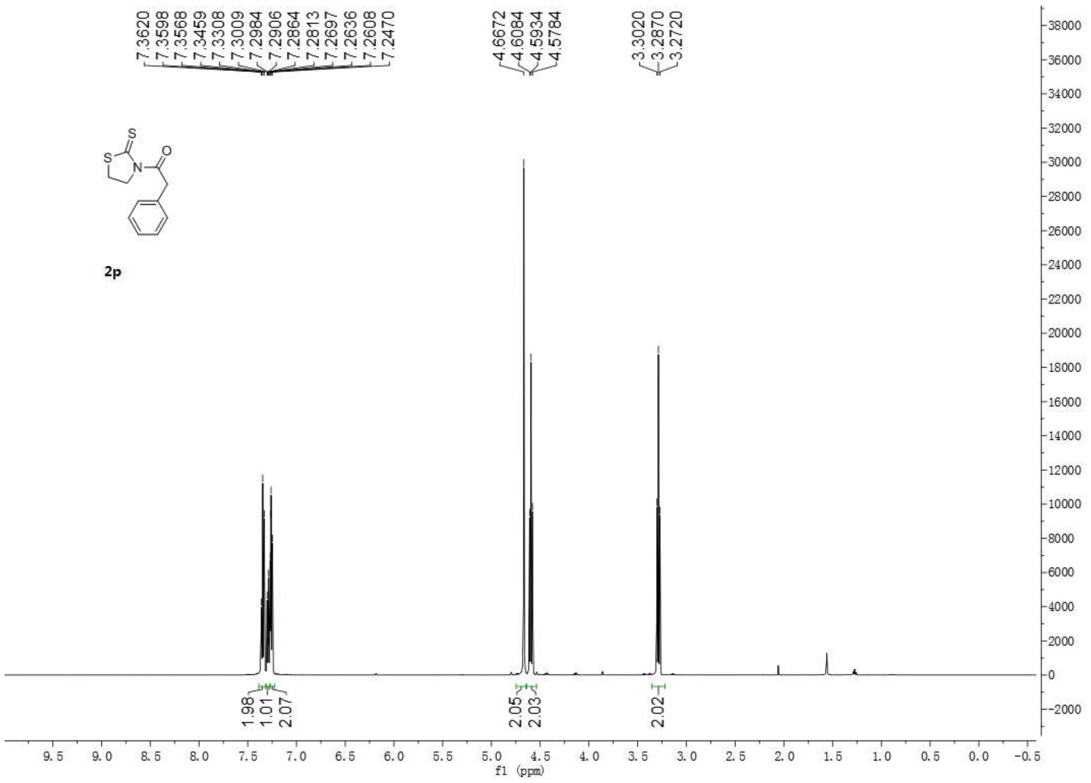

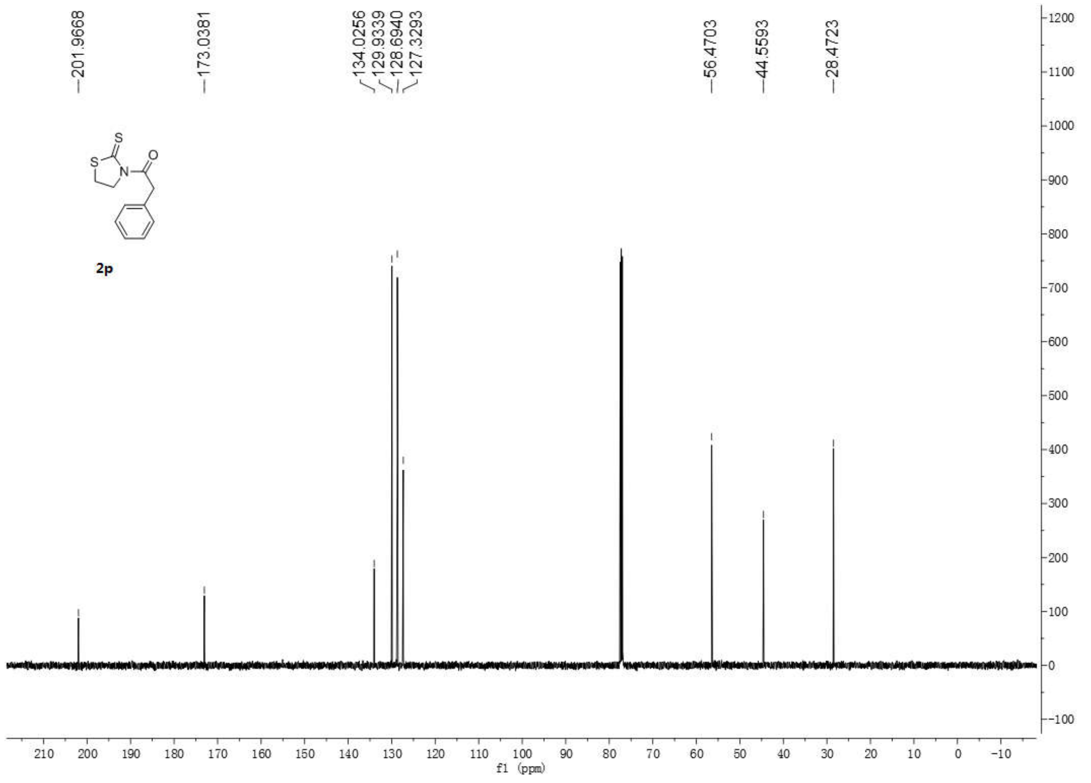

Supplementary figure 22. ^{1}H and ^{13}C NMR spectrum of compound **2p**

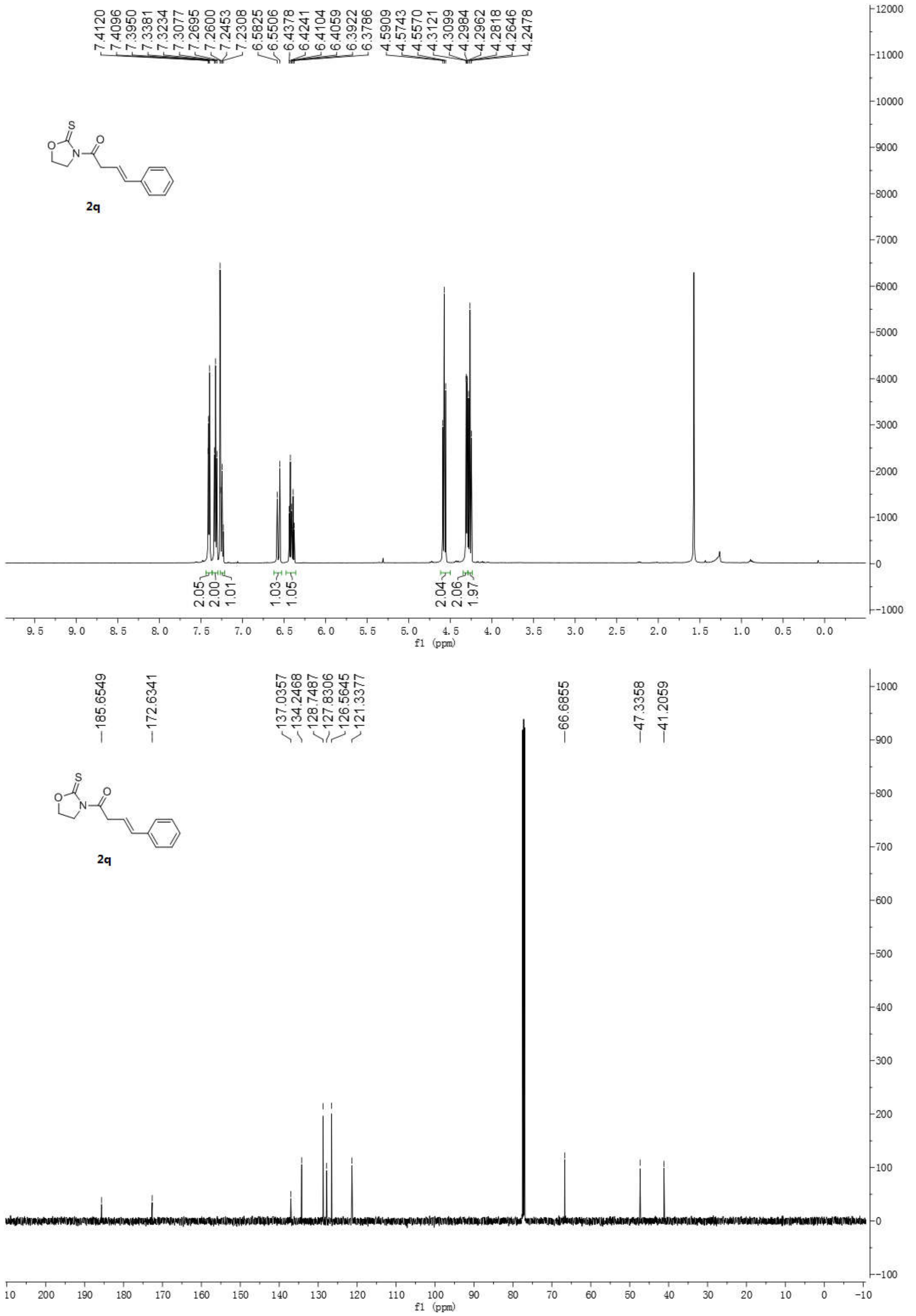

Supplementary figure 23. ^{1}H and ^{13}C NMR spectrum of compound **2q**

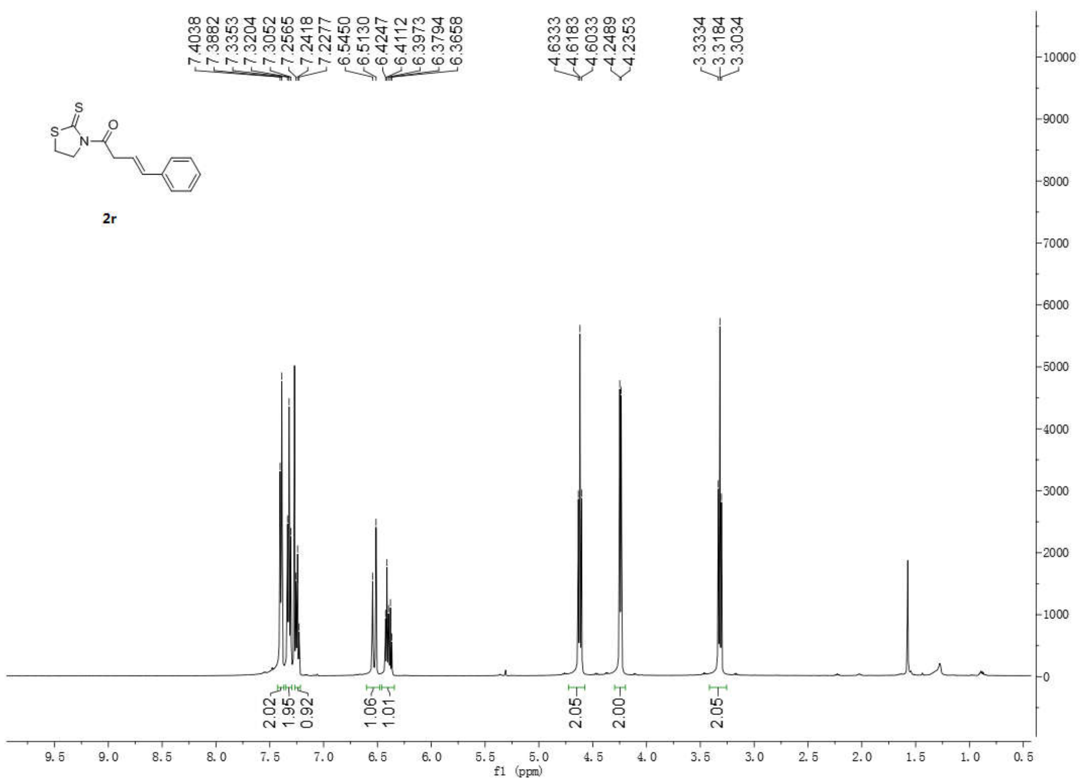

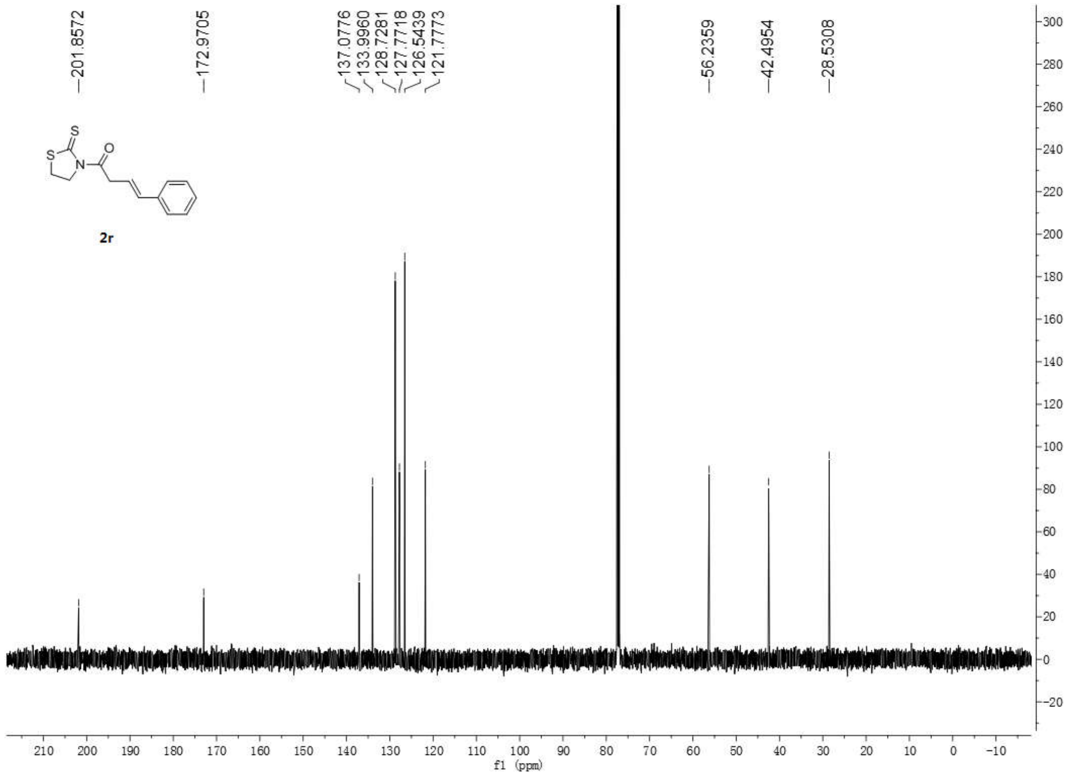

Supplementary figure 24. ^{1}H and ^{13}C NMR spectrum of compound **2r**

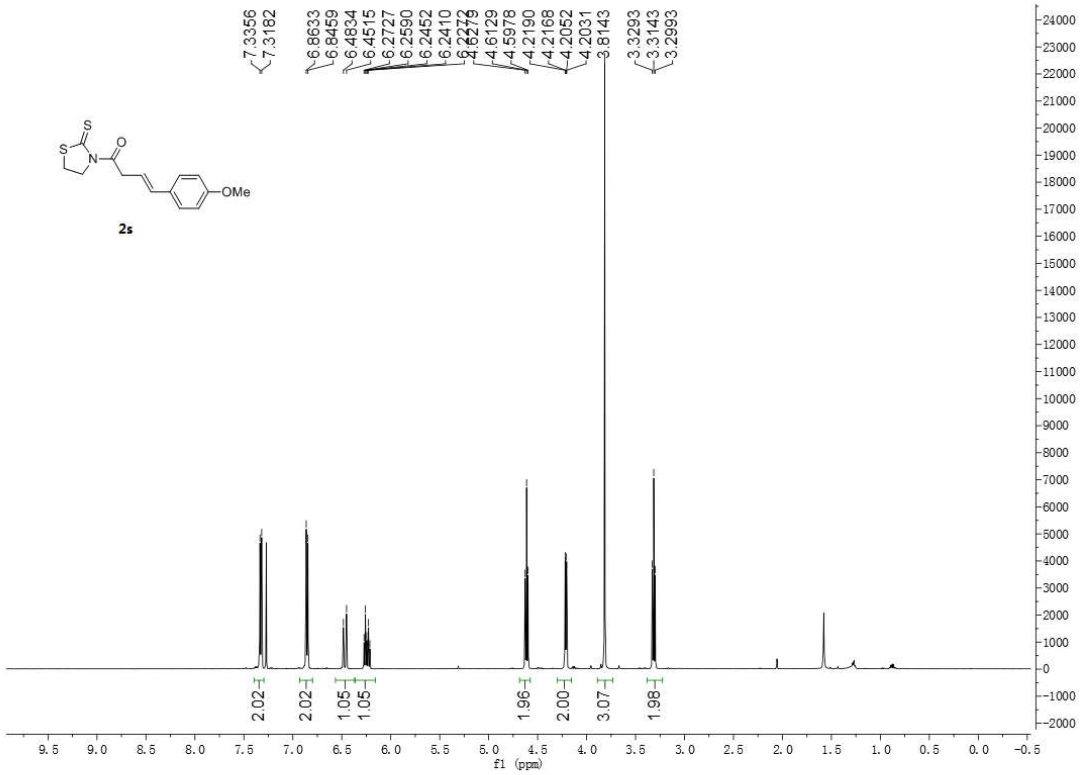

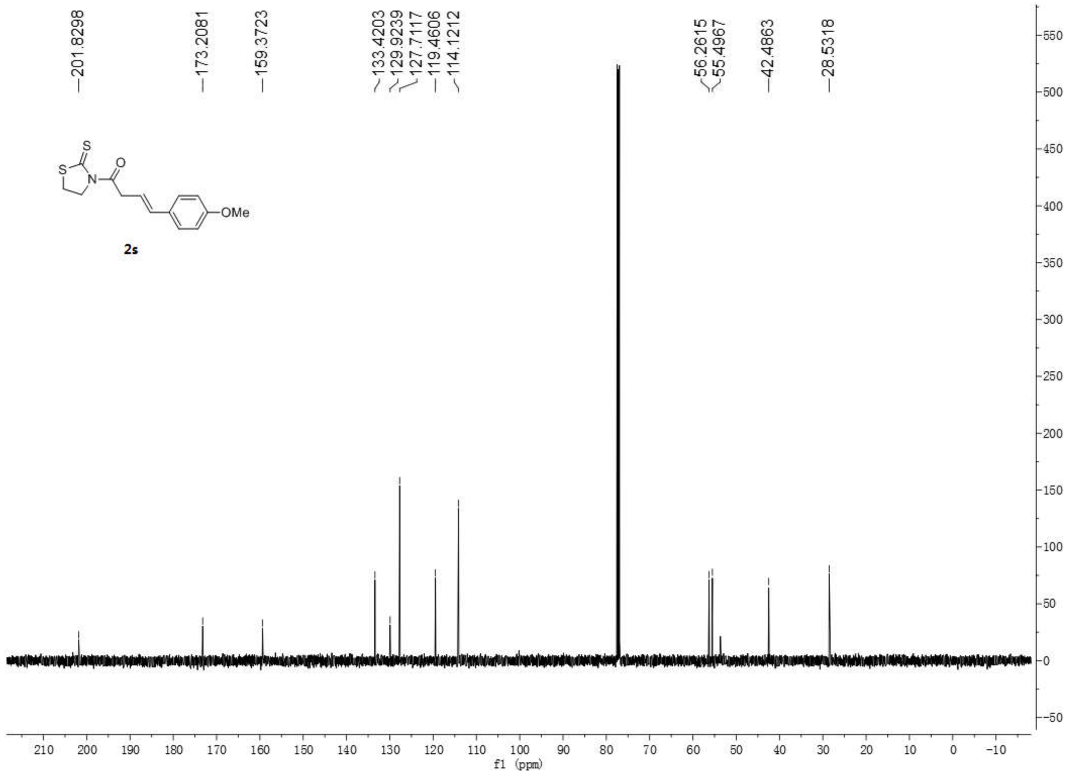

Supplementary figure 25. ^{1}H and ^{13}C NMR spectrum of compound **2s**

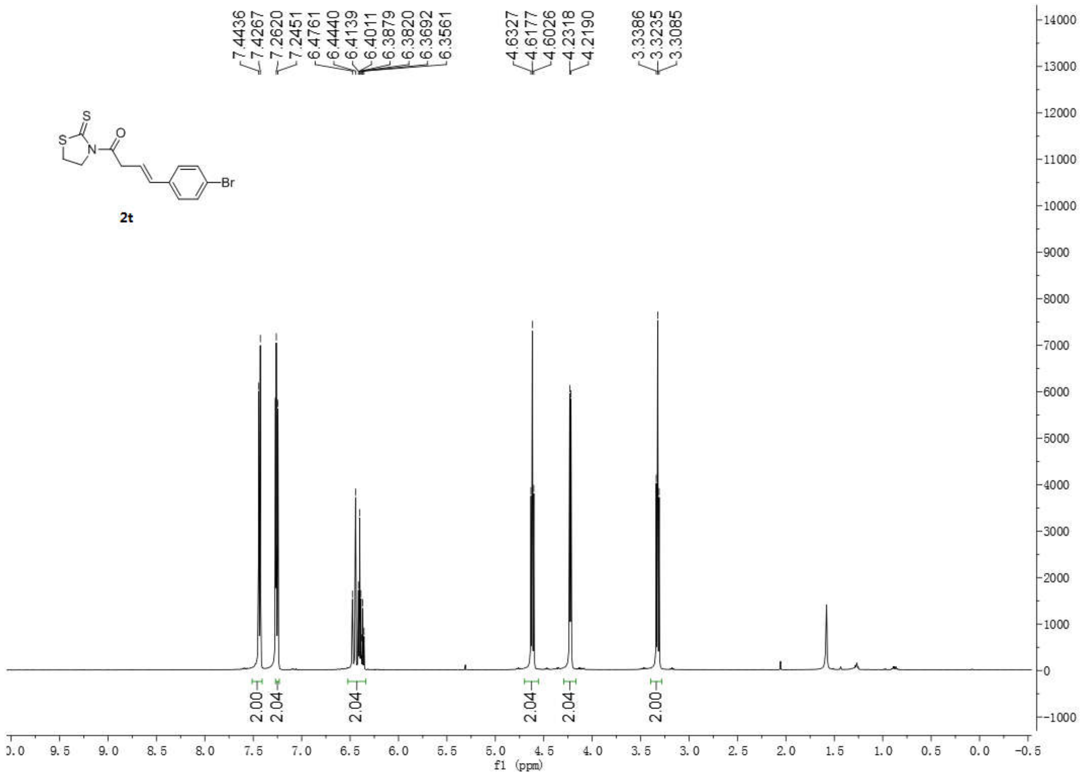

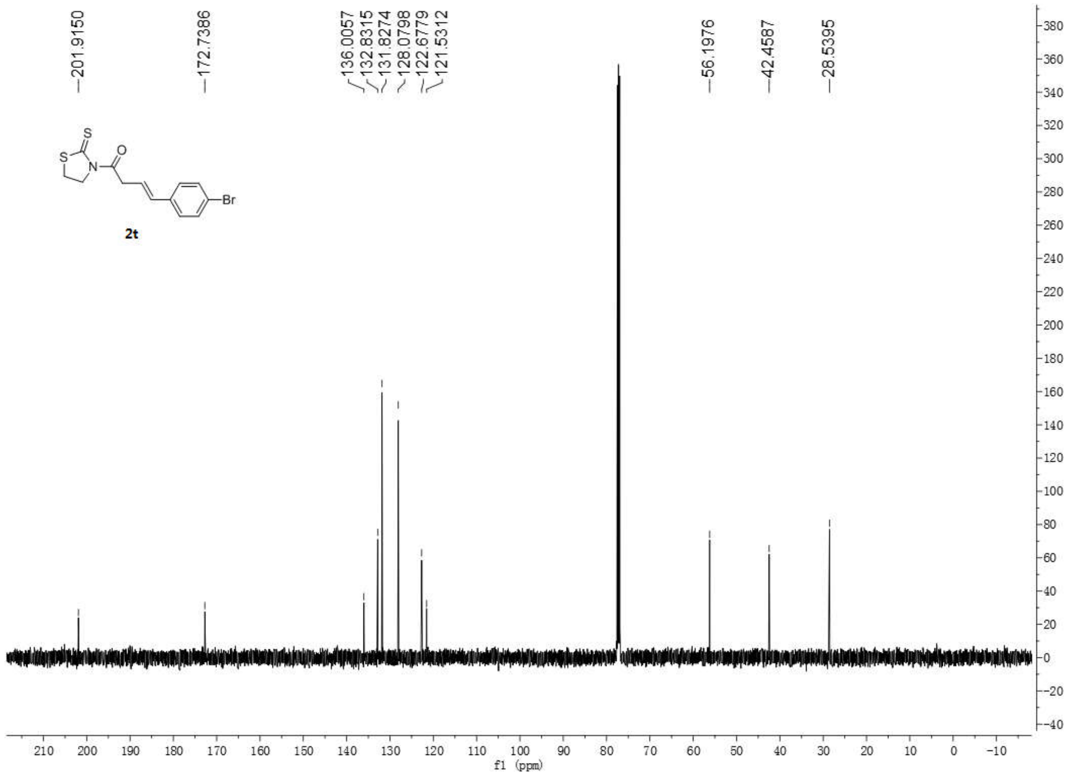

Supplementary figure 26. ^{1}H and ^{13}C NMR spectrum of compound **2t**

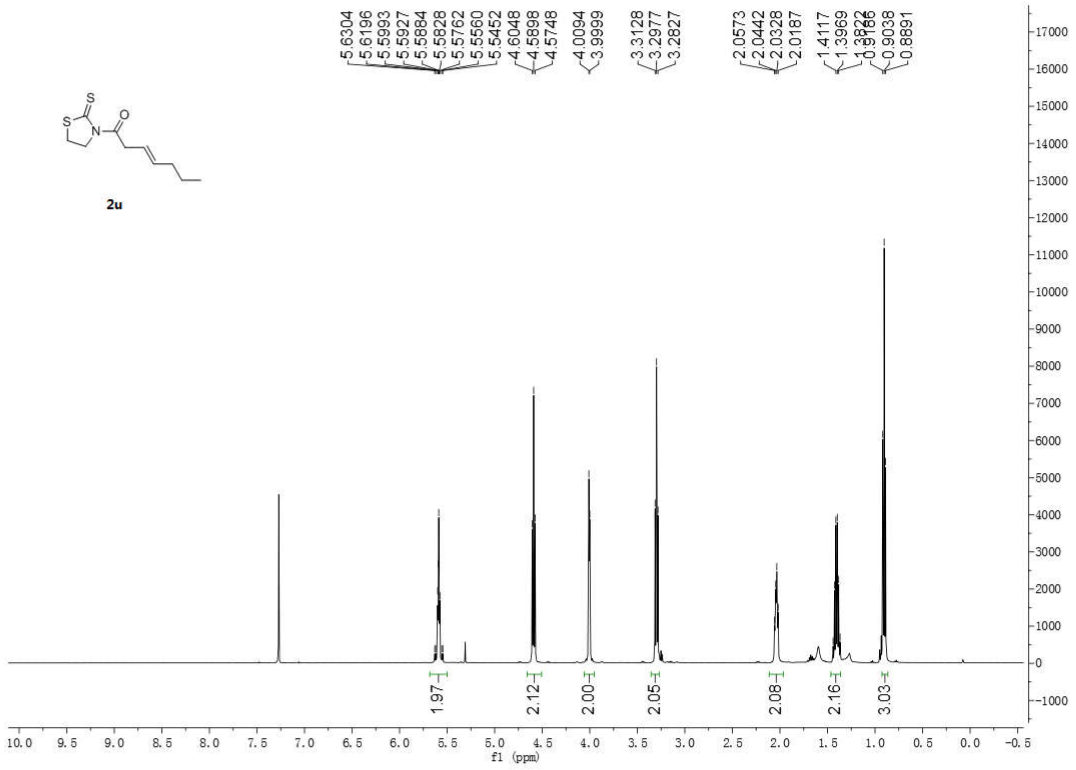

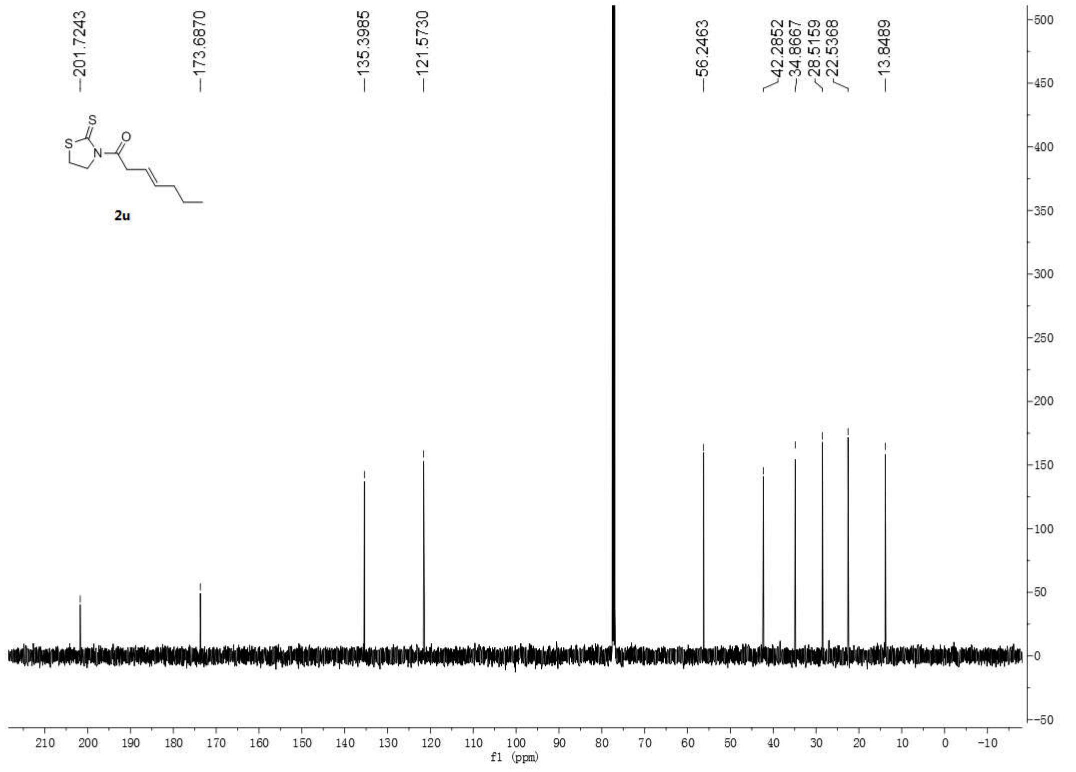

Supplementary figure 27. ^{1}H and ^{13}C NMR spectrum of compound **2u**

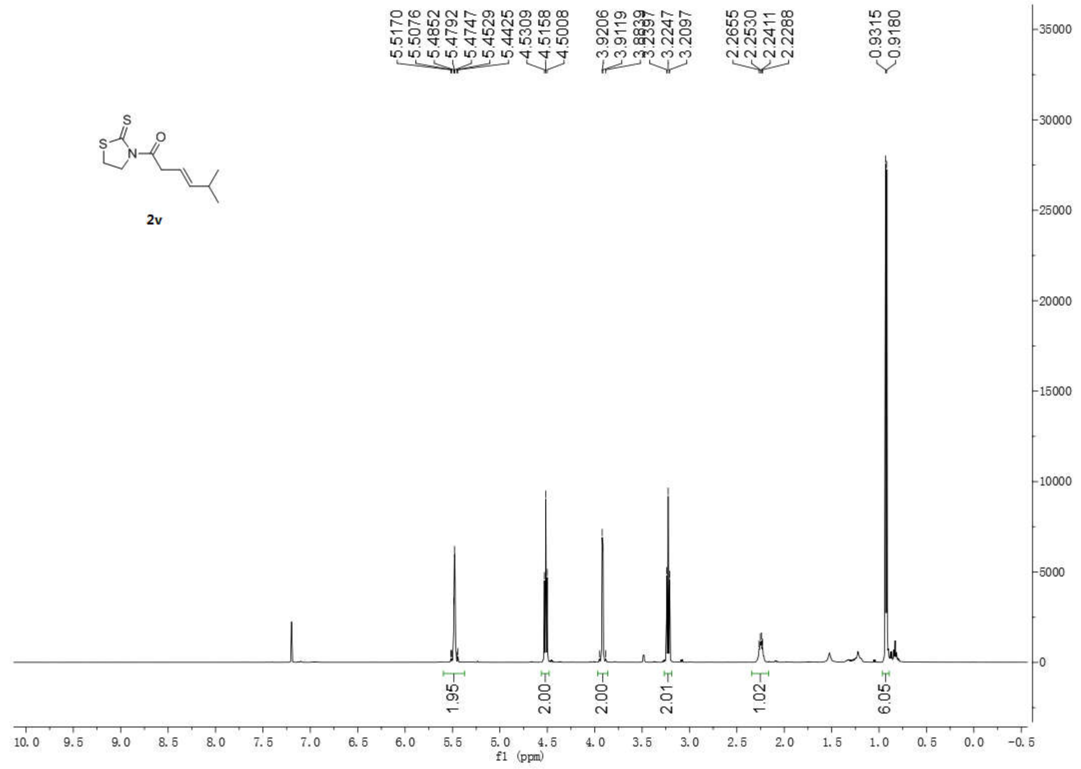

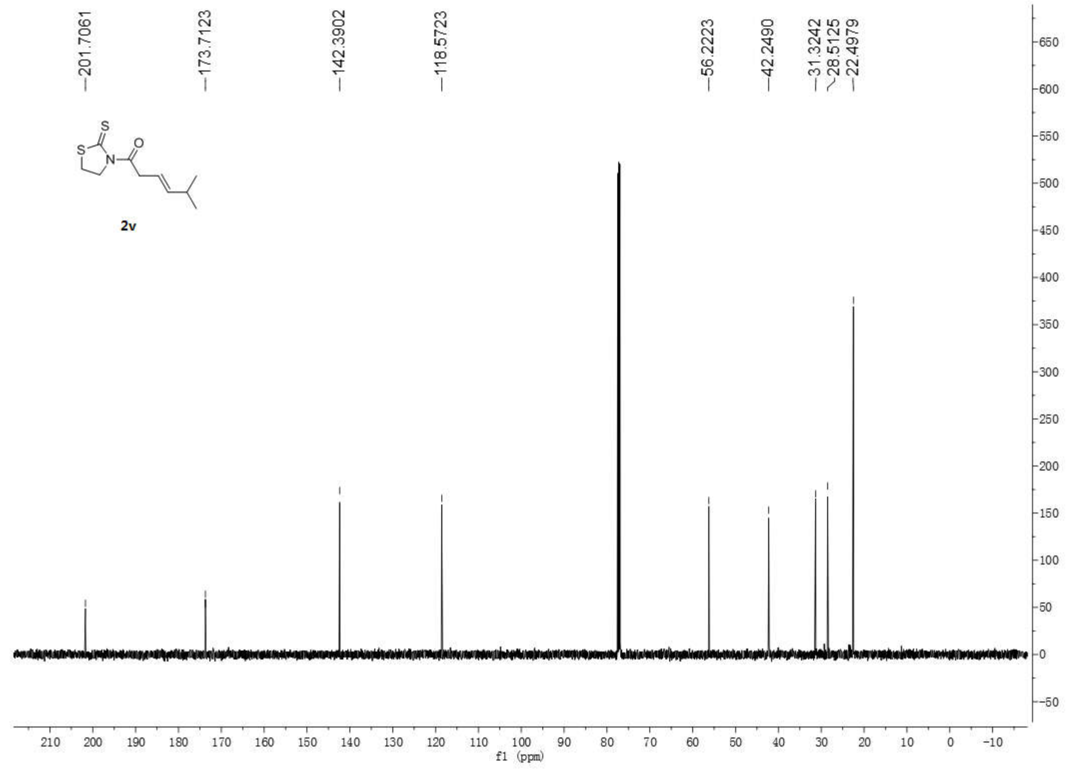

Supplementary figure 28. ^{1}H and ^{13}C NMR spectrum of compound **2v**

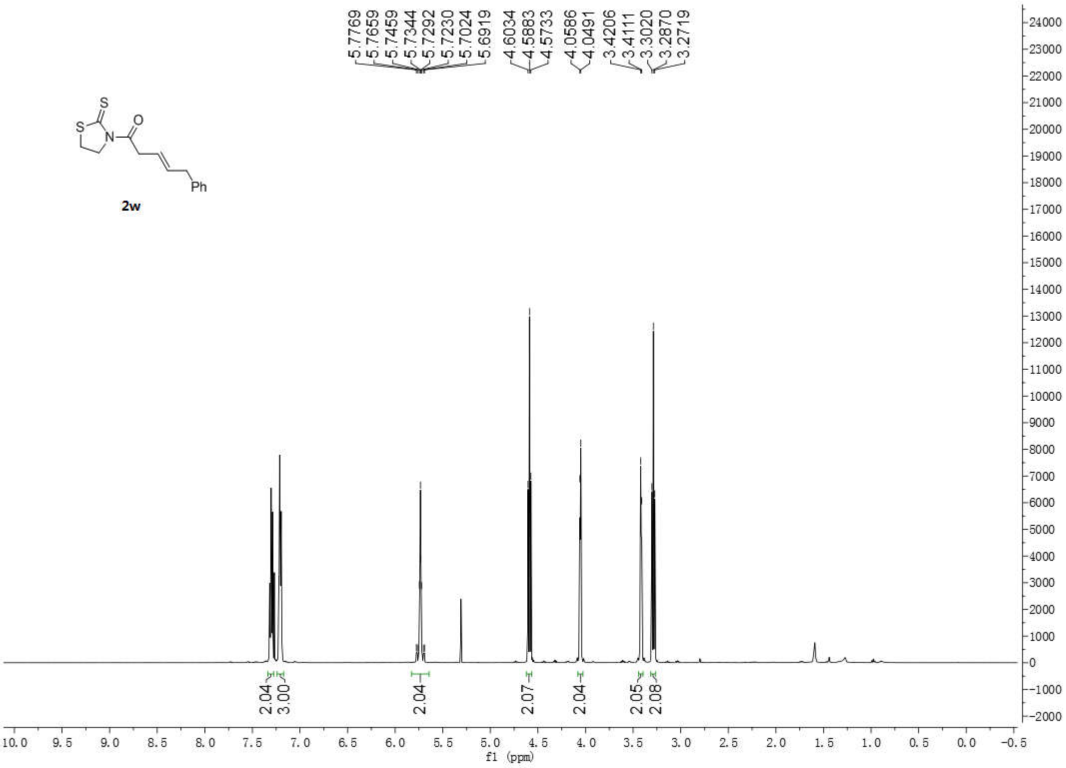

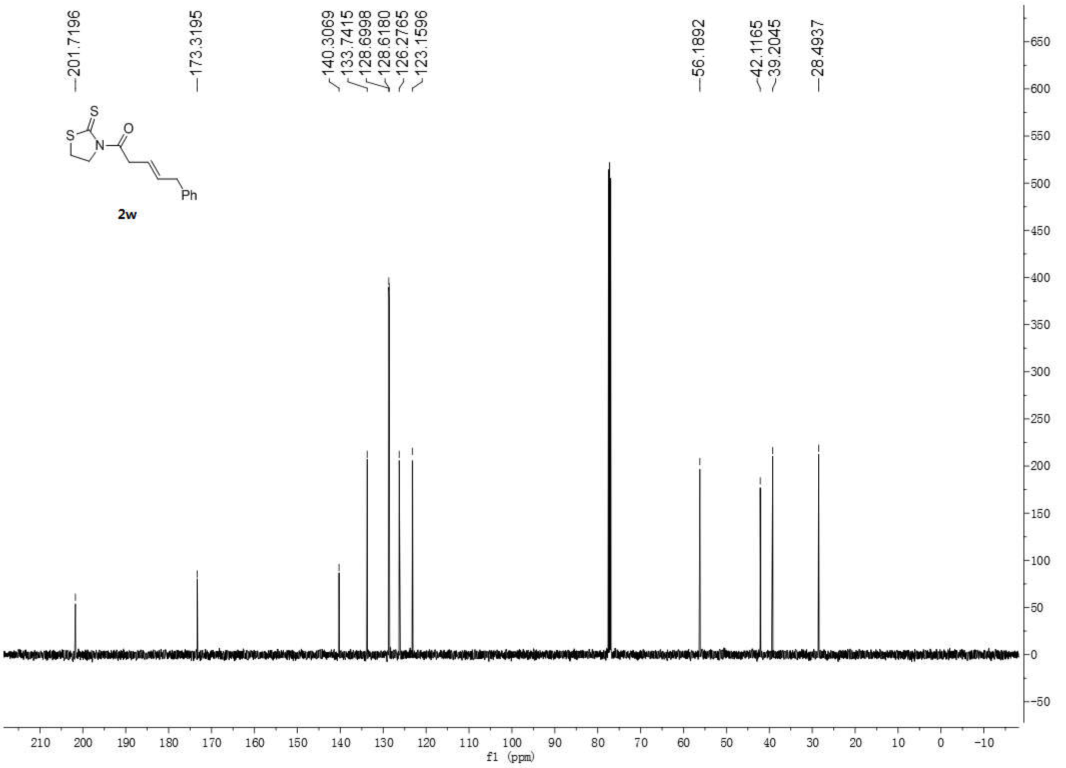

Supplementary figure 29. ^{1}H and ^{13}C NMR spectrum of compound **2w**

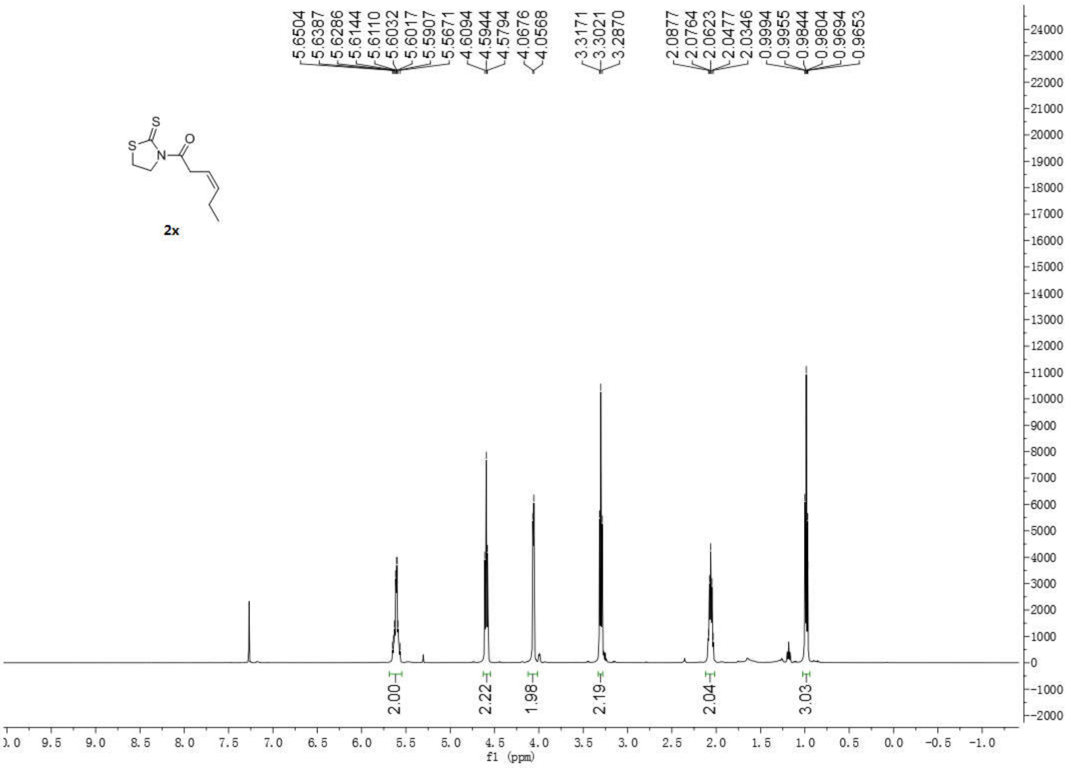

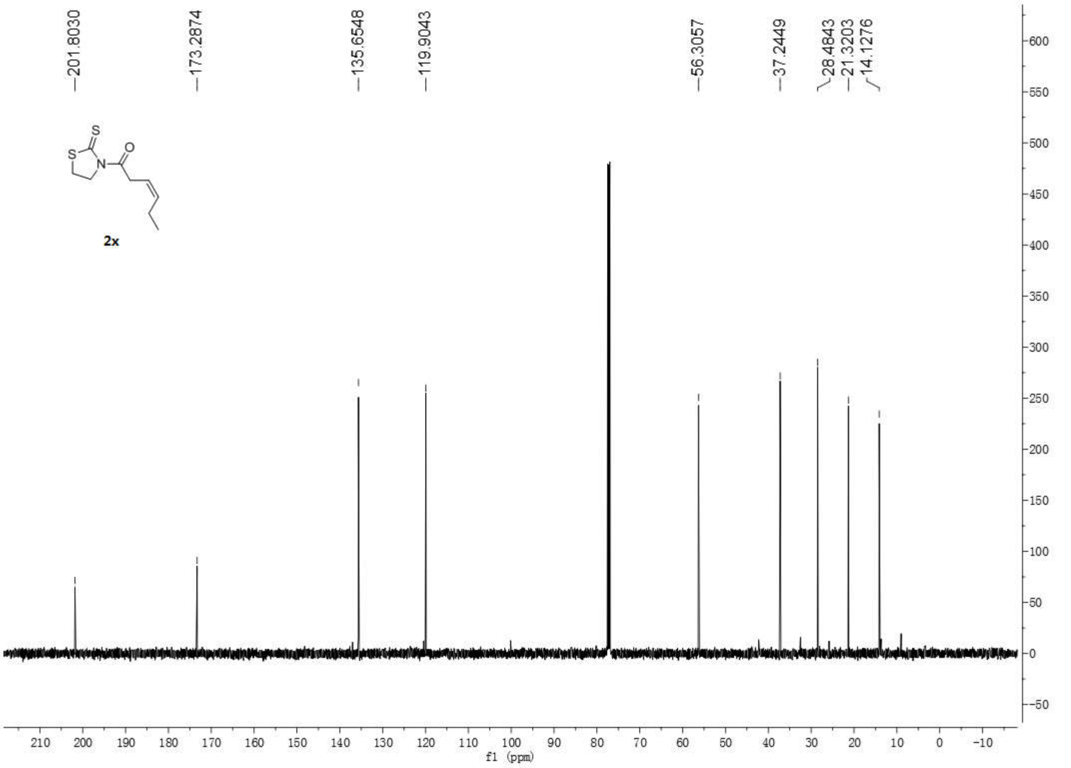

Supplementary figure 30. ^{1}H and ^{13}C NMR spectrum of compound **2x**

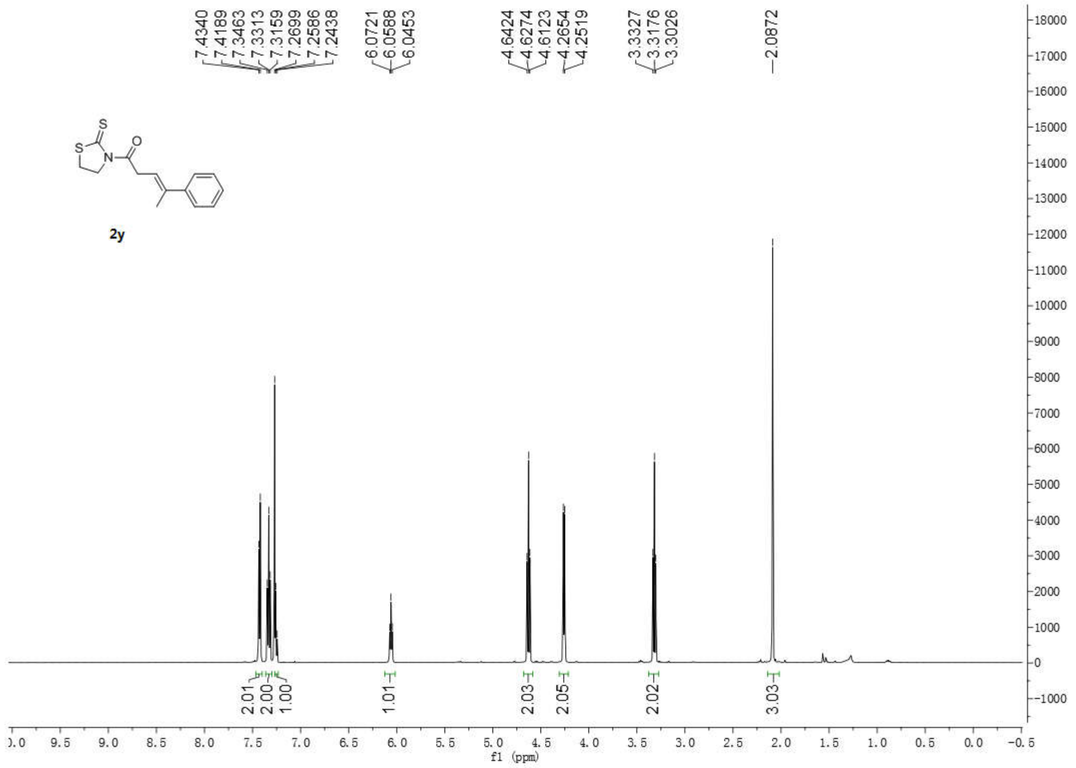

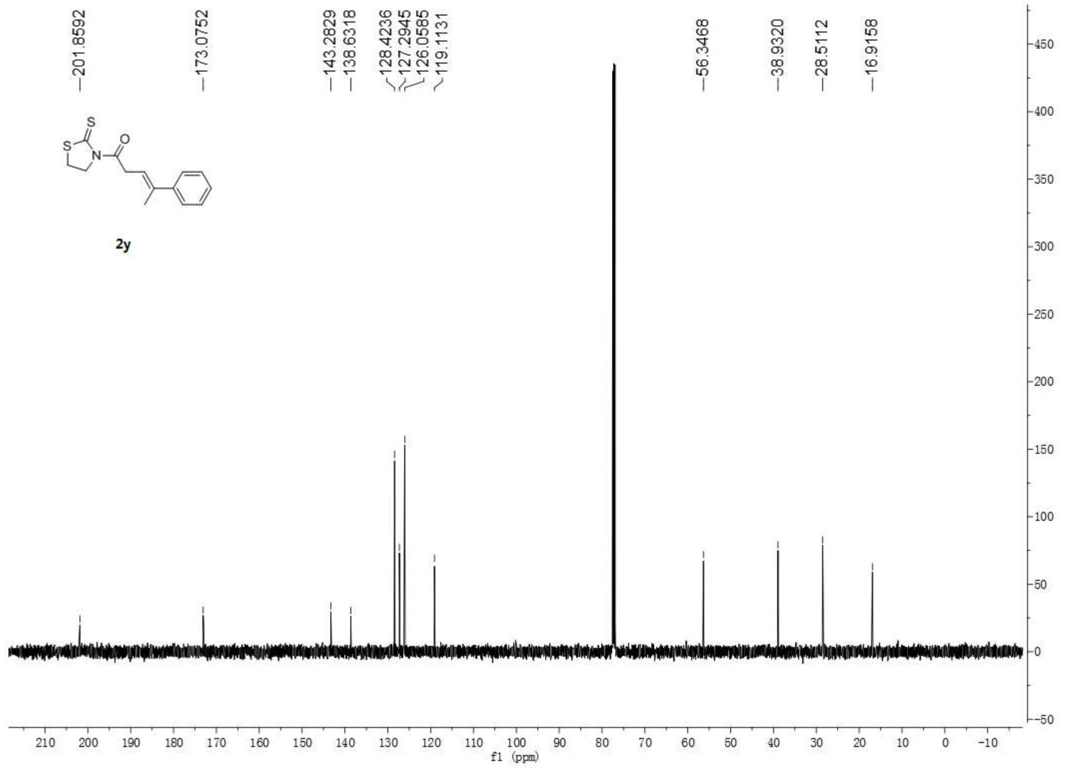

Supplementary figure 31. ^{1}H and ^{13}C NMR spectrum of compound **2y**

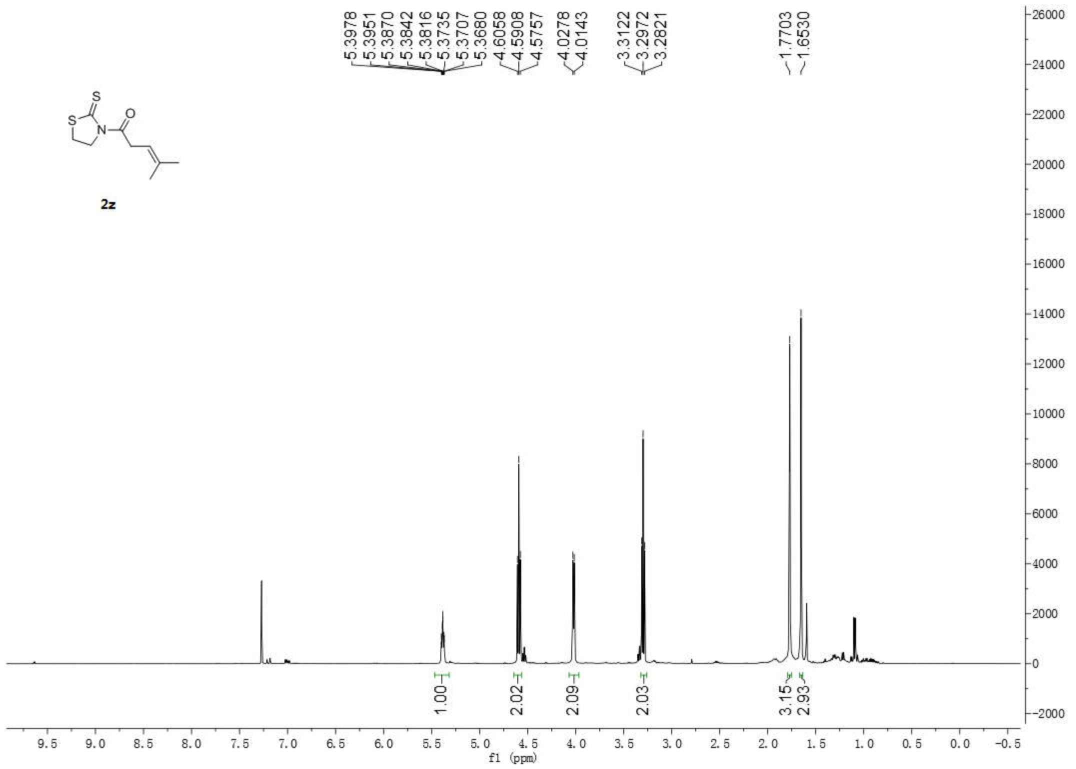

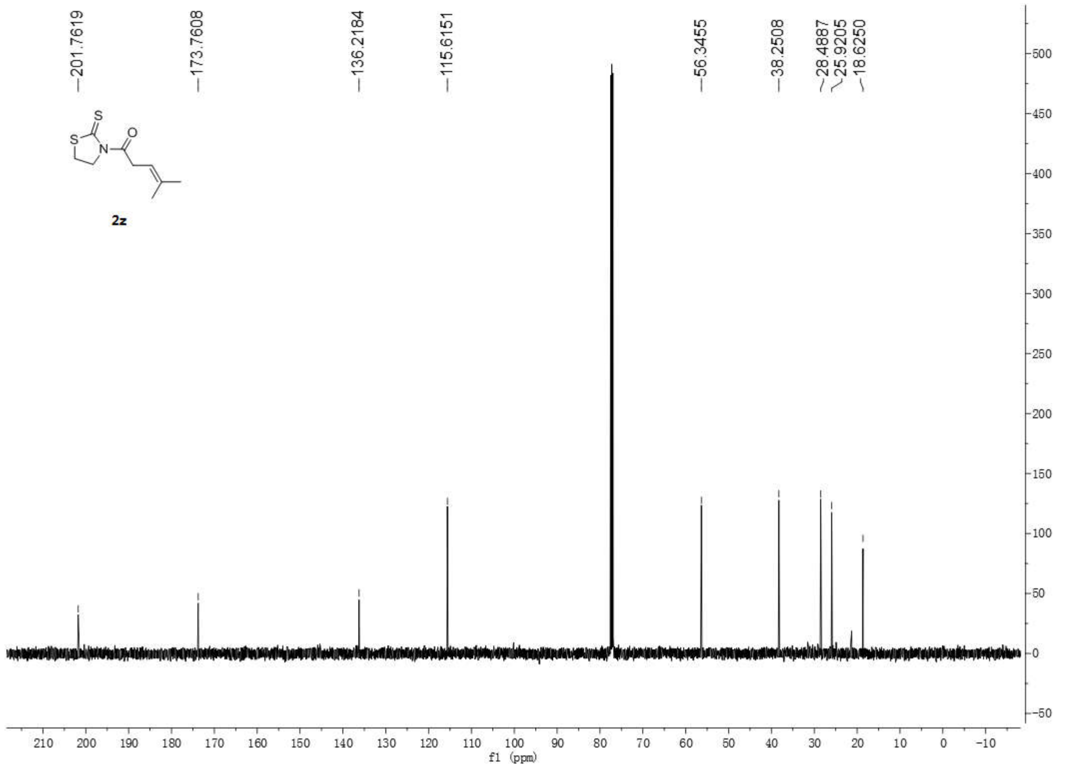

Supplementary figure 32. ^{1}H and ^{13}C NMR spectrum of compound **2z**

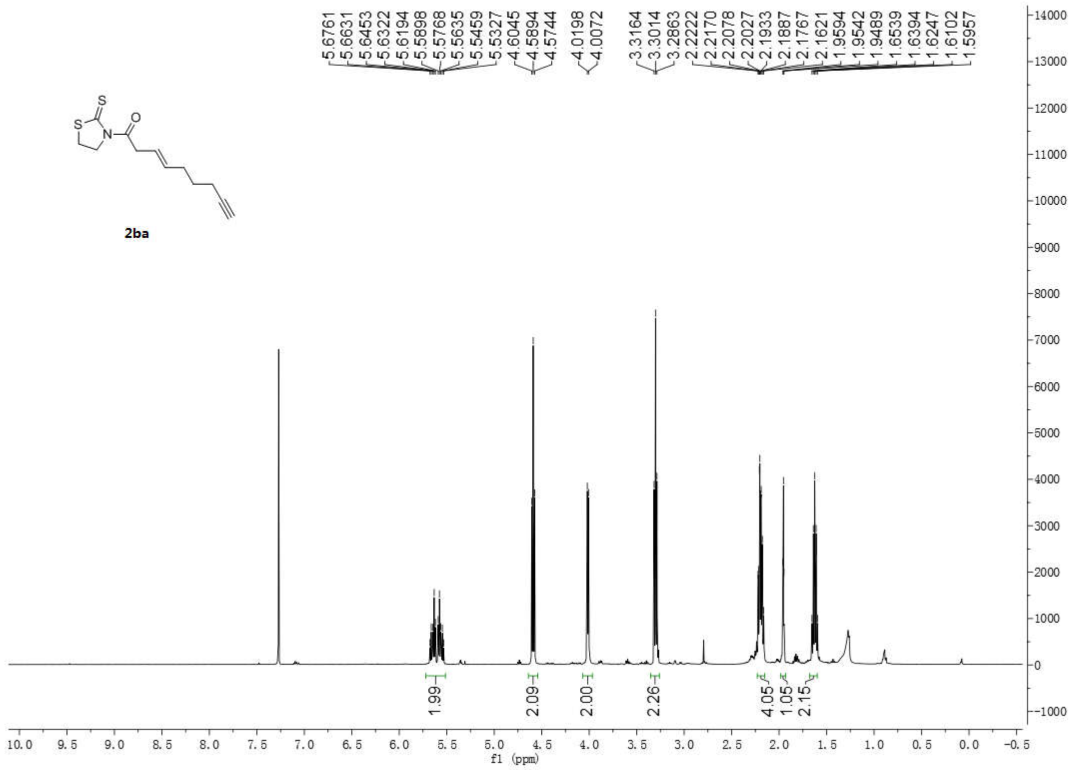

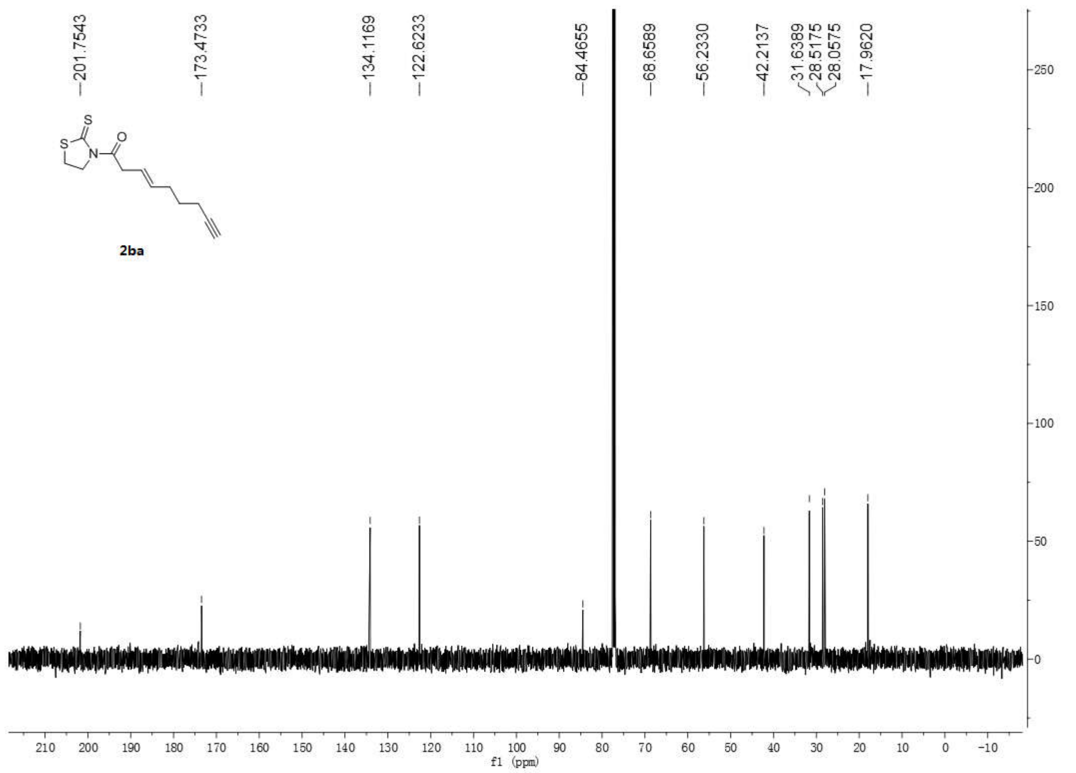

Supplementary figure 33. ^{1}H and ^{13}C NMR spectrum of compound **2ba**

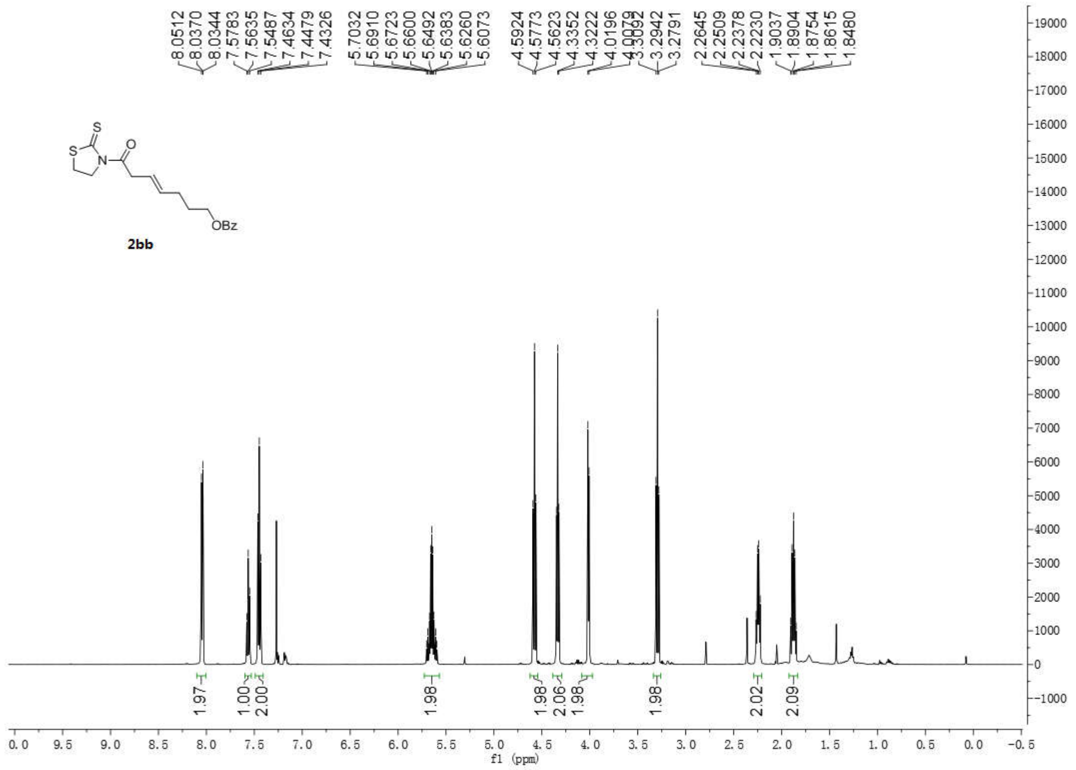

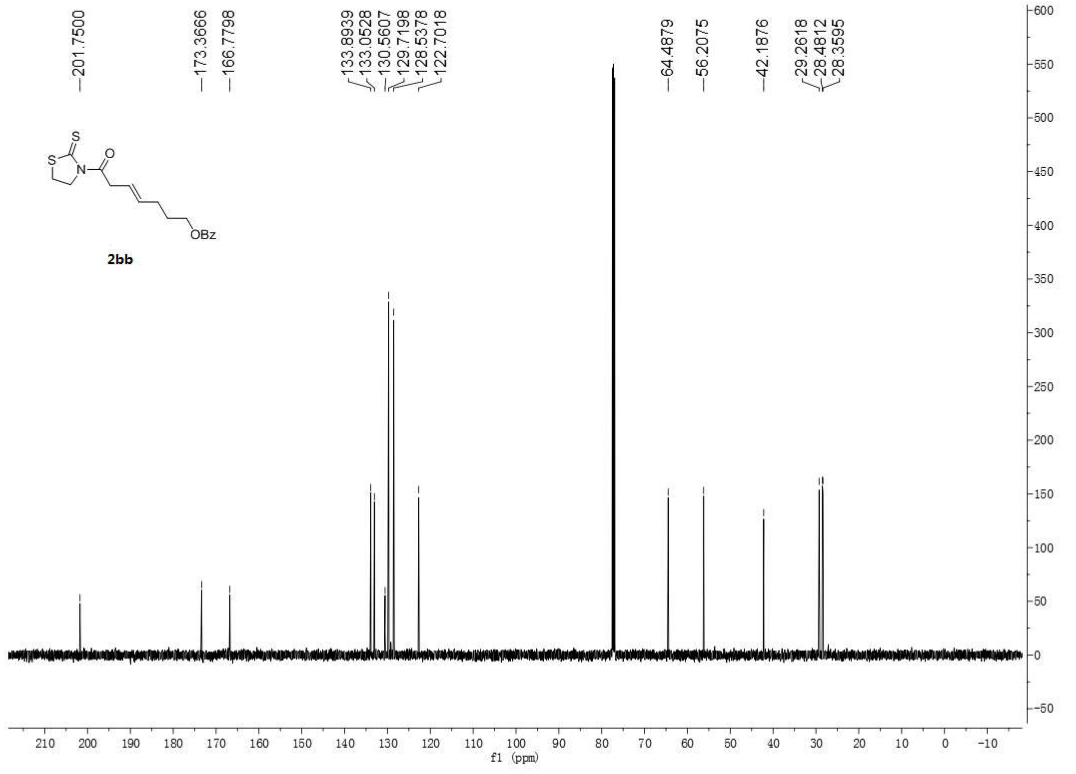

Supplementary figure 34. ^{1}H and ^{13}C NMR spectrum of compound **2bb**

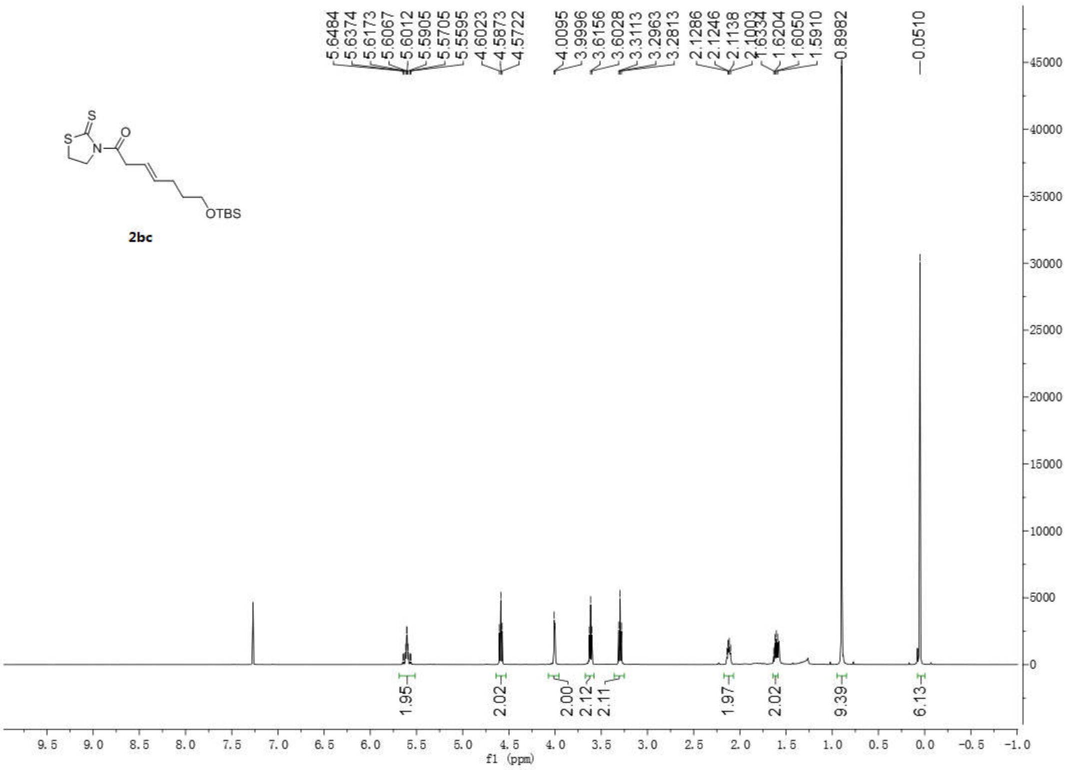

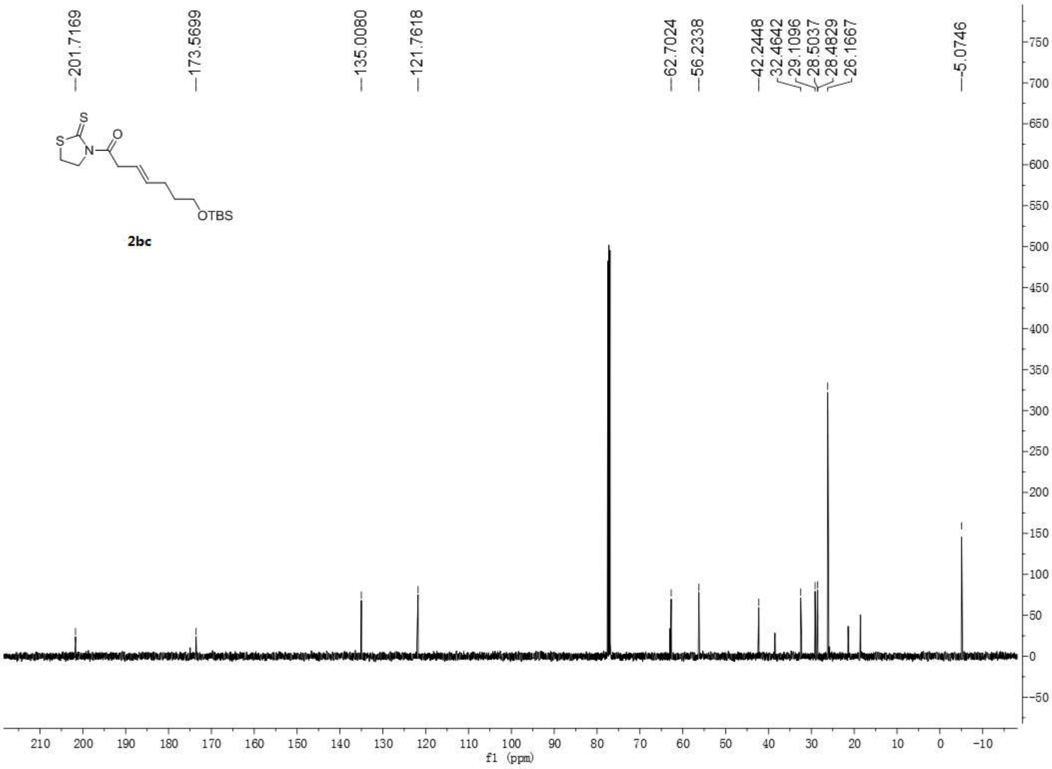

Supplementary figure 35. ^{1}H and ^{13}C NMR spectrum of compound **2bc**

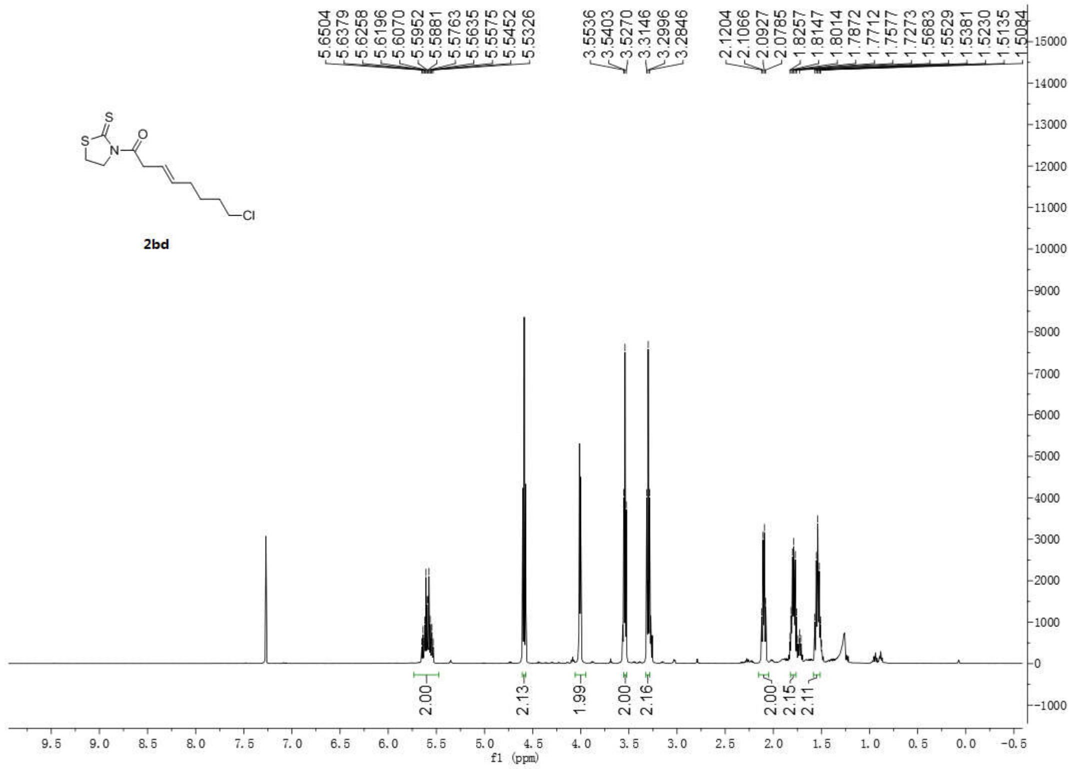

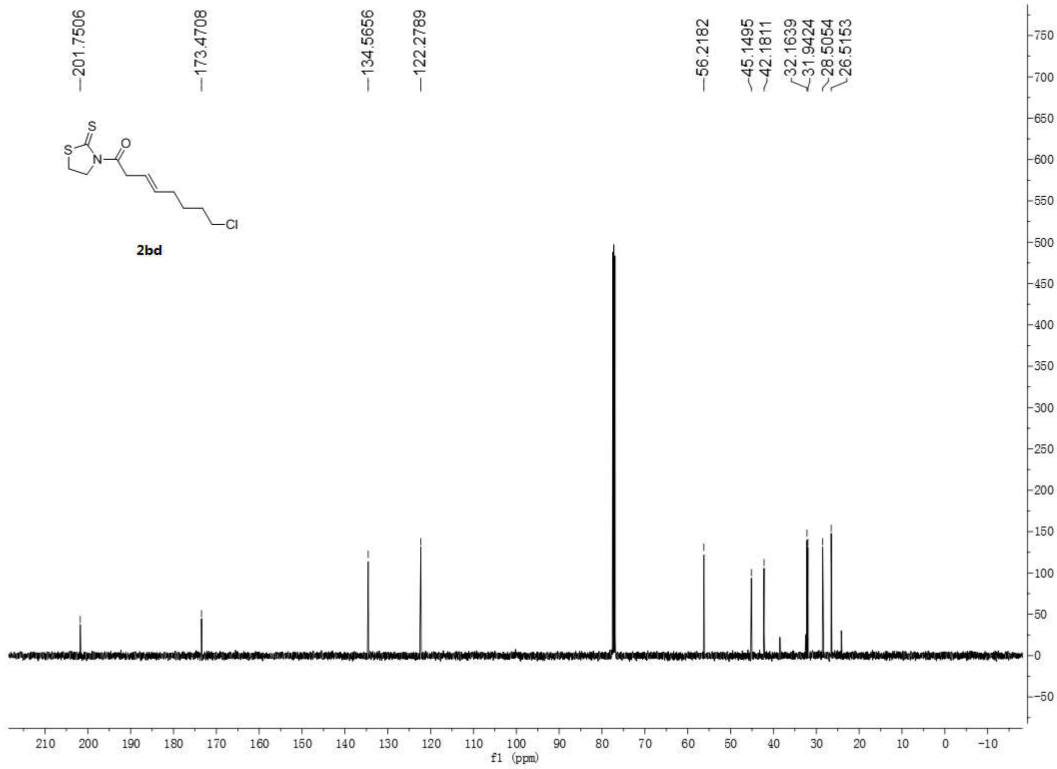

Supplementary figure 36. ^{1}H and ^{13}C NMR spectrum of compound **2bd**

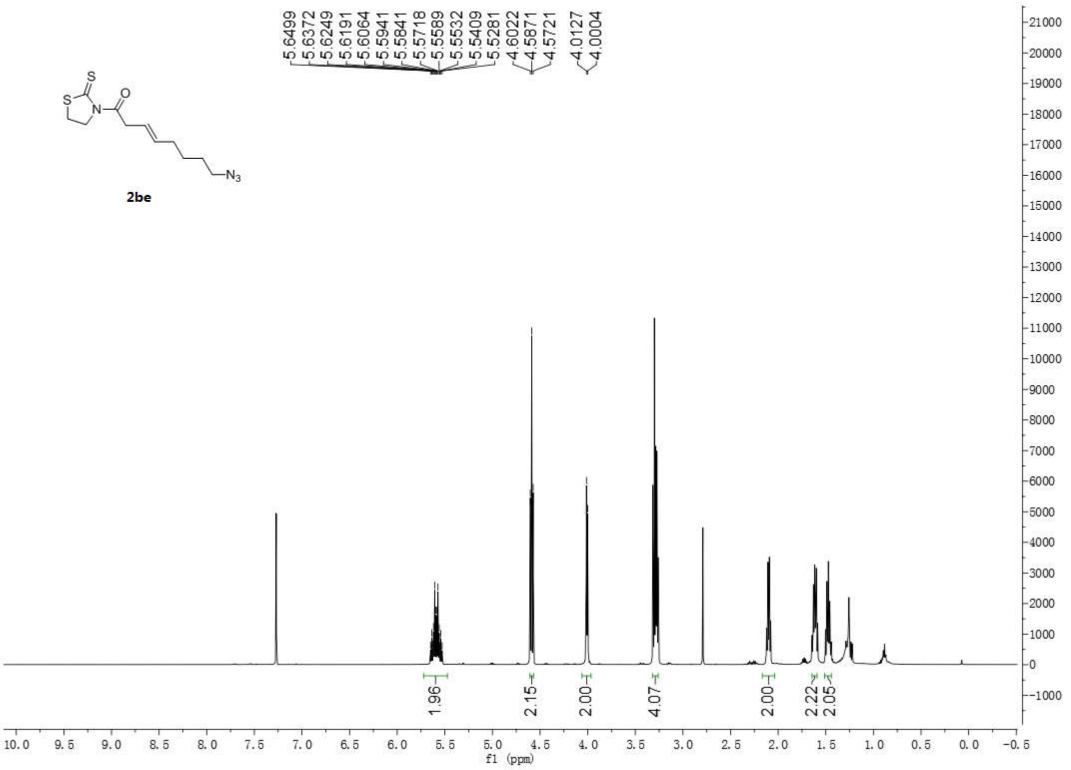

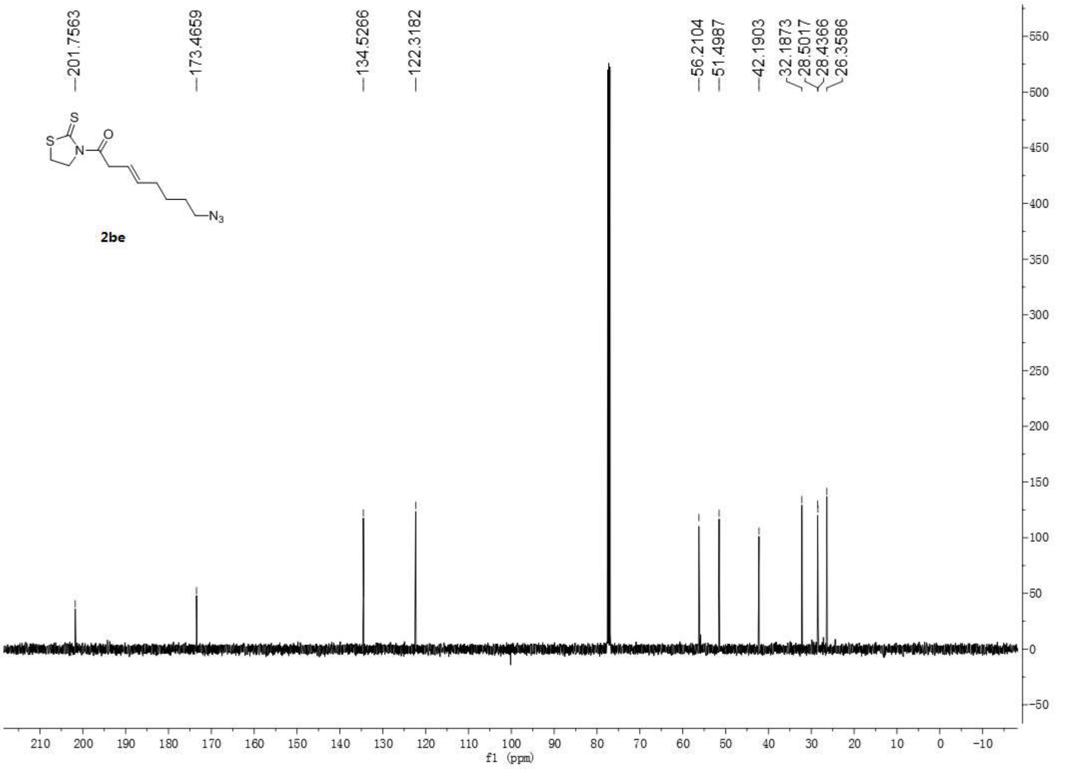

Supplementary figure 37. ^{1}H and ^{13}C NMR spectrum of compound **2be**

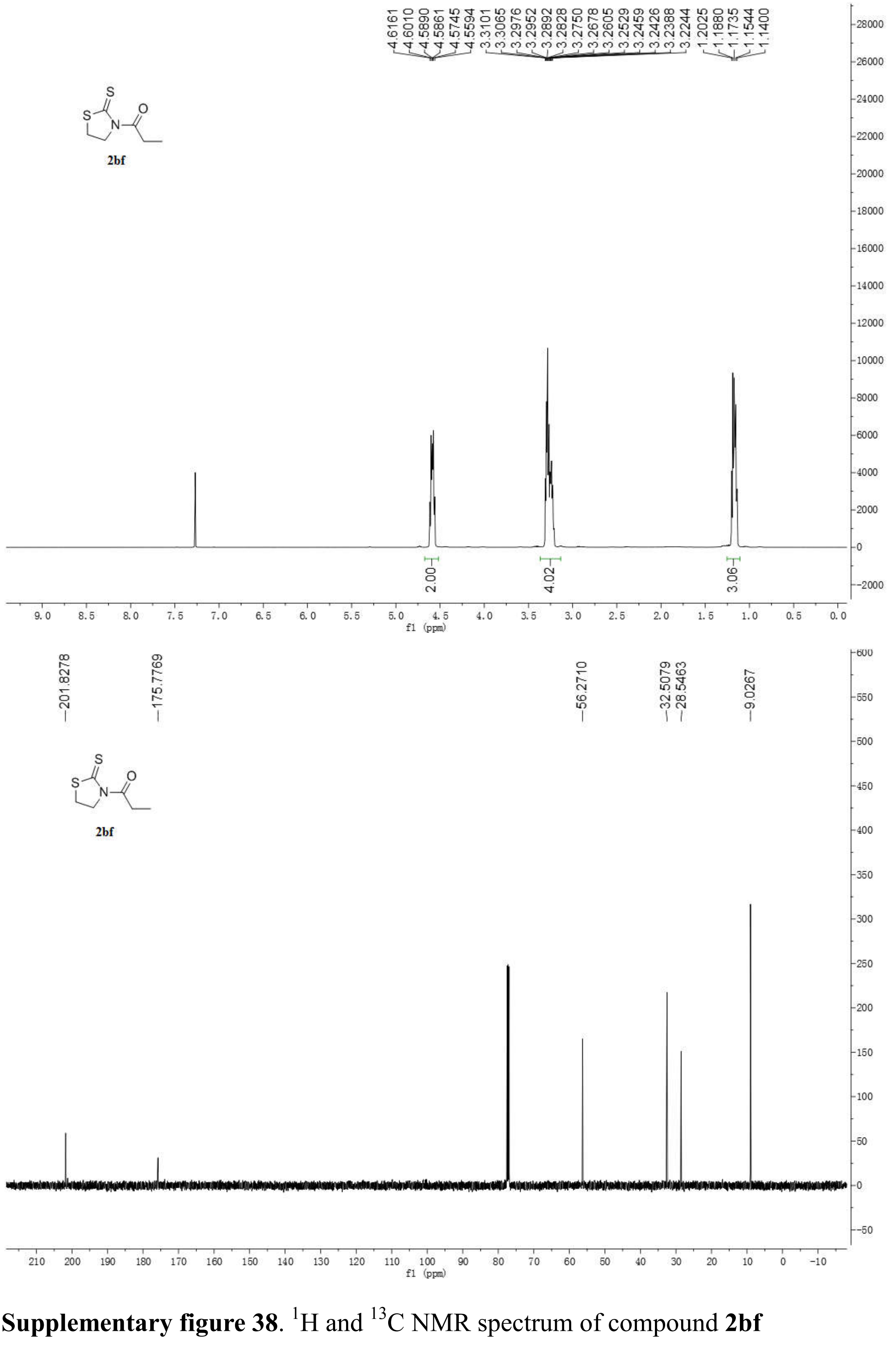

Supplementary figure 38. ^{1}H and ^{13}C NMR spectrum of compound **2bf**

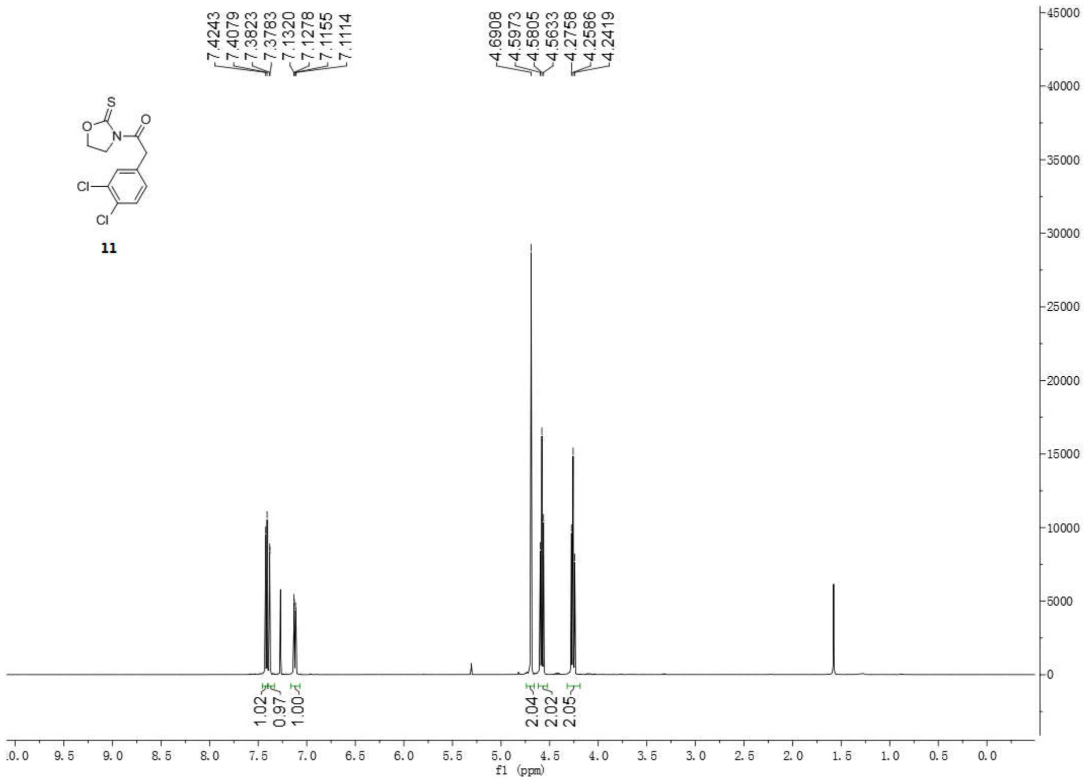

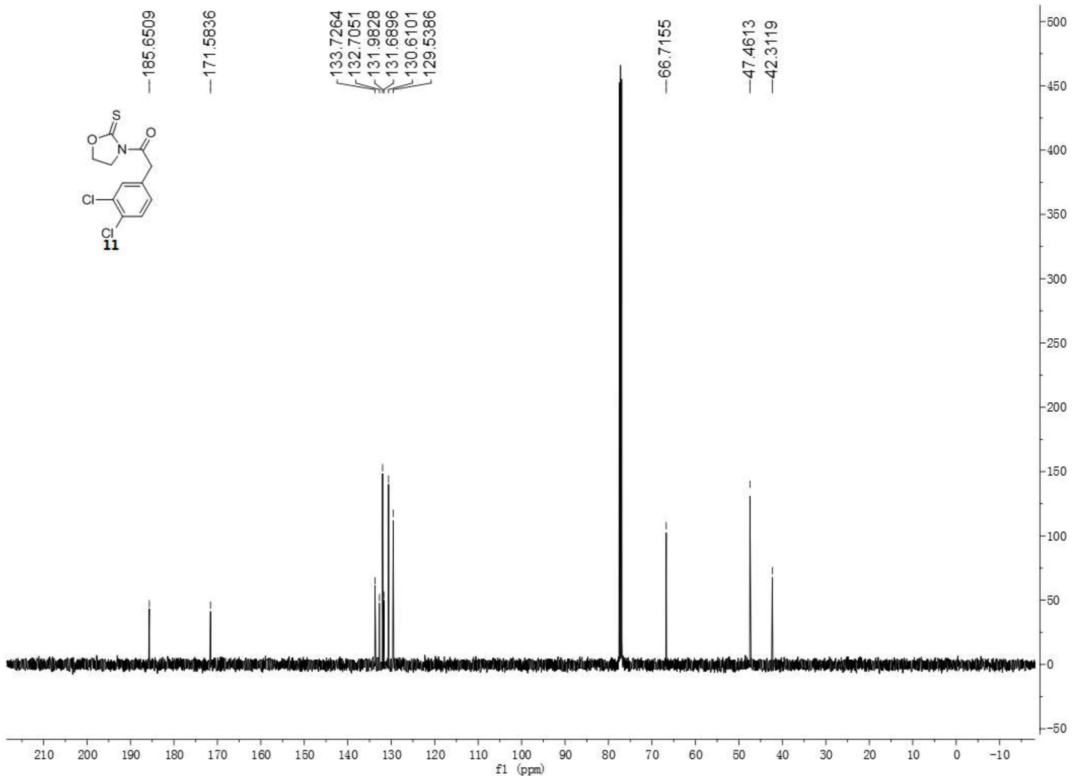

Supplementary figure 39. ^{1}H and ^{13}C NMR spectrum of compound **11**

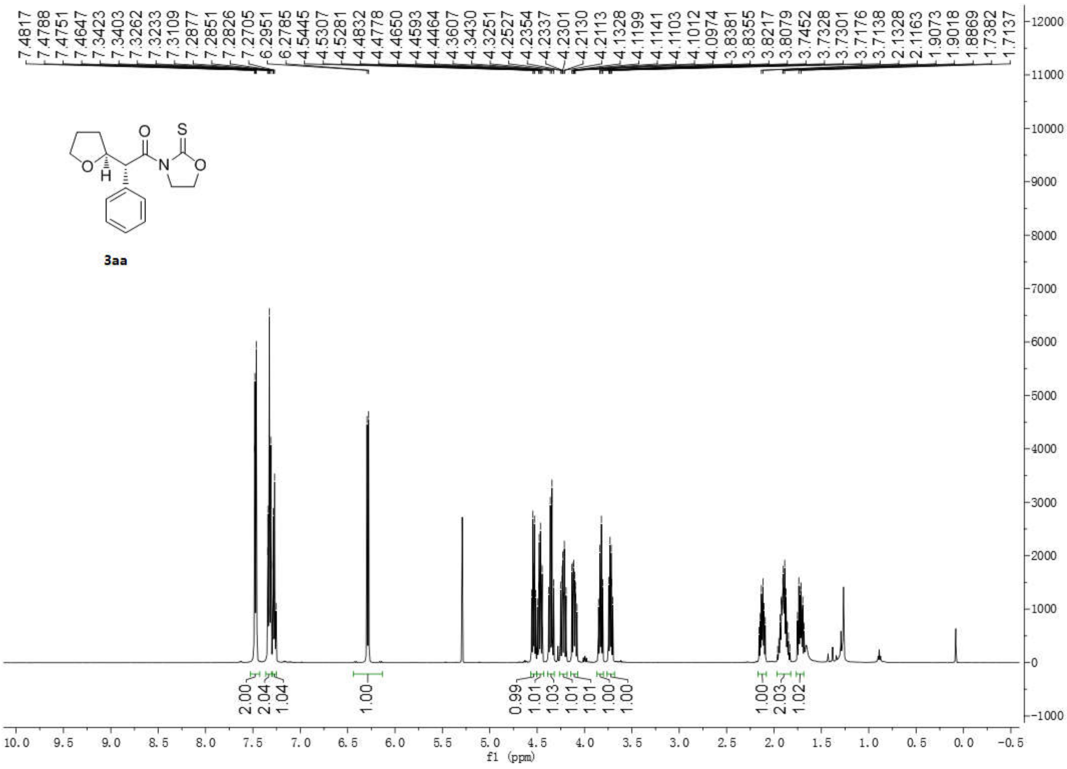

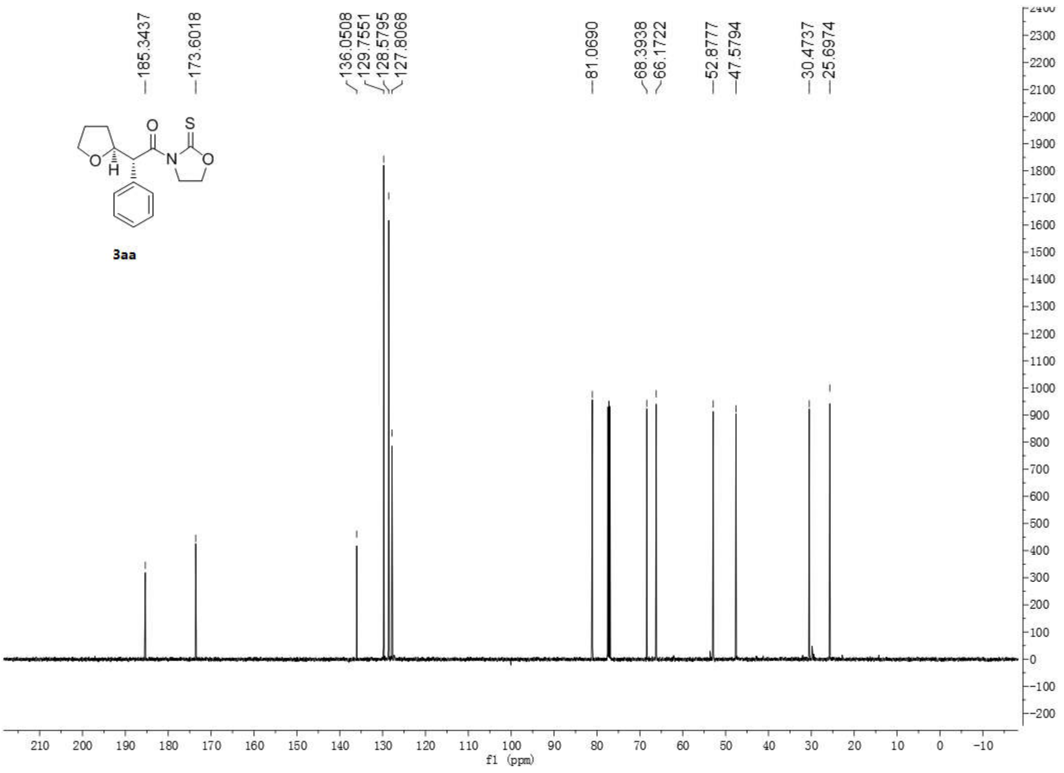

Supplementary figure 40. ^{1}H and ^{13}C NMR spectrum of compound **3aa**

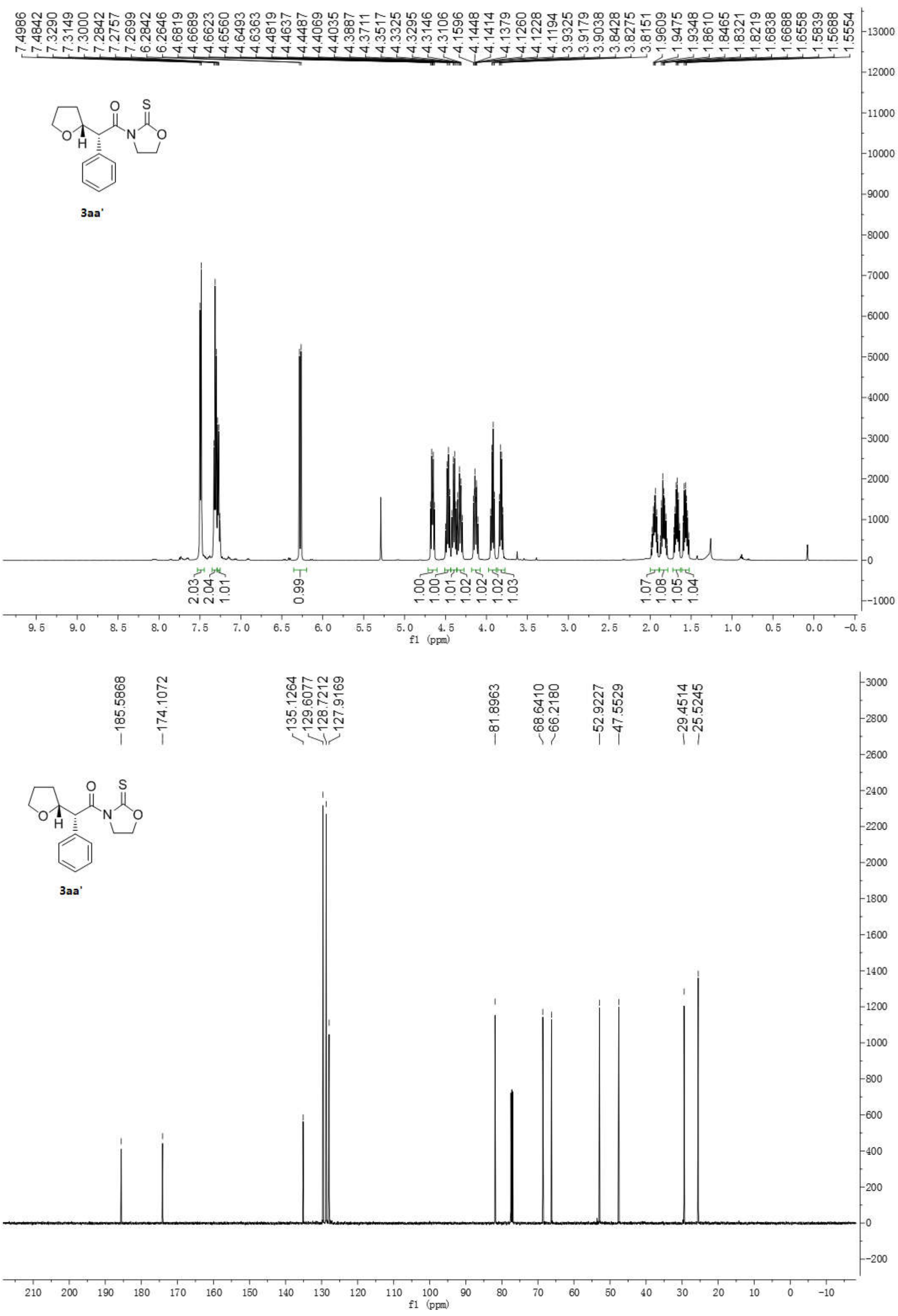

Supplementary figure 41. ^{1}H and ^{13}C NMR spectrum of compound **3aa’**

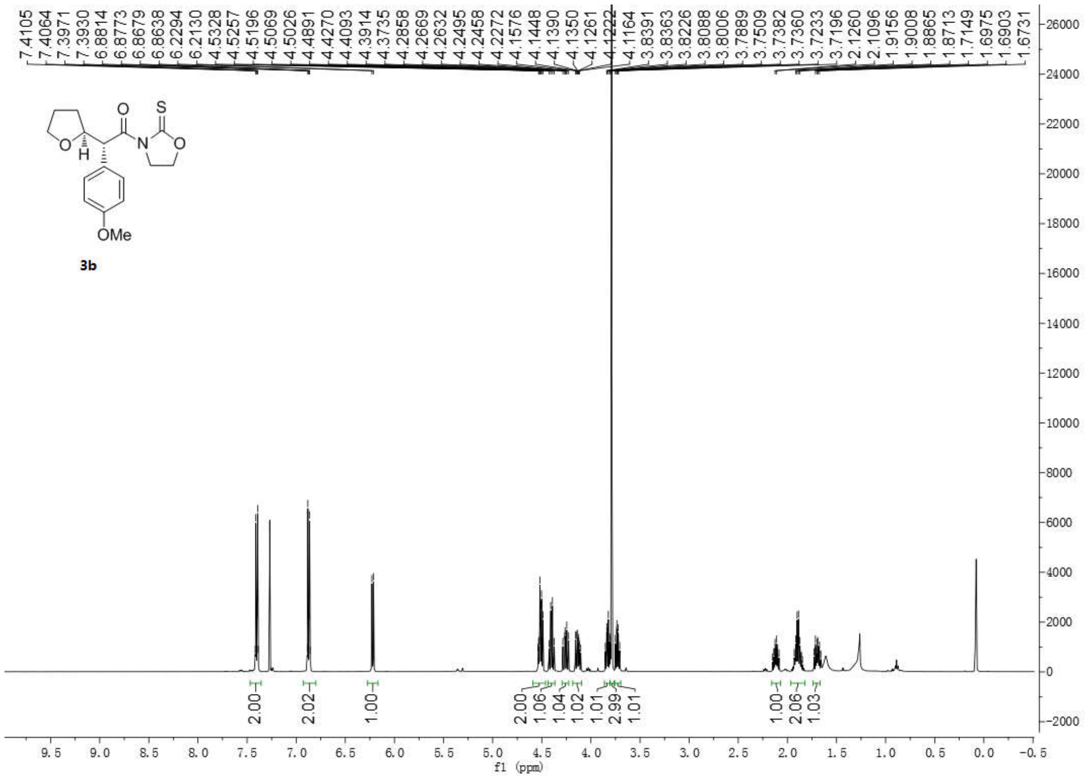

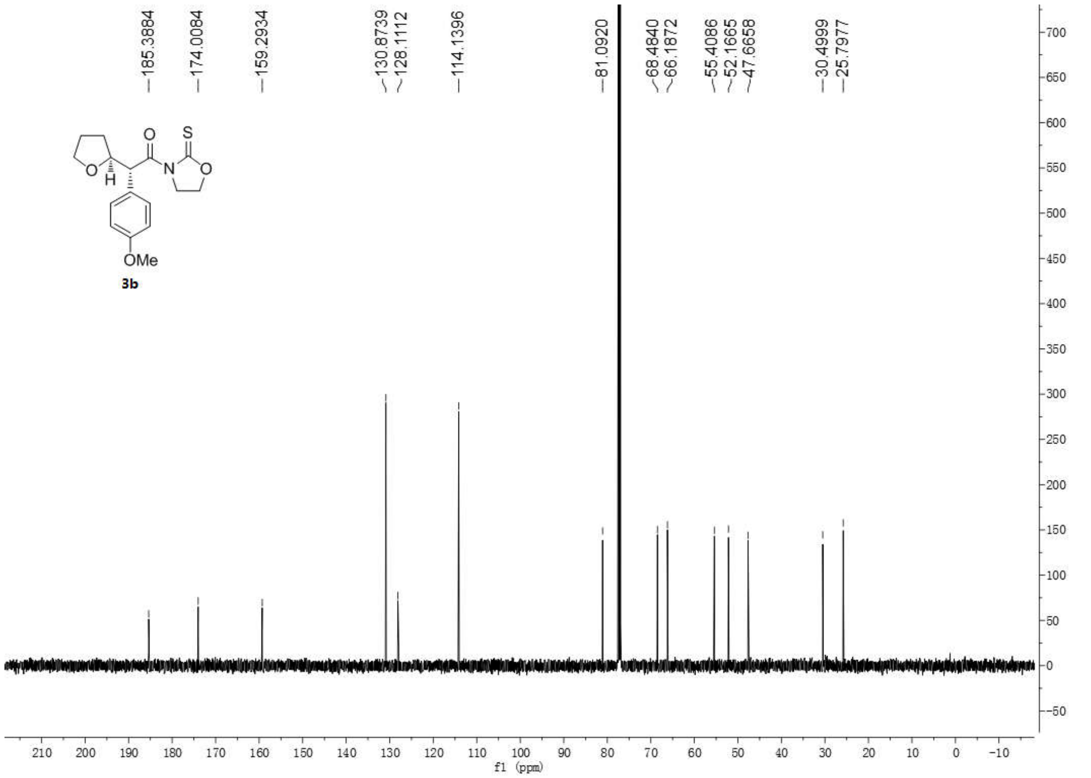

Supplementary figure 42. ^{1}H and ^{13}C NMR spectrum of compound **3b**

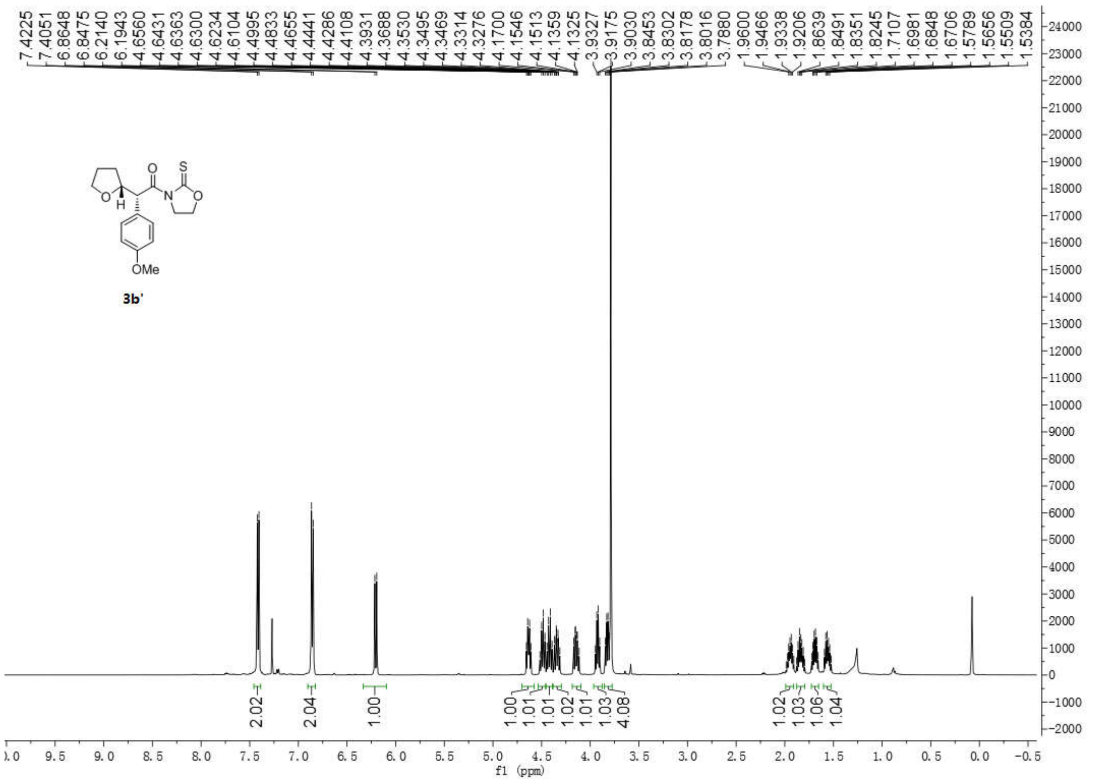

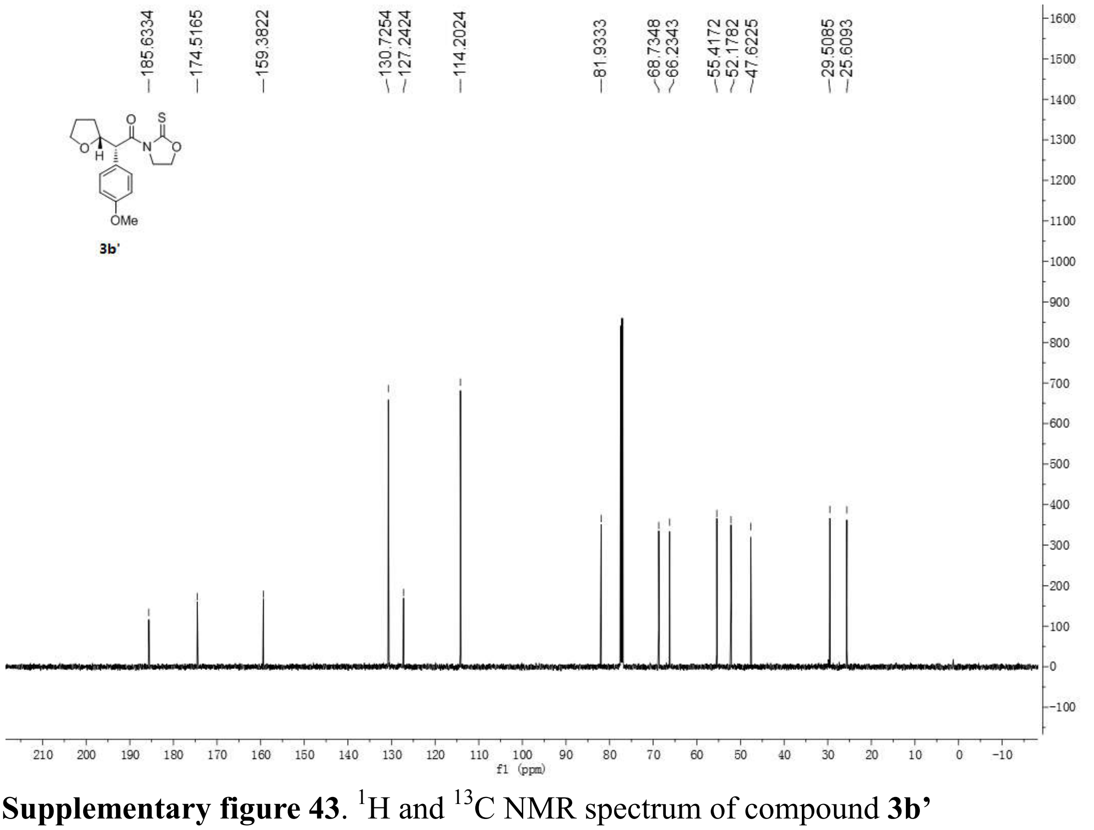

Supplementary figure 43. ^{1}H and ^{13}C NMR spectrum of compound **3b’**

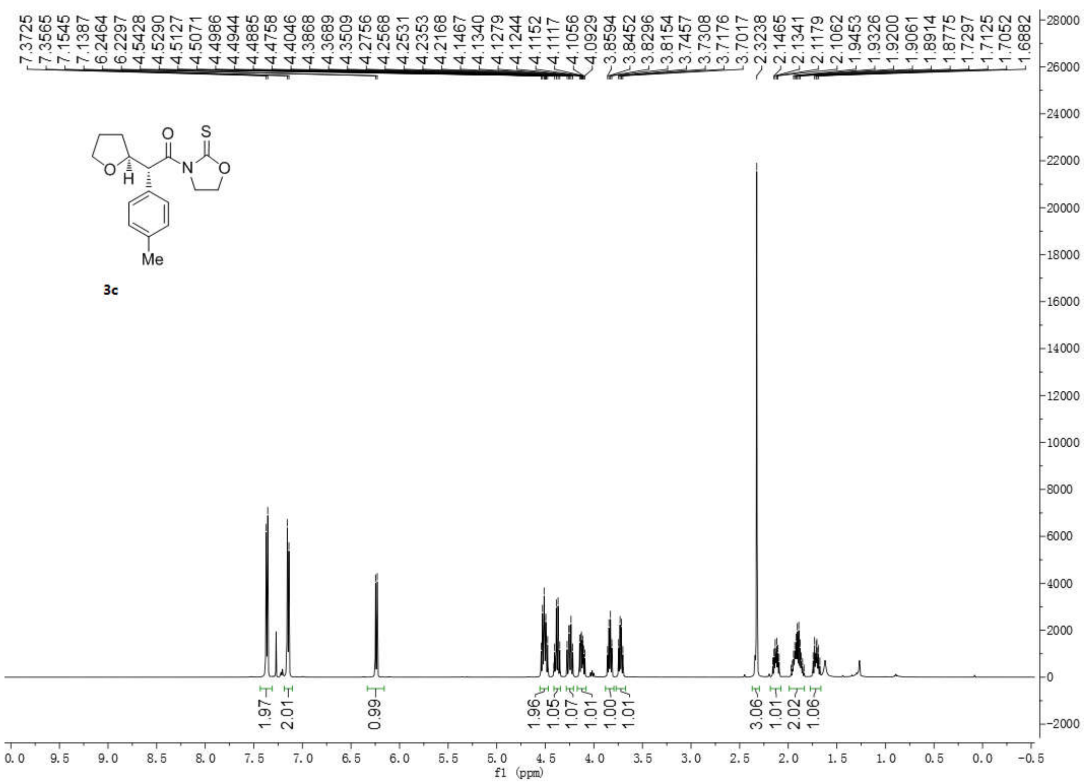

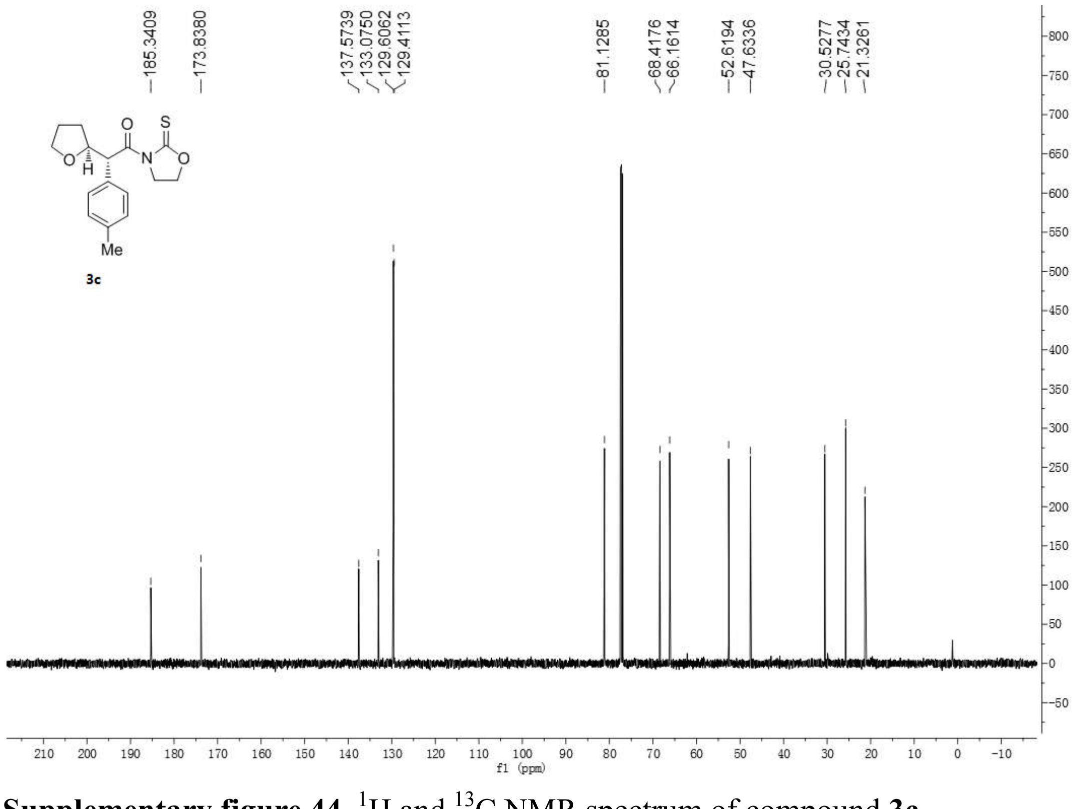

Supplementary figure 44. ^{1}H and ^{13}C NMR spectrum of compound **3c**

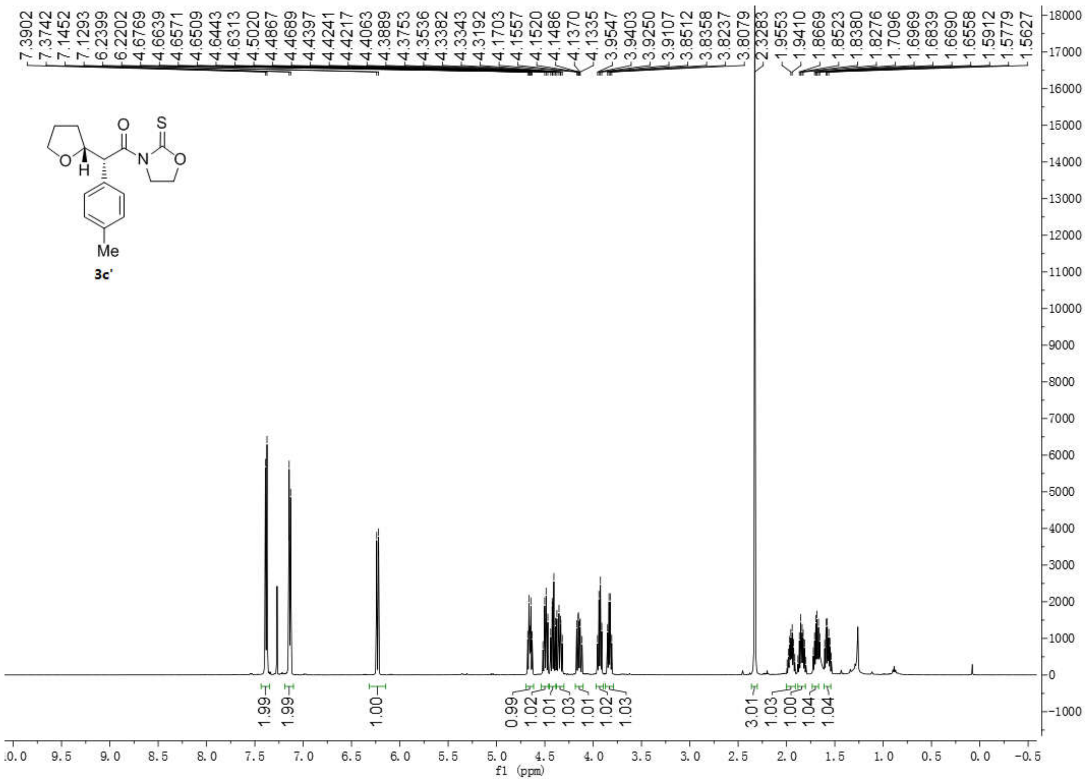

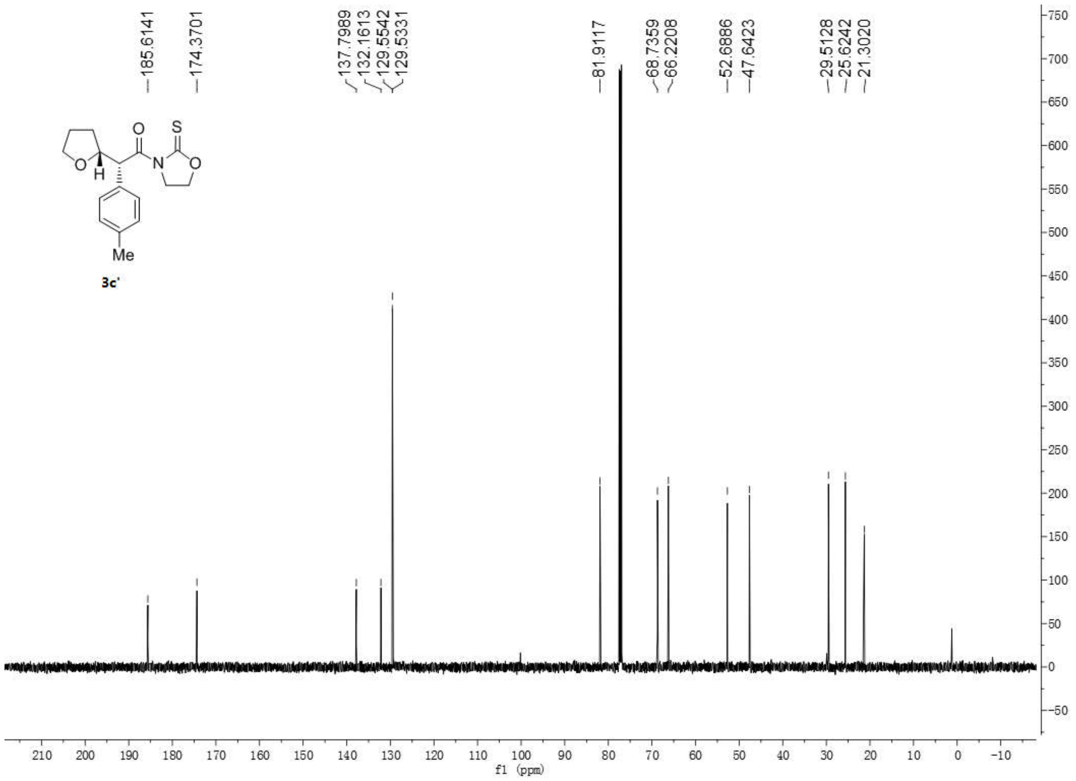

Supplementary figure 45. ^{1}H and ^{13}C NMR spectrum of compound **3c’**

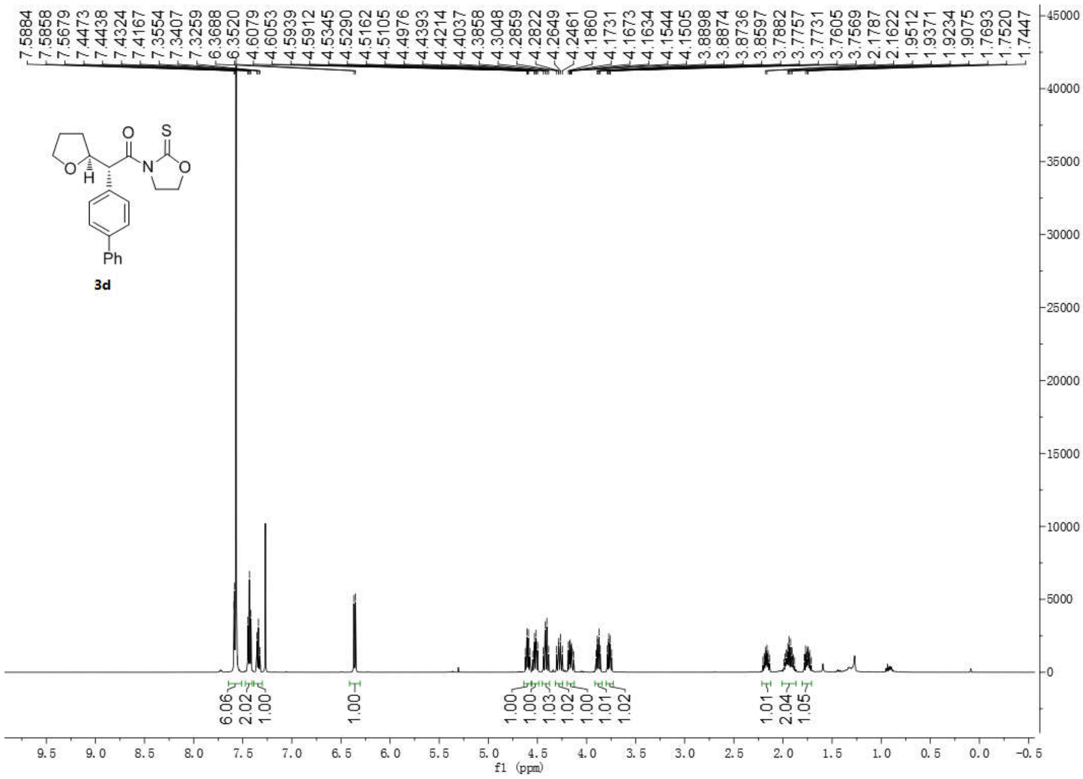

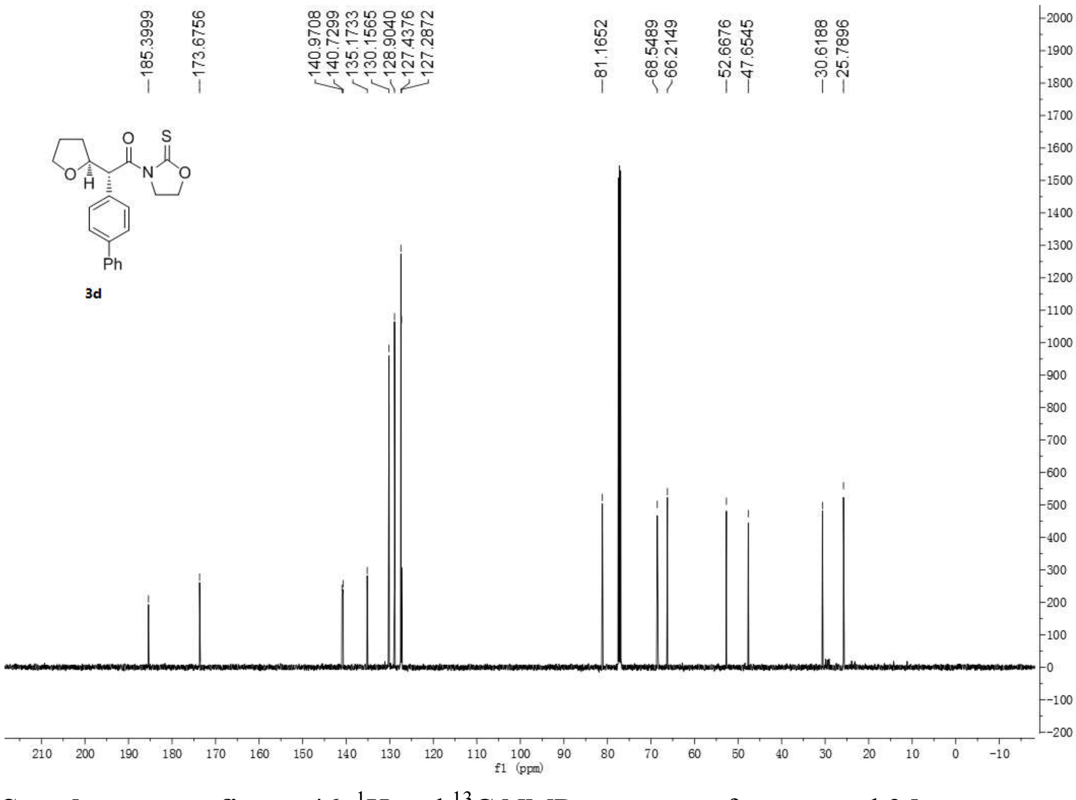

Supplementary figure 46. ^{1}H and ^{13}C NMR spectrum of compound **3d**

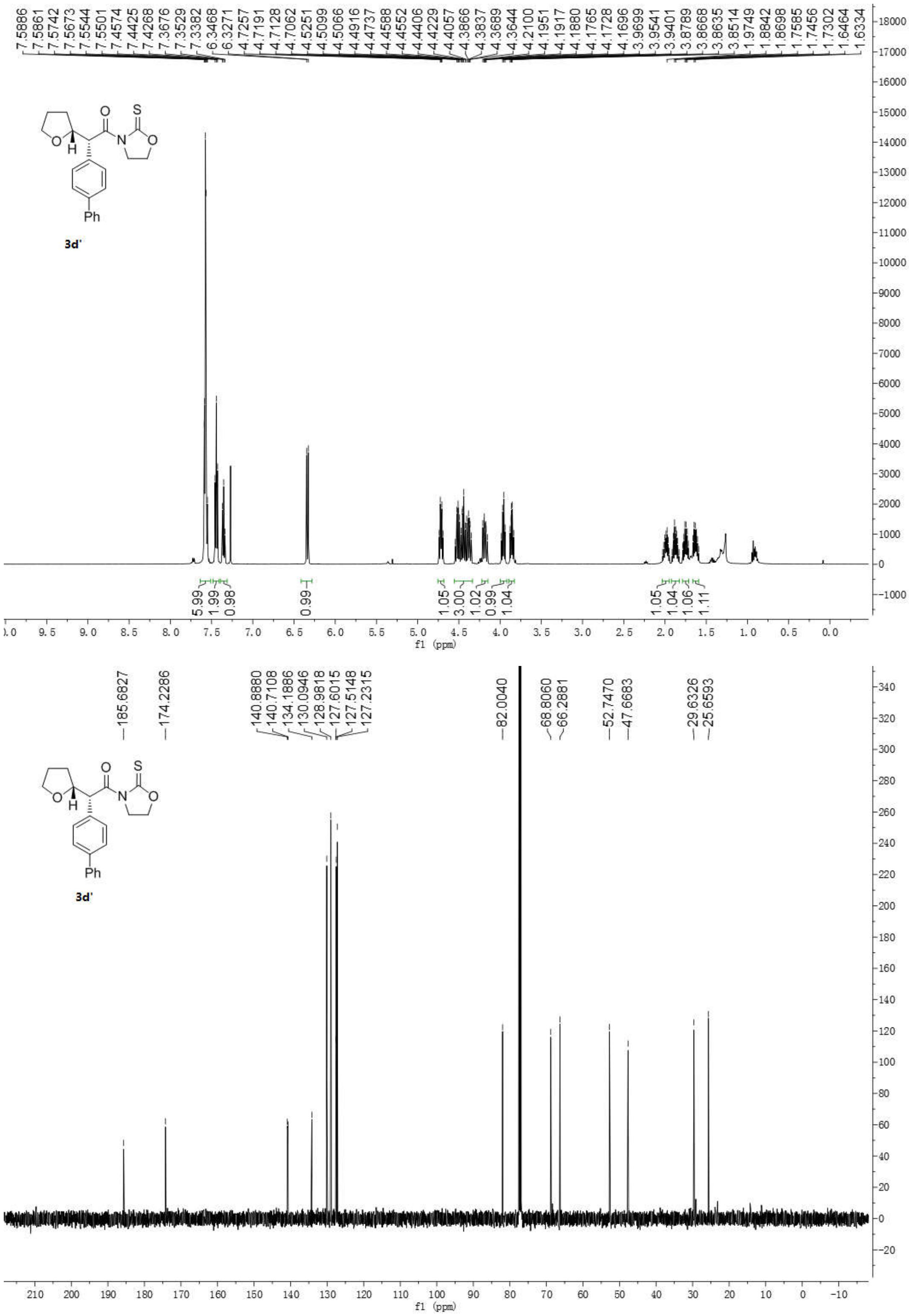

Supplementary figure 47. ^{1}H and ^{13}C NMR spectrum of compound **3d’**

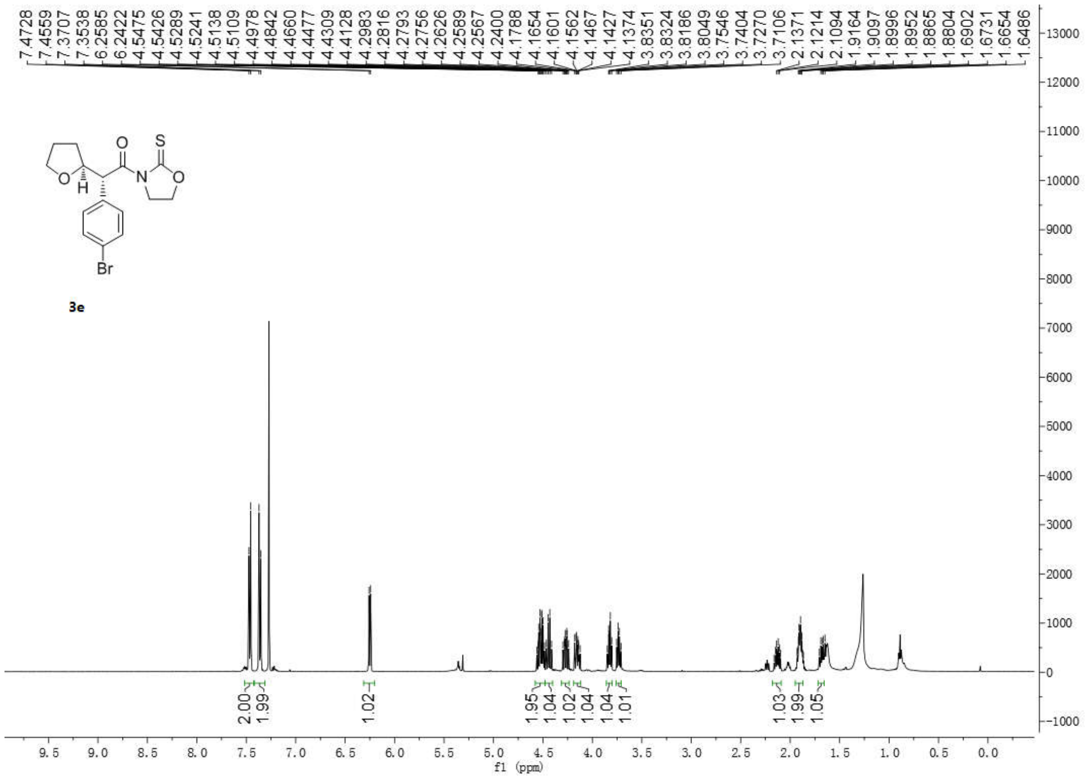

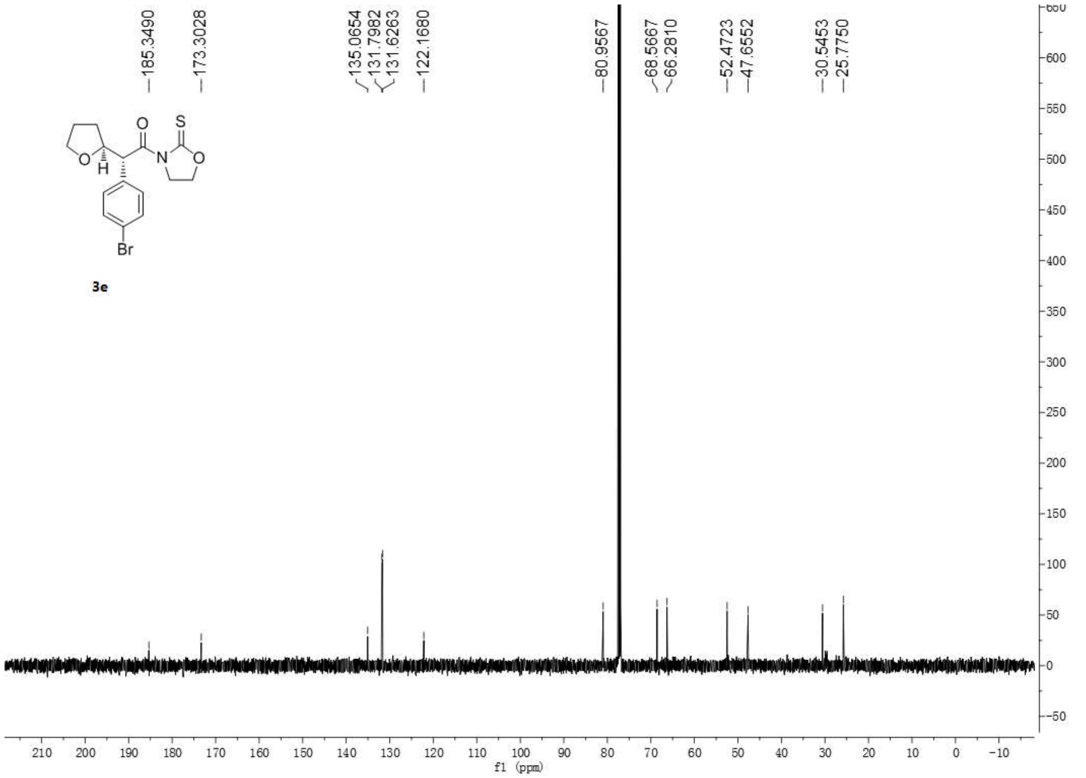

Supplementary figure 48. ^{1}H and ^{13}C NMR spectrum of compound **3e**

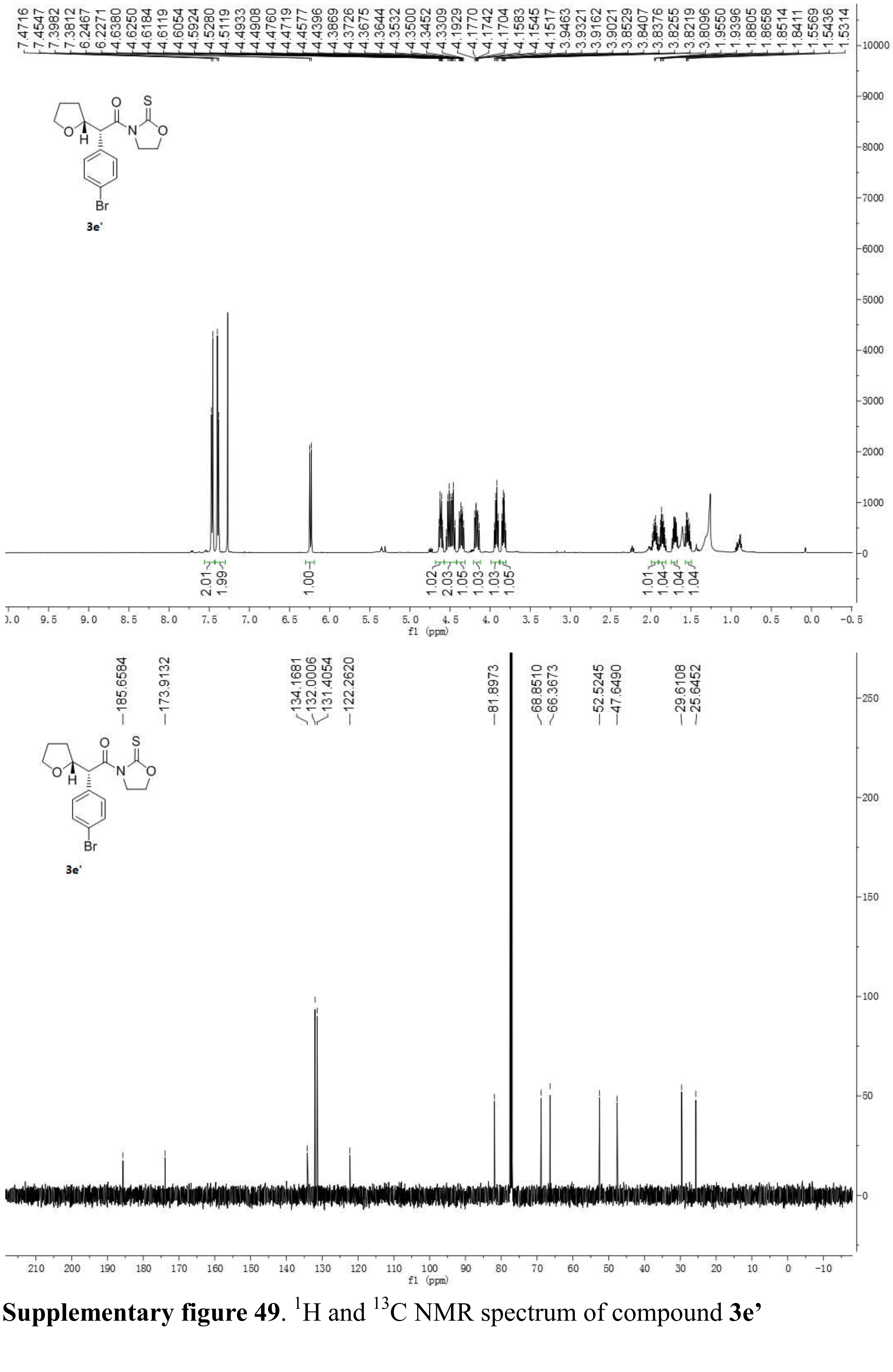

Supplementary figure 49. ^{1}H and ^{13}C NMR spectrum of compound **3e'**

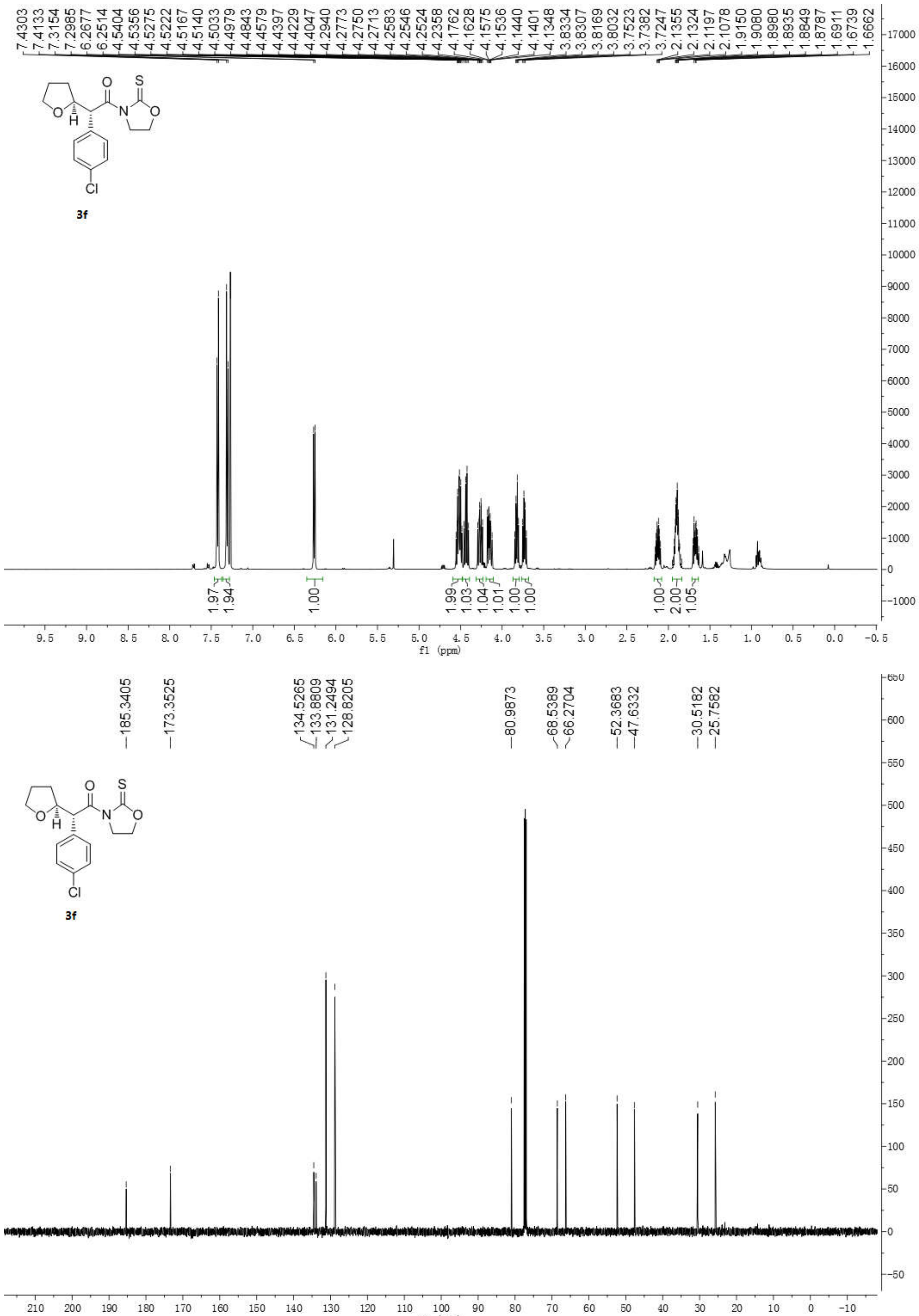

Supplementary figure 50. ^{1}H and ^{13}C NMR spectrum of compound **3f**

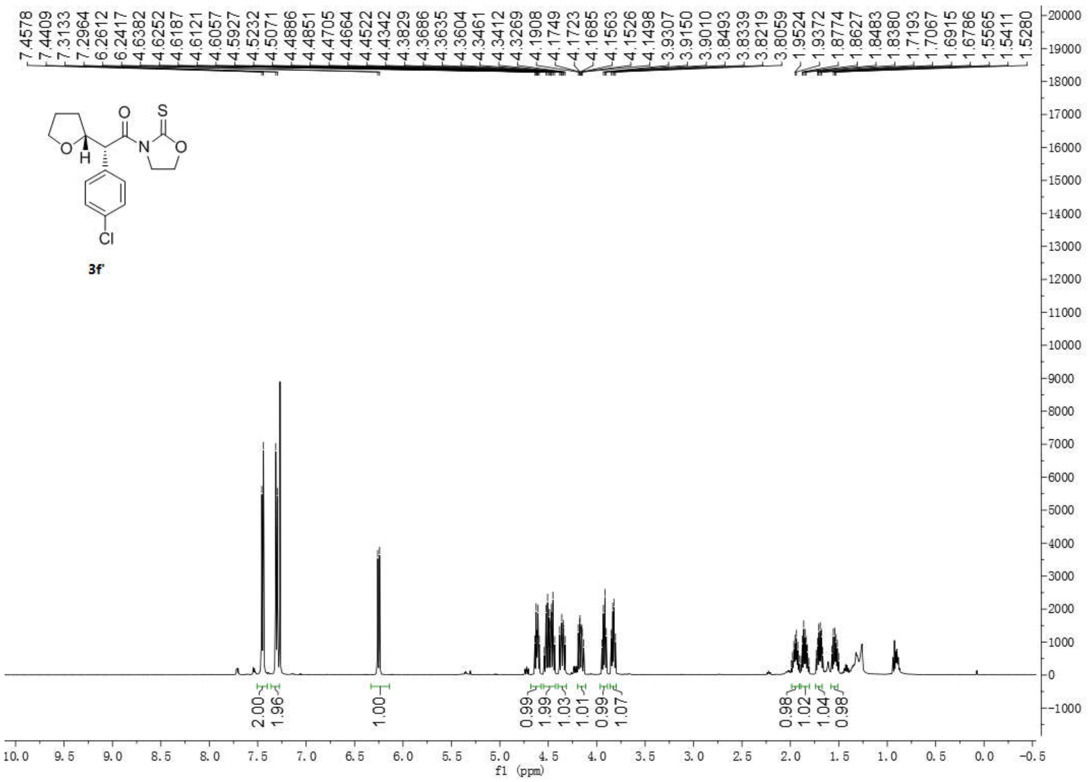

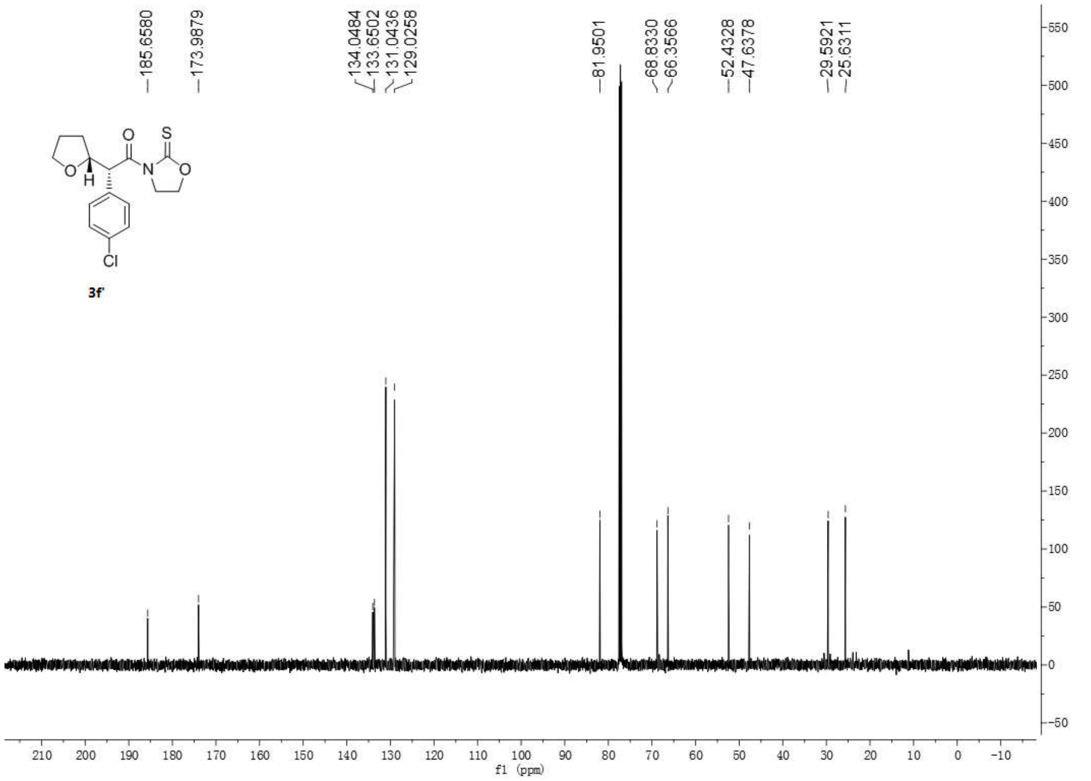

Supplementary figure 51. ^{1}H and ^{13}C NMR spectrum of compound **3f’**

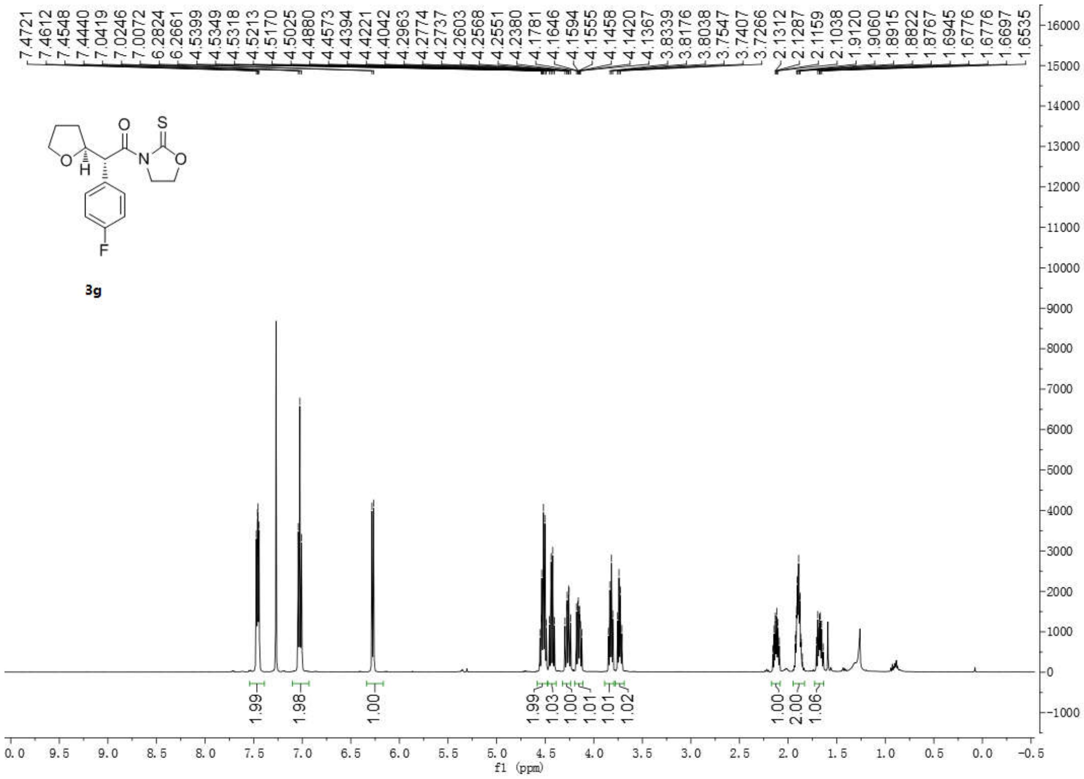

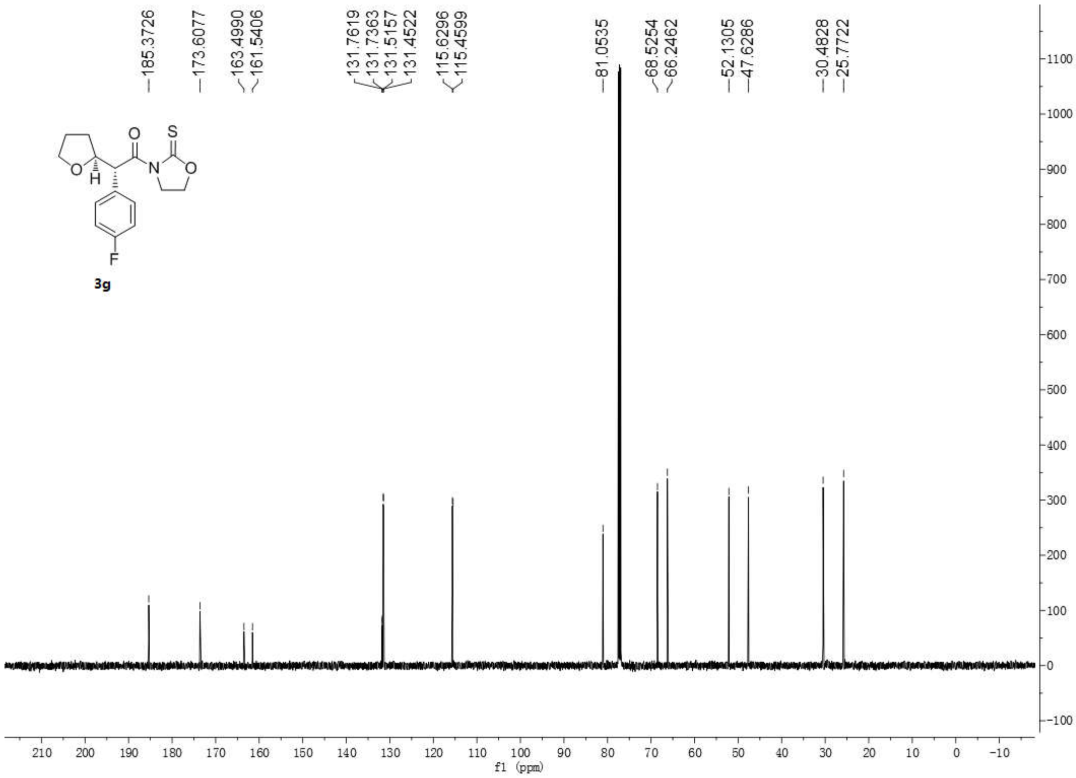

Supplementary figure 52. ^{1}H and ^{13}C NMR spectrum of compound **3g**

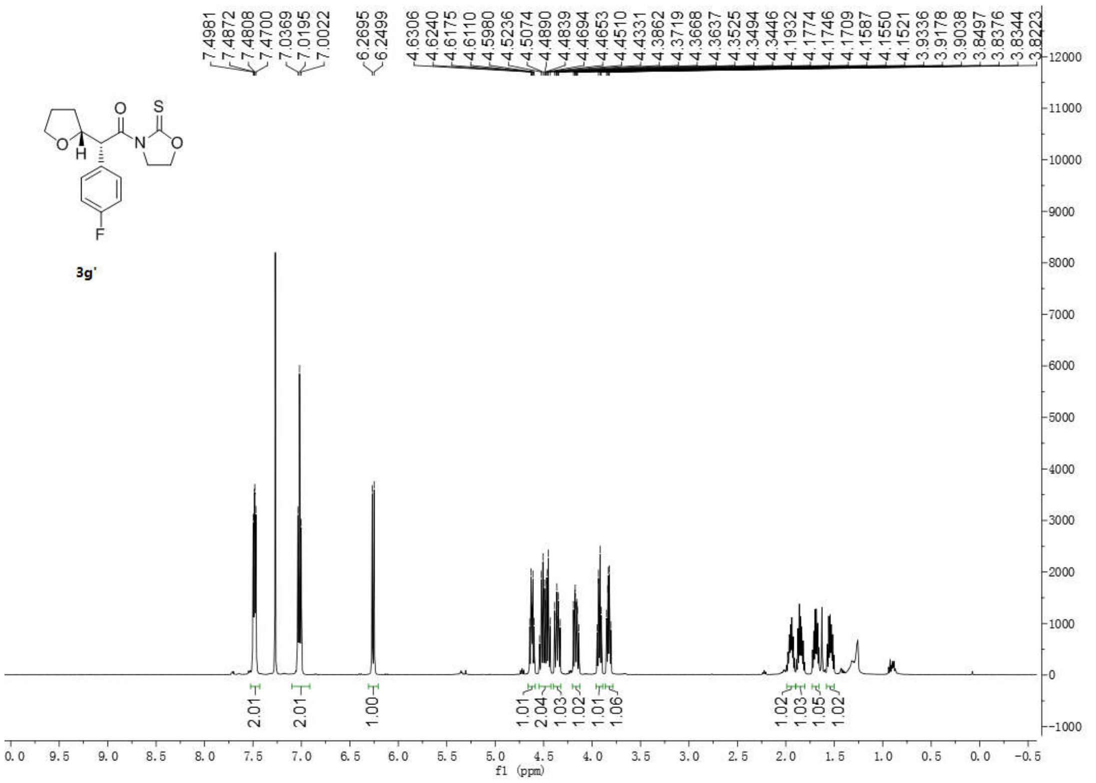

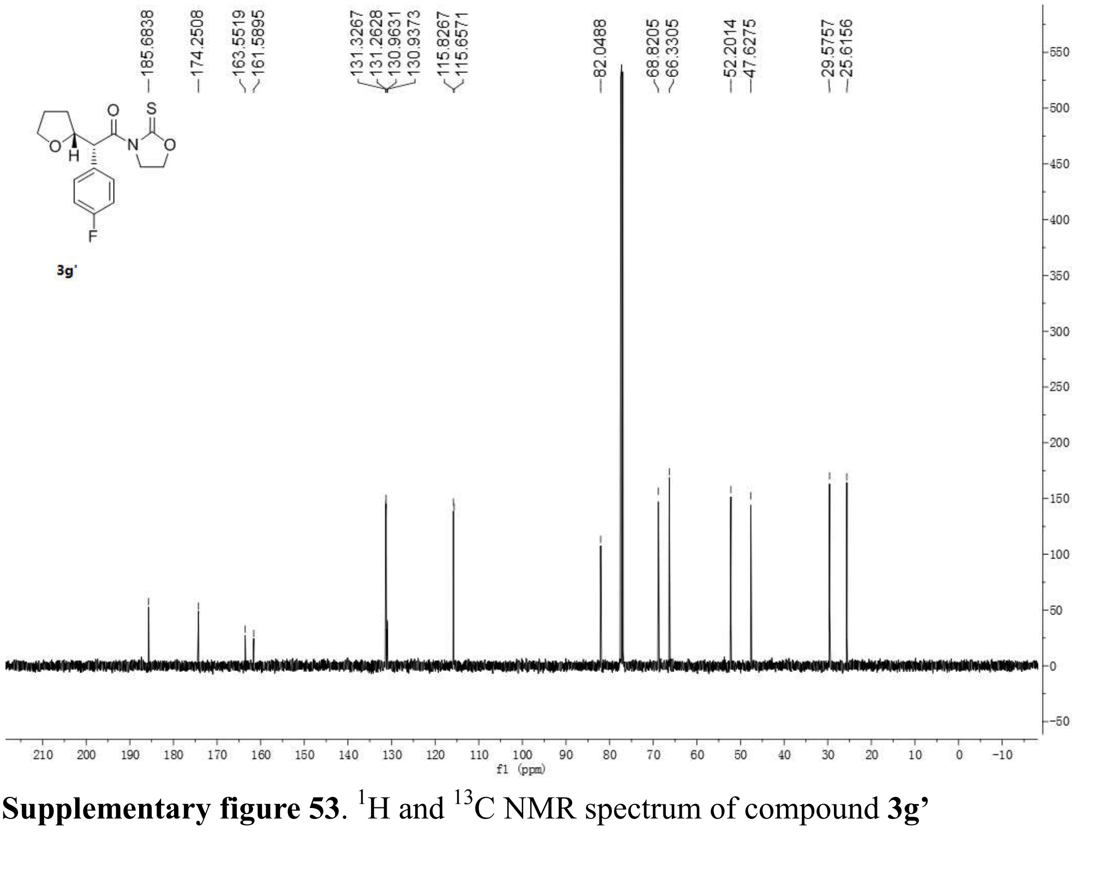

Supplementary figure 53. ^{1}H and ^{13}C NMR spectrum of compound **3g’**

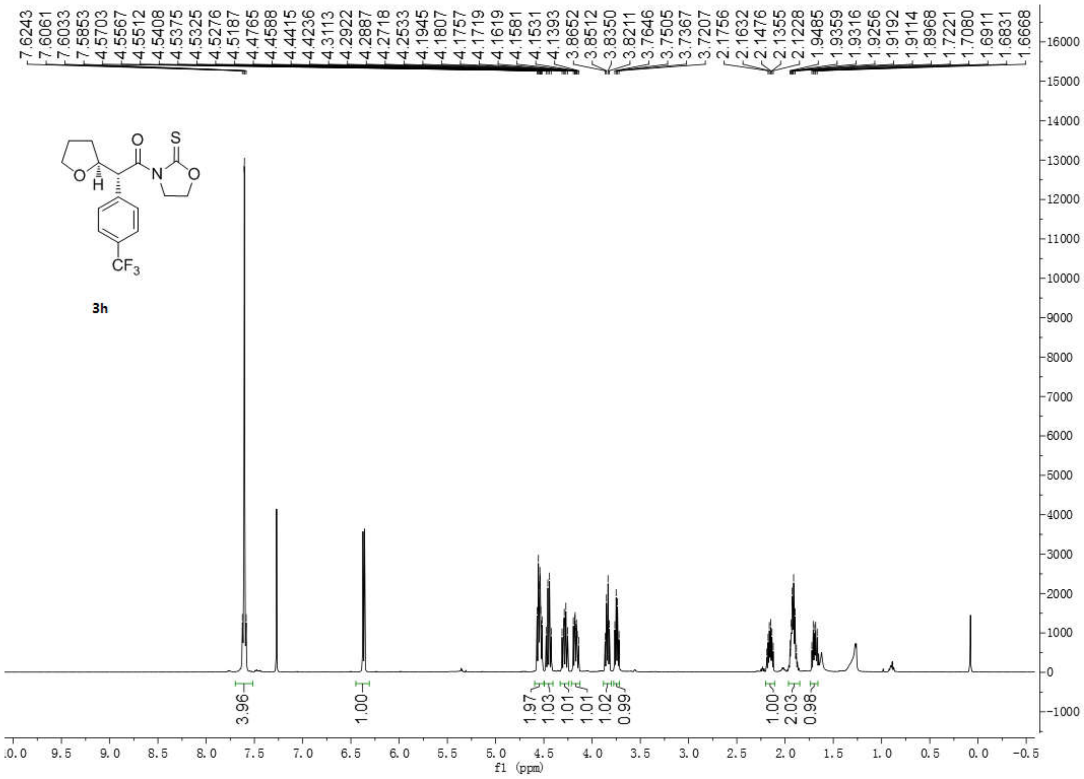

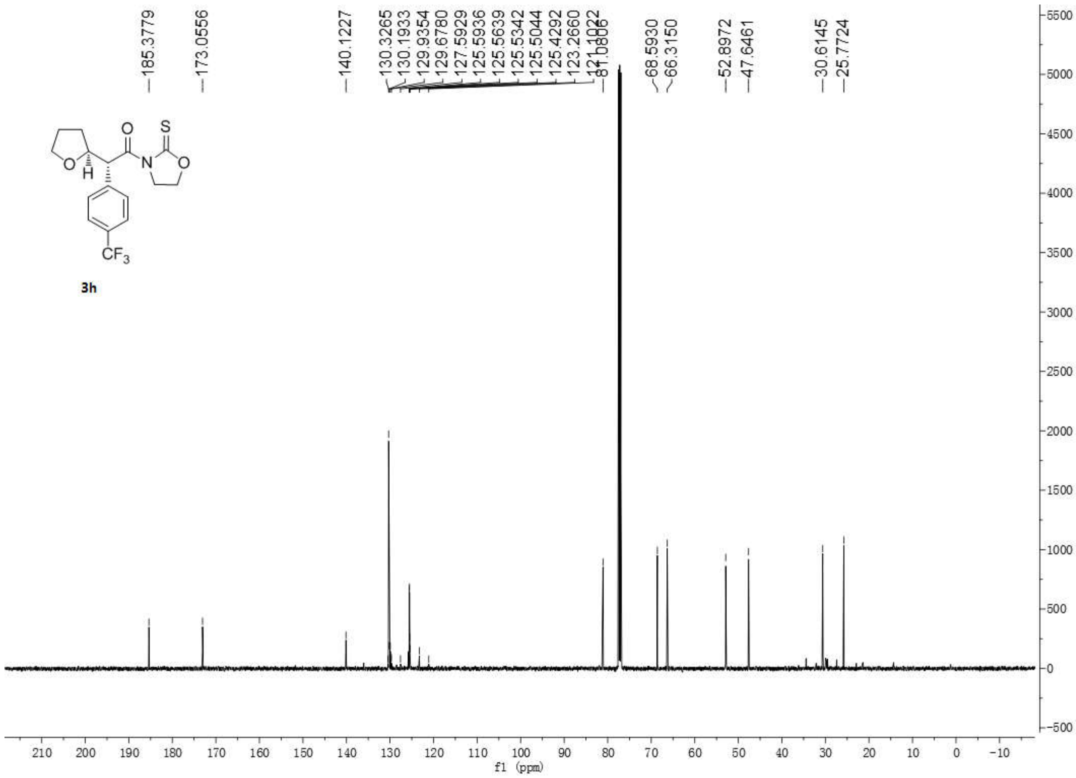

Supplementary figure 54. ^{1}H and ^{13}C NMR spectrum of compound **3h**

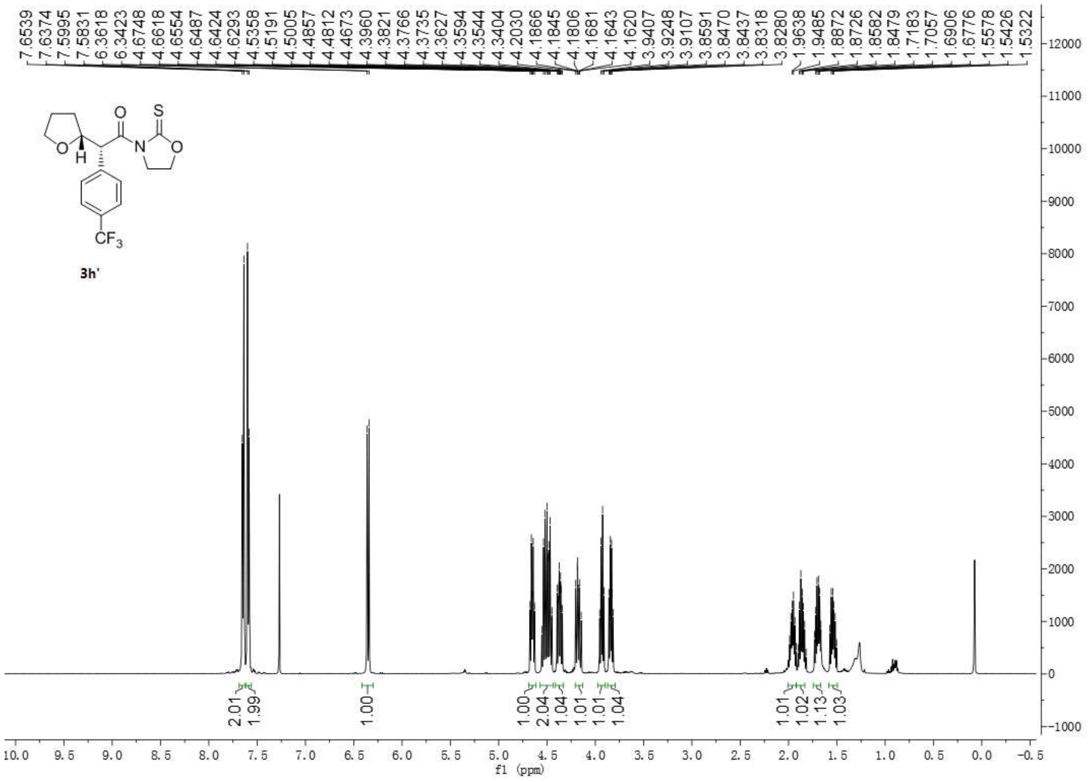

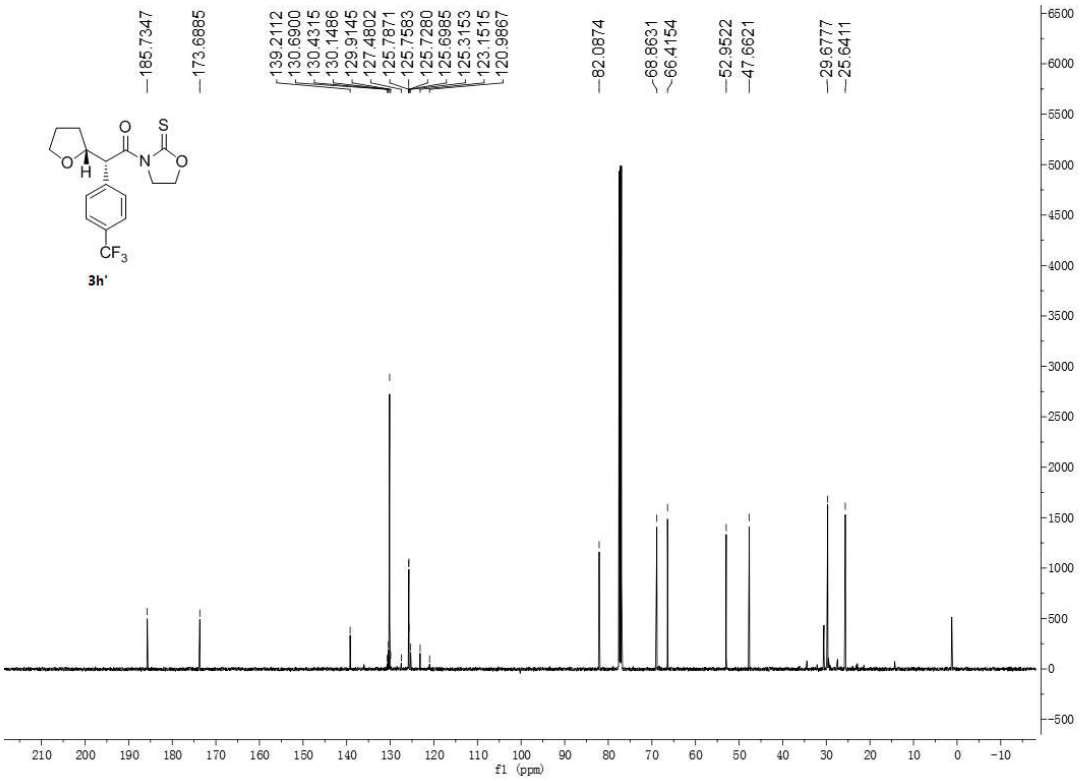

Supplementary figure 55. ^{1}H and ^{13}C NMR spectrum of compound **3h'**

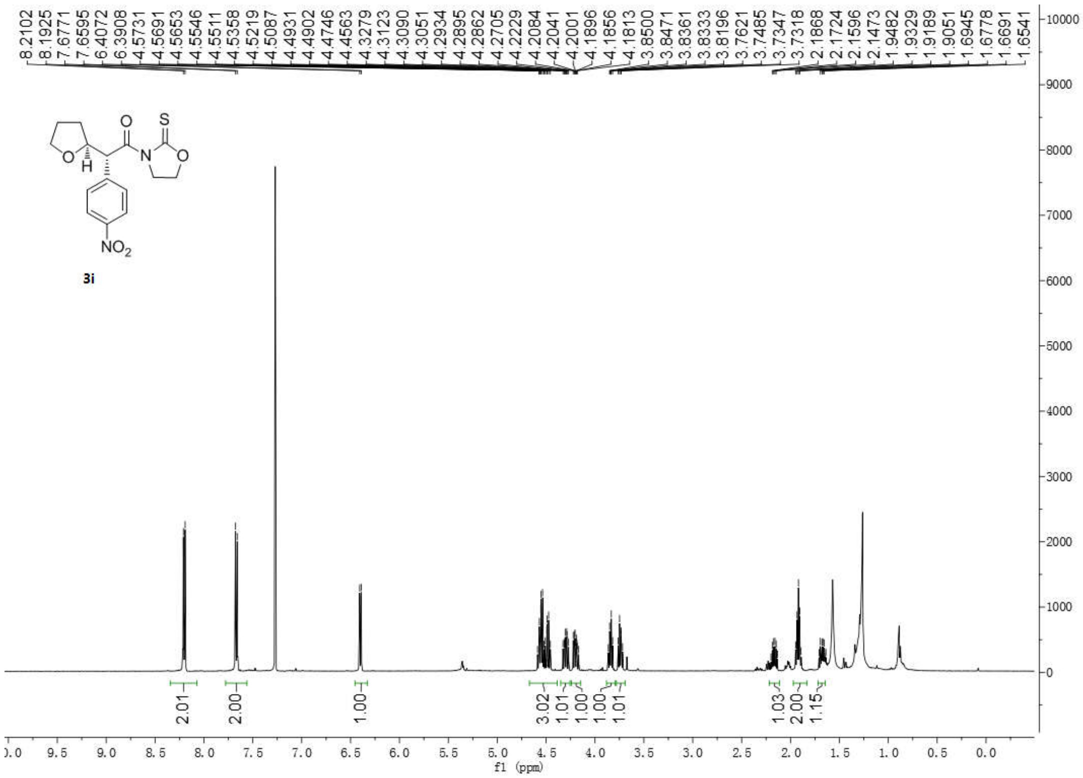

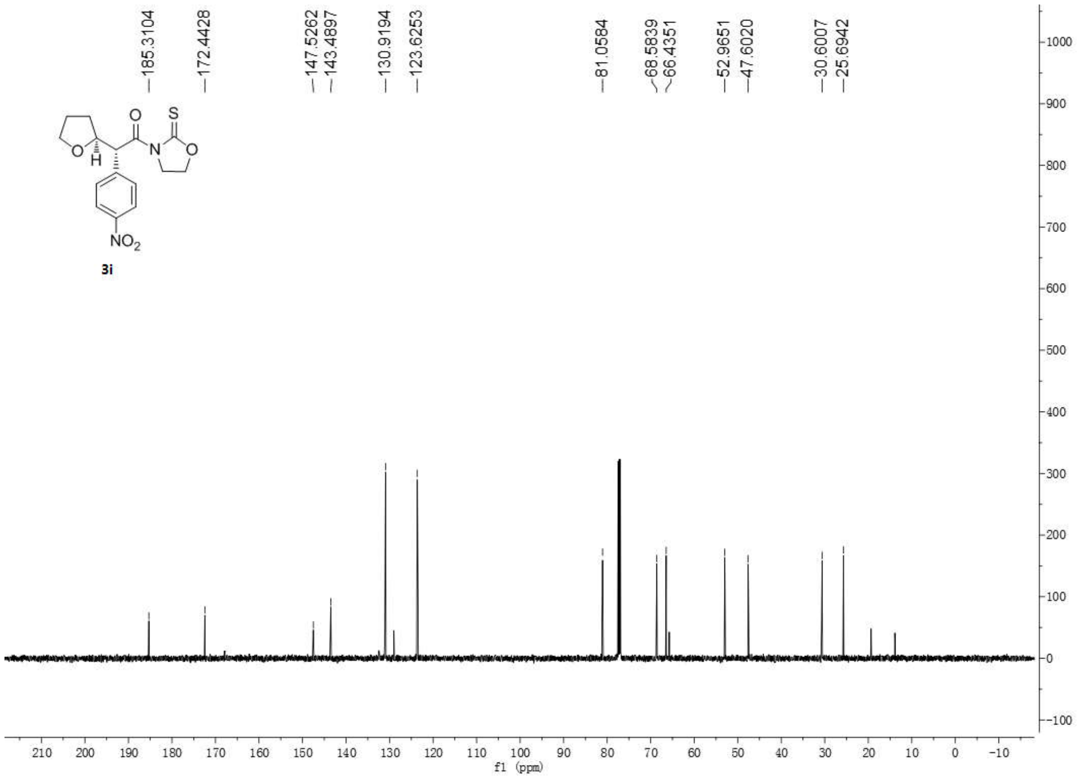

Supplementary figure 56. ^{1}H and ^{13}C NMR spectrum of compound **3i**

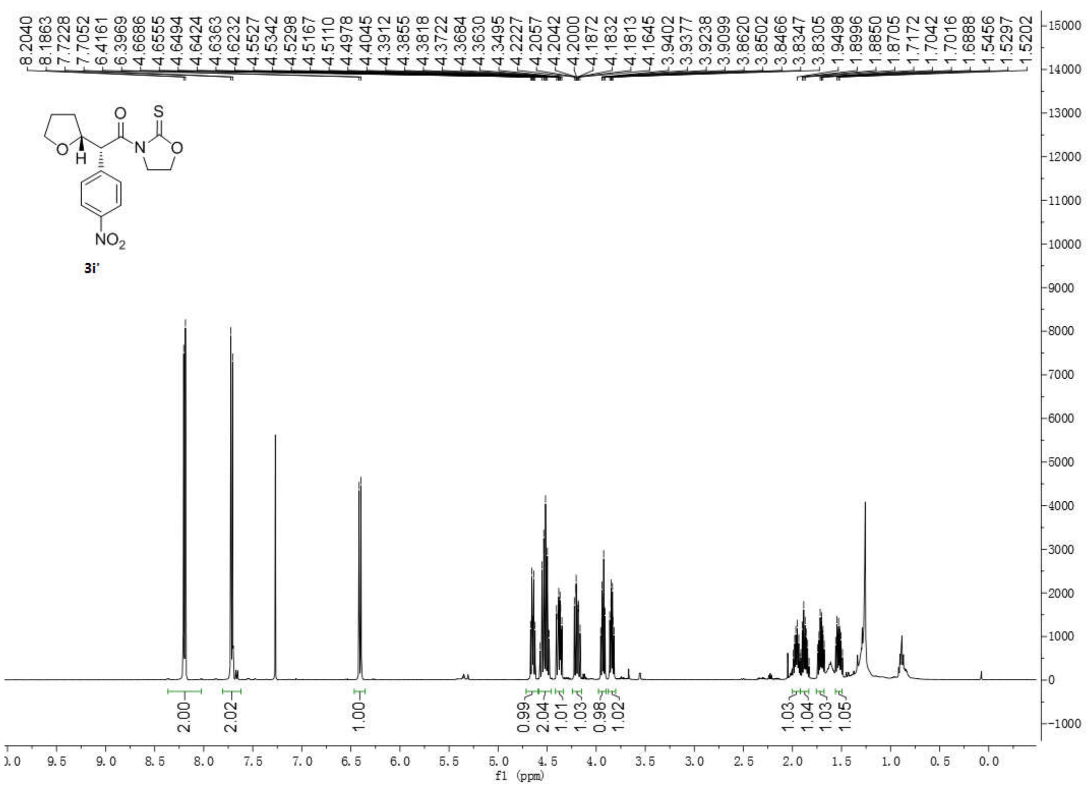

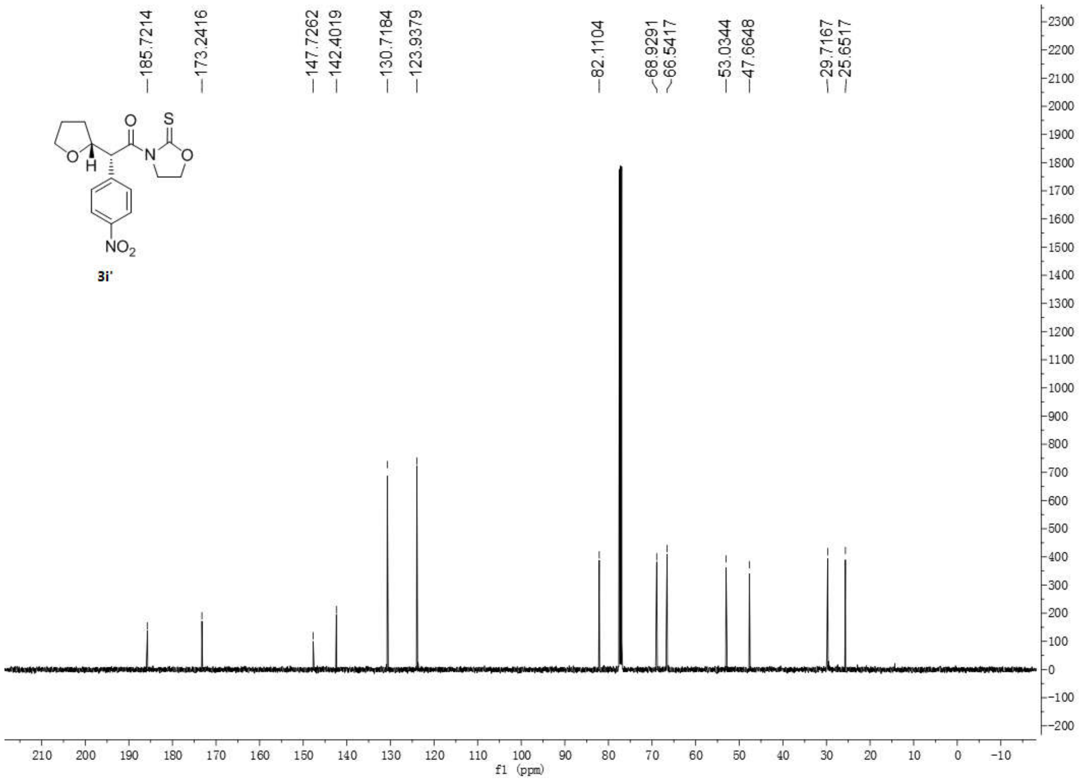

Supplementary figure 57. ^{1}H and ^{13}C NMR spectrum of compound **3i’**

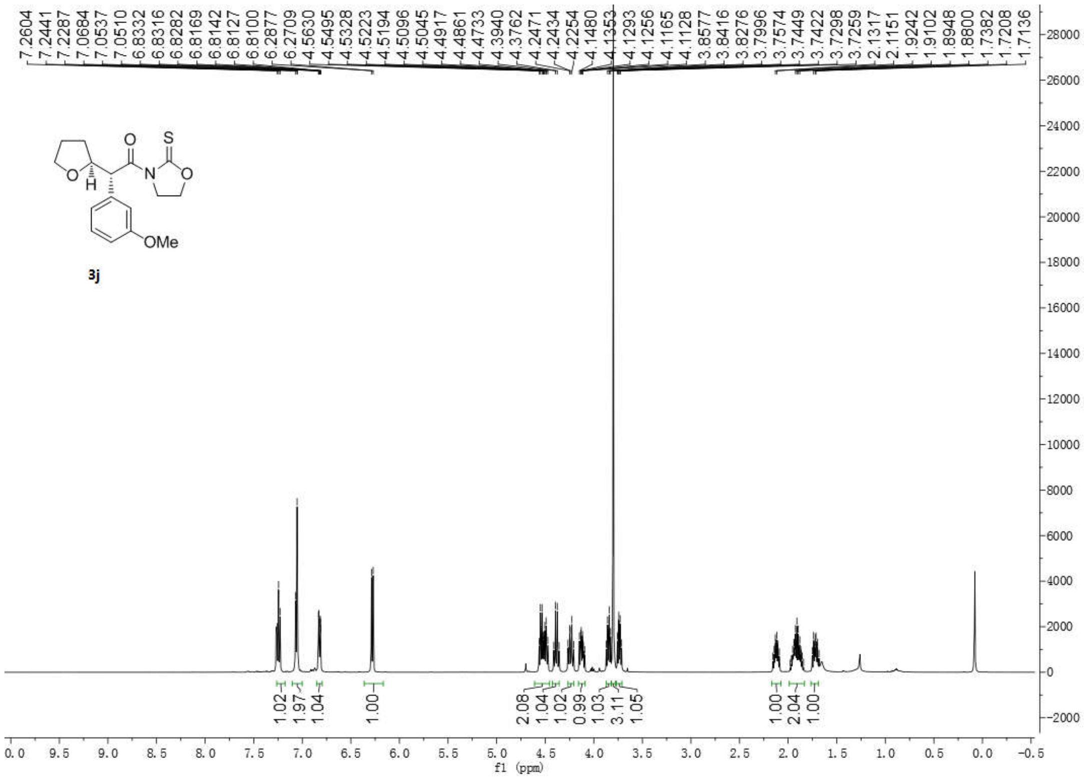

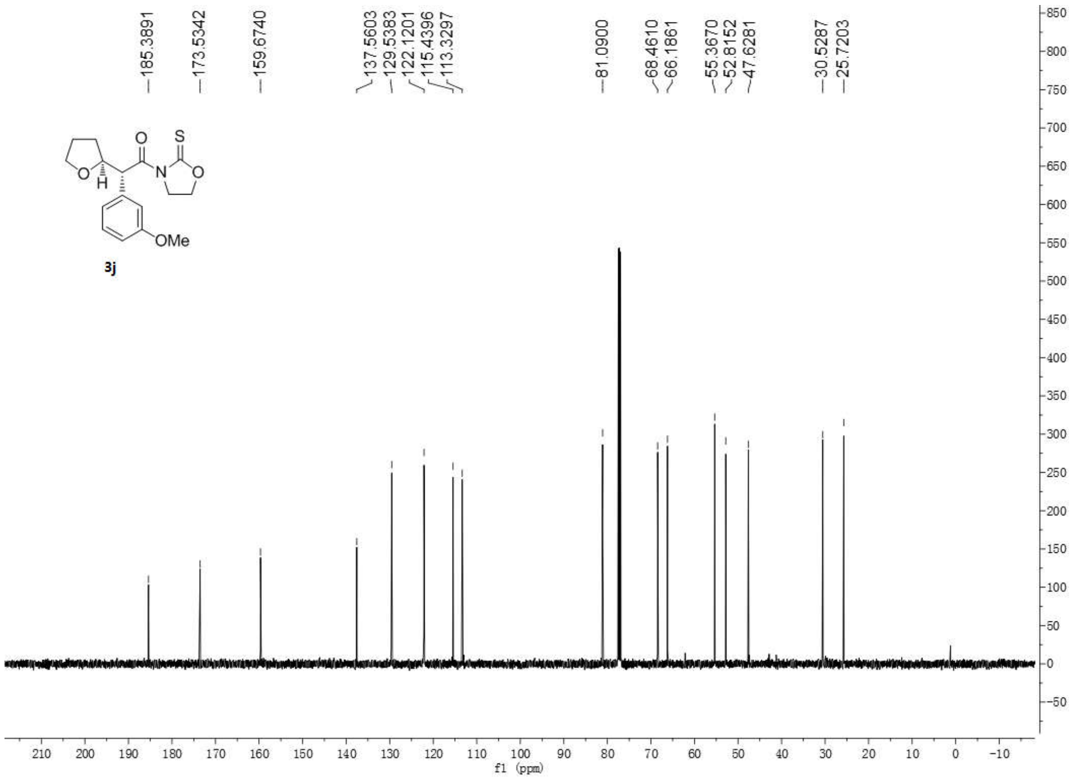

Supplementary figure 58. ^{1}H and ^{13}C NMR spectrum of compound **3j**

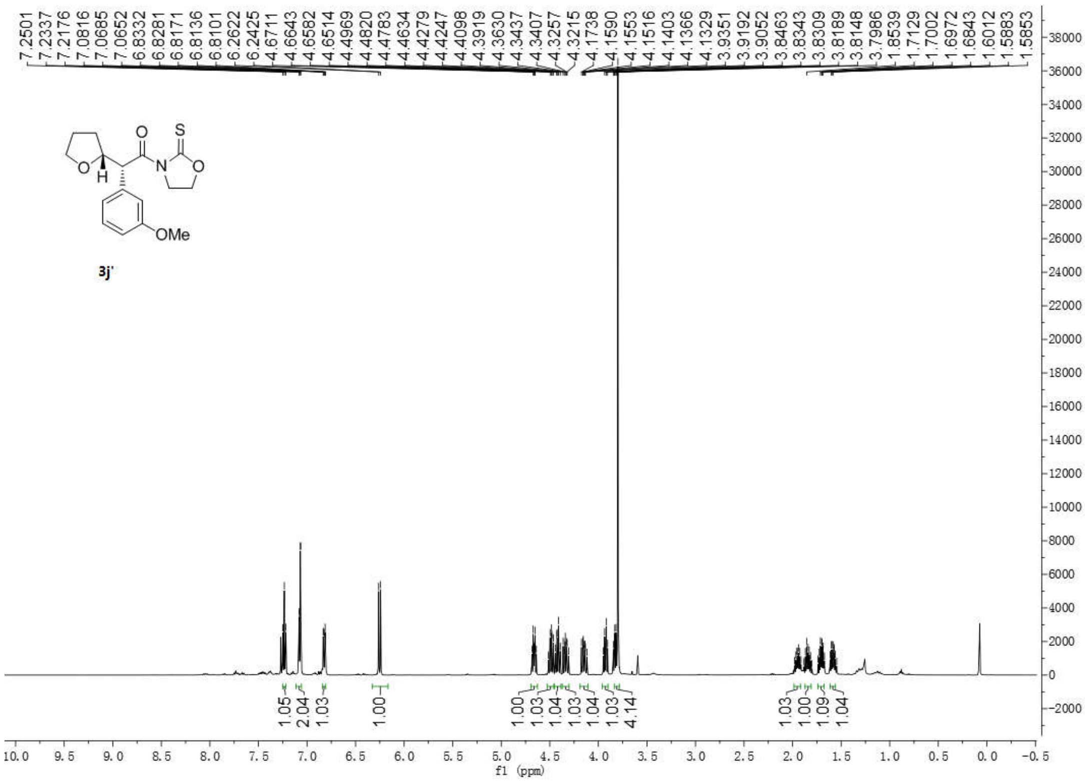

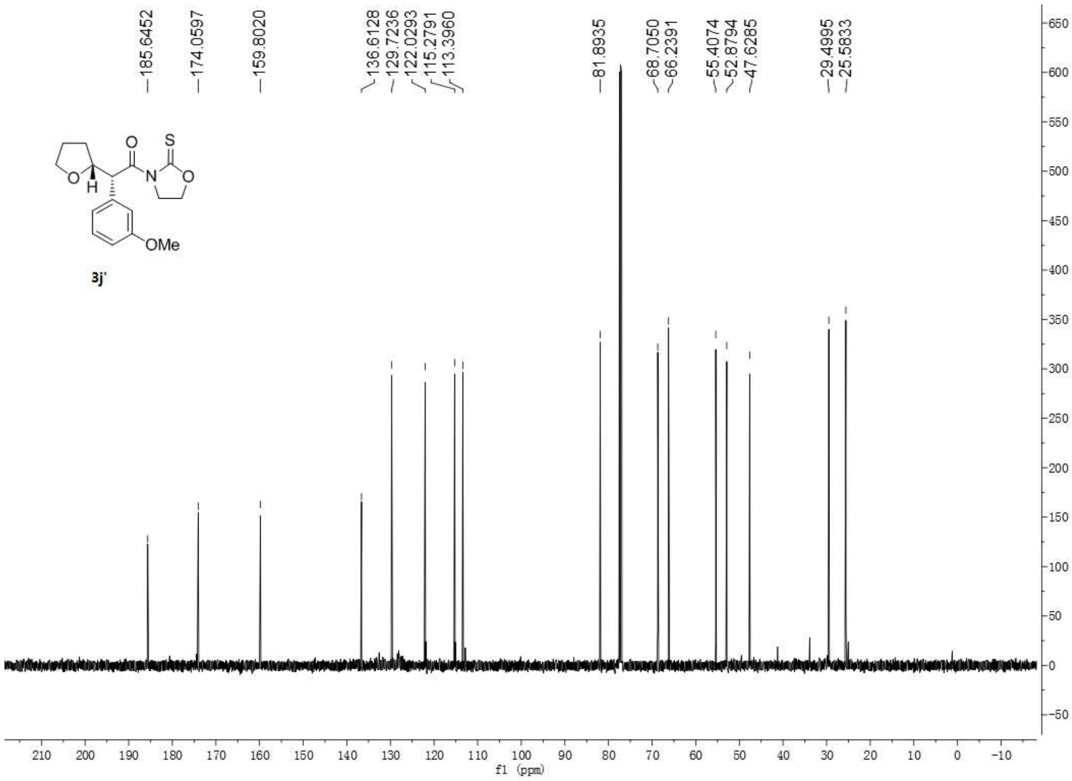

Supplementary figure 59. ^{1}H and ^{13}C NMR spectrum of compound **3j'**

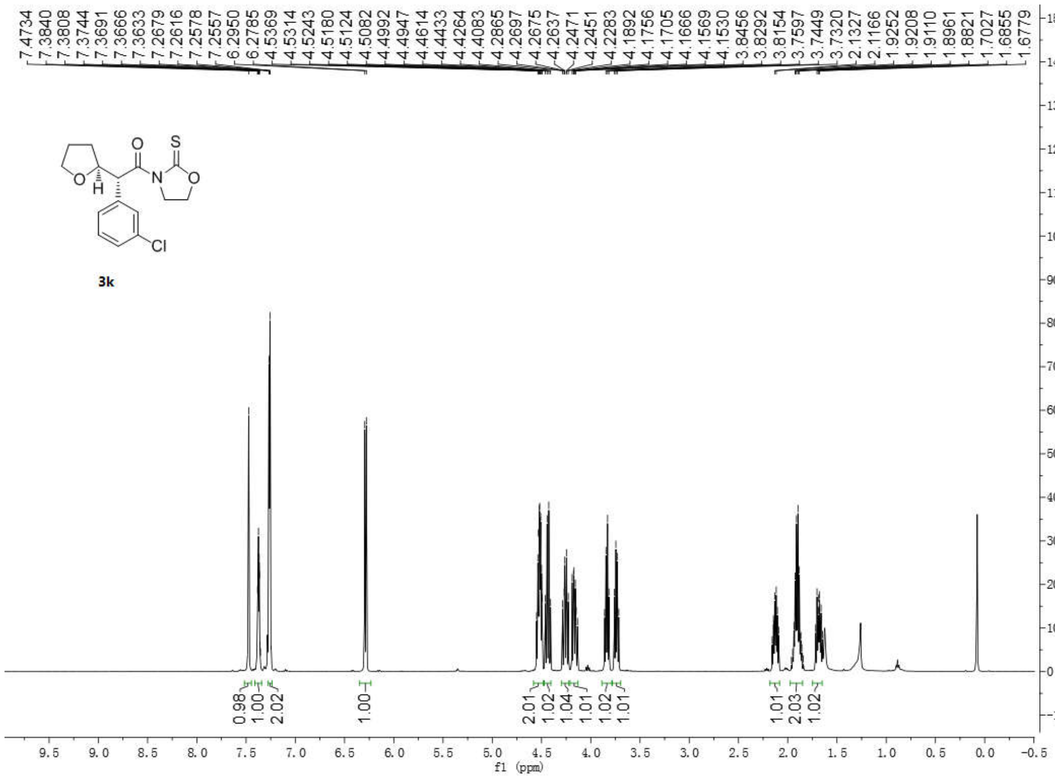

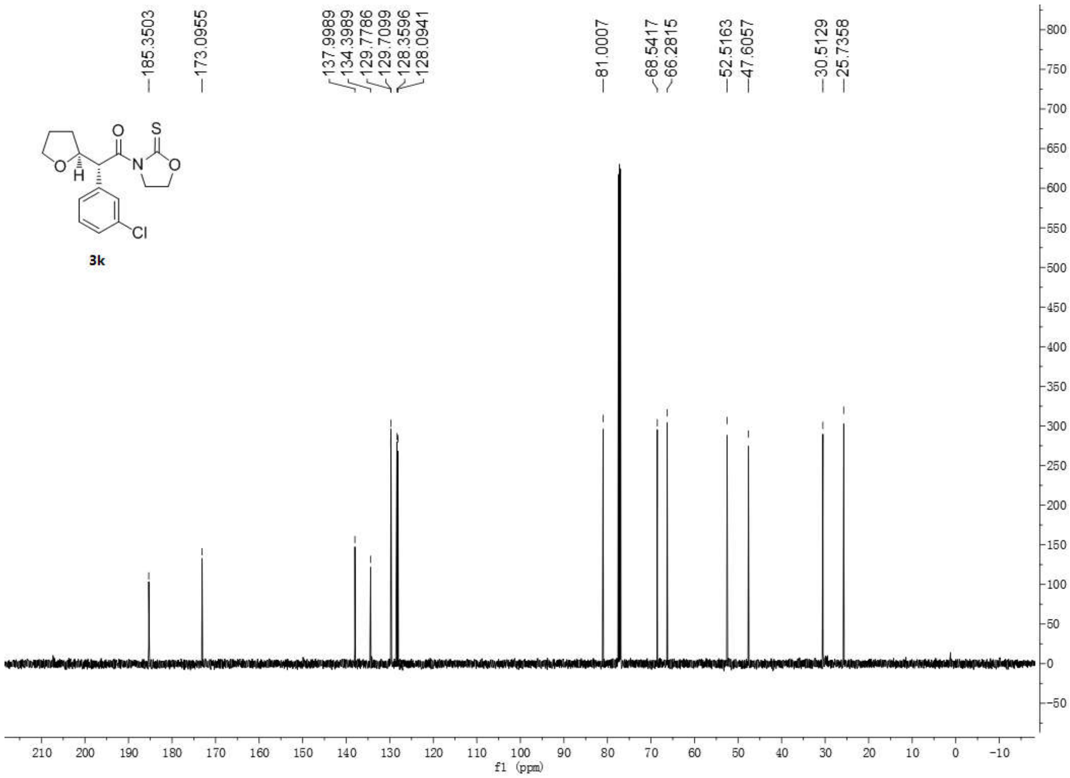

Supplementary figure 60. ^{1}H and ^{13}C NMR spectrum of compound **3k**

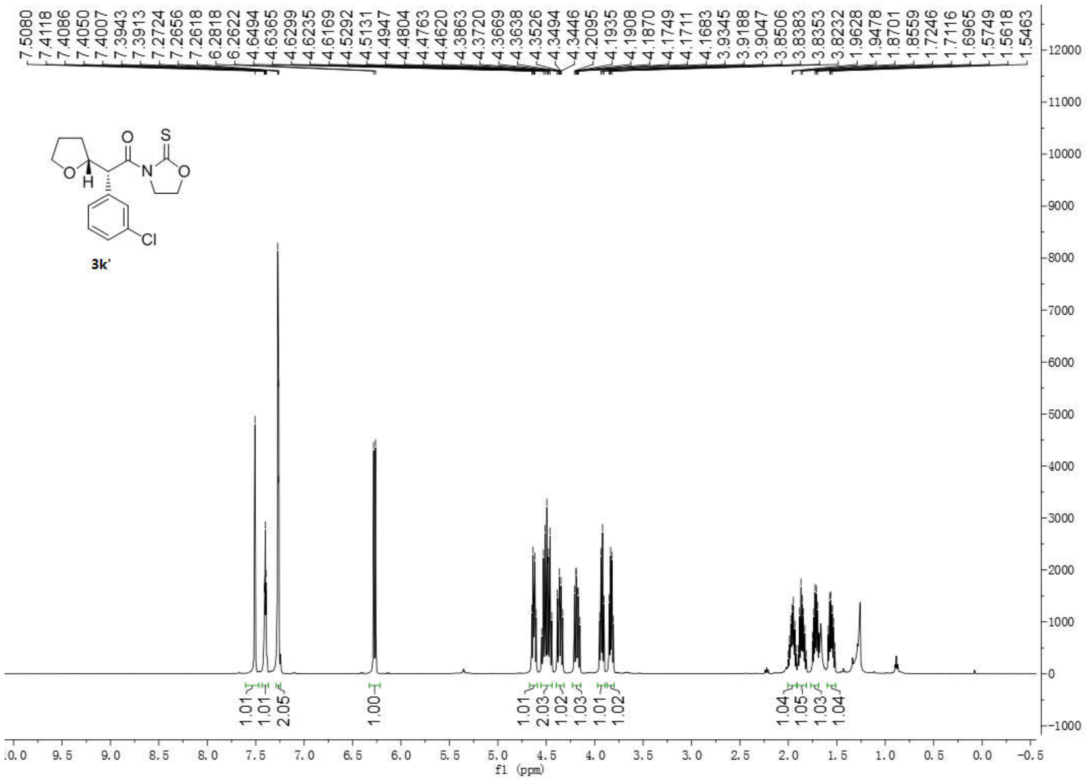

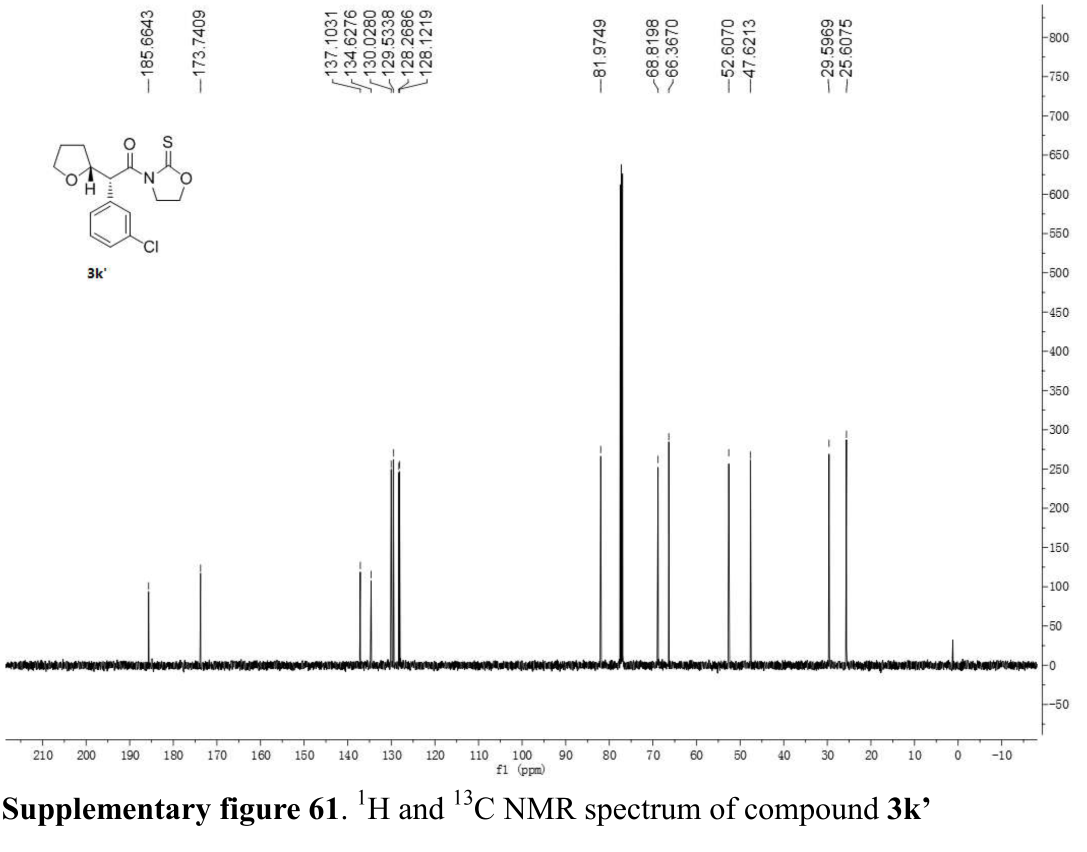

Supplementary figure 61. ^{1}H and ^{13}C NMR spectrum of compound **3k'**

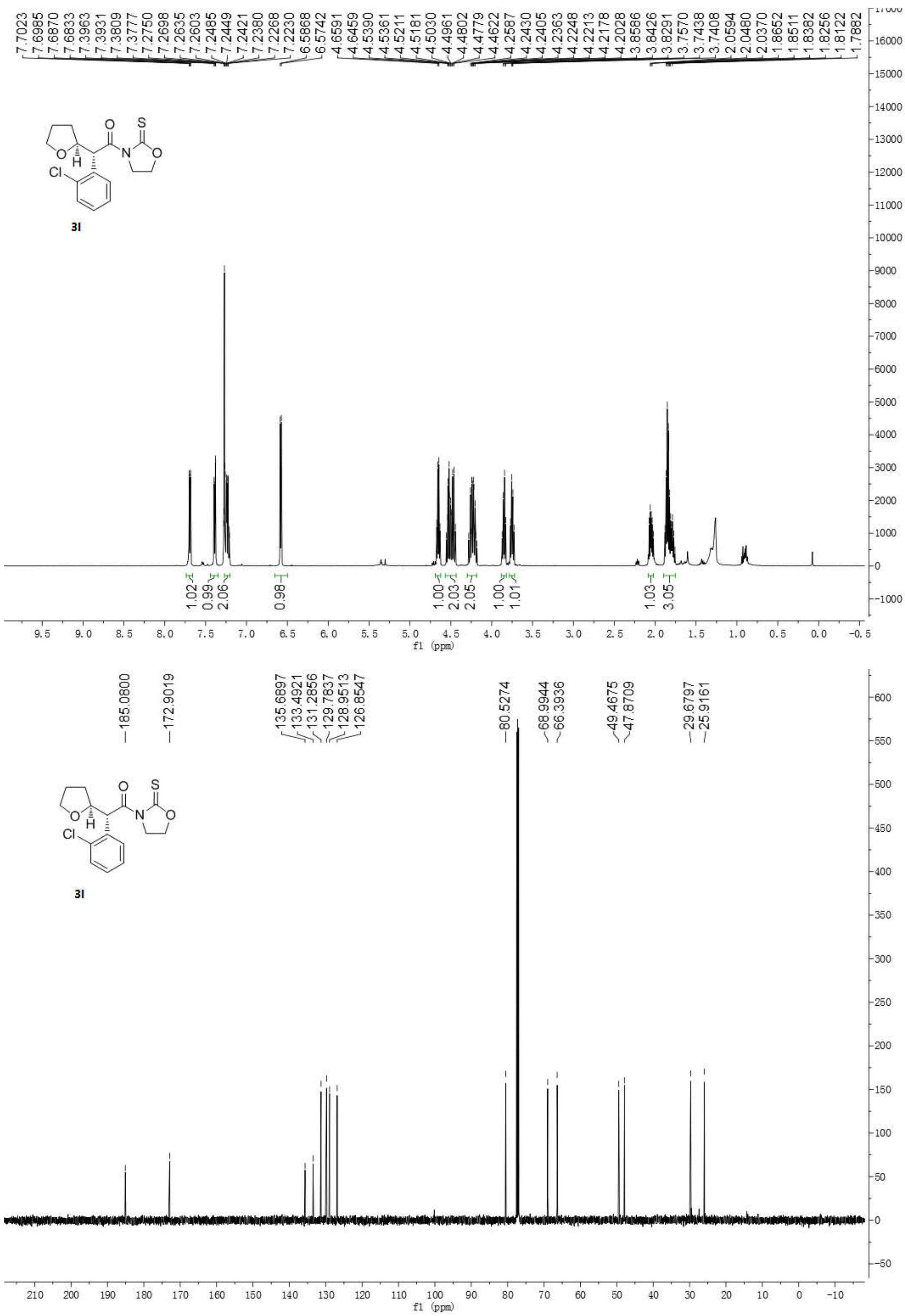

Supplementary figure 62. ^{1}H and ^{13}C NMR spectrum of compound **3l**

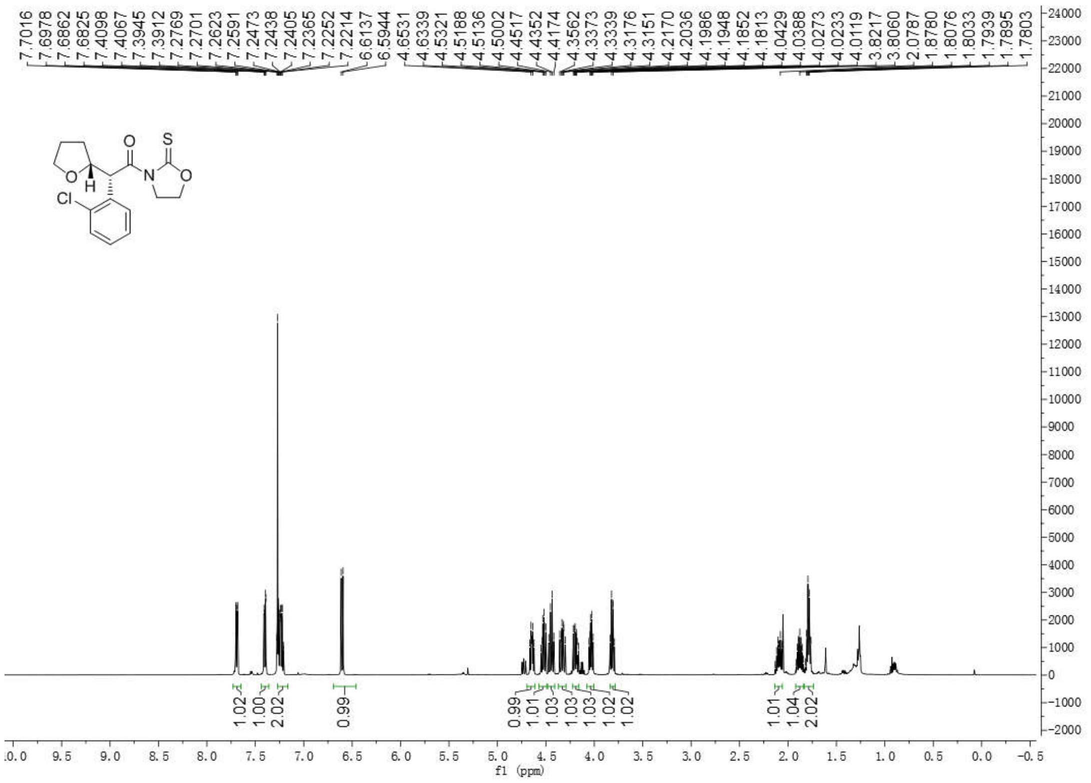

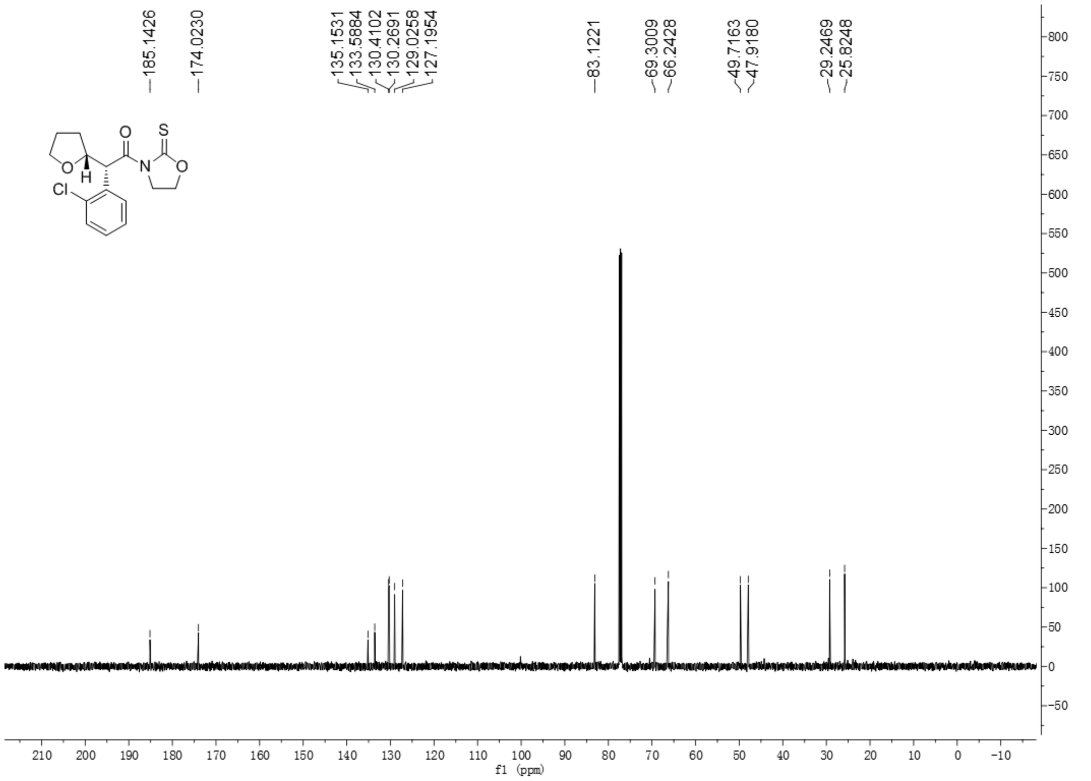

Supplementary figure 63. ^{1}H and ^{13}C NMR spectrum of compound **3l’**

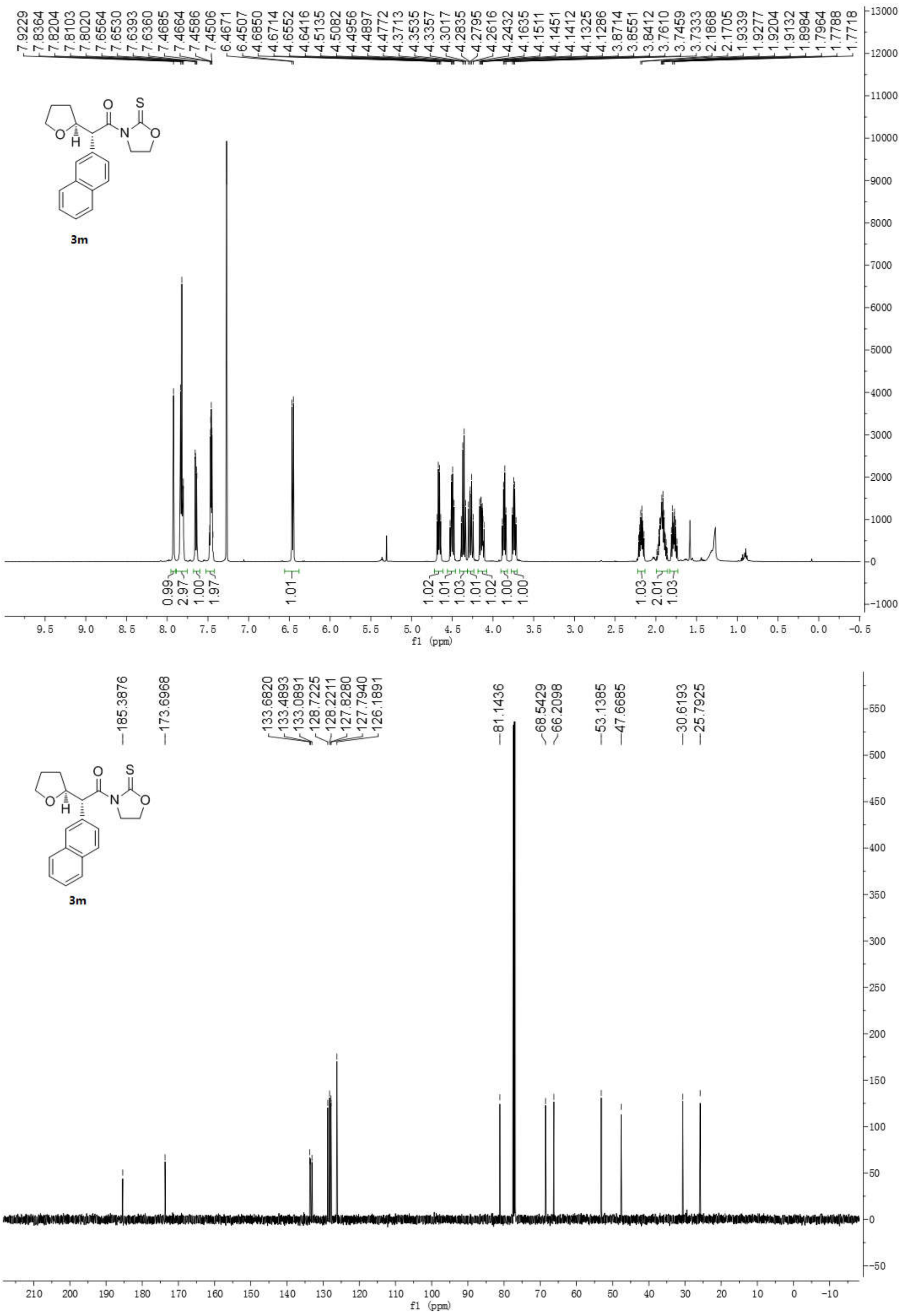

Supplementary figure 64. ^{1}H and ^{13}C NMR spectrum of compound **3m**

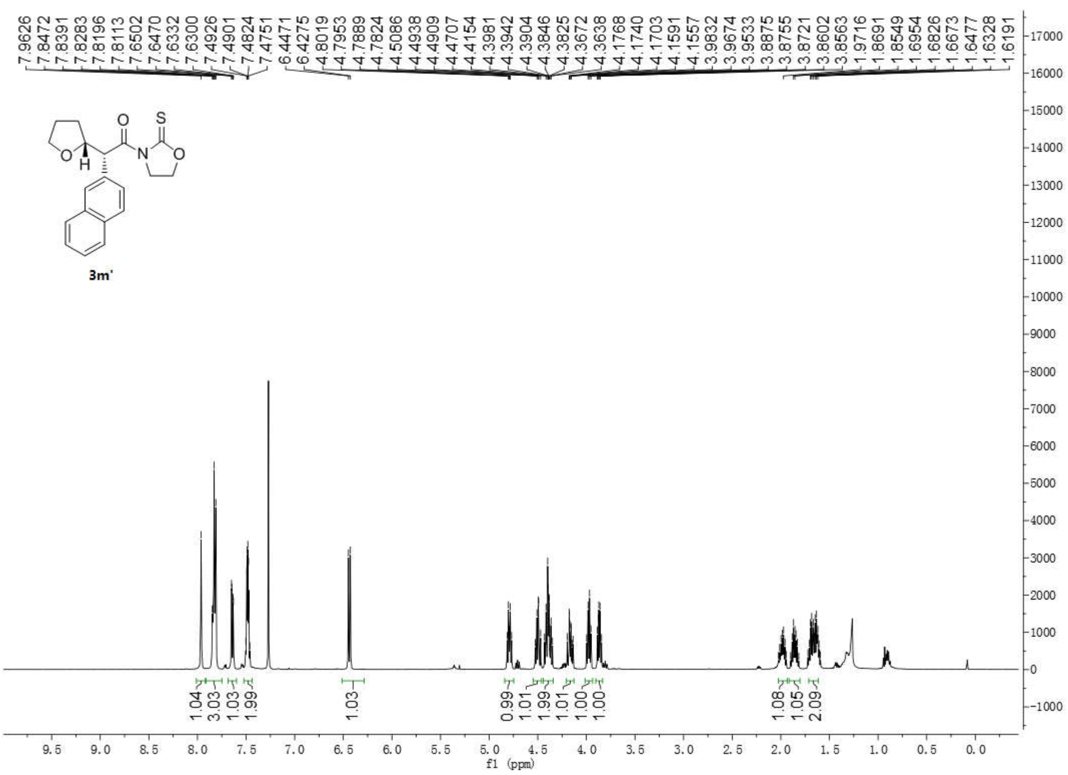

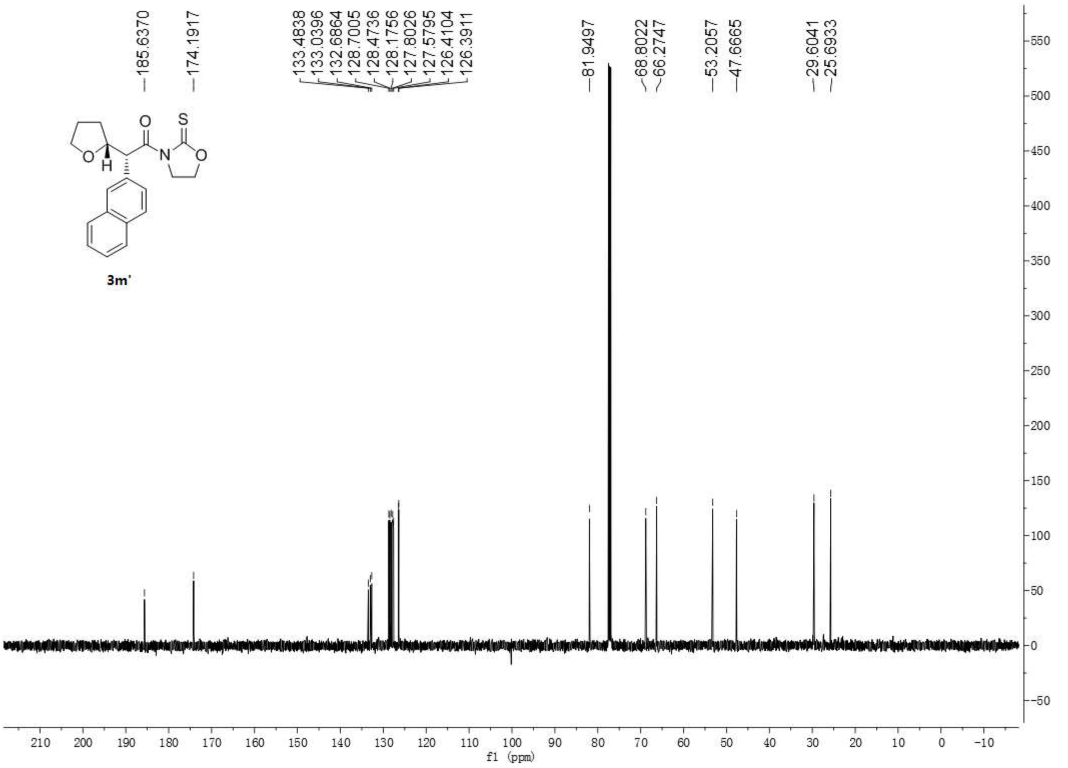

Supplementary figure 65. ^{1}H and ^{13}C NMR spectrum of compound **3m’**

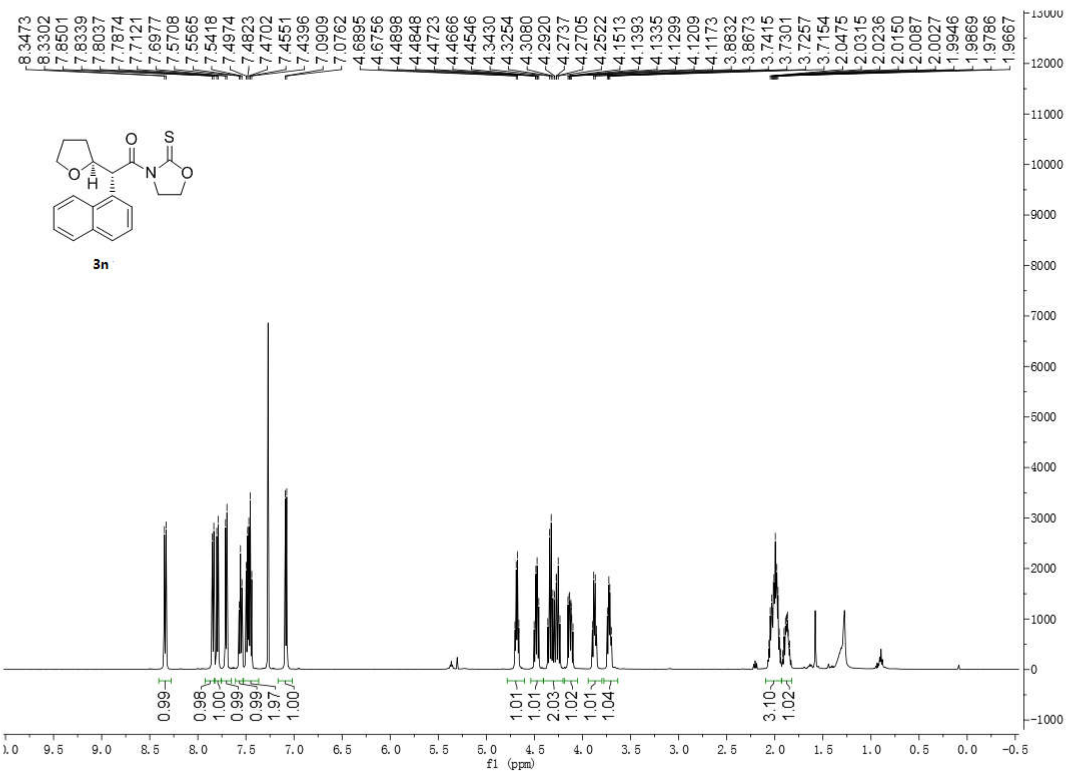

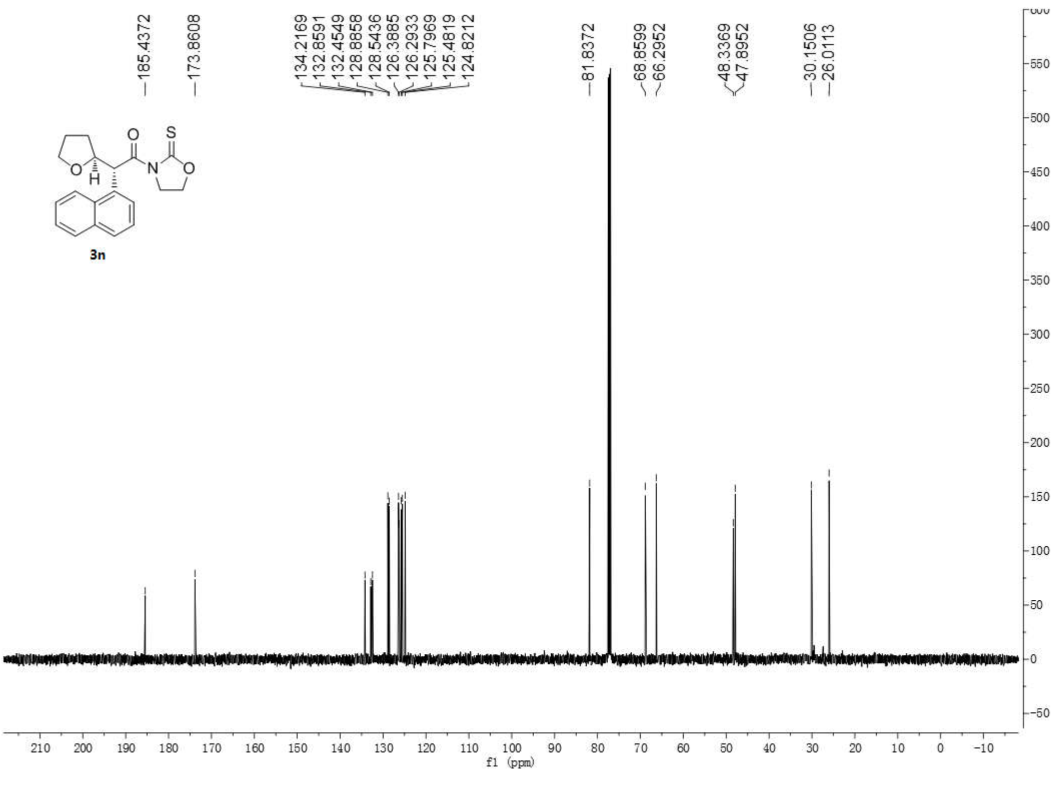

Supplementary figure 66. ^{1}H and ^{13}C NMR spectrum of compound **3n**

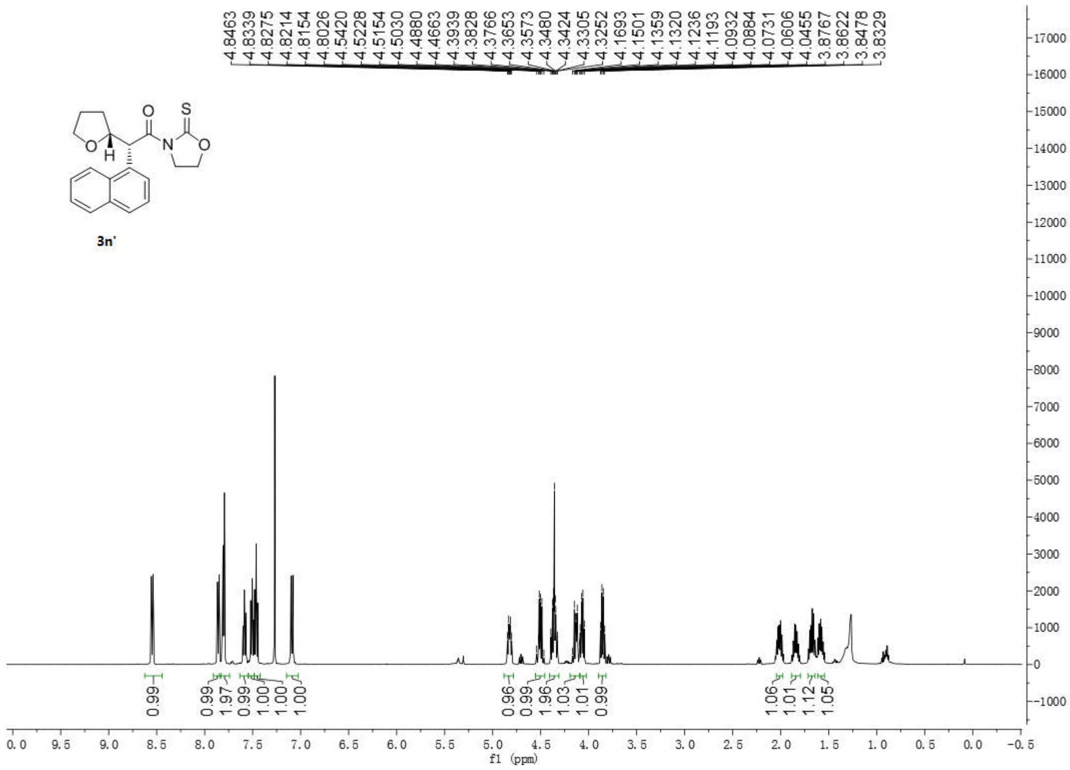

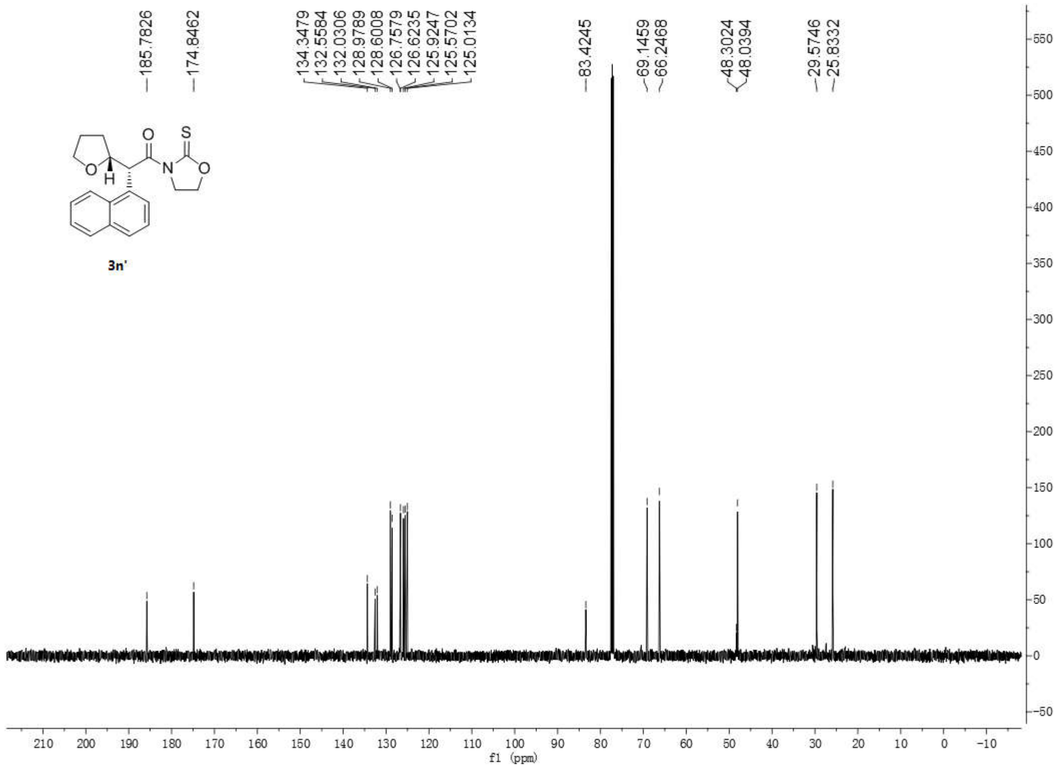

Supplementary figure 67. ^{1}H and ^{13}C NMR spectrum of compound **3n'**

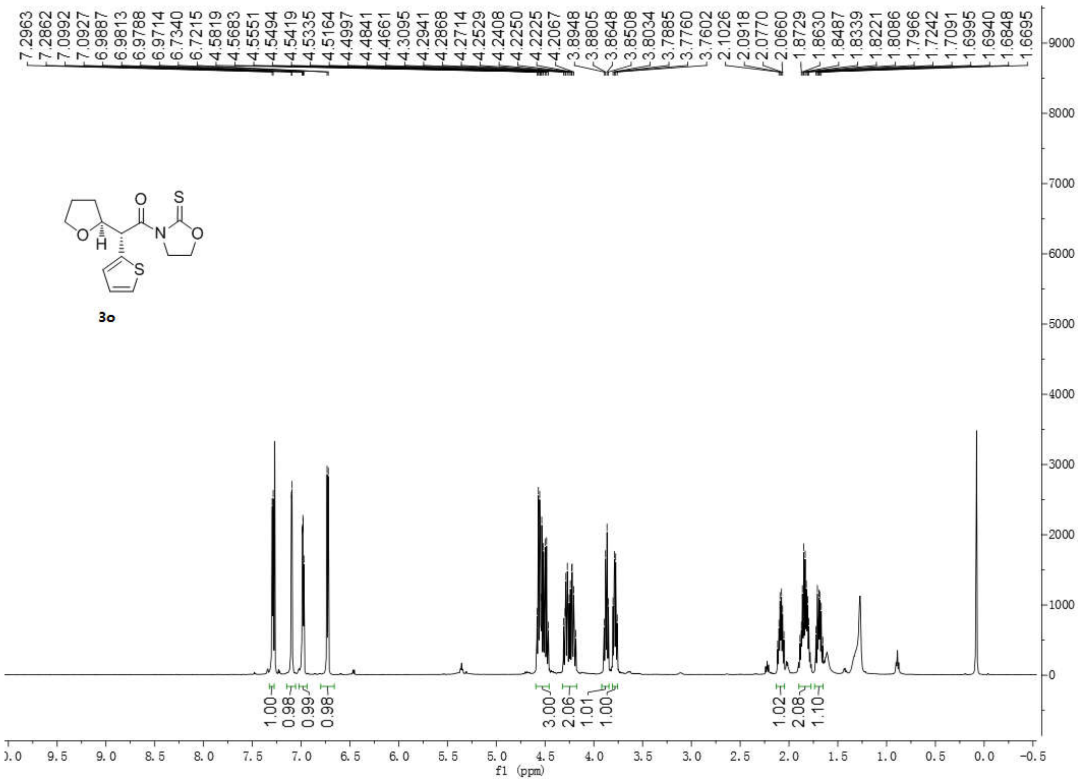

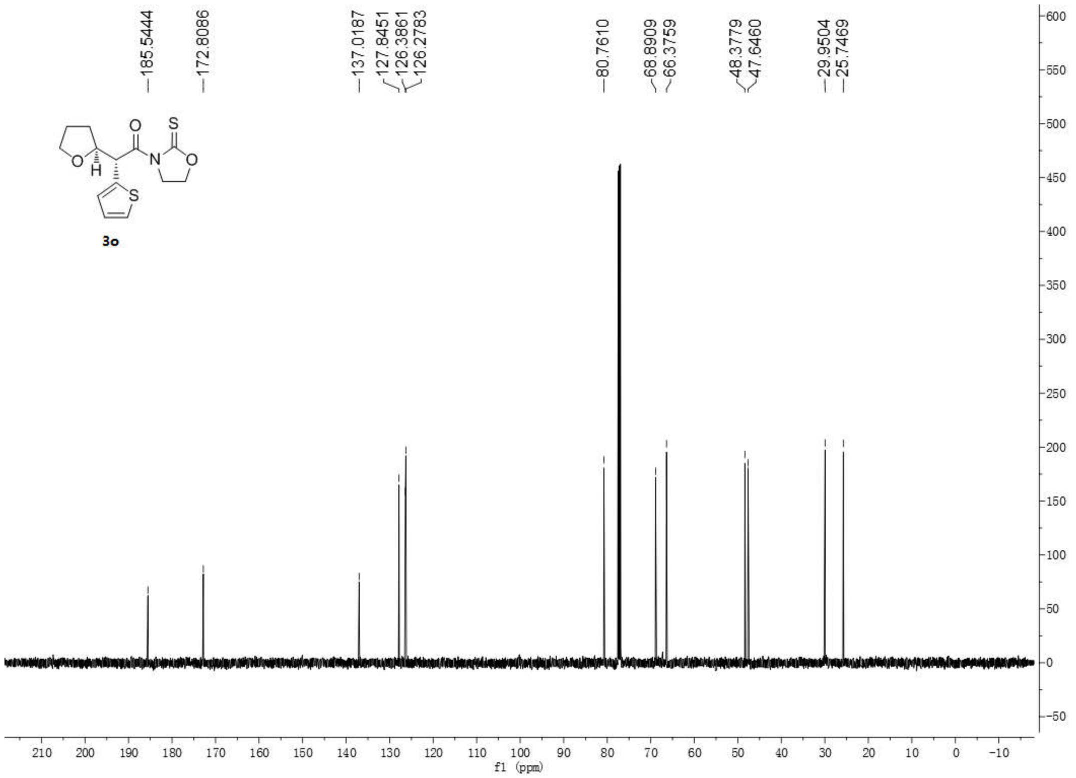

Supplementary figure 68. ^{1}H and ^{13}C NMR spectrum of compound **3o**

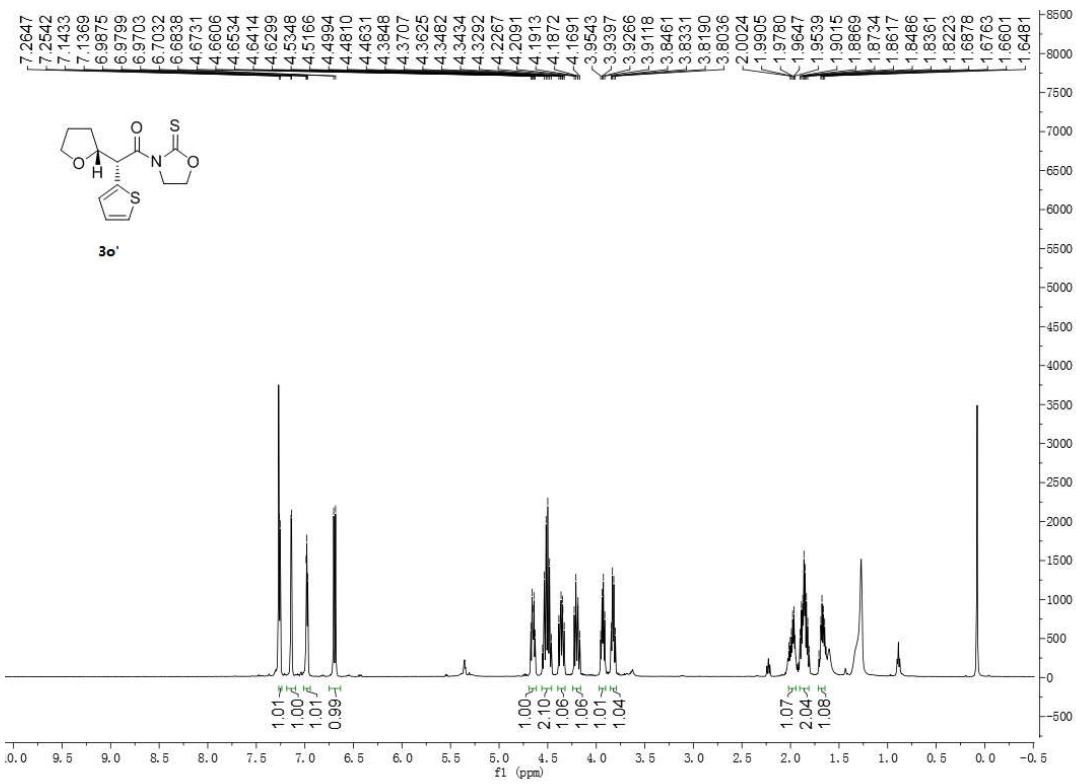

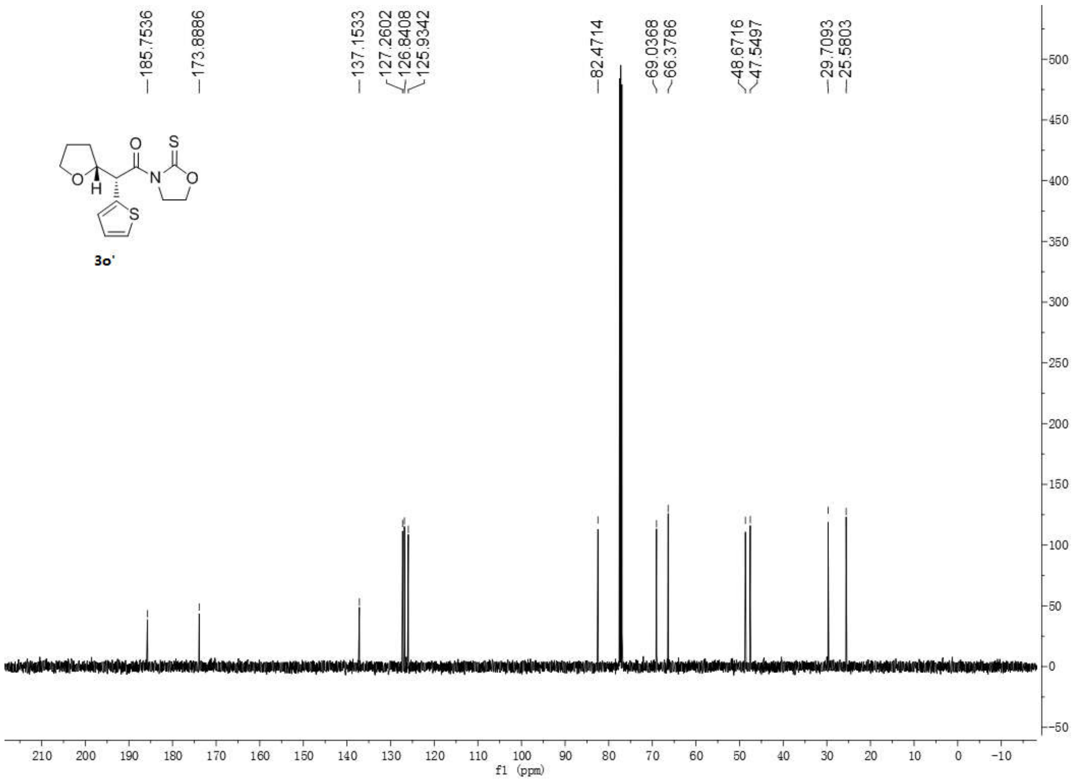

Supplementary figure 69. ^{1}H and ^{13}C NMR spectrum of compound **3o'**

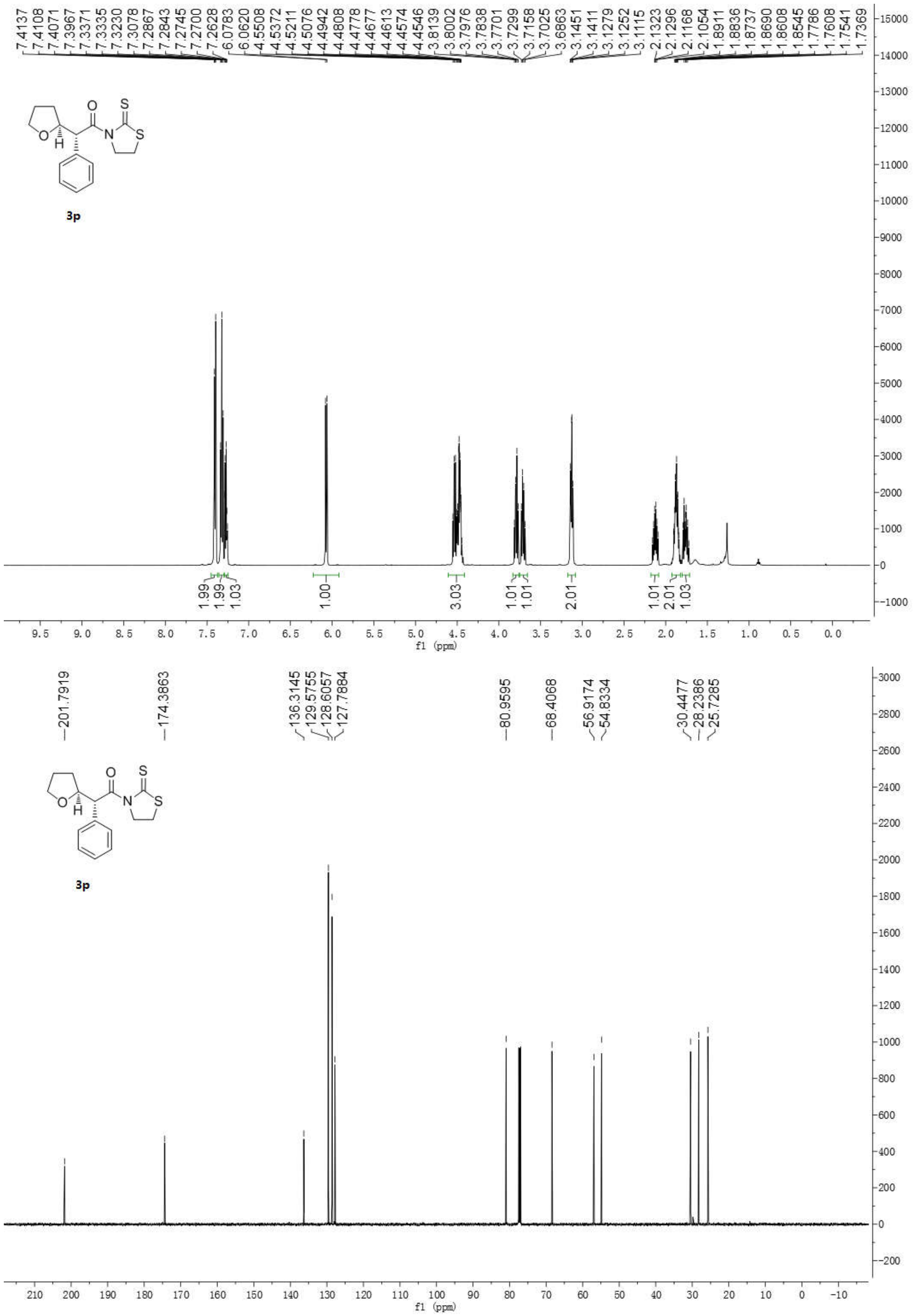

Supplementary figure 70. ^{1}H and ^{13}C NMR spectrum of compound **3p**

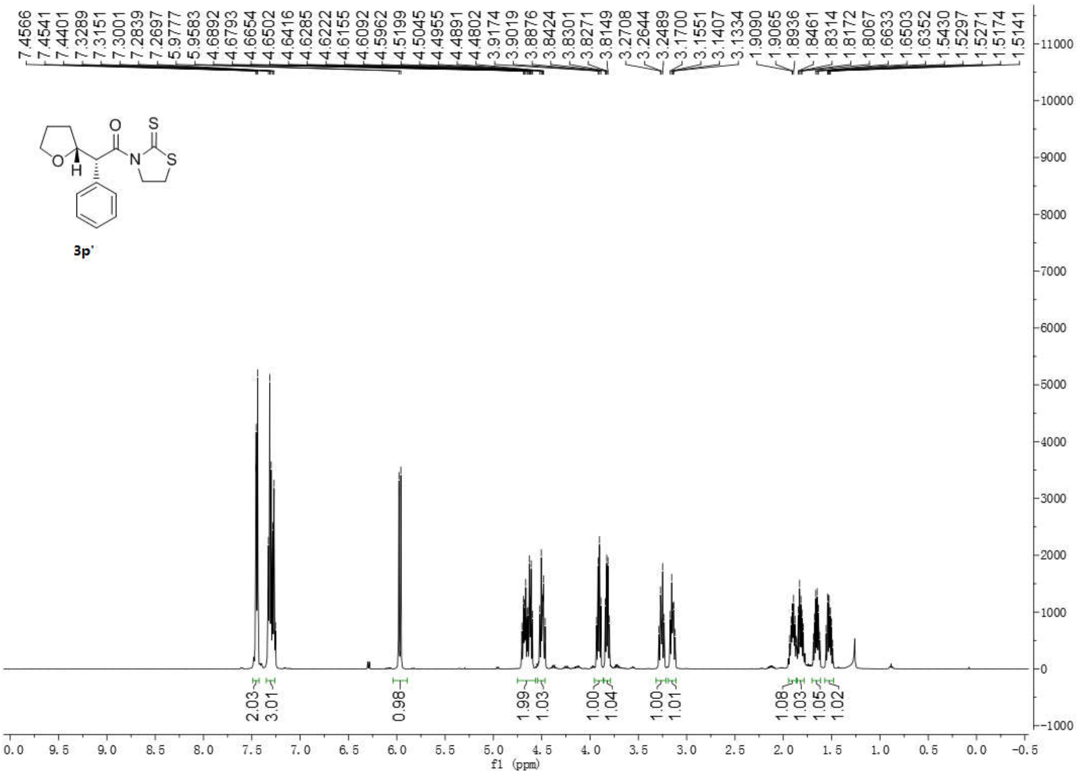

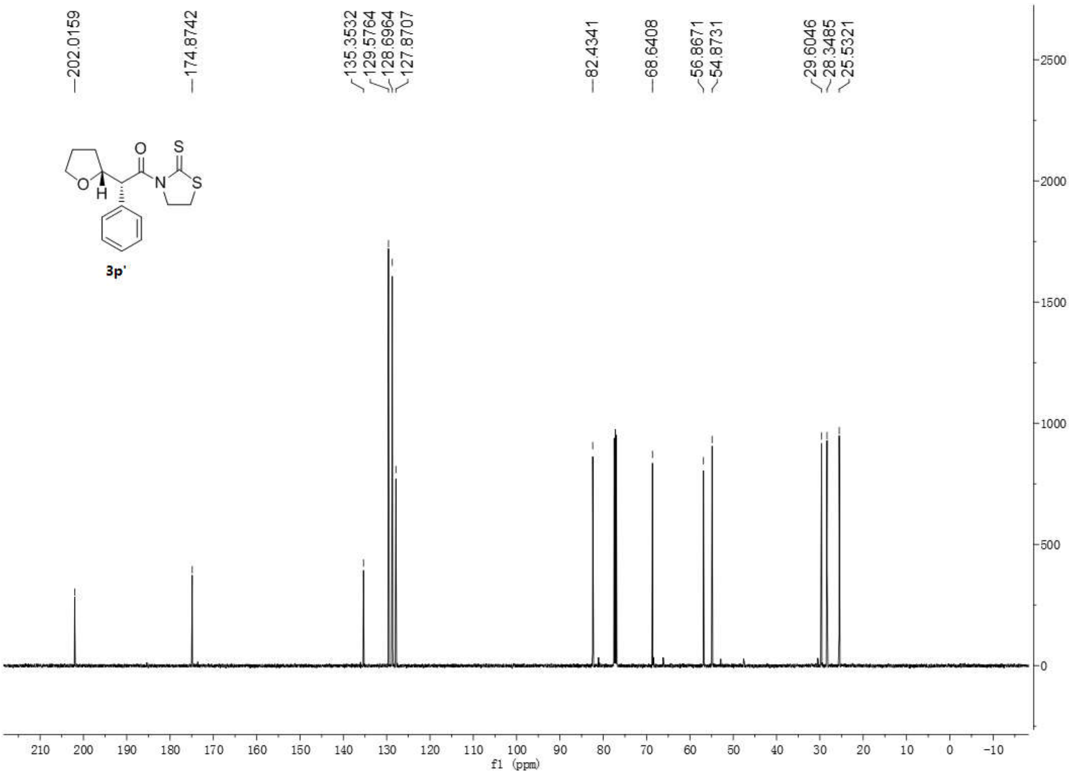

Supplementary figure 71. ^{1}H and ^{13}C NMR spectrum of compound **3p'**

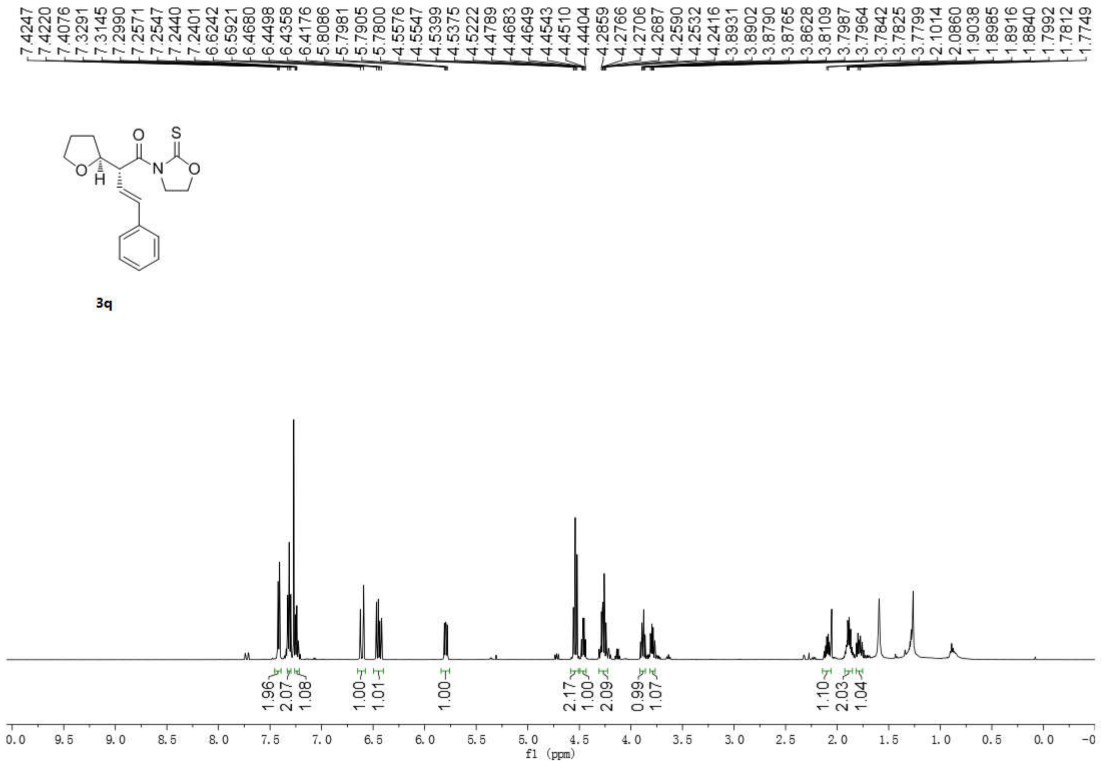

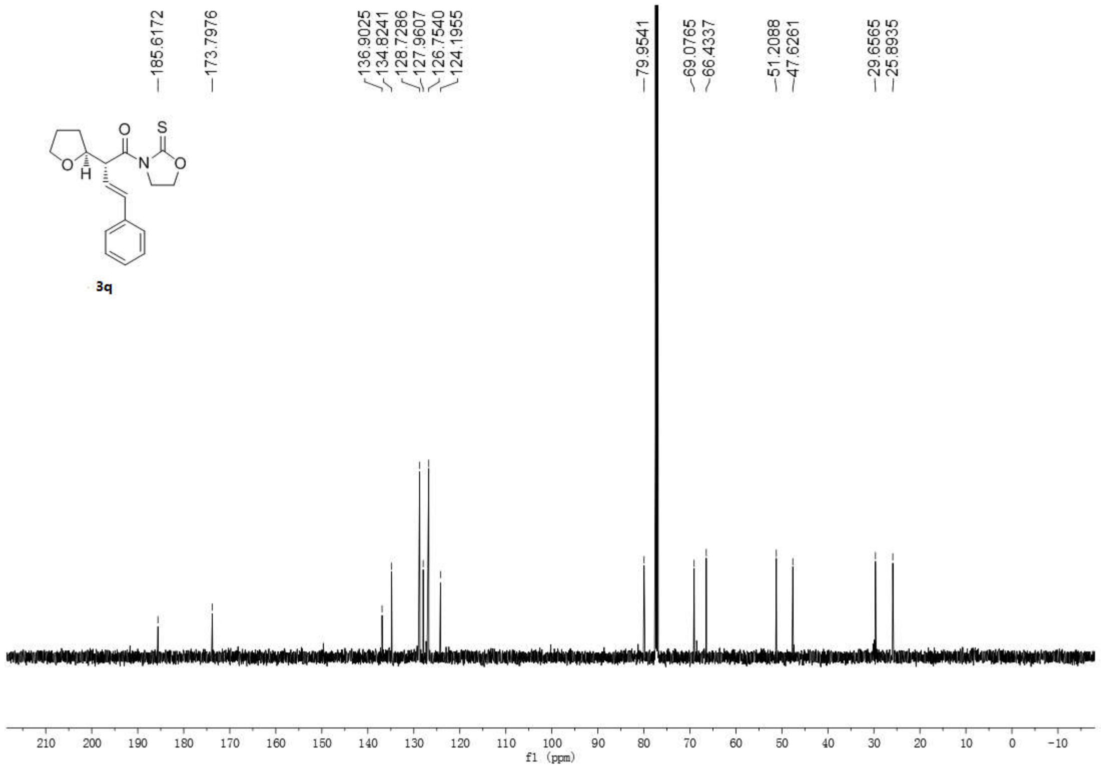

Supplementary figure 72. ^{1}H and ^{13}C NMR spectrum of compound **3q**

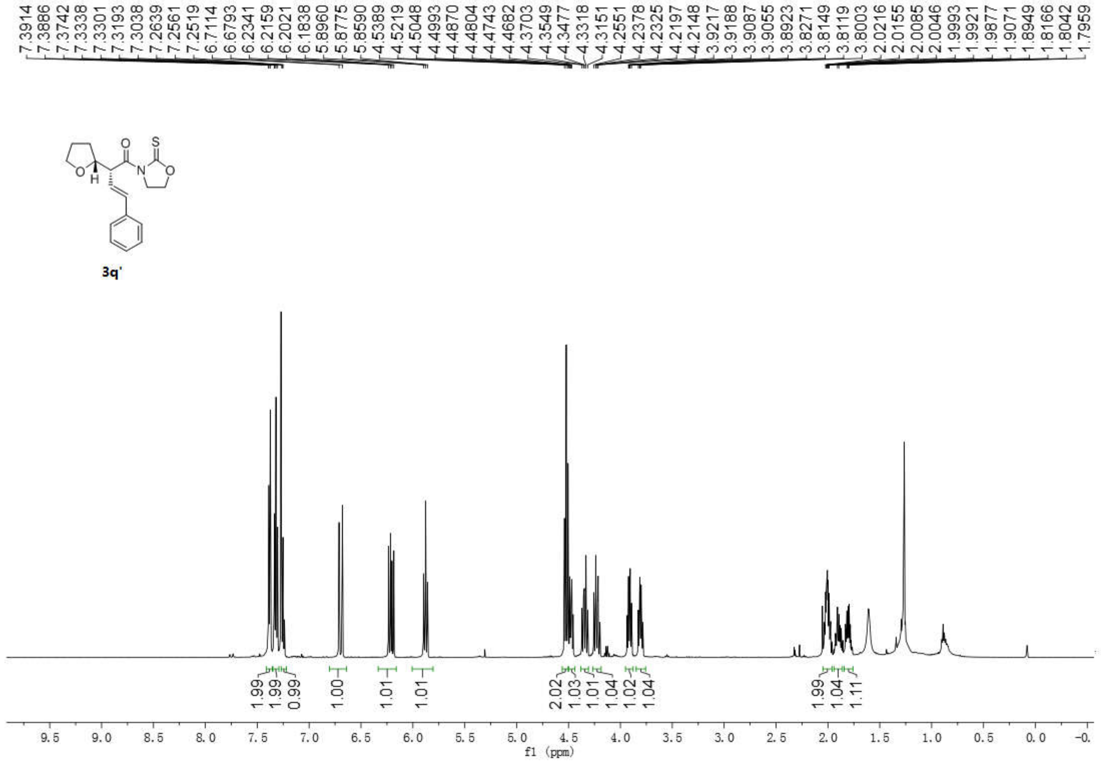

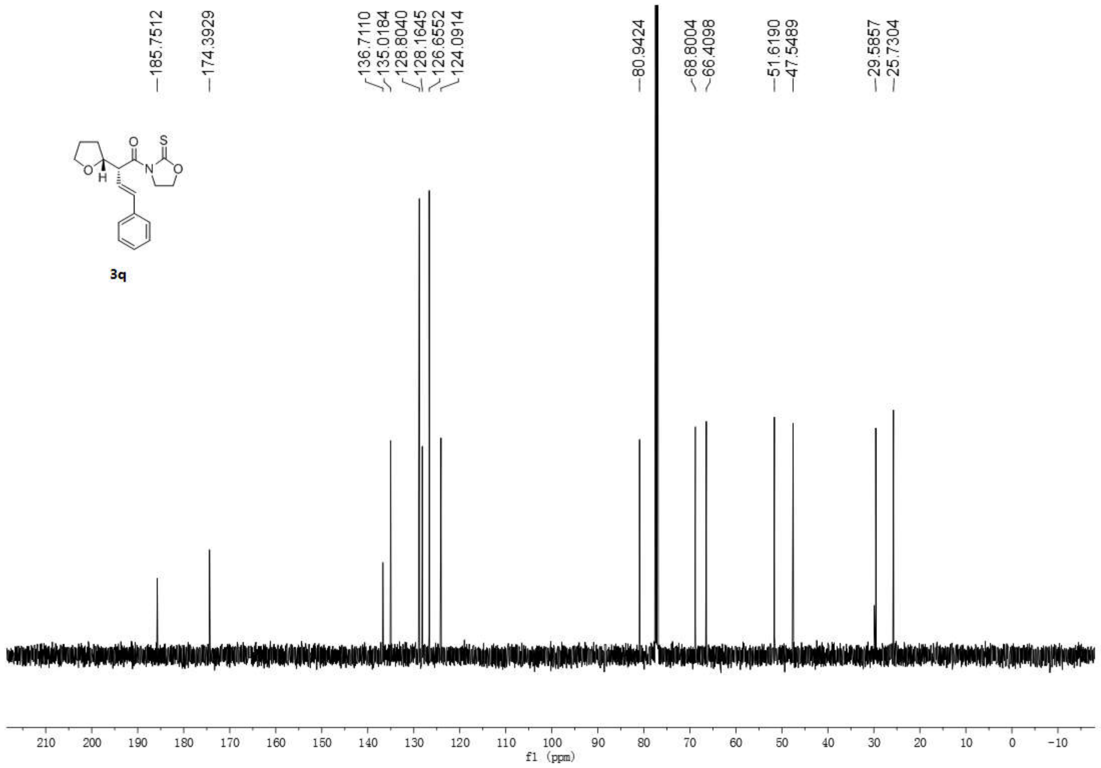

Supplementary figure 73. ^{1}H and ^{13}C NMR spectrum of compound **3q'**

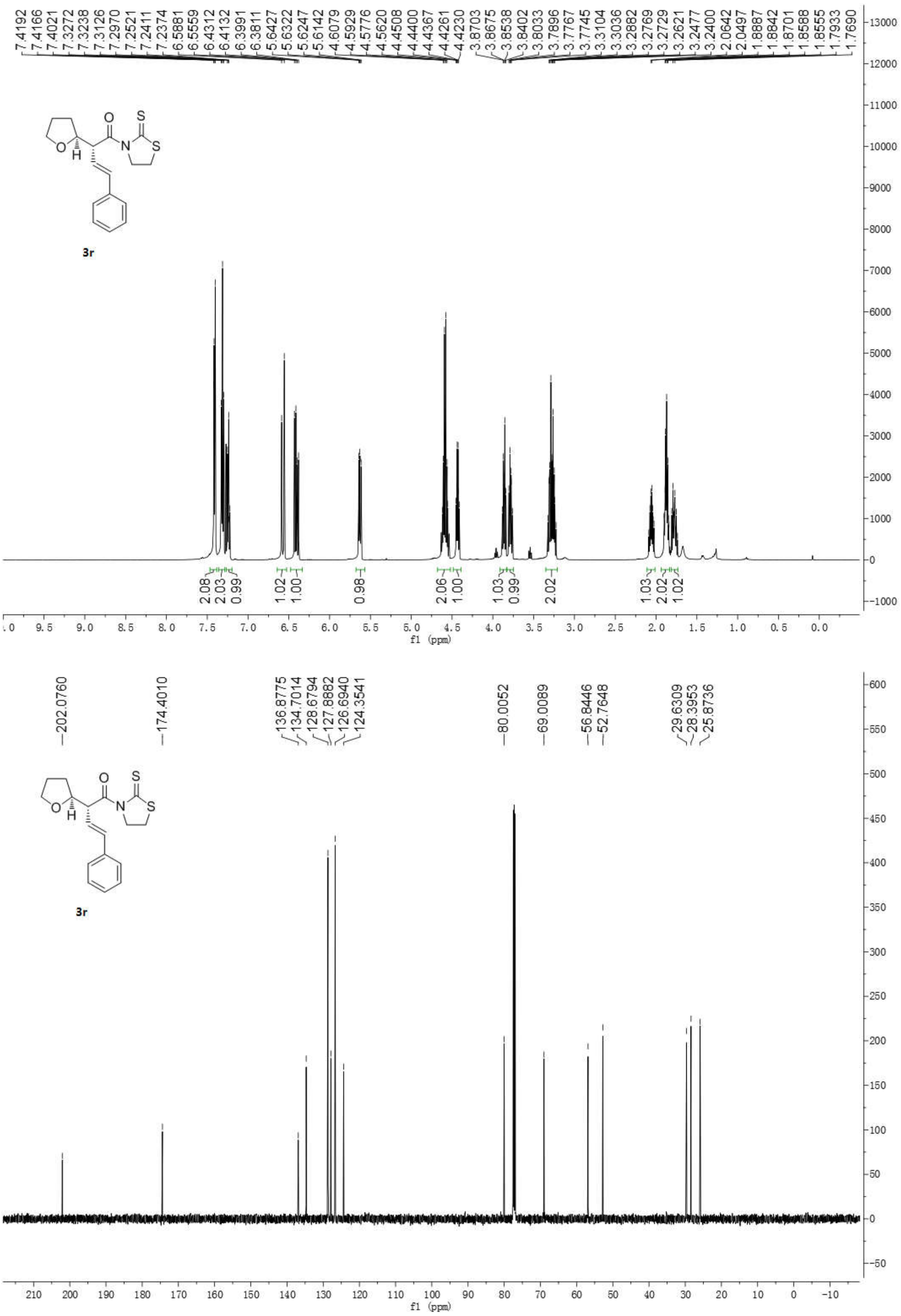

Supplementary figure 74. ^{1}H and ^{13}C NMR spectrum of compound **3r**

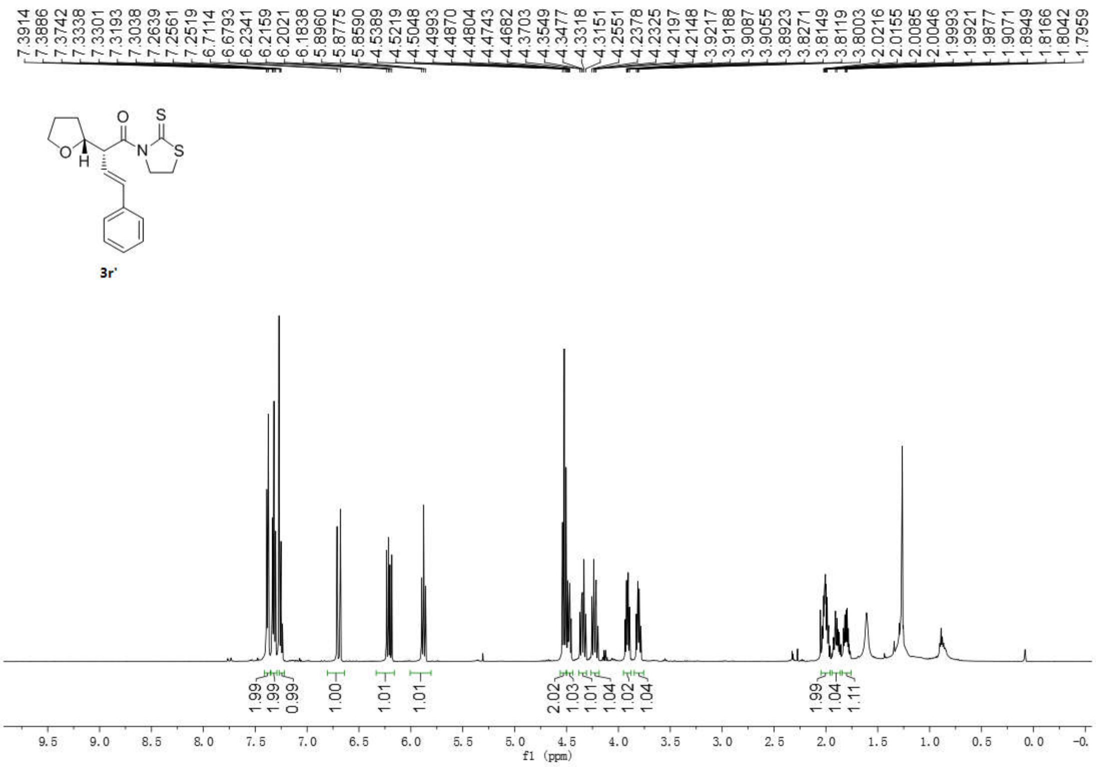

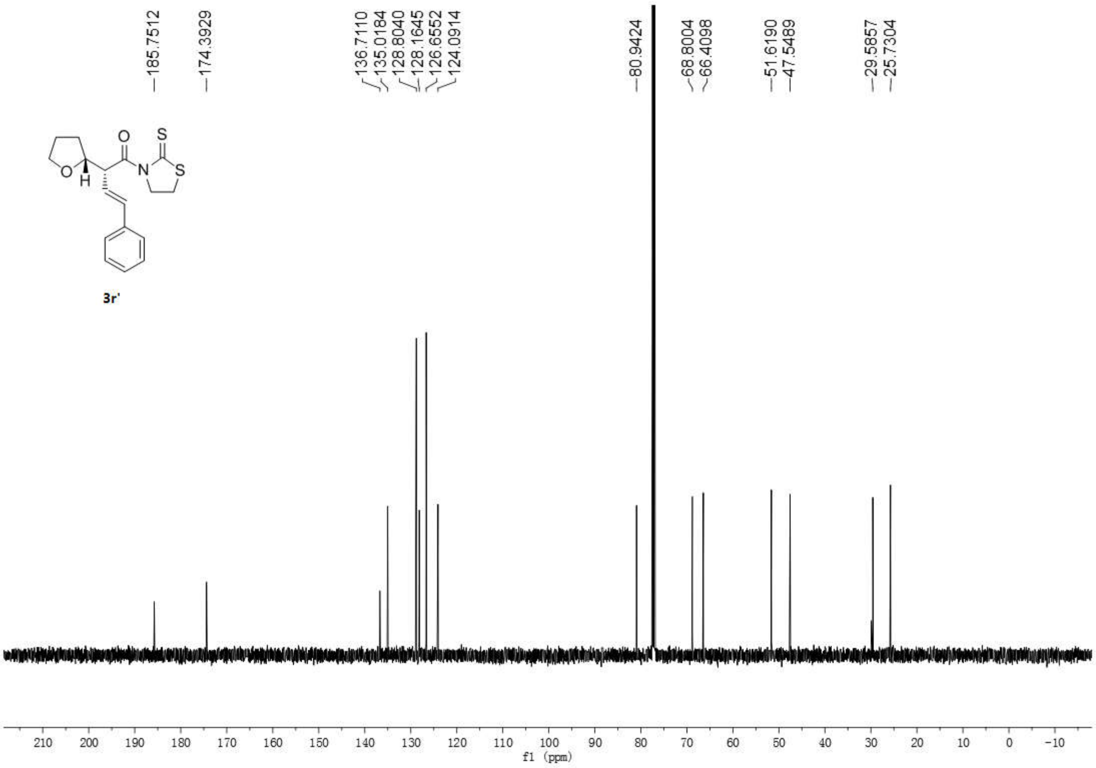

Supplementary figure 75. ^{1}H and ^{13}C NMR spectrum of compound **3r'**

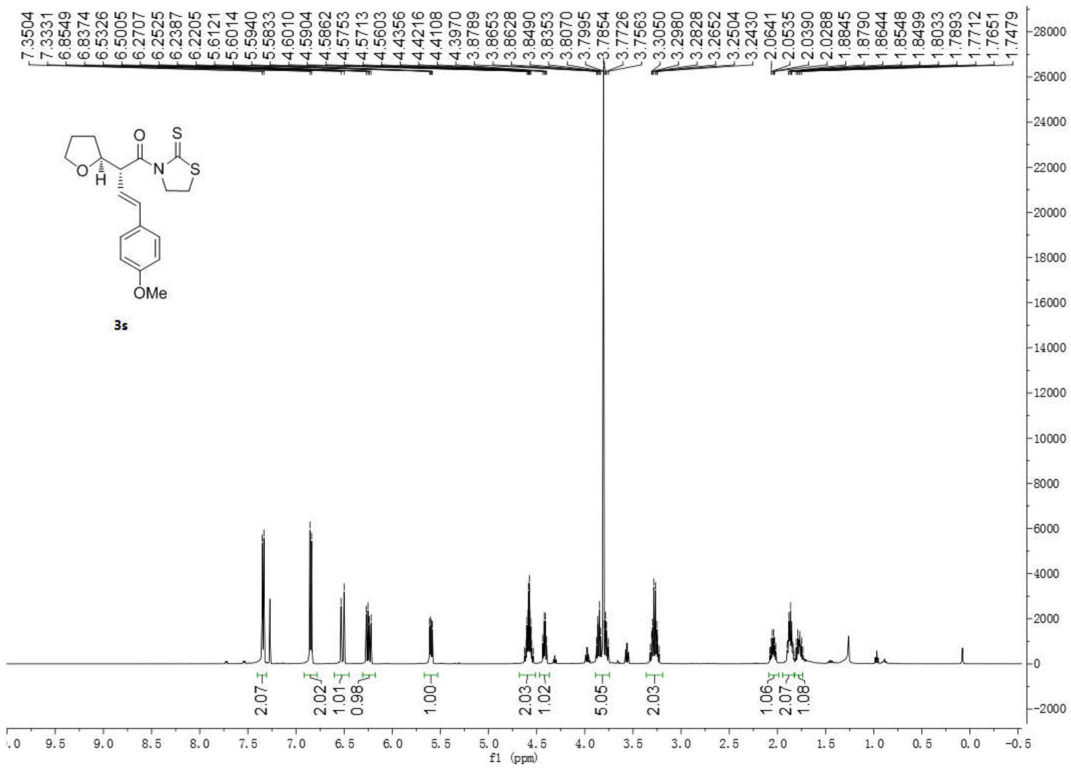

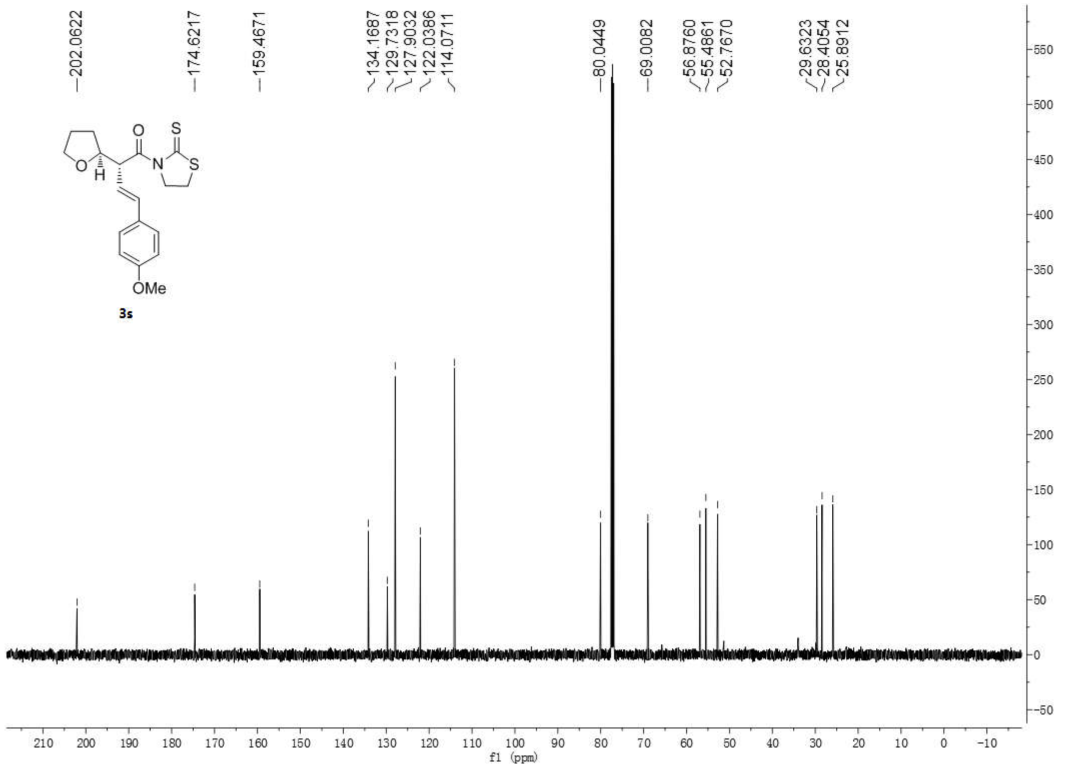

Supplementary figure 76. ^{1}H and ^{13}C NMR spectrum of compound **3s**

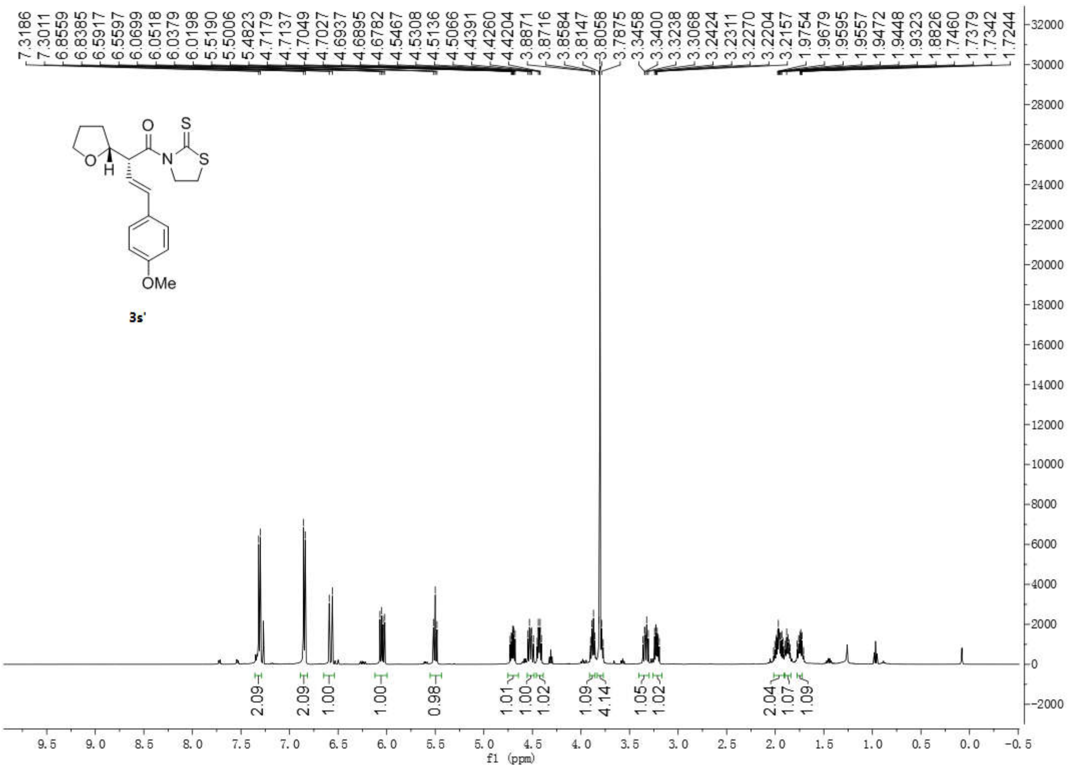

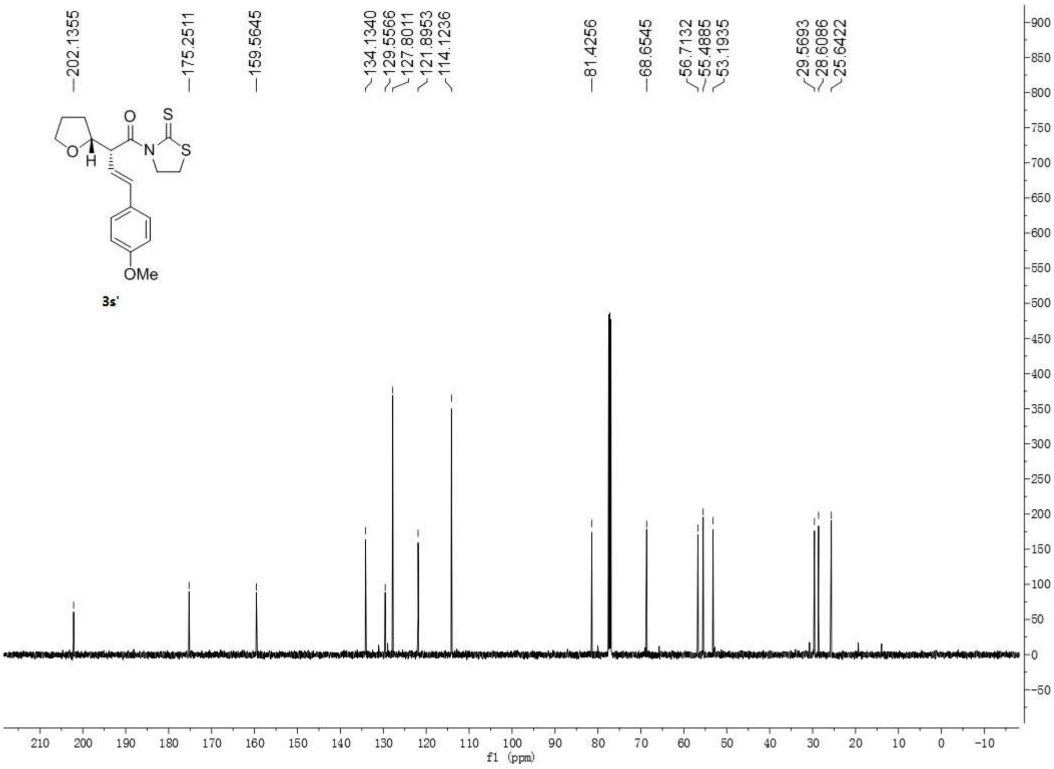

Supplementary figure 77. ^{1}H and ^{13}C NMR spectrum of compound **3s'**

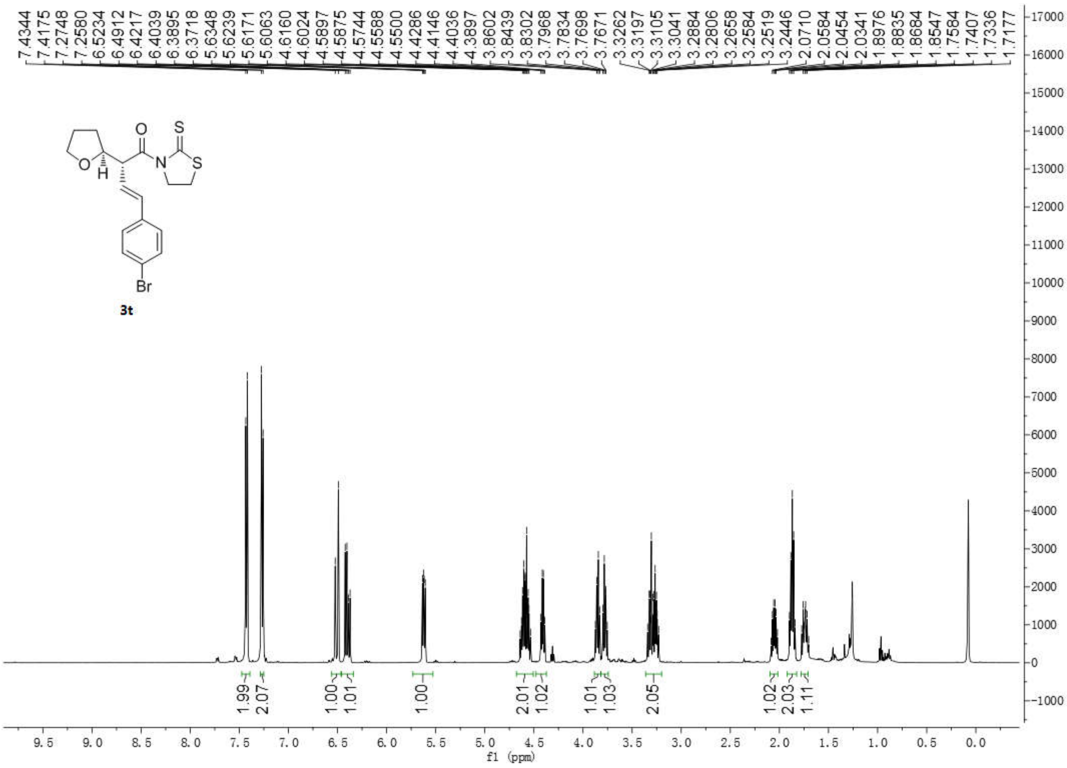

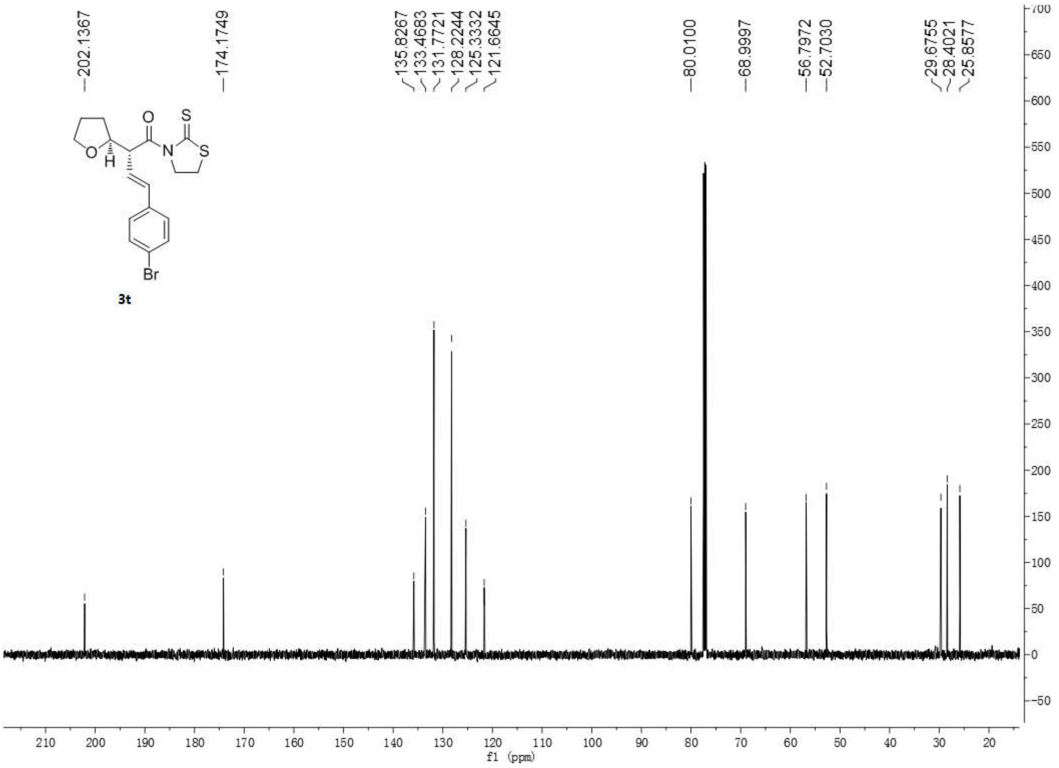

Supplementary figure 78. ^{1}H and ^{13}C NMR spectrum of compound **3t**

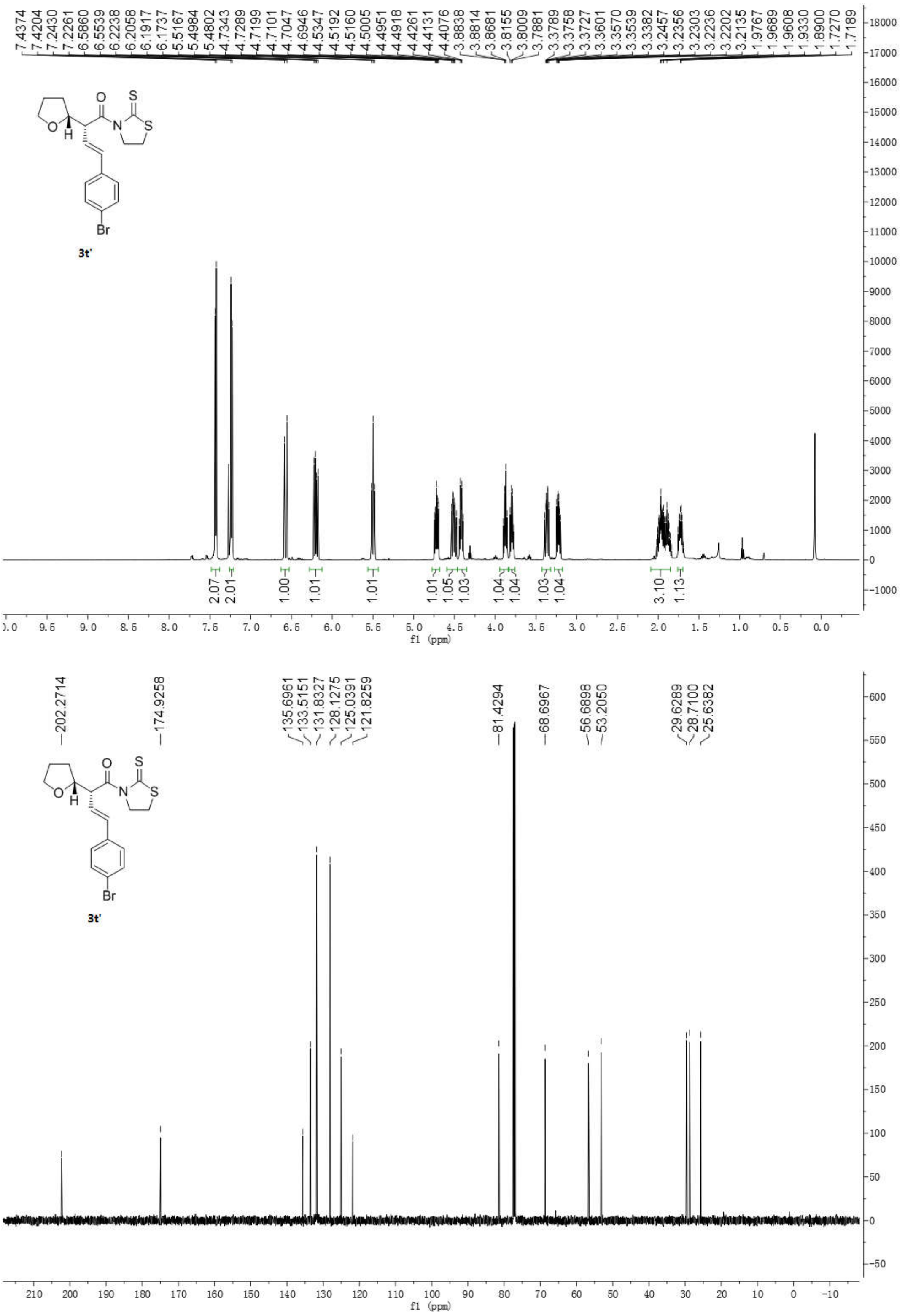

Supplementary figure 79. ^{1}H and ^{13}C NMR spectrum of compound **3t'**

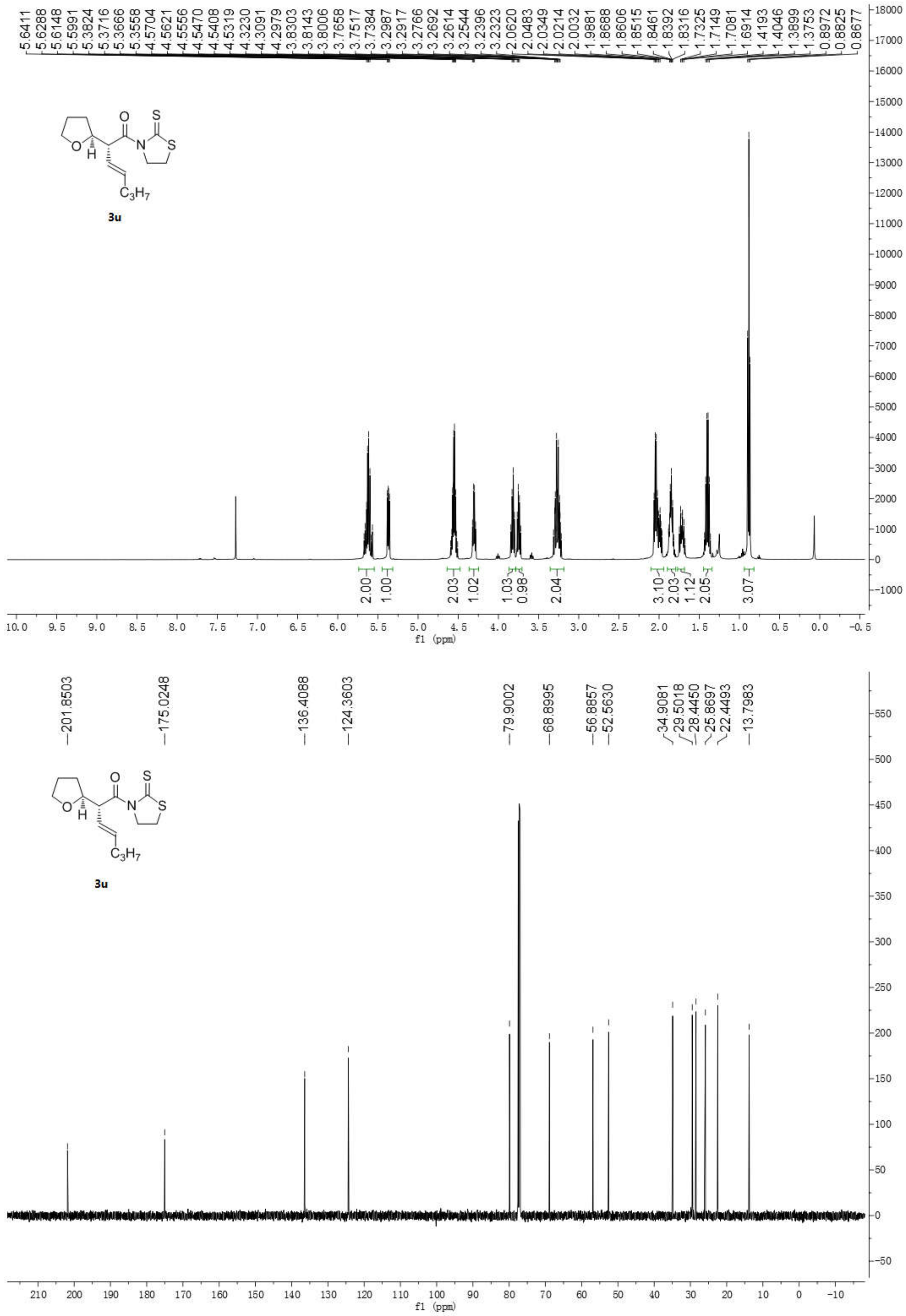

Supplementary figure 80. ^{1}H and ^{13}C NMR spectrum of compound **3u**

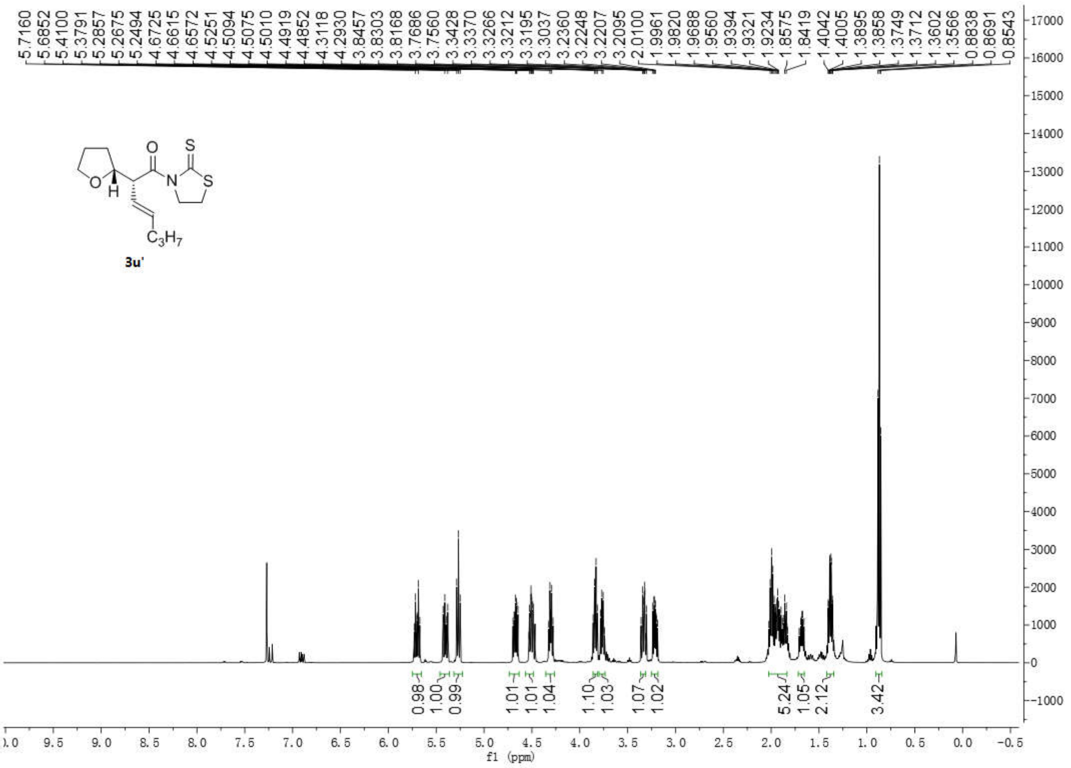

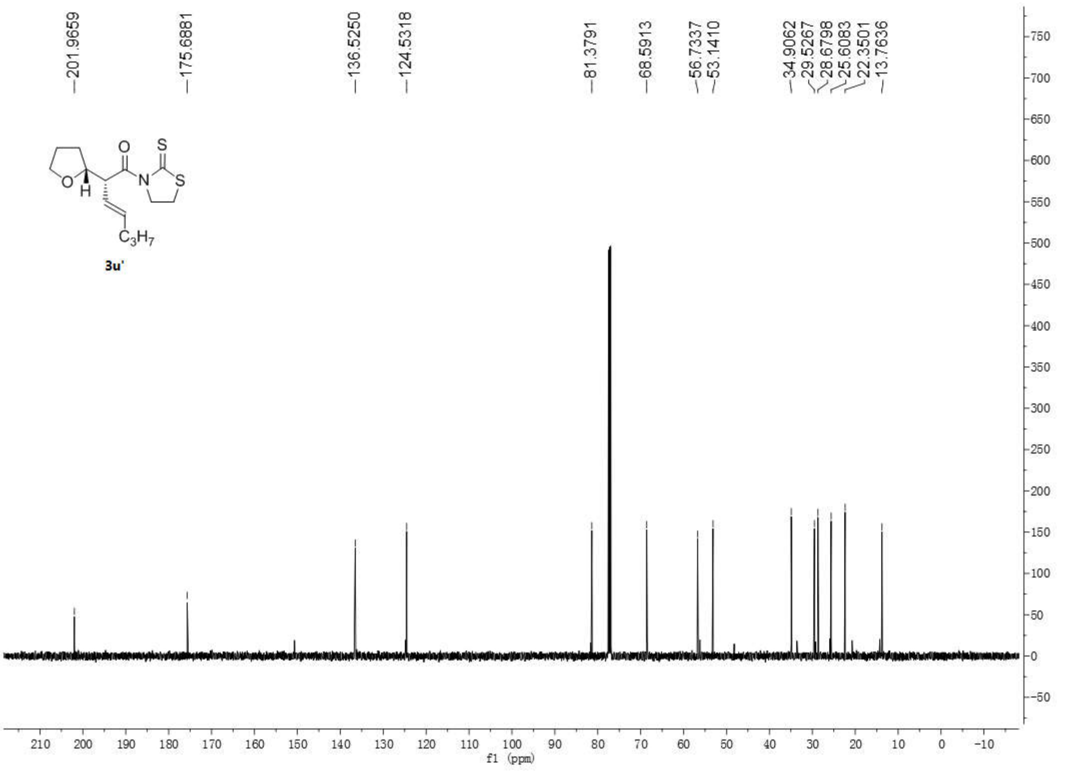

Supplementary figure 81. ^{1}H and ^{13}C NMR spectrum of compound **3u'**

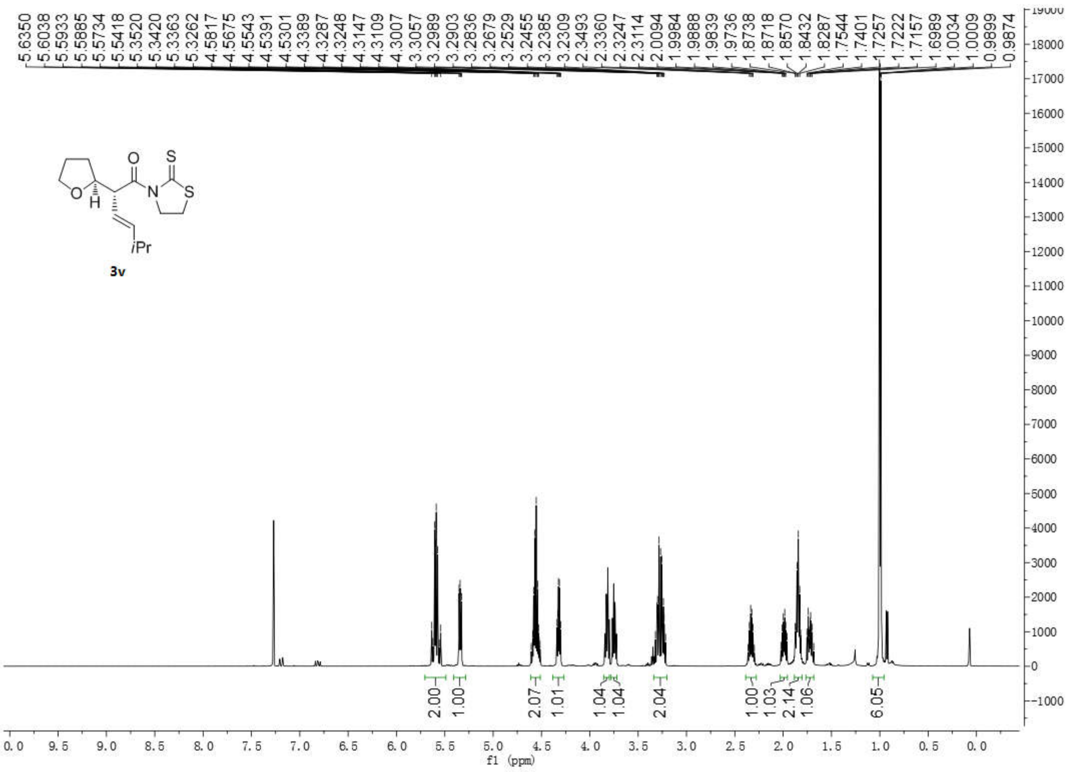

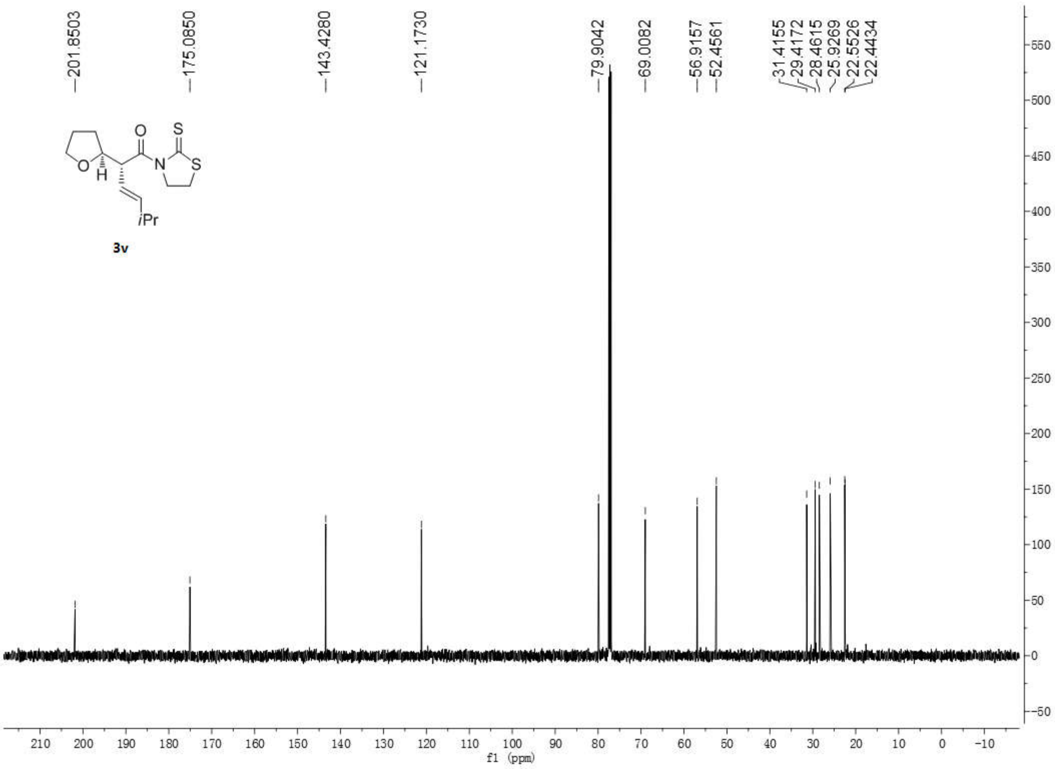

Supplementary figure 82. ^{1}H and ^{13}C NMR spectrum of compound **3v**

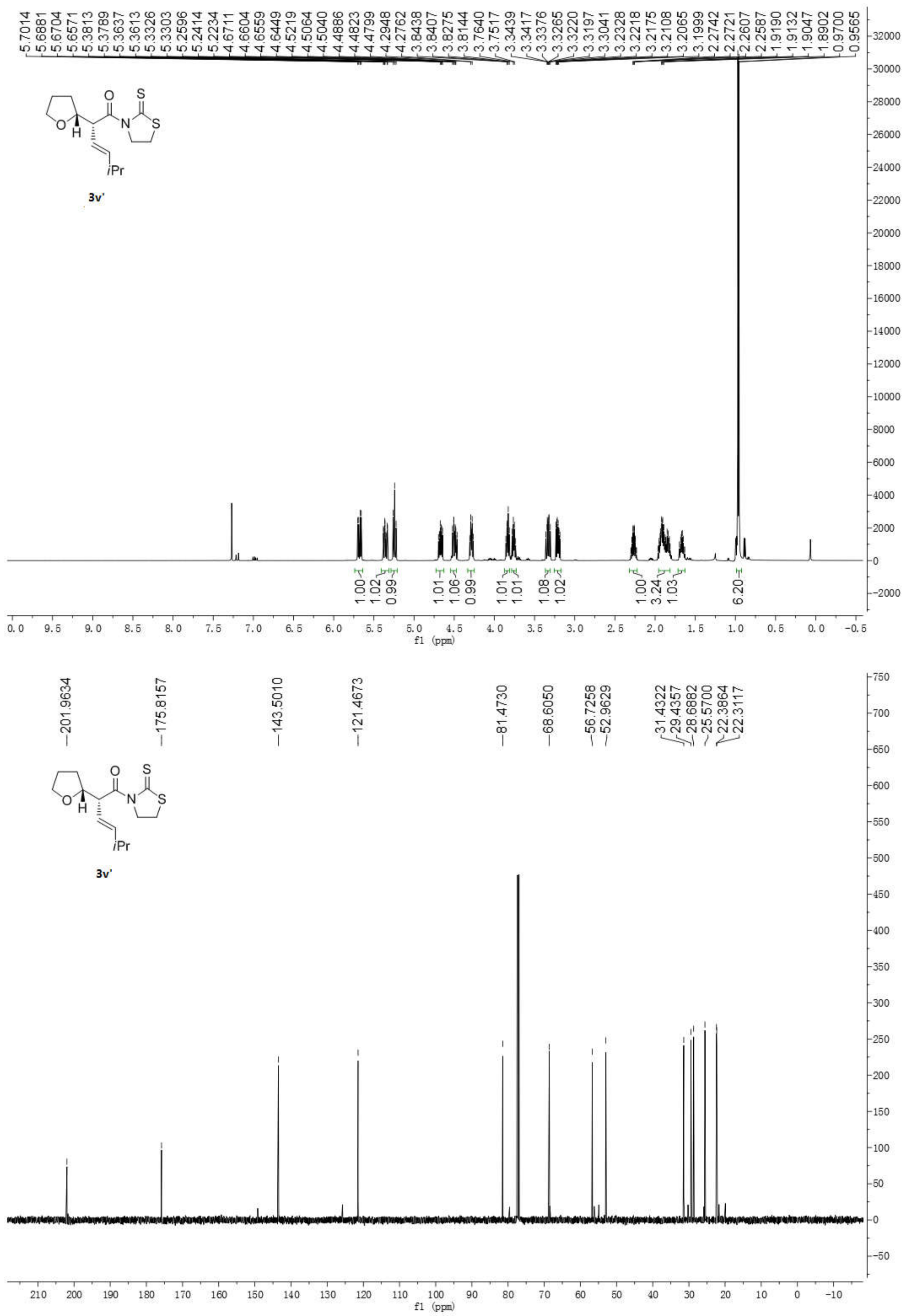

Supplementary figure 83. ^{1}H and ^{13}C NMR spectrum of compound **3v'**

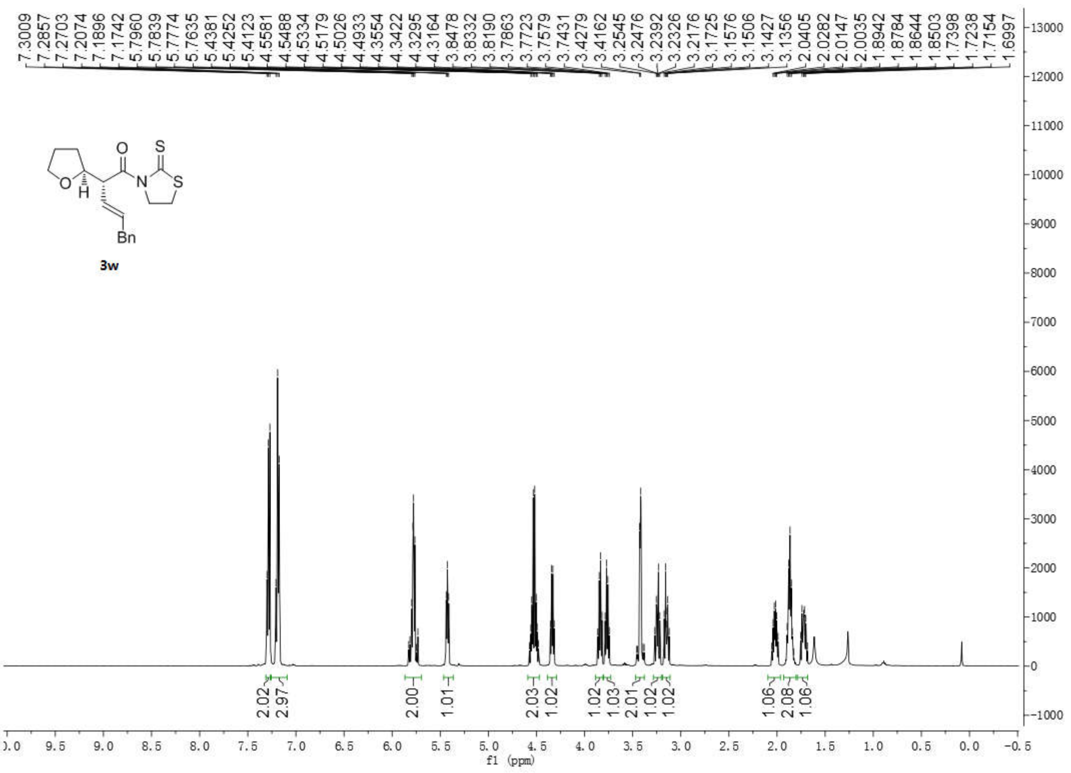

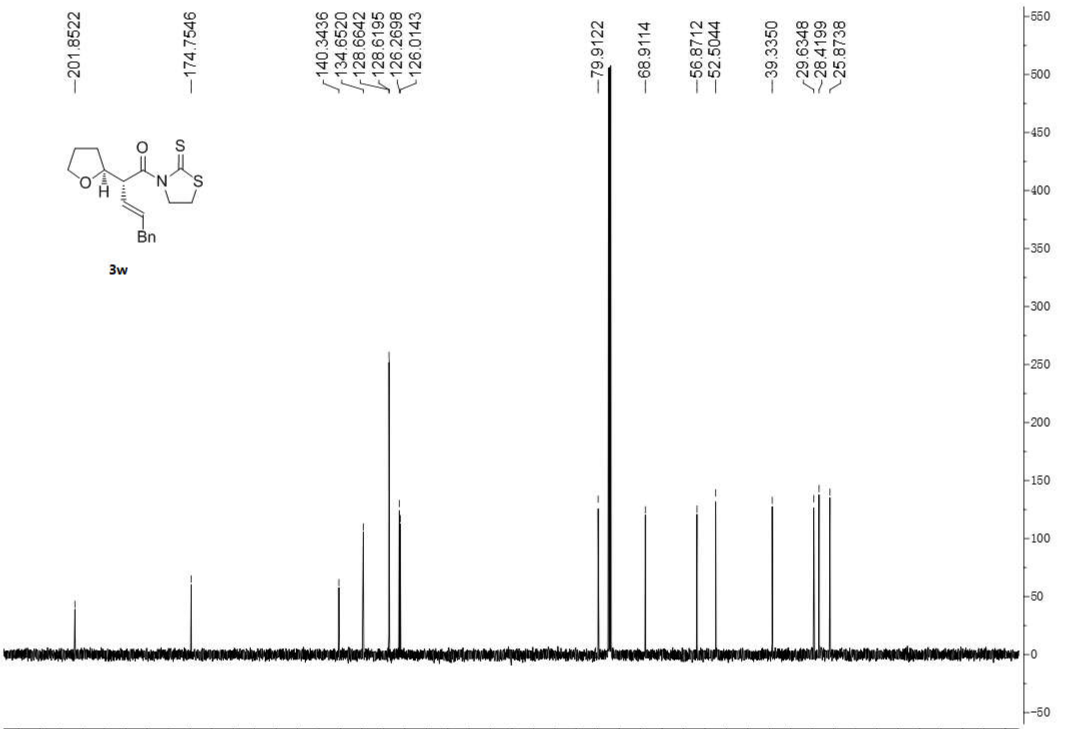

Supplementary figure 84. ^{1}H and ^{13}C NMR spectrum of compound **3w**

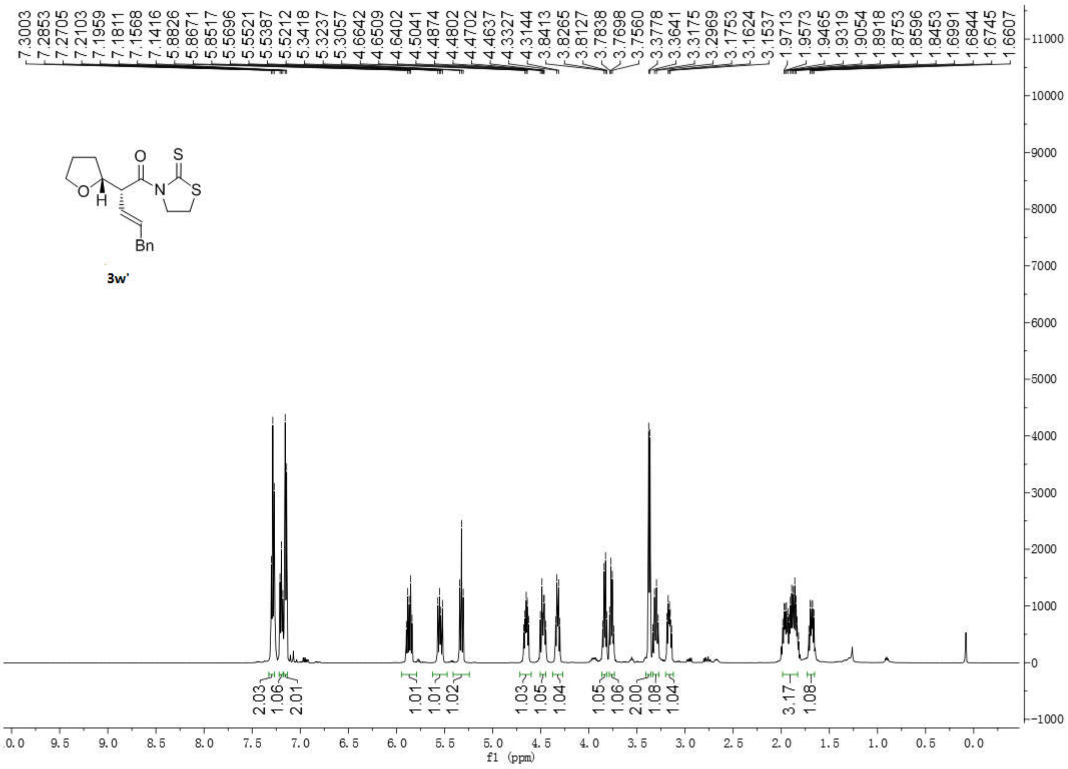

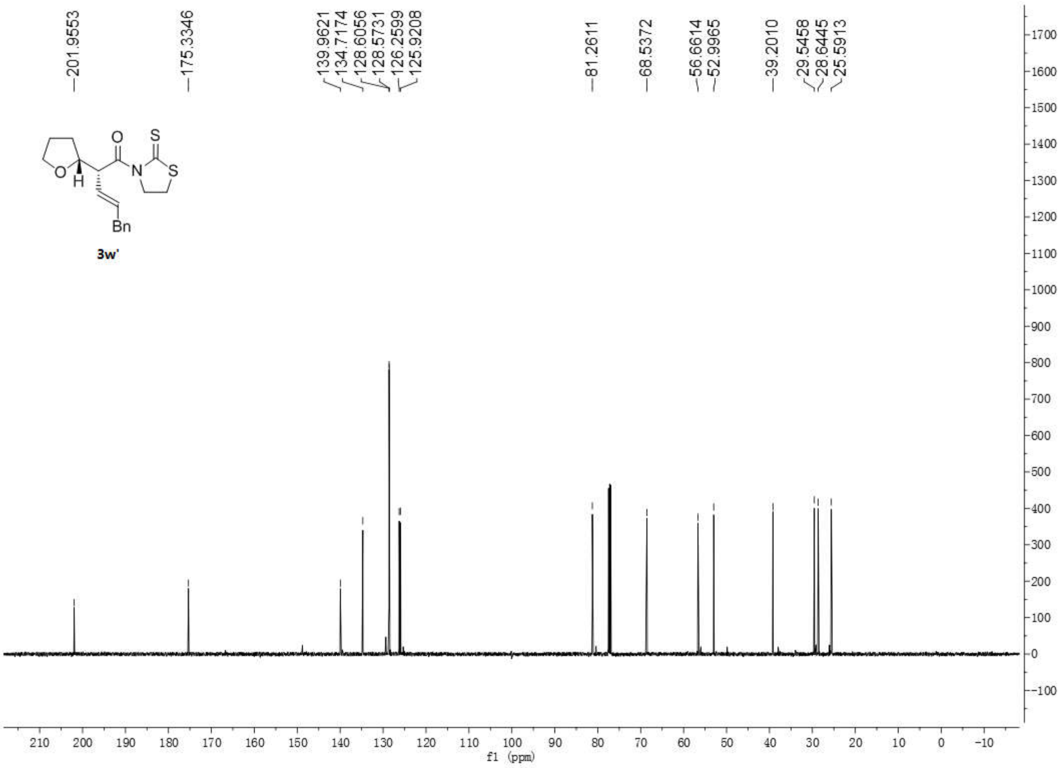

Supplementary figure 85. ^{1}H and ^{13}C NMR spectrum of compound **3w’**

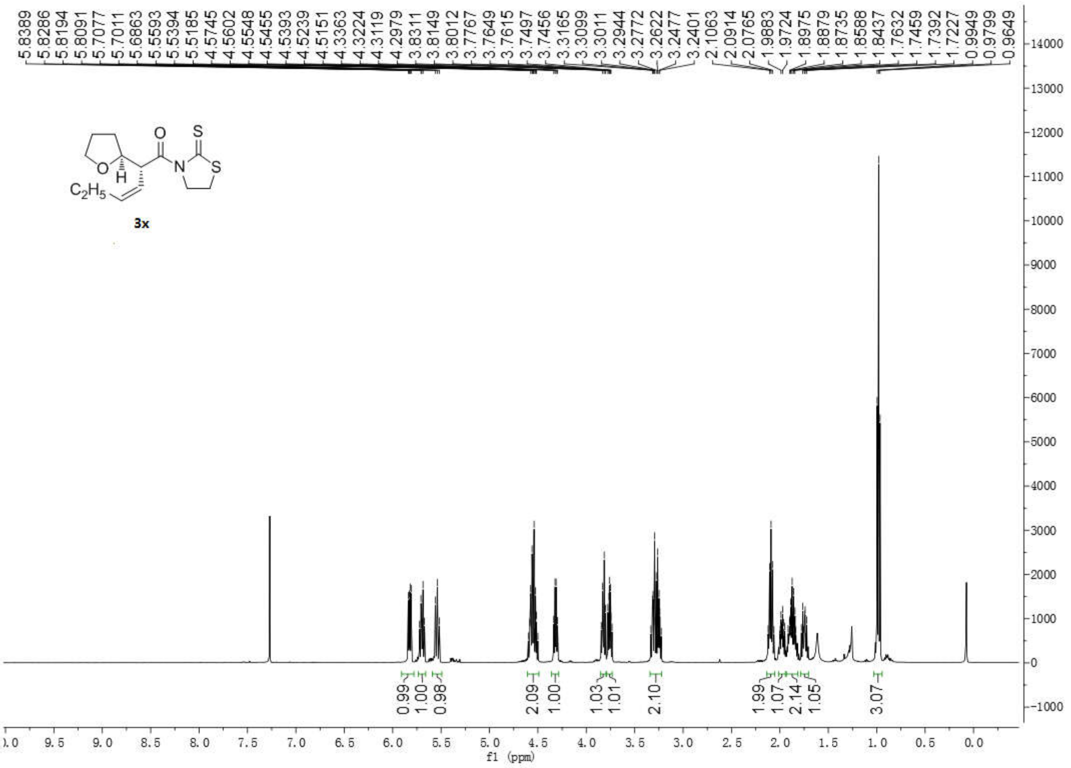

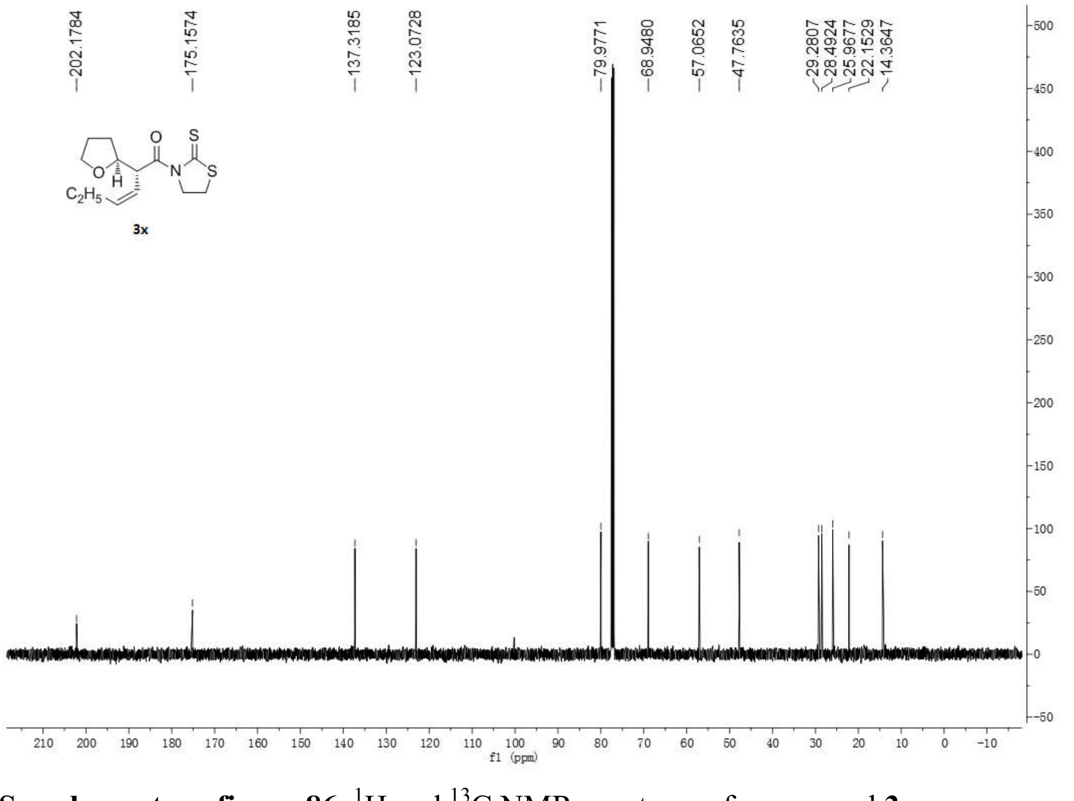

Supplementary figure 86. ^{1}H and ^{13}C NMR spectrum of compound **3x**

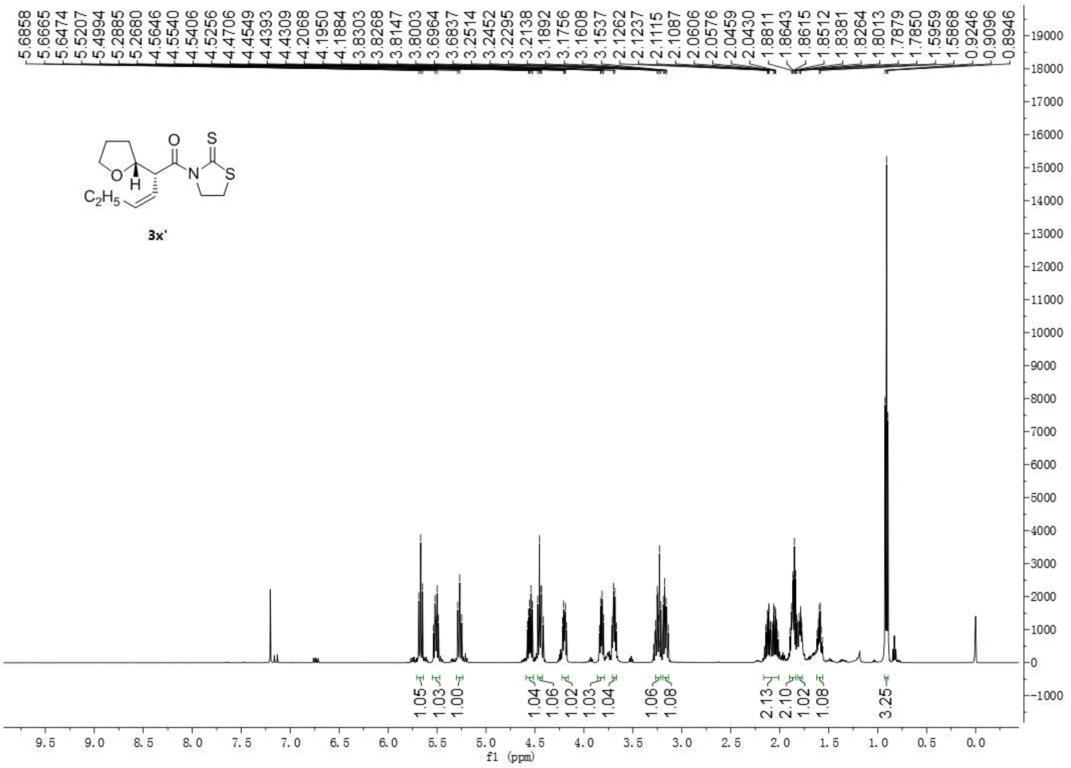

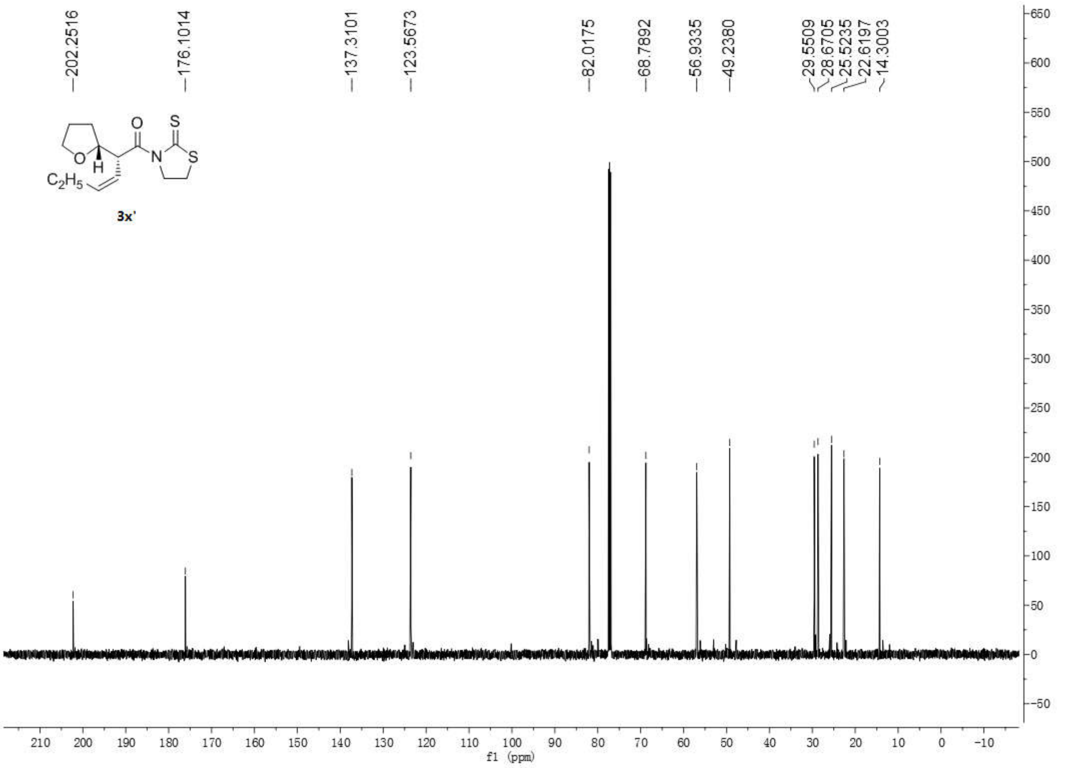

Supplementary figure 87. ^{1}H and ^{13}C NMR spectrum of compound **3x'**

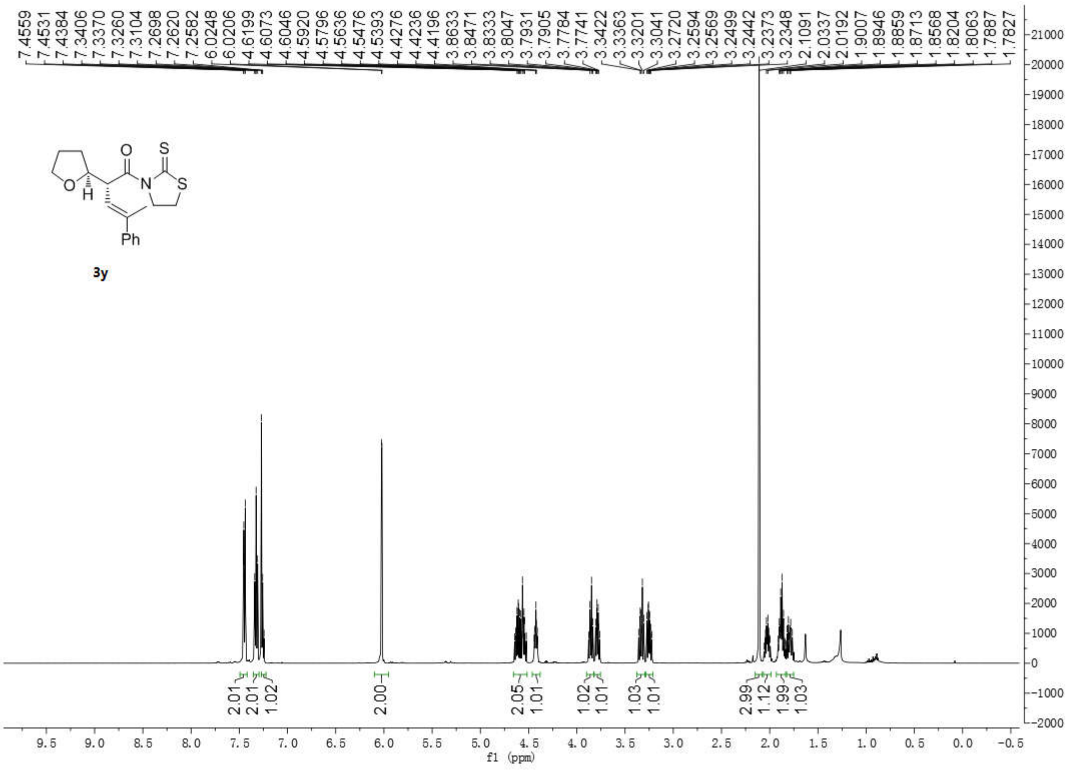

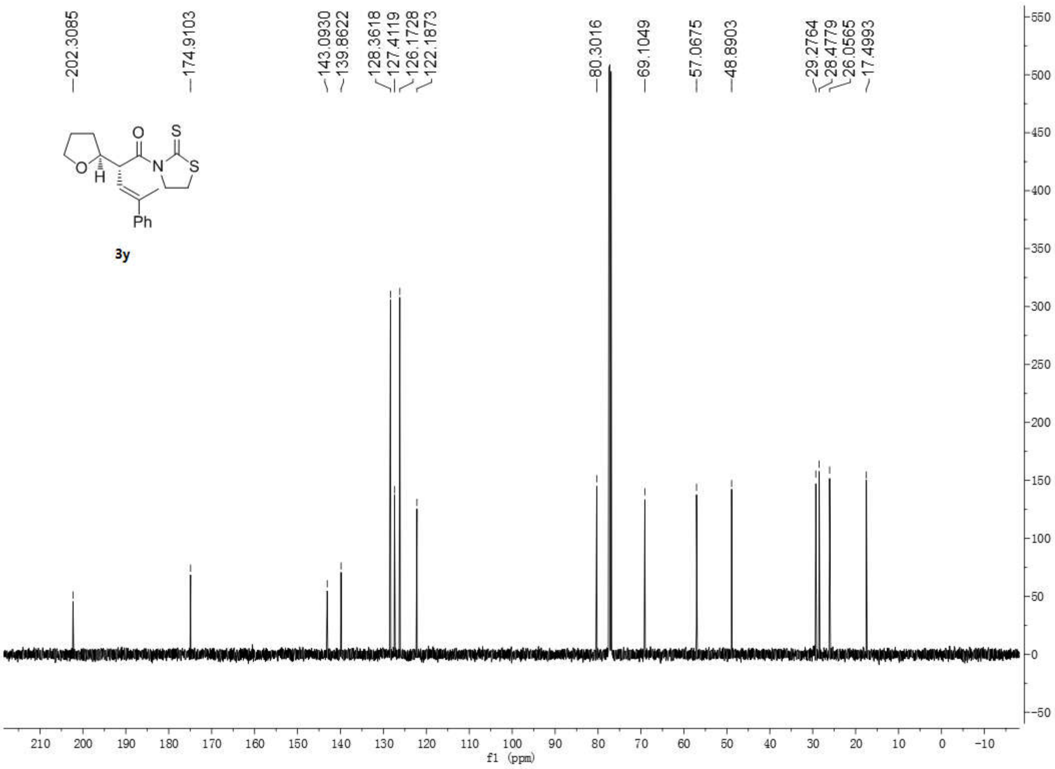

Supplementary figure 88. ^{1}H and ^{13}C NMR spectrum of compound **3y**

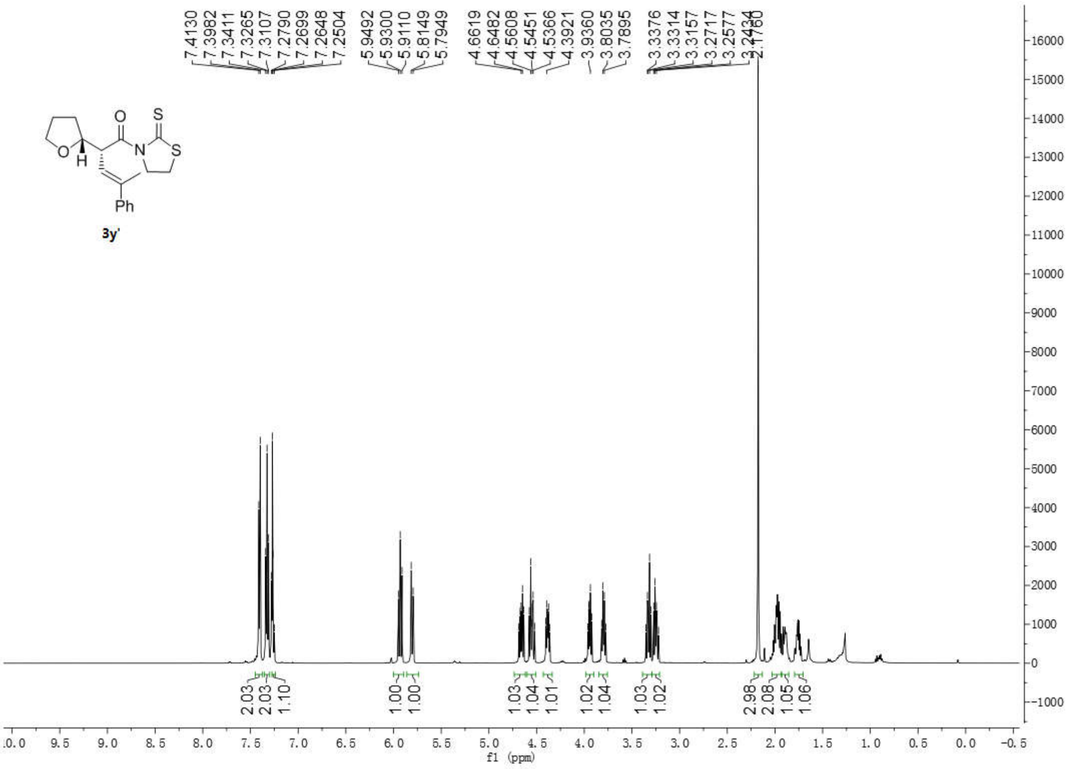

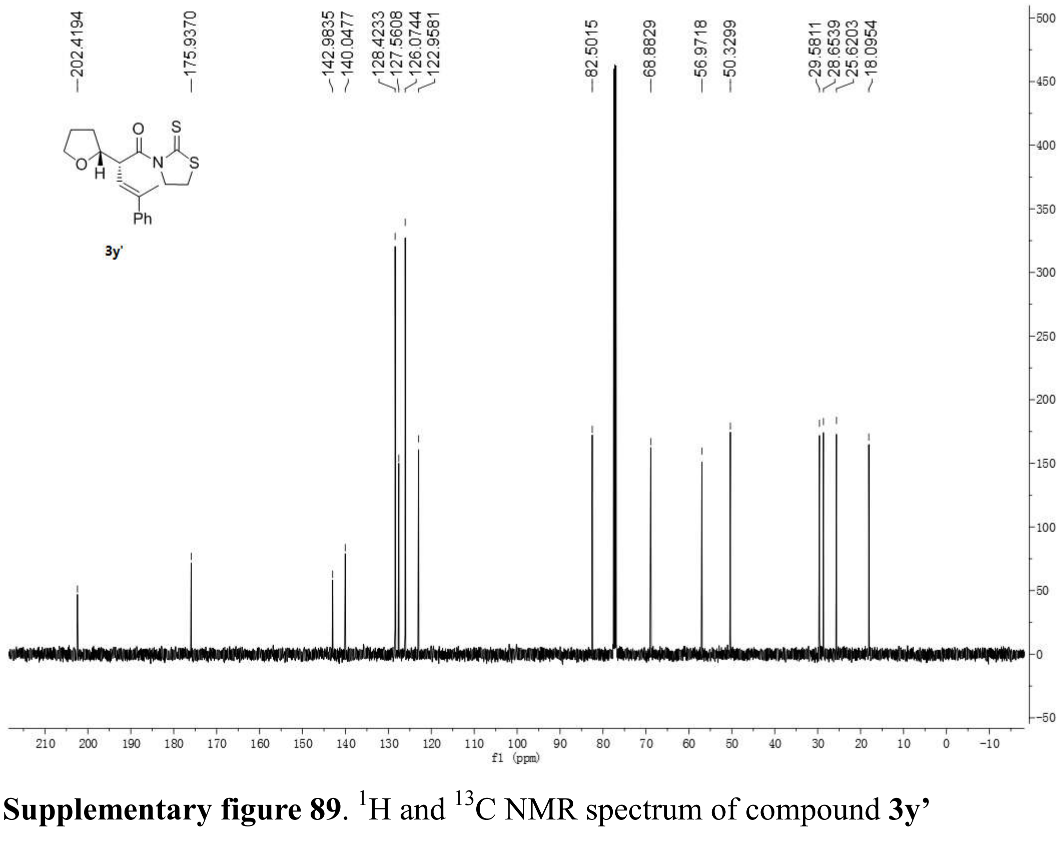

Supplementary figure 89. ^{1}H and ^{13}C NMR spectrum of compound **3y'**

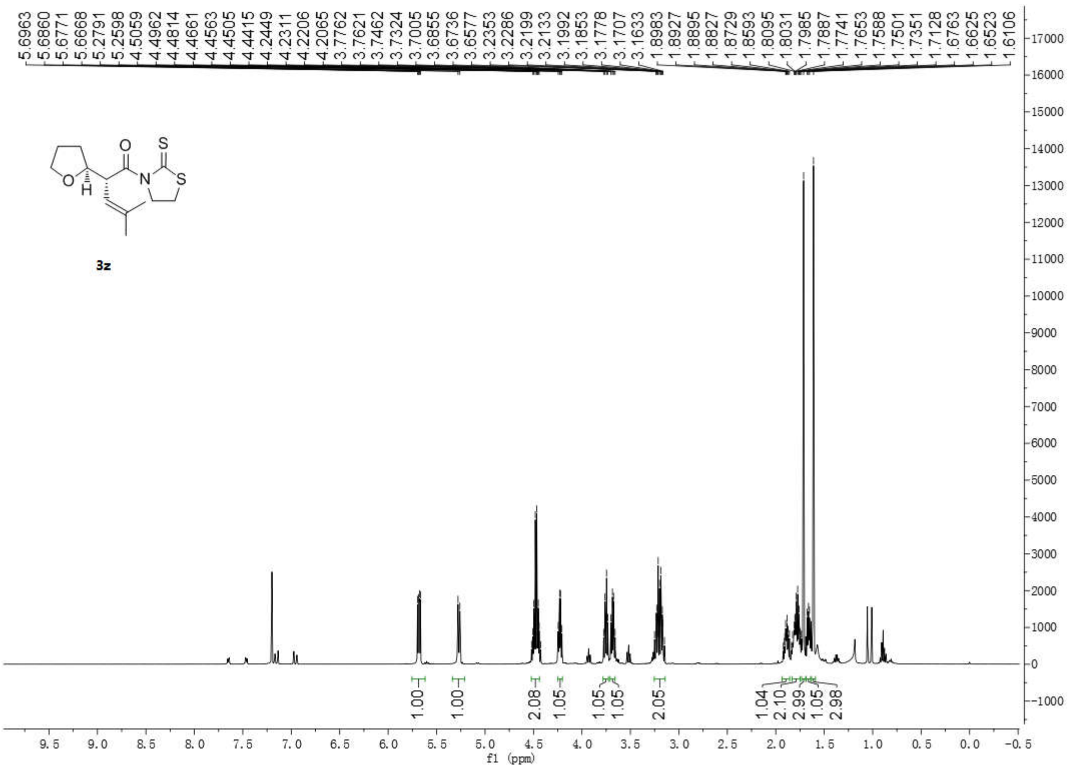

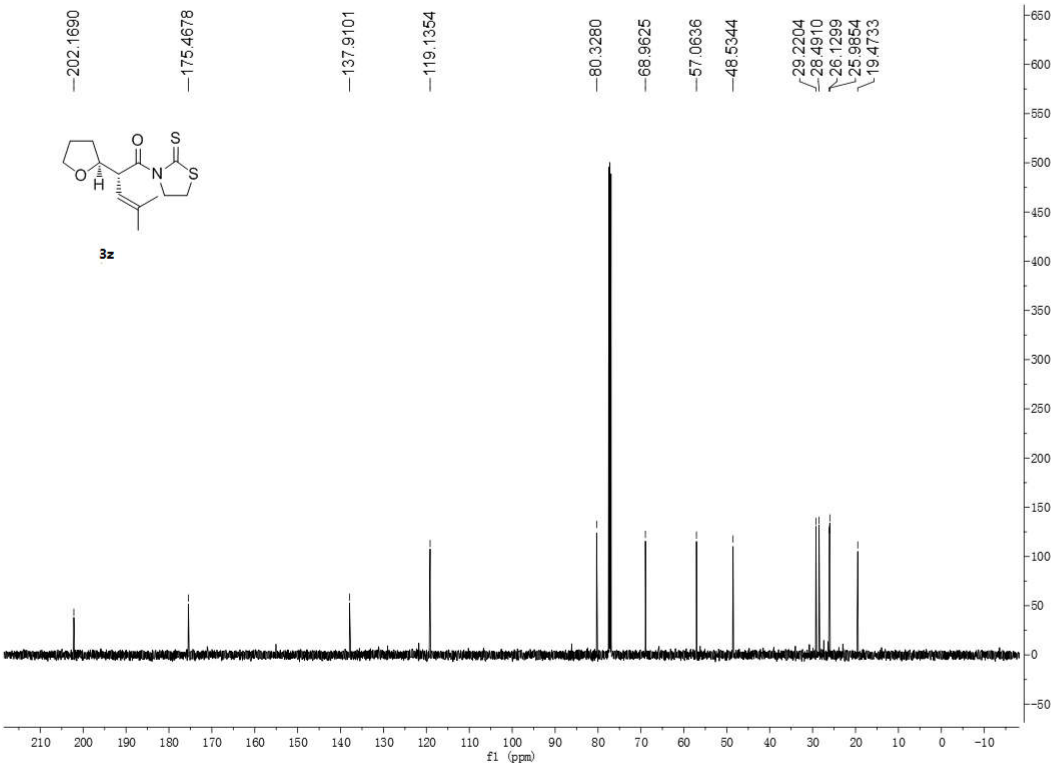

Supplementary figure 90. ^{1}H and ^{13}C NMR spectrum of compound **3z**

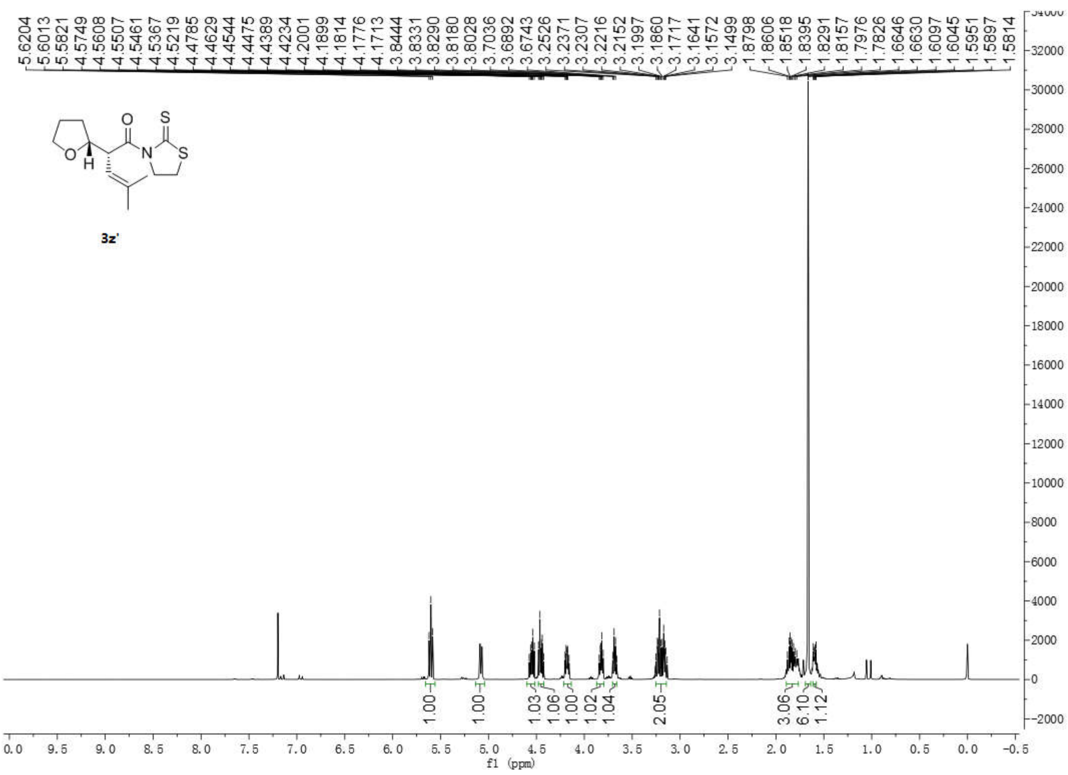

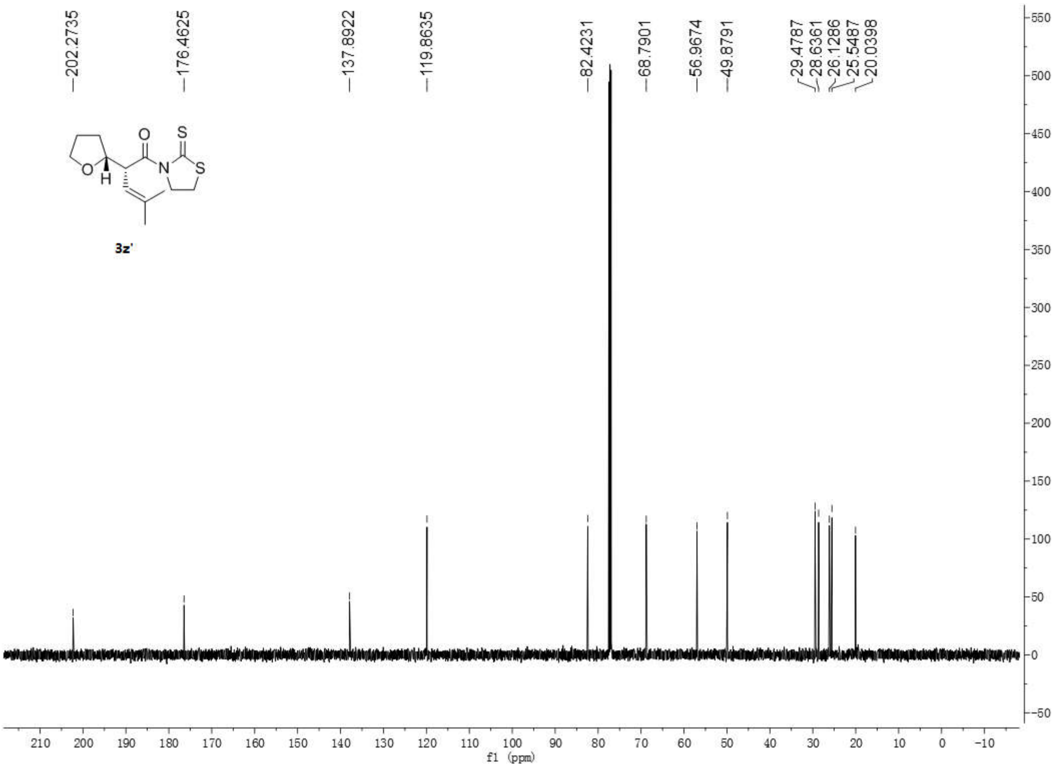

Supplementary figure 91. ^{1}H and ^{13}C NMR spectrum of compound **3z'**

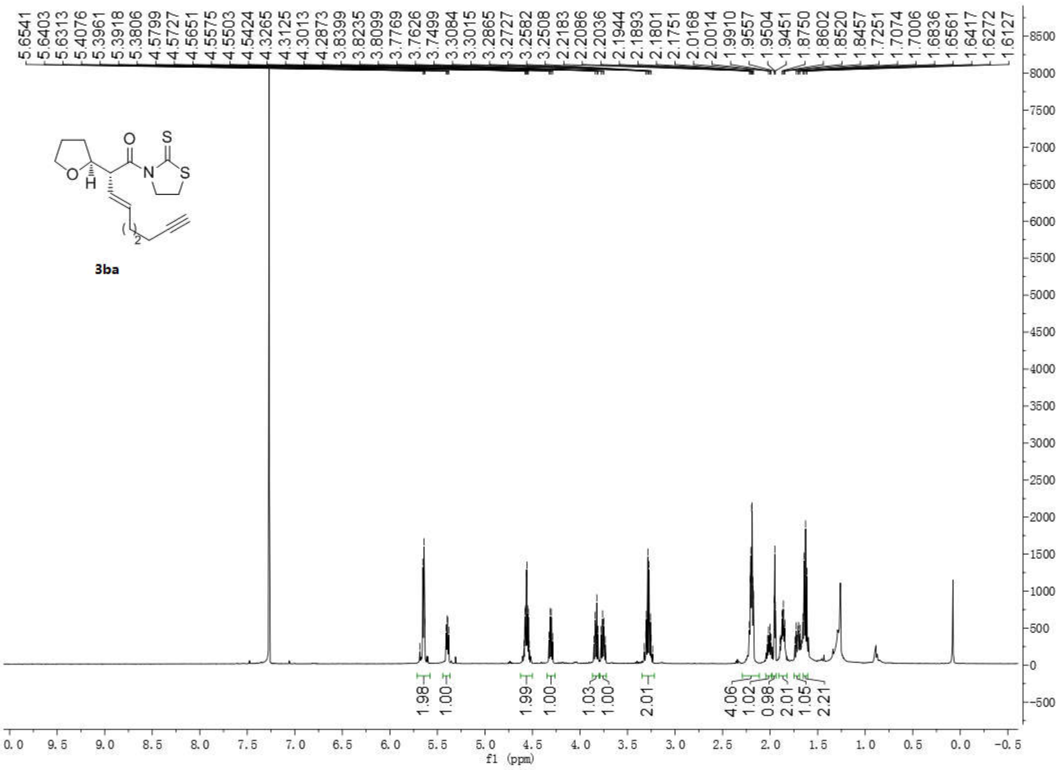

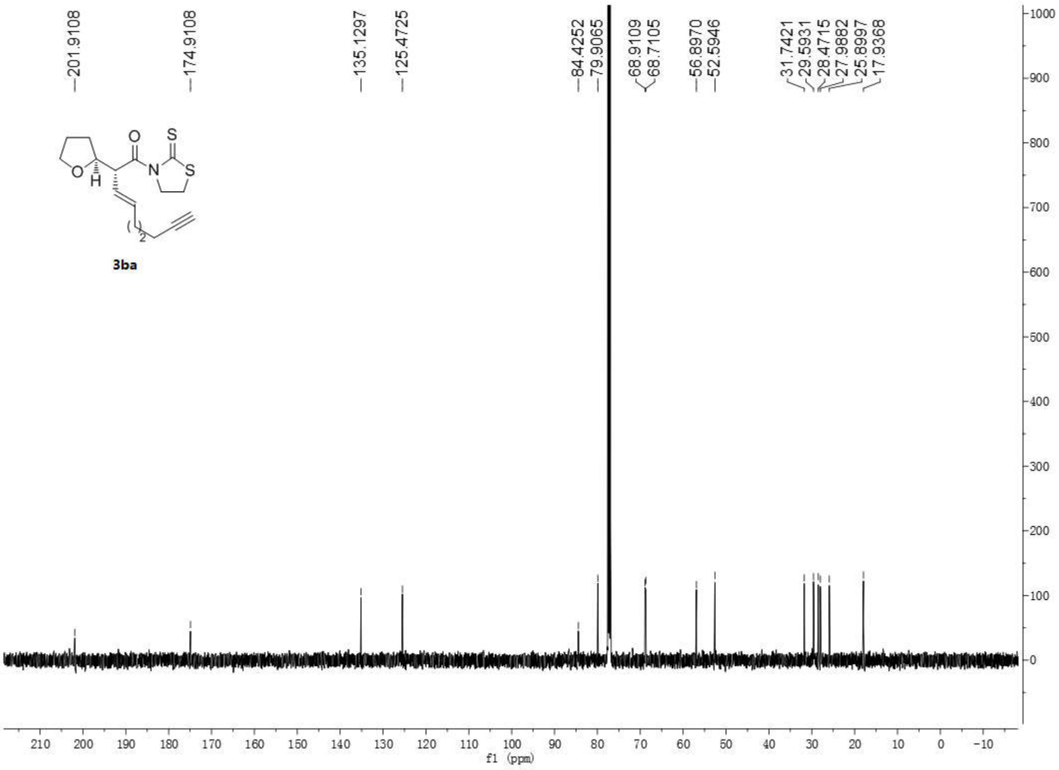

Supplementary figure 92. ^{1}H and ^{13}C NMR spectrum of compound **3**

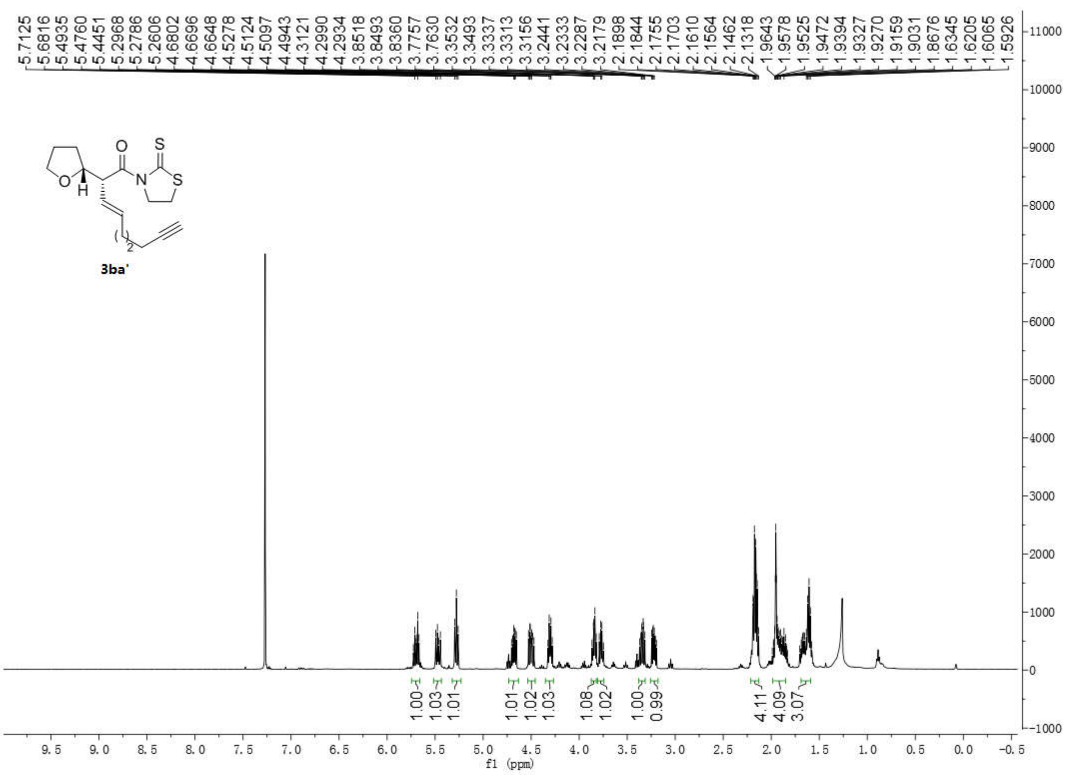

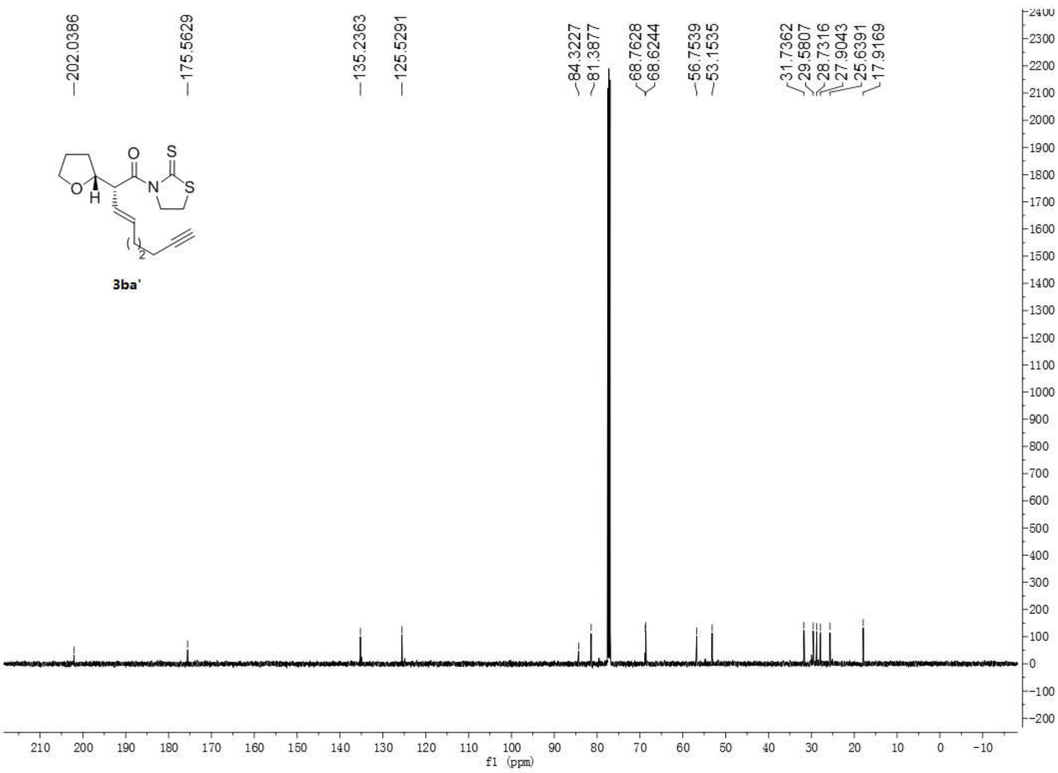

Supplementary figure 93. ^{1}H and ^{13}C NMR spectrum of compound **3**

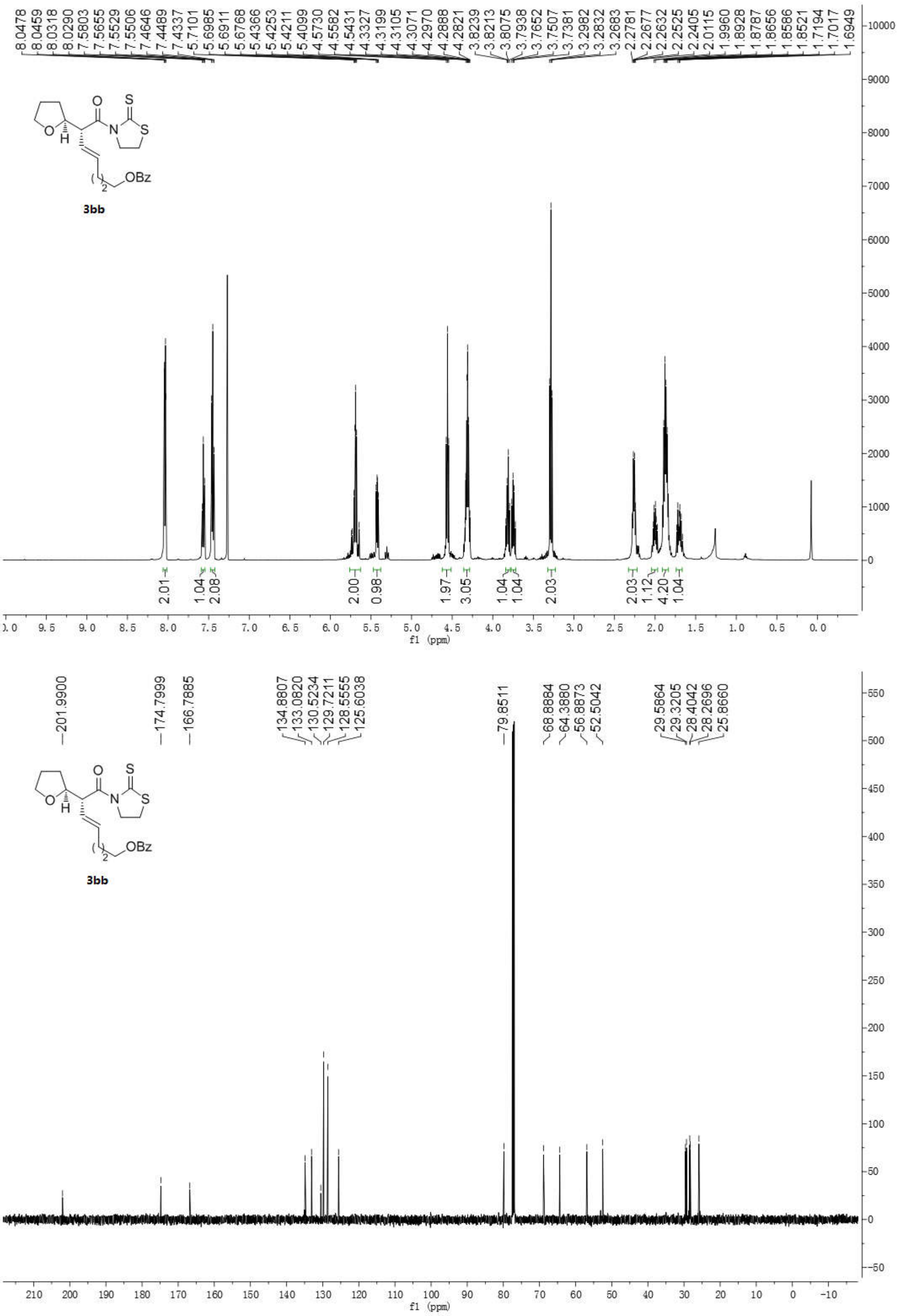

Supplementary figure 94. ^{1}H and ^{13}C NMR spectrum of compound **3bb**

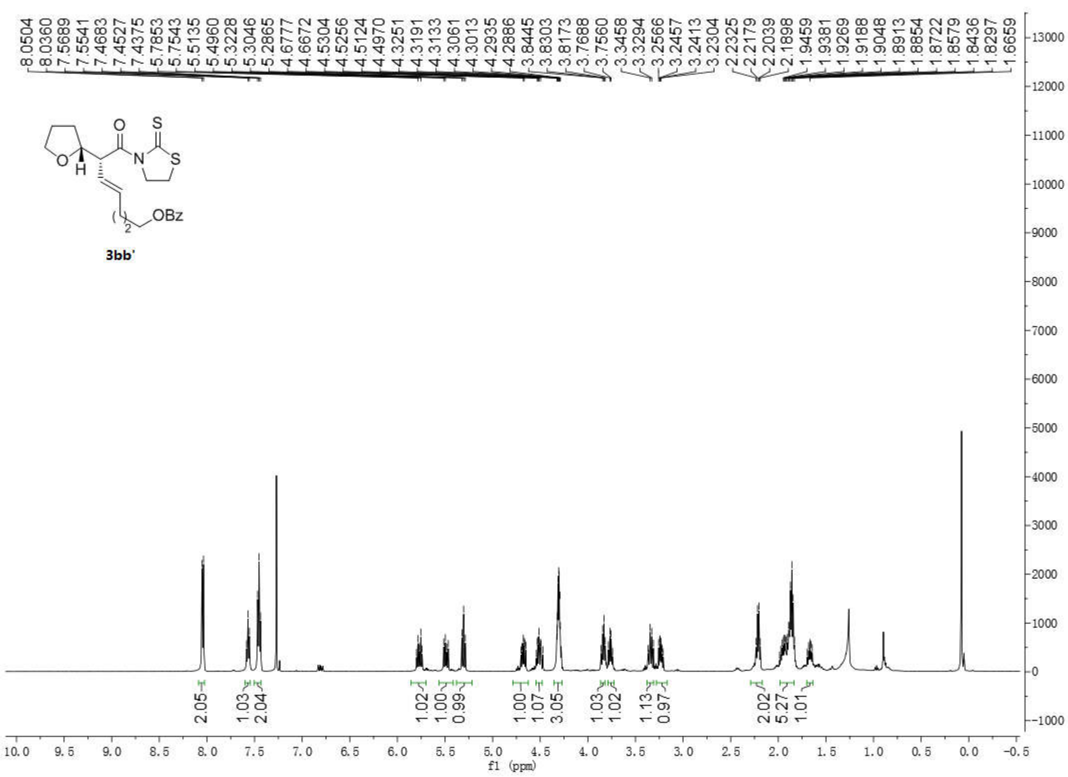

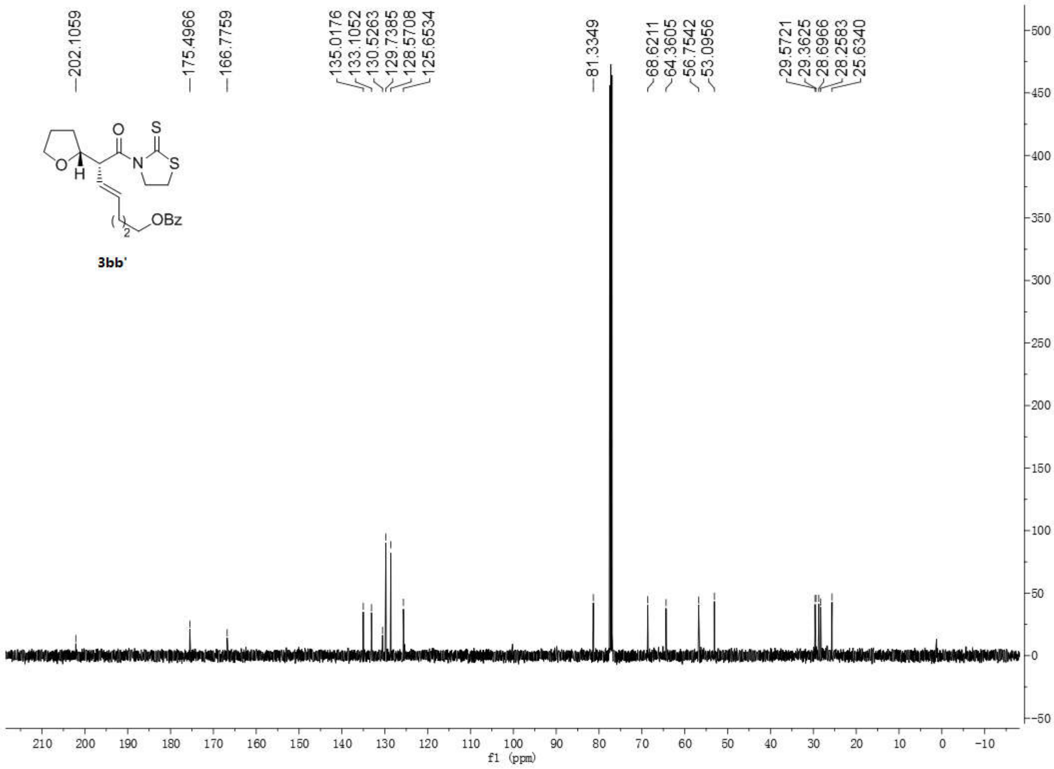

Supplementary figure 95. ^{1}H and ^{13}C NMR spectrum of compound **3bb'**

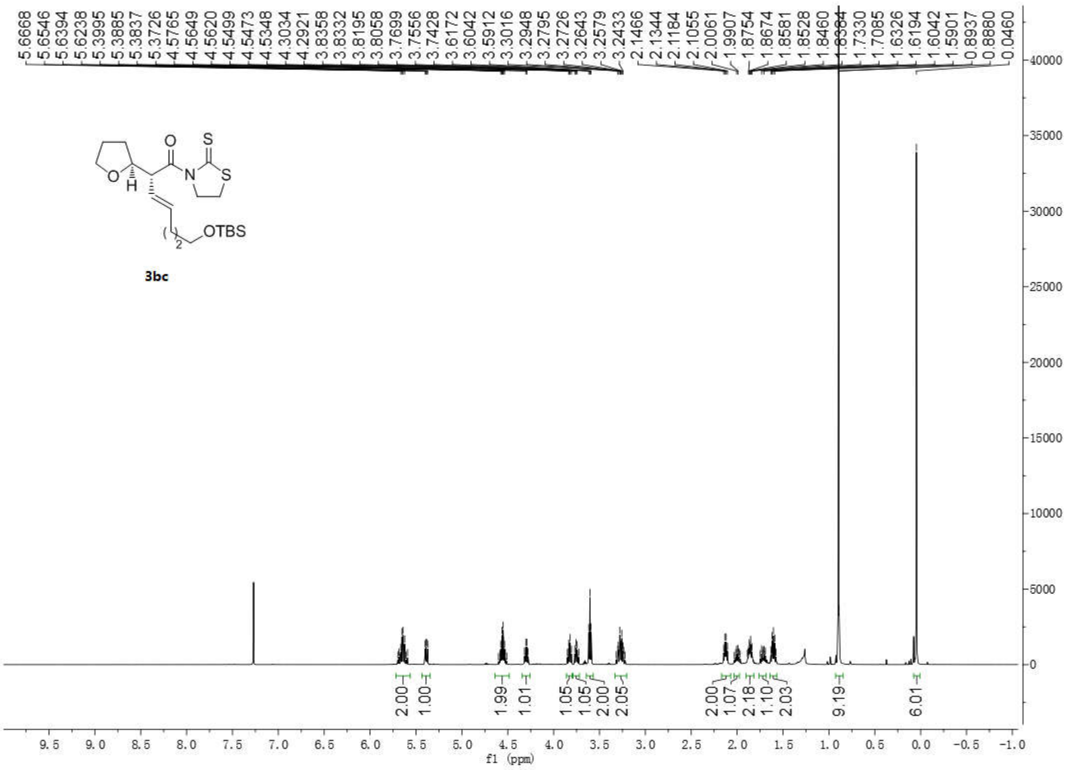

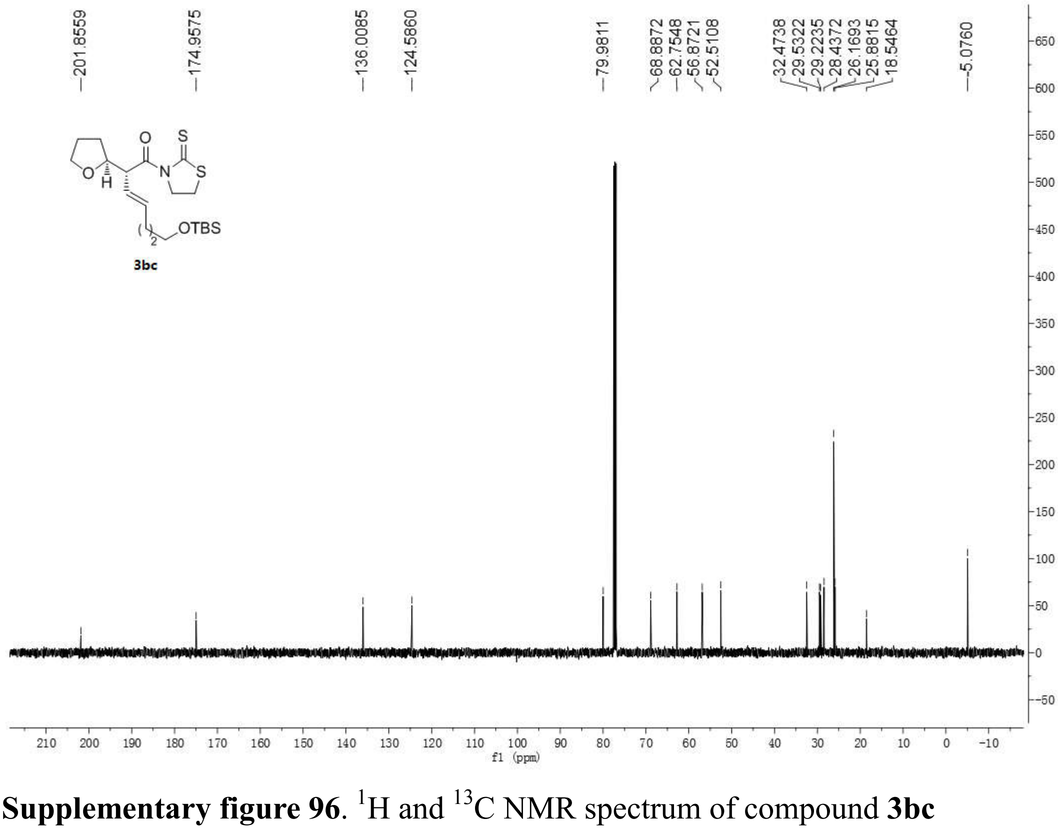

Supplementary figure 96. ^{1}H and ^{13}C NMR spectrum of compound **3bc**

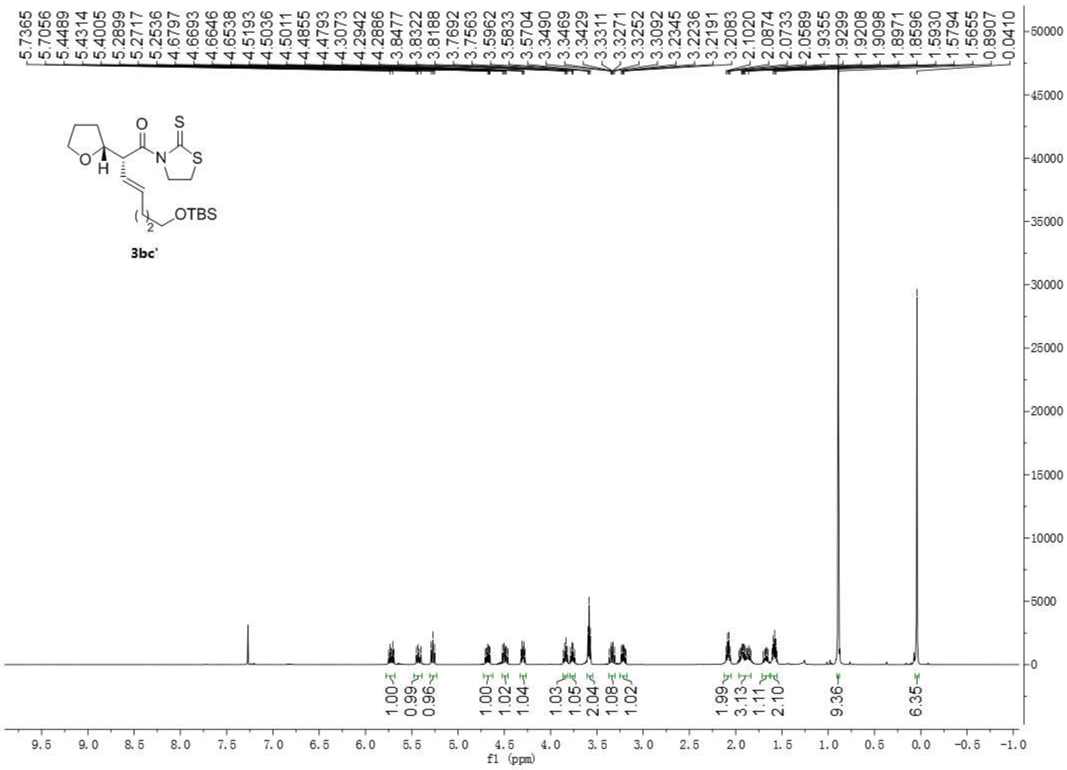

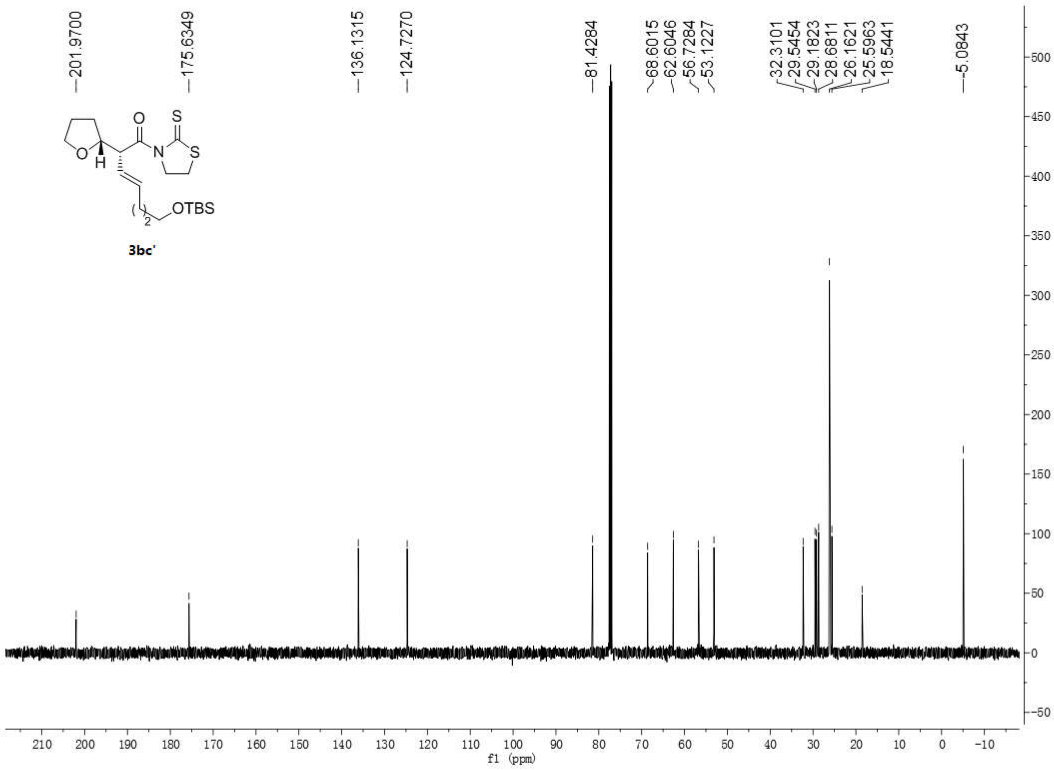

Supplementary figure 97. ^{1}H and ^{13}C NMR spectrum of compound **3bc'**

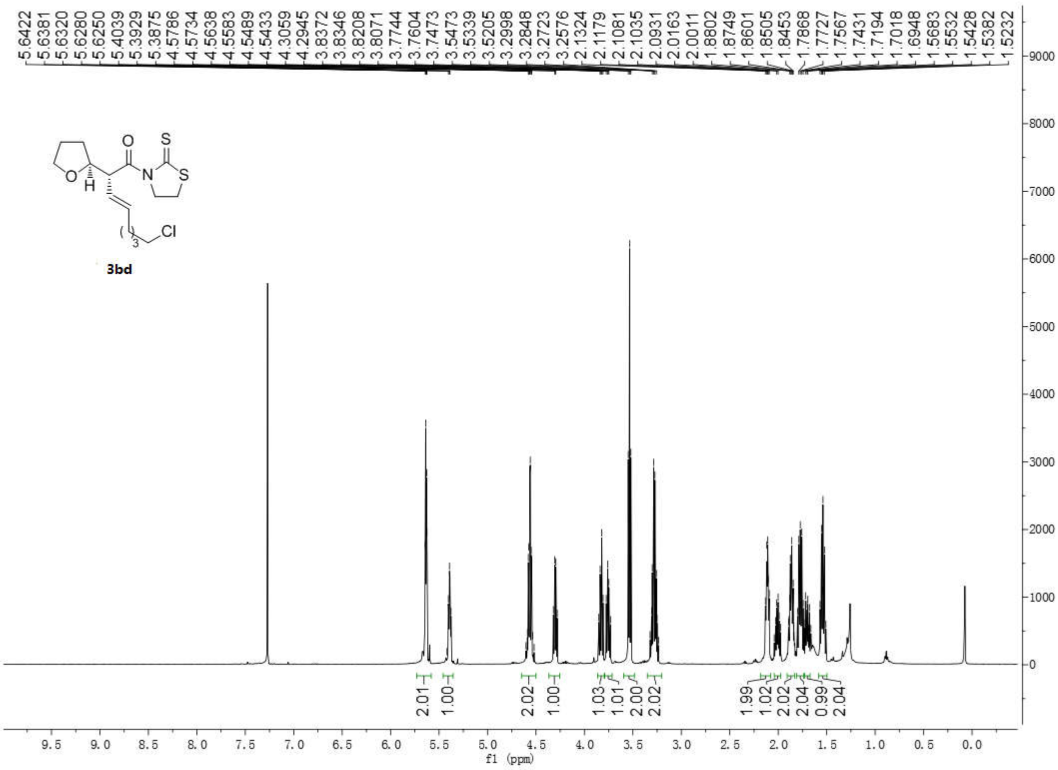

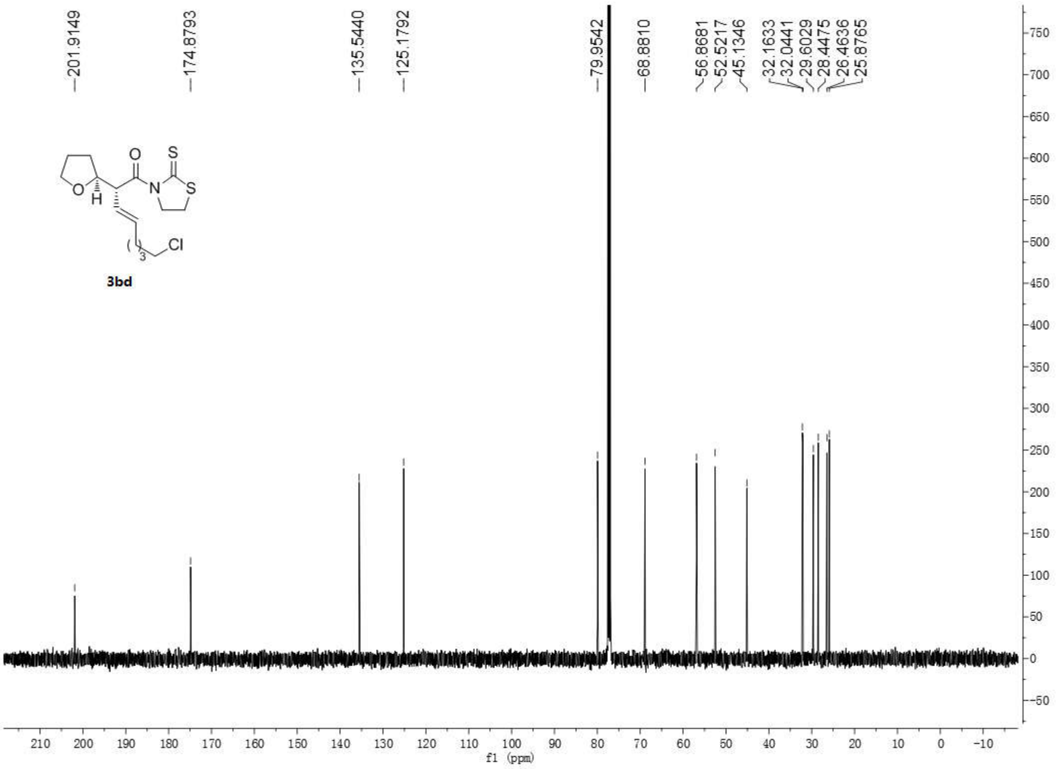

Supplementary figure 98. ^{1}H and ^{13}C NMR spectrum of compound **3bd**

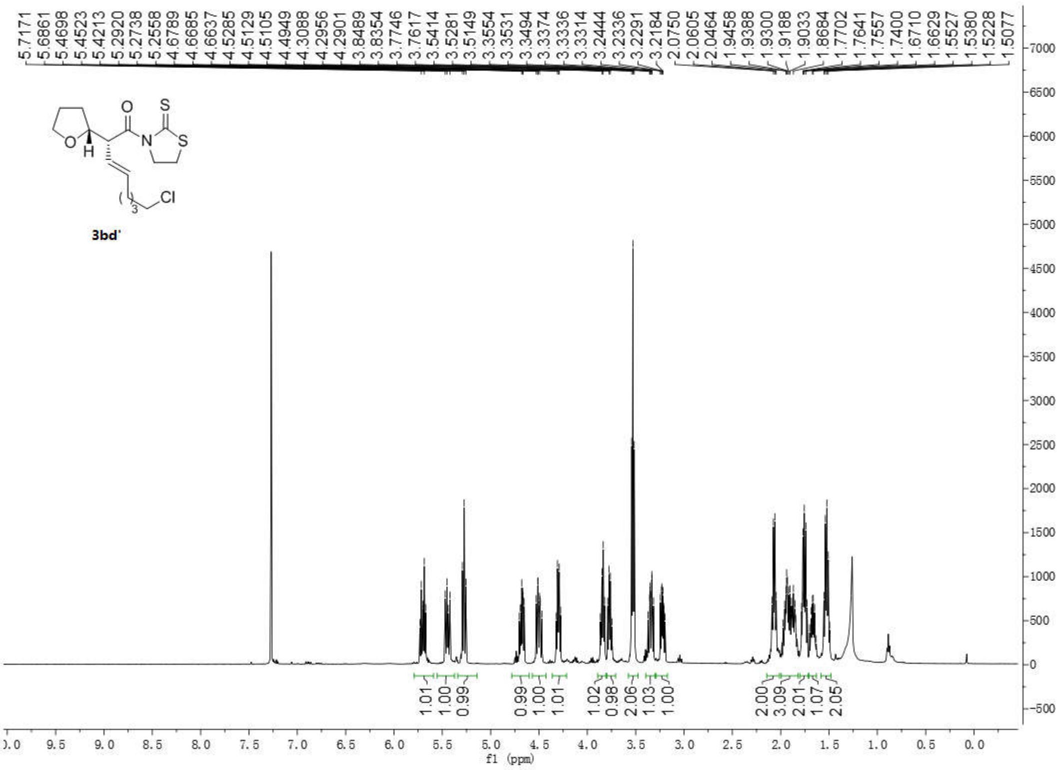

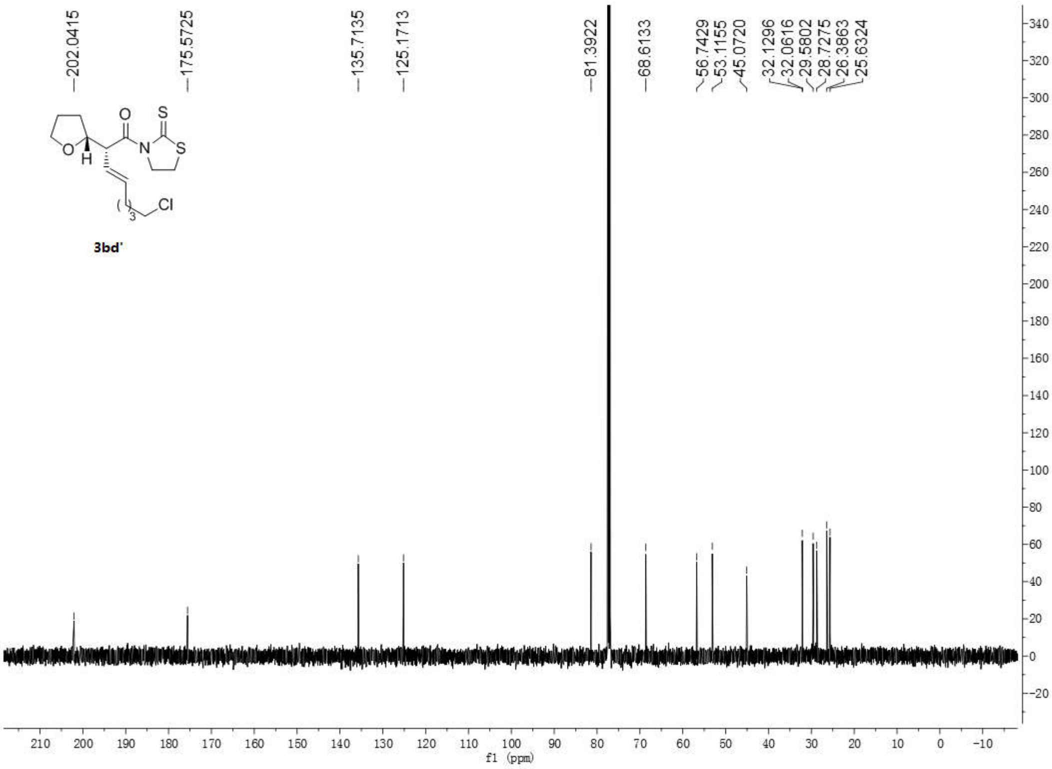

Supplementary figure 99. ^{1}H and ^{13}C NMR spectrum of compound **3bd’**

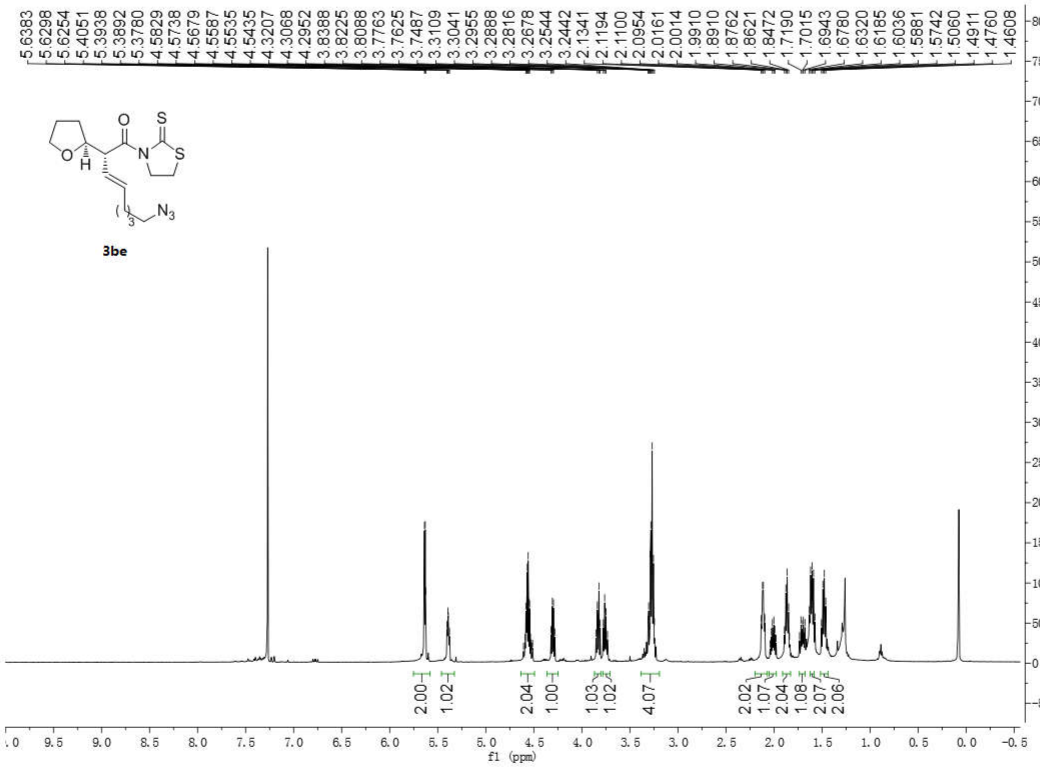

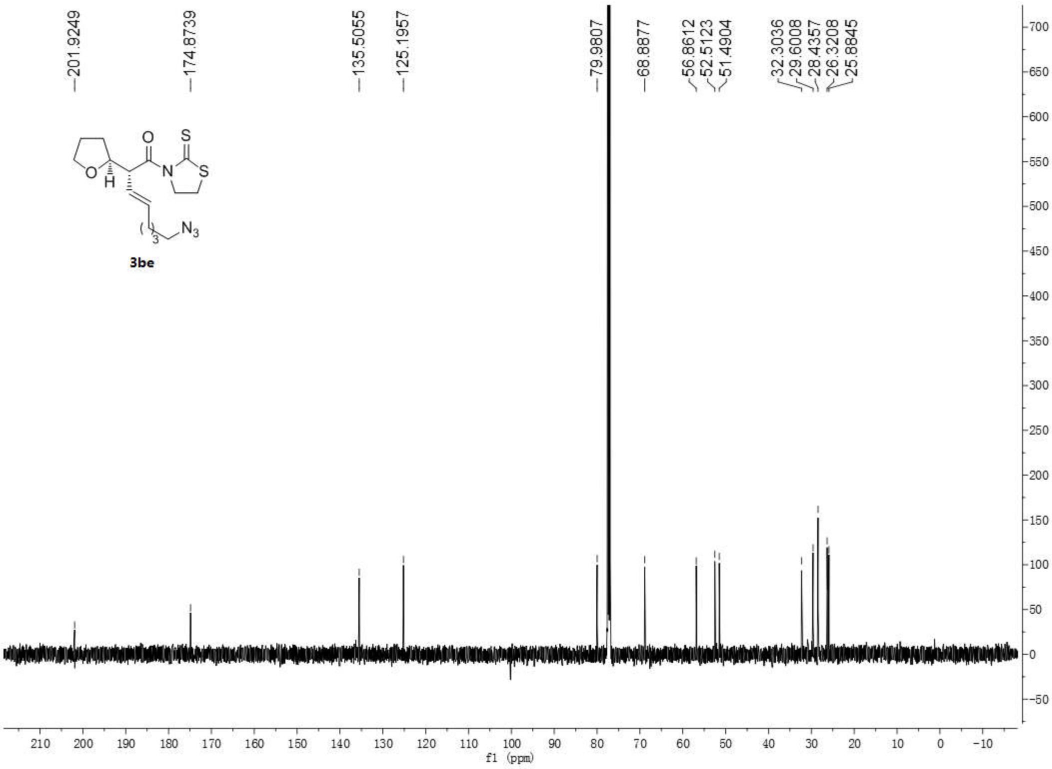

Supplementary figure 100. ^{1}H and ^{13}C NMR spectrum of compound **3be**

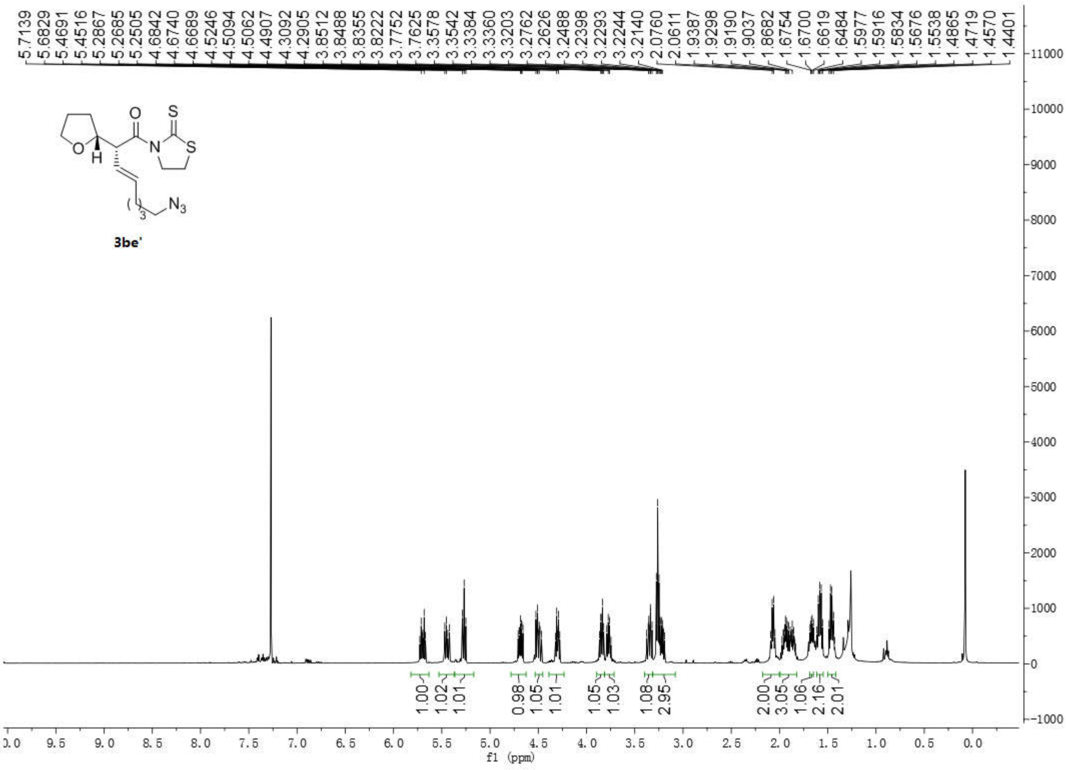

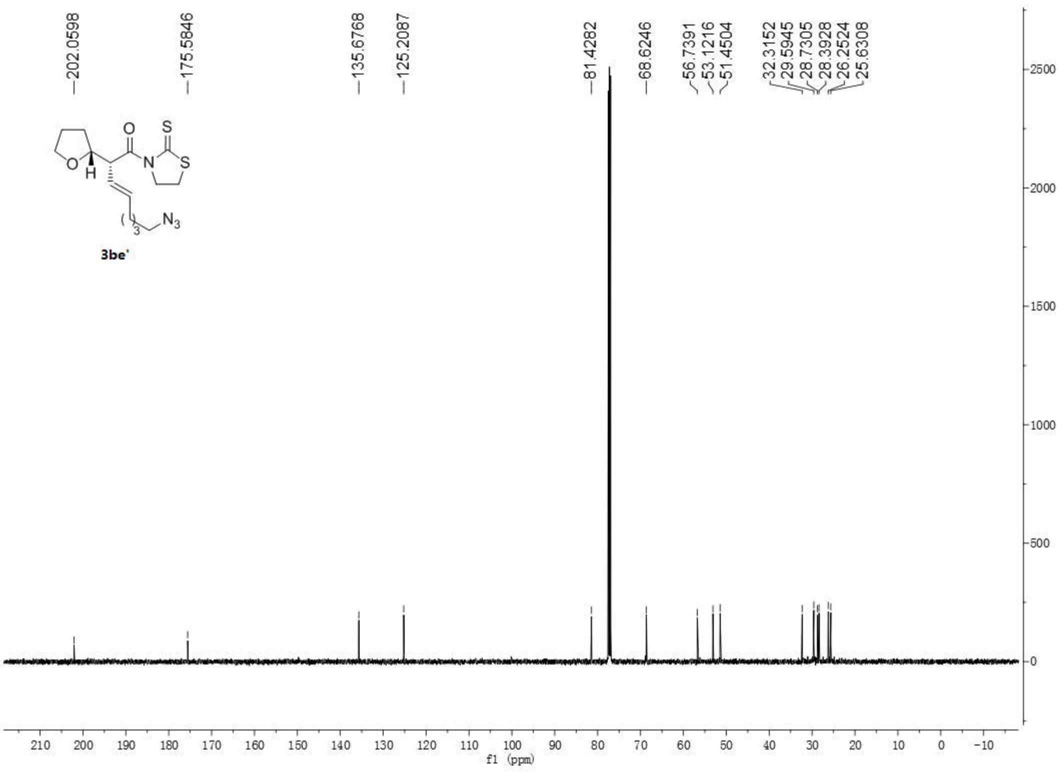

Supplementary figure 101. ^{1}H and ^{13}C NMR spectrum of compound **3be'**

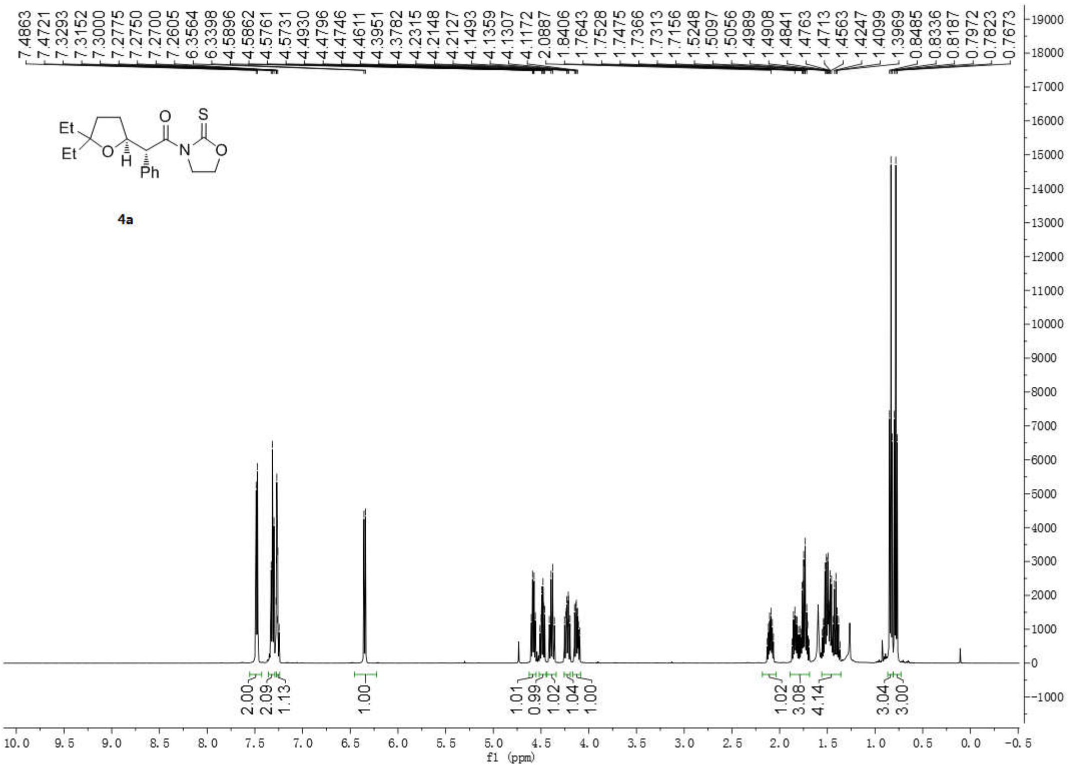

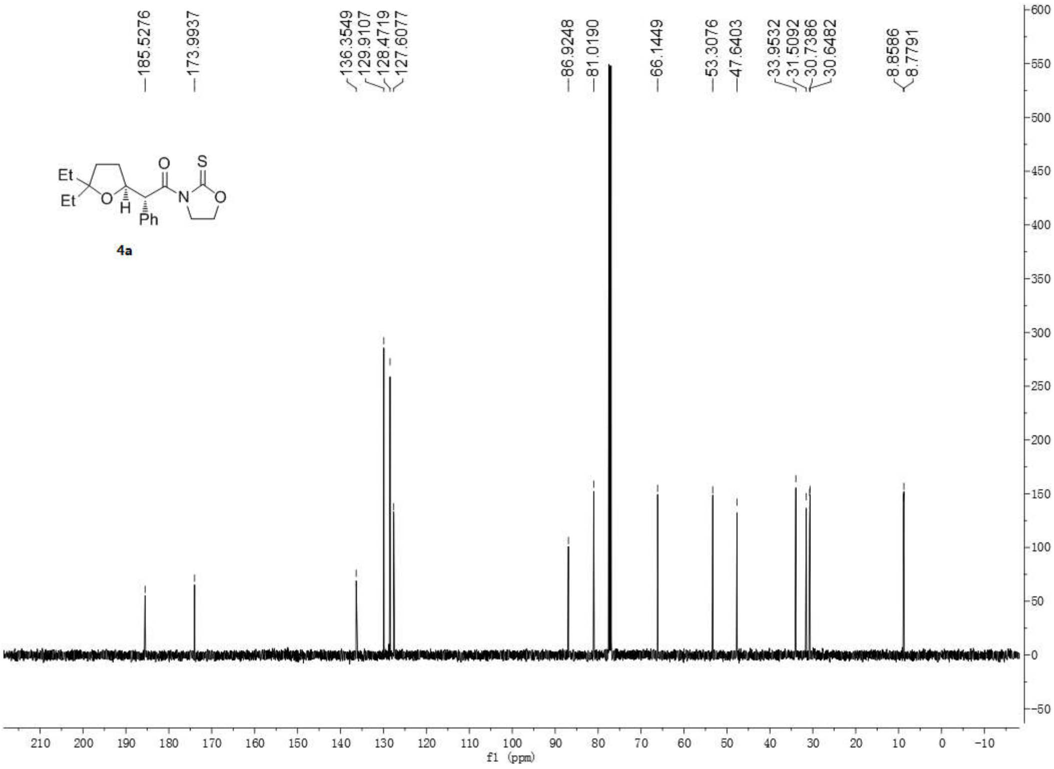

Supplementary figure 102. ^{1}H and ^{13}C NMR spectrum of compound **4a**

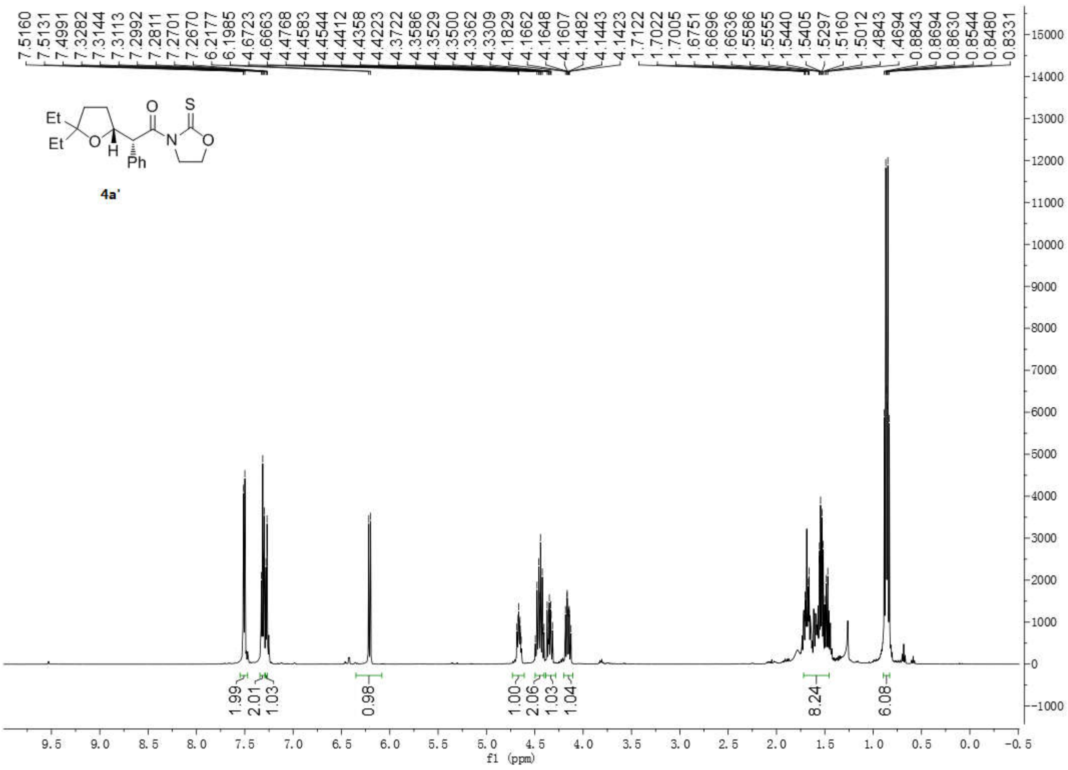

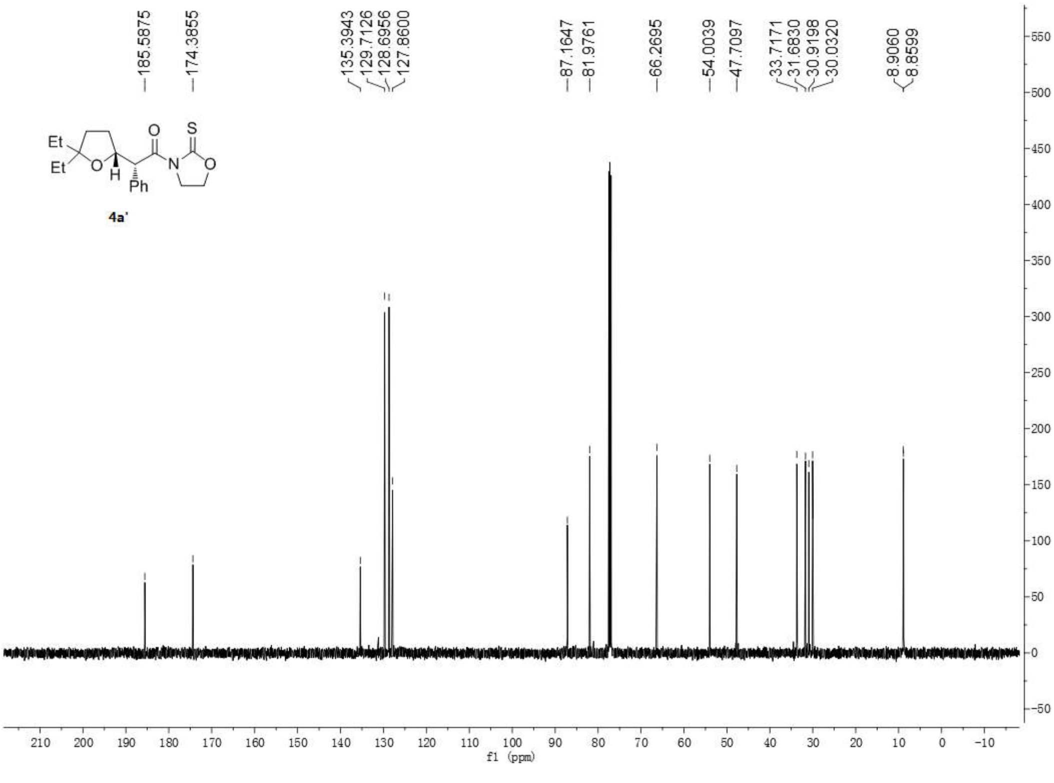

Supplementary figure 103. ^{1}H and ^{13}C NMR spectrum of compound **4a'**

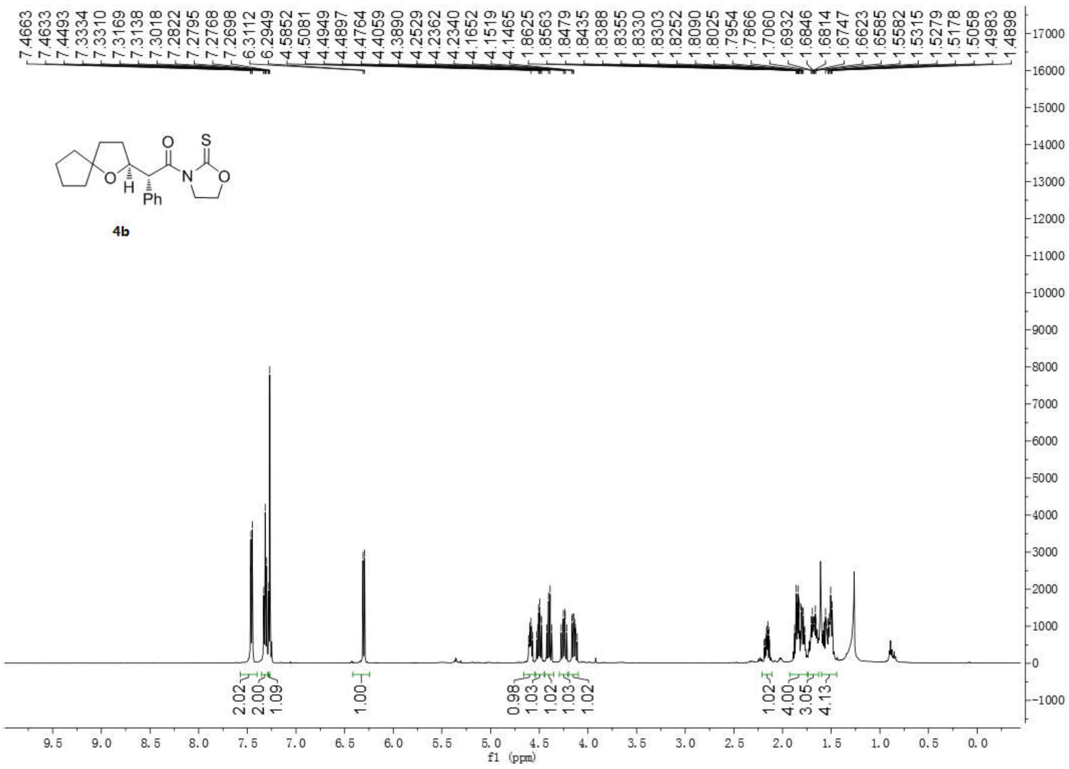

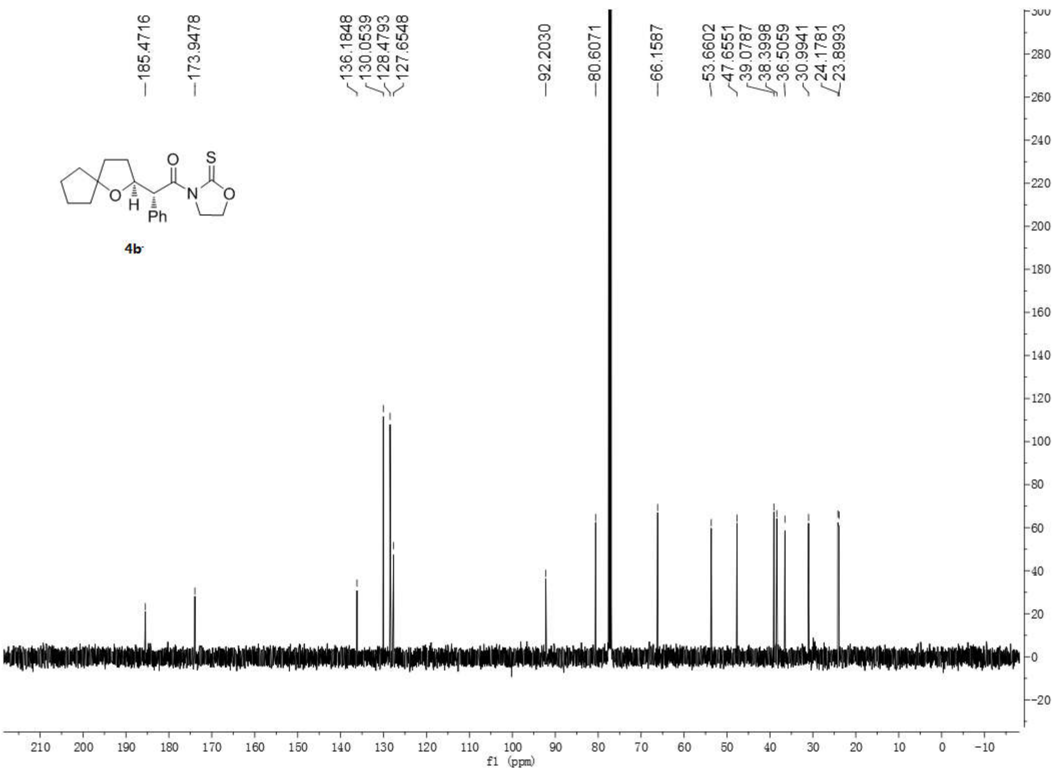

Supplementary figure 104. ^{1}H and ^{13}C NMR spectrum of compound **4b**

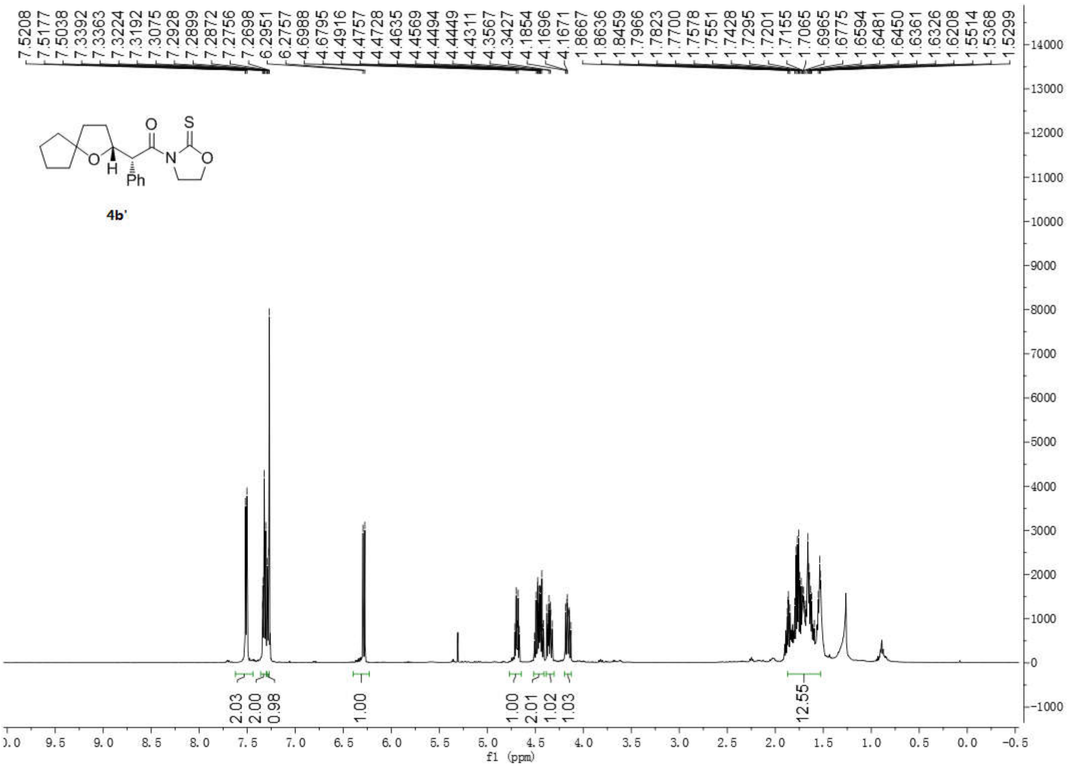

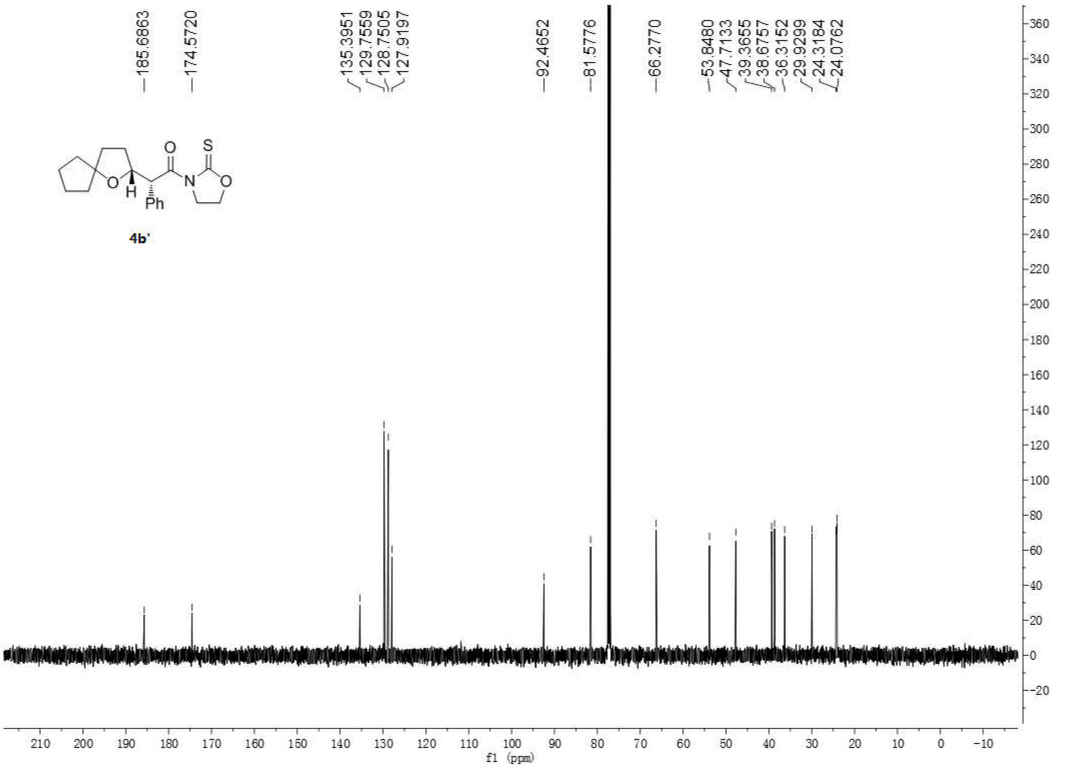

Supplementary figure 105. ^{1}H and ^{13}C NMR spectrum of compound **4b’**

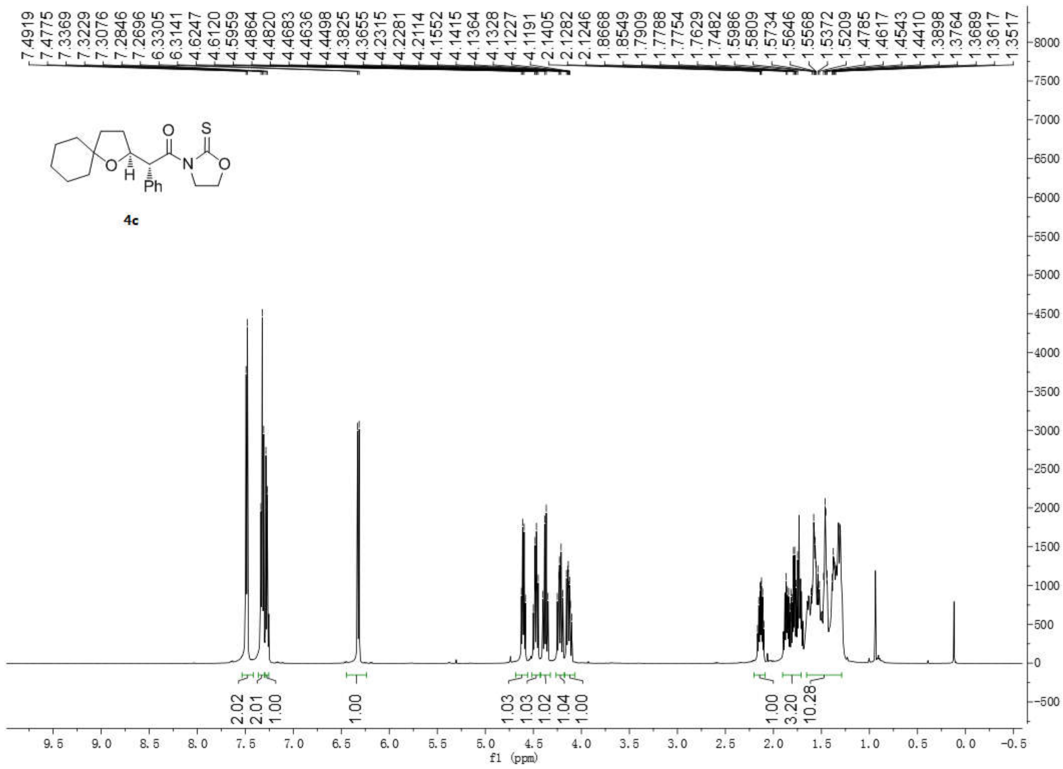

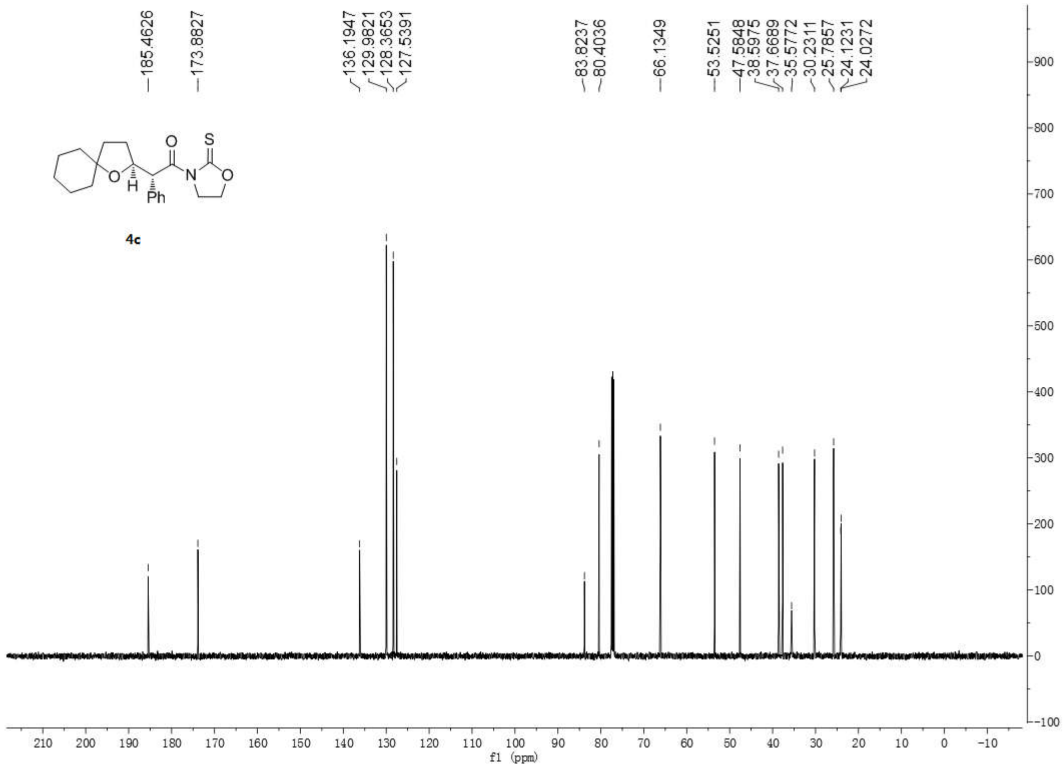

Supplementary figure 106. ^{1}H and ^{13}C NMR spectrum of compound **4c**

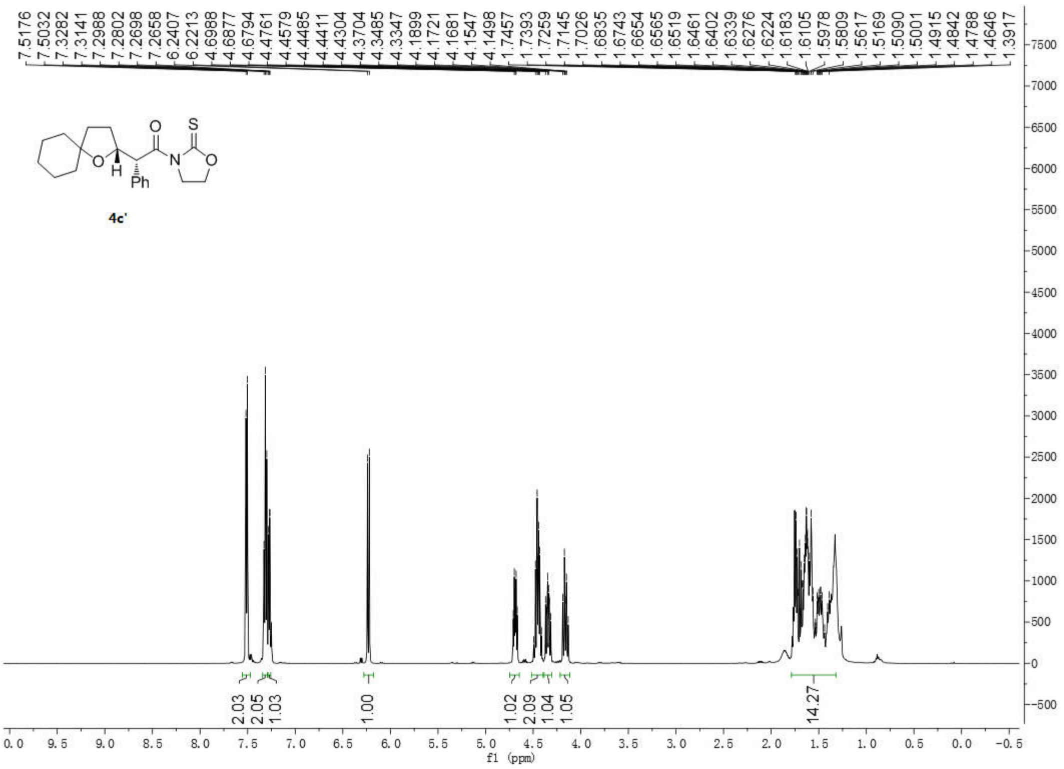

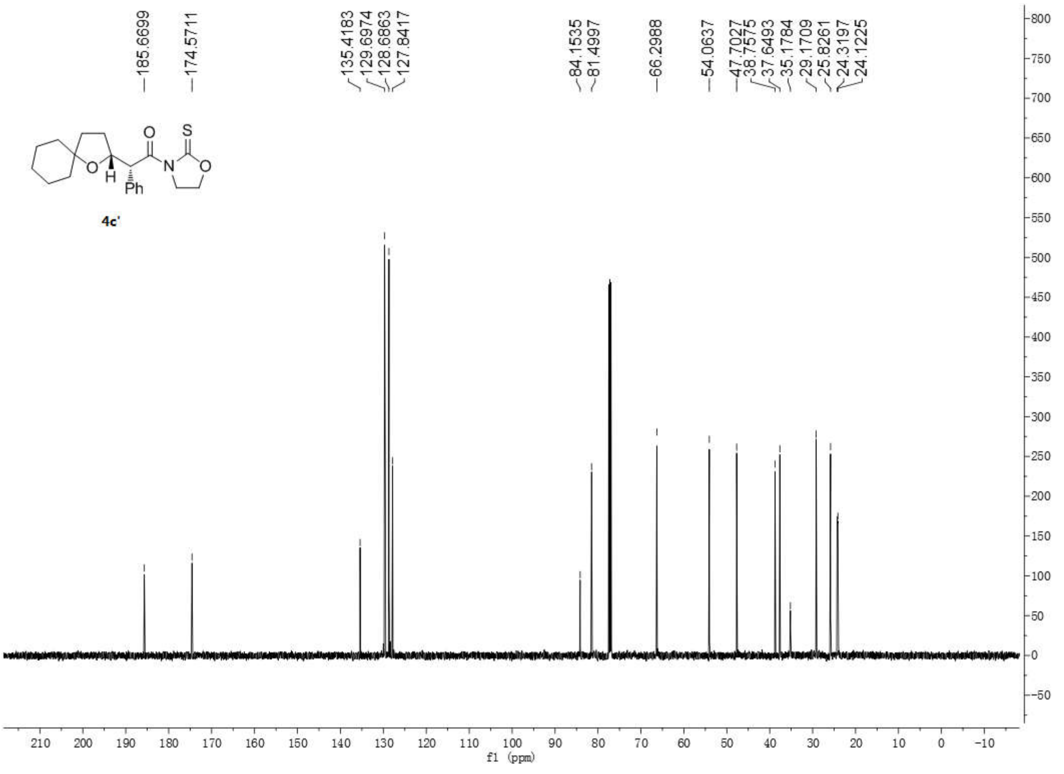

Supplementary figure 107. ^{1}H and ^{13}C NMR spectrum of compound **4c'**

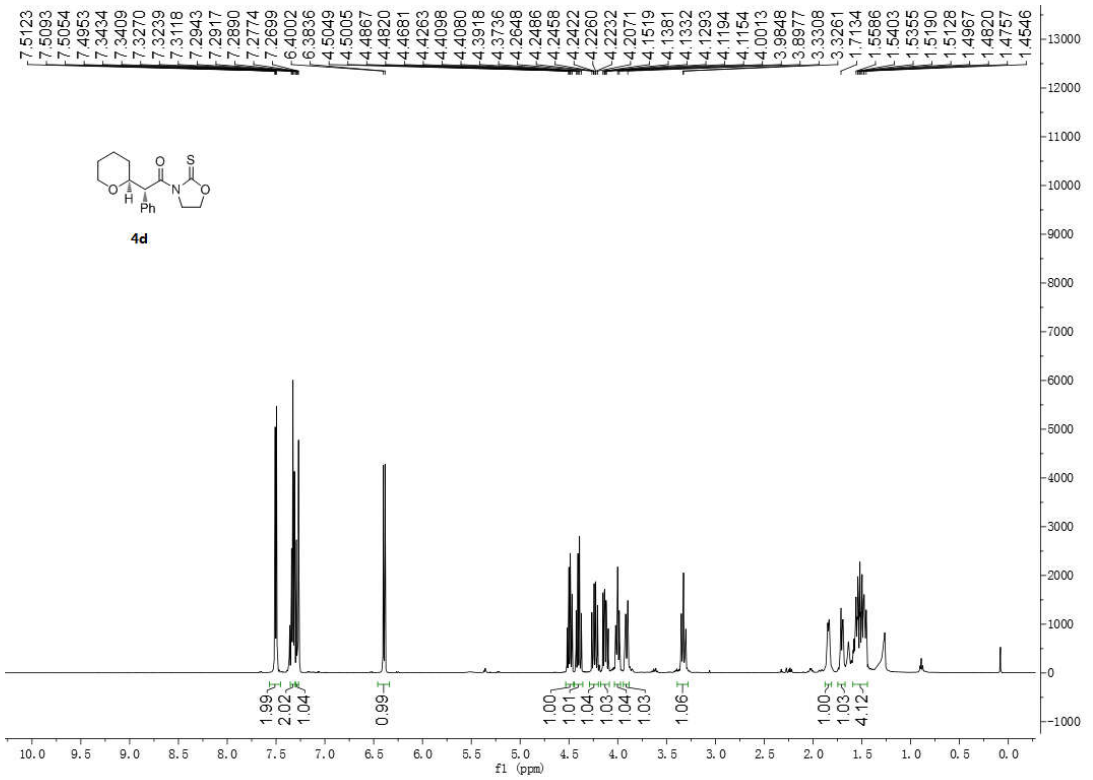

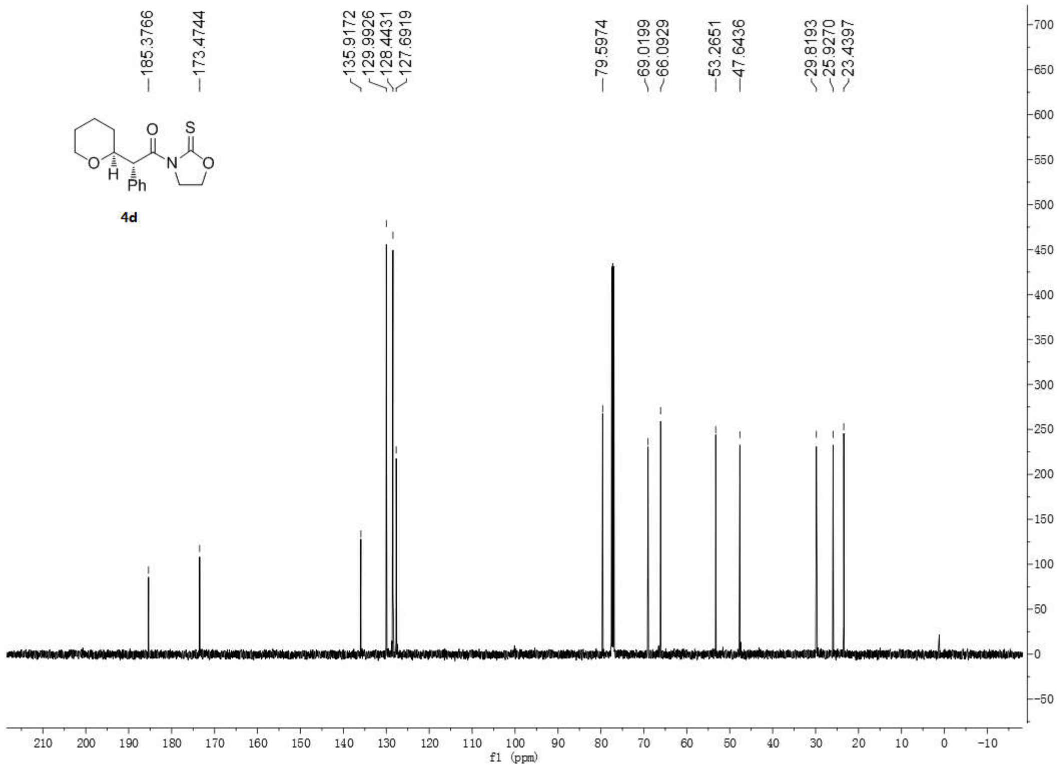

Supplementary figure 108. ^{1}H and ^{13}C NMR spectrum of compound **4d**

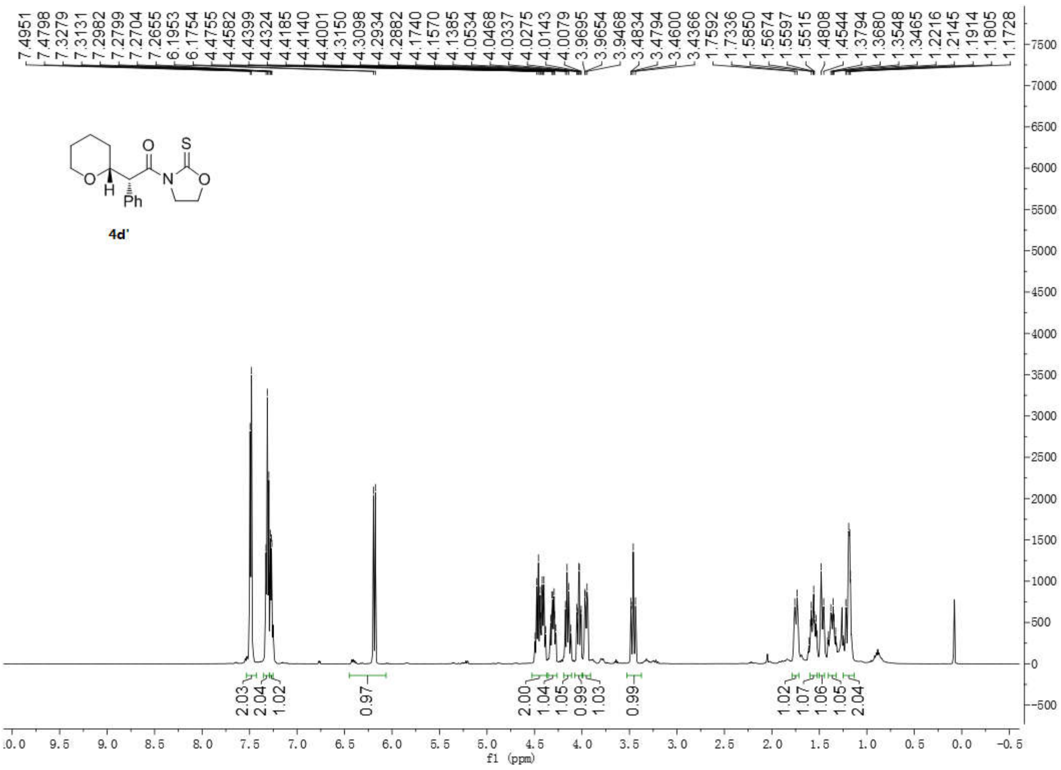

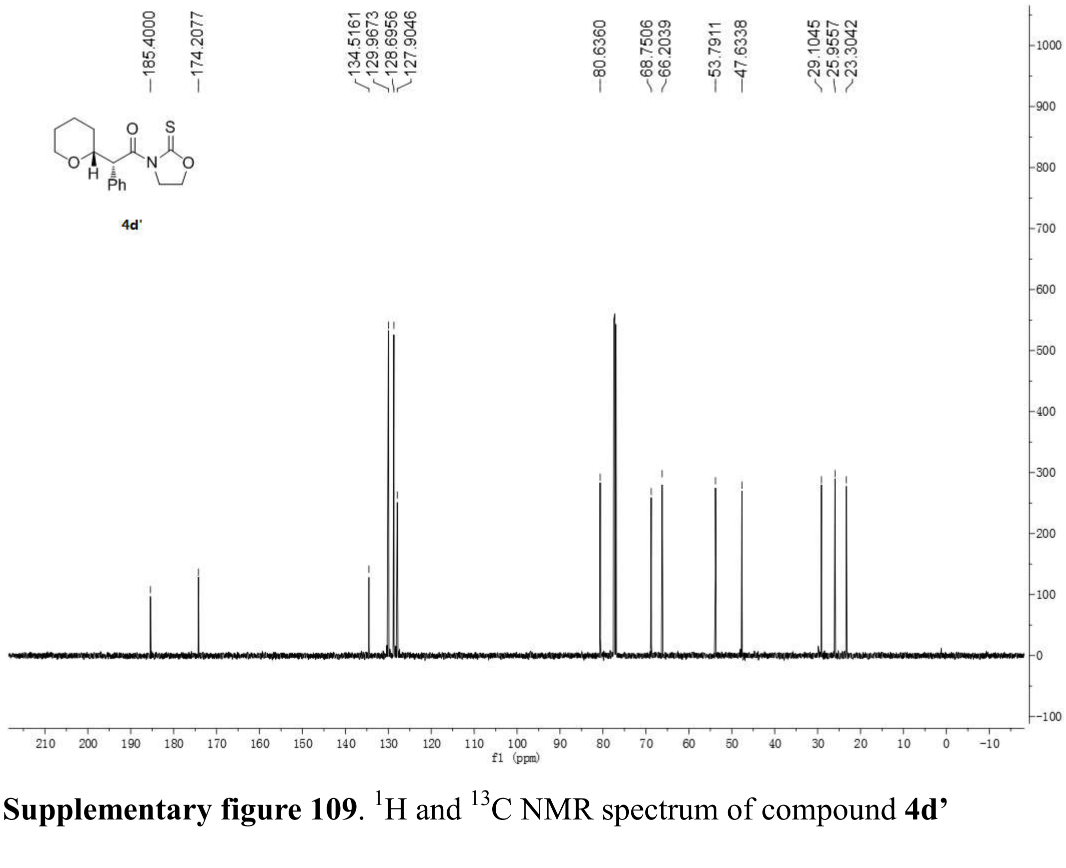

Supplementary figure 109. ^{1}H and ^{13}C NMR spectrum of compound **4d’**

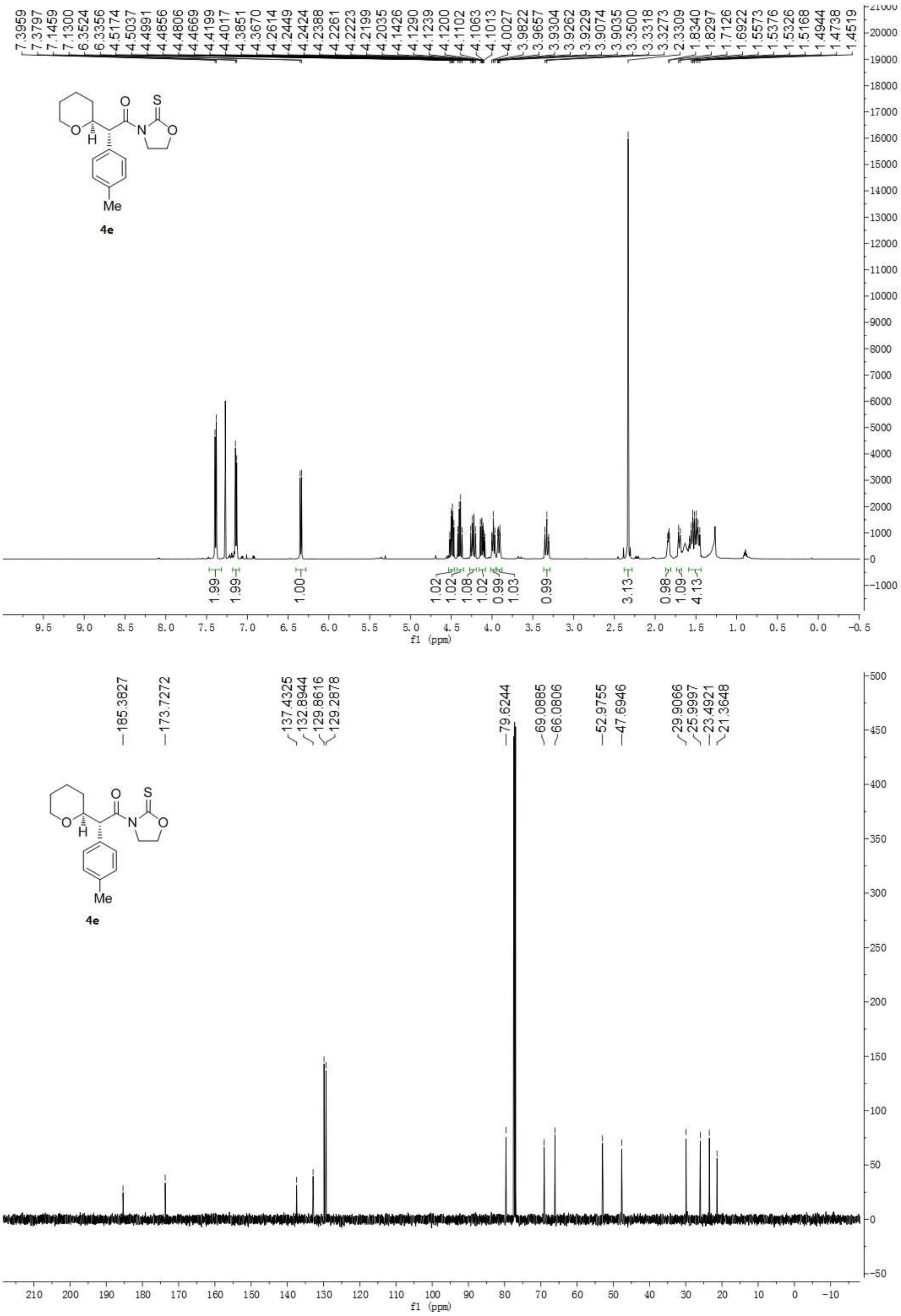

Supplementary figure 110. ^{1}H and ^{13}C NMR spectrum of compound **4e**

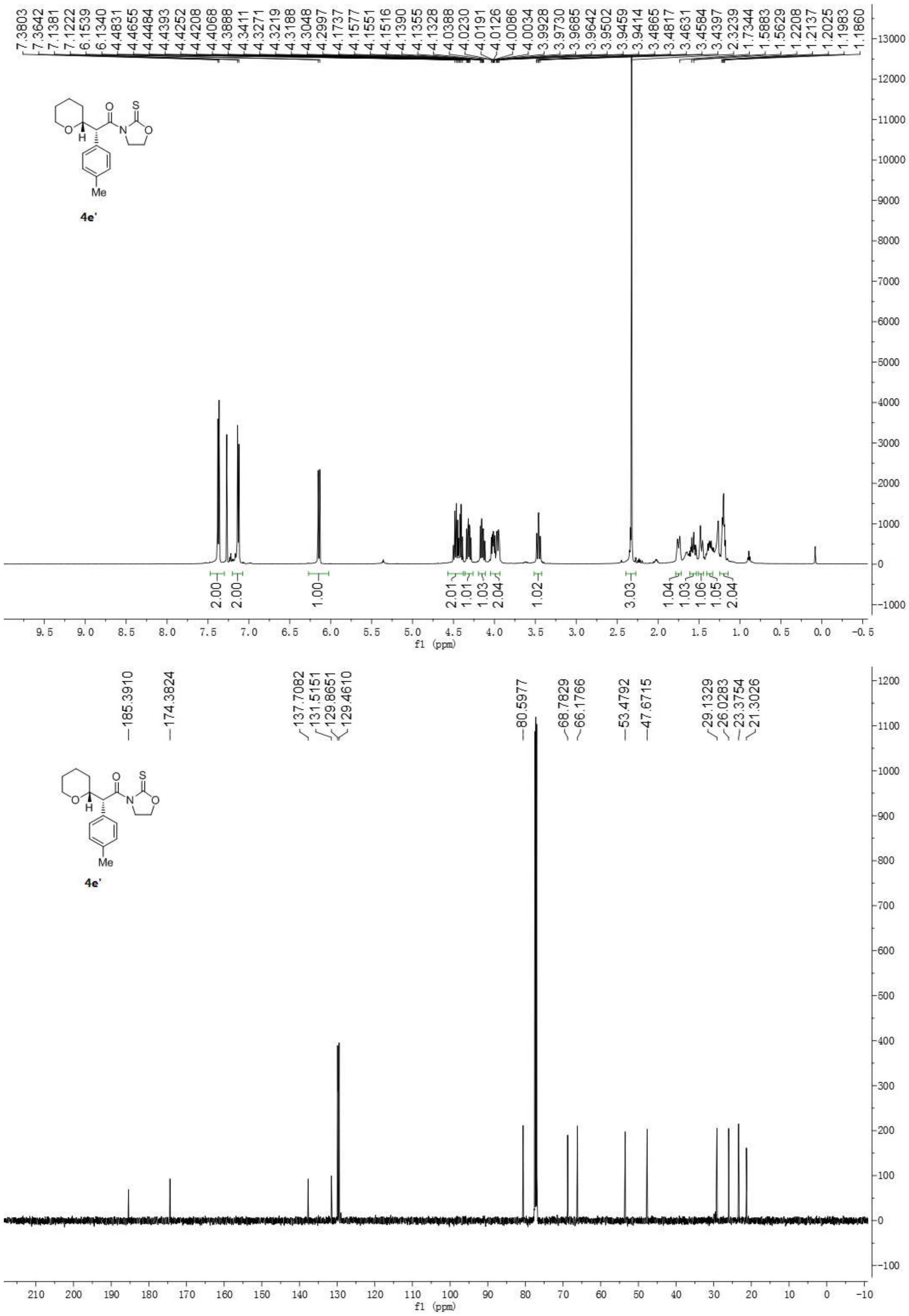

Supplementary figure 111. ^{1}H and ^{13}C NMR spectrum of compound **4e'**

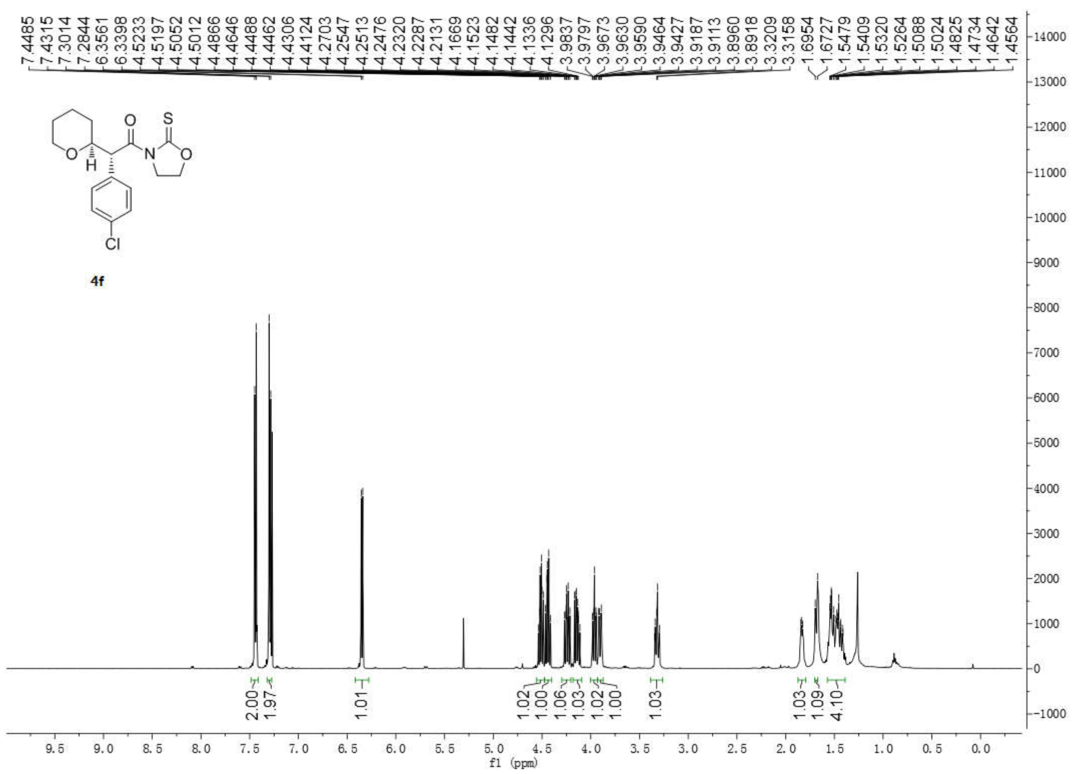

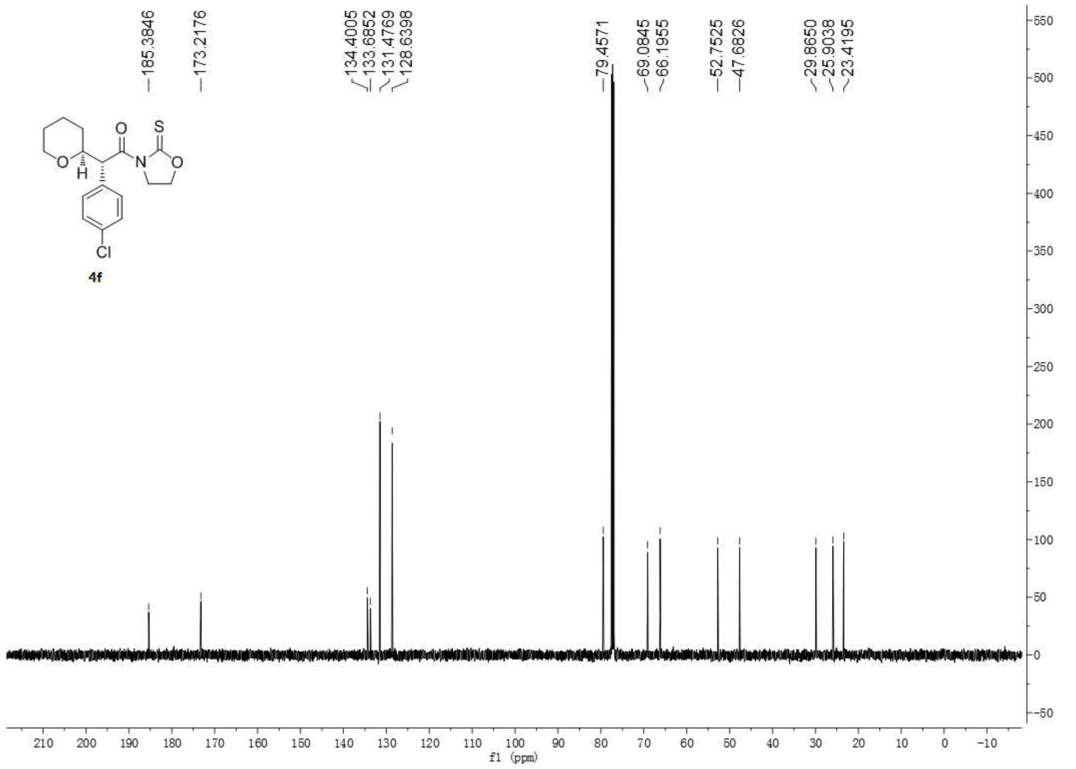

Supplementary figure 112. ^{1}H and ^{13}C NMR spectrum of compound **4f**

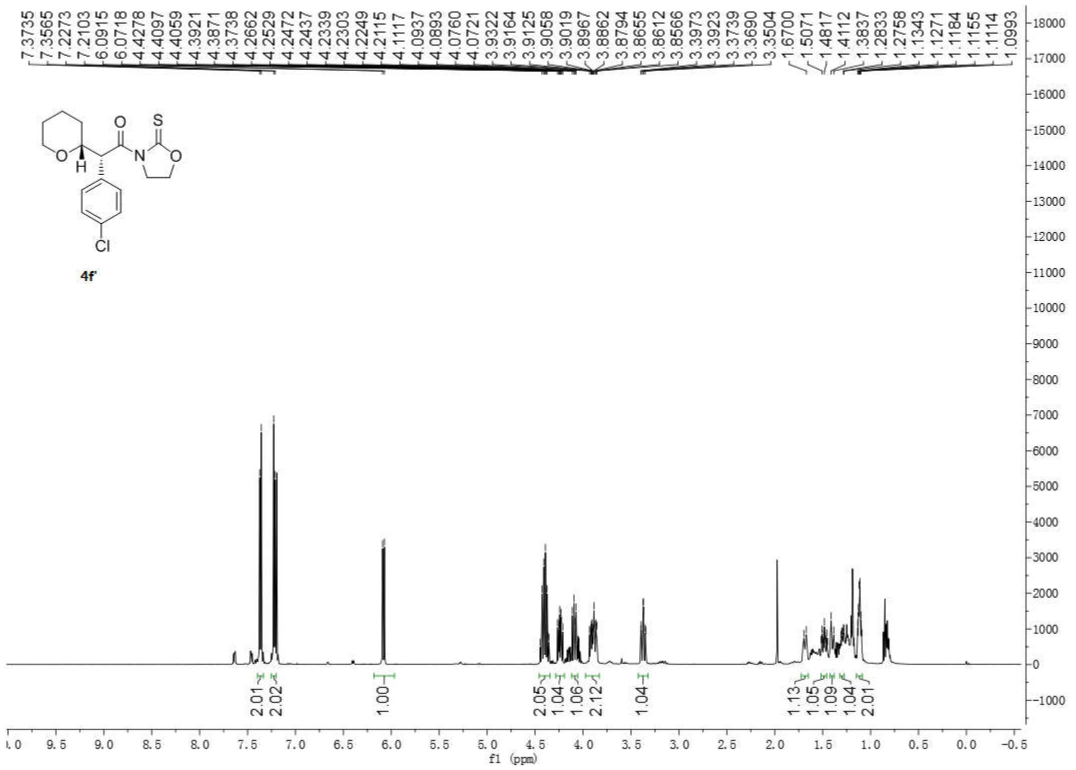

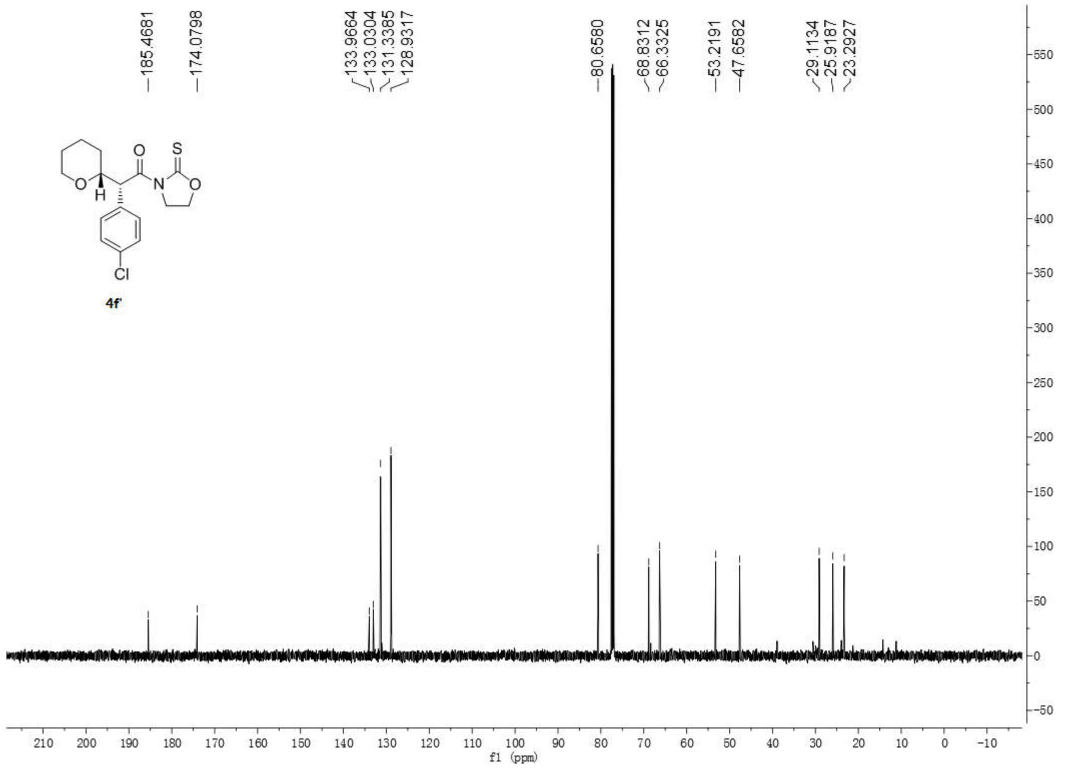

Supplementary figure 113. ^{1}H and ^{13}C NMR spectrum of compound **4f'**

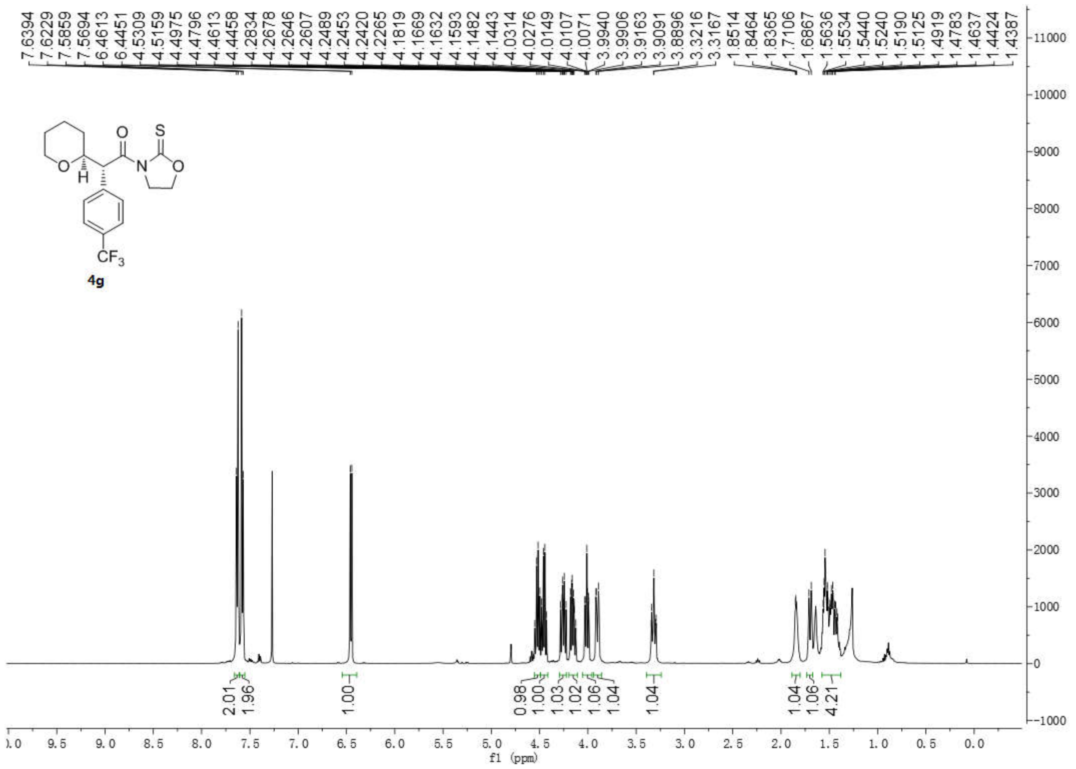

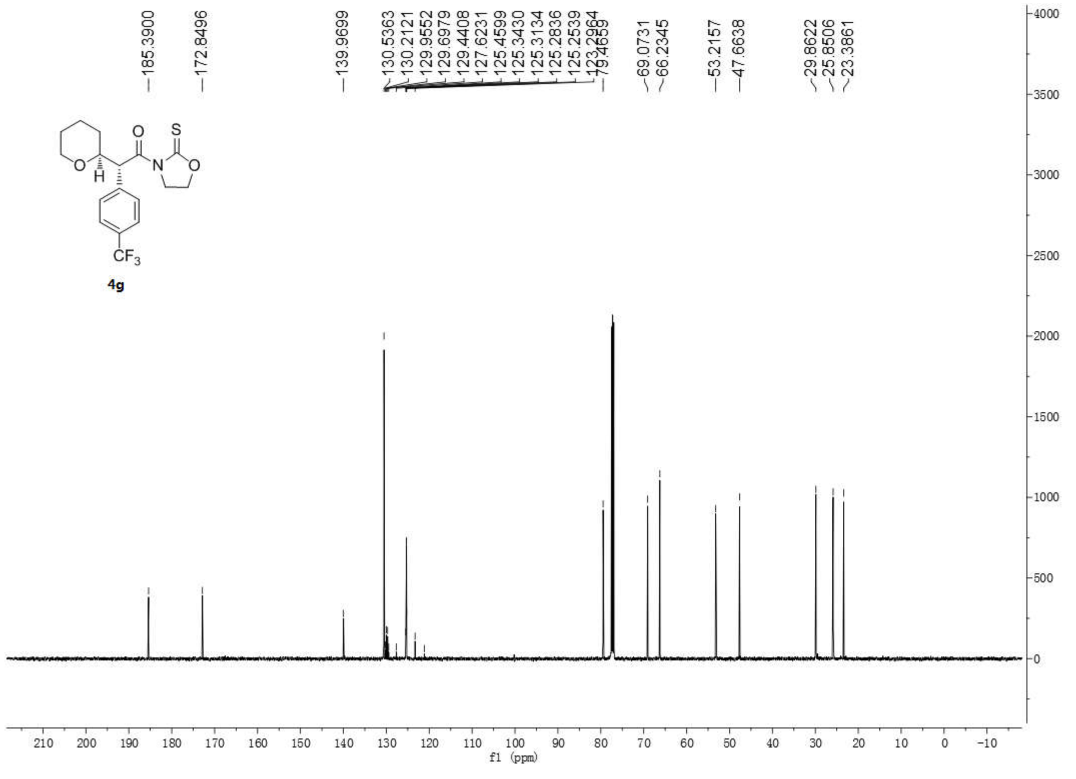

Supplementary figure 114. ^{1}H and ^{13}C NMR spectrum of compound **4g**

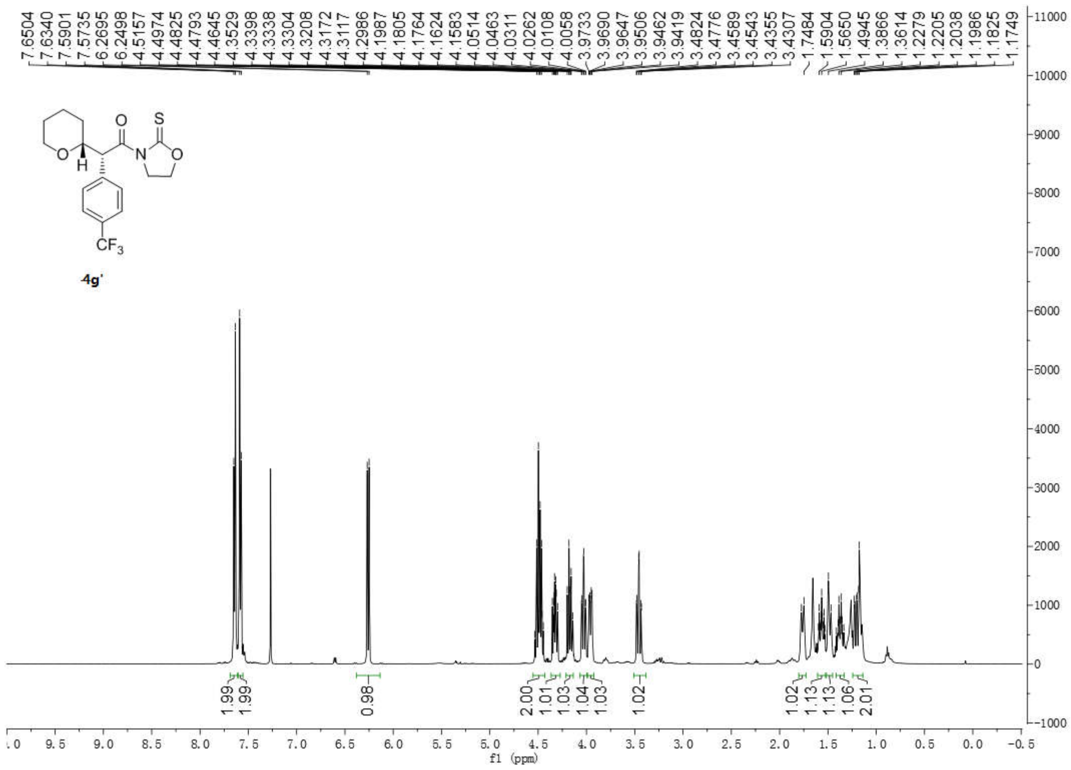

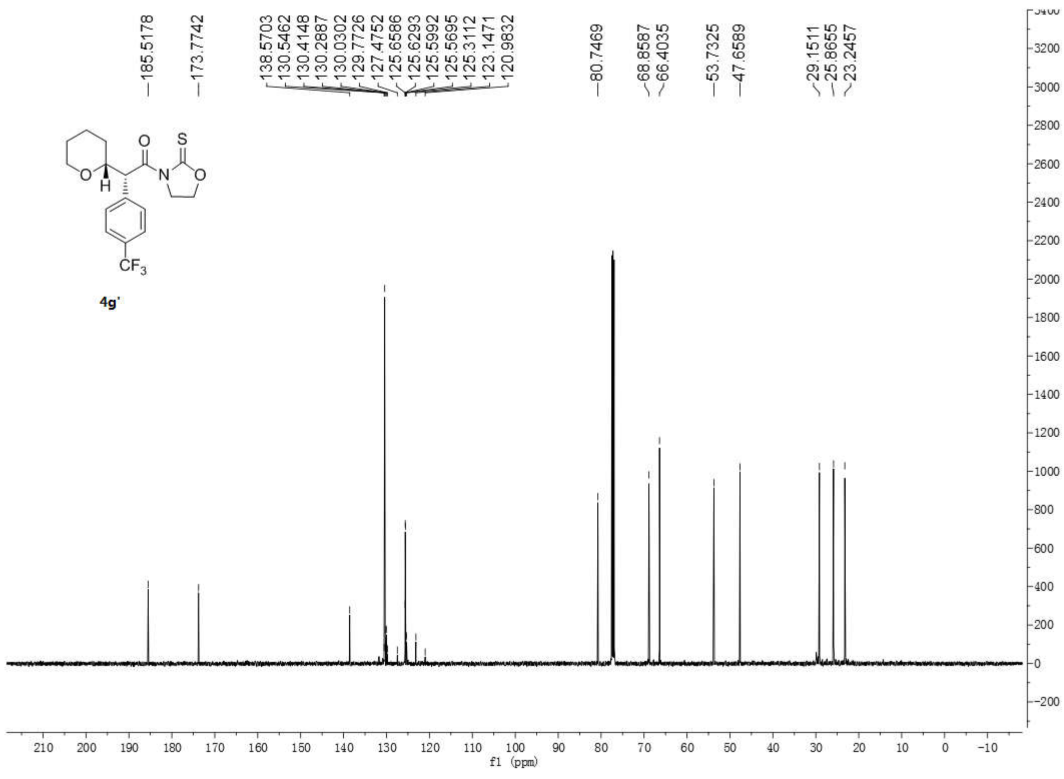

Supplementary figure 115. ^{1}H and ^{13}C NMR spectrum of compound **4g'**

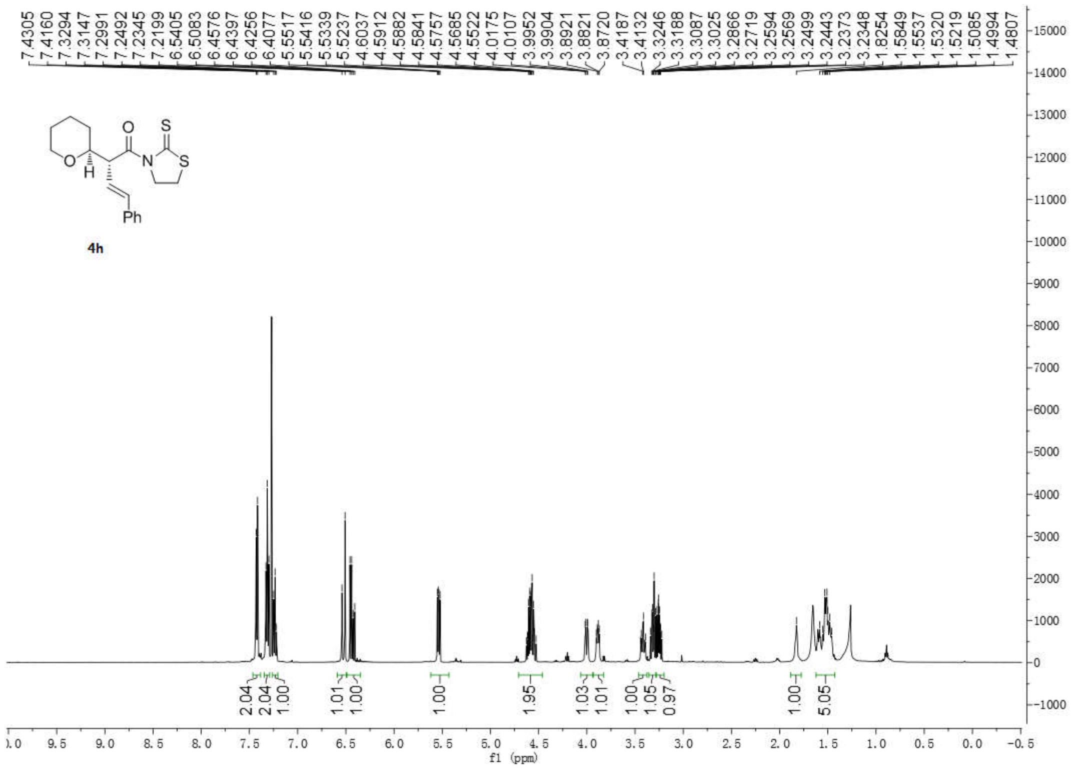

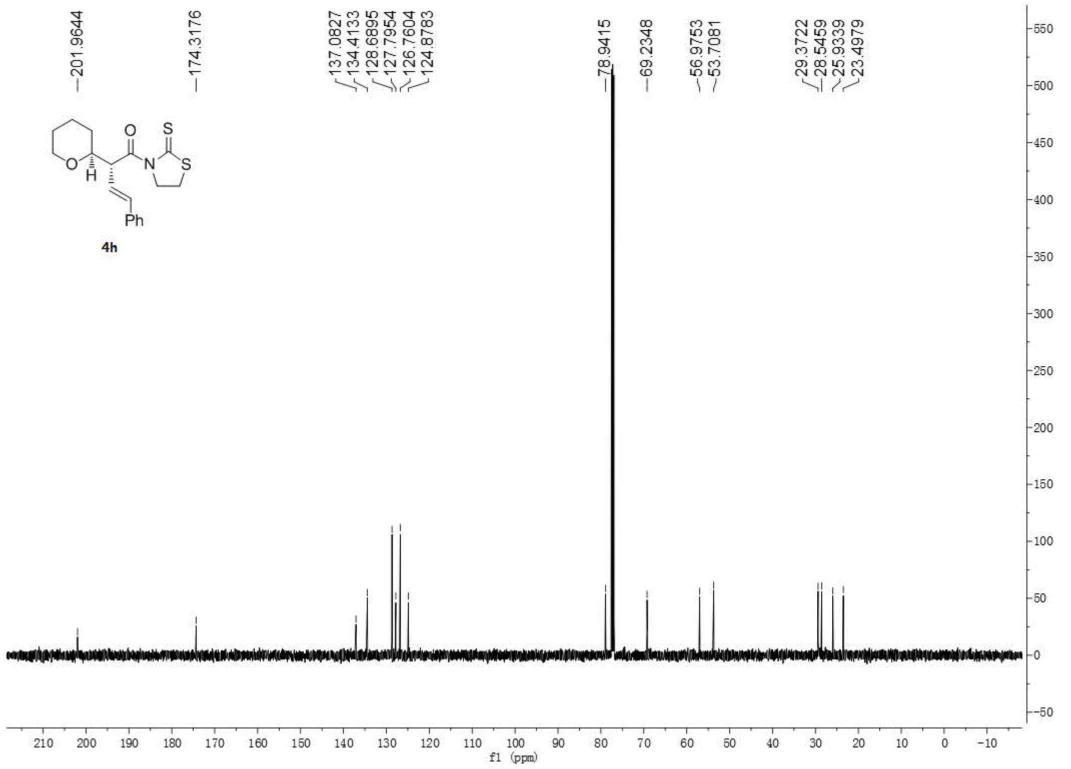

Supplementary figure 116. ^{1}H and ^{13}C NMR spectrum of compound **4h**

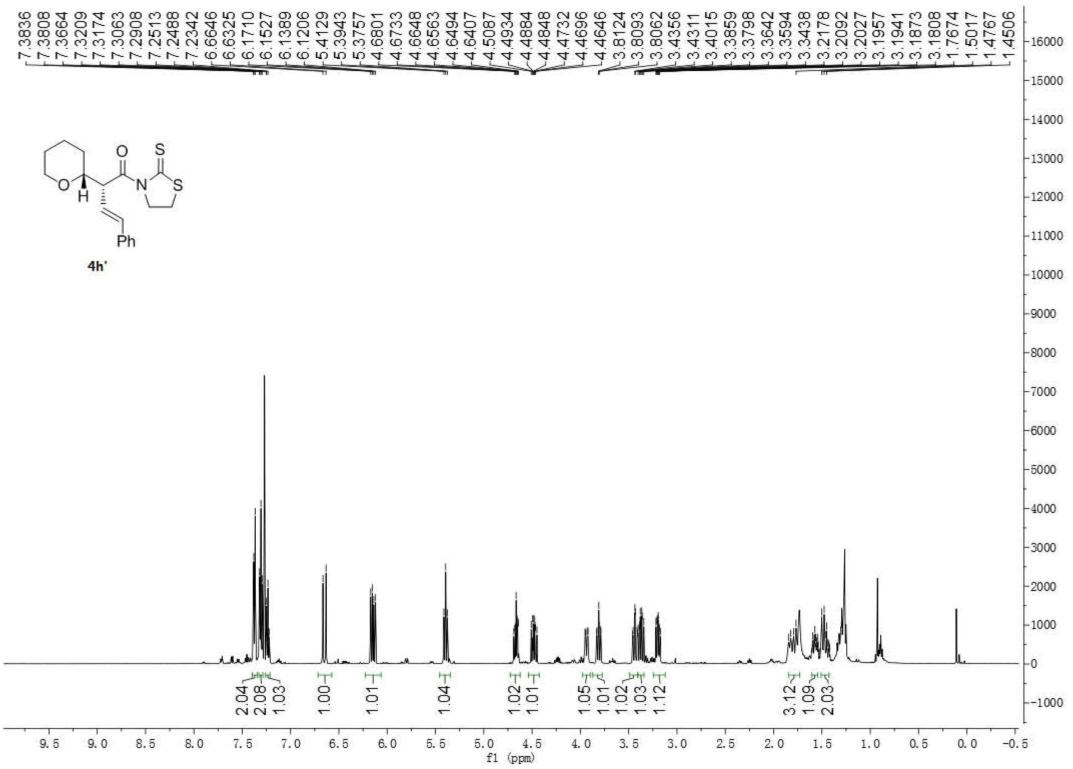

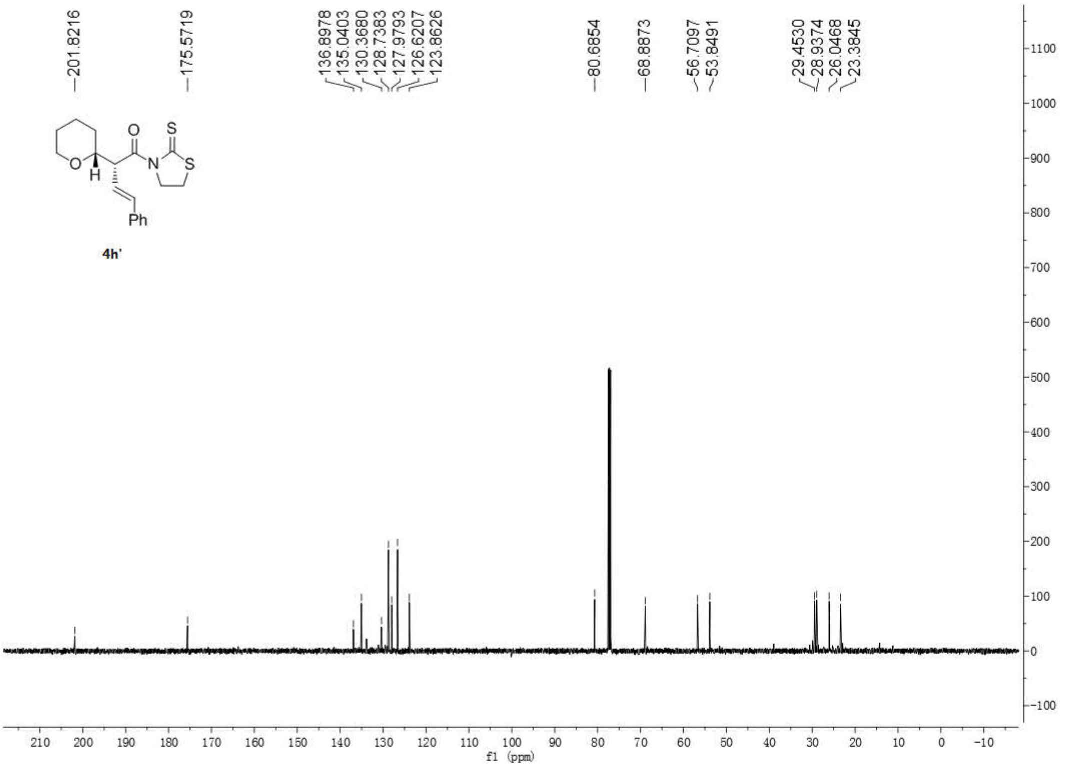

Supplementary figure 117. ^{1}H and ^{13}C NMR spectrum of compound **4h’**

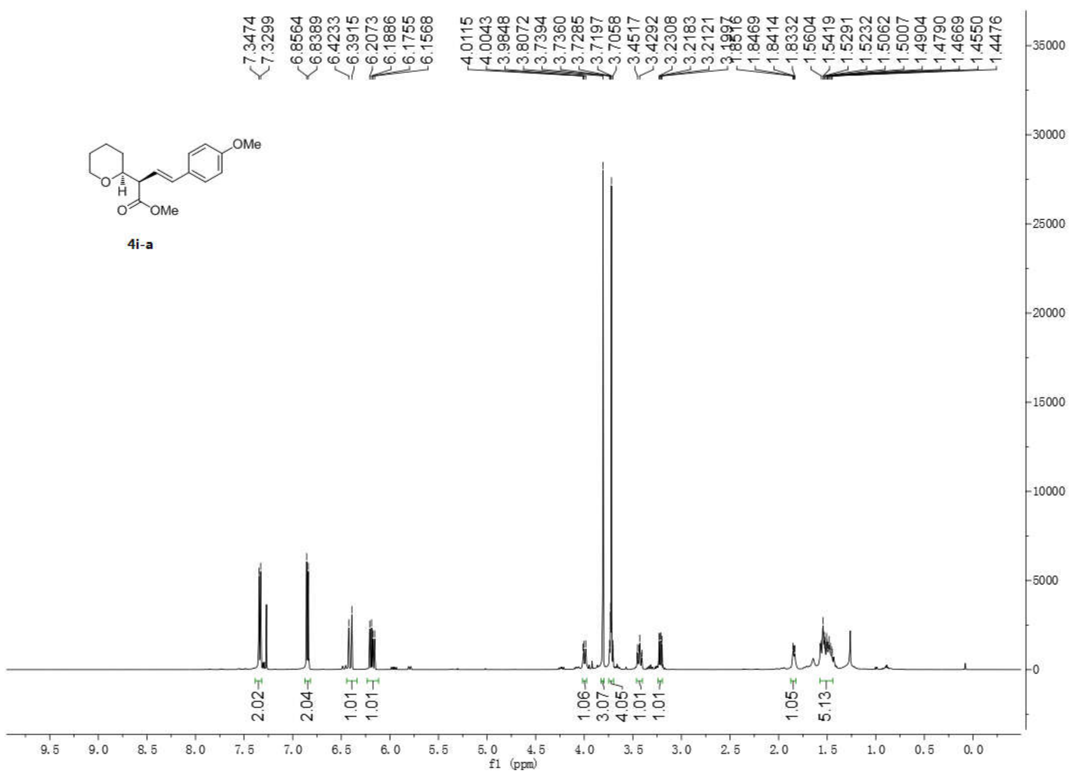

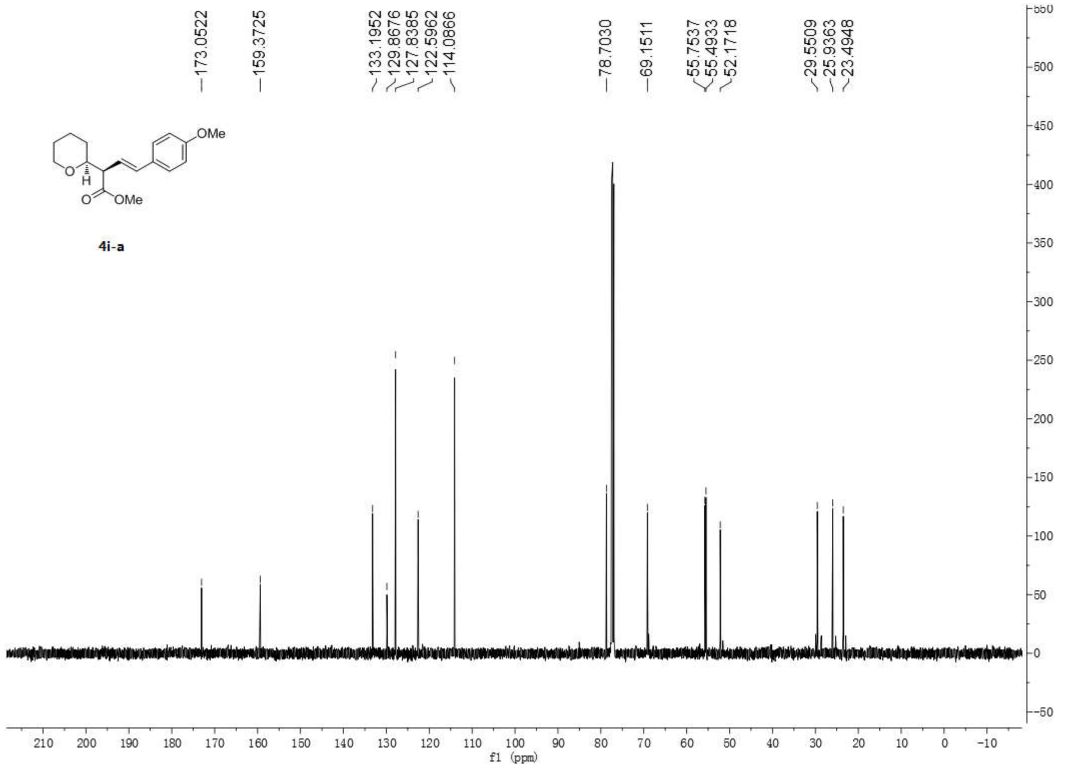

Supplementary figure 118. ^{1}H and ^{13}C NMR spectrum of compound **4i-a**

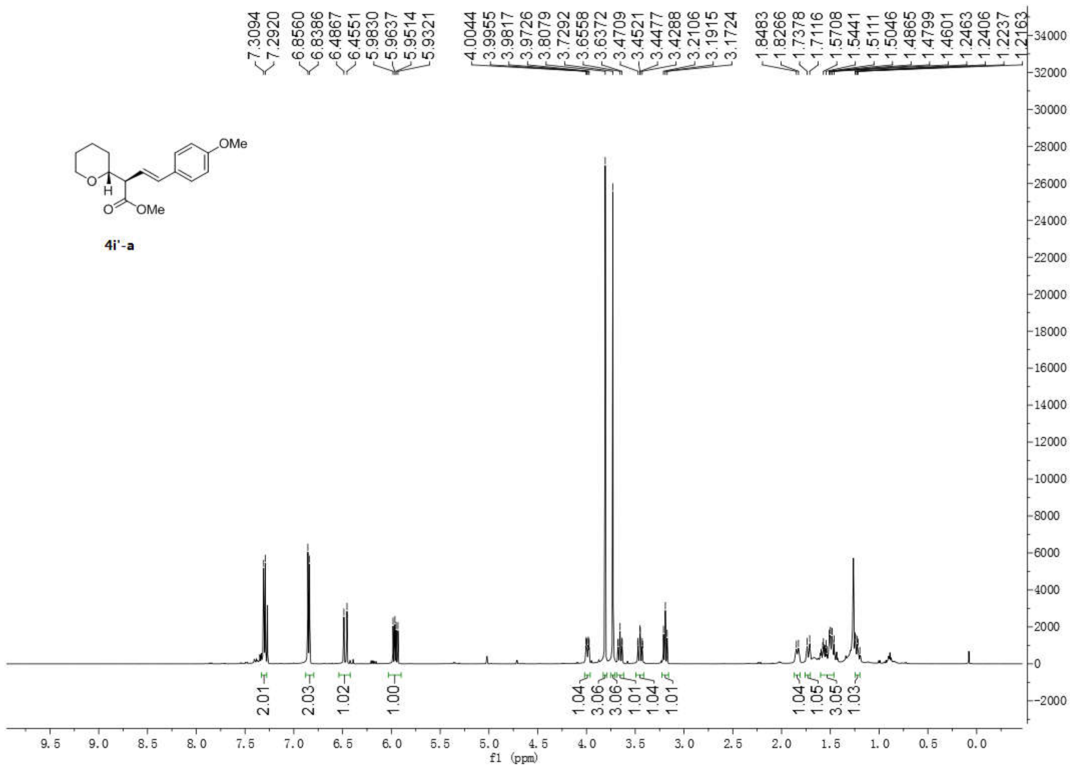

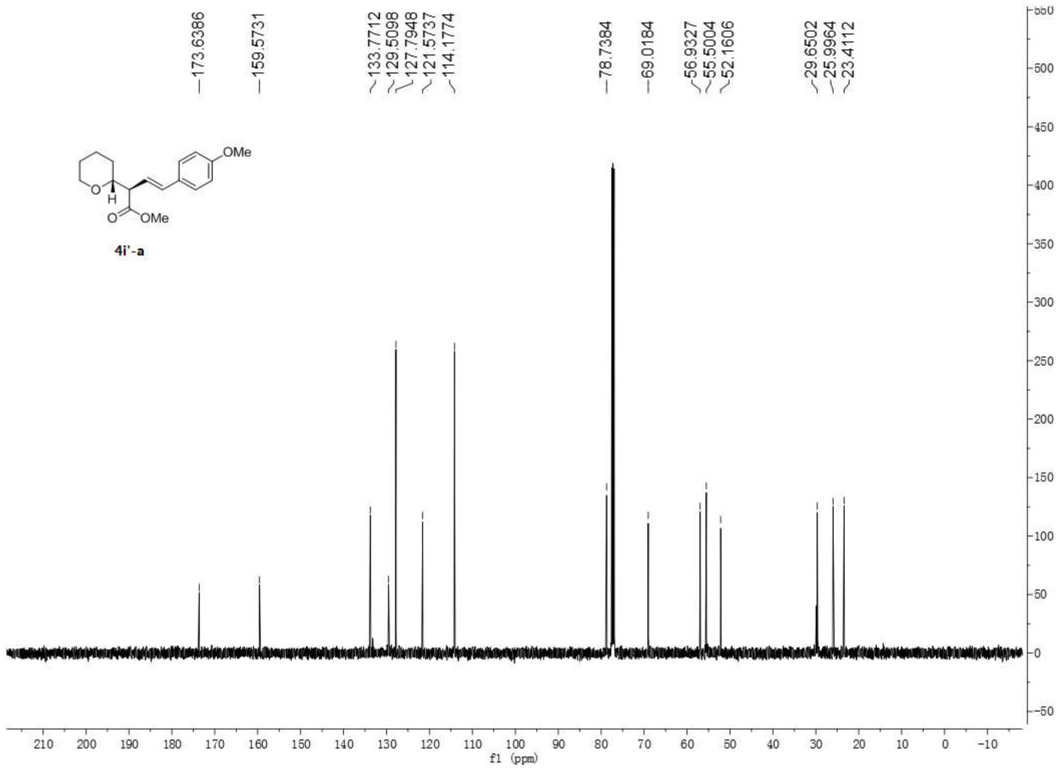

Supplementary figure 119. ^{1}H and ^{13}C NMR spectrum of compound **4i-a'**

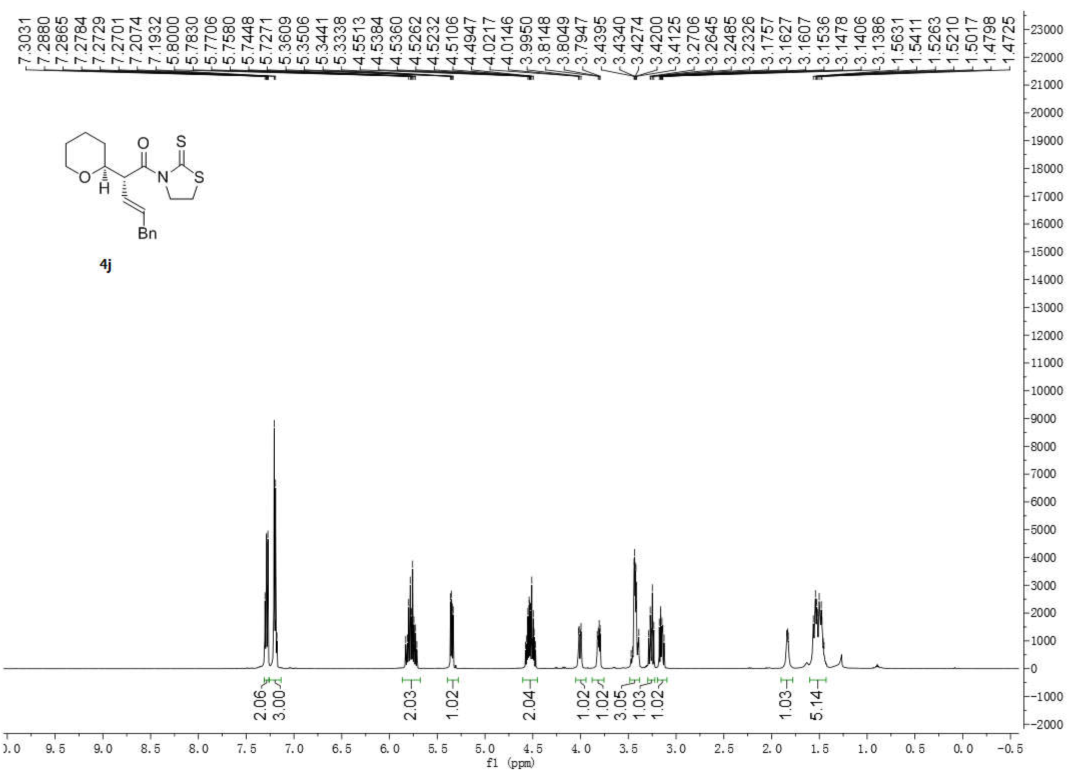

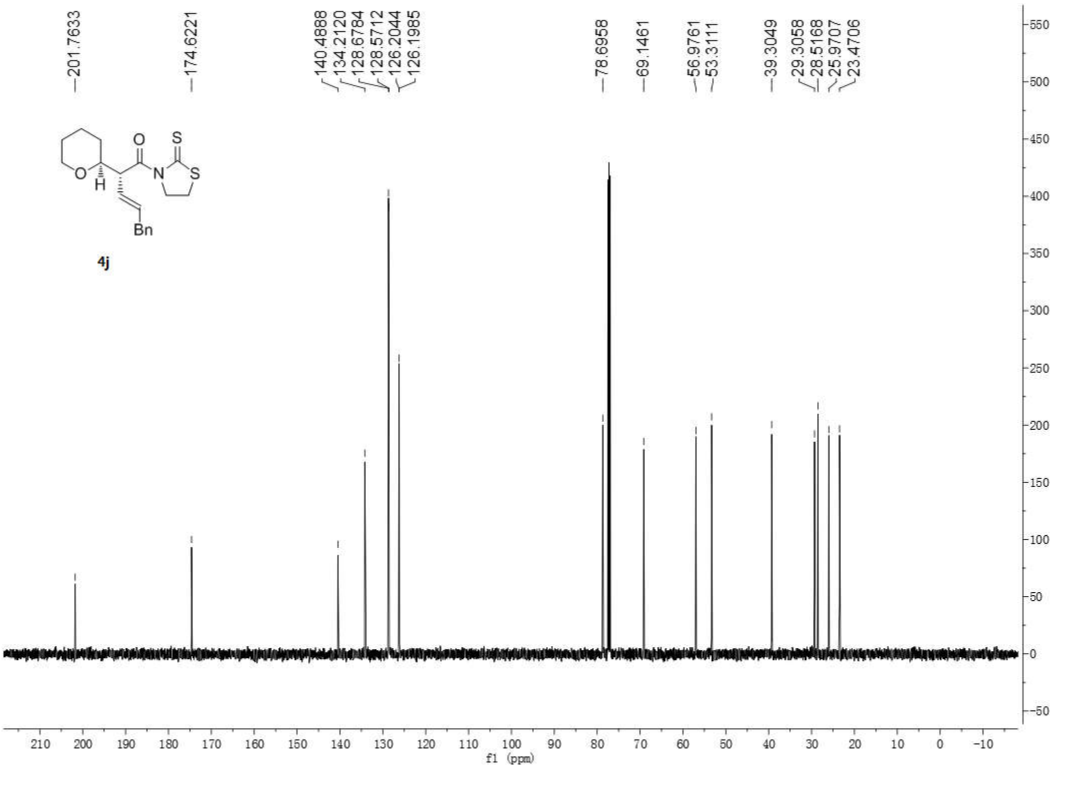

Supplementary figure 120. ^{1}H and ^{13}C NMR spectrum of compound **4j**

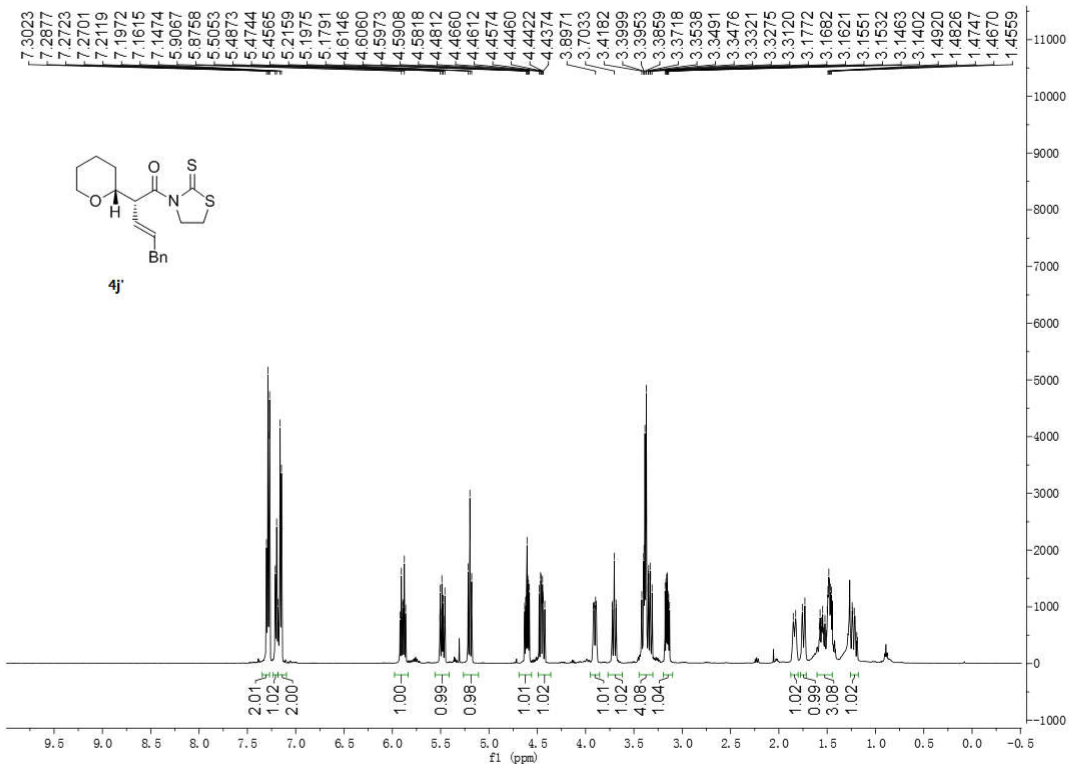

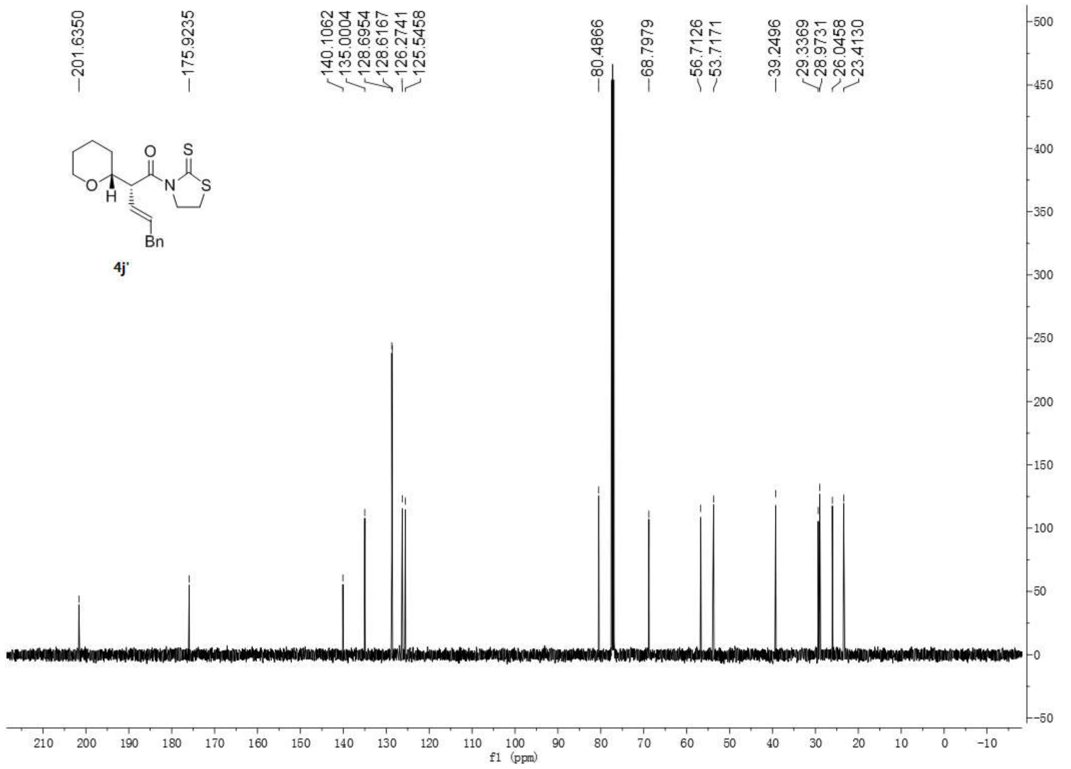

Supplementary figure 121. ^{1}H and ^{13}C NMR spectrum of compound **4j'**

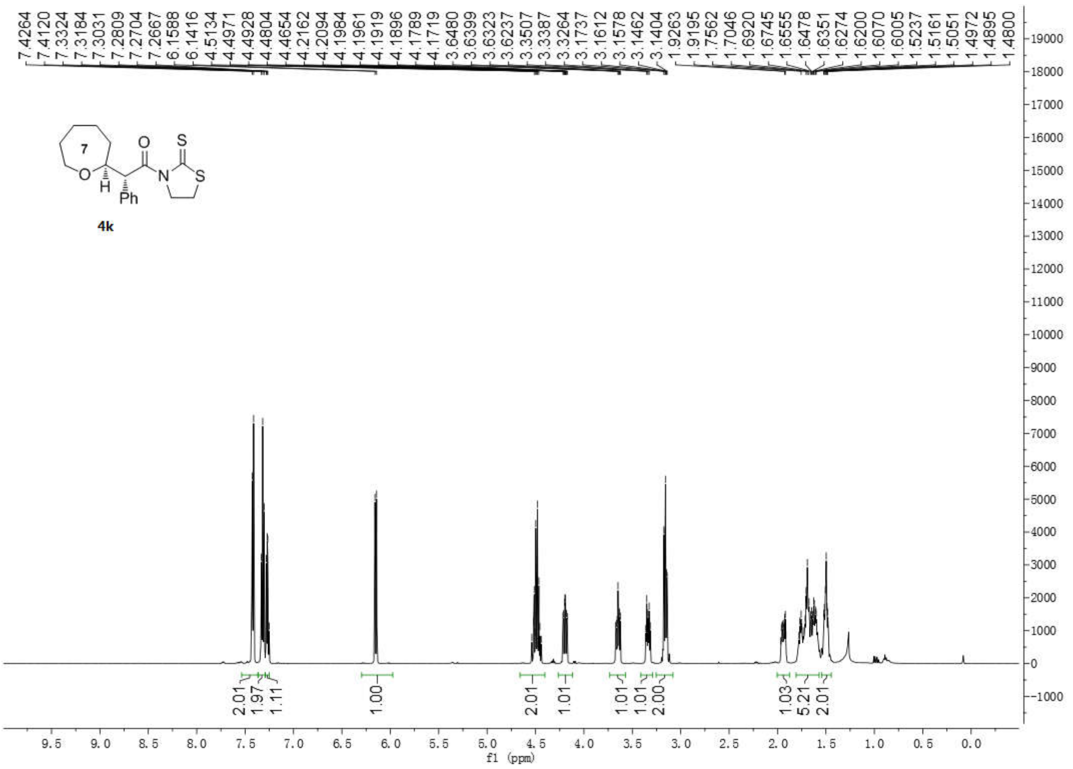

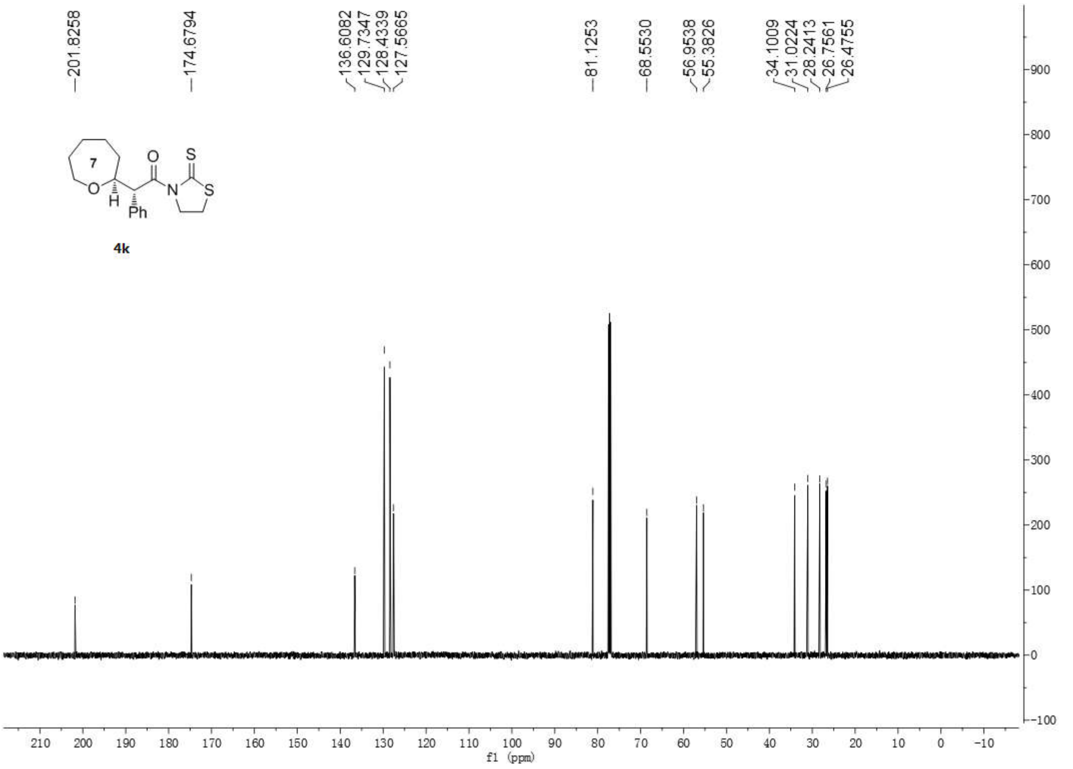

Supplementary figure 122. ^{1}H and ^{13}C NMR spectrum of compound **4k**

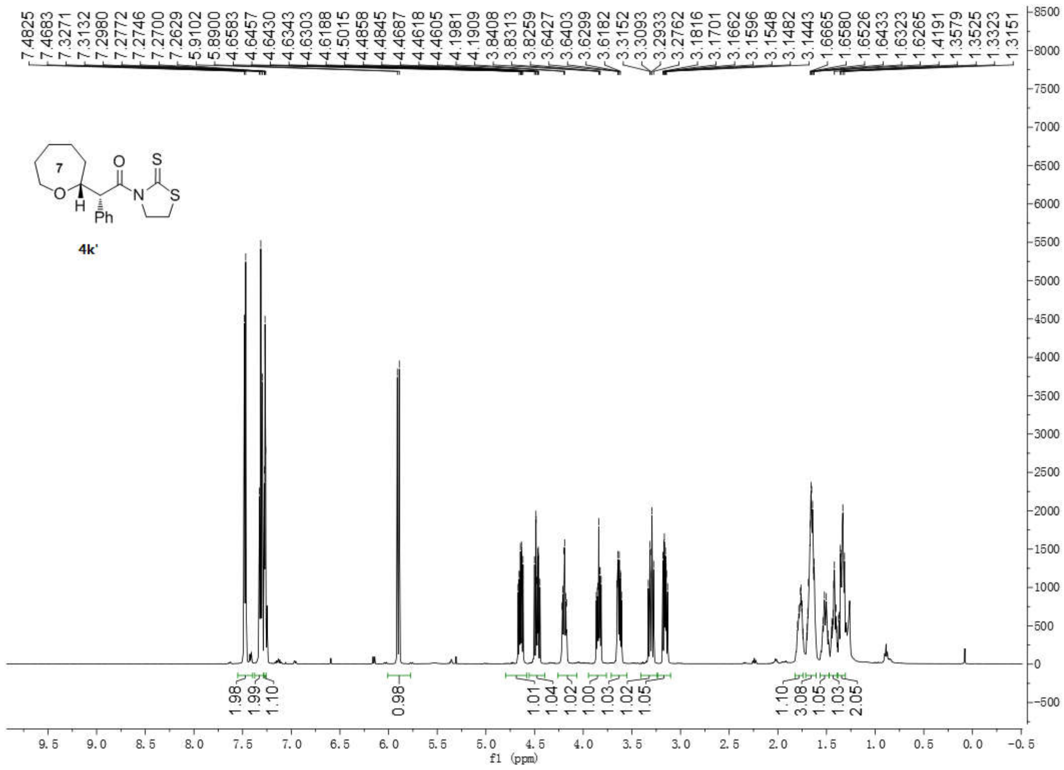

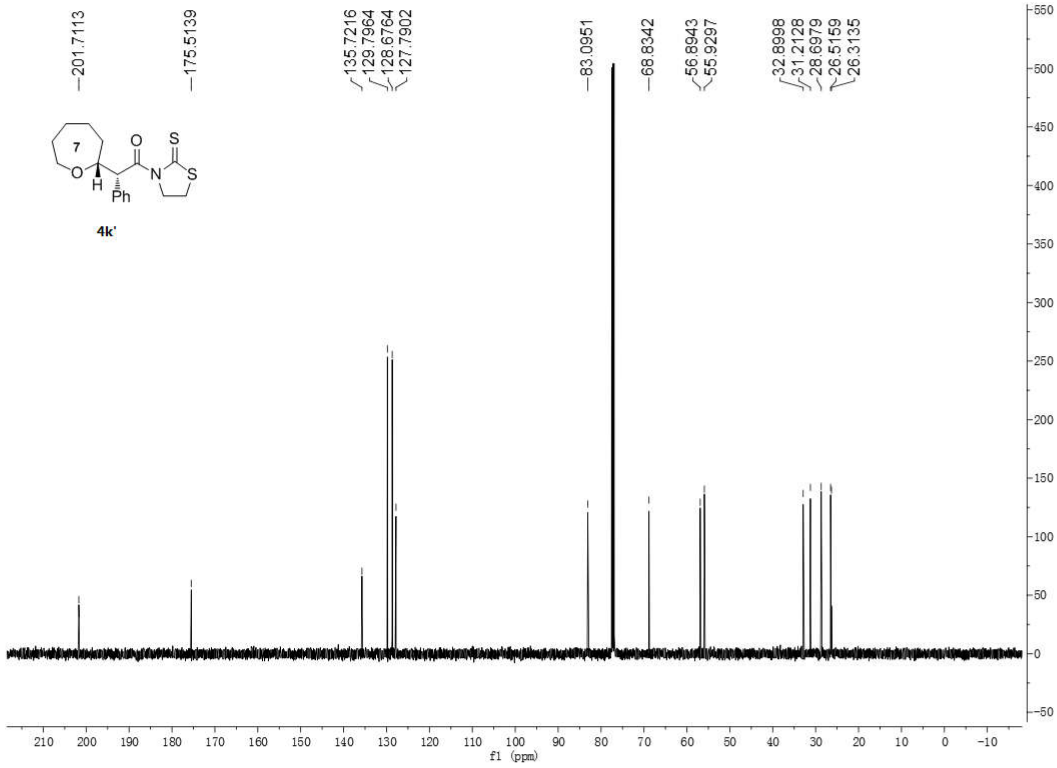

Supplementary figure 123. ^{1}H and ^{13}C NMR spectrum of compound **4k’**

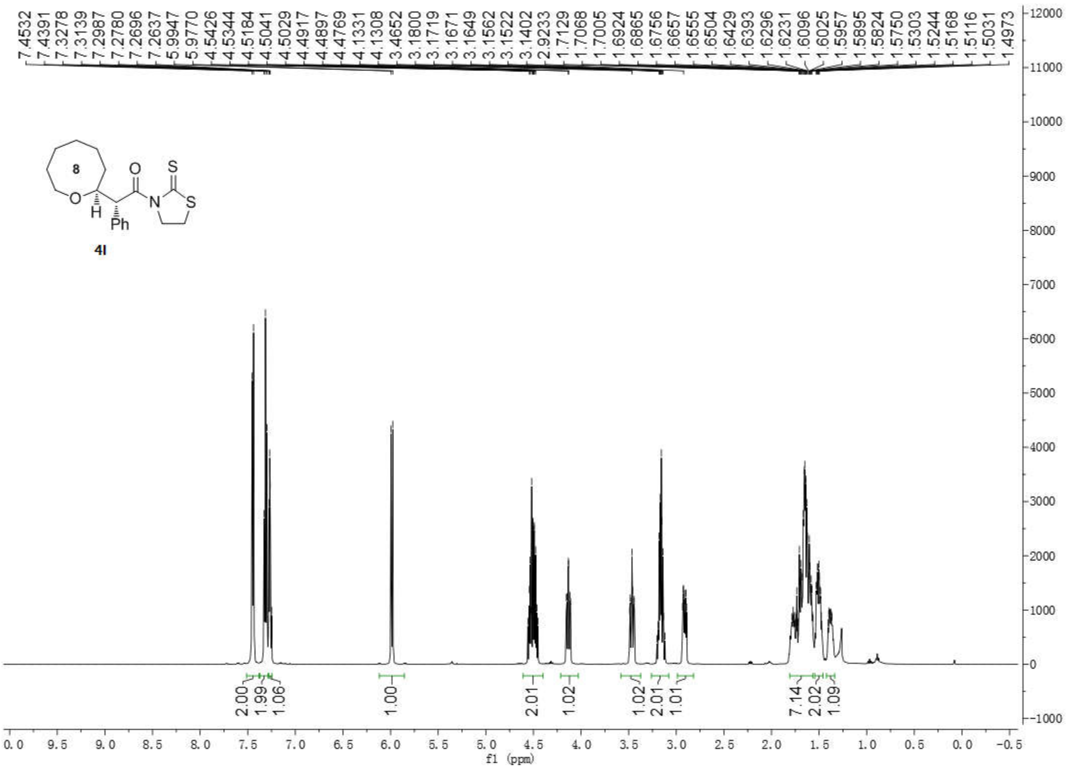

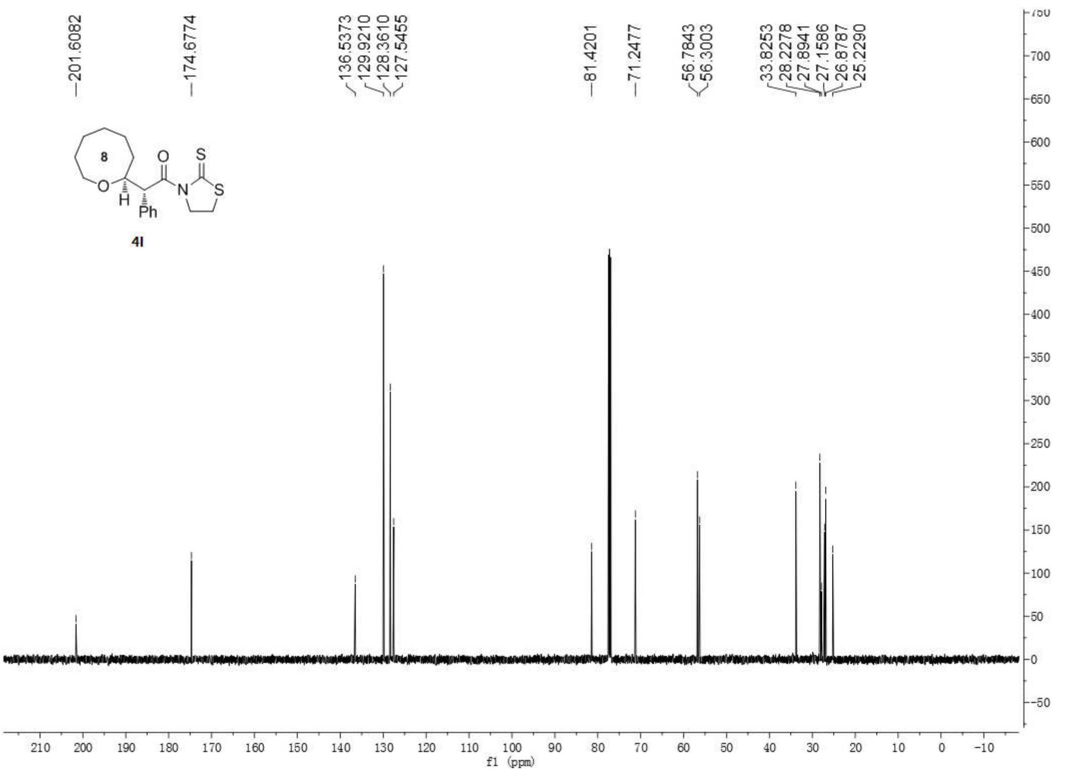

Supplementary figure 124. ^{1}H and ^{13}C NMR spectrum of compound **4l**

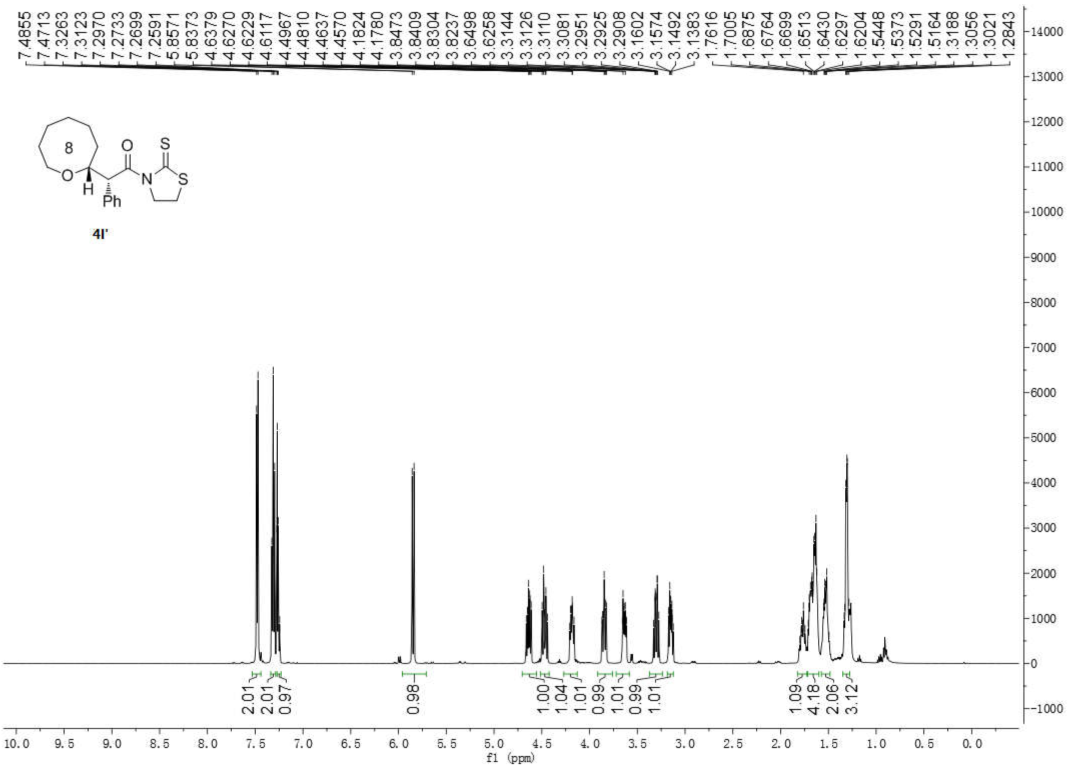

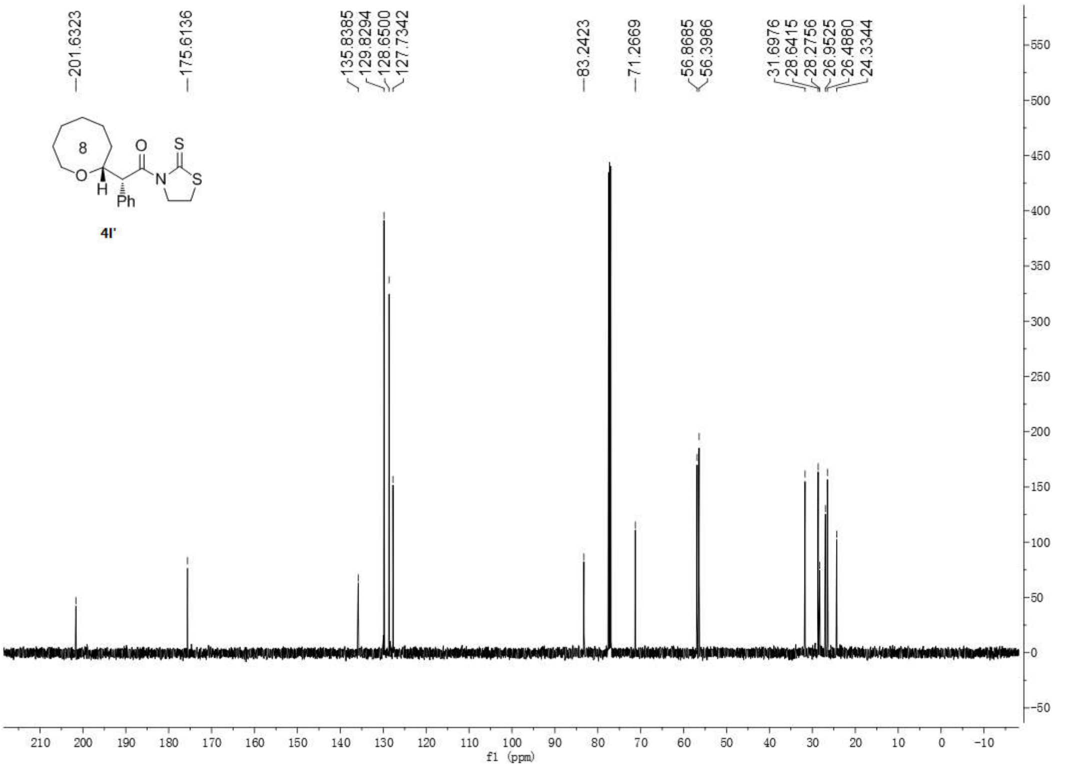

Supplementary figure 125. ^{1}H and ^{13}C NMR spectrum of compound **4l’**

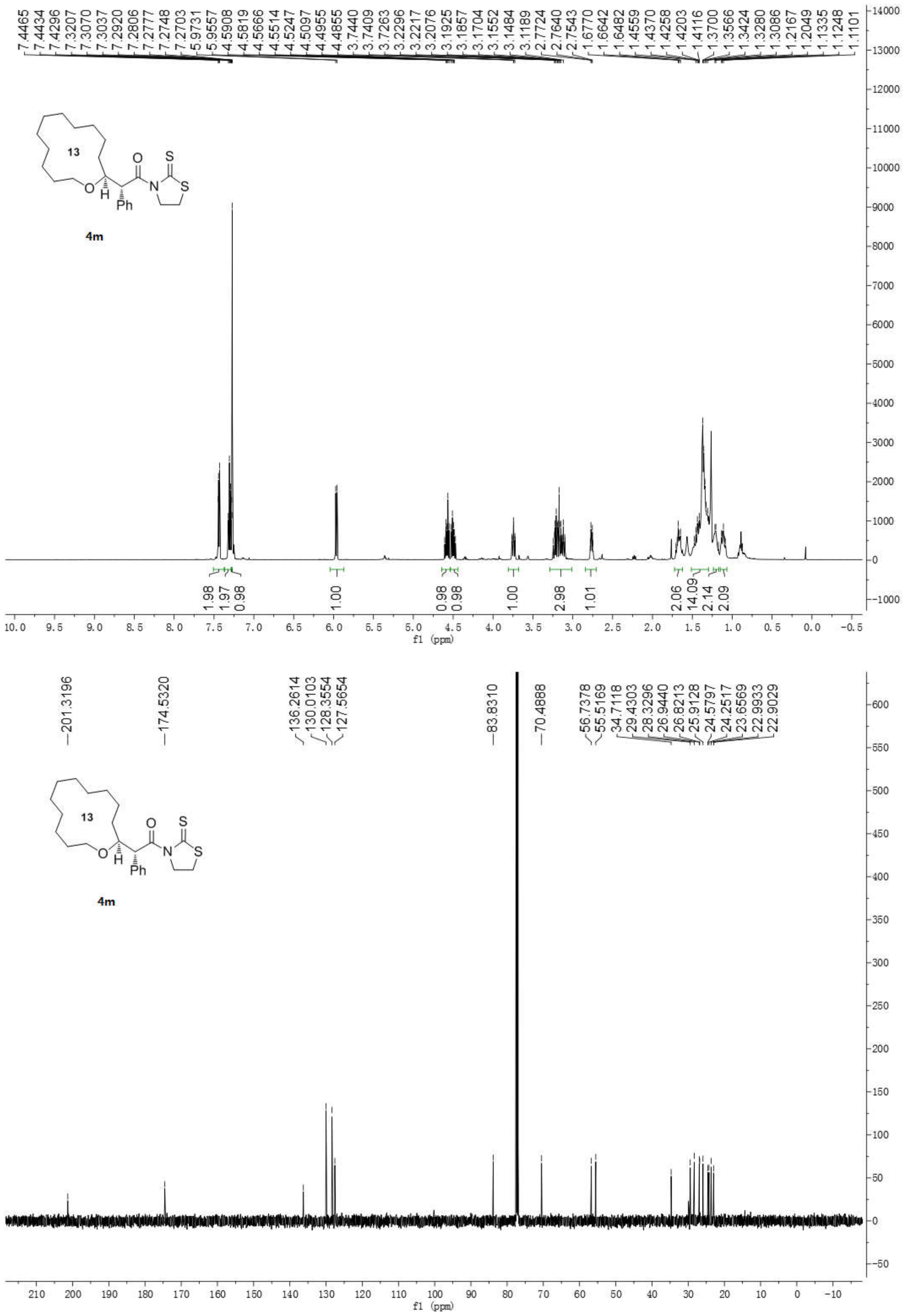

Supplementary figure 126. ^{1}H and ^{13}C NMR spectrum of compound **4m**

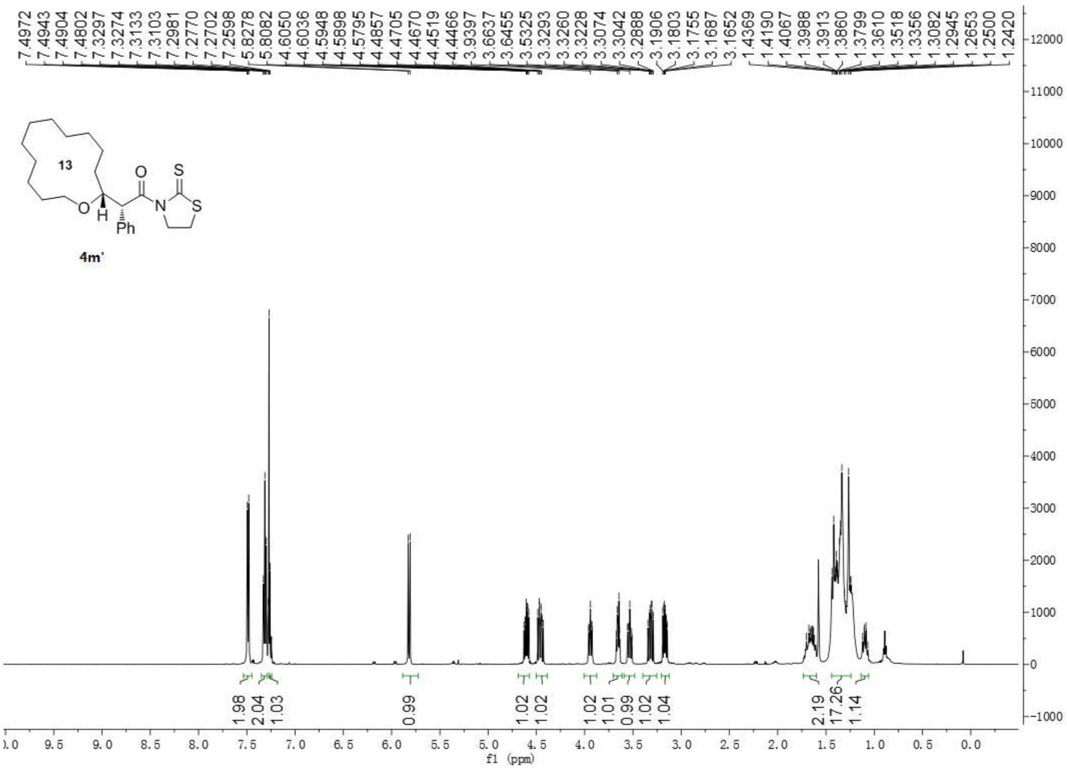

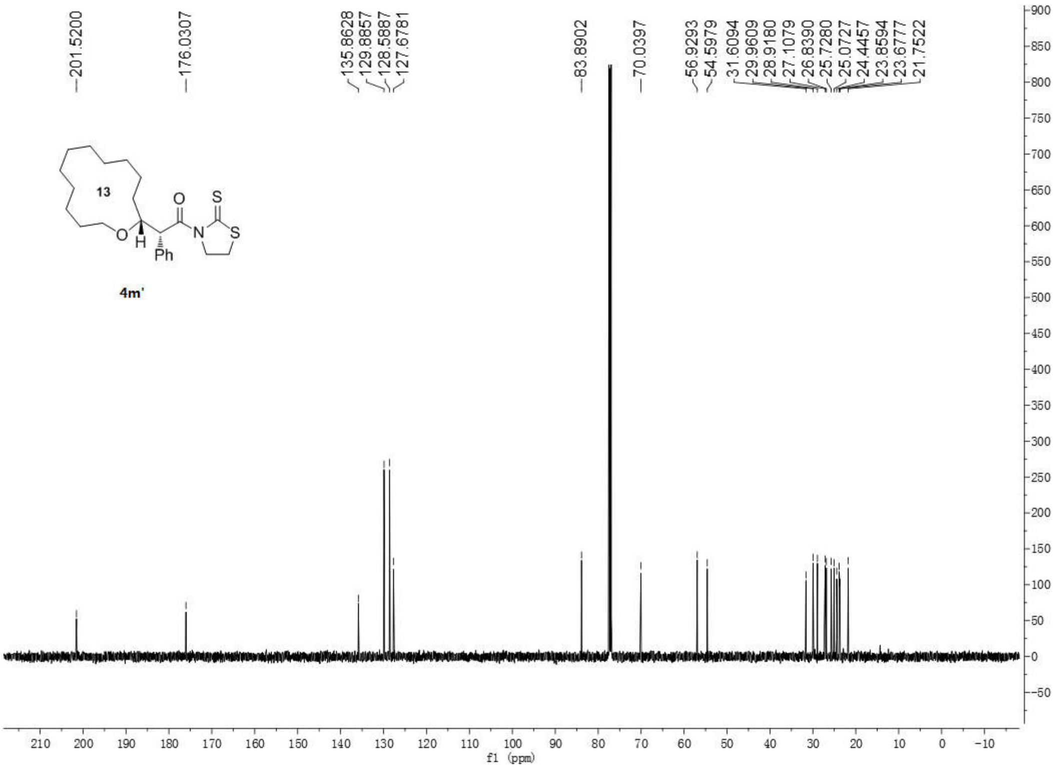

Supplementary figure 127. ^{1}H and ^{13}C NMR spectrum of compound **4m'**

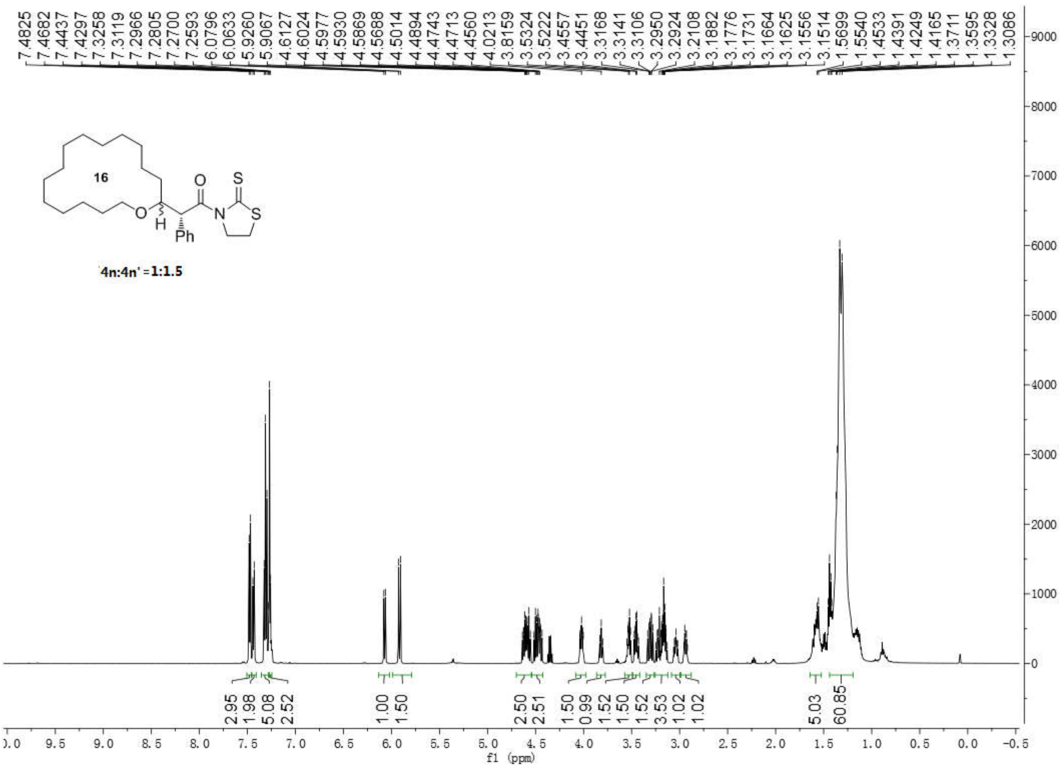

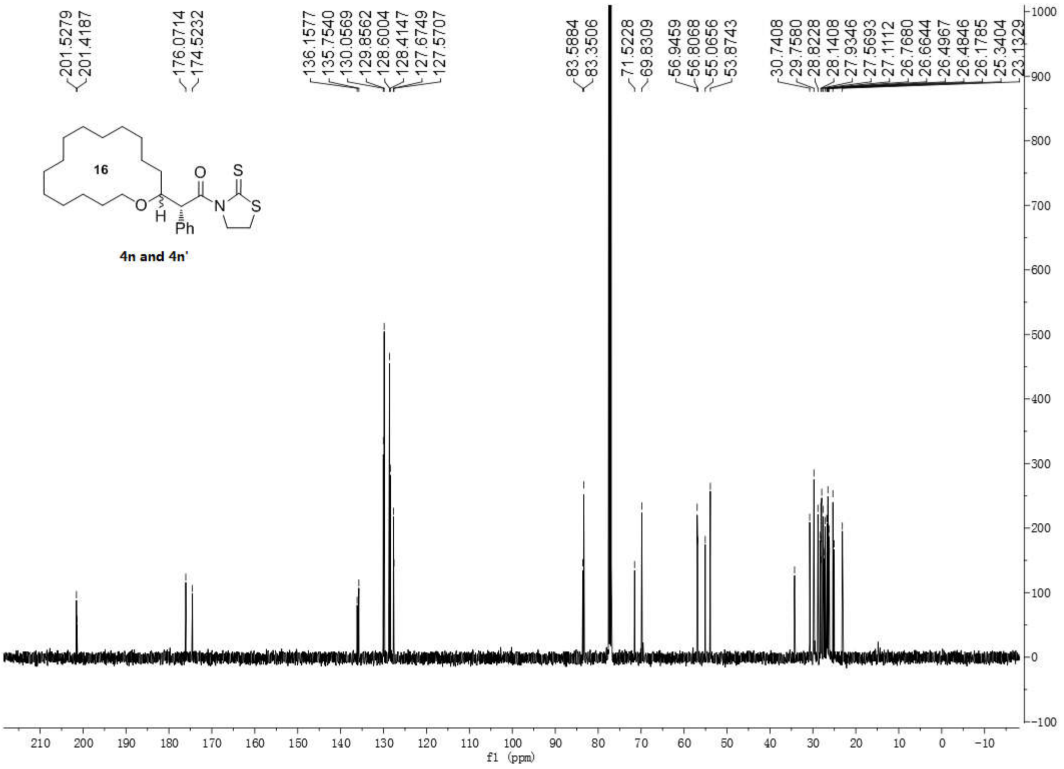

Supplementary figure 128. ^{1}H and ^{13}C NMR spectrum of compound **4n** and **4n'**

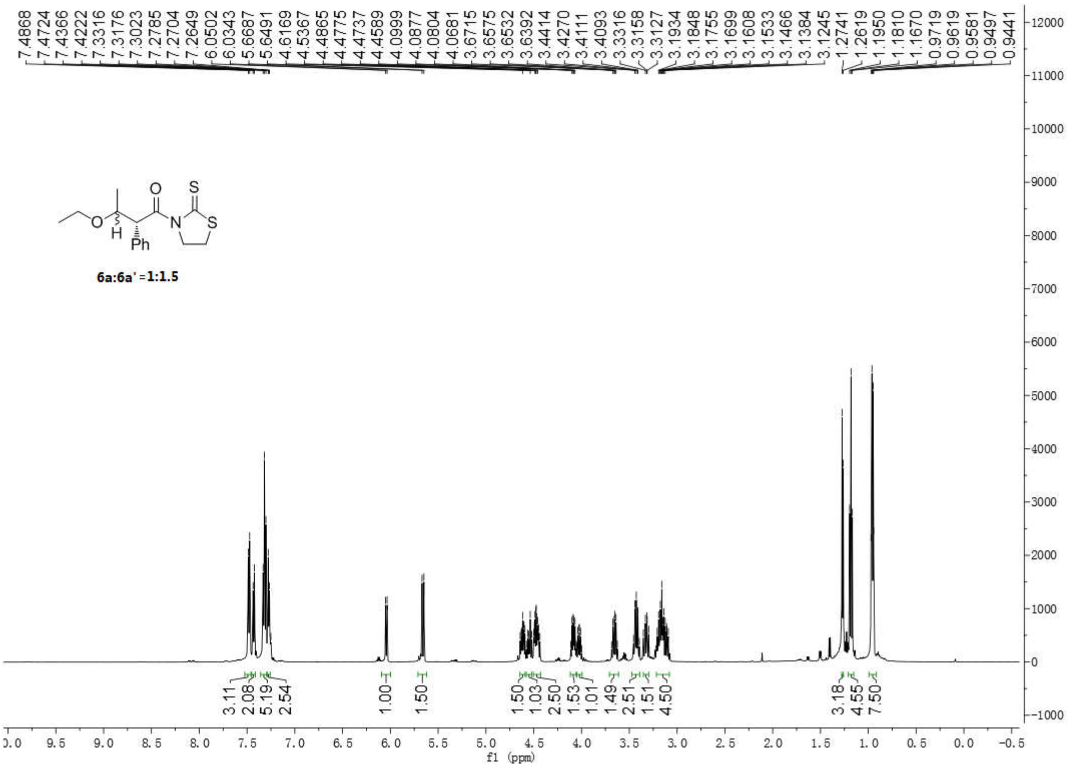

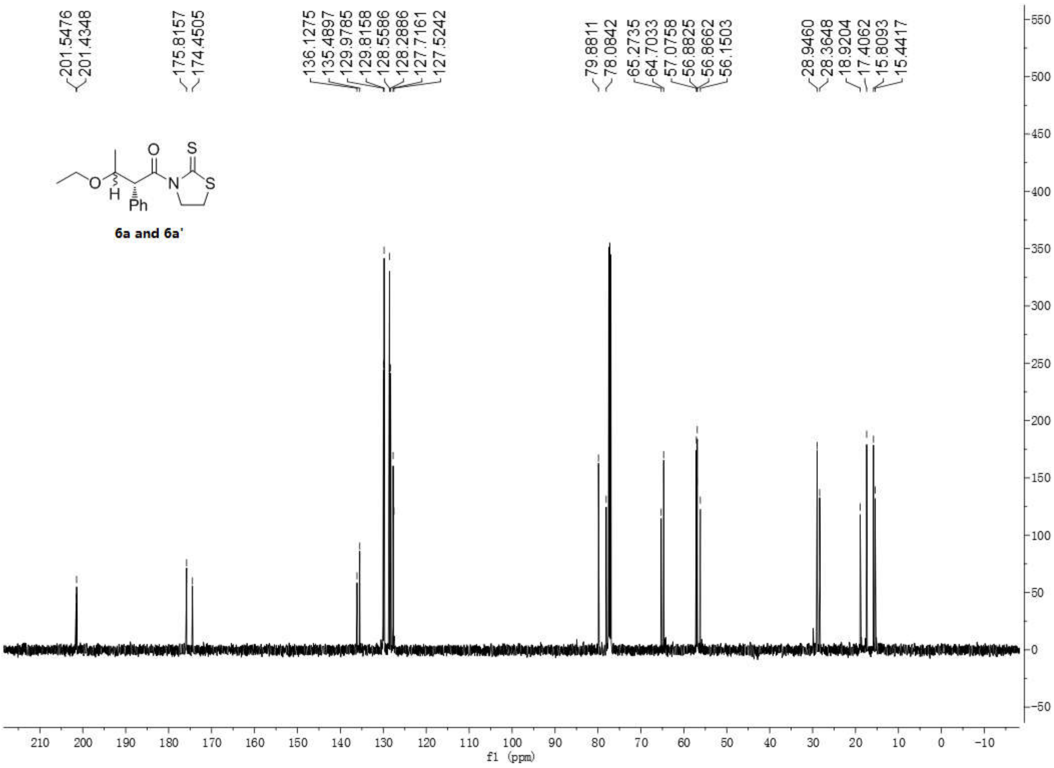

Supplementary figure 129. ^{1}H and ^{13}C NMR spectrum of compound **6a** and **6a’**

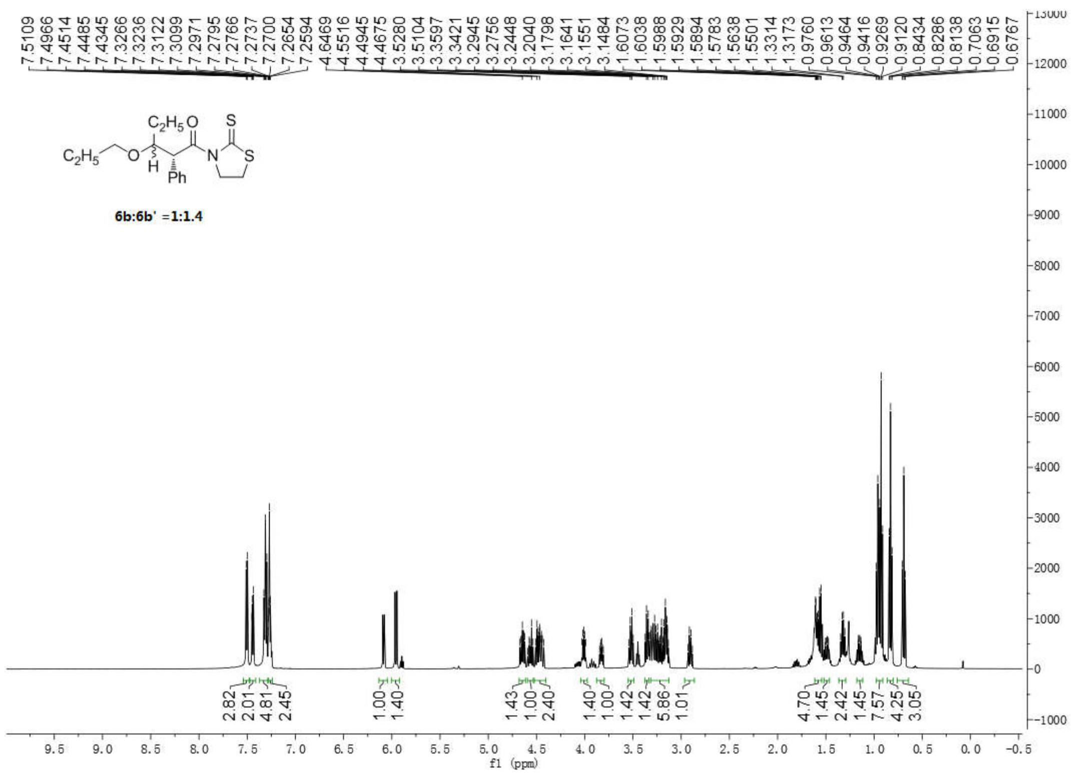

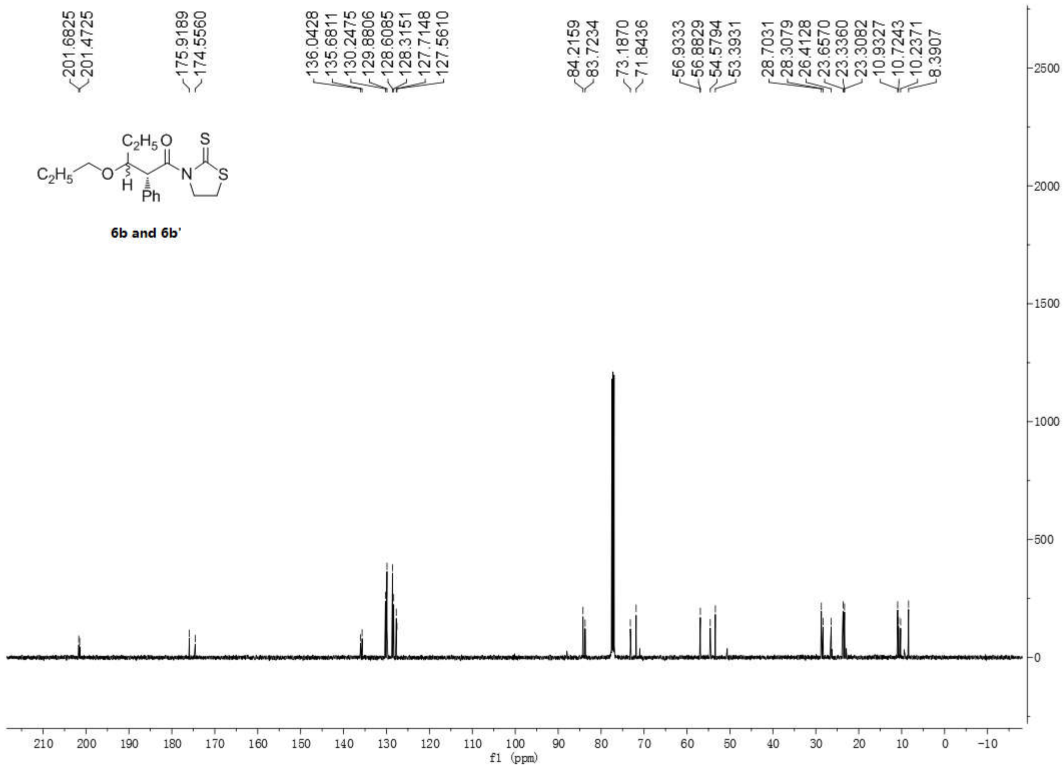

Supplementary figure 130. ^{1}H and ^{13}C NMR spectrum of compound **6b** and **6b'**

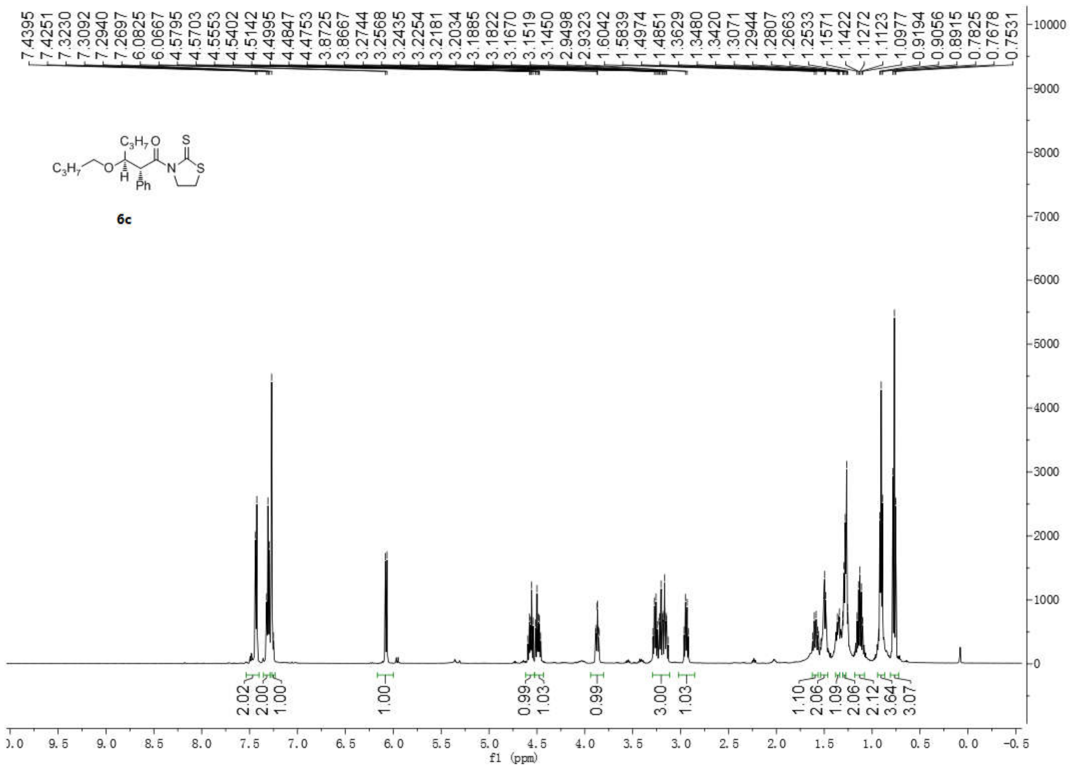

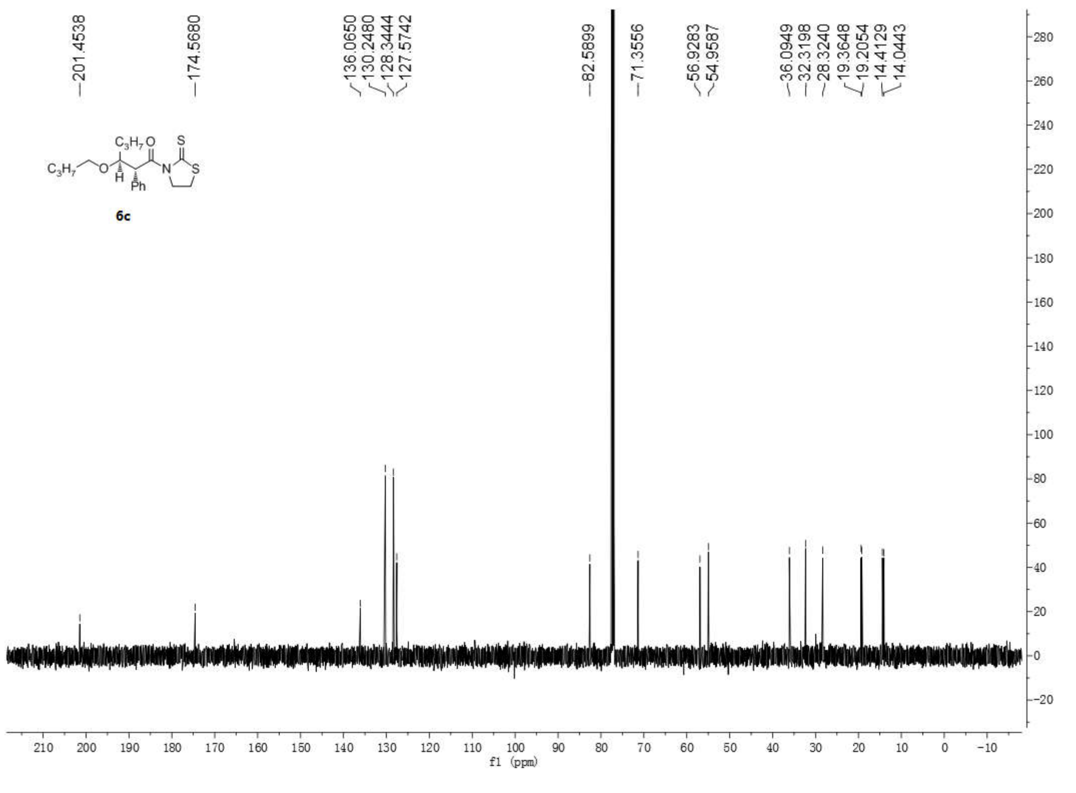

Supplementary figure 131. ^{1}H and ^{13}C NMR spectrum of compound **6c**

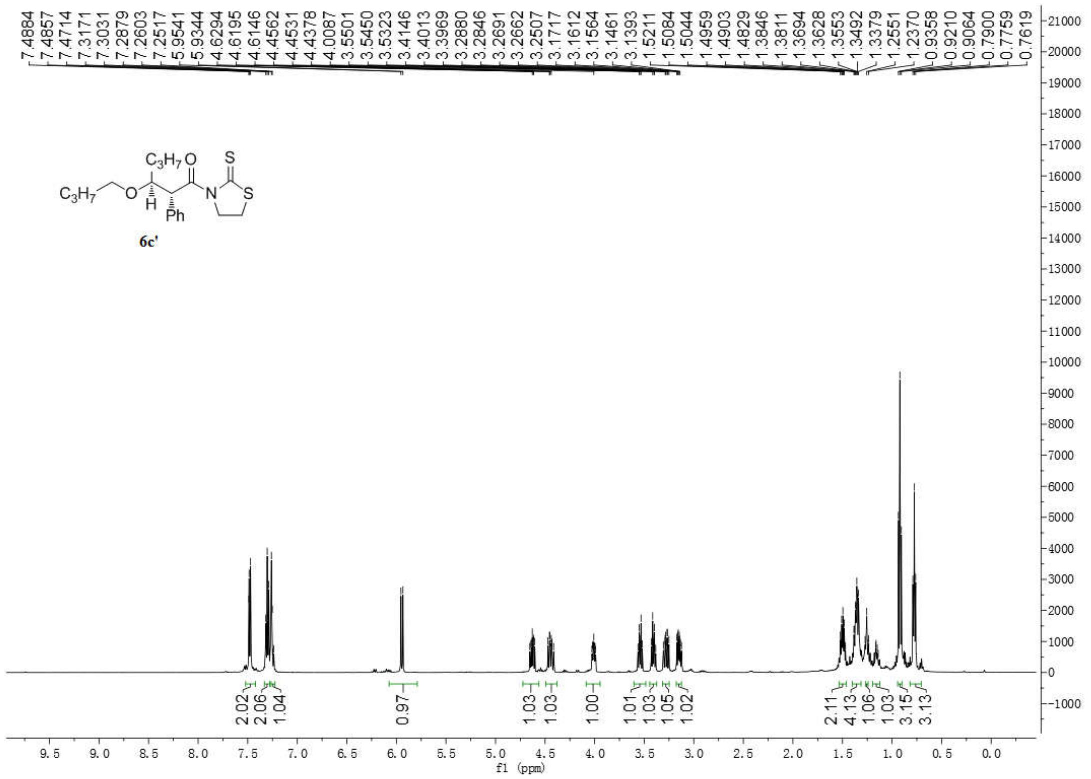

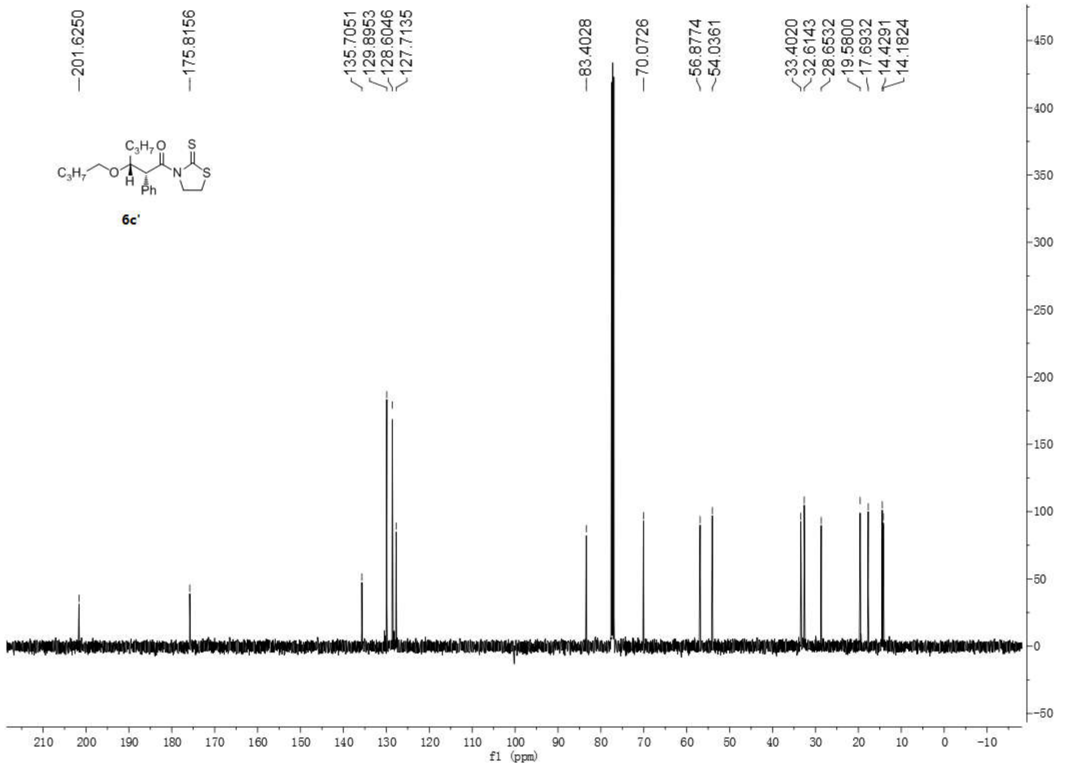

Supplementary figure 132. ^{1}H and ^{13}C NMR spectrum of compound **6c’**

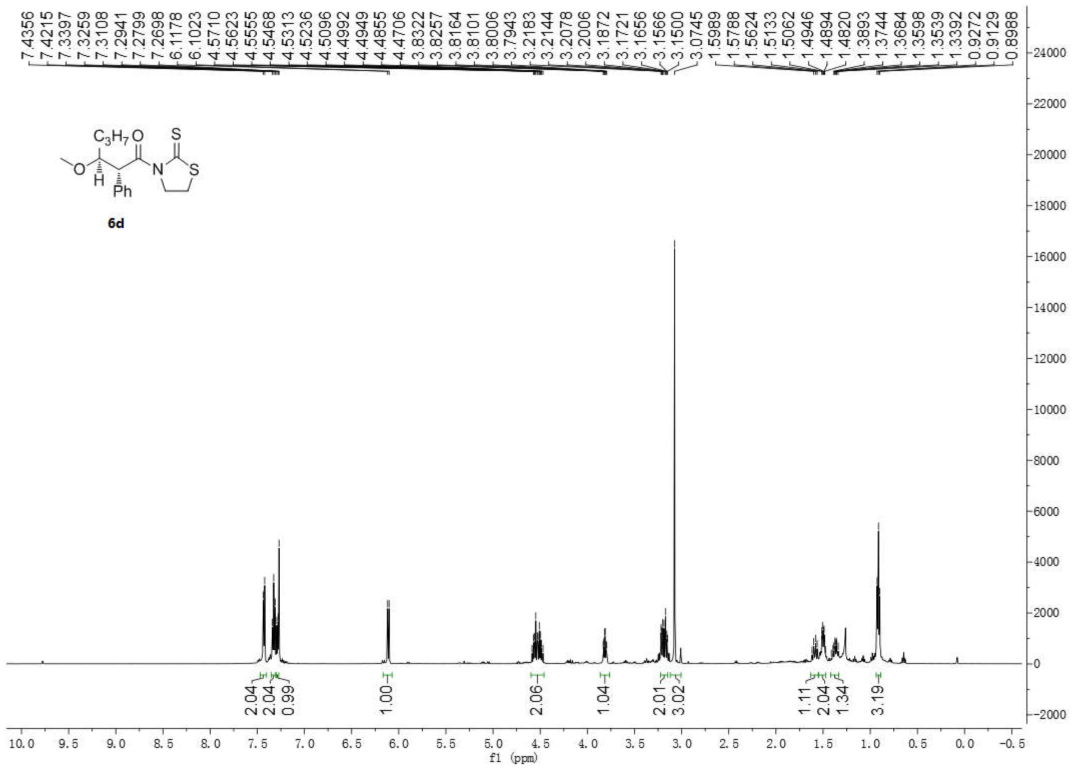

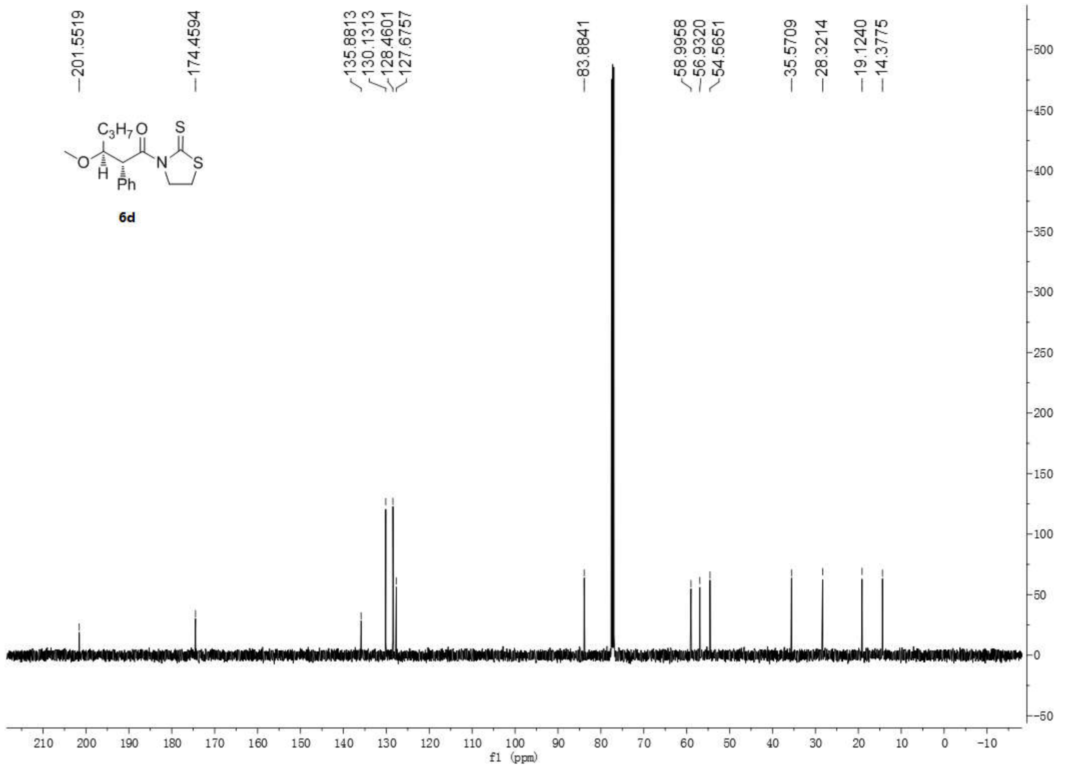

Supplementary figure 133. ^{1}H and ^{13}C NMR spectrum of compound **6d**

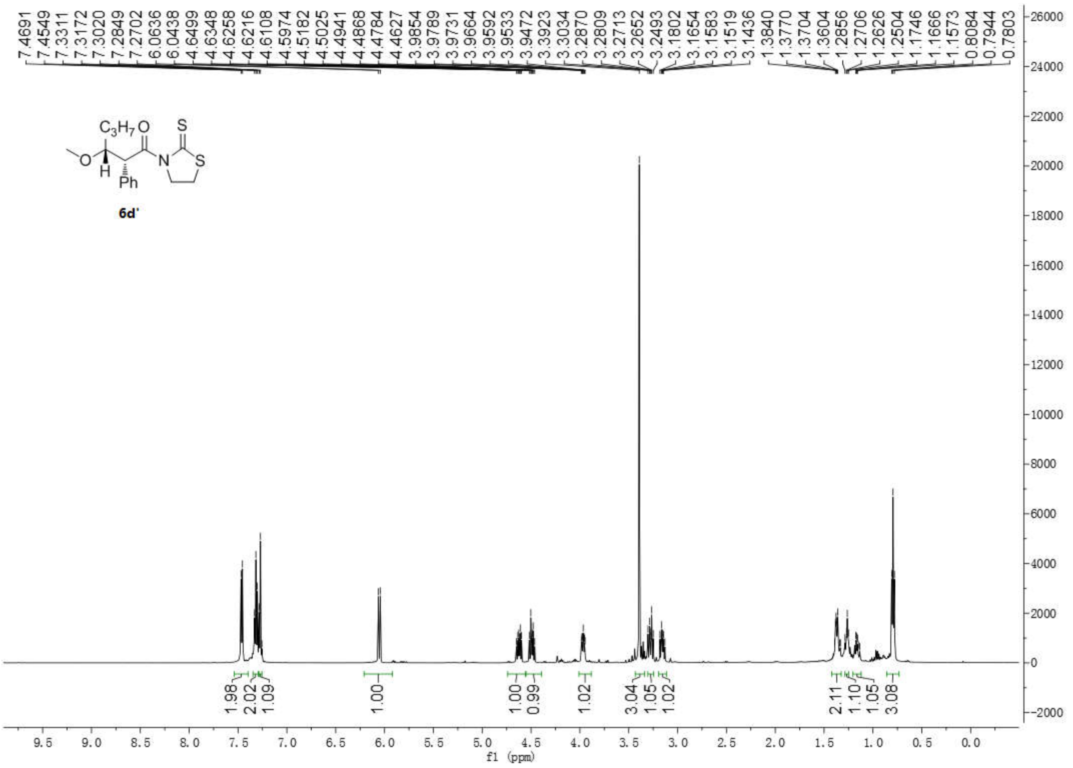

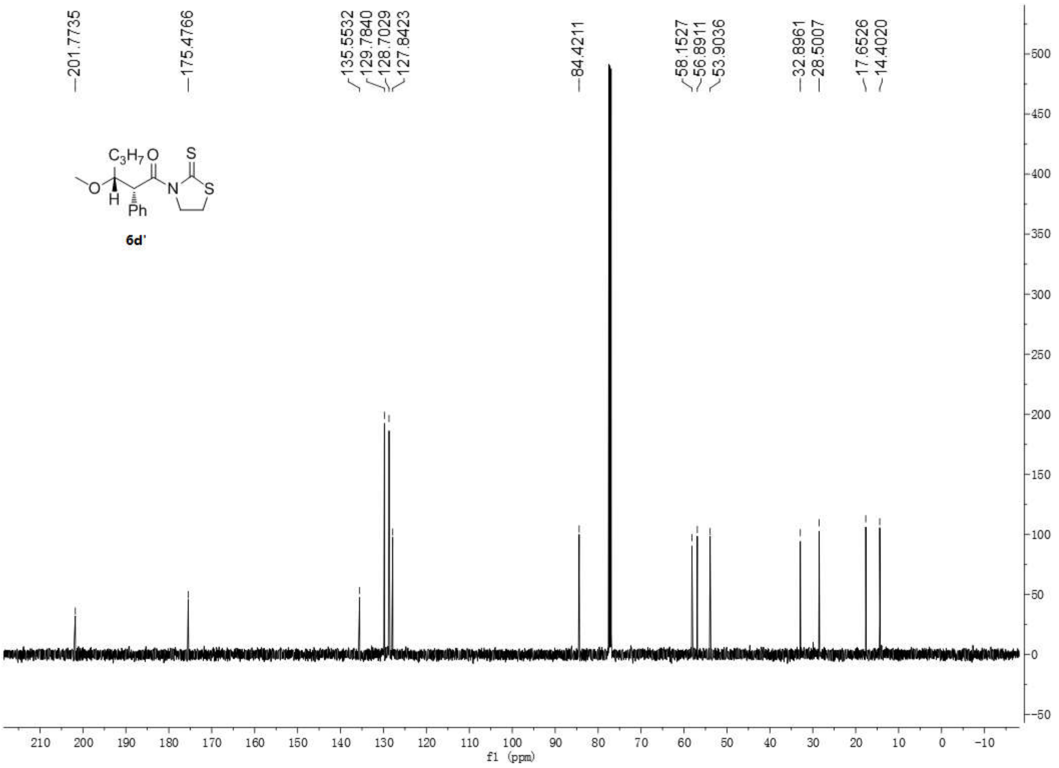

Supplementary figure 134. ^{1}H and ^{13}C NMR spectrum of compound **6d’**

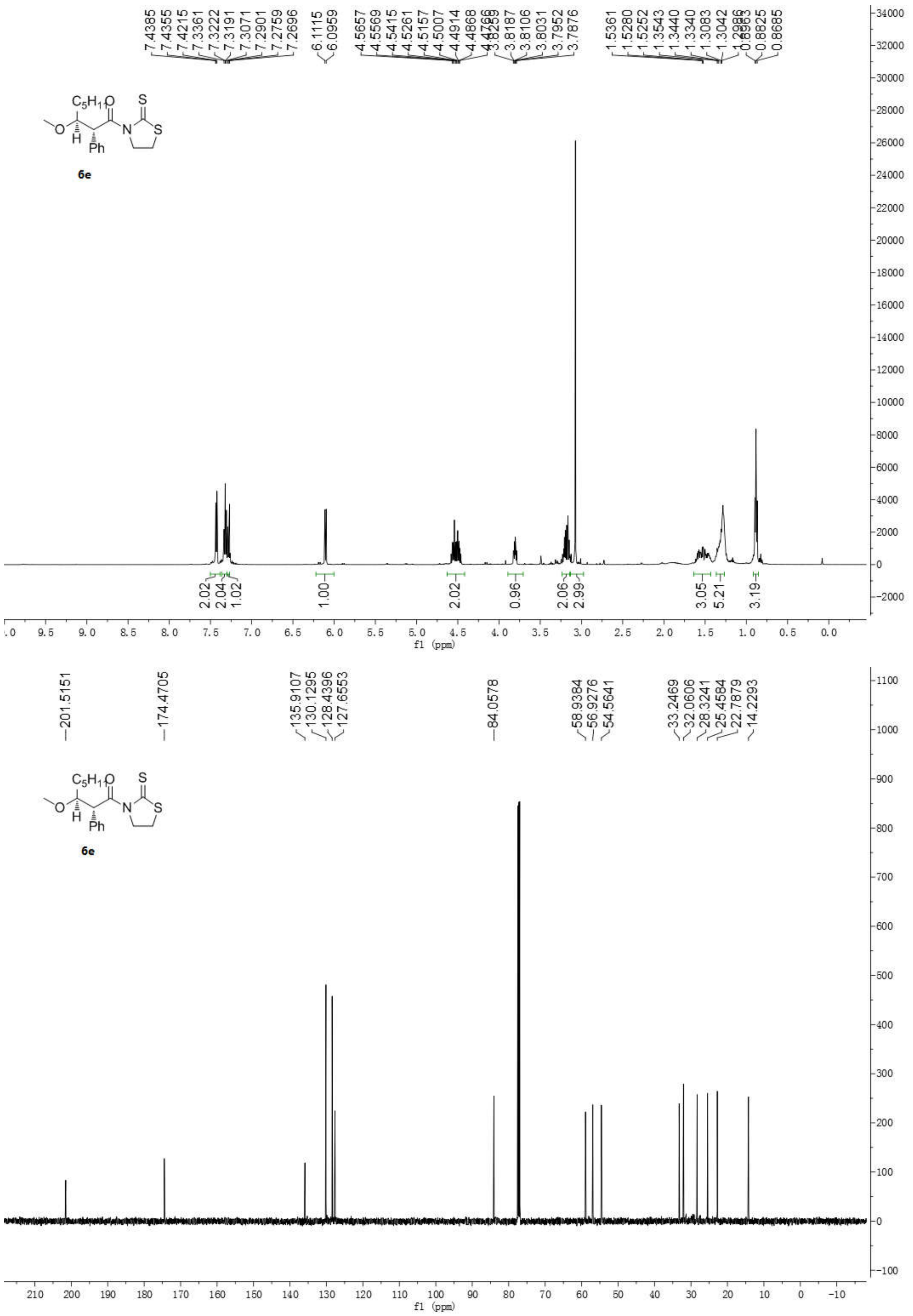

Supplementary figure 135. ^{1}H and ^{13}C NMR spectrum of compound **6e**

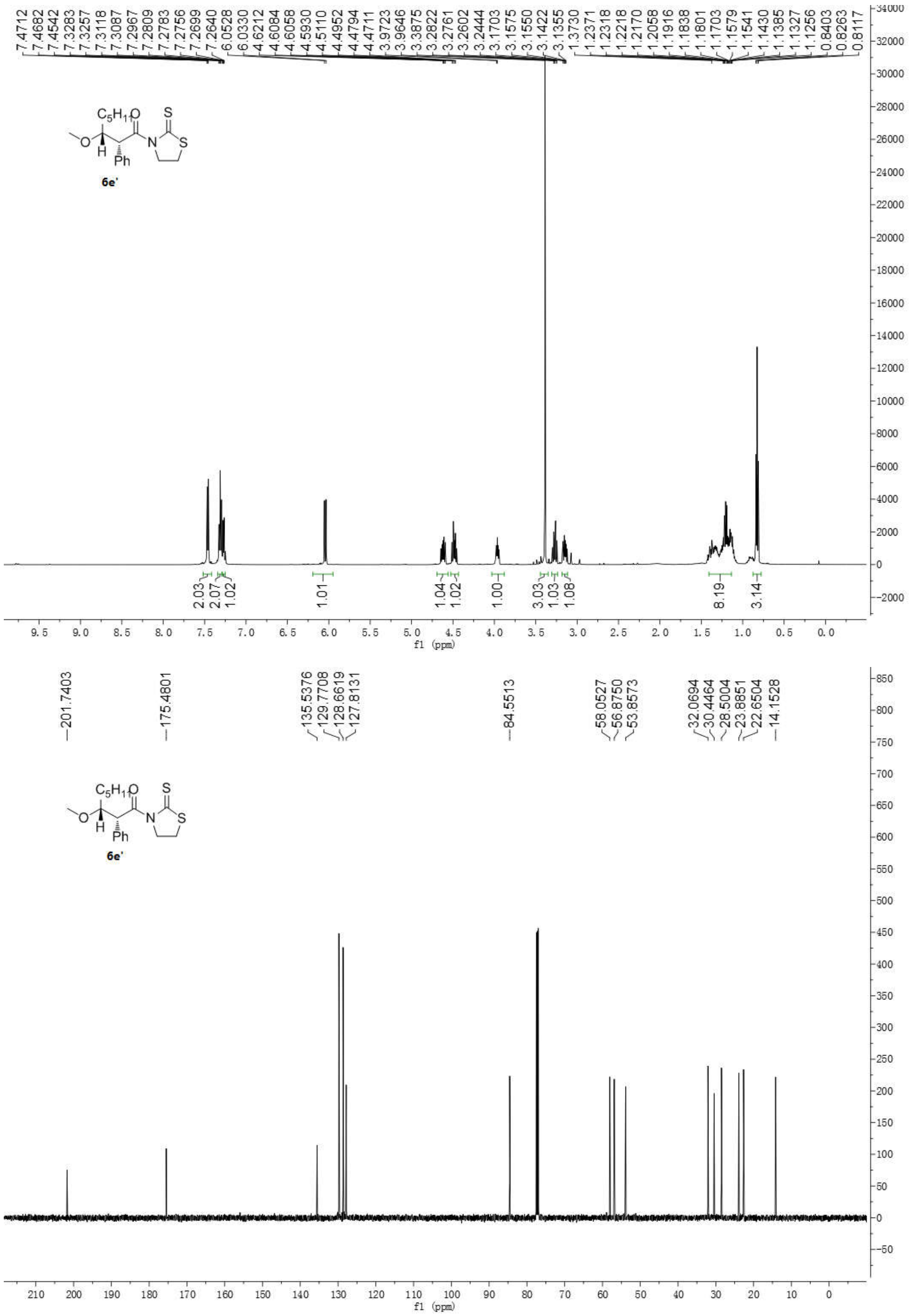

Supplementary figure 136. ^{1}H and ^{13}C NMR spectrum of compound **6e’**

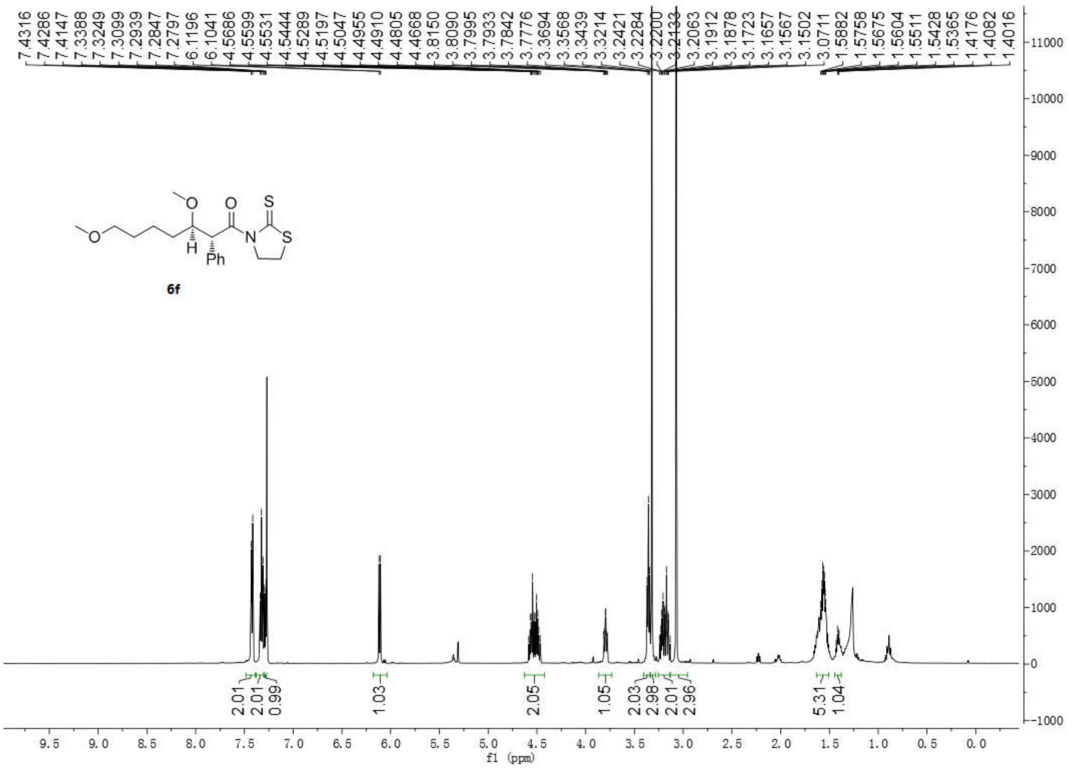

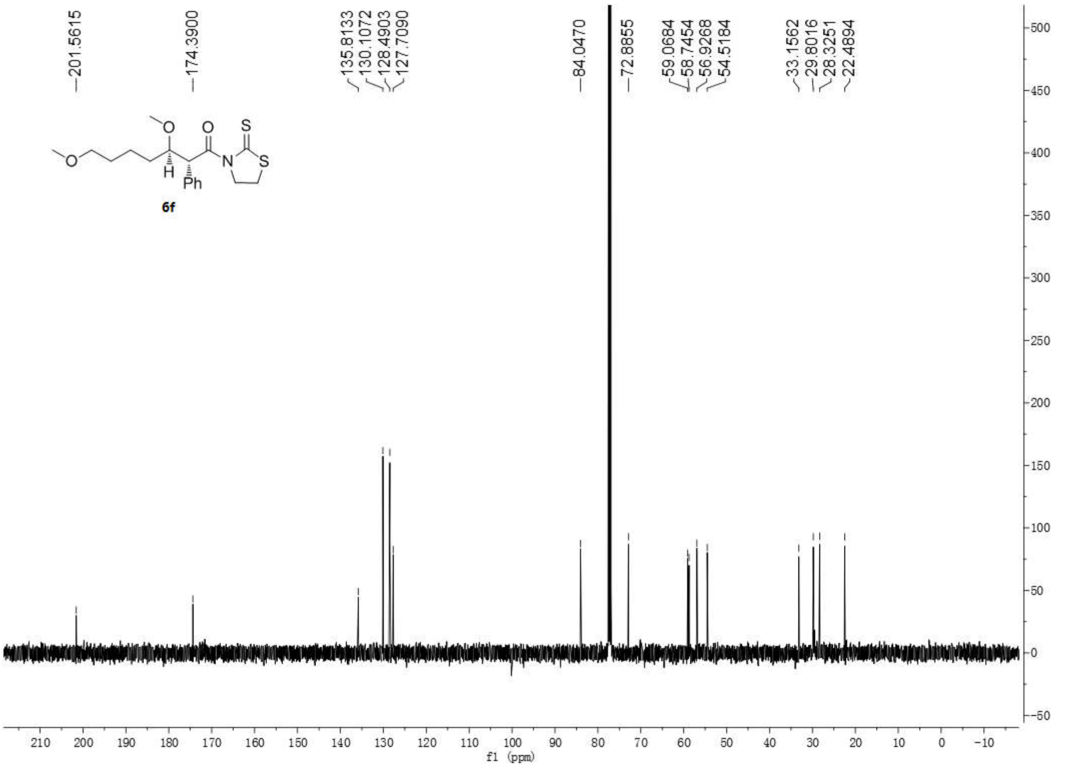

Supplementary figure 137. ^{1}H and ^{13}C NMR spectrum of compound **6f**

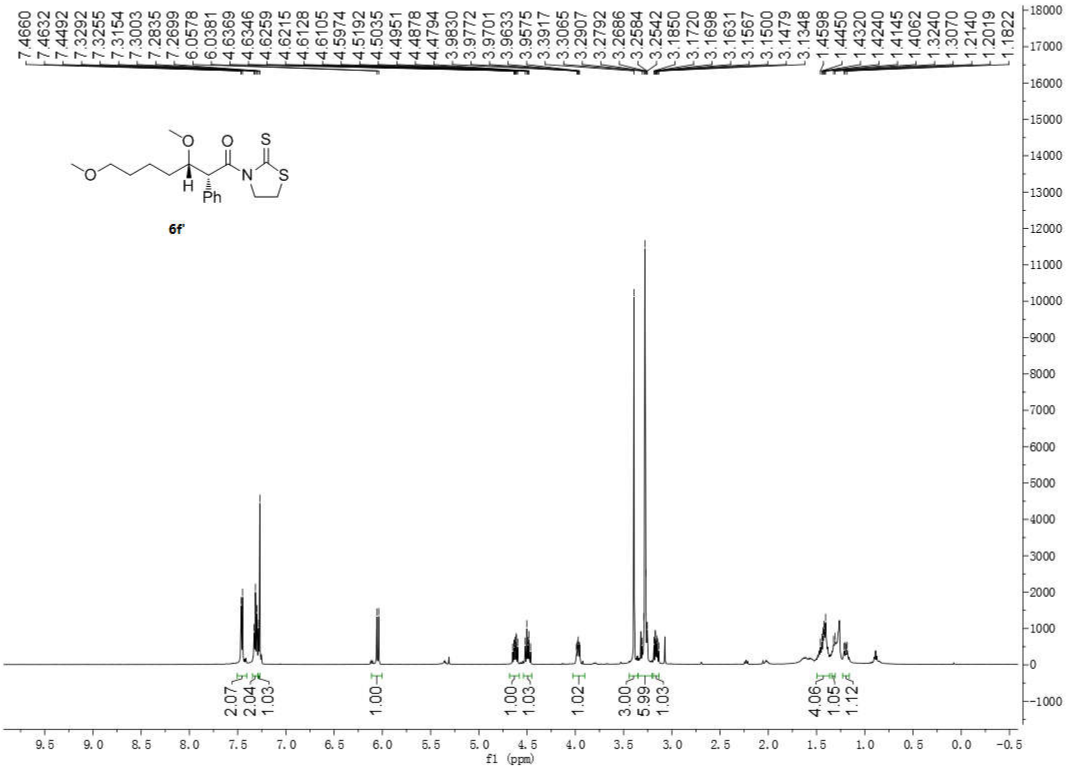

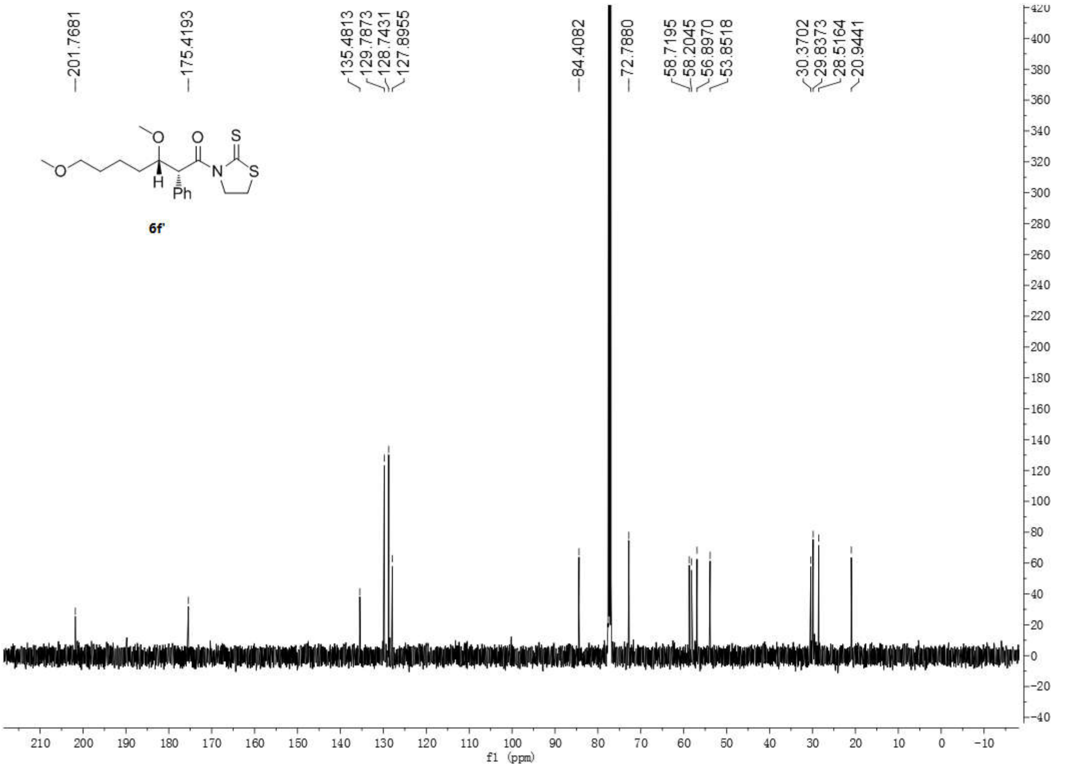

Supplementary figure 138. ^{1}H and ^{13}C NMR spectrum of compound **6f’**

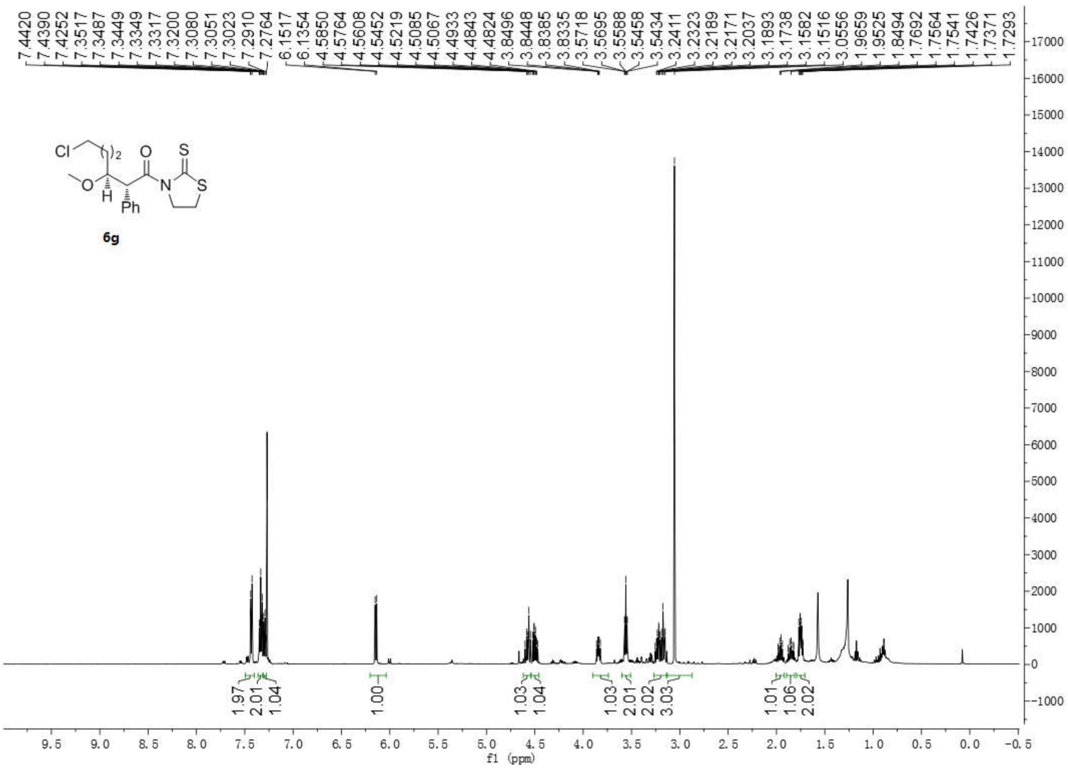

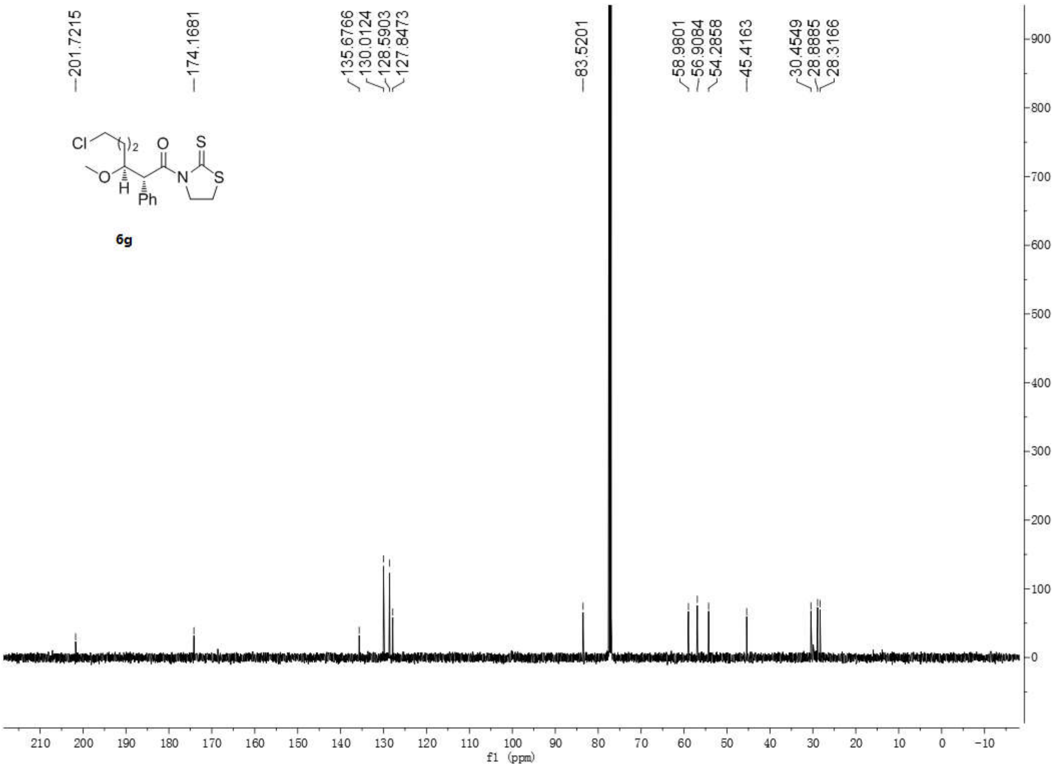

Supplementary figure 139. ^{1}H and ^{13}C NMR spectrum of compound **6g**

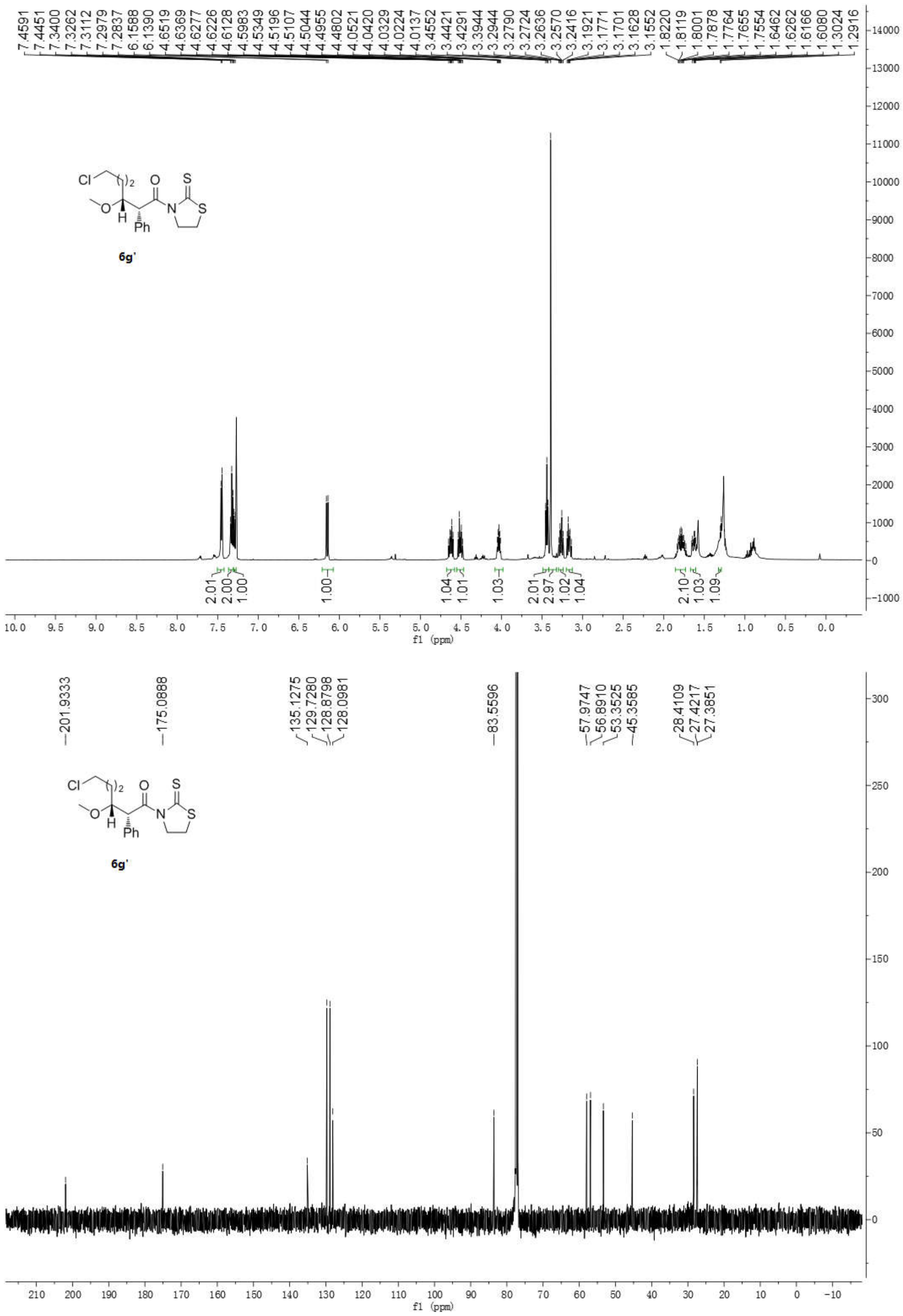

Supplementary figure 140. ^{1}H and ^{13}C NMR spectrum of compound **6g’**

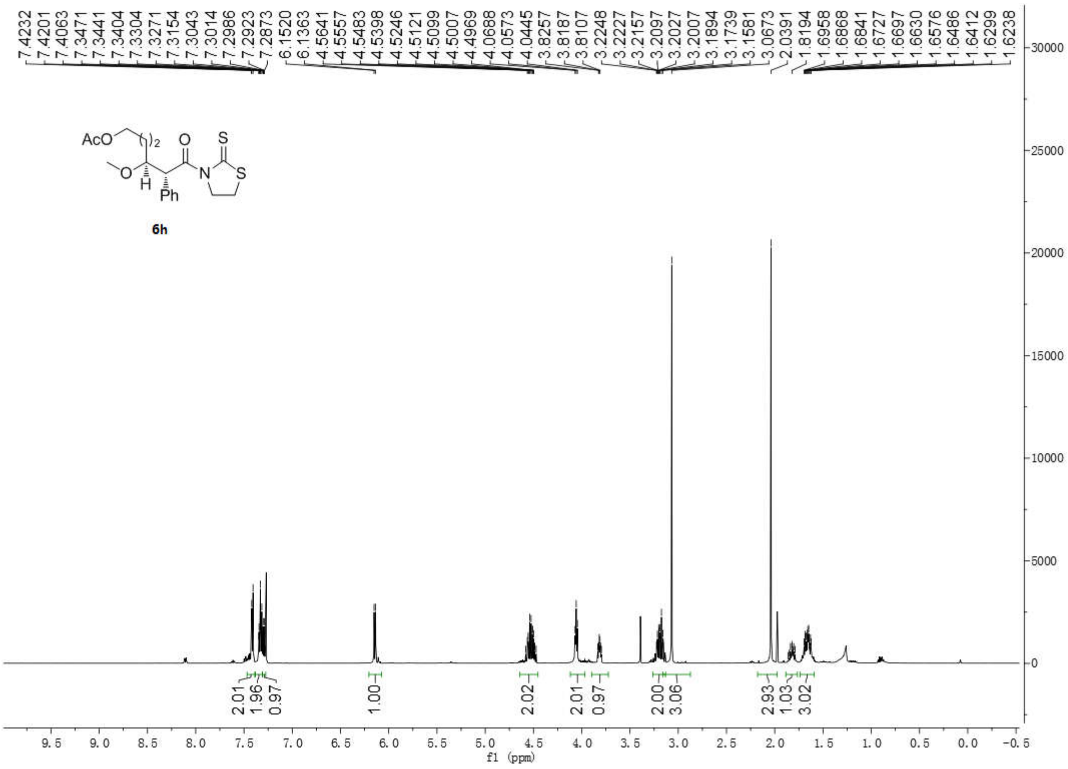

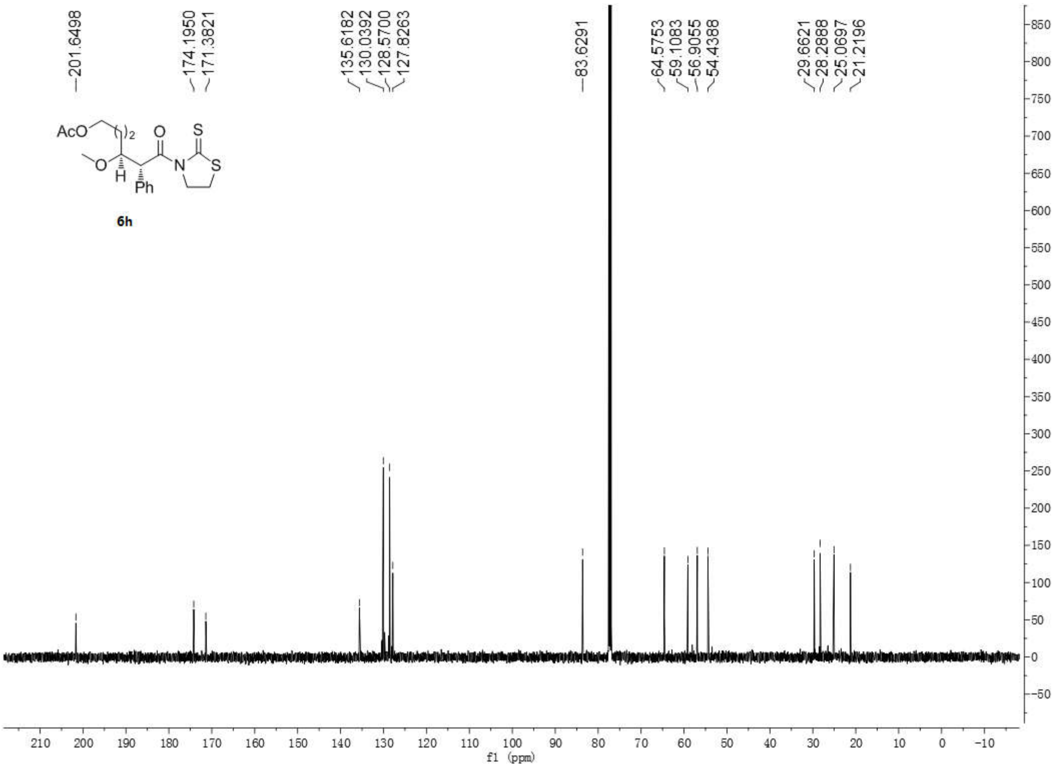

Supplementary figure 141. ^{1}H and ^{13}C NMR spectrum of compound **6h**

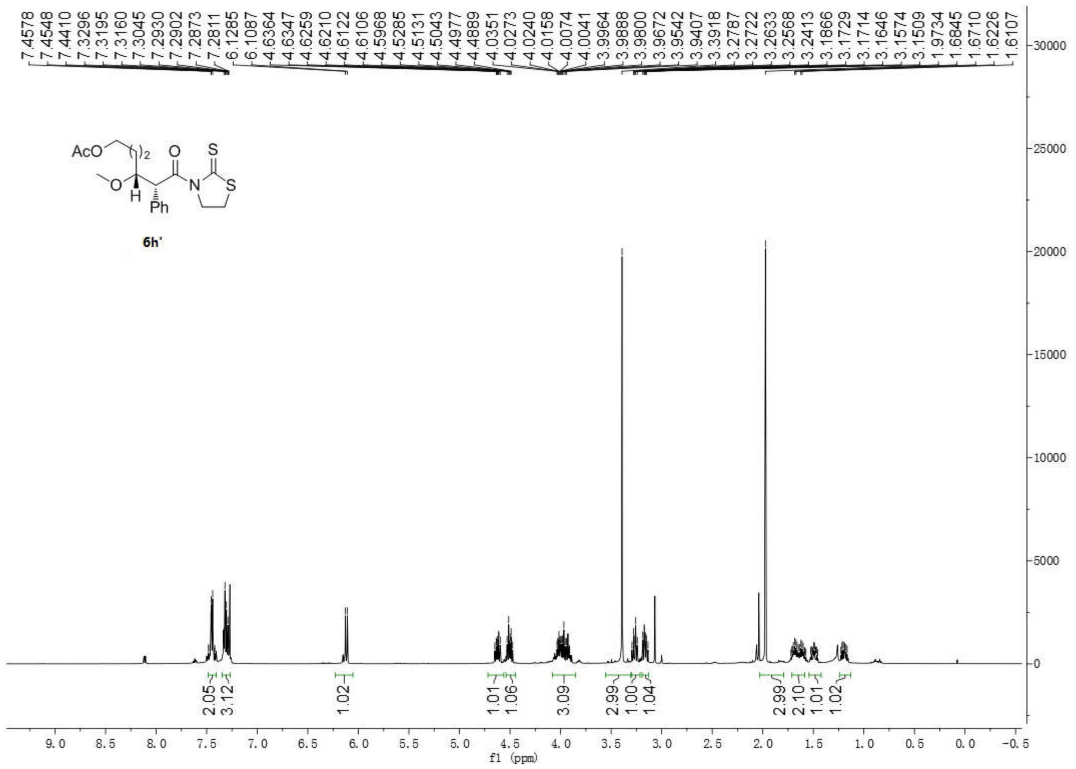

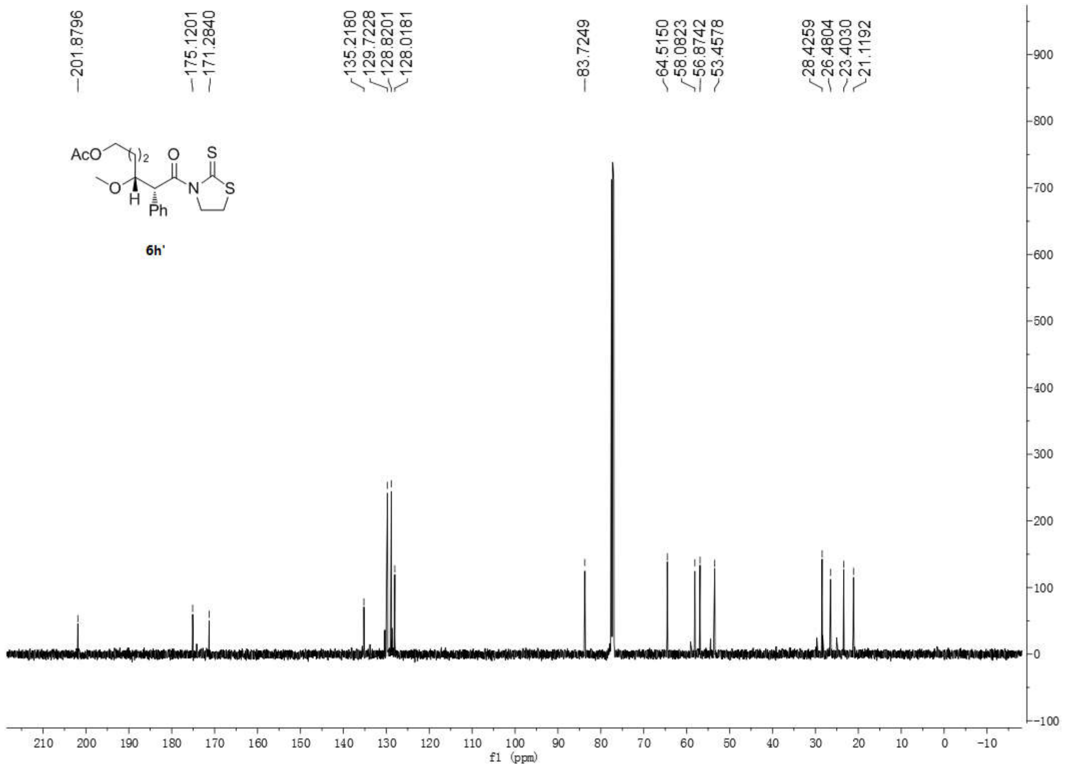

Supplementary figure 142. ^{1}H and ^{13}C NMR spectrum of compound **6h'**

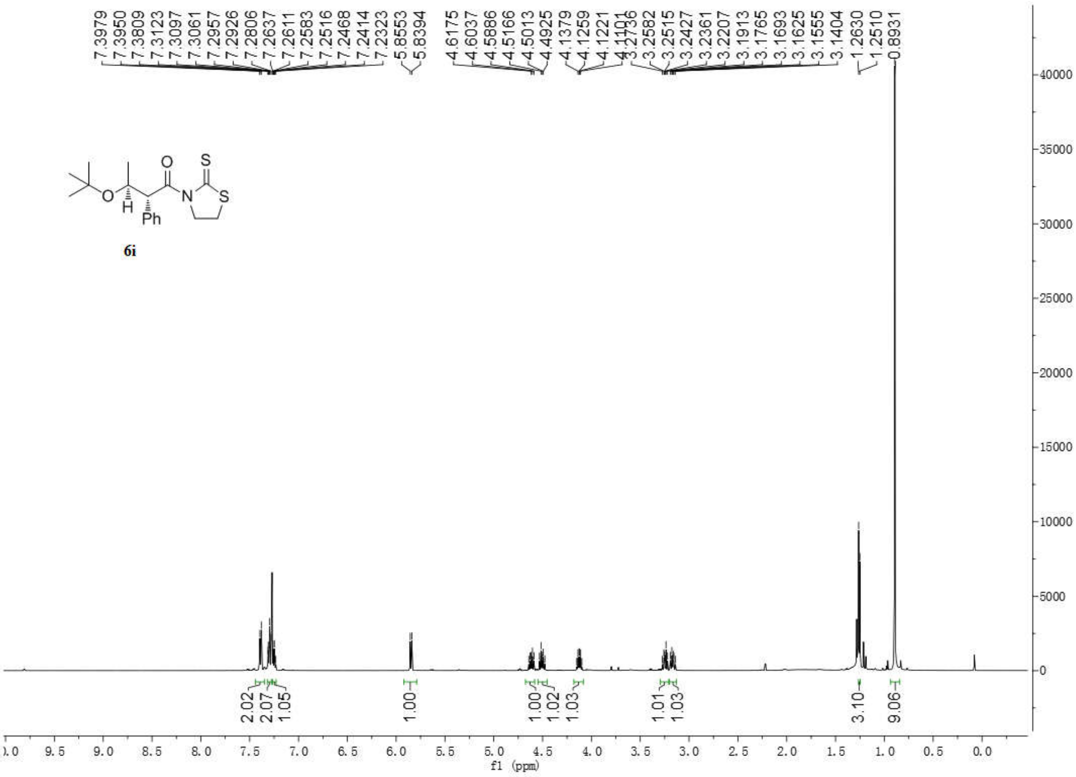

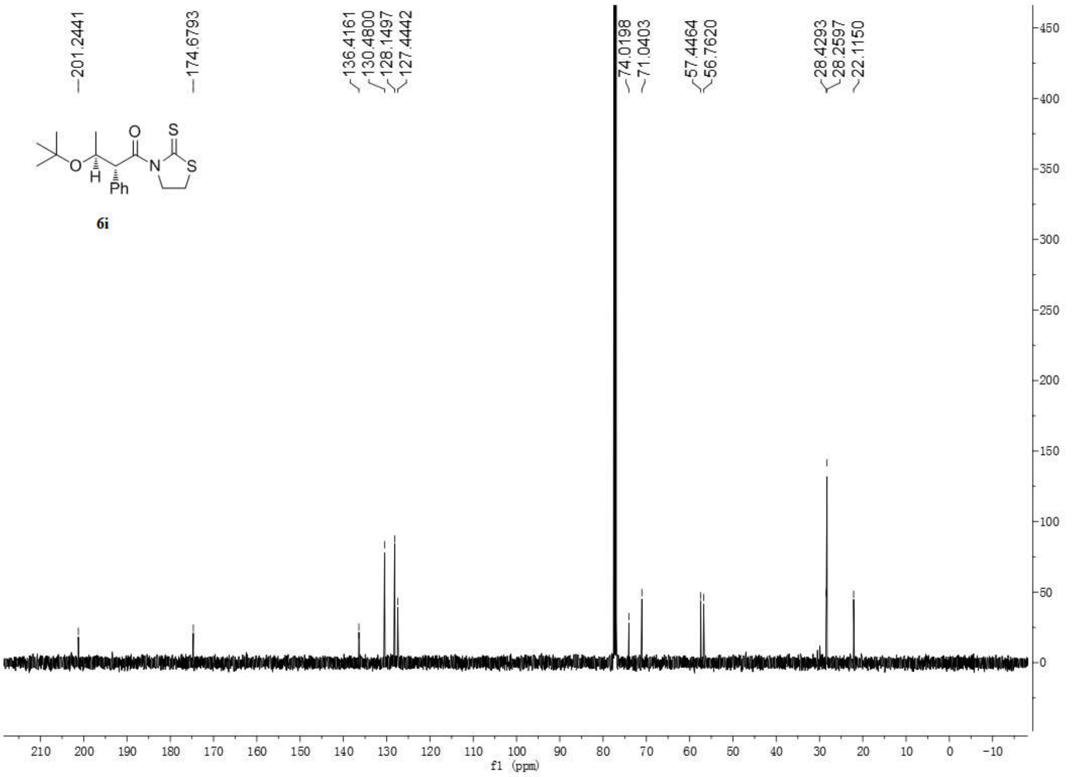

Supplementary figure 143. ^{1}H and ^{13}C NMR spectrum of compound **6i**

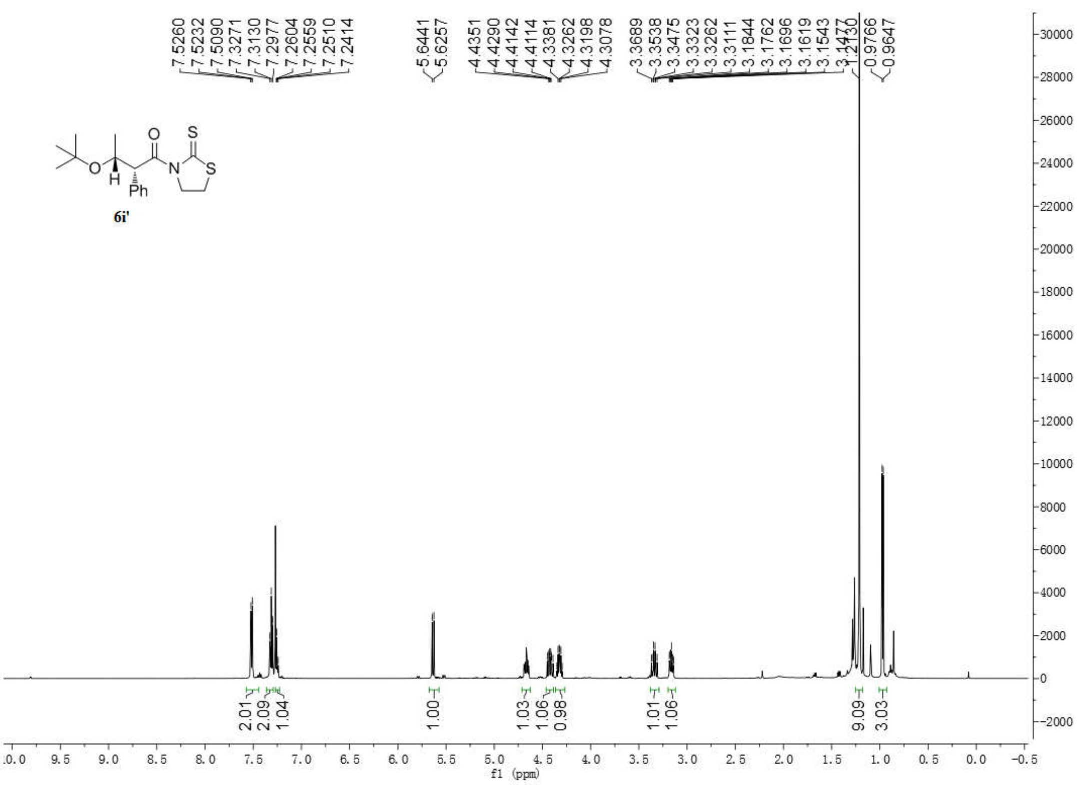

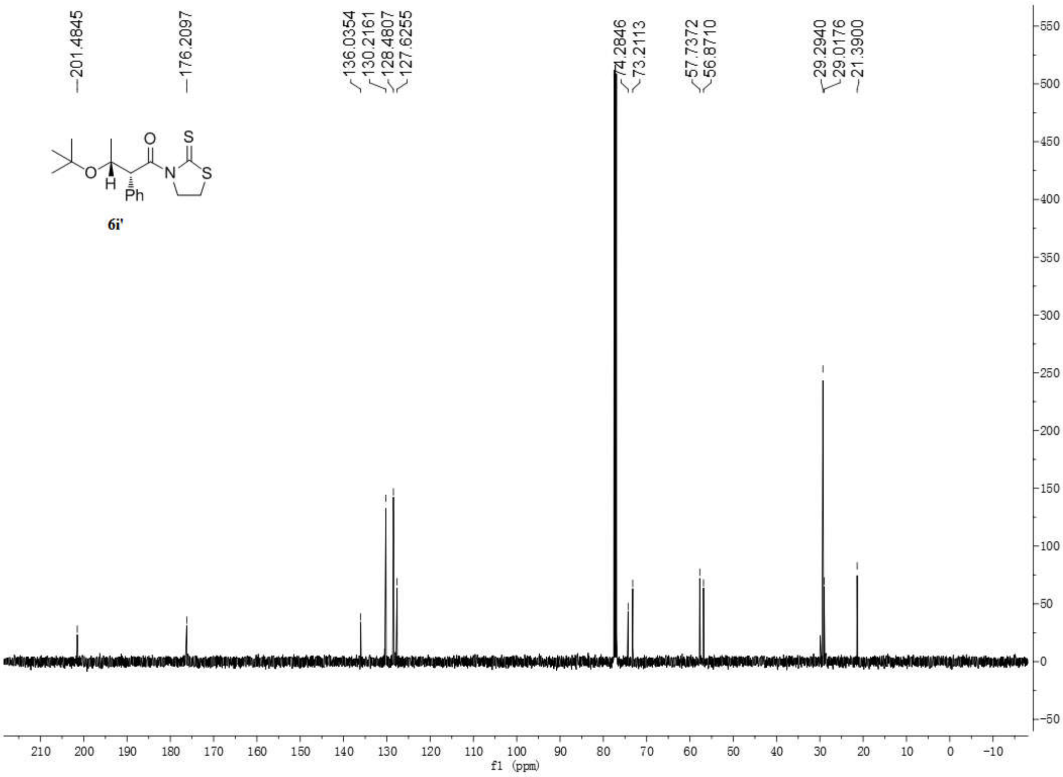

Supplementary figure 144. ^{1}H and ^{13}C NMR spectrum of compound **6i’**

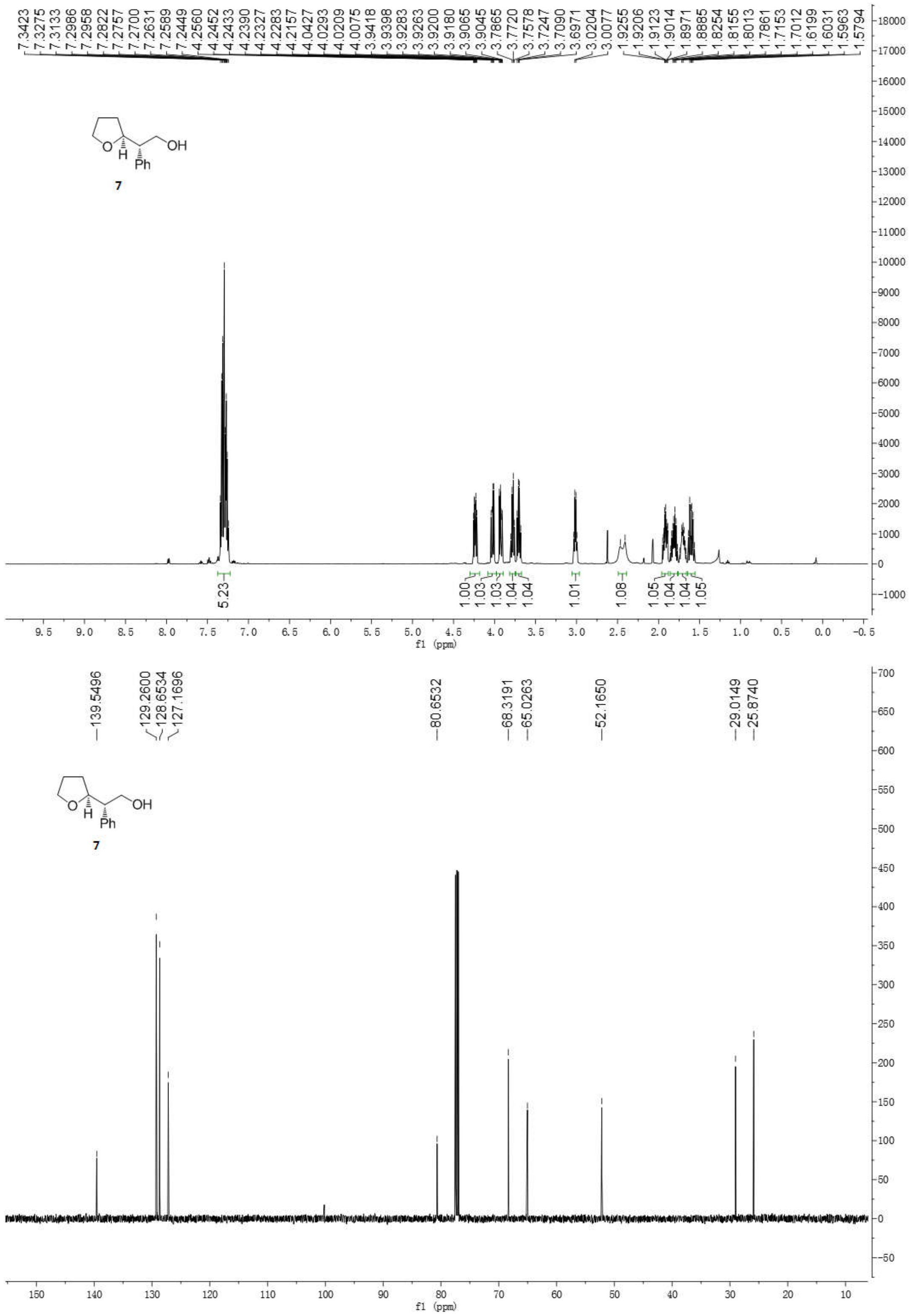

Supplementary figure 145. ^{1}H and ^{13}C NMR spectrum of compound **7**

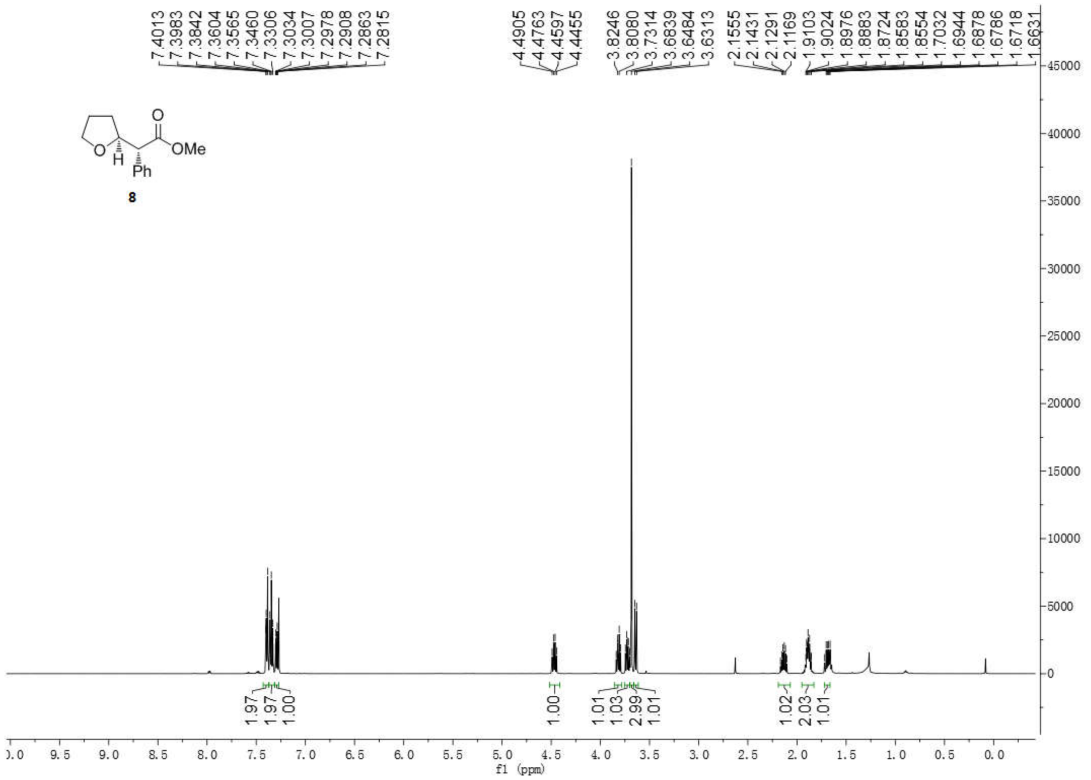

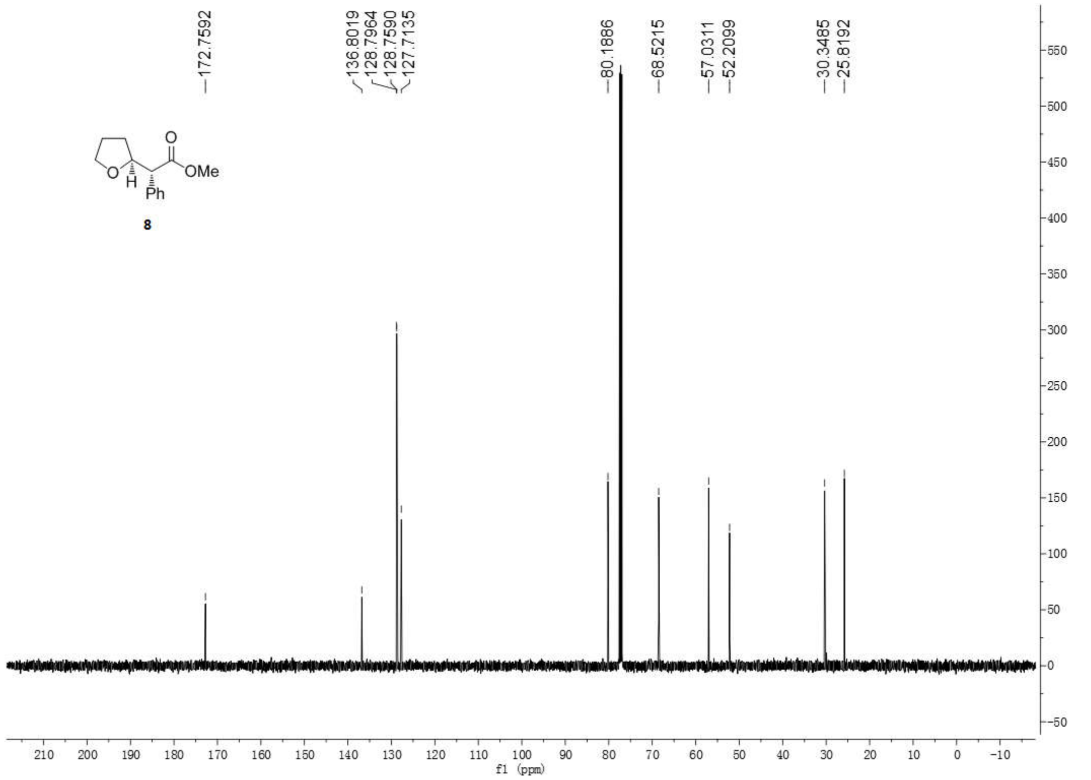

Supplementary figure 146. ^{1}H and ^{13}C NMR spectrum of compound **8**

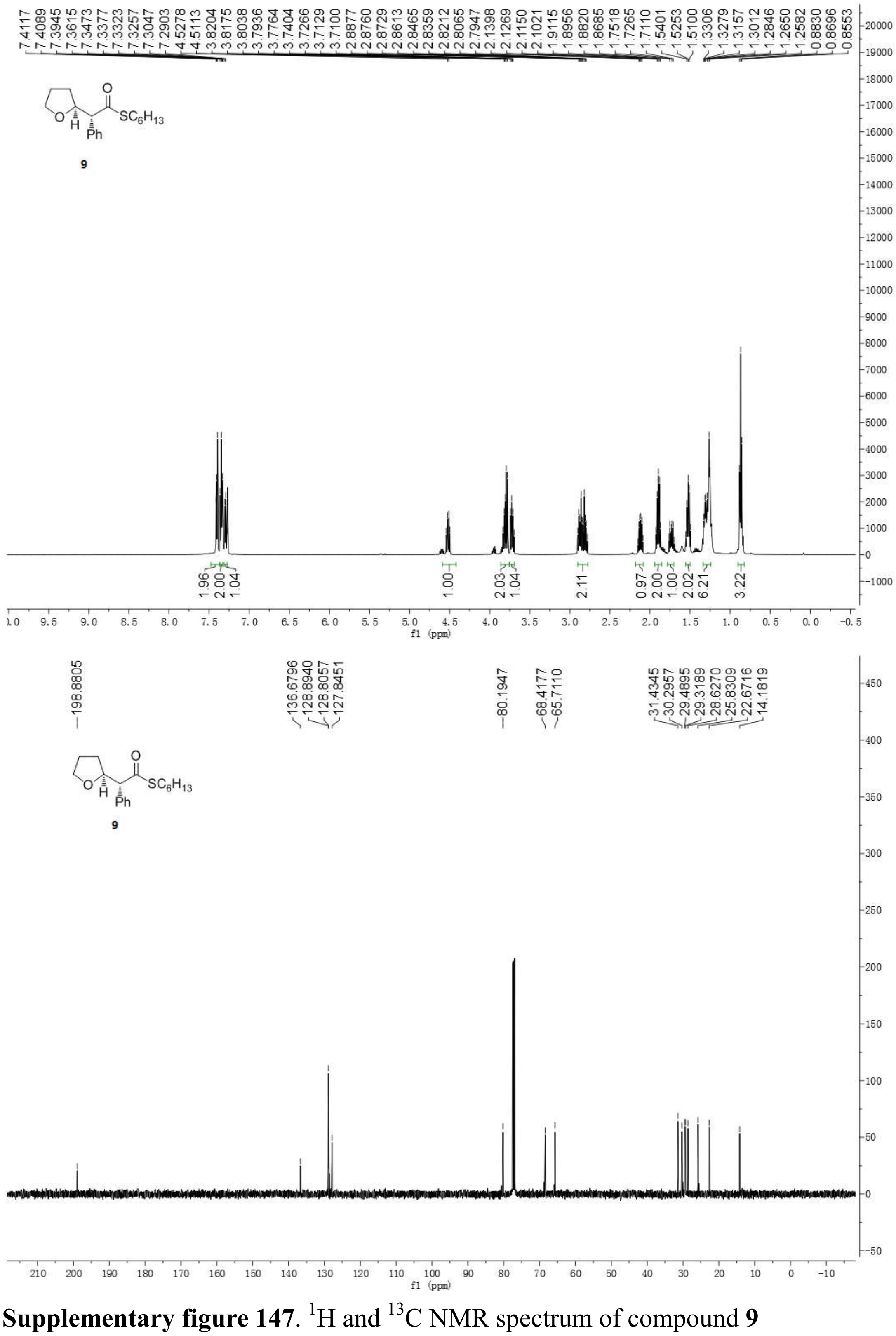

Supplementary figure 147. ^{1}H and ^{13}C NMR spectrum of compound **9**

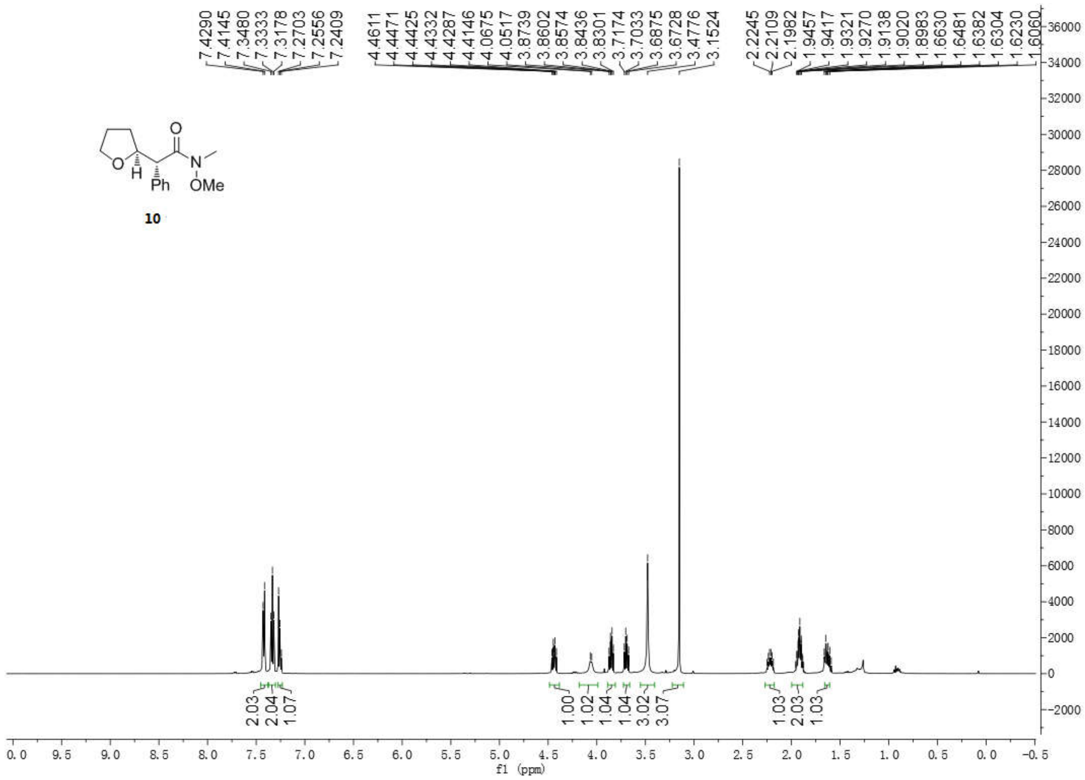

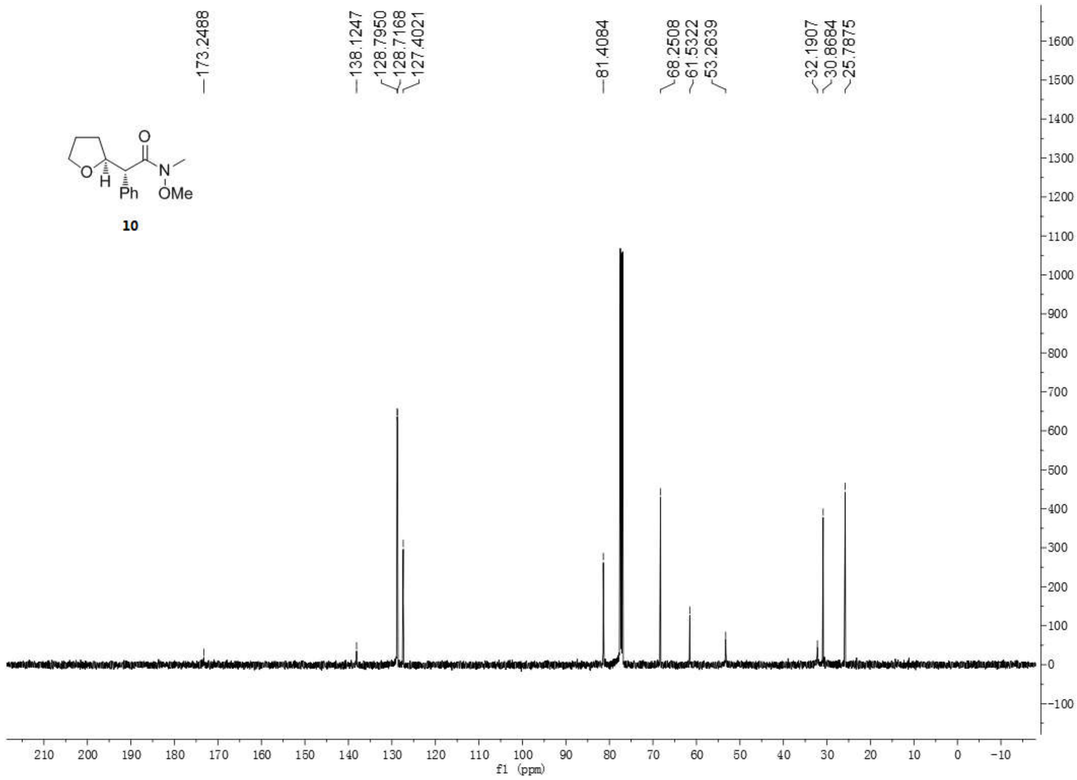

Supplementary figure 148. ^{1}H and ^{13}C NMR spectrum of compound **10**

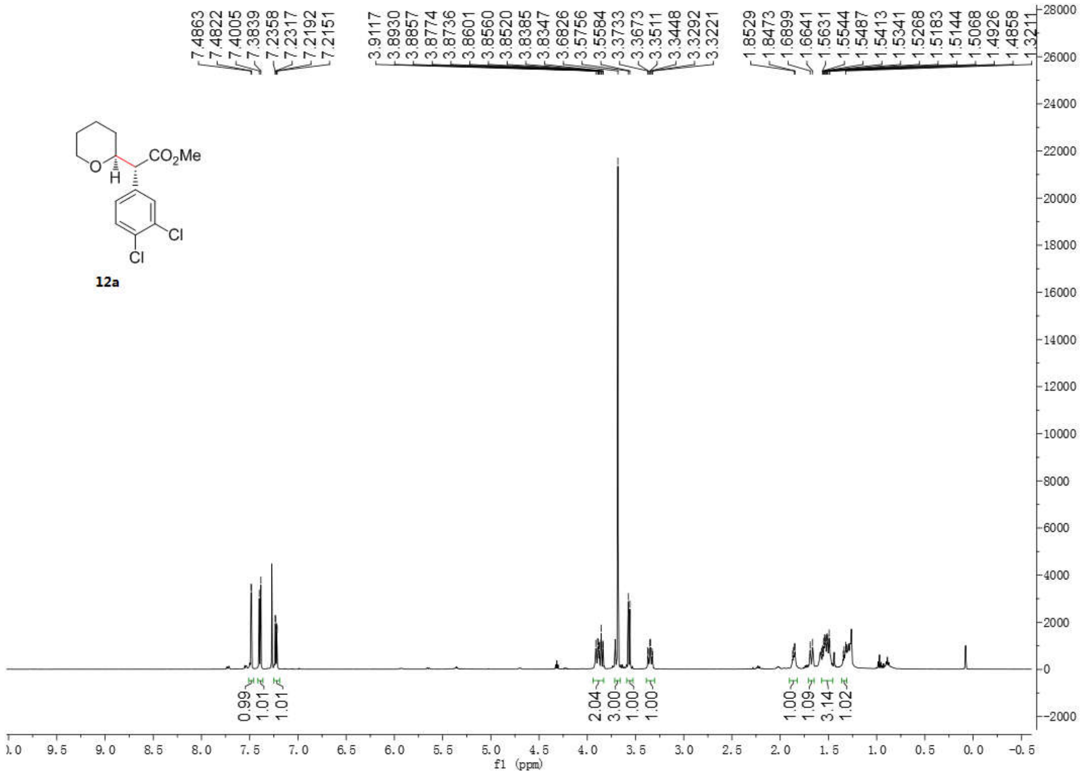

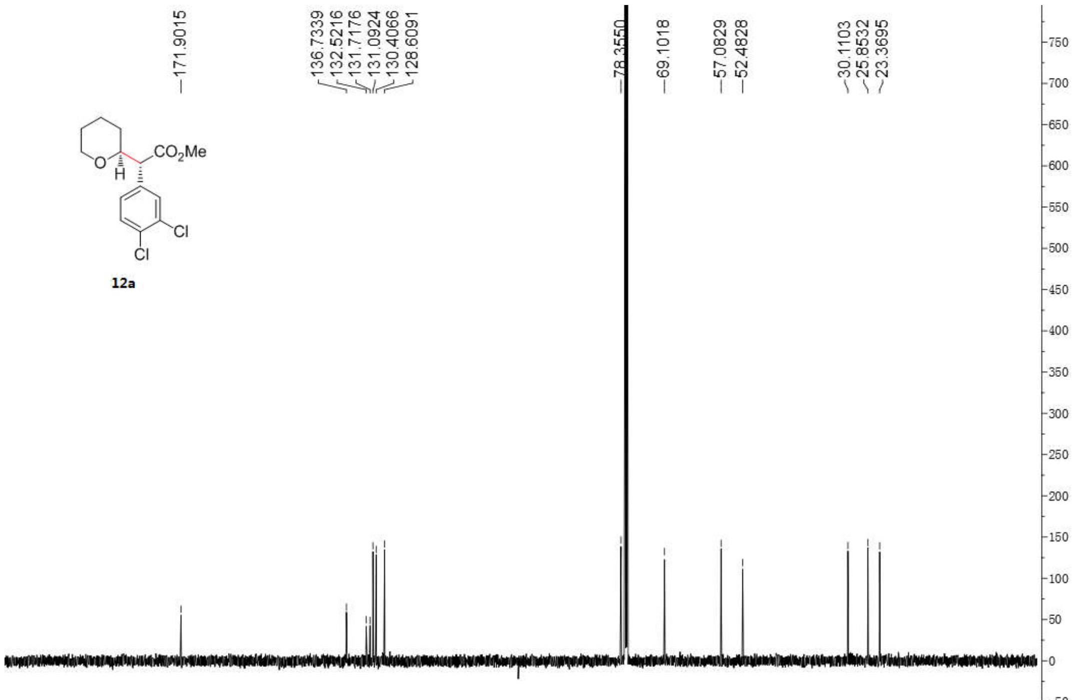

Supplementary figure 149. ^{1}H and ^{13}C NMR spectrum of compound **12a**

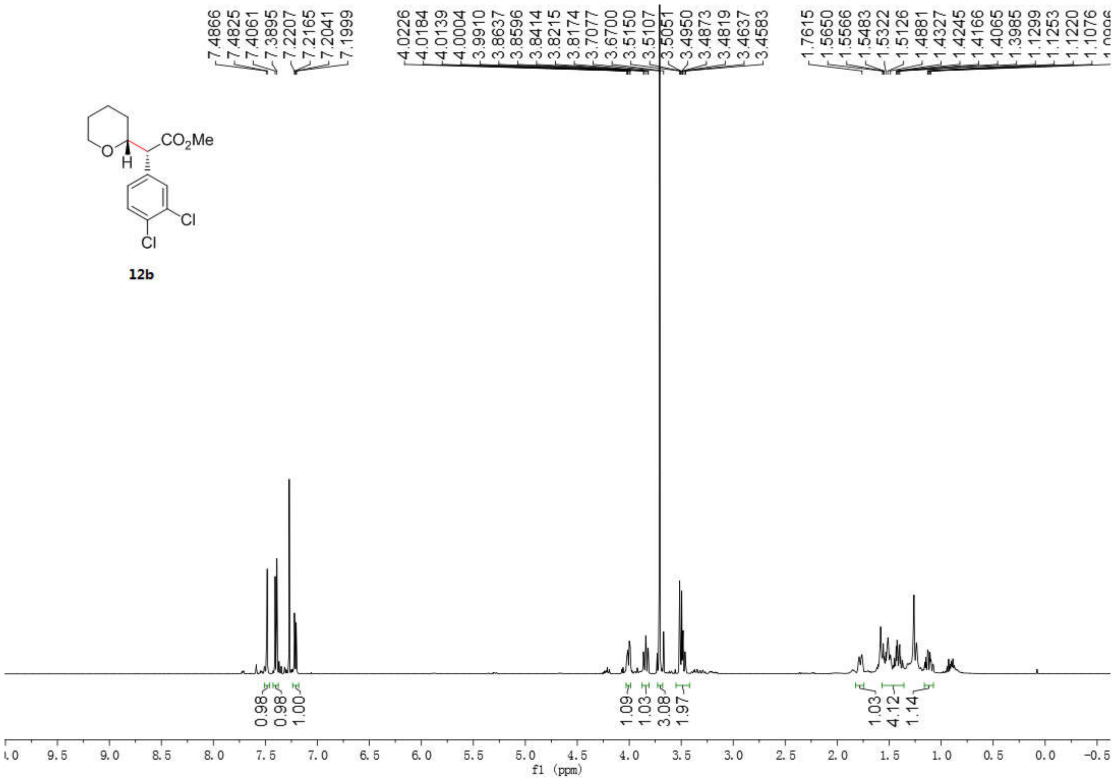

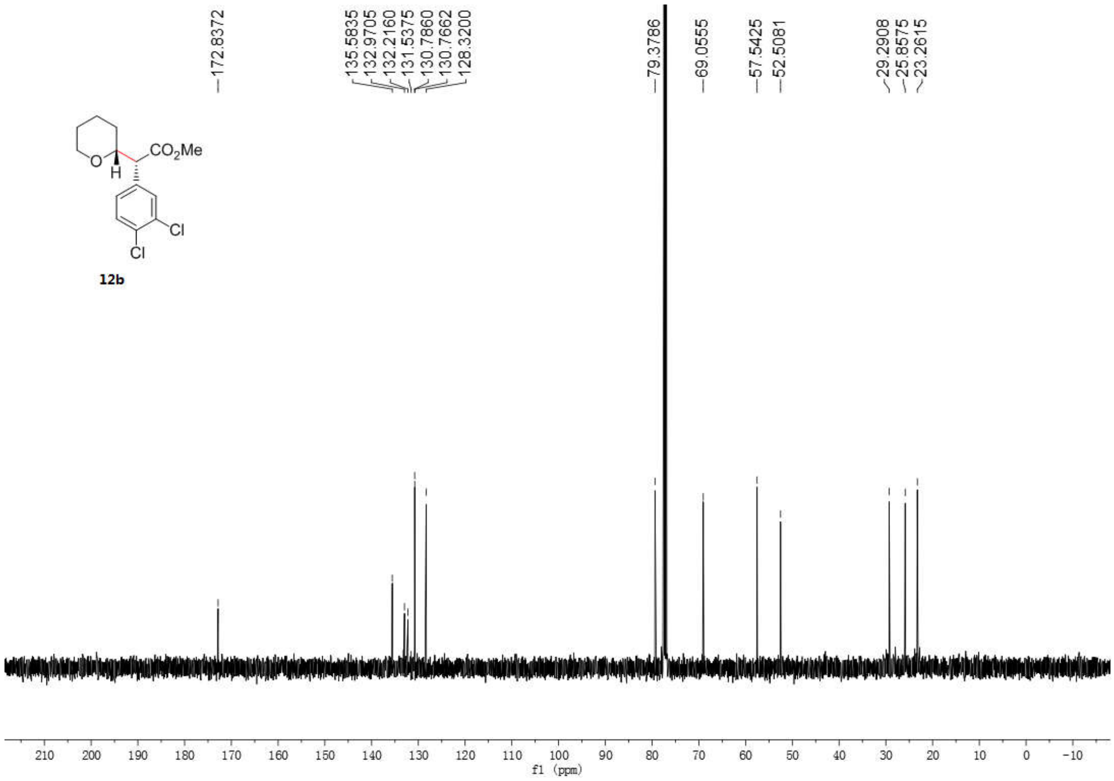

Supplementary figure 150. ^{1}H and ^{13}C NMR spectrum of compound **12b**

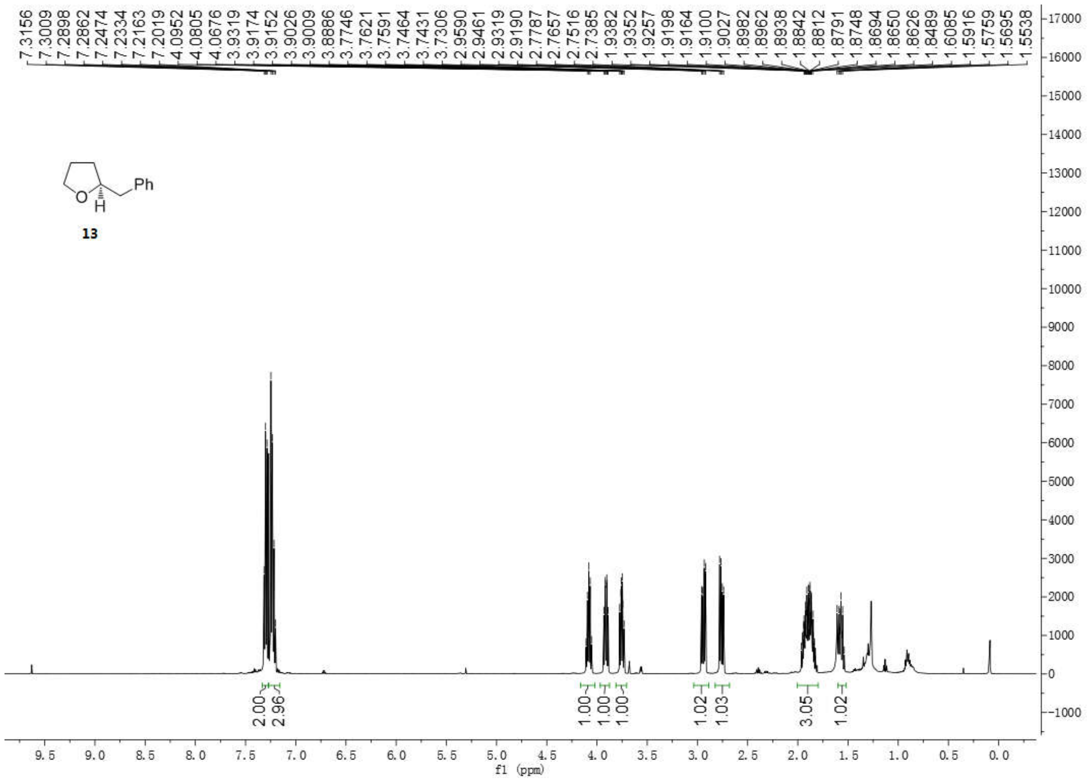

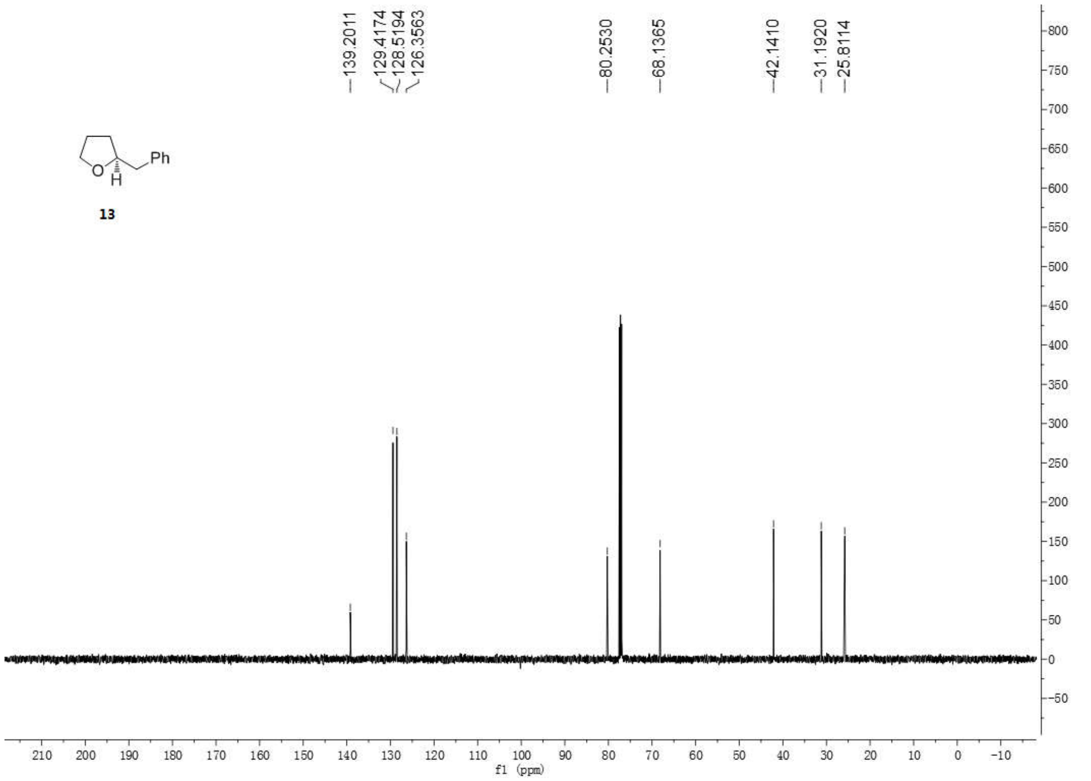

Supplementary figure 151. ^{1}H and ^{13}C NMR spectrum of compound **13**

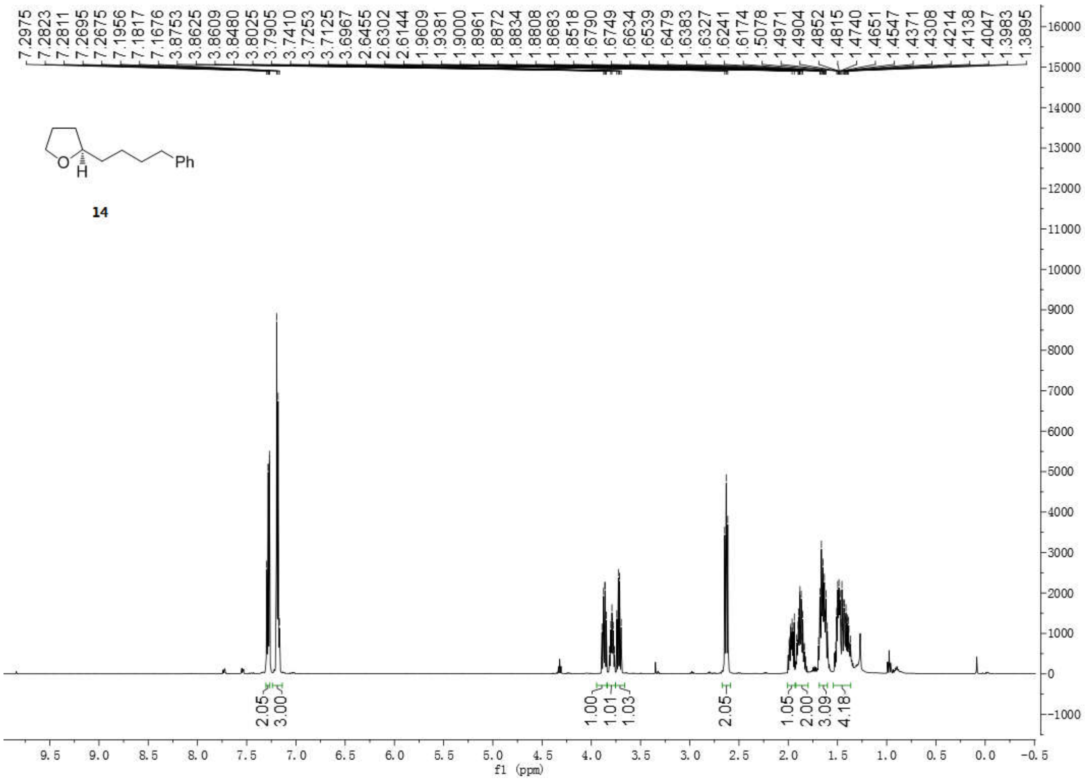

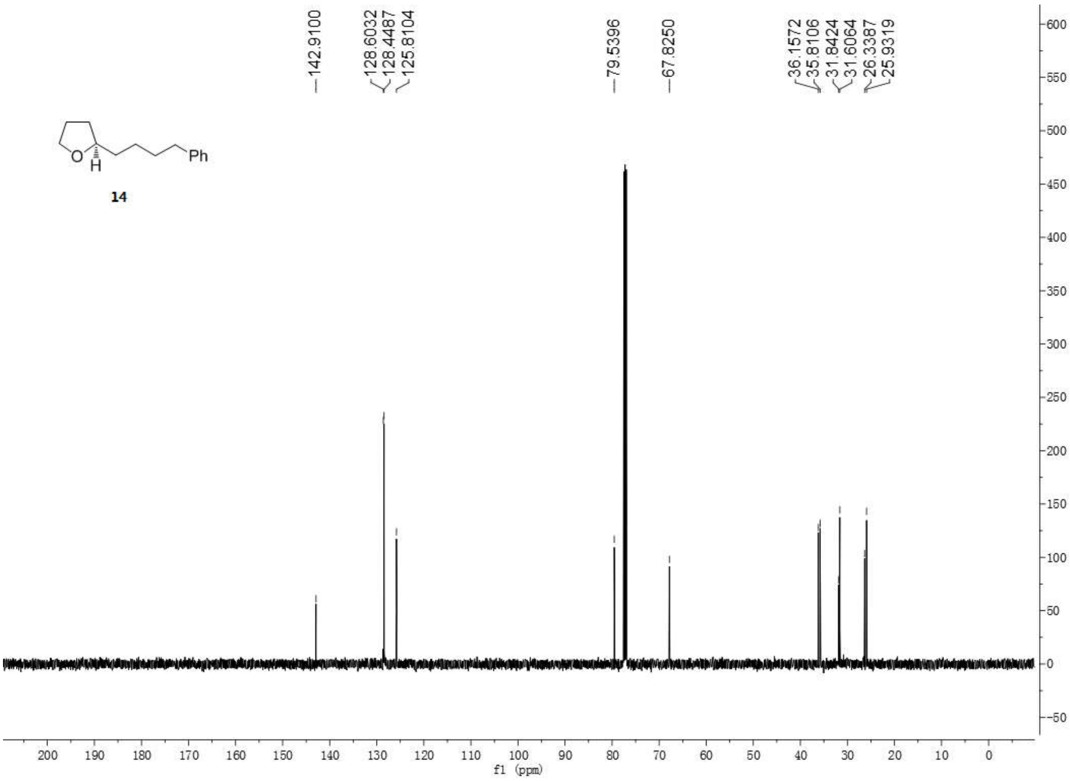

Supplementary figure 152. ^{1}H and ^{13}C NMR spectrum of compound **14**

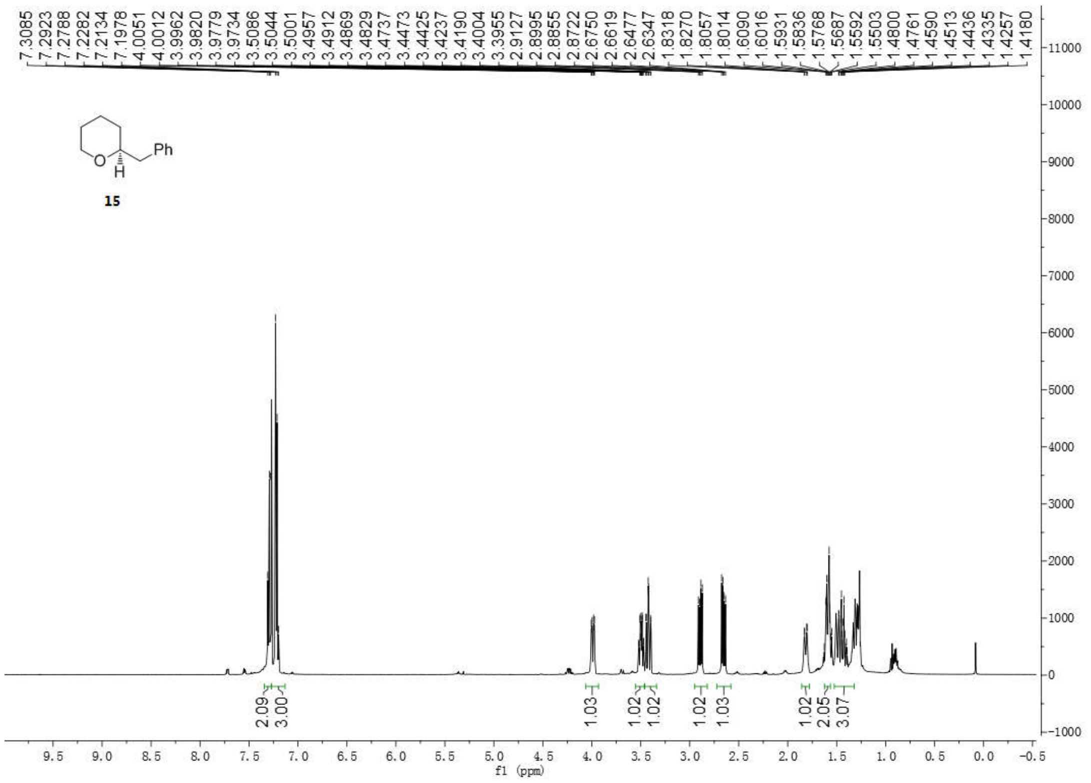

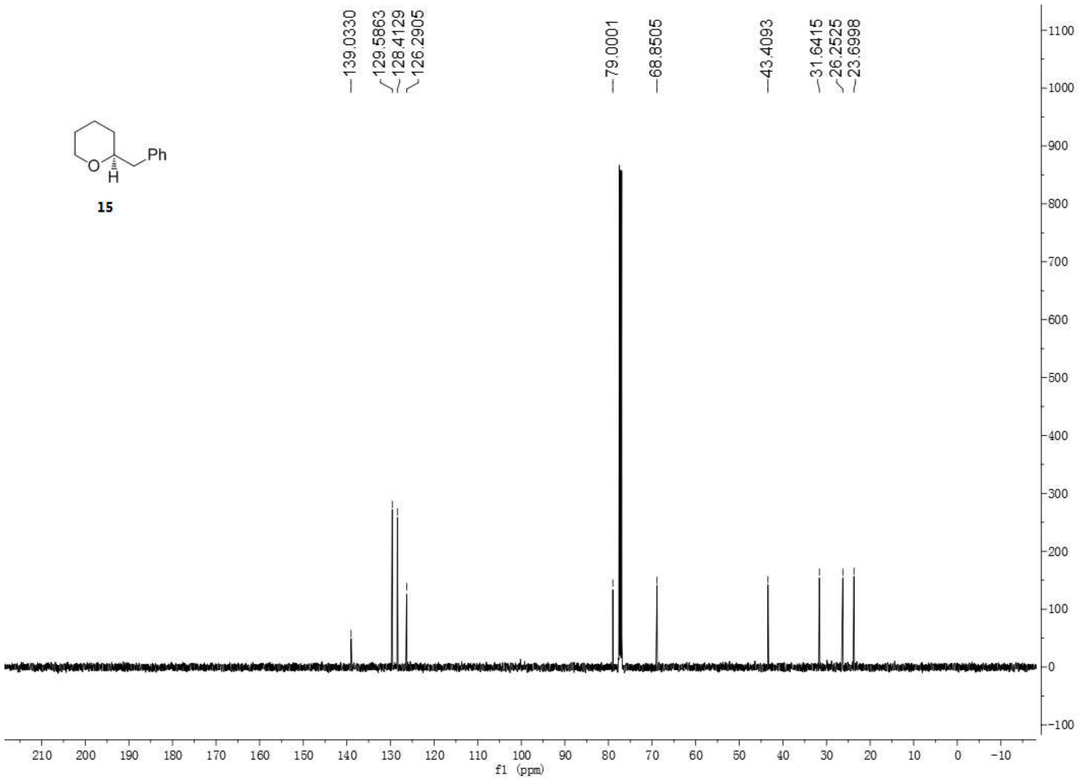

Supplementary figure 153. ^{1}H and ^{13}C NMR spectrum of compound **15**

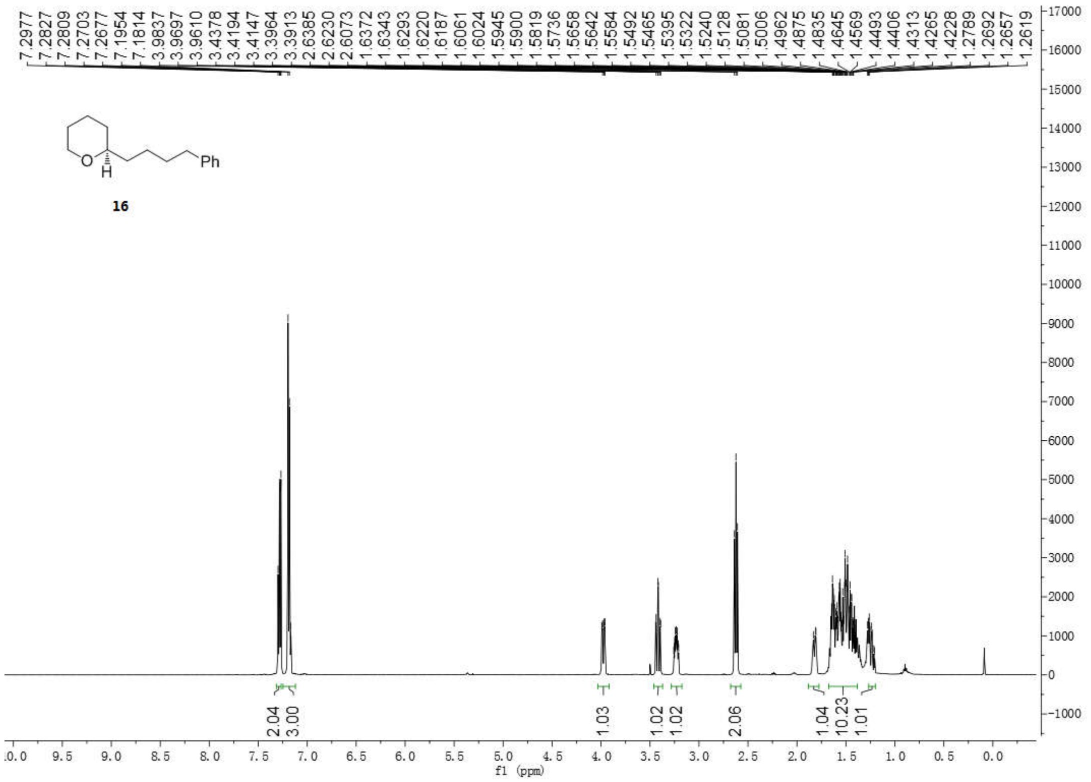

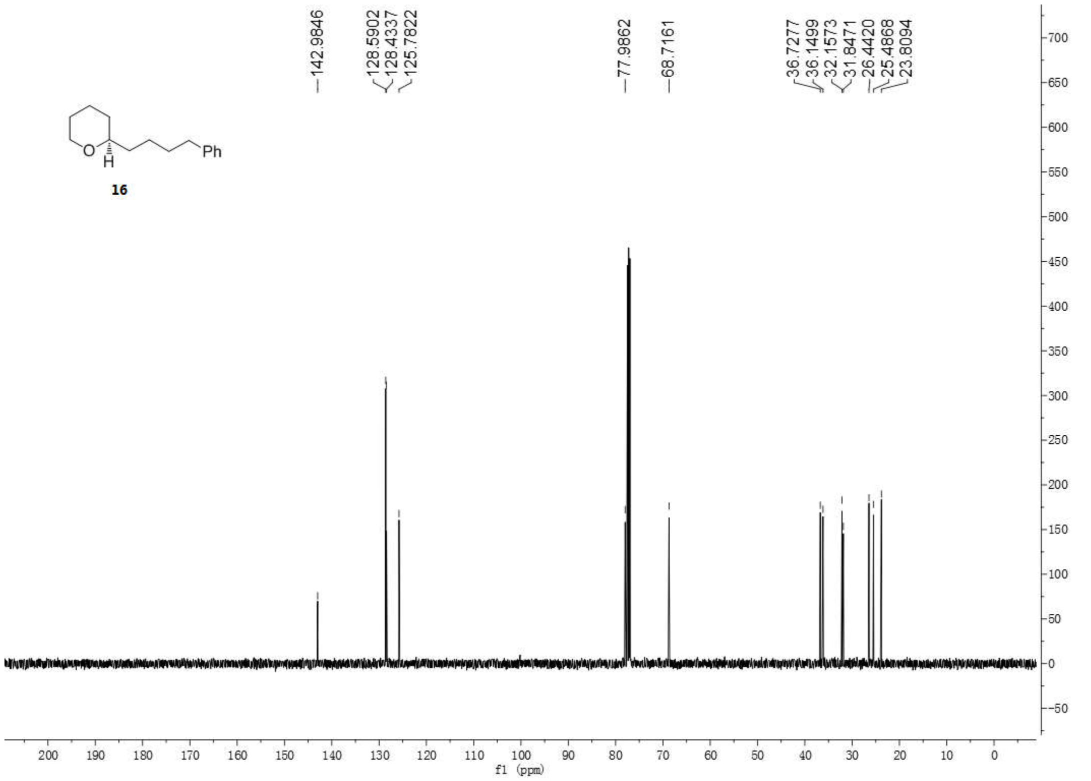

Supplementary figure 154. ^{1}H and ^{13}C NMR spectrum of compound **16**

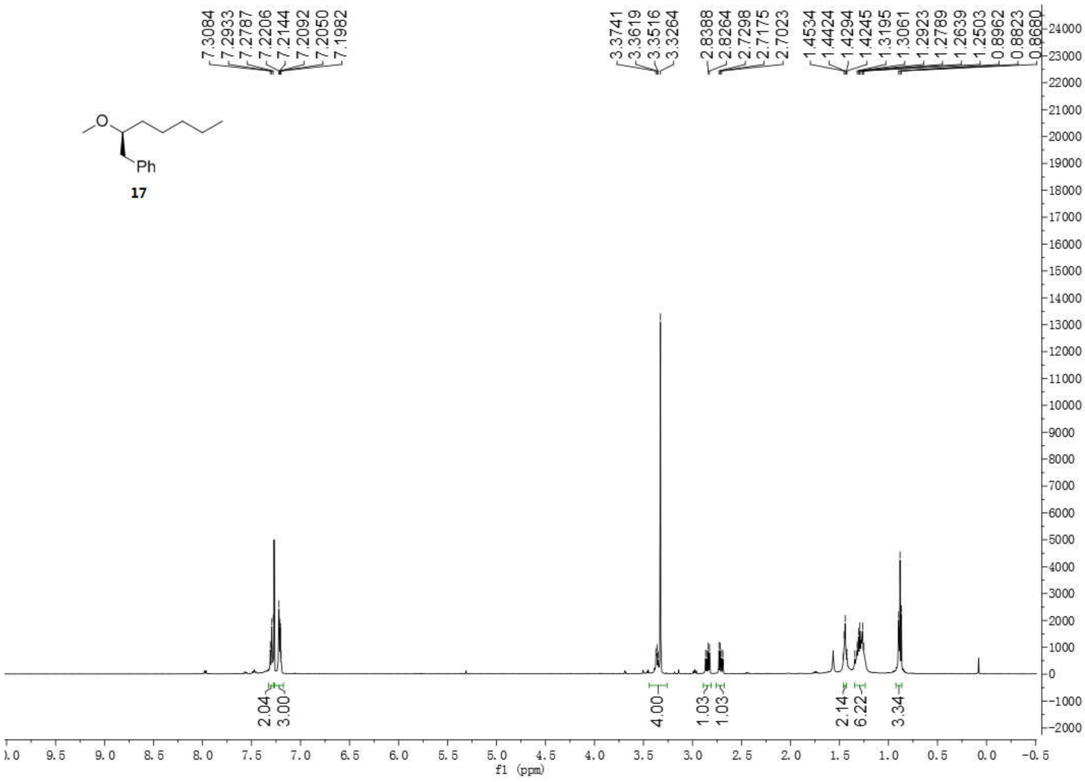

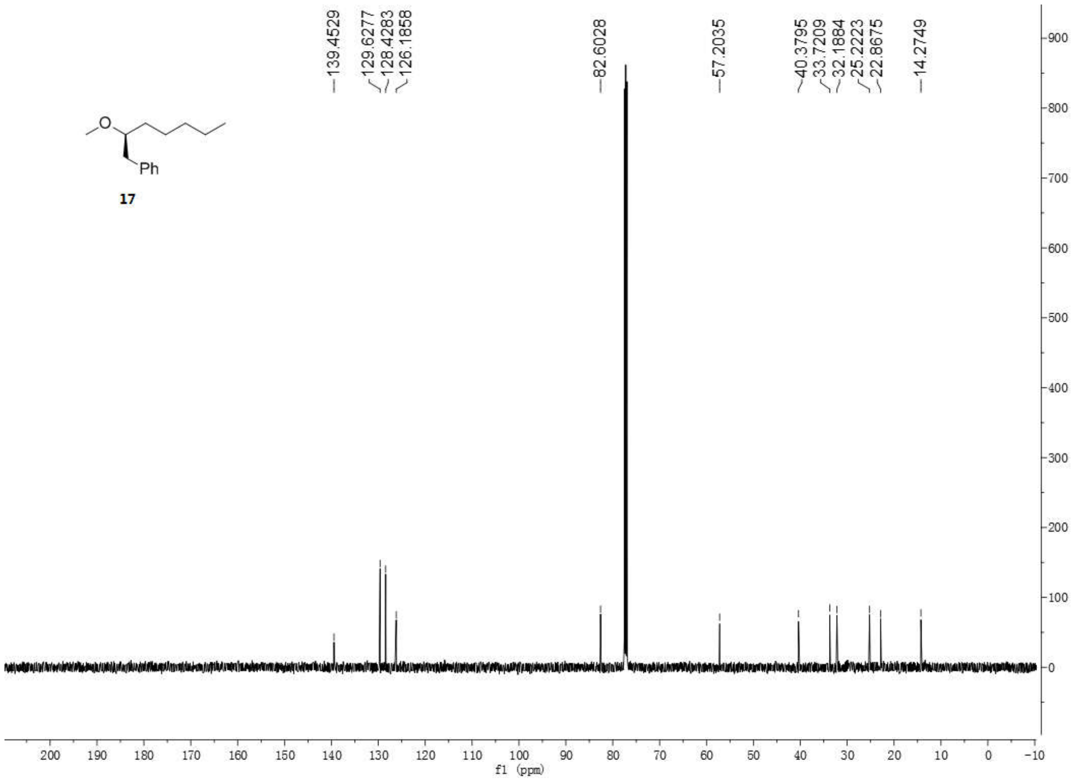

Supplementary figure 155. ^{1}H and ^{13}C NMR spectrum of compound **17**

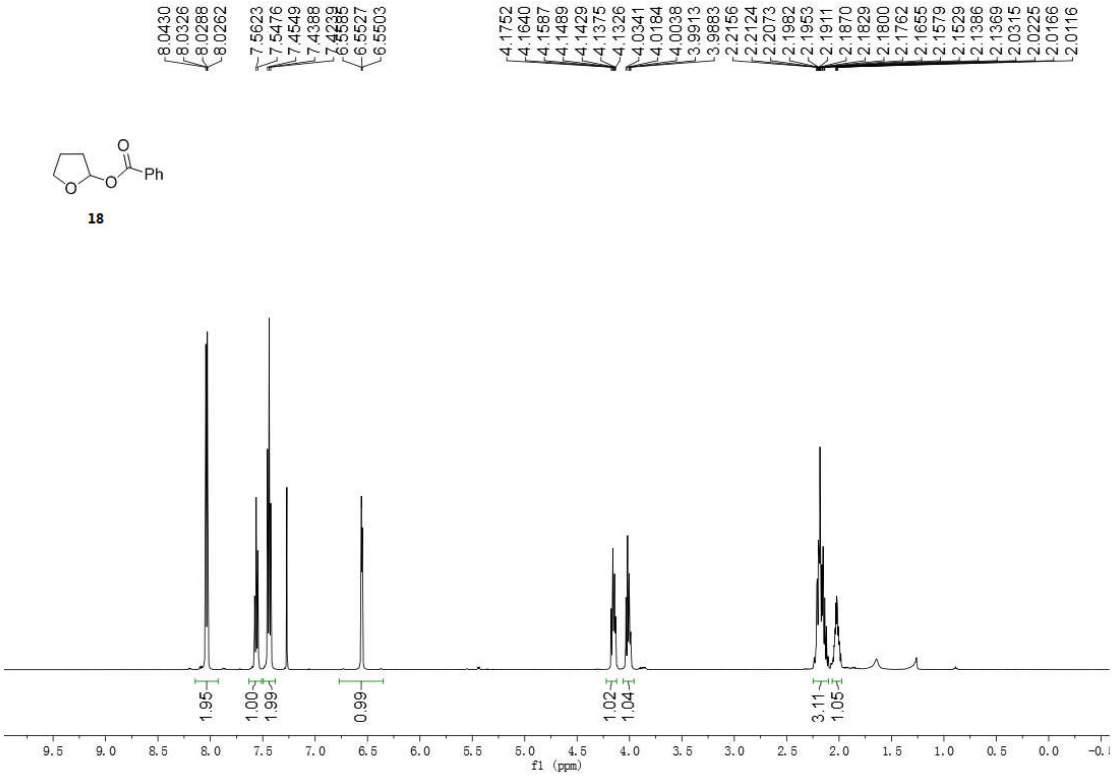

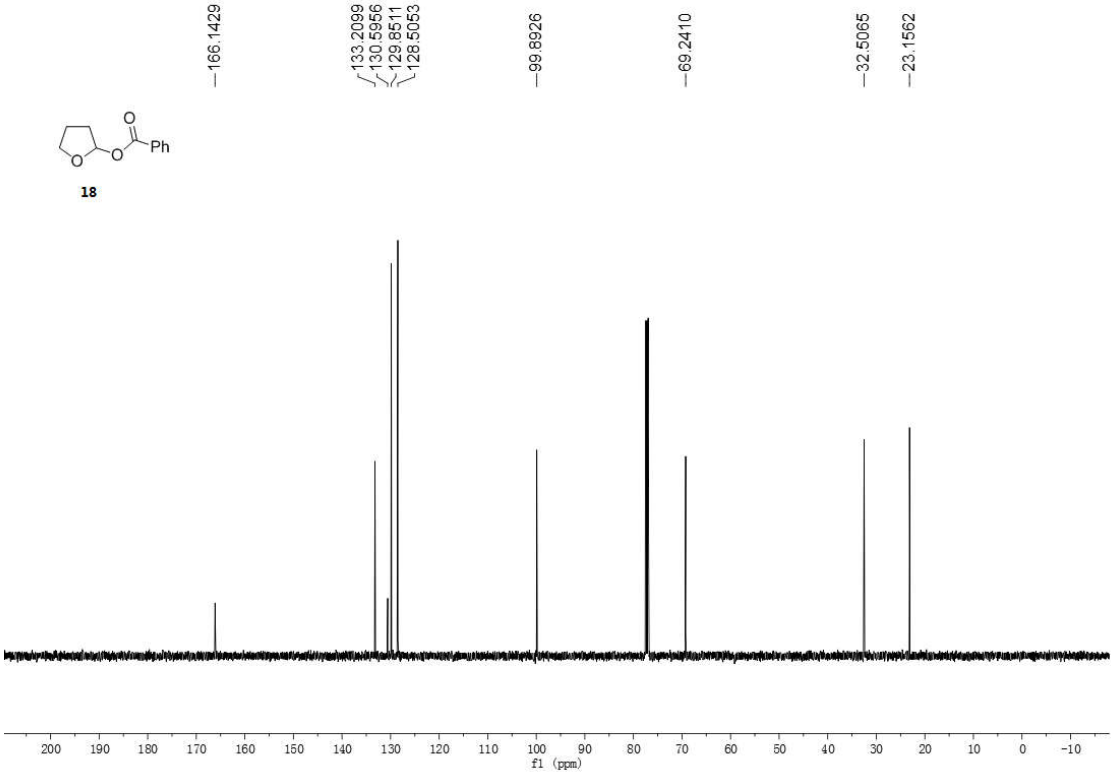

Supplementary figure 156. ^{1}H and ^{13}C NMR spectrum of compound **18**

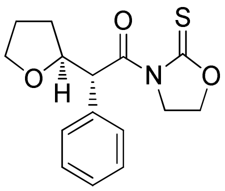

3aa

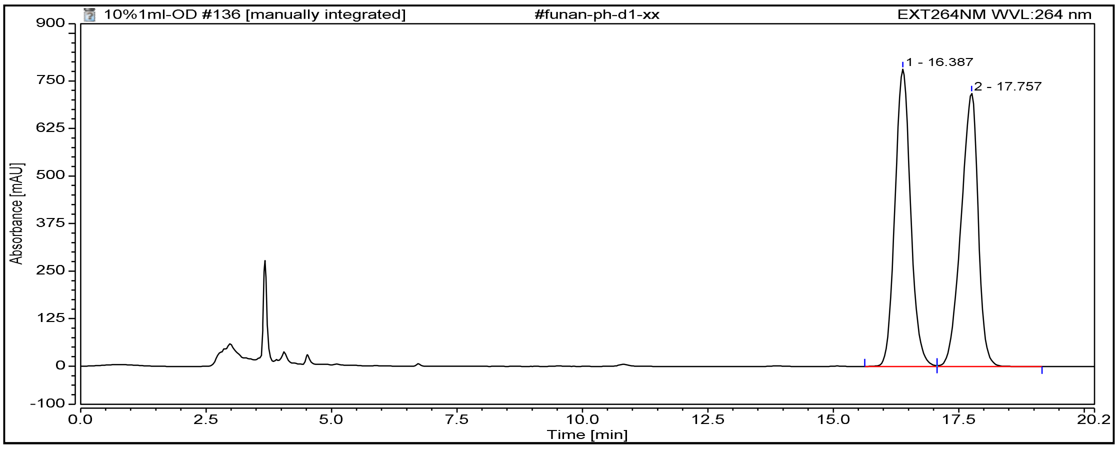

Integration Results					
No.	Peak Name	Retention Time<br>min	Area<br>mAU*min	Relative Area<br>%	Amount<br>n.a.
1		16.387	283.419	49.97	n.a.
2		17.757	283.803	50.03	n.a.
**Total:**			**567.222**	**100.00**	

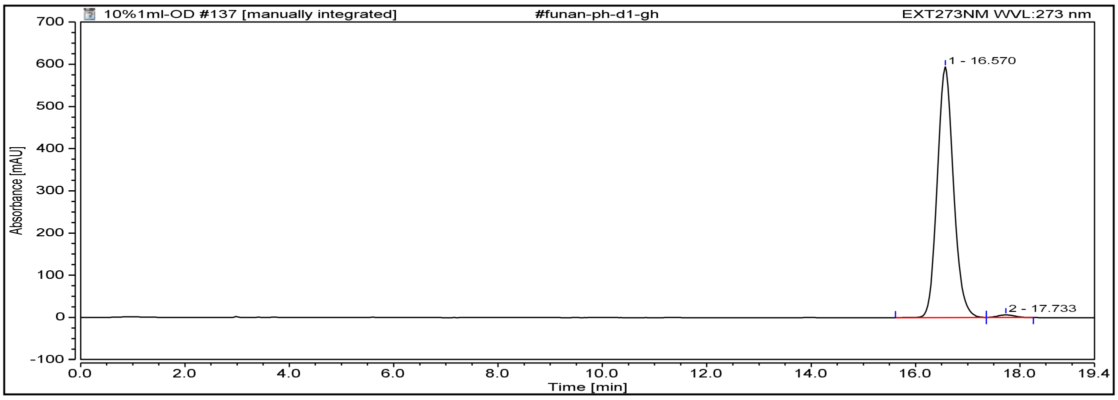

Integration Results					
No.	Peak Name	Retention Time<br>min	Area<br>mAU*min	Relative Area<br>%	Amount<br>n.a.
1		16.570	214.743	98.87	n.a.
2		17.733	2.455	1.13	n.a.
**Total:**			**217.198**	**100.00**	

Supplementary figure 157. HPLC chromatogram for compound **3aa**

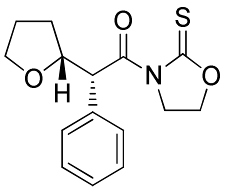

3aa’

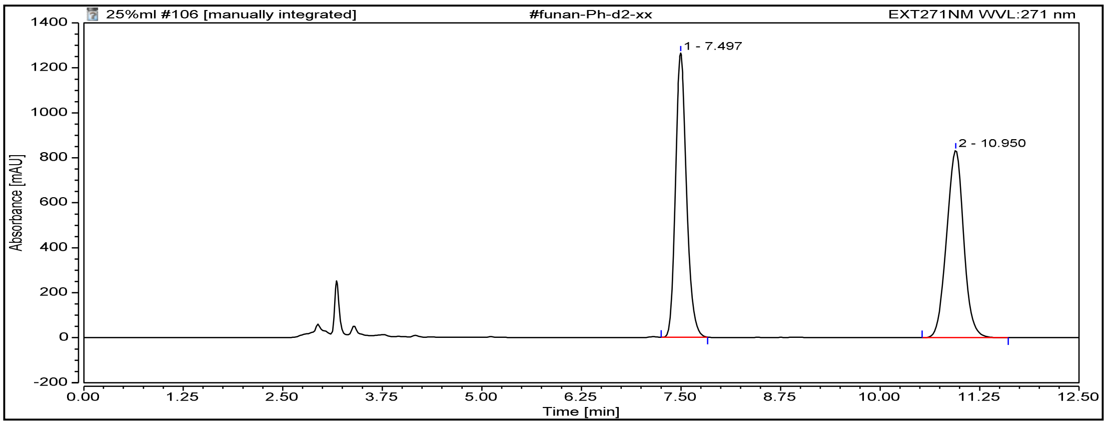

**Integration Results**					
No.	Peak Name	Retention Time<br>min	Area<br>mAU*min	Relative Area<br>%	Amount<br>n.a.
1		7.497	205.228	49.83	n.a.
2		10.950	206.595	50.17	n.a.
**Total:**			**411.823**	**100.00**	

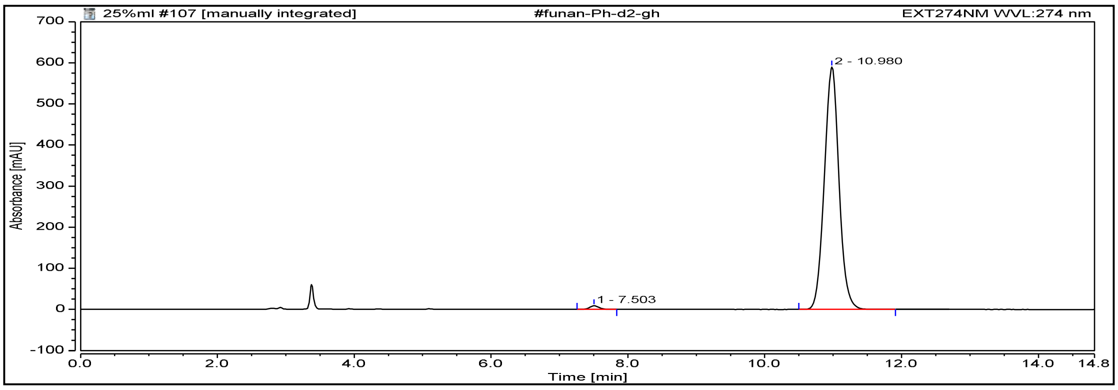

**Integration Results**					
No.	Peak Name	Retention Time<br>min	Area<br>mAU*min	Relative Area<br>%	Amount<br>n.a.
1		7.503	1.437	0.97	n.a.
2		10.980	146.639	99.03	n.a.
**Total:**			**148.076**	**100.00**	

Supplementary figure 158. HPLC chromatogram for compound **3aa’**

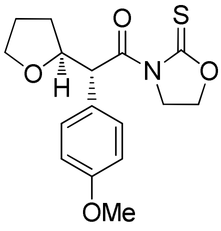

3b

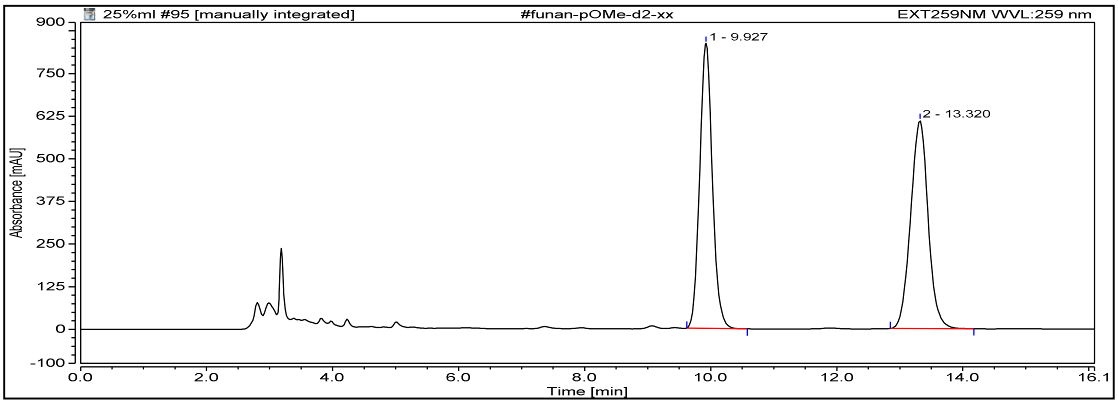

Integration Results					
No.	Peak Name	Retention Time<br>min	Area<br>mAU*min	Relative Area<br>%	Amount<br>n.a.
1		9.927	194.524	49.73	n.a.
2		13.320	196.656	50.27	n.a.
**Total:**			**391.180**	**100.00**	

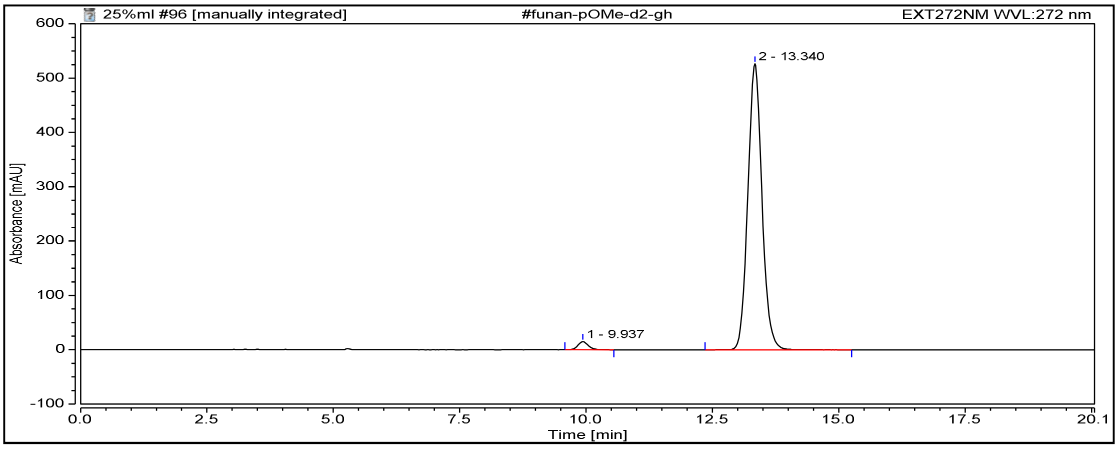

Integration Results					
No.	Peak Name	Retention Time<br>min	Area<br>mAU*min	Relative Area<br>%	Amount<br>n.a.
1		9.937	3.525	2.02	n.a.
2		13.340	171.168	97.98	n.a.
**Total:**			**174.693**	**100.00**	

Supplementary figure 159. HPLC chromatogram for compound **3b**

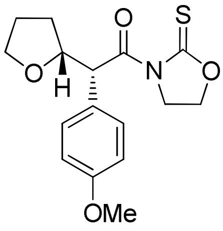

3b’

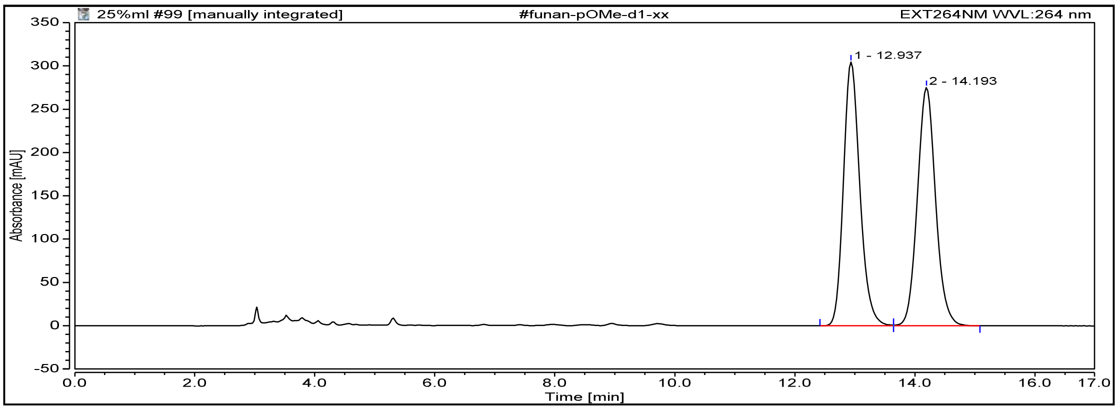

Integration Results					
No.	Peak Name	Retention Time<br>min	Area<br>mAU*min	Relative Area<br>%	Amount<br>n.a.
1		12.937	96.912	49.96	n.a.
2		14.193	97.082	50.04	n.a.
**Total:**			**193.994**	**100.00**	

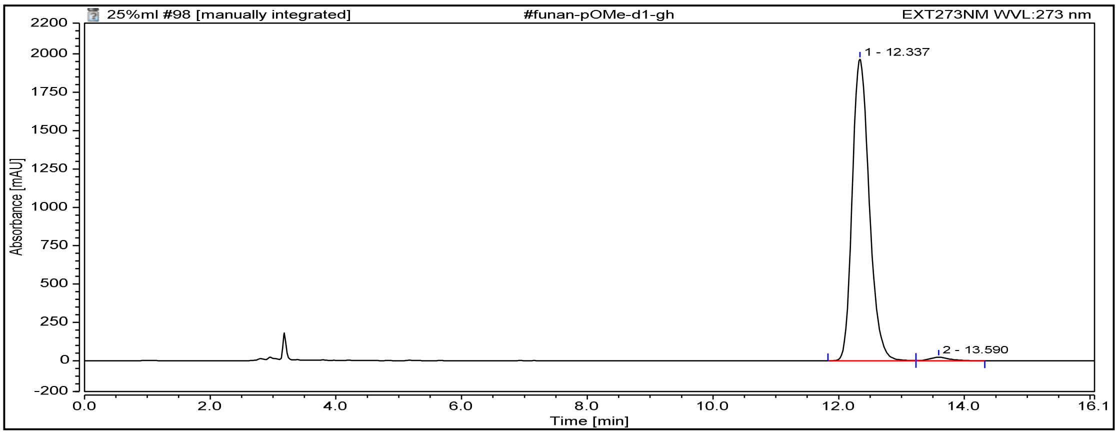

Integration Results					
No.	Peak Name	Retention Time<br>min	Area<br>mAU*min	Relative Area<br>%	Amount<br>n.a.
1		12.337	616.166	98.65	n.a.
2		13.590	8.405	1.35	n.a.
**Total:**			**624.570**	**100.00**	

Supplementary figure 160. HPLC chromatogram for compound **3b’**

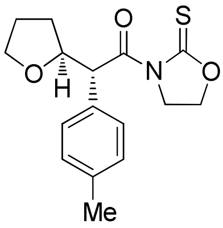

3c

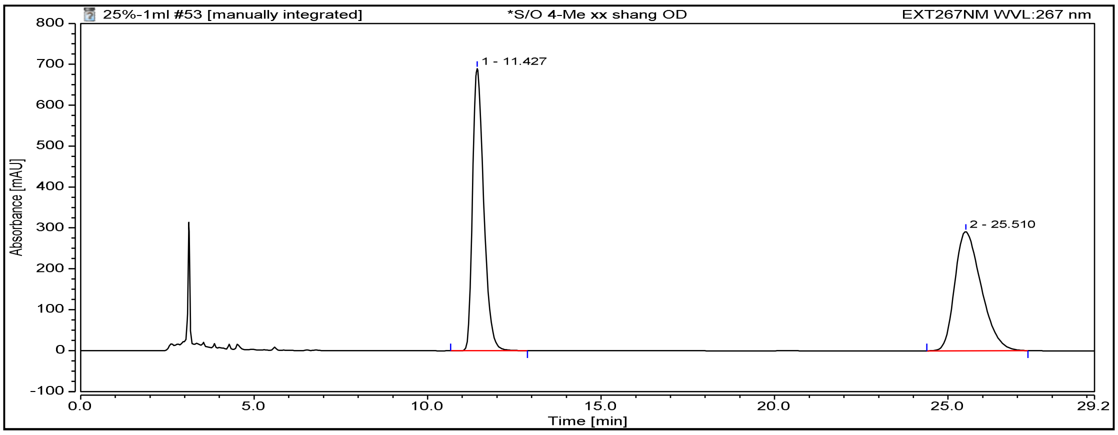

Integration Results					
No.	Peak Name	Retention Time<br>min	Area<br>mAU*min	Relative Area<br>%	Amount<br>n.a.
1		11.427	256.949	49.99	n.a.
2		25.510	257.070	50.01	n.a.
**Total:**			**514.019**	**100.00**	

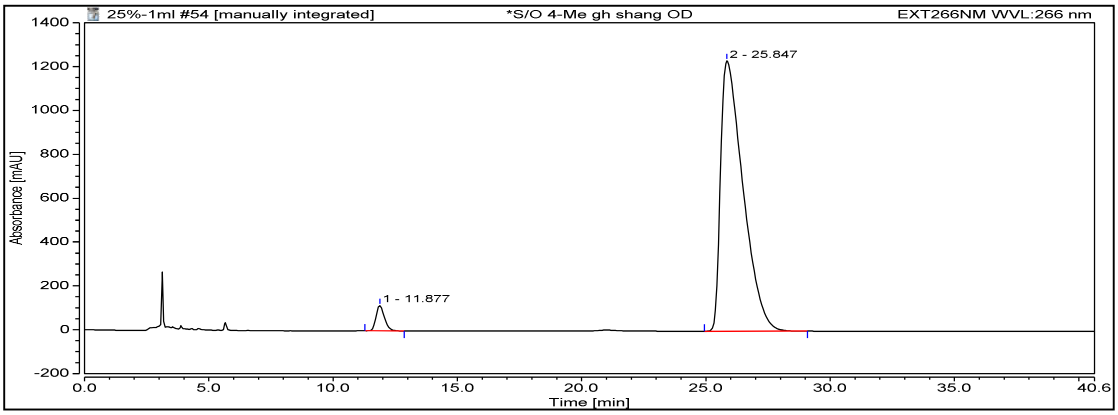

Integration Results					
No.	Peak Name	Retention Time<br>min	Area<br>mAU*min	Relative Area<br>%	Amount<br>n.a.
1		11.877	45.490	3.41	n.a.
2		25.847	1290.276	96.59	n.a.
**Total:**			**1335.765**	**100.00**	

Supplementary figure 161. HPLC chromatogram for compound **3c**

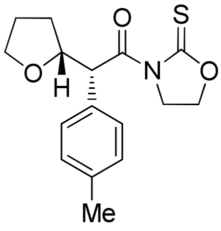

3c’

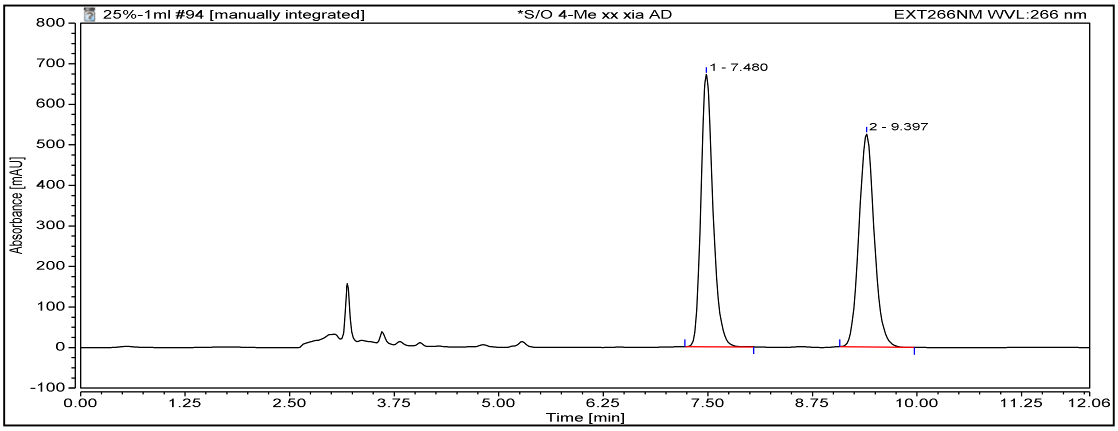

Integration Results					
No.	Peak Name	Retention Time min	Area mAU*min	Relative Area %	Amount n.a.
1		7.480	112.095	50.16	n.a.
2		9.397	111.368	49.84	n.a.
**Total:**			**223.463**	**100.00**	

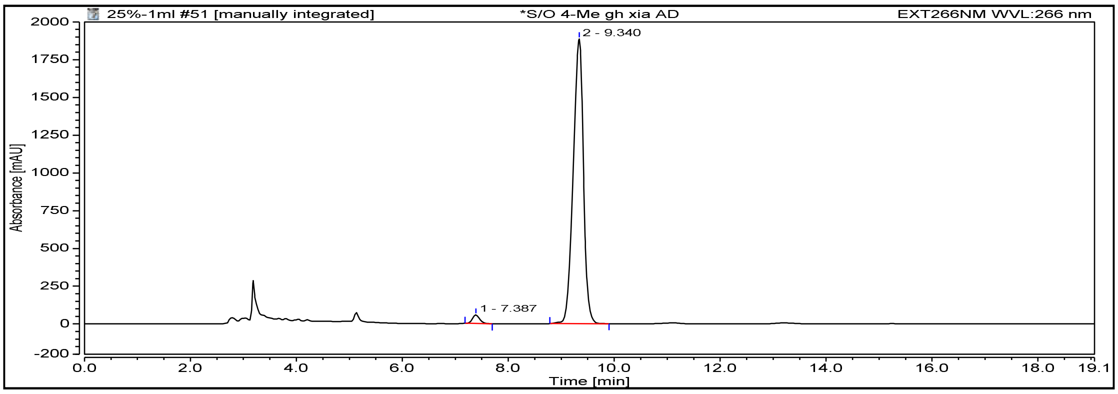

Integration Results					
No.	Peak Name	Retention Time min	Area mAU*min	Relative Area %	Amount n.a.
1		7.387	8.759	2.03	n.a.
2		9.340	422.018	97.97	n.a.
**Total:**			**430.778**	**100.00**	

Supplementary figure 162. HPLC chromatogram for compound **3c’**

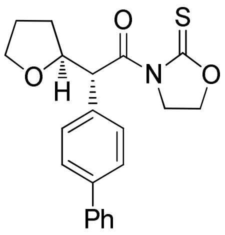

3d

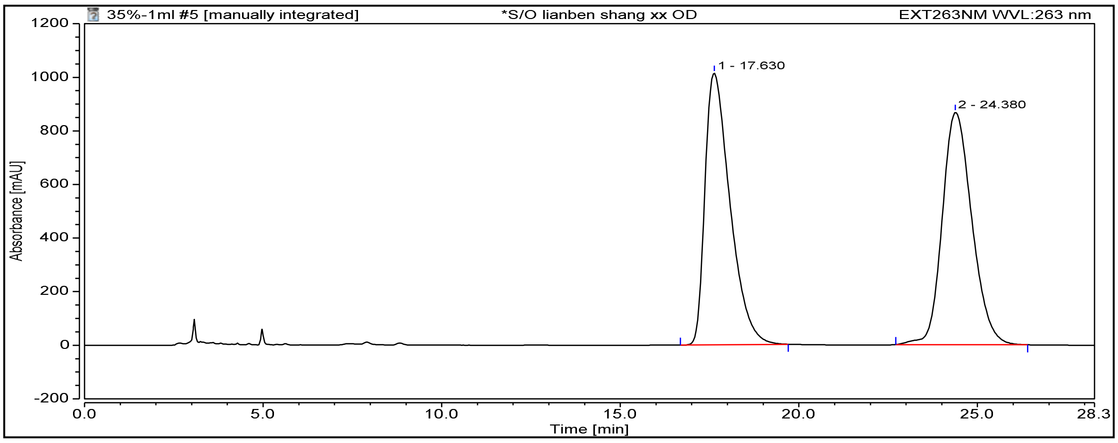

Integration Results					
No.	Peak Name	Retention Time<br>min	Area<br>mAU*min	Relative Area<br>%	Amount<br>n.a.
1		17.630	824.358	49.68	n.a.
2		24.380	835.050	50.32	n.a.
**Total:**			**1659.409**	**100.00**	

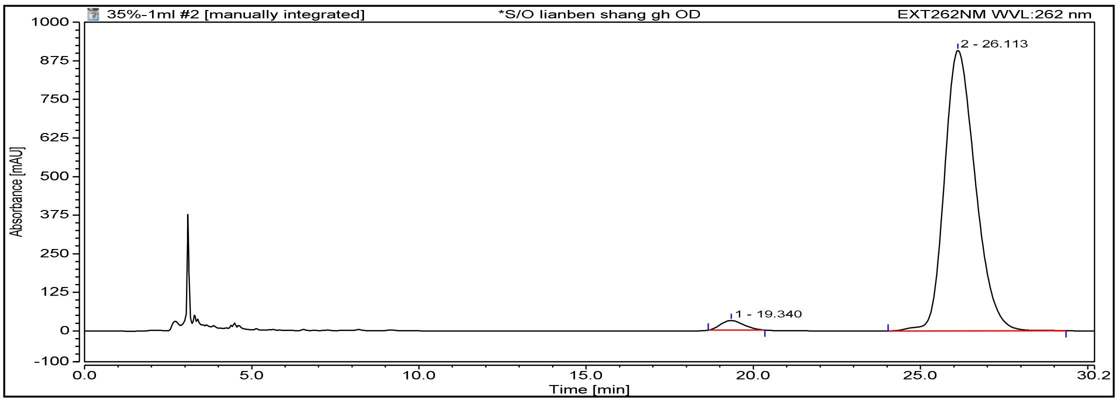

Integration Results					
No.	Peak Name	Retention Time<br>min	Area<br>mAU*min	Relative Area<br>%	Amount<br>n.a.
1		19.340	24.997	2.50	n.a.
2		26.113	973.615	97.50	n.a.
**Total:**			**998.612**	**100.00**	

Supplementary figure 163. HPLC chromatogram for compound **3d**

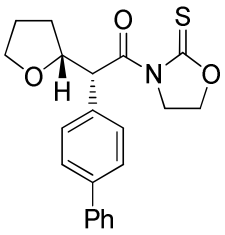

3d'

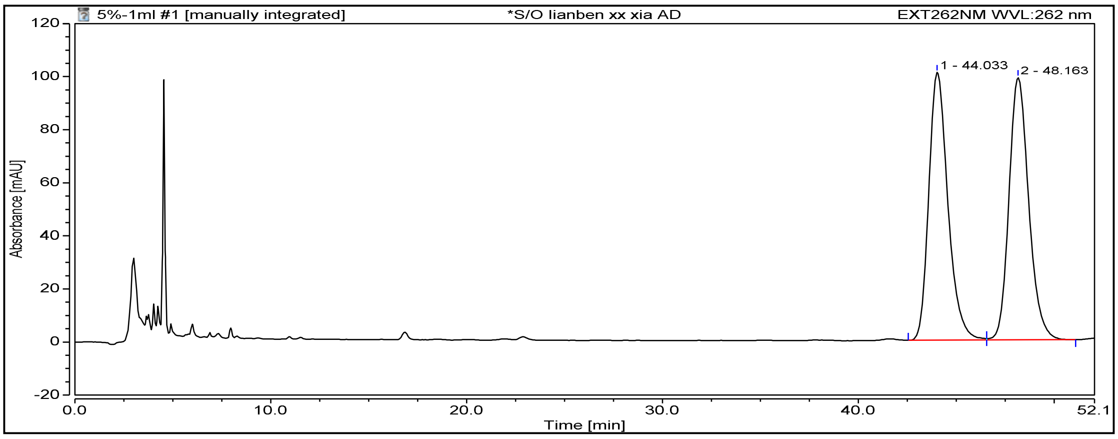

Integration Results					
No.	Peak Name	Retention Time<br>min	Area<br>mAU*min	Relative Area<br>%	Amount<br>n.a.
1		44.033	114.387	49.77	n.a.
2		48.163	115.459	50.23	n.a.
**Total:**			**229.846**	**100.00**	

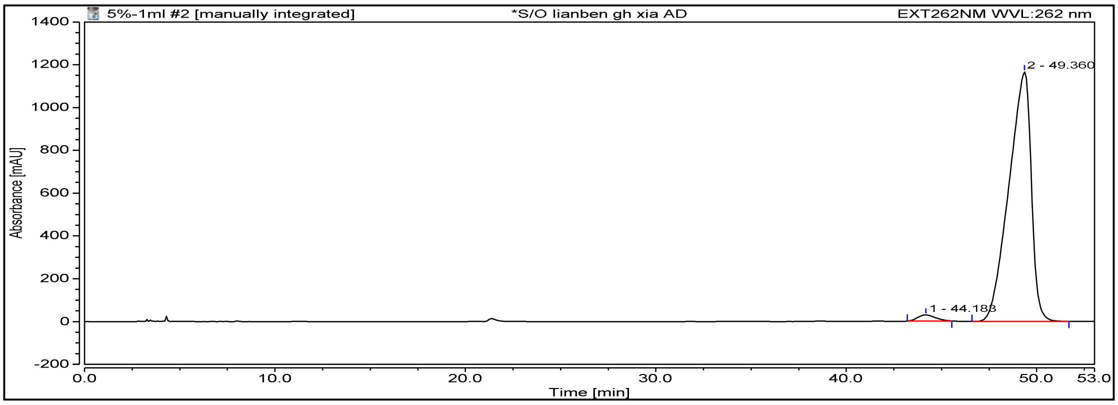

Integration Results					
No.	Peak Name	Retention Time<br>min	Area<br>mAU*min	Relative Area<br>%	Amount<br>n.a.
1		44.183	30.808	1.91	n.a.
2		49.360	1582.659	98.09	n.a.
**Total:**			**1613.467**	**100.00**	

Supplementary figure 164. HPLC chromatogram for compound **3d'**

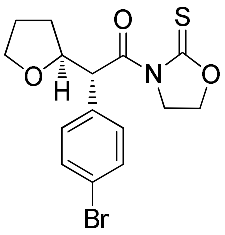

3e

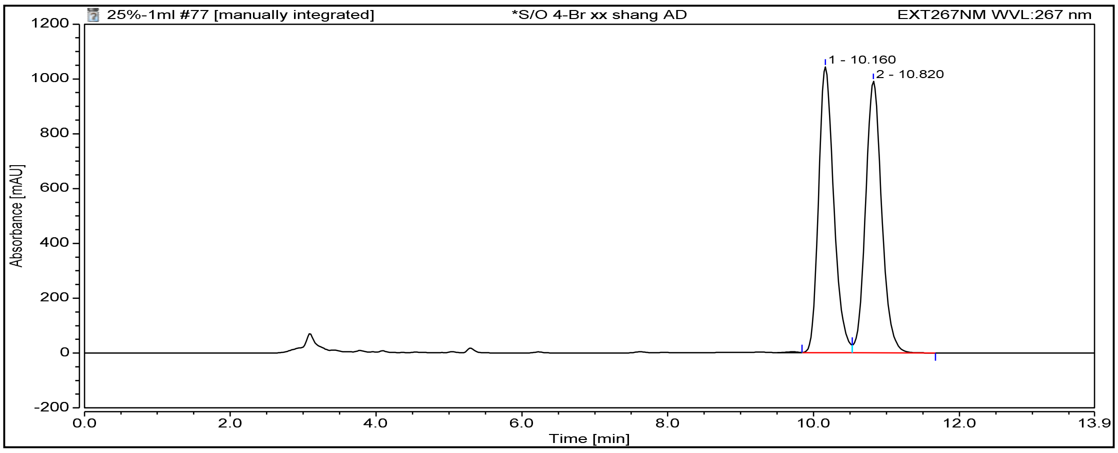

Integration Results

No.	Peak Name	Retention Time<br>min	Area<br>mAU*min	Relative Area<br>%	Amount<br>n.a.
1		10.160	252.392	49.76	n.a.
2		10.820	254.874	50.24	n.a.
**Total:**			**507.266**	**100.00**	

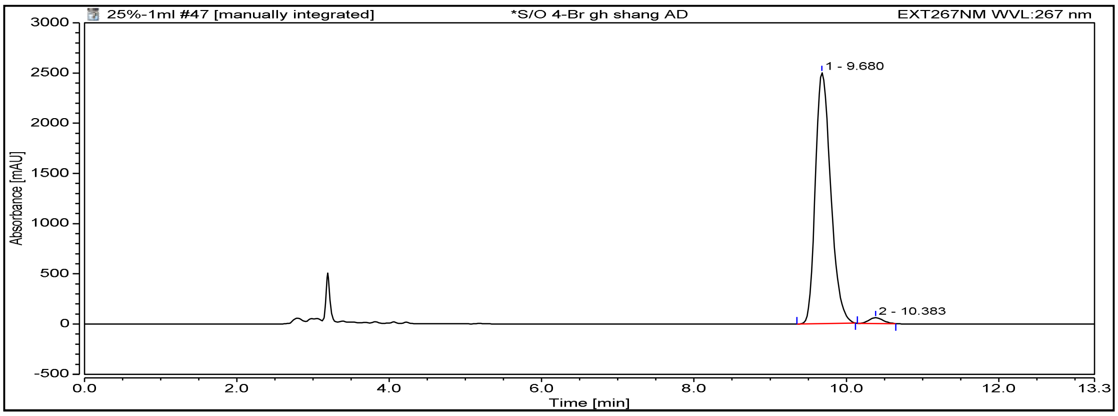

Integration Results

No.	Peak Name	Retention Time<br>min	Area<br>mAU*min	Relative Area<br>%	Amount<br>n.a.
1		9.680	583.615	97.98	n.a.
2		10.383	12.038	2.02	n.a.
**Total:**			**595.654**	**100.00**	

Supplementary figure 165. HPLC chromatogram for compound **3e**

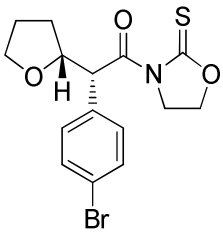

3e'

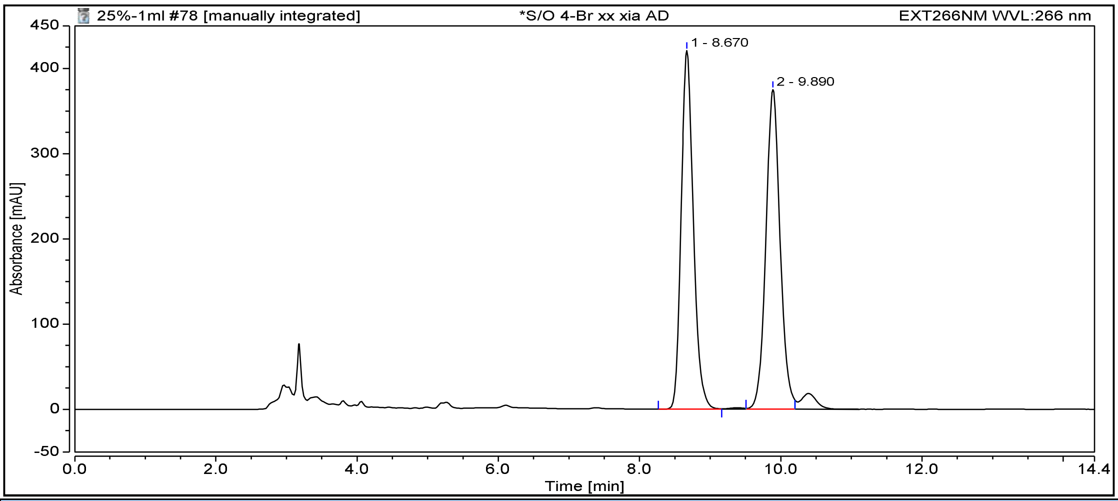

Integration Results					
No.	Peak Name	Retention Time<br>min	Area<br>mAU*min	Relative Area<br>%	Amount<br>n.a.
1		8.670	83.711	48.88	n.a.
2		9.890	87.560	51.12	n.a.
**Total:**			**171.271**	**100.00**	

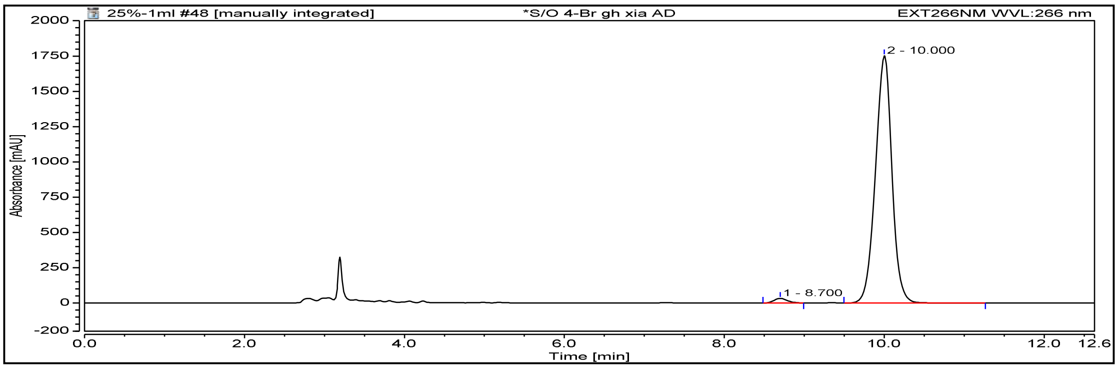

Integration Results					
No.	Peak Name	Retention Time<br>min	Area<br>mAU*min	Relative Area<br>%	Amount<br>n.a.
1		8.700	6.335	1.49	n.a.
2		10.000	417.849	98.51	n.a.
**Total:**			**424.184**	**100.00**	

Supplementary figure 166. HPLC chromatogram for compound **3e'**

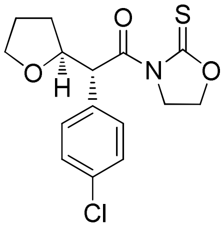

3f

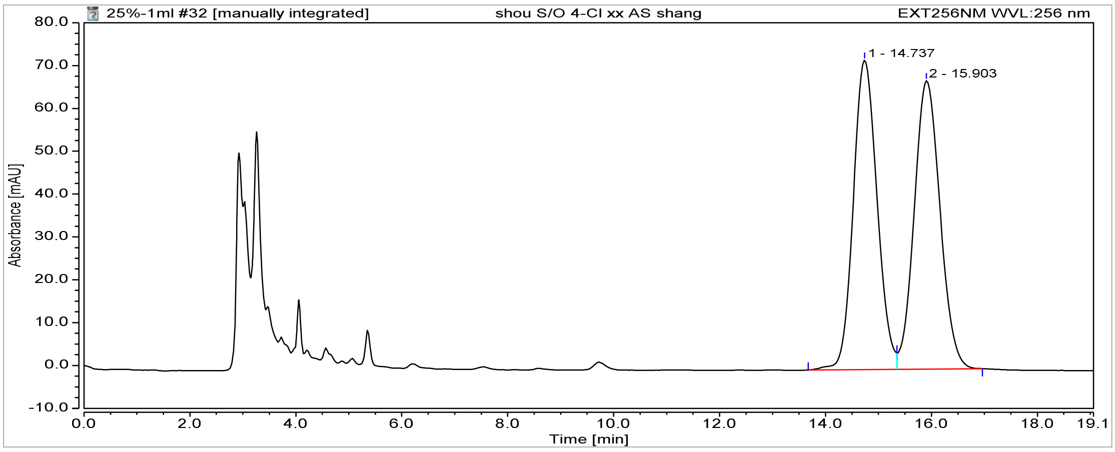

Integration Results					
No.	Peak Name	Retention Time<br>min	Area<br>mAU*min	Relative Area<br>%	Amount<br>n.a.
1		14.737	37.601	49.76	n.a.
2		15.903	37.966	50.24	n.a.
**Total:**			**75.567**	**100.00**	

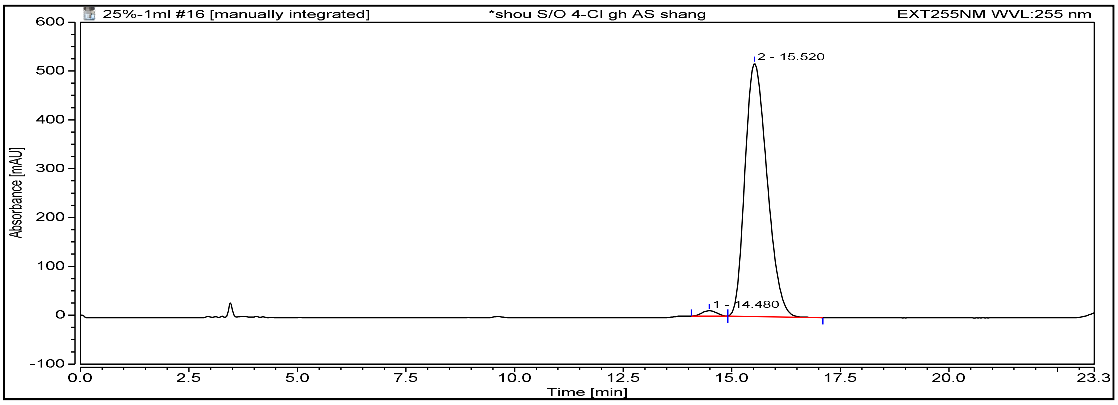

Integration Results					
No.	Peak Name	Retention Time<br>min	Area<br>mAU*min	Relative Area<br>%	Amount<br>n.a.
1		14.480	4.344	1.40	n.a.
2		15.520	305.385	98.60	n.a.
**Total:**			**309.729**	**100.00**	

Supplementary figure 167. HPLC chromatogram for compound **3f**

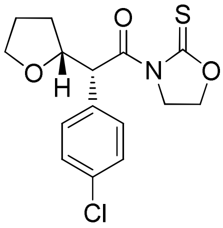

3f’

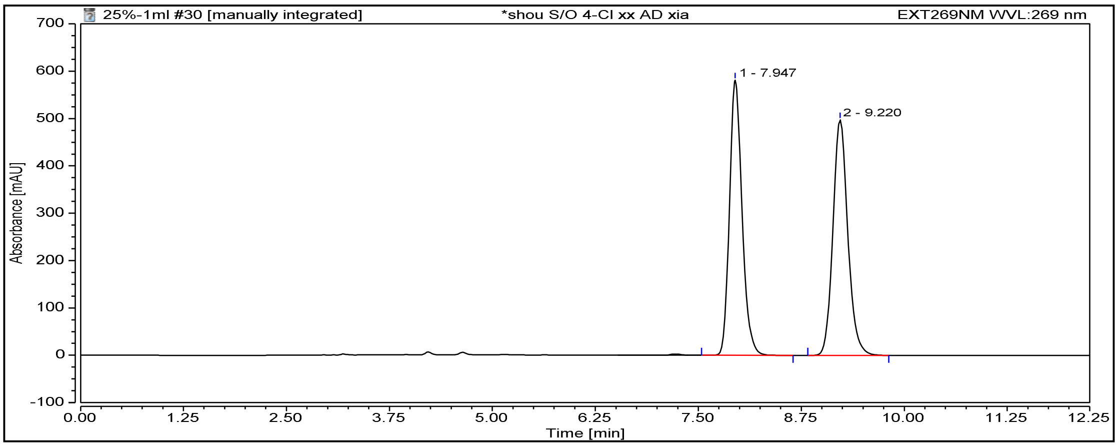

**Integration Results**					
No.	Peak Name	Retention Time<br>min	Area<br>mAU*min	Relative Area<br>%	Amount<br>n.a.
1		7.947	102.842	49.66	n.a.
2		9.220	104.243	50.34	n.a.
**Total:**			**207.085**	**100.00**	

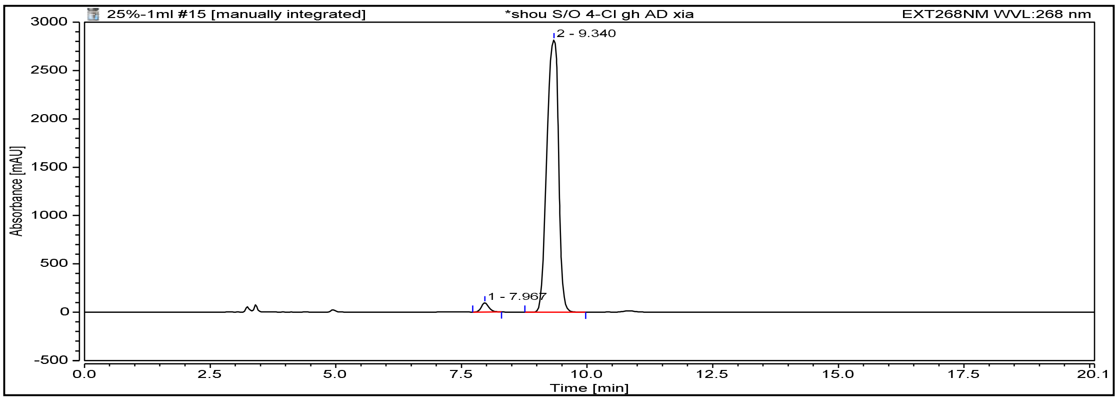

**Integration Results**					
No.	Peak Name	Retention Time<br>min	Area<br>mAU*min	Relative Area<br>%	Amount<br>n.a.
1		7.967	16.604	2.14	n.a.
2		9.340	760.072	97.86	n.a.
**Total:**			**776.675**	**100.00**	

Supplementary figure 168. HPLC chromatogram for compound **3f’**

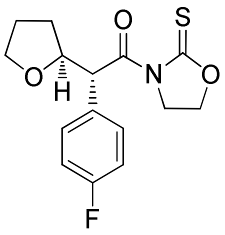

3g

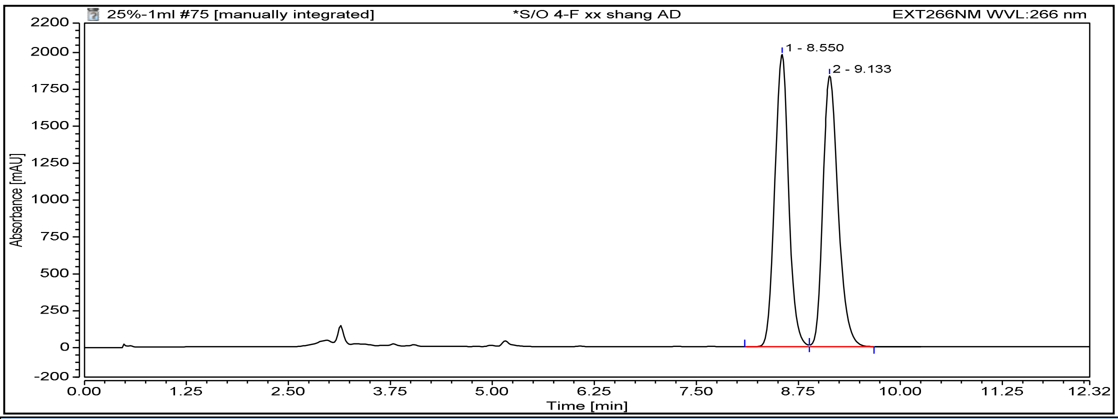

Integration Results					
No.	Peak Name	Retention Time<br>min	Area<br>mAU*min	Relative Area<br>%	Amount<br>n.a.
1		8.550	392.301	49.74	n.a.
2		9.133	396.323	50.26	n.a.
**Total:**			**788.625**	**100.00**	

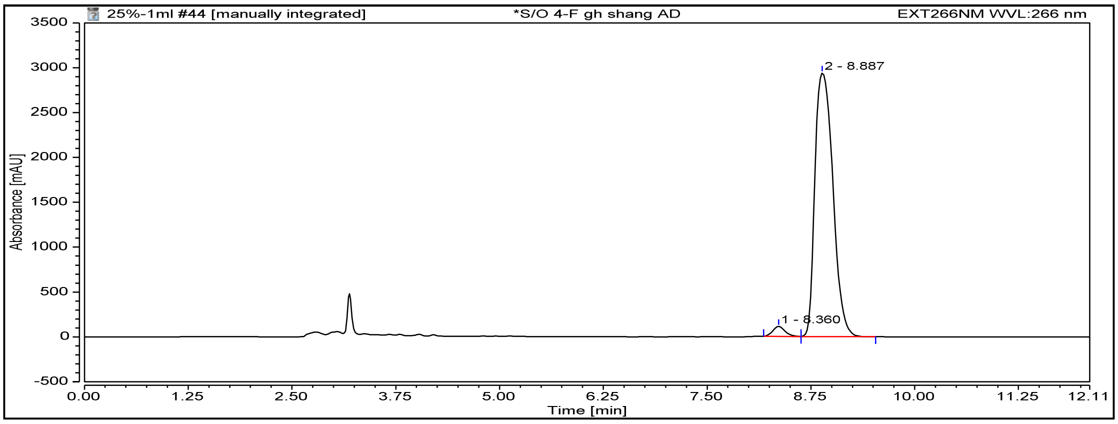

Integration Results					
No.	Peak Name	Retention Time<br>min	Area<br>mAU*min	Relative Area<br>%	Amount<br>n.a.
1		8.360	18.887	2.50	n.a.
2		8.887	736.382	97.50	n.a.
**Total:**			**755.269**	**100.00**	

Supplementary figure 169. HPLC chromatogram for compound **3g**

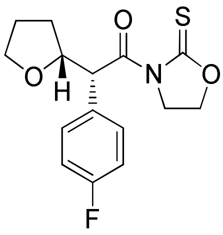

3g'

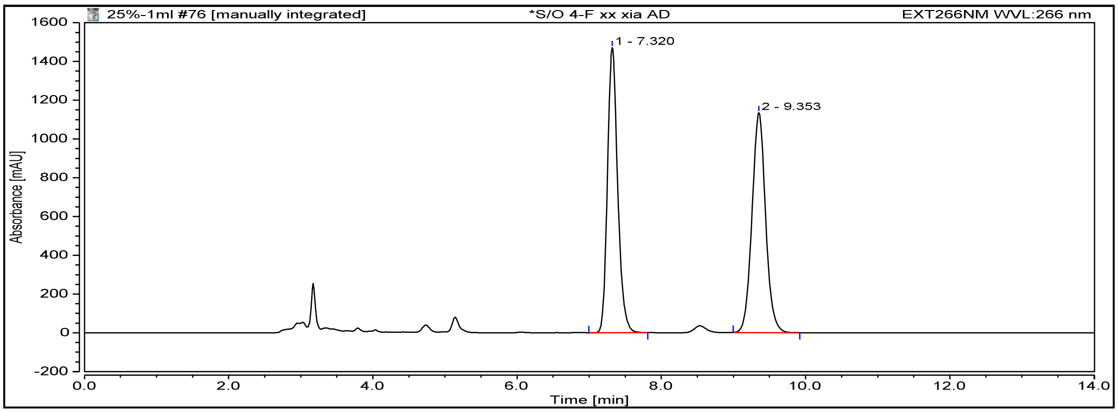

Integration Results					
No.	Peak Name	Retention Time<br>min	Area<br>mAU*min	Relative Area<br>%	Amount<br>n.a.
1		7.320	240.461	49.20	n.a.
2		9.353	248.327	50.80	n.a.
**Total:**			**488.789**	**100.00**	

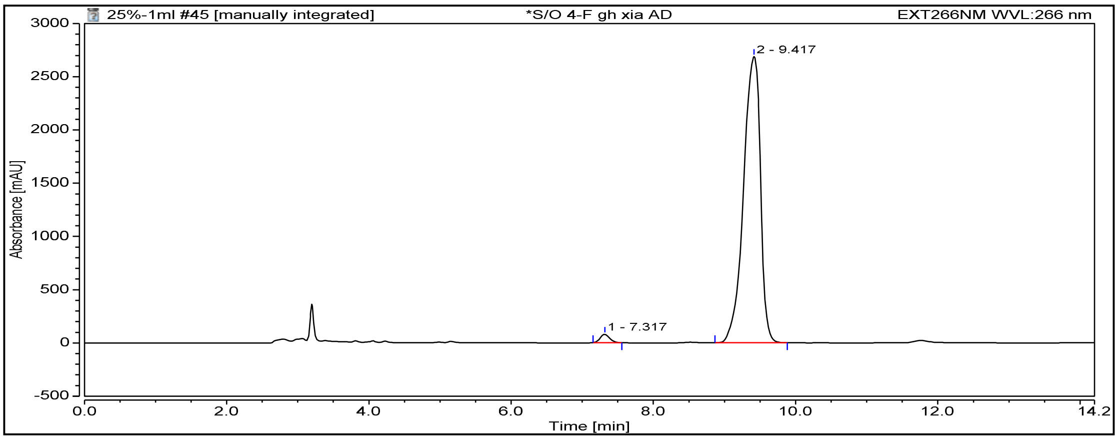

Integration Results					
No.	Peak Name	Retention Time<br>min	Area<br>mAU*min	Relative Area<br>%	Amount<br>n.a.
1		7.317	12.156	1.61	n.a.
2		9.417	741.931	98.39	n.a.
**Total:**			**754.087**	**100.00**	

Supplementary figure 170. HPLC chromatogram for compound **3g'**

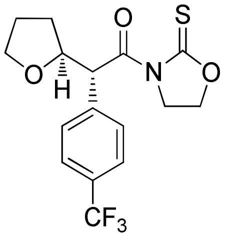

3h

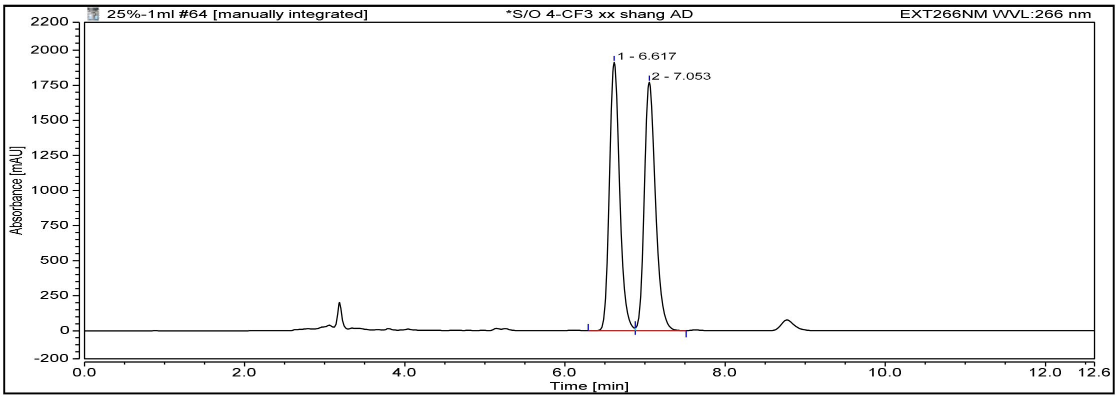

Integration Results					
No.	Peak Name	Retention Time<br>min	Area<br>mAU*min	Relative Area<br>%	Amount<br>n.a.
1		6.617	284.352	49.78	n.a.
2		7.053	286.832	50.22	n.a.
**Total:**			**571.184**	**100.00**	

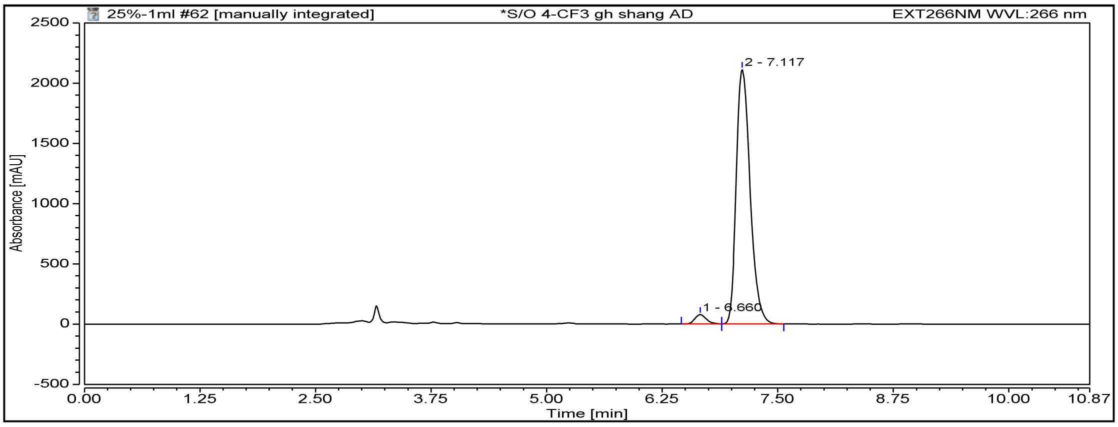

Integration Results					
No.	Peak Name	Retention Time<br>min	Area<br>mAU*min	Relative Area<br>%	Amount<br>n.a.
1		6.660	11.083	2.92	n.a.
2		7.117	368.188	97.08	n.a.
**Total:**			**379.271**	**100.00**	

Supplementary figure 171. HPLC chromatogram for compound **3h**

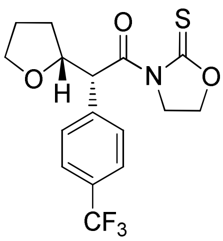

3h’

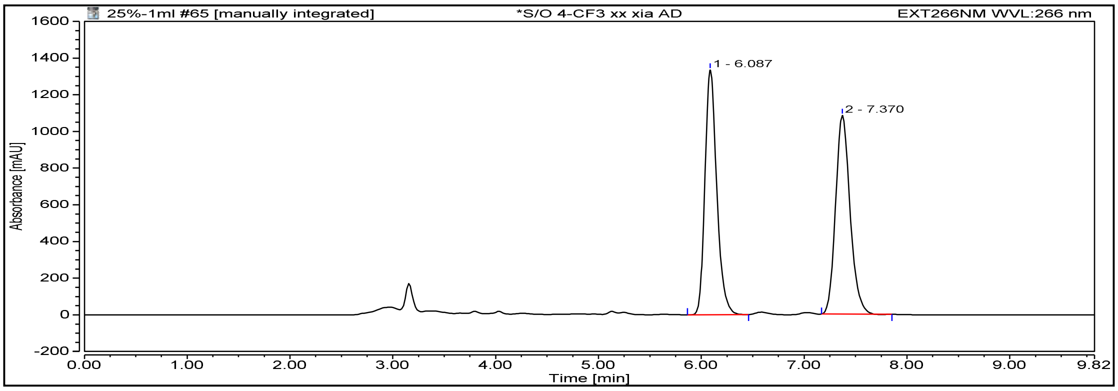

Integration Results					
No.	Peak Name	Retention Time min	Area mAU*min	Relative Area %	Amount n.a.
1		6.087	177.993	50.12	n.a.
2		7.370	177.123	49.88	n.a.
**Total:**			**355.115**	**100.00**	

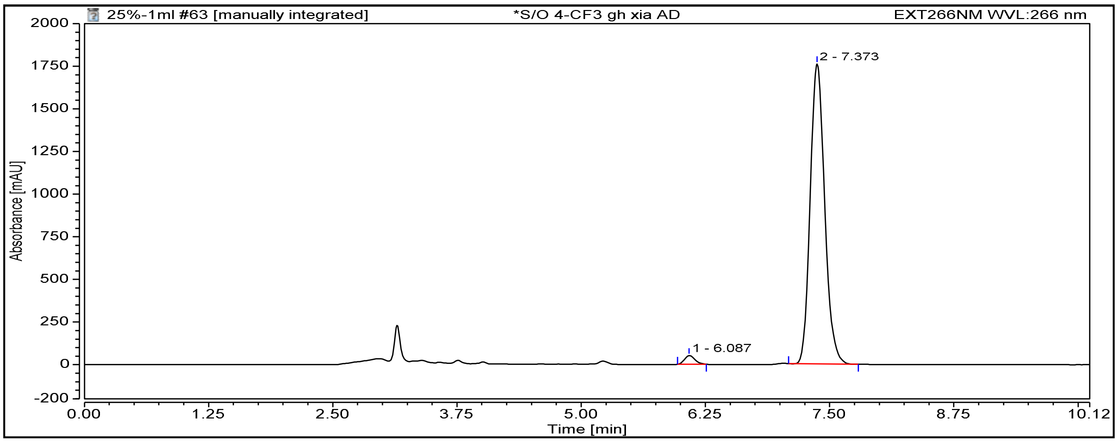

Integration Results					
No.	Peak Name	Retention Time min	Area mAU*min	Relative Area %	Amount n.a.
1		6.087	6.215	2.05	n.a.
2		7.373	296.695	97.95	n.a.
**Total:**			**302.910**	**100.00**	

Supplementary figure 172. HPLC chromatogram for compound **3h’**

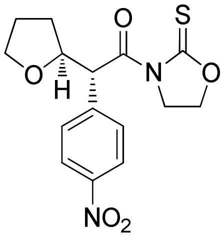

3i

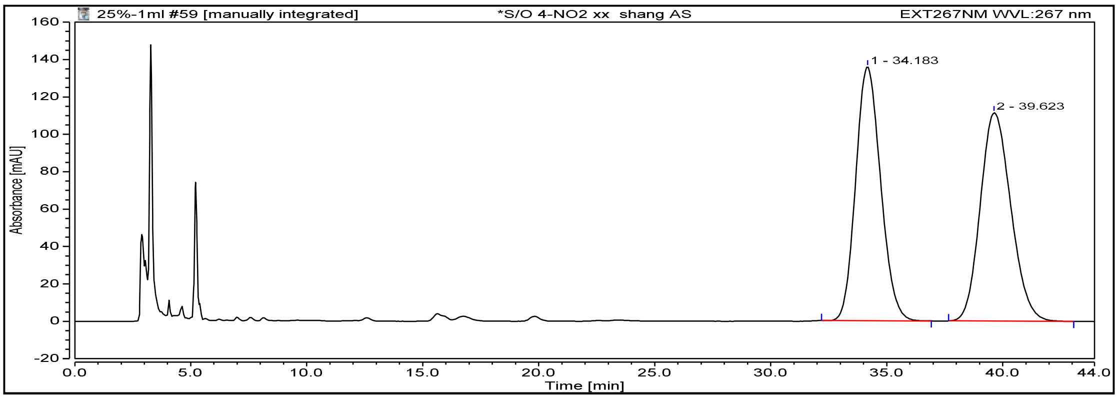

Integration Results					
No.	Peak Name	Retention Time min	Area mAU*min	Relative Area %	Amount n.a.
1		34.183	169.243	49.79	n.a.
2		39.623	170.664	50.21	n.a.
**Total:**			**339.907**	**100.00**	

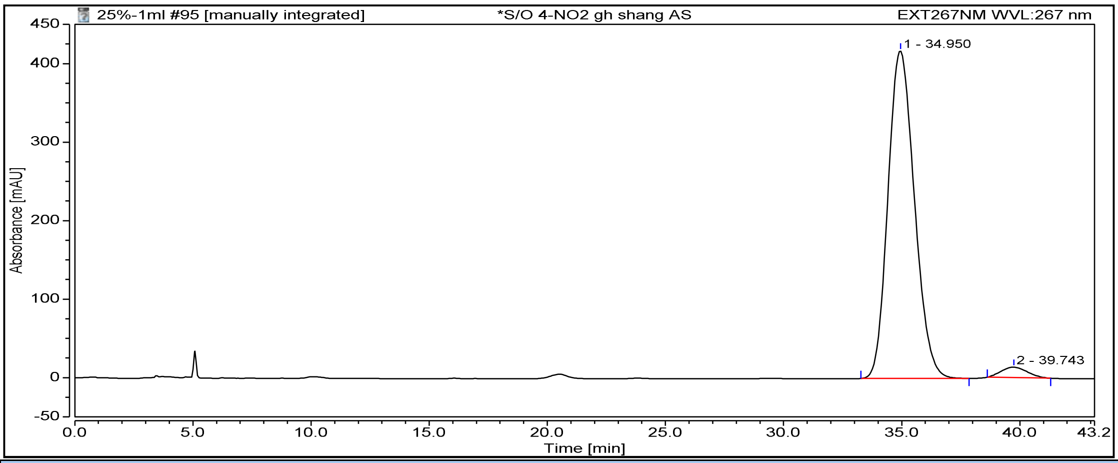

Integration Results					
No.	Peak Name	Retention Time min	Area mAU*min	Relative Area %	Amount n.a.
1		34.950	540.912	96.96	n.a.
2		39.743	16.988	3.04	n.a.
**Total:**			**557.899**	**100.00**	

Supplementary figure 173. HPLC chromatogram for compound **3i**

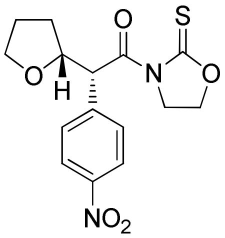

3i'

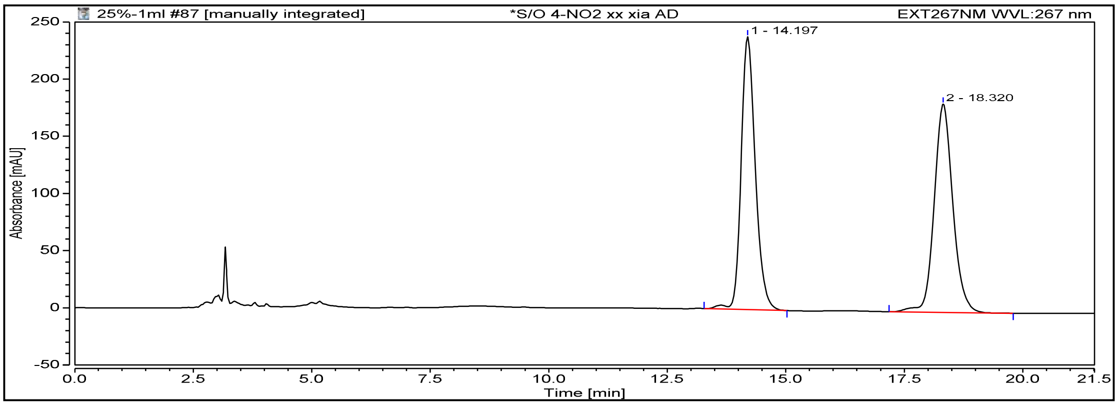

Integration Results					
No.	Peak Name	Retention Time<br>min	Area<br>mAU*min	Relative Area<br>%	Amount<br>n.a.
1		14.197	82.713	49.75	n.a.
2		18.320	83.542	50.25	n.a.
**Total:**			**166.255**	**100.00**	

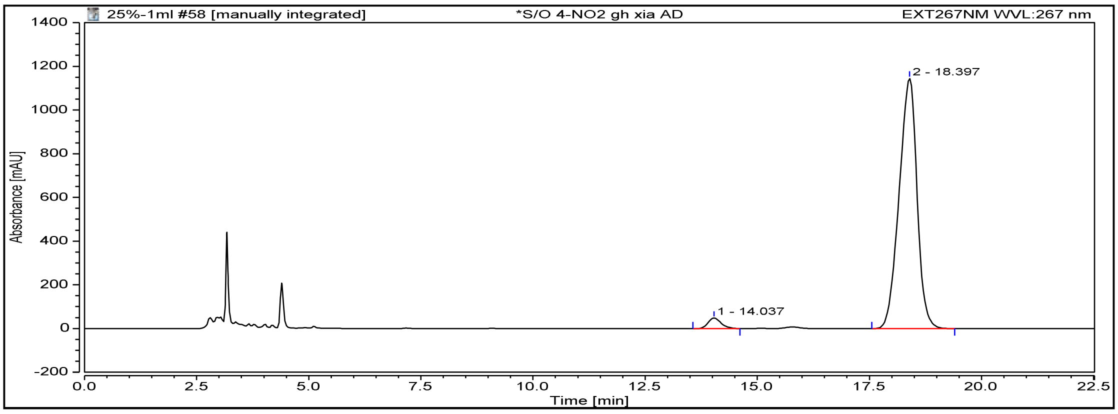

Integration Results					
No.	Peak Name	Retention Time<br>min	Area<br>mAU*min	Relative Area<br>%	Amount<br>n.a.
1		14.037	16.381	3.00	n.a.
2		18.397	530.028	97.00	n.a.
**Total:**			**546.409**	**100.00**	

Supplementary figure 174. HPLC chromatogram for compound **3i'**

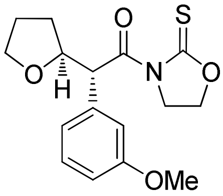

3j

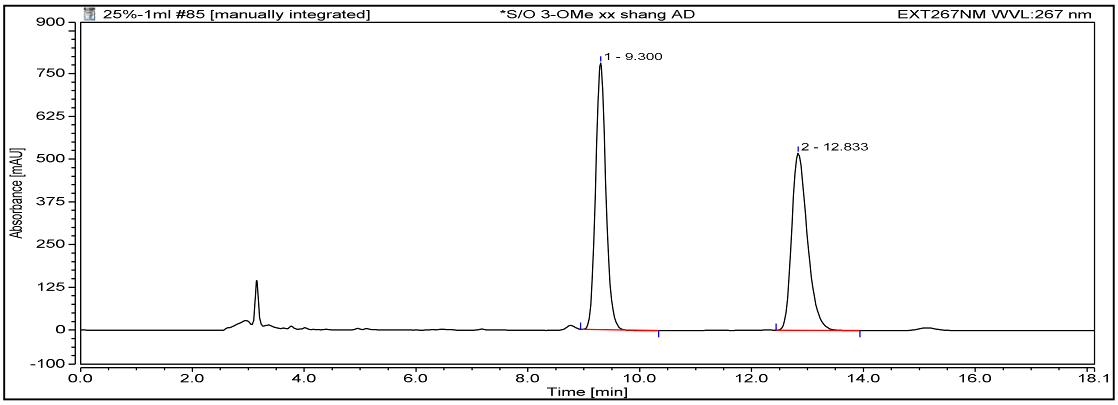

Integration Results					
No.	Peak Name	Retention Time<br>min	Area<br>mAU*min	Relative Area<br>%	Amount<br>n.a.
1		9.300	165.904	49.63	n.a.
2		12.833	168.364	50.37	n.a.
**Total:**			**334.269**	**100.00**	

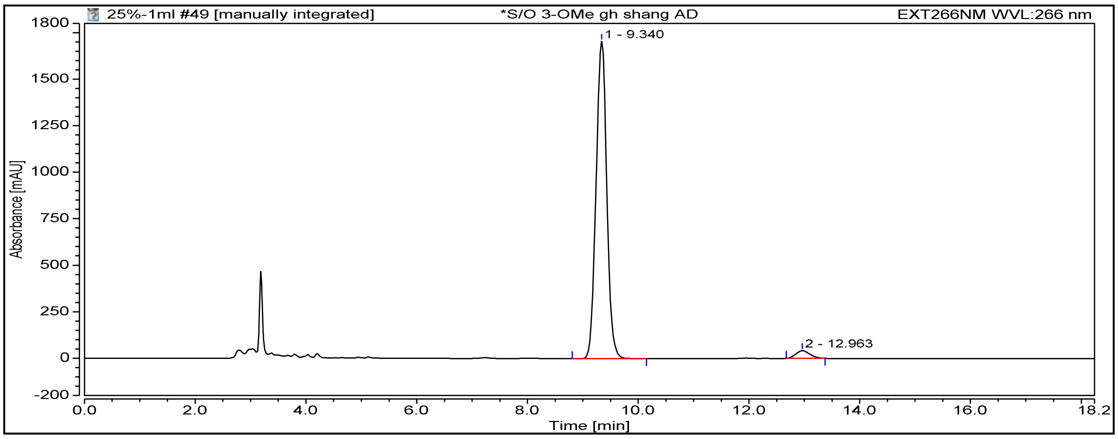

Integration Results					
No.	Peak Name	Retention Time<br>min	Area<br>mAU*min	Relative Area<br>%	Amount<br>n.a.
1		9.340	381.601	96.91	n.a.
2		12.963	12.149	3.09	n.a.
**Total:**			**393.751**	**100.00**	

Supplementary figure 175. HPLC chromatogram for compound **3j**

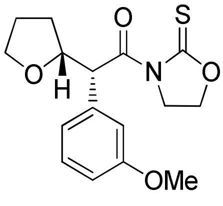

3j'

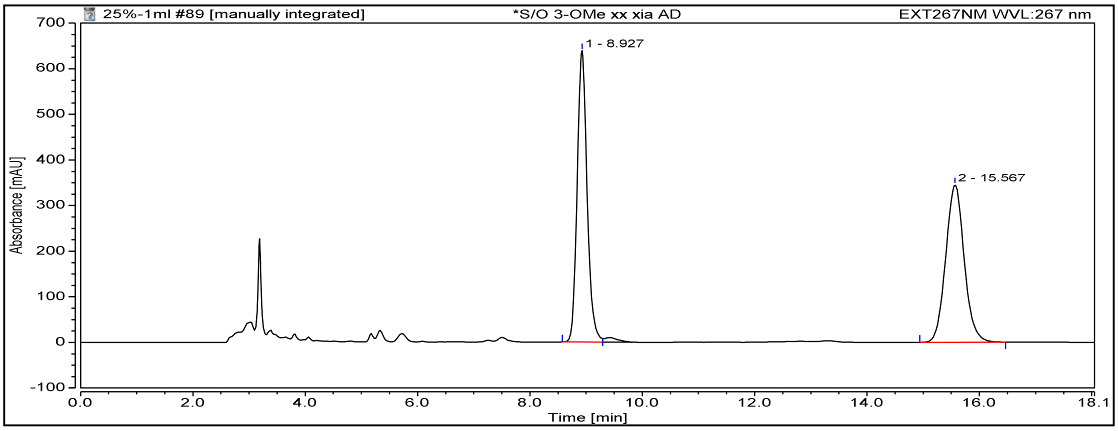

Integration Results					
No.	Peak Name	Retention Time<br>min	Area<br>mAU*min	Relative Area<br>%	Amount<br>n.a.
1		8.927	131.019	49.98	n.a.
2		15.567	131.129	50.02	n.a.
**Total:**			**262.148**	**100.00**	

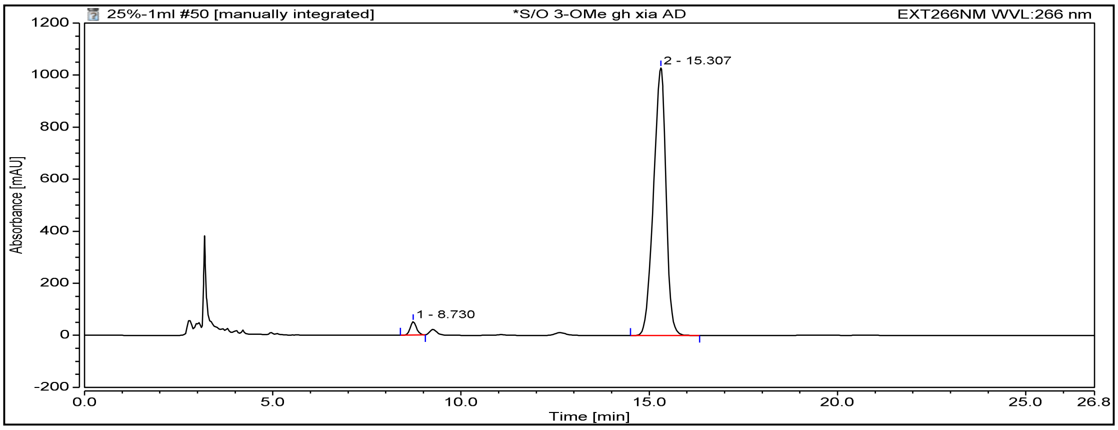

Integration Results					
No.	Peak Name	Retention Time<br>min	Area<br>mAU*min	Relative Area<br>%	Amount<br>n.a.
1		8.730	9.758	2.41	n.a.
2		15.307	394.867	97.59	n.a.
**Total:**			**404.625**	**100.00**	

Supplementary figure 176. HPLC chromatogram for compound **3j'**

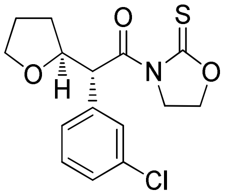

3k

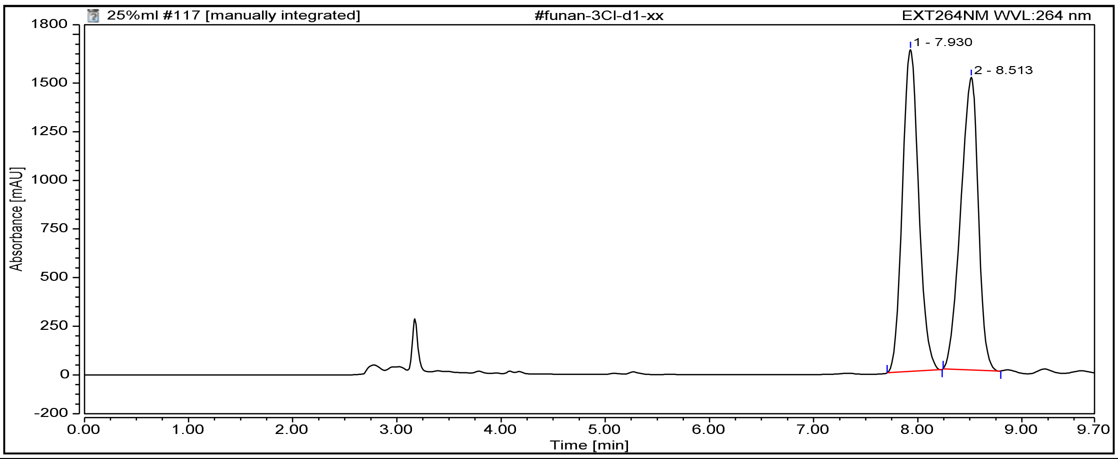

Integration Results					
No.	Peak Name	Retention Time min	Area mAU*min	Relative Area %	Amount n.a.
1		7.930	300.221	50.55	n.a.
2		8.513	293.651	49.45	n.a.
**Total:**			**593.872**	**100.00**	

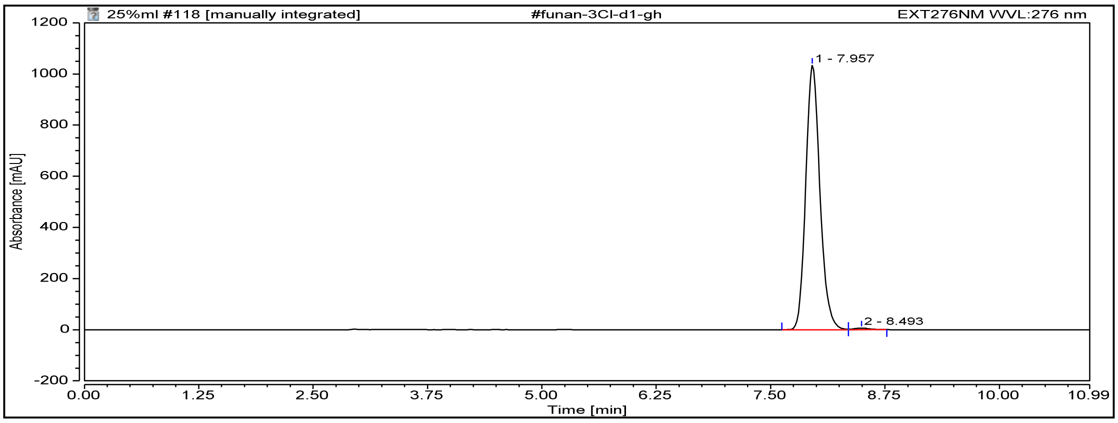

Integration Results					
No.	Peak Name	Retention Time min	Area mAU*min	Relative Area %	Amount n.a.
1		7.957	186.175	99.36	n.a.
2		8.493	1.197	0.64	n.a.
**Total:**			**187.372**	**100.00**	

Supplementary figure 177. HPLC chromatogram for compound **3k**

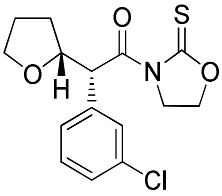

3k’

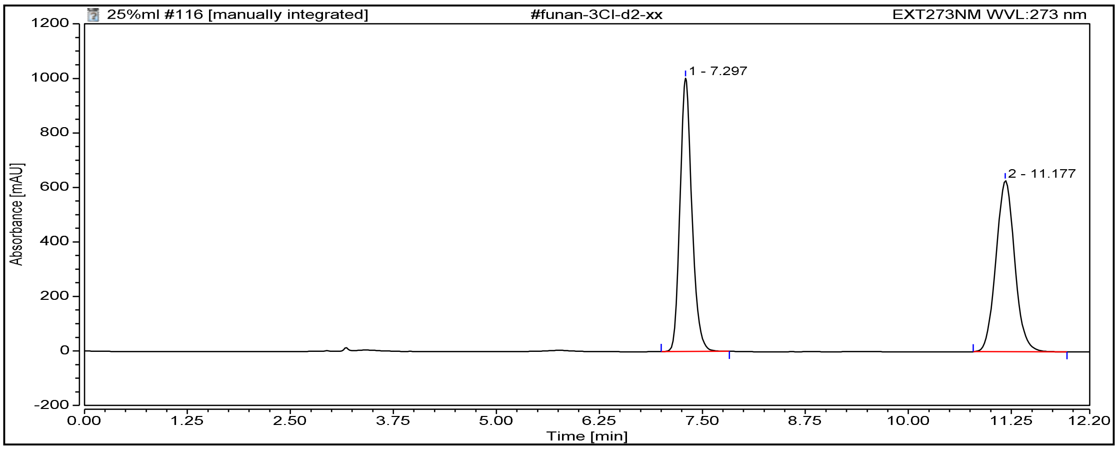

Integration Results					
No.	Peak Name	Retention Time<br>min	Area<br>mAU*min	Relative Area<br>%	Amount<br>n.a.
1		7.297	167.838	50.34	n.a.
2		11.177	165.571	49.66	n.a.
**Total:**			**333.409**	**100.00**	

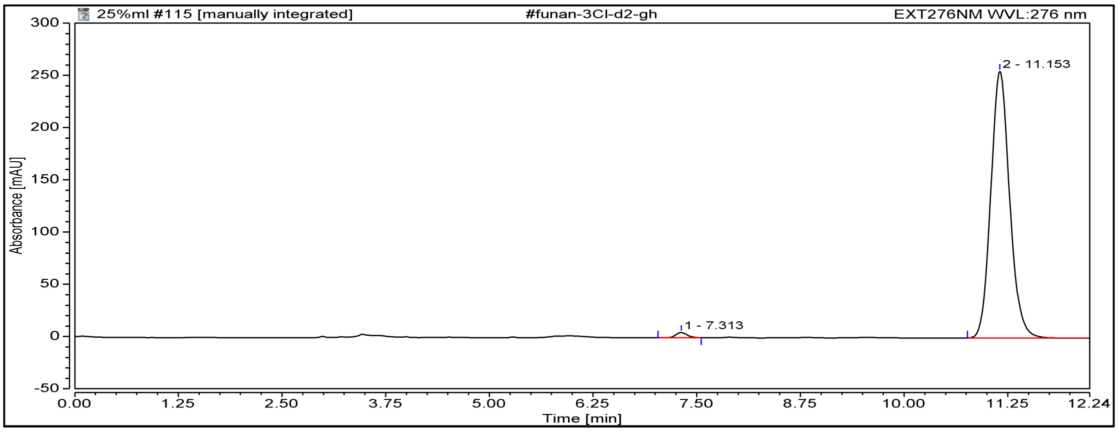

Integration Results					
No.	Peak Name	Retention Time<br>min	Area<br>mAU*min	Relative Area<br>%	Amount<br>n.a.
1		7.313	0.808	1.19	n.a.
2		11.153	67.179	98.81	n.a.
**Total:**			**67.987**	**100.00**	

Supplementary figure 178. HPLC chromatogram for compound **3k’**

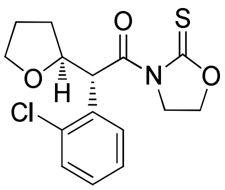

3l

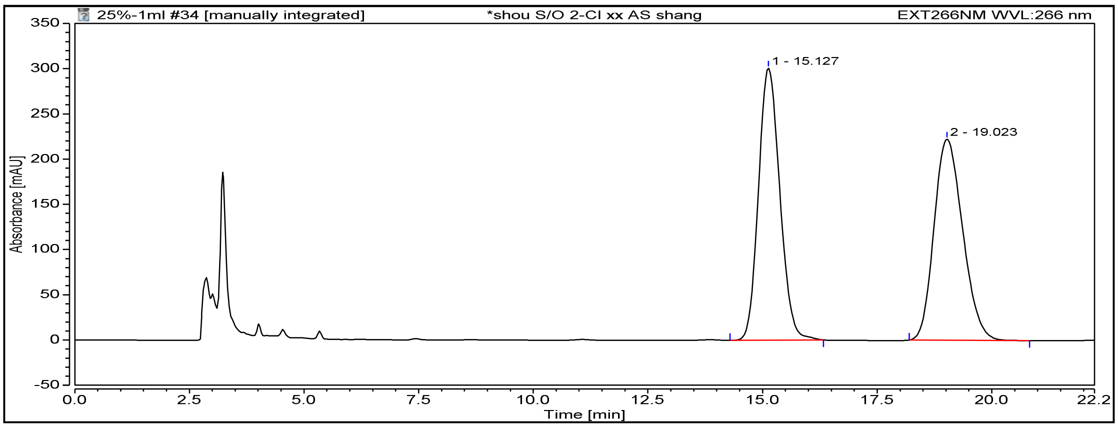

Integration Results					
No.	Peak Name	Retention Time min	Area mAU*min	Relative Area %	Amount n.a.
1		15.127	162.521	50.24	n.a.
2		19.023	160.967	49.76	n.a.
**Total:**			**323.489**	**100.00**	

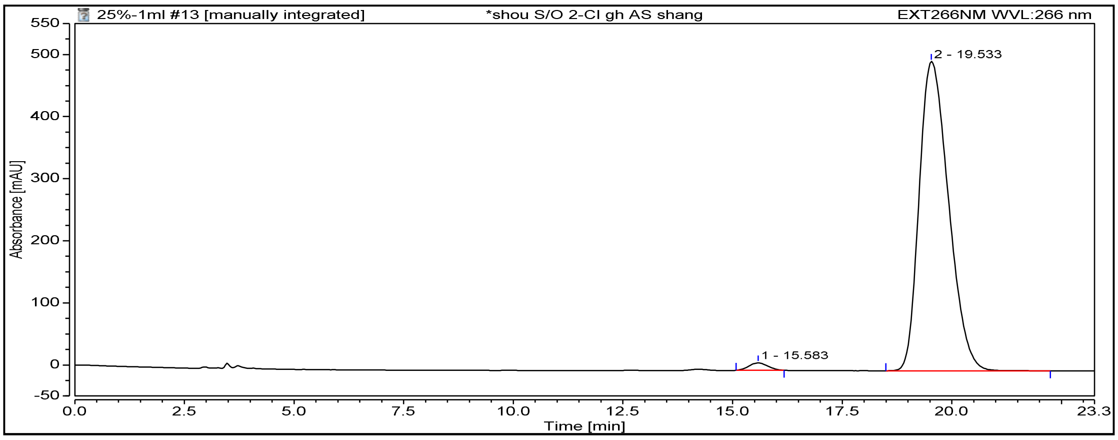

Integration Results					
No.	Peak Name	Retention Time min	Area mAU*min	Relative Area %	Amount n.a.
1		15.583	6.020	1.53	n.a.
2		19.533	387.062	98.47	n.a.
**Total:**			**393.081**	**100.00**	

Supplementary figure 179. HPLC chromatogram for compound **3l**

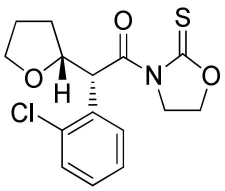

3l'

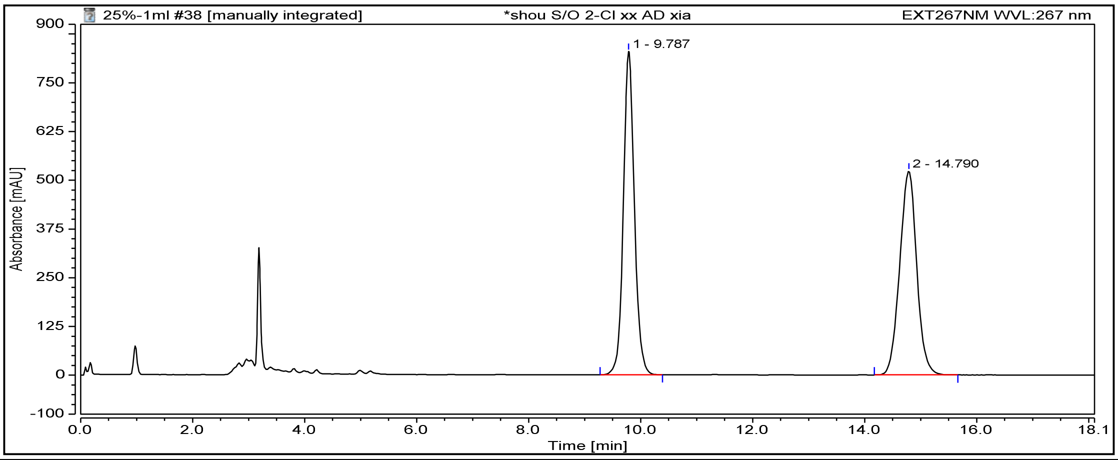

Integration Results					
No.	Peak Name	Retention Time<br>min	Area<br>mAU*min	Relative Area<br>%	Amount<br>n.a.
1		9.787	189.135	50.74	n.a.
2		14.790	183.630	49.26	n.a.
**Total:**			**372.765**	**100.00**	

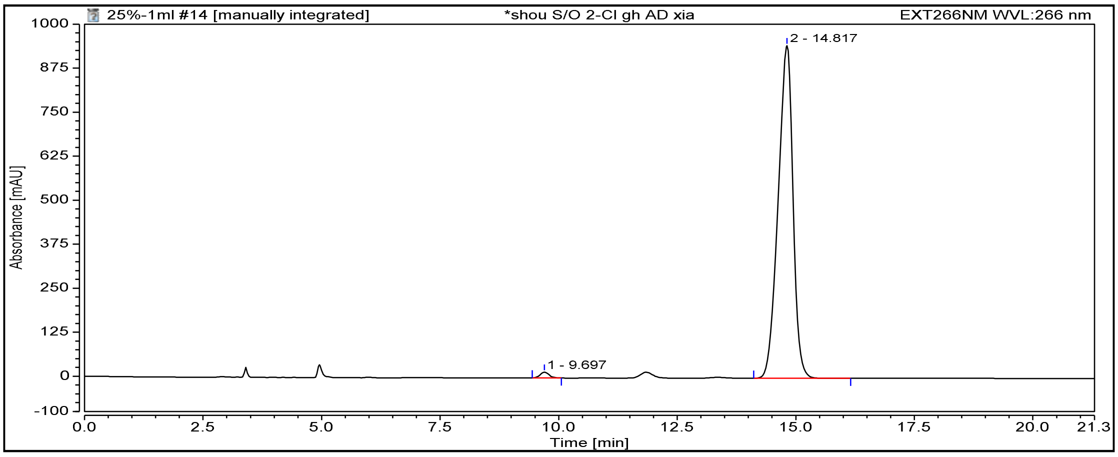

Integration Results					
No.	Peak Name	Retention Time<br>min	Area<br>mAU*min	Relative Area<br>%	Amount<br>n.a.
1		9.697	3.599	1.04	n.a.
2		14.817	344.017	98.96	n.a.
**Total:**			**347.616**	**100.00**	

Supplementary figure 180. HPLC chromatogram for compound **3l'**

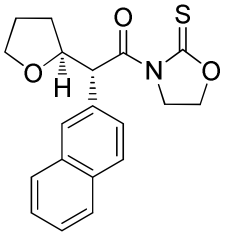

3m

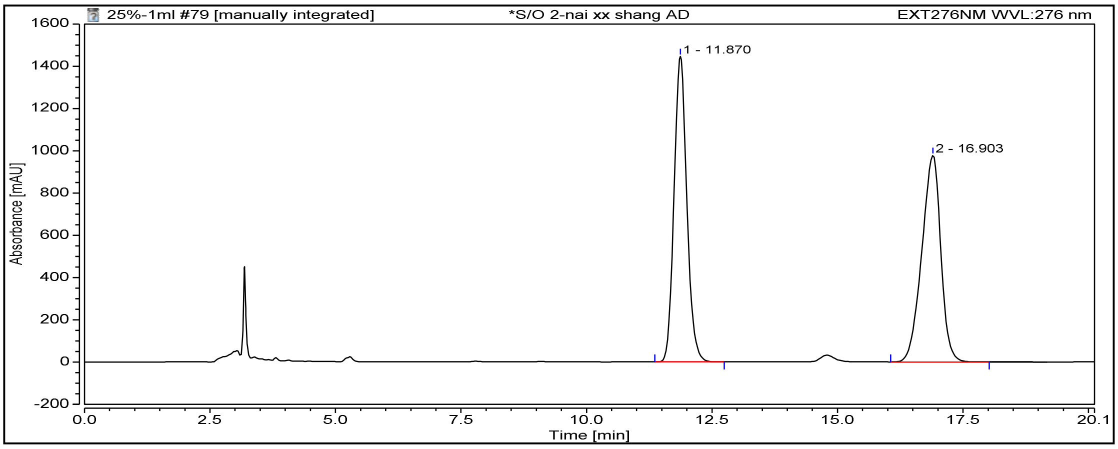

Integration Results					
No.	Peak Name	Retention Time<br>min	Area<br>mAU*min	Relative Area<br>%	Amount<br>n.a.
1		11.870	425.818	49.92	n.a.
2		16.903	427.131	50.08	n.a.
**Total:**			**852.950**	**100.00**	

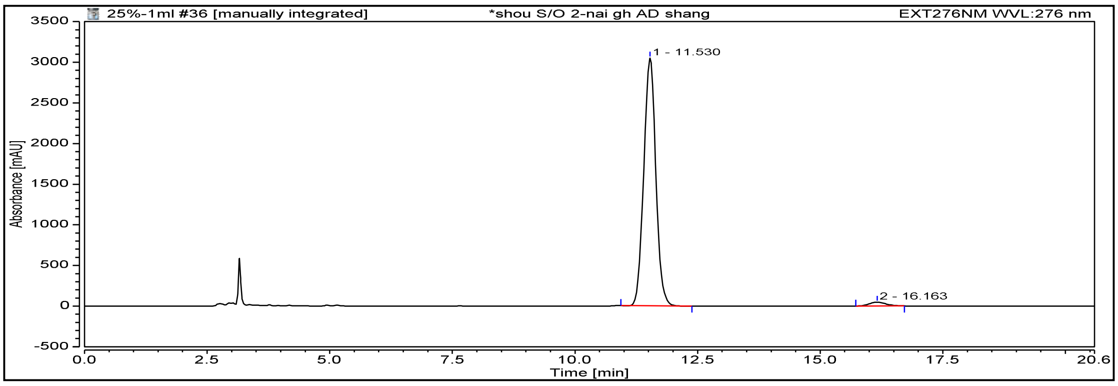

Integration Results					
No.	Peak Name	Retention Time<br>min	Area<br>mAU*min	Relative Area<br>%	Amount<br>n.a.
1		11.530	869.394	97.89	n.a.
2		16.163	18.753	2.11	n.a.
**Total:**			**888.148**	**100.00**	

Supplementary figure 181. HPLC chromatogram for compound **3m**

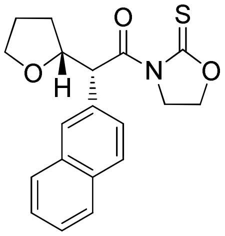

3m’

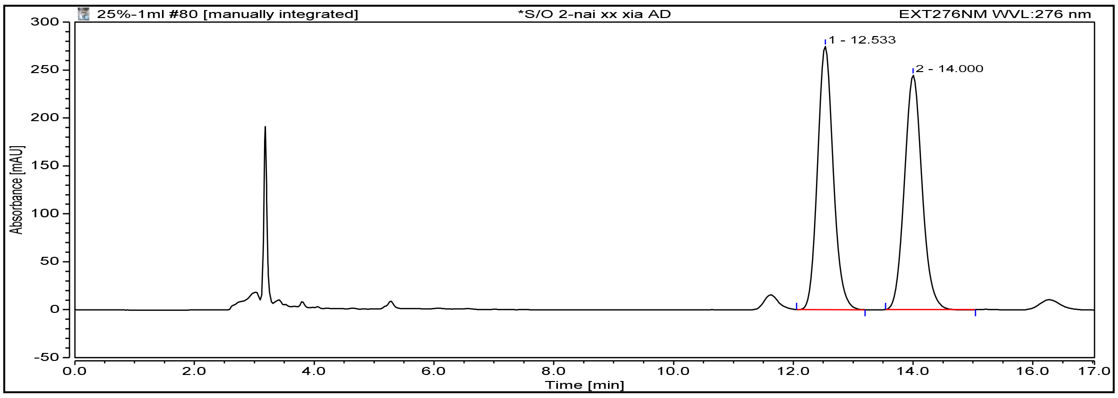

Integration Results					
No.	Peak Name	Retention Time min	Area mAU*min	Relative Area %	Amount n.a.
1		12.533	83.632	50.03	n.a.
2		14.000	83.521	49.97	n.a.
**Total:**			**167.154**	**100.00**	

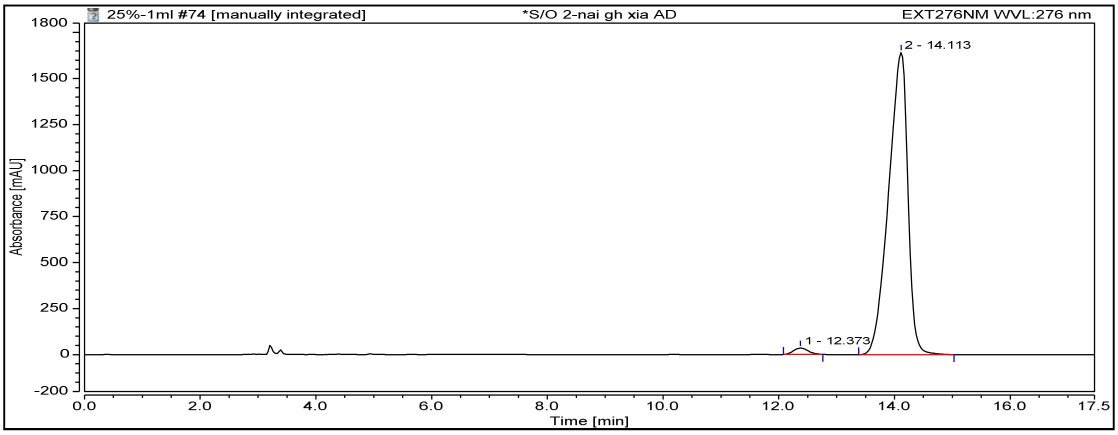

Integration Results					
No.	Peak Name	Retention Time min	Area mAU*min	Relative Area %	Amount n.a.
1		12.373	10.304	1.60	n.a.
2		14.113	632.357	98.40	n.a.
**Total:**			**642.661**	**100.00**	

Supplementary figure 182. HPLC chromatogram for compound **3m’**

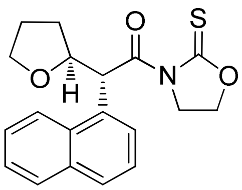

3n

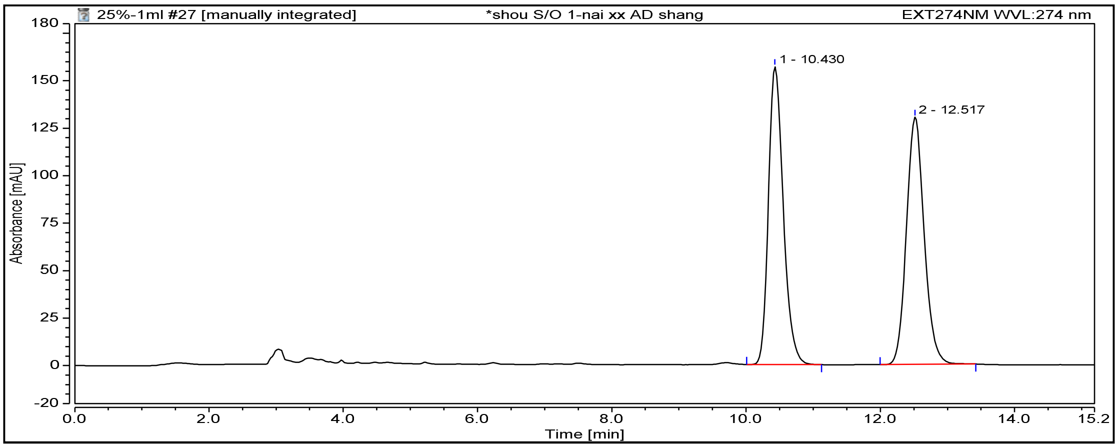

Integration Results					
No.	Peak Name	Retention Time<br>min	Area<br>mAU*min	Relative Area<br>%	Amount<br>n.a.
1		10.430	40.105	50.11	n.a.
2		12.517	39.929	49.89	n.a.
**Total:**			**80.034**	**100.00**	

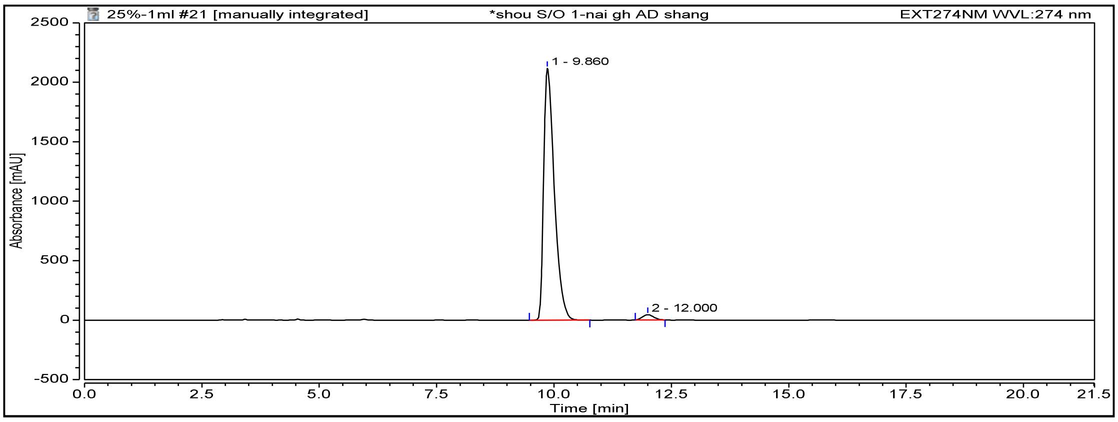

Integration Results					
No.	Peak Name	Retention Time<br>min	Area<br>mAU*min	Relative Area<br>%	Amount<br>n.a.
1		9.860	558.282	97.88	n.a.
2		12.000	12.064	2.12	n.a.
**Total:**			**570.345**	**100.00**	

Supplementary figure 183. HPLC chromatogram for compound **3n**

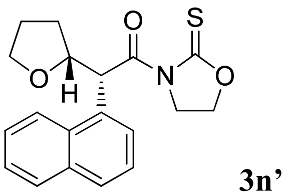

3n'

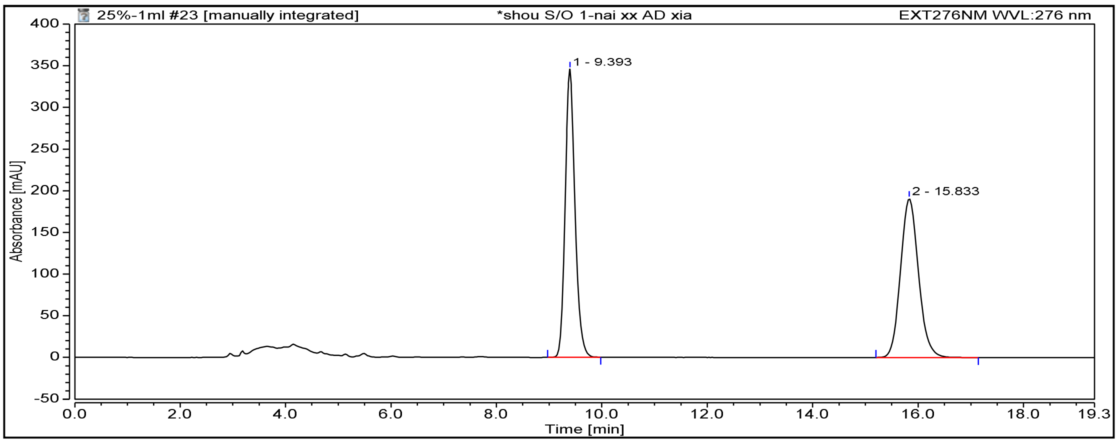

Integration Results					
No.	Peak Name	Retention Time min	Area mAU*min	Relative Area %	Amount n.a.
1		9.393	75.224	50.10	n.a.
2		15.833	74.921	49.90	n.a.
**Total:**			**150.145**	**100.00**	

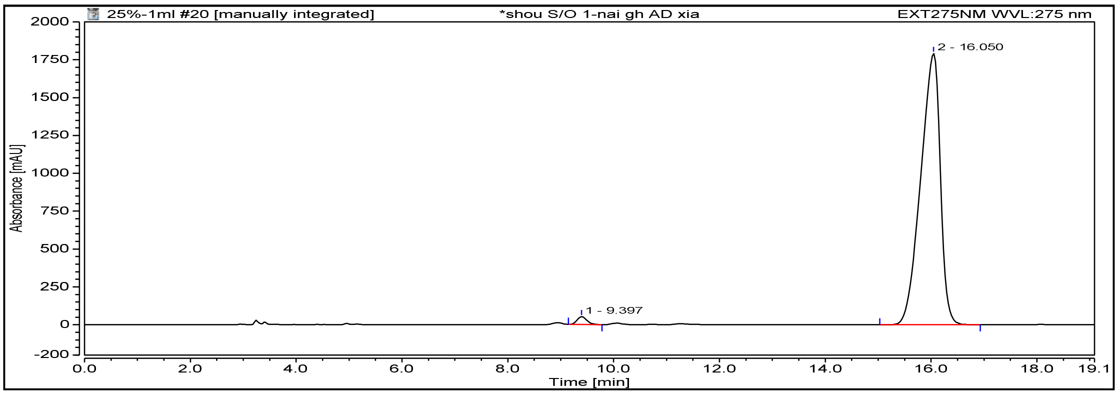

Integration Results					
No.	Peak Name	Retention Time min	Area mAU*min	Relative Area %	Amount n.a.
1		9.397	11.046	1.42	n.a.
2		16.050	765.062	98.58	n.a.
**Total:**			**776.108**	**100.00**	

Supplementary figure 184. HPLC chromatogram for compound **3n'**

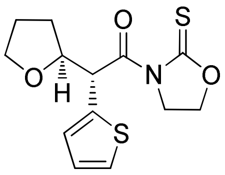

3o

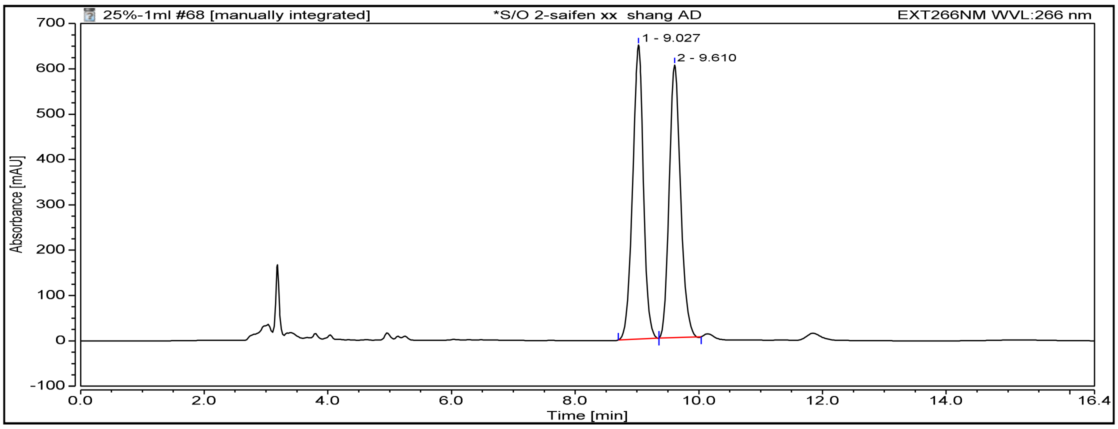

**Integration Results**					
No.	Peak Name	Retention Time<br>min	Area<br>mAU*min	Relative Area<br>%	Amount<br>n.a.
1		9.027	130.569	50.61	n.a.
2		9.610	127.418	49.39	n.a.
**Total:**			**257.987**	**100.00**	

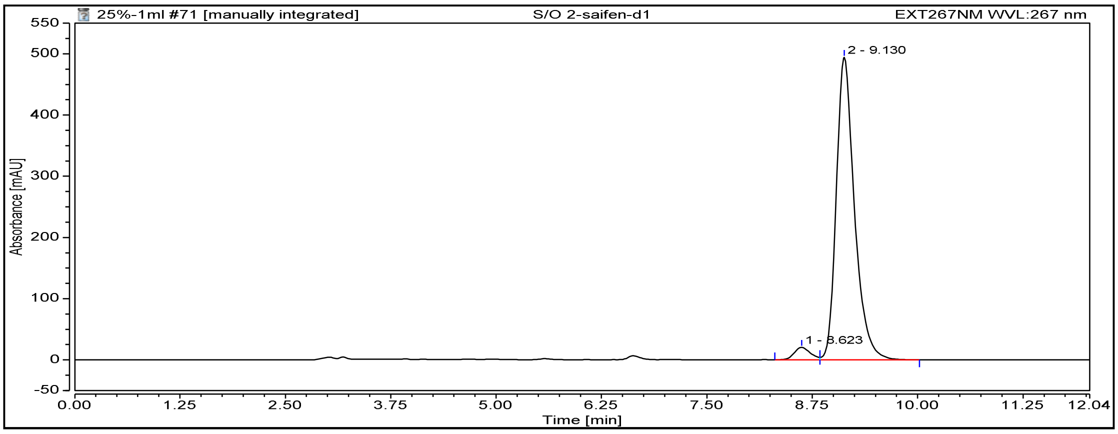

**Integration Results**					
No.	Peak Name	Retention Time<br>min	Area<br>mAU*min	Relative Area<br>%	Amount<br>n.a.
1		8.623	4.499	3.51	n.a.
2		9.130	123.757	96.49	n.a.
**Total:**			**128.256**	**100.00**	

Supplementary figure 185. HPLC chromatogram for compound **3o**

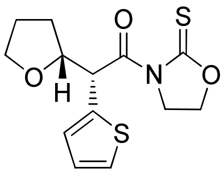

3o’

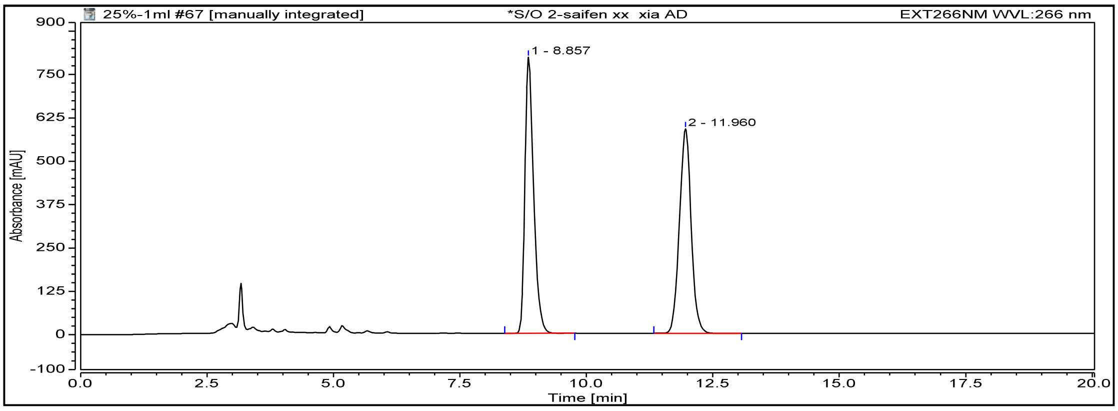

Integration Results					
No.	Peak Name	Retention Time<br>min	Area<br>mAU*min	Relative Area<br>%	Amount<br>n.a.
1		8.857	158.657	50.00	n.a.
2		11.960	158.650	50.00	n.a.
**Total:**			**317.308**	**100.00**	

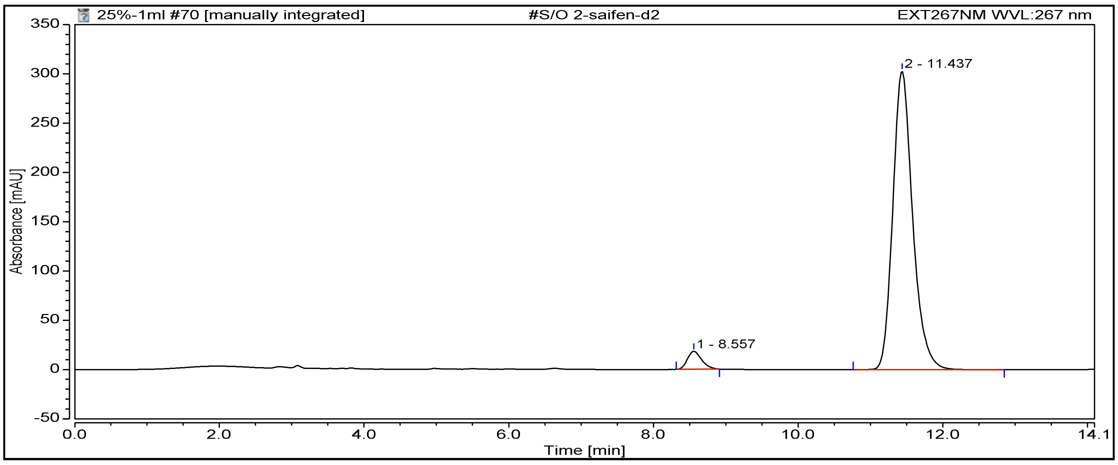

Integration Results					
No.	Peak Name	Retention Time<br>min	Area<br>mAU*min	Relative Area<br>%	Amount<br>n.a.
1		8.557	4.145	4.13	n.a.
2		11.437	96.253	95.87	n.a.
**Total:**			**100.398**	**100.00**	

Supplementary figure 186. HPLC chromatogram for compound **3o’**

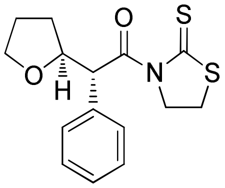

3p

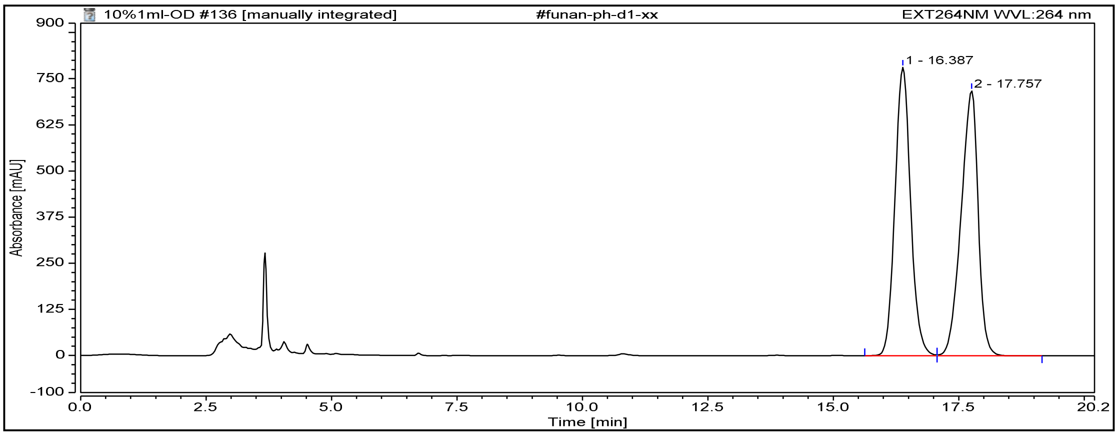

Integration Results					
No.	Peak Name	Retention Time<br>min	Area<br>mAU*min	Relative Area<br>%	Amount<br>n.a.
1		16.387	283.419	49.97	n.a.
2		17.757	283.803	50.03	n.a.
**Total:**			**567.222**	**100.00**	

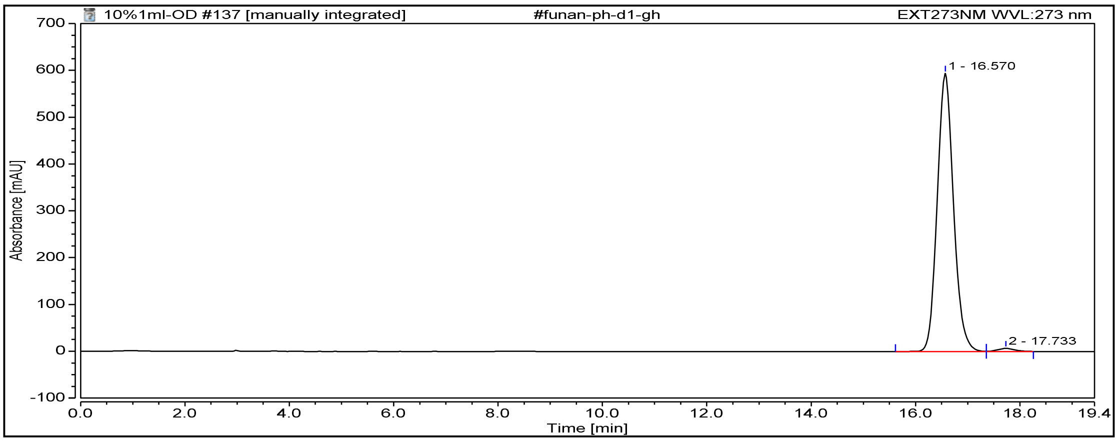

Integration Results					
No.	Peak Name	Retention Time<br>min	Area<br>mAU*min	Relative Area<br>%	Amount<br>n.a.
1		16.570	214.743	98.87	n.a.
2		17.733	2.455	1.13	n.a.
**Total:**			**217.198**	**100.00**	

Supplementary figure 187. HPLC chromatogram for compound **3p**

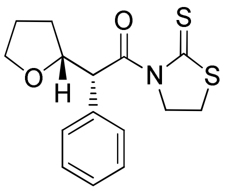

3p'

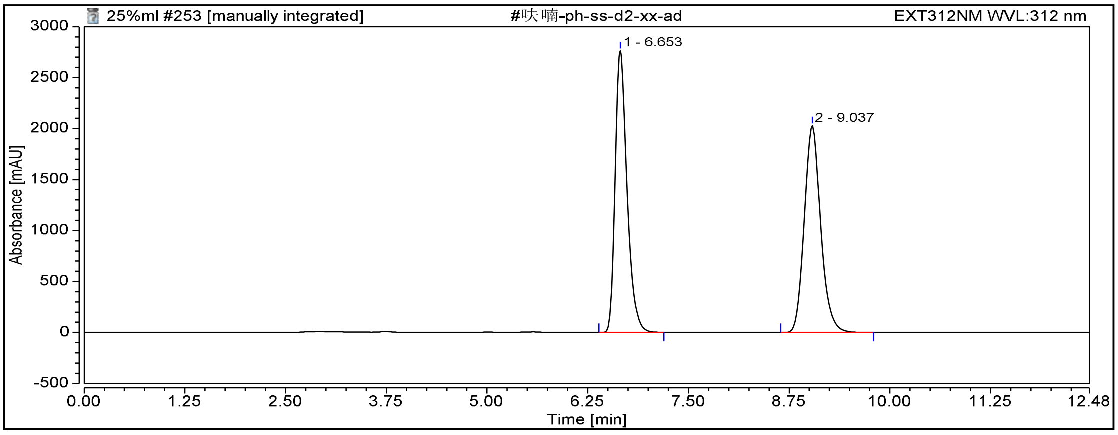

Integration Results					
No.	Peak Name	Retention Time<br>min	Area<br>mAU*min	Relative Area<br>%	Amount<br>n.a.
1		6.653	474.697	49.85	n.a.
2		9.037	477.548	50.15	n.a.
**Total:**			**952.245**	**100.00**	

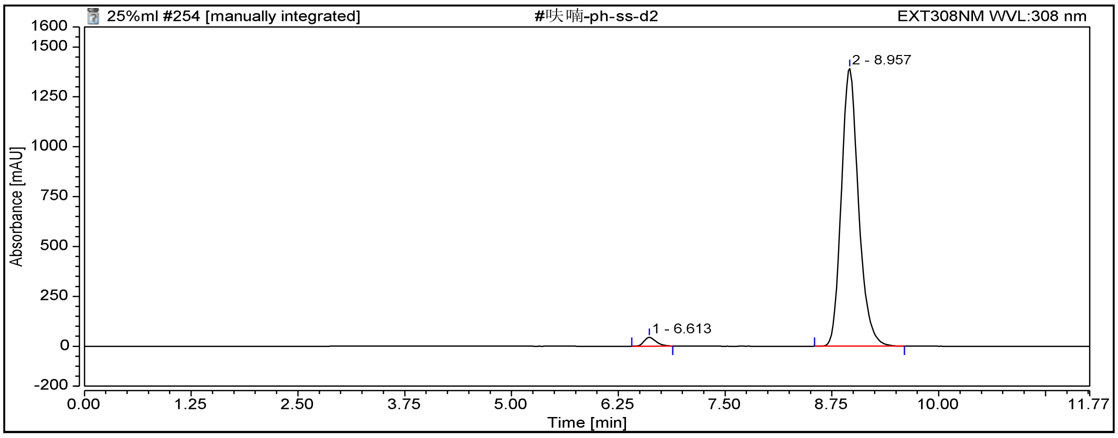

Integration Results					
No.	Peak Name	Retention Time<br>min	Area<br>mAU*min	Relative Area<br>%	Amount<br>n.a.
1		6.613	7.262	2.18	n.a.
2		8.957	325.887	97.82	n.a.
**Total:**			**333.149**	**100.00**	

Supplementary figure 188. HPLC chromatogram for compound **3p'**

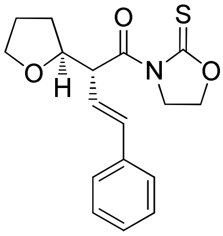

3q

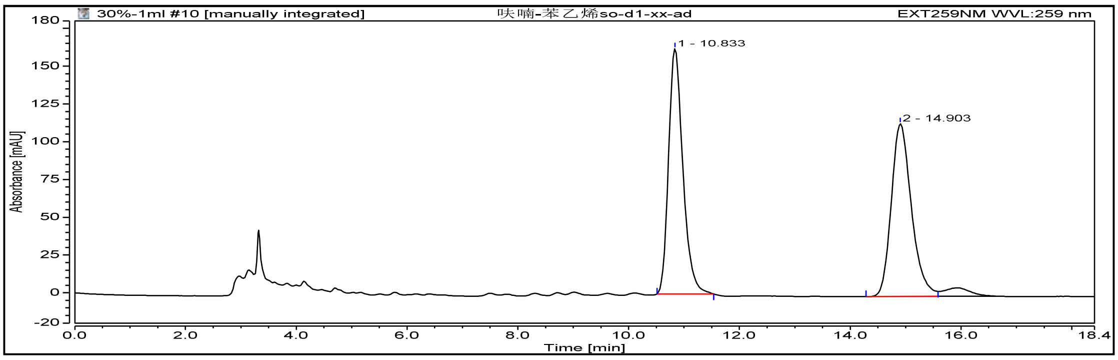

Integration Results					
No.	Peak Name	Retention Time<br>min	Area<br>mAU*min	Relative Area<br>%	Amount<br>n.a.
1		10.833	48.609	50.30	n.a.
2		14.903	48.033	49.70	n.a.
**Total:**			**96.643**	**100.00**	

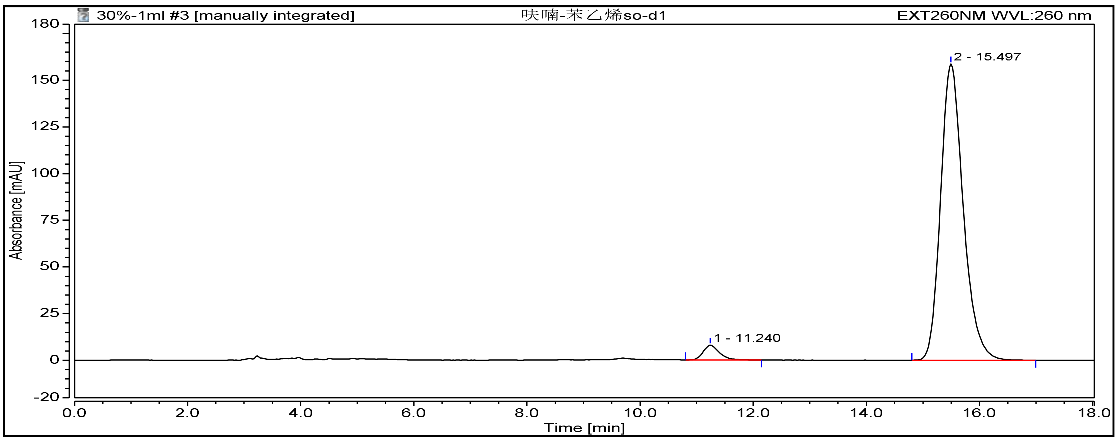

Integration Results					
No.	Peak Name	Retention Time<br>min	Area<br>mAU*min	Relative Area<br>%	Amount<br>n.a.
1		11.240	2.638	3.46	n.a.
2		15.497	73.701	96.54	n.a.
**Total:**			**76.340**	**100.00**	

Supplementary figure 189. HPLC chromatogram for compound **3q**

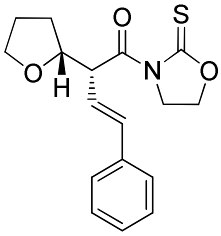

3q’

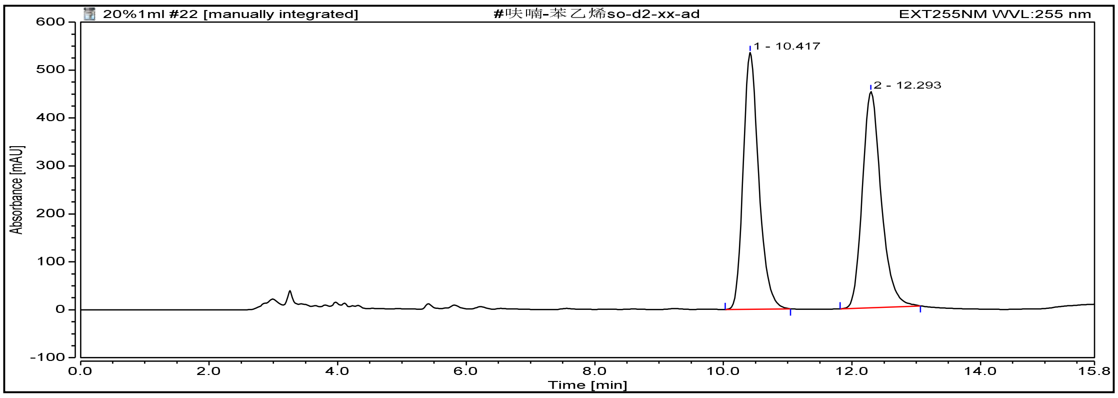

Integration Results					
No.	Peak Name	Retention Time<br>min	Area<br>mAU*min	Relative Area<br>%	Amount<br>n.a.
1		10.417	150.861	49.62	n.a.
2		12.293	153.152	50.38	n.a.
**Total:**			**304.013**	**100.00**	

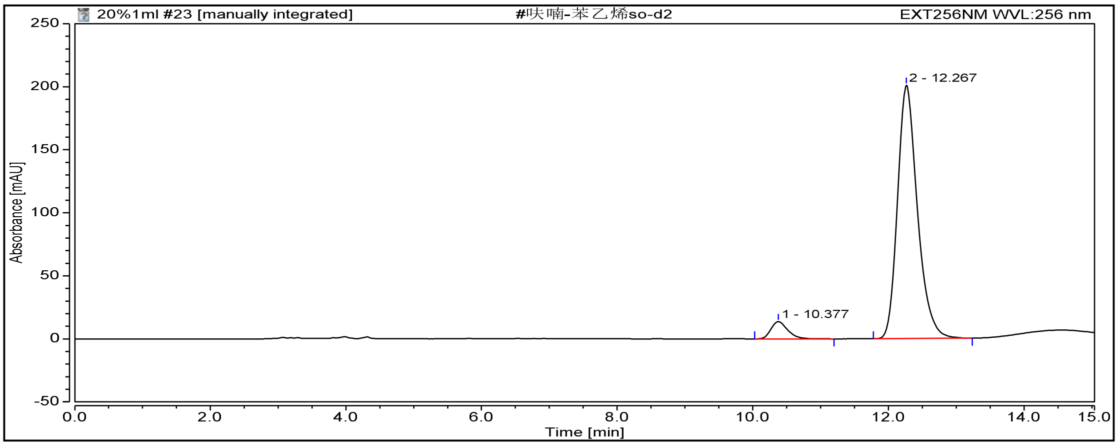

Integration Results					
No.	Peak Name	Retention Time<br>min	Area<br>mAU*min	Relative Area<br>%	Amount<br>n.a.
1		10.377	3.939	5.44	n.a.
2		12.267	68.441	94.56	n.a.
**Total:**			**72.380**	**100.00**	

Supplementary figure 190. HPLC chromatogram for compound **3q’**

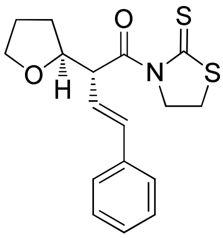

3r

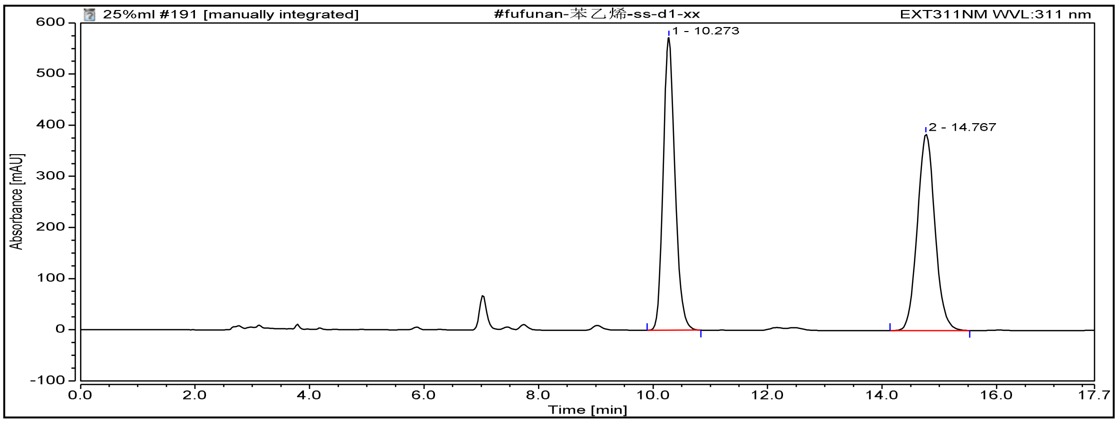

Integration Results					
No.	Peak Name	Retention Time<br>min	Area<br>mAU*min	Relative Area<br>%	Amount<br>n.a.
1		10.273	136.938	50.05	n.a.
2		14.767	136.672	49.95	n.a.
**Total:**			**273.610**	**100.00**	

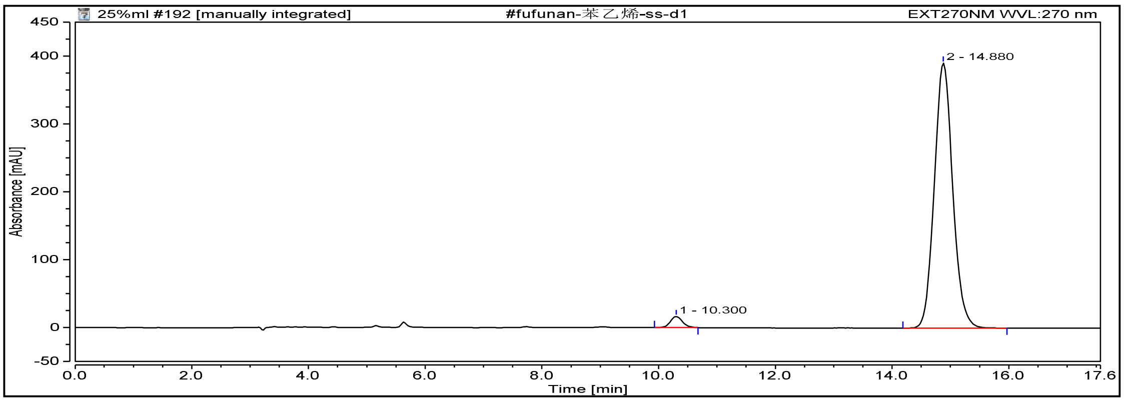

Integration Results					
No.	Peak Name	Retention Time<br>min	Area<br>mAU*min	Relative Area<br>%	Amount<br>n.a.
1		10.300	3.907	2.67	n.a.
2		14.880	142.478	97.33	n.a.
**Total:**			**146.385**	**100.00**	

Supplementary figure 191. HPLC chromatogram for compound **3r**

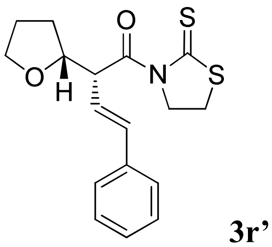

3r'

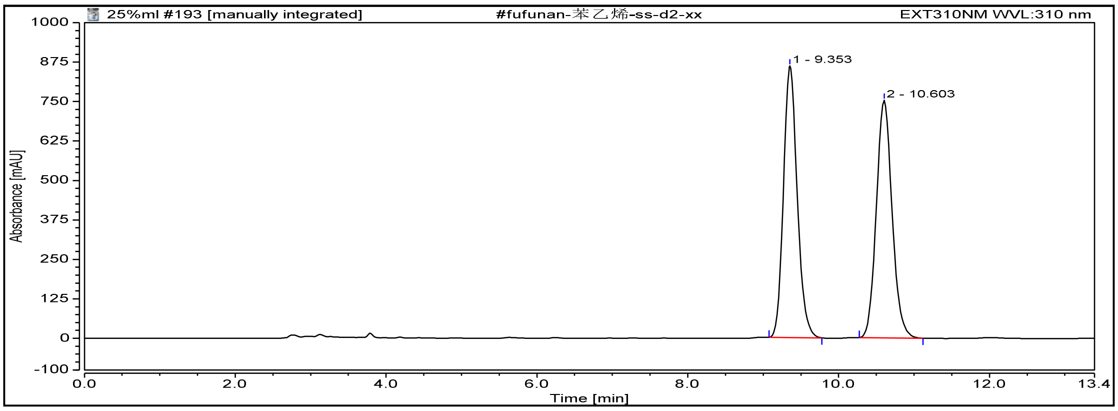

Integration Results					
No.	Peak Name	Retention Time<br>min	Area<br>mAU*min	Relative Area<br>%	Amount<br>n.a.
1		9.353	181.692	49.92	n.a.
2		10.603	182.252	50.08	n.a.
**Total:**			**363.943**	**100.00**	

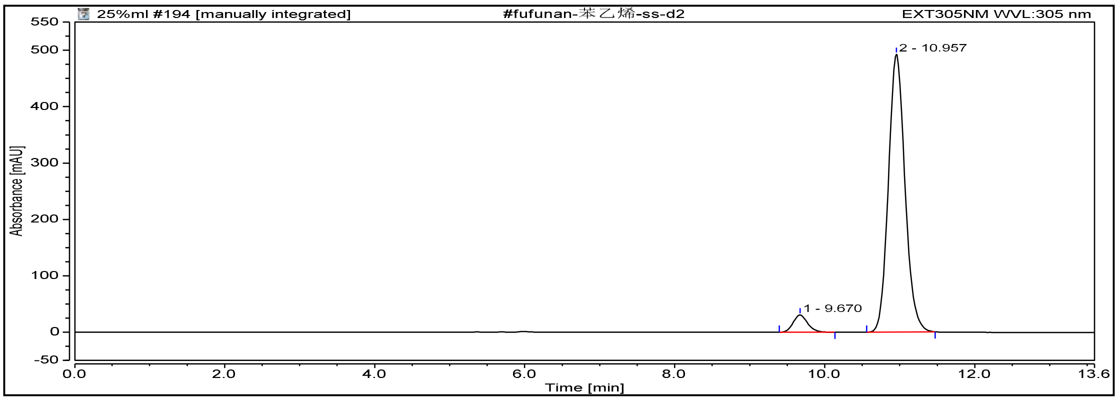

Integration Results					
No.	Peak Name	Retention Time<br>min	Area<br>mAU*min	Relative Area<br>%	Amount<br>n.a.
1		9.670	6.845	5.15	n.a.
2		10.957	125.989	94.85	n.a.
**Total:**			**132.834**	**100.00**	

Supplementary figure 192. HPLC chromatogram for compound **3r'**

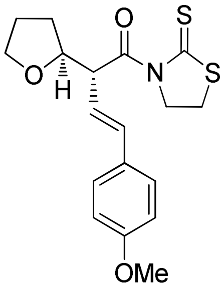

3s

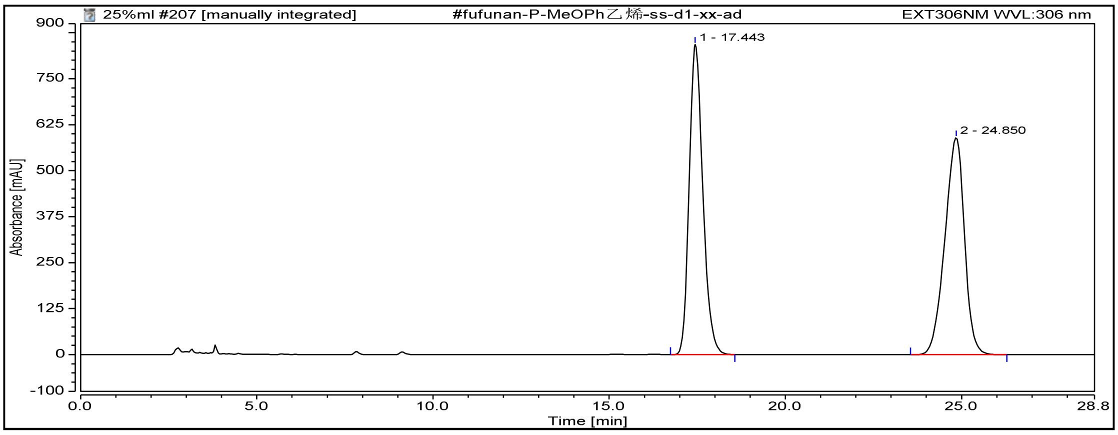

Integration Results					
No.	Peak Name	Retention Time<br>min	Area<br>mAU*min	Relative Area<br>%	Amount<br>n.a.
1		17.443	379.380	49.93	n.a.
2		24.850	380.486	50.07	n.a.
**Total:**			**759.866**	**100.00**	

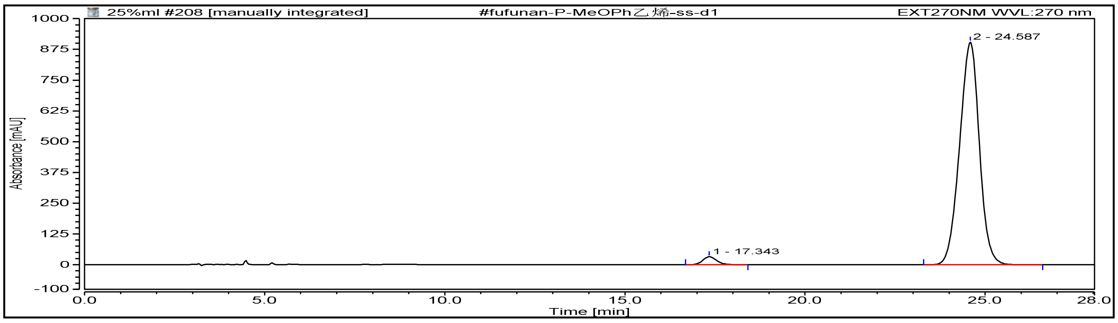

Integration Results					
No.	Peak Name	Retention Time<br>min	Area<br>mAU*min	Relative Area<br>%	Amount<br>n.a.
1		17.343	14.388	2.44	n.a.
2		24.587	574.179	97.56	n.a.
**Total:**			**588.567**	**100.00**	

Supplementary figure 193. HPLC chromatogram for compound **3s**

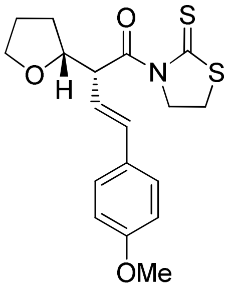

3s'

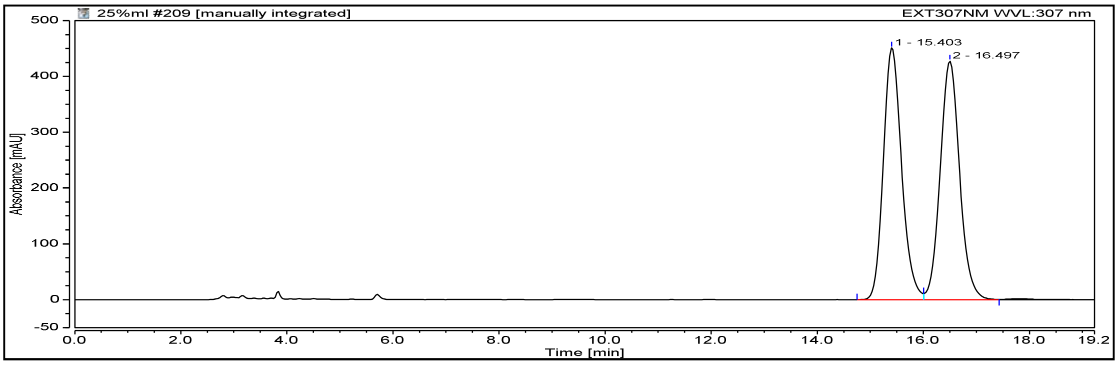

Integration Results					
No.	Peak Name	Retention Time<br>min	Area<br>mAU*min	Relative Area<br>%	Amount<br>n.a.
1		15.403	180.707	49.83	n.a.
2		16.497	181.926	50.17	n.a.
**Total:**			**362.634**	**100.00**	

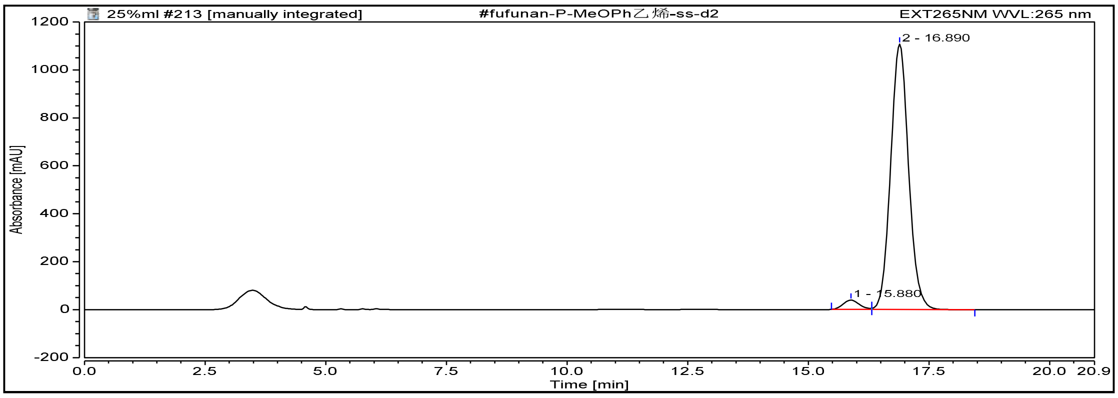

Integration Results					
No.	Peak Name	Retention Time<br>min	Height<br>mAU	Relative Area<br>%	Amount<br>n.a.
1		15.880	39.019	3.08	n.a.
2		16.890	1107.279	96.92	n.a.
**Total:**			**1146.298**	**100.00**	

Supplementary figure 194. HPLC chromatogram for compound **3s'**

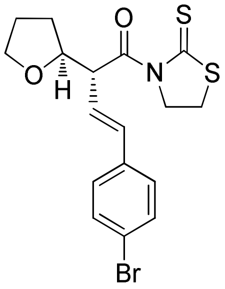

3t

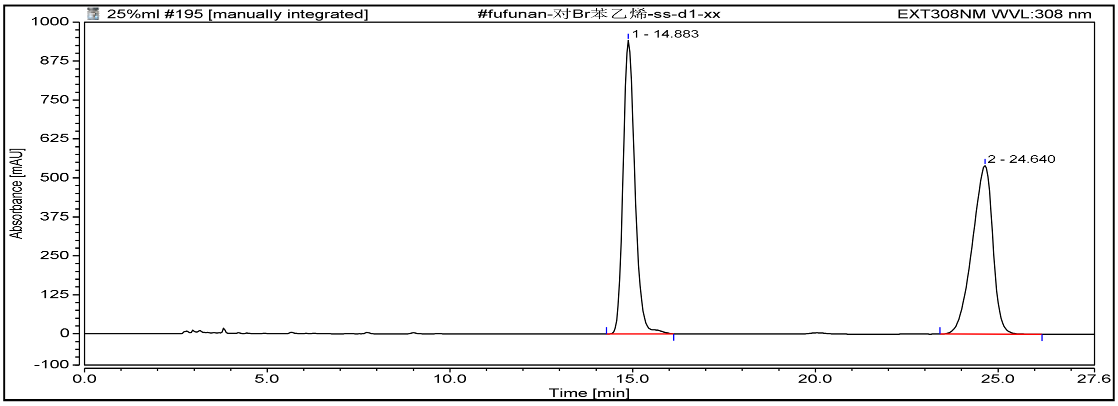

Integration Results					
No.	Peak Name	Retention Time<br>min	Area<br>mAU*min	Relative Area<br>%	Amount<br>n.a.
1		14.883	353.629	50.23	n.a.
2		24.640	350.377	49.77	n.a.
**Total:**			**704.006**	**100.00**	

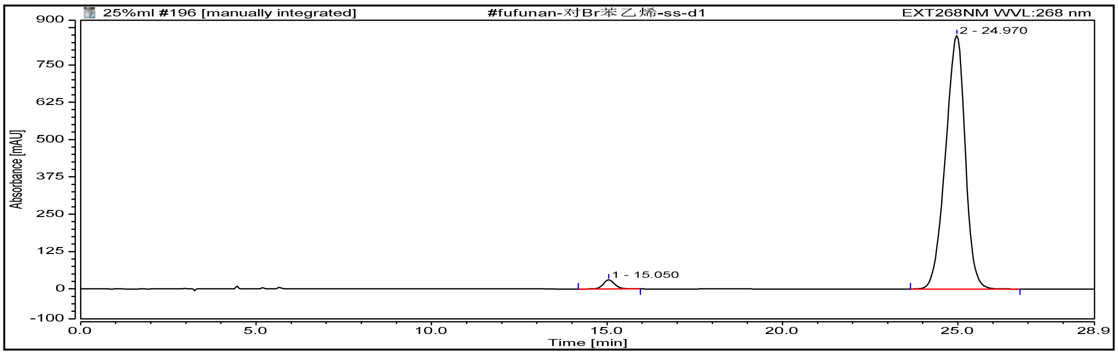

Integration Results					
No.	Peak Name	Retention Time<br>min	Area<br>mAU*min	Relative Area<br>%	Amount<br>n.a.
1		15.050	11.468	2.04	n.a.
2		24.970	551.723	97.96	n.a.
**Total:**			**563.192**	**100.00**	

Supplementary figure 195. HPLC chromatogram for compound **3t**

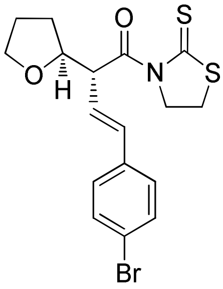

3t'

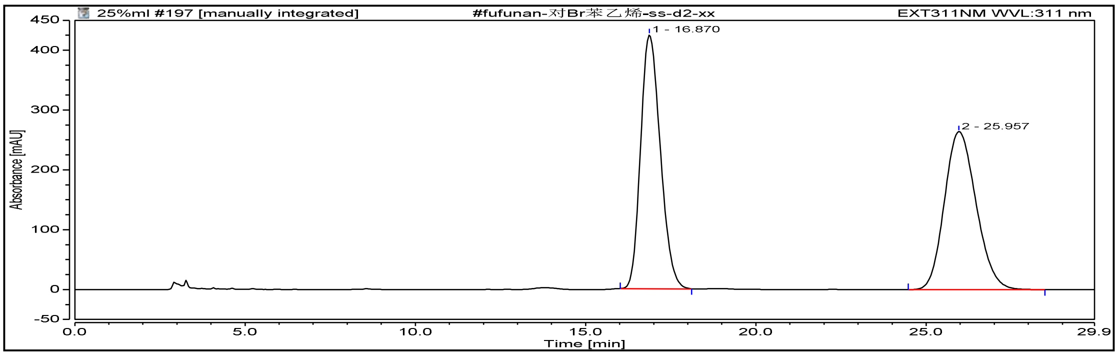

Integration Results					
No.	Peak Name	Retention Time<br>min	Area<br>mAU*min	Relative Area<br>%	Amount<br>n.a.
1		16.870	284.860	50.00	n.a.
2		25.957	284.875	50.00	n.a.
**Total:**			**569.735**	**100.00**	

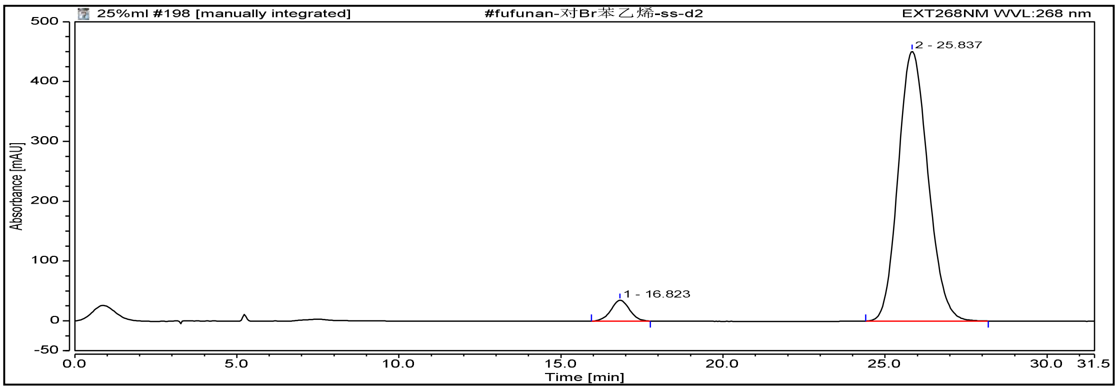

Integration Results					
No.	Peak Name	Retention Time<br>min	Area<br>mAU*min	Relative Area<br>%	Amount<br>n.a.
1		16.823	23.121	4.59	n.a.
2		25.837	480.275	95.41	n.a.
**Total:**			**503.395**	**100.00**	

Supplementary figure 196. HPLC chromatogram for compound **3t'**

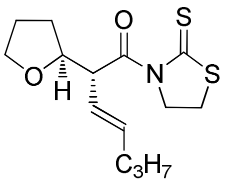

3u

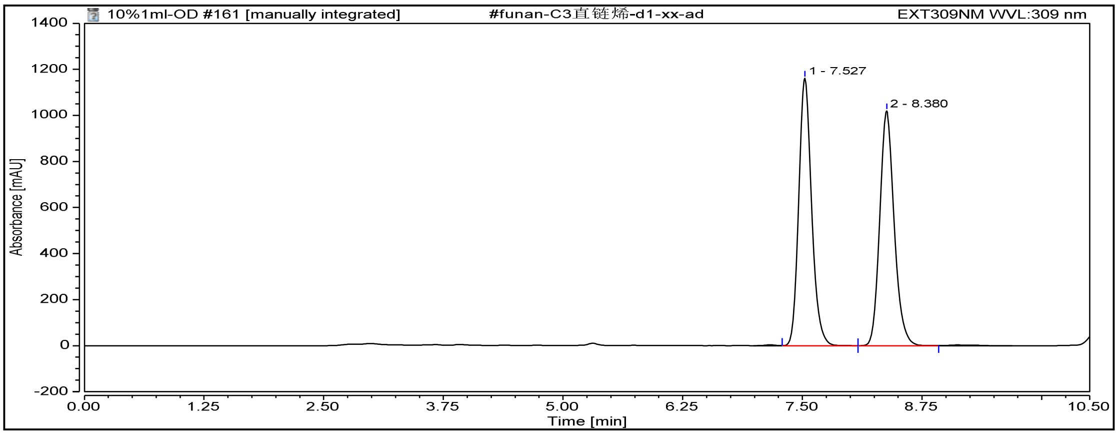

Integration Results					
No.	Peak Name	Retention Time min	Area mAU*min	Relative Area %	Amount n.a.
1		7.527	180.730	49.96	n.a.
2		8.380	181.013	50.04	n.a.
**Total:**			**361.742**	**100.00**	

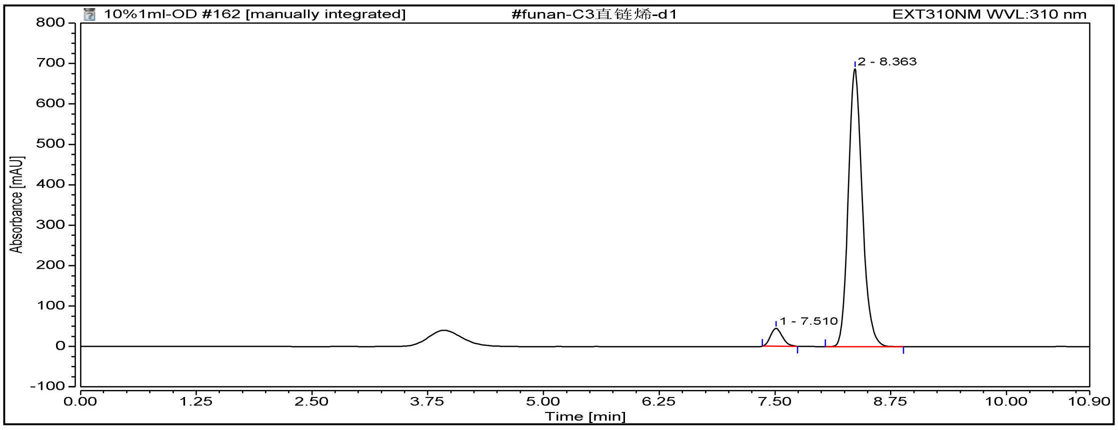

Integration Results					
No.	Peak Name	Retention Time min	Area mAU*min	Relative Area %	Amount n.a.
1		7.510	6.543	5.15	n.a.
2		8.363	120.381	94.85	n.a.
**Total:**			**126.924**	**100.00**	

Supplementary figure 197. HPLC chromatogram for compound **3u**

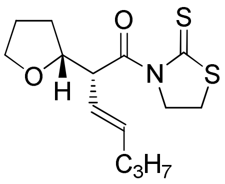

3u’

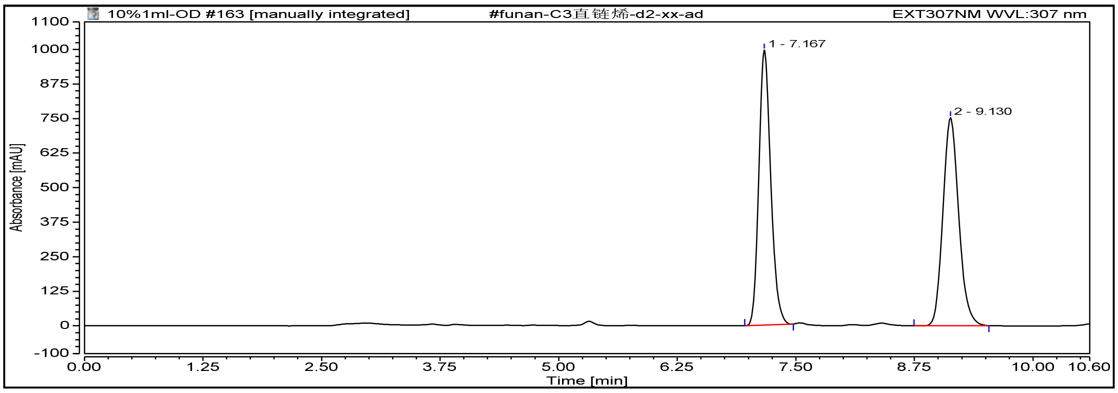

Integration Results

No.	Peak Name	Retention Time<br>min	Area<br>mAU*min	Relative Area<br>%	Amount<br>n.a.
1		7.167	143.766	49.81	n.a.
2		9.130	144.865	50.19	n.a.
**Total:**			**288.631**	**100.00**	

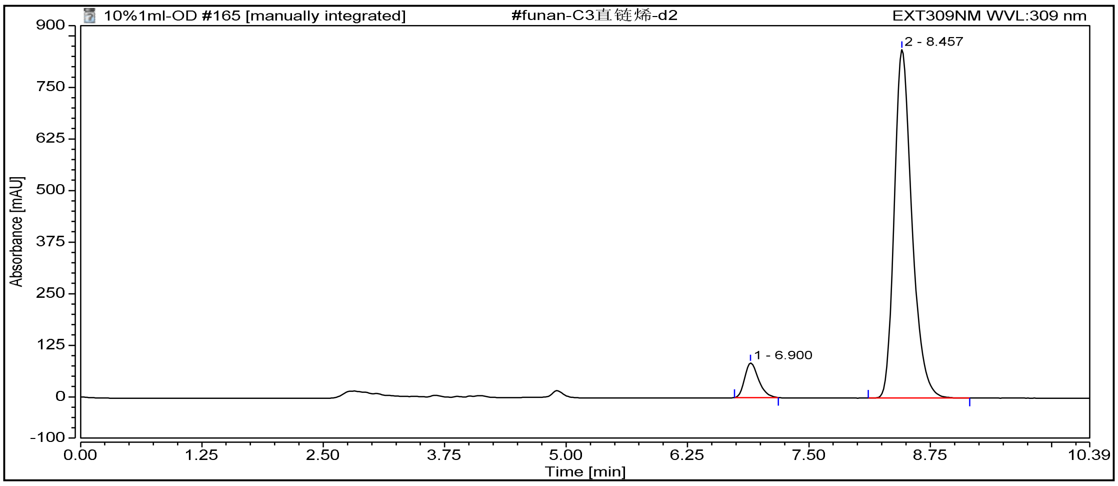

Integration Results

No.	Peak Name	Retention Time<br>min	Area<br>mAU*min	Relative Area<br>%	Amount<br>n.a.
1		6.900	13.757	7.12	n.a.
2		8.457	179.361	92.88	n.a.
**Total:**			**193.118**	**100.00**	

Supplementary figure 198. HPLC chromatogram for compound **3u’**

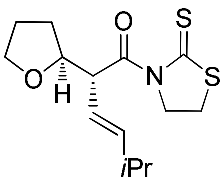

3v

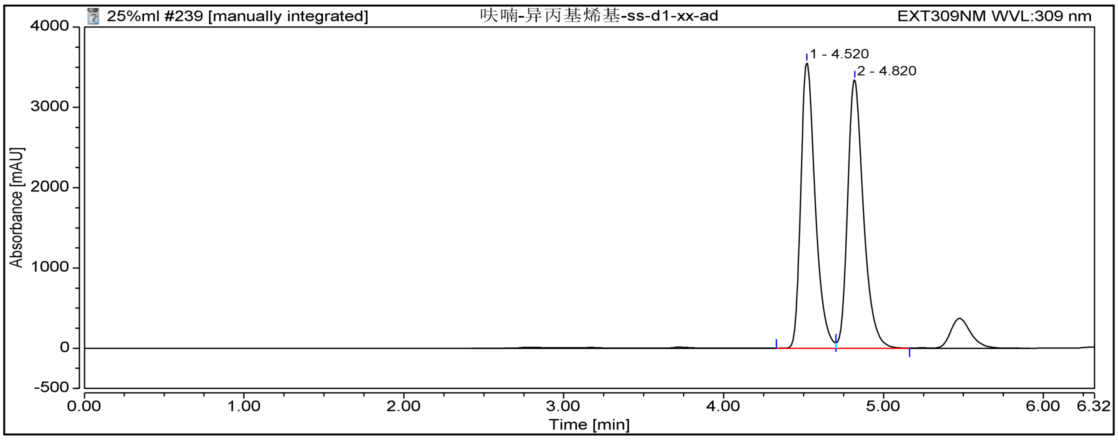

Integration Results					
No.	Peak Name	Retention Time<br>min	Area<br>mAU*min	Relative Area<br>%	Amount<br>n.a.
1		4.520	377.683	49.59	n.a.
2		4.820	383.854	50.41	n.a.
**Total:**			**761.536**	**100.00**	

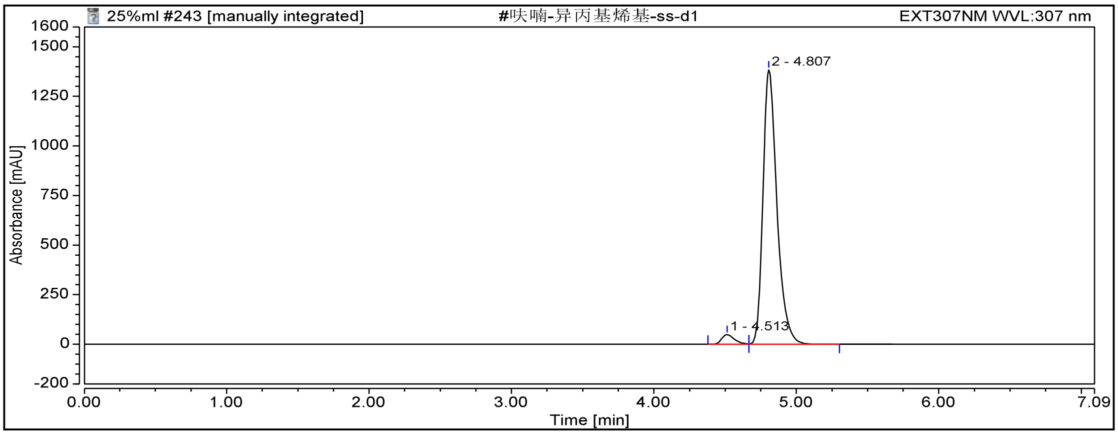

Integration Results					
No.	Peak Name	Retention Time<br>min	Area<br>mAU*min	Relative Area<br>%	Amount<br>n.a.
1		4.513	5.100	3.15	n.a.
2		4.807	156.988	96.85	n.a.
**Total:**			**162.088**	**100.00**	

Supplementary figure 199. HPLC chromatogram for compound **3v**

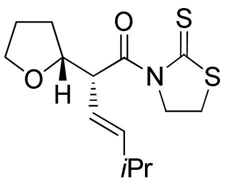

3v'

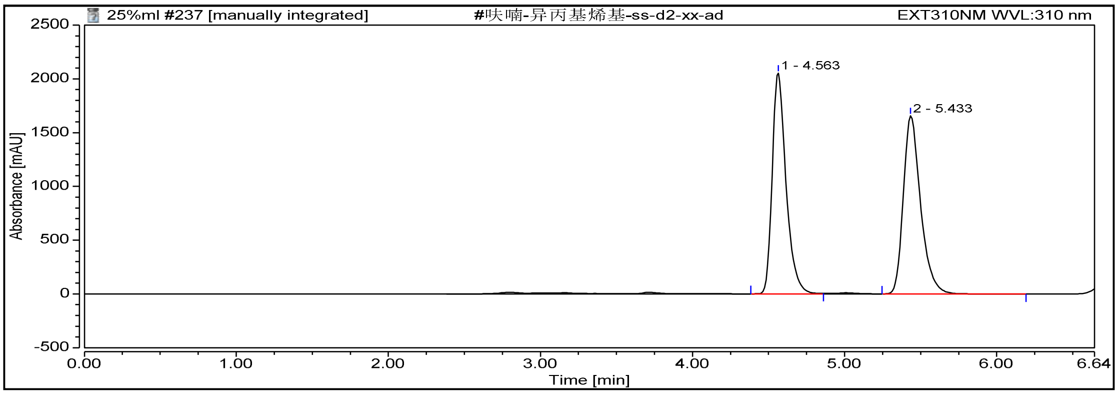

Integration Results					
No.	Peak Name	Retention Time min	Area mAU*min	Relative Area %	Amount n.a.
1		4.563	216.584	49.92	n.a.
2		5.433	217.255	50.08	n.a.
**Total :**			**433.839**	**100.00**	

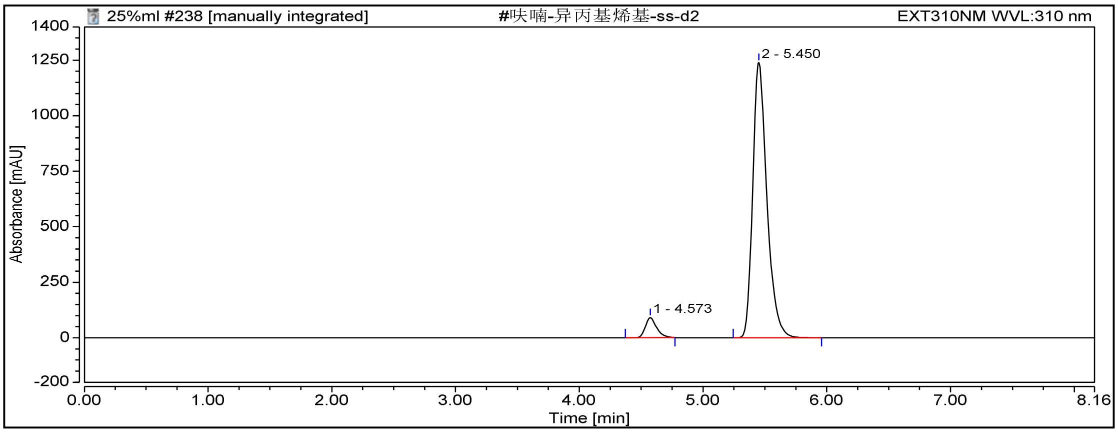

Integration Results					
No.	Peak Name	Retention Time min	Area mAU*min	Relative Area %	Amount n.a.
1		4.573	9.533	5.48	n.a.
2		5.450	164.513	94.52	n.a.
**Total:**			**174.046**	**100.00**	

Supplementary figure 200. HPLC chromatogram for compound **3v'**

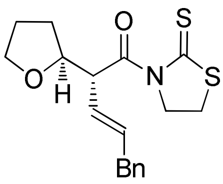

3w

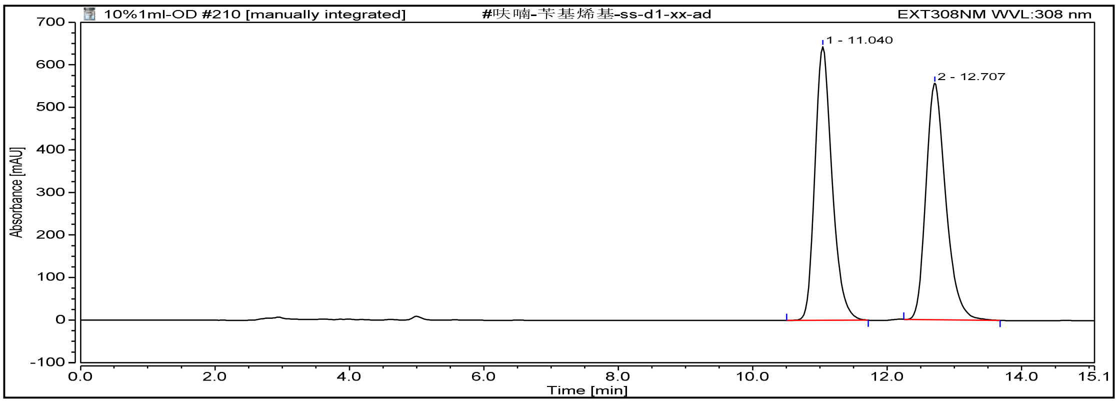

Integration Results					
No.	Peak Name	Retention Time<br>min	Area<br>mAU*min	Relative Area<br>%	Amount<br>n.a.
1		11.040	190.740	50.13	n.a.
2		12.707	189.727	49.87	n.a.
**Total:**			**380.467**	**100.00**	

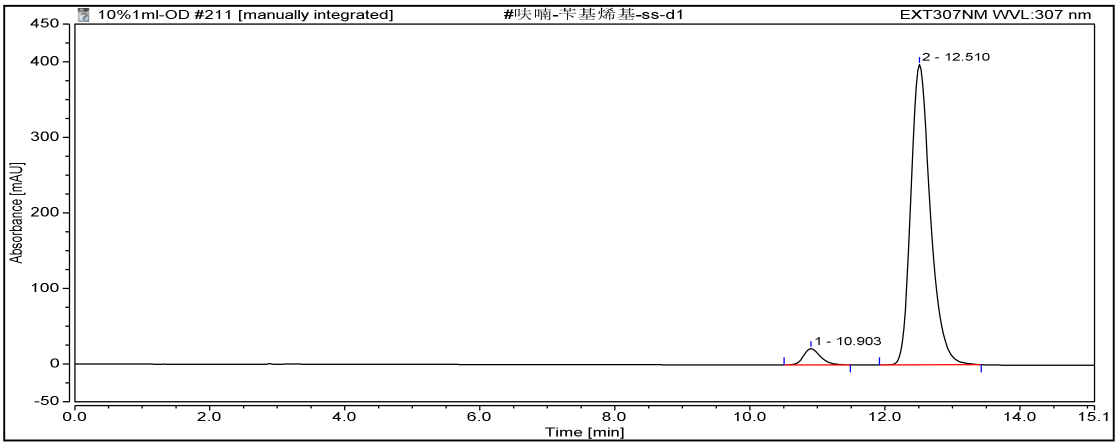

Integration Results					
No.	Peak Name	Retention Time<br>min	Area<br>mAU*min	Relative Area<br>%	Amount<br>n.a.
1		10.903	6.193	4.44	n.a.
2		12.510	133.259	95.56	n.a.
**Total:**			**139.452**	**100.00**	

Supplementary figure 201. HPLC chromatogram for compound **3w**

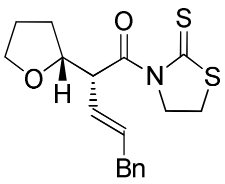

3w'

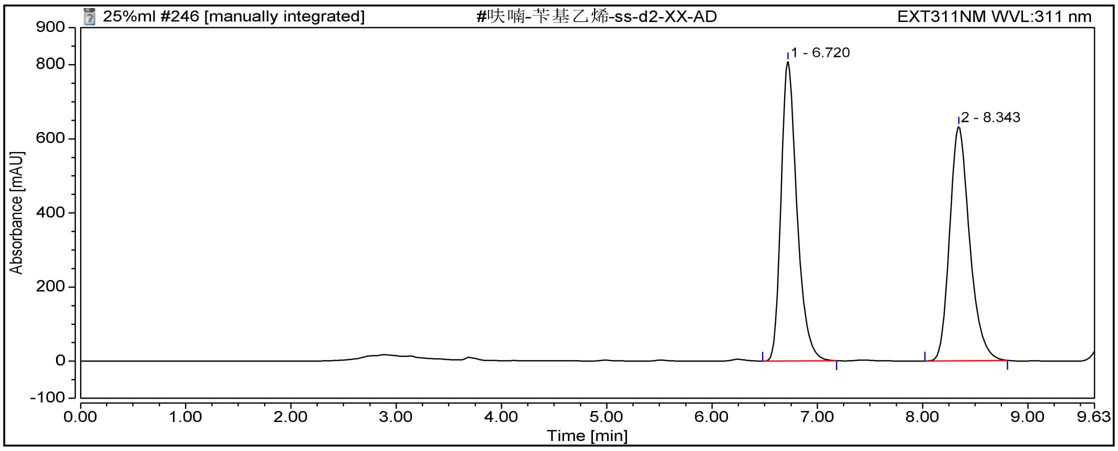

Integration Results					
No.	Peak Name	Retention Time<br>min	Area<br>mAU*min	Relative Area<br>%	Amount<br>n.a.
1		6.720	141.432	50.35	n.a.
2		8.343	139.464	49.65	n.a.
**Total:**			**280.896**	**100.00**	

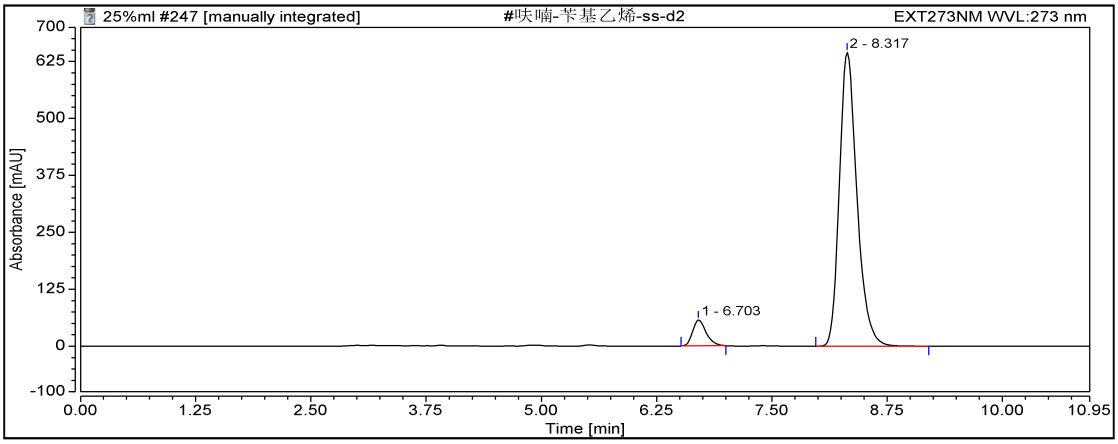

Integration Results					
No.	Peak Name	Retention Time<br>min	Area<br>mAU*min	Relative Area<br>%	Amount<br>n.a.
1		6.703	9.662	6.35	n.a.
2		8.317	142.552	93.65	n.a.
**Total:**			**152.215**	**100.00**	

Supplementary figure 202. HPLC chromatogram for compound **3w'**

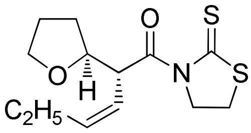

3x

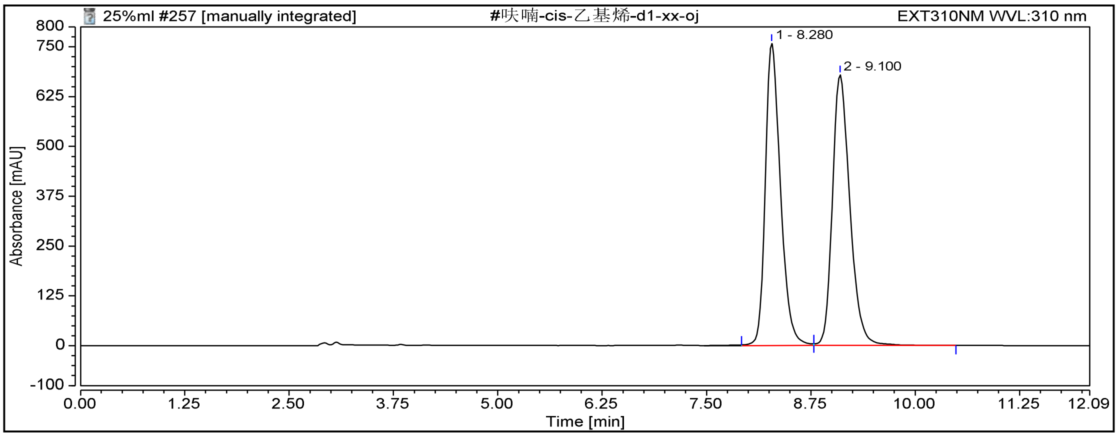

Integration Results					
No.	Peak Name	Retention Time<br>min	Area<br>mAU*min	Relative Area<br>%	Amount<br>n.a.
1		8.280	160.041	49.24	n.a.
2		9.100	164.963	50.76	n.a.
**Total:**			**325.004**	**100.00**	

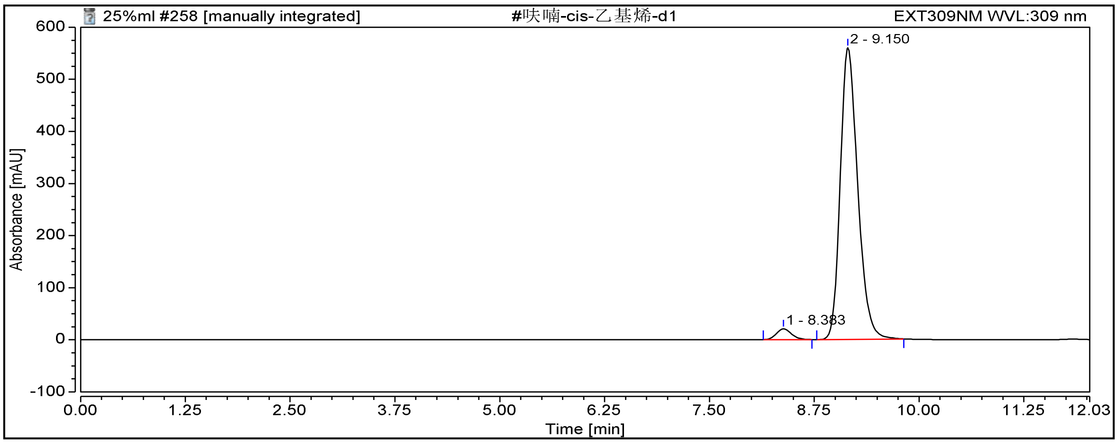

Integration Results					
No.	Peak Name	Retention Time<br>min	Area<br>mAU*min	Relative Area<br>%	Amount<br>n.a.
1		8.383	4.331	3.11	n.a.
2		9.150	134.936	96.89	n.a.
**Total:**			**139.267**	**100.00**	

Supplementary figure 203. HPLC chromatogram for compound **3x**

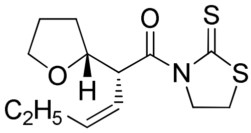

3x'

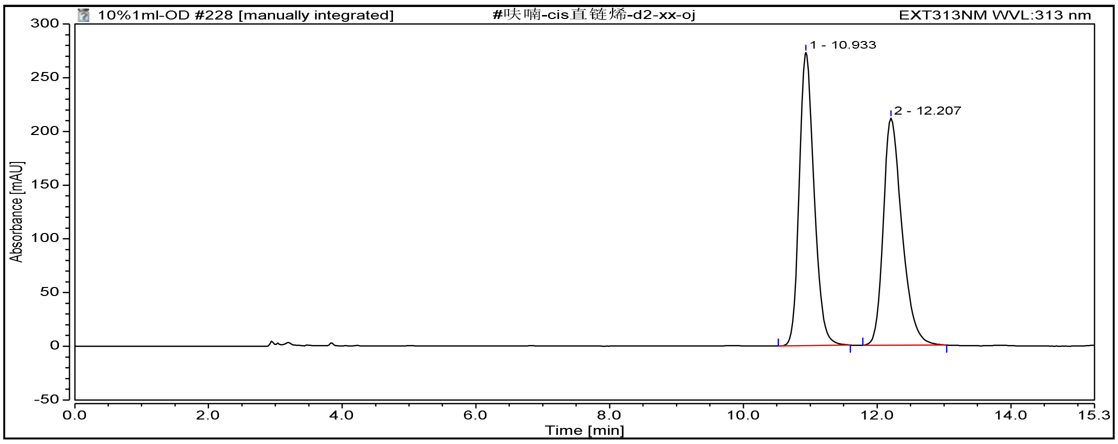

Integration Results					
No.	Peak Name	Retention Time<br>min	Area<br>mAU*min	Relative Area<br>%	Amount<br>n.a.
1		10.933	70.275	50.57	n.a.
2		12.207	68.687	49.43	n.a.
**Total:**			**138.962**	**100.00**	

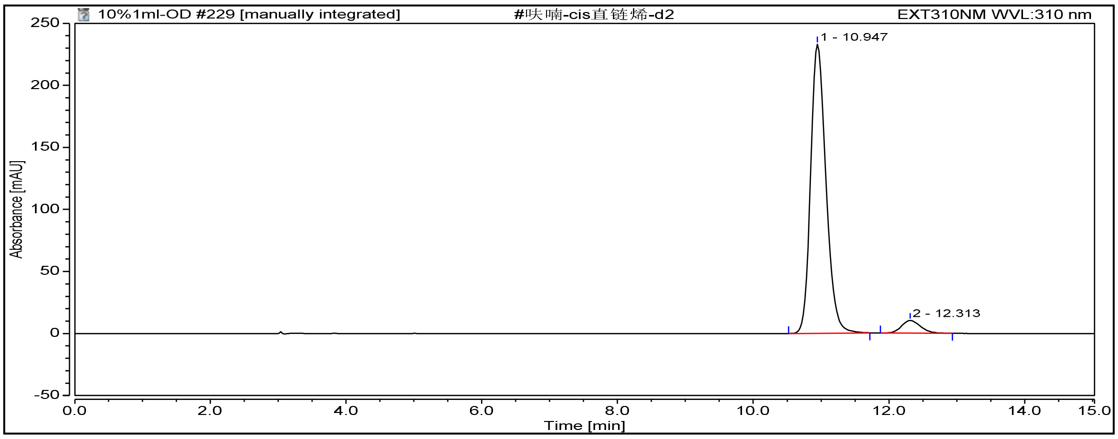

Integration Results					
No.	Peak Name	Retention Time<br>min	Area<br>mAU*min	Relative Area<br>%	Amount<br>n.a.
1		10.947	60.843	94.82	n.a.
2		12.313	3.324	5.18	n.a.
**Total:**			**64.167**	**100.00**	

Supplementary figure 204. HPLC chromatogram for compound **3x'**

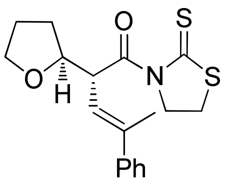

3y

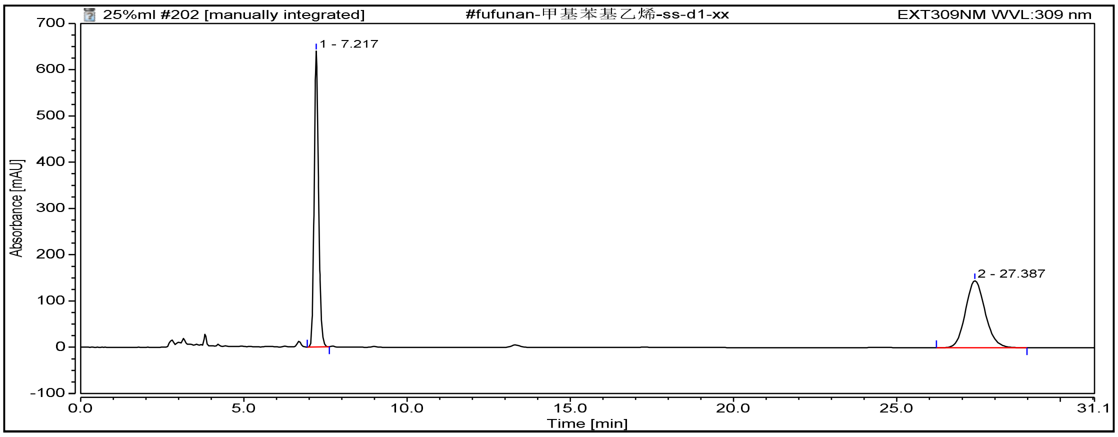

Integration Results					
No.	Peak Name	Retention Time<br>min	Area<br>mAU*min	Relative Area<br>%	Amount<br>n.a.
1		7.217	100.376	49.94	n.a.
2		27.387	100.616	50.06	n.a.
**Total:**			**200.992**	**100.00**	

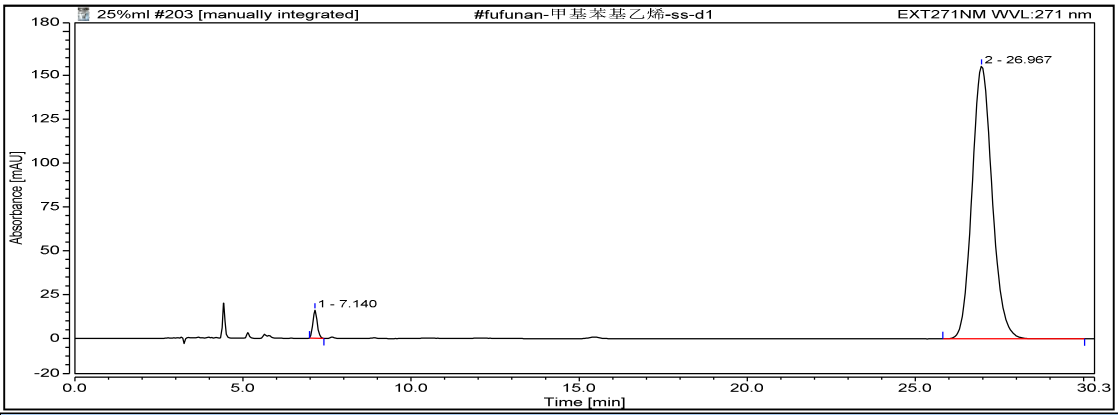

Integration Results					
No.	Peak Name	Retention Time<br>min	Area<br>mAU*min	Relative Area<br>%	Amount<br>n.a.
1		7.140	2.408	2.18	n.a.
2		26.967	107.953	97.82	n.a.
**Total:**			**110.361**	**100.00**	

Supplementary figure 205. HPLC chromatogram for compound **3y**

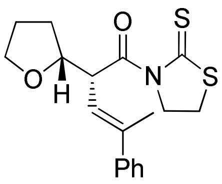

3y’

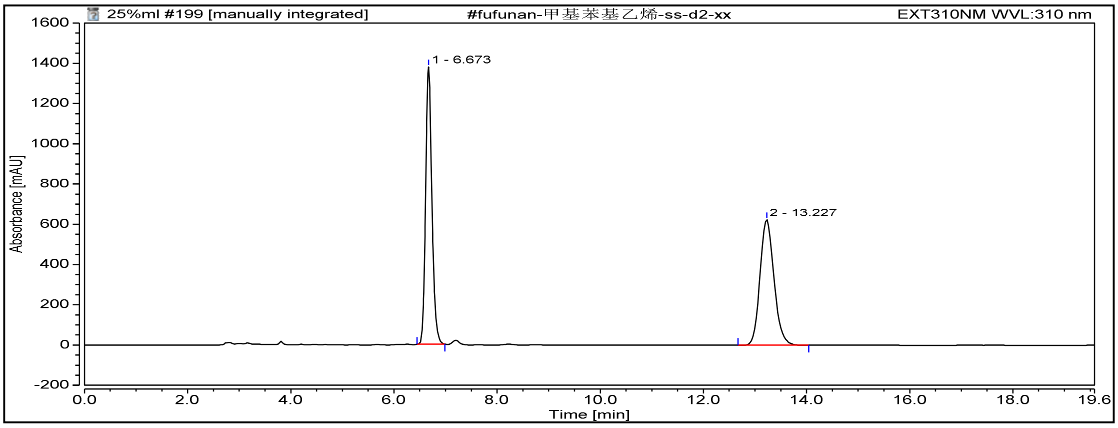

Integration Results					
No.	Peak Name	Retention Time<br>min	Area<br>mAU*min	Relative Area<br>%	Amount<br>n.a.
1		6.673	194.920	49.84	n.a.
2		13.227	196.208	50.16	n.a.
**Total:**			**391.128**	**100.00**	

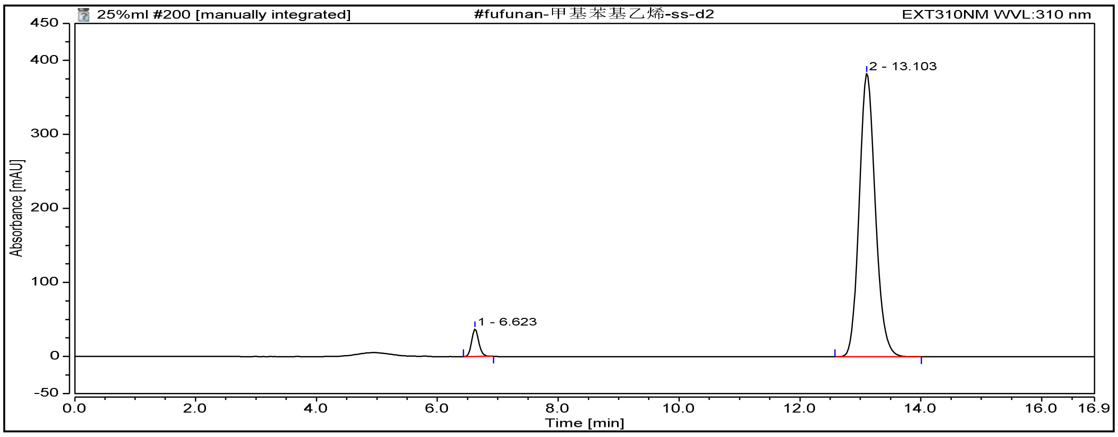

Integration Results					
No.	Peak Name	Retention Time<br>min	Area<br>mAU*min	Relative Area<br>%	Amount<br>n.a.
1		6.623	5.128	4.14	n.a.
2		13.103	118.880	95.86	n.a.
**Total:**			**124.008**	**100.00**	

Supplementary figure 206. HPLC chromatogram for compound **3y’**

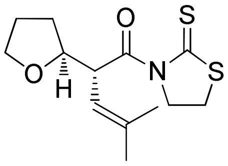

3z

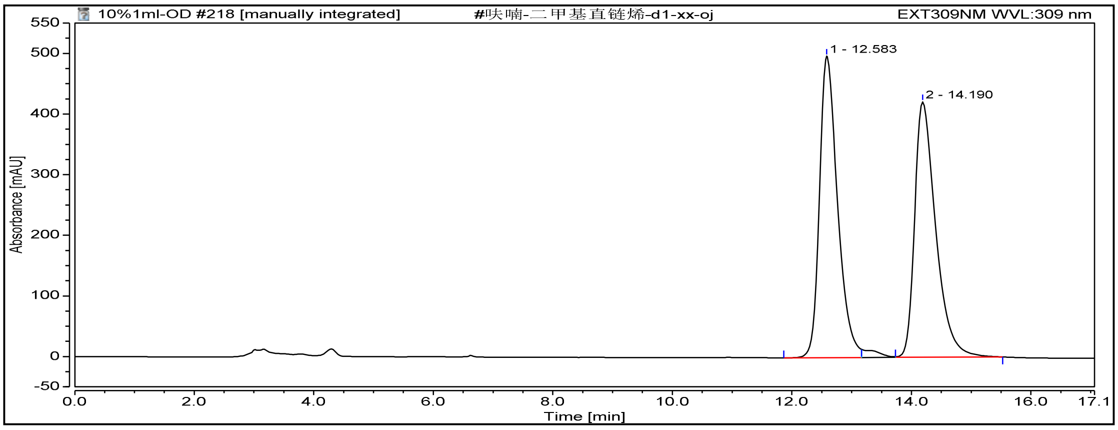

Integration Results					
No.	Peak Name	Retention Time<br>min	Area<br>mAU*min	Relative Area<br>%	Amount<br>n.a.
1		12.583	174.966	50.34	n.a.
2		14.190	172.621	49.66	n.a.
**Total:**			**347.587**	**100.00**	

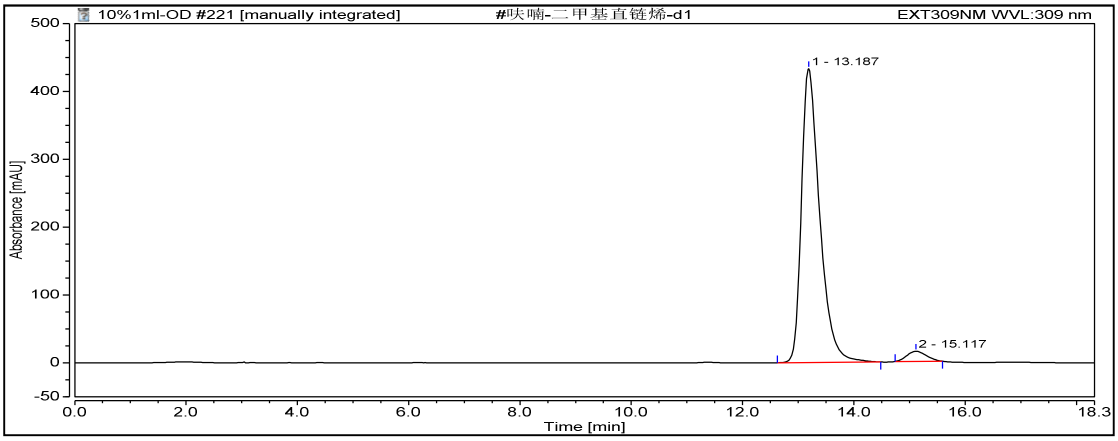

Integration Results					
No.	Peak Name	Retention Time<br>min	Area<br>mAU*min	Relative Area<br>%	Amount<br>n.a.
1		13.187	164.465	96.45	n.a.
2		15.117	6.055	3.55	n.a.
**Total:**			**170.520**	**100.00**	

Supplementary figure 207. HPLC chromatogram for compound **3z**

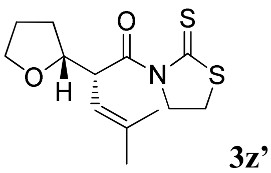

3z'

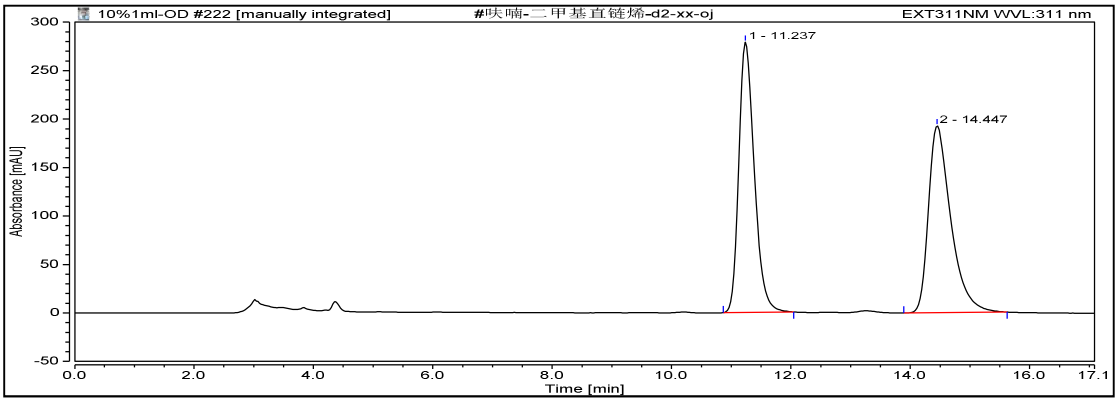

Integration Results					
No.	Peak Name	Retention Time<br>min	Area<br>mAU*min	Relative Area<br>%	Amount<br>n.a.
1		11.237	83.953	49.95	n.a.
2		14.447	84.133	50.05	n.a.
**Total:**			**168.085**	**100.00**	

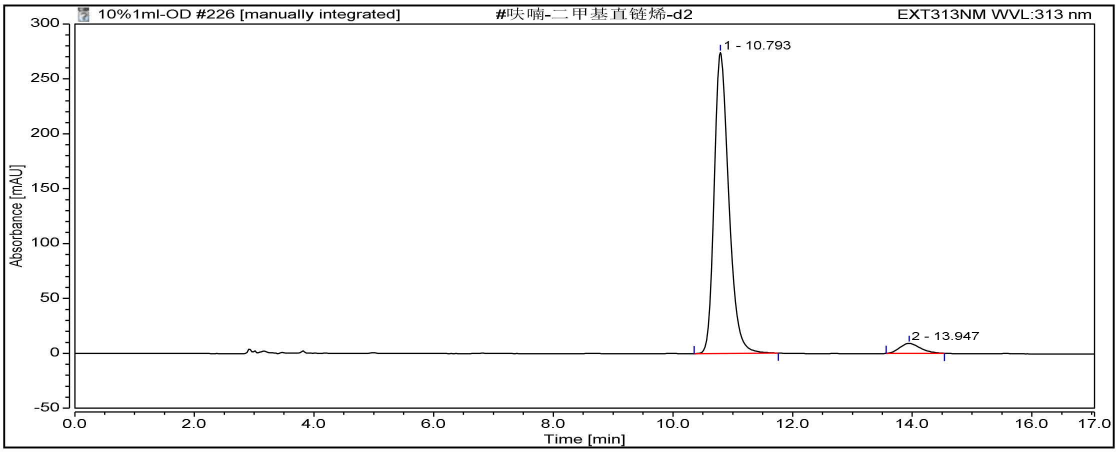

Integration Results					
No.	Peak Name	Retention Time<br>min	Area<br>mAU*min	Relative Area<br>%	Amount<br>n.a.
1		10.793	77.422	95.51	n.a.
2		13.947	3.643	4.49	n.a.
**Total:**			**81.065**	**100.00**	

Supplementary figure 208. HPLC chromatogram for compound **3z'**

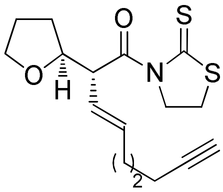

3ba

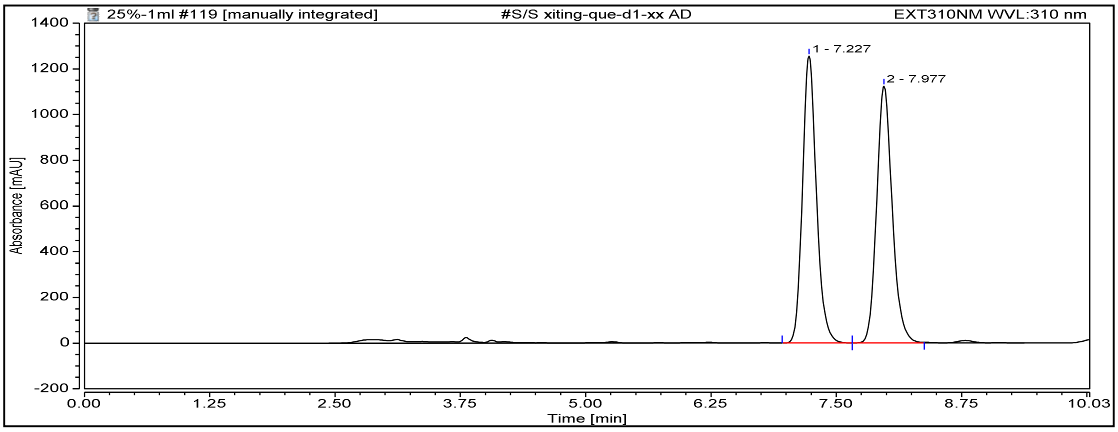

Integration Results					
No.	Peak Name	Retention Time<br>min	Area<br>mAU*min	Relative Area<br>%	Amount<br>n.a.
1		7.227	202.769	49.93	n.a.
2		7.977	203.302	50.07	n.a.
**Total:**			**406.070**	**100.00**	

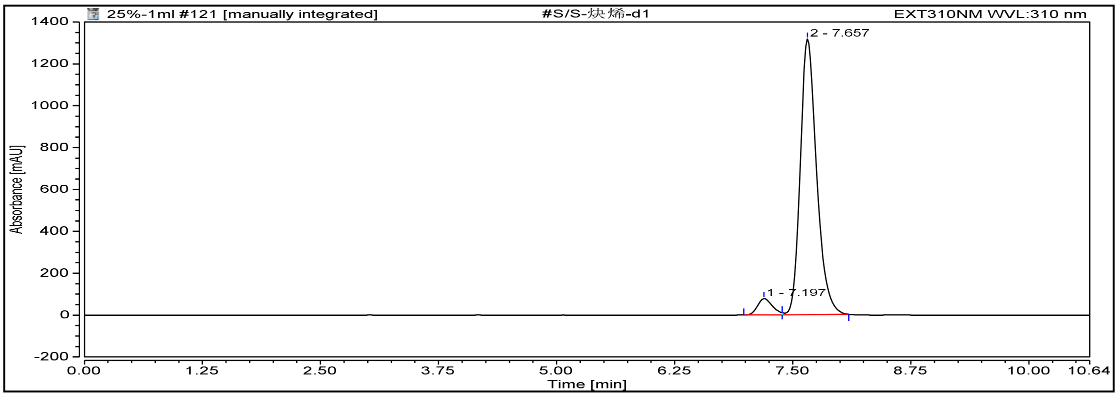

Integration Results					
No.	Peak Name	Retention Time<br>min	Area<br>mAU*min	Relative Area<br>%	Amount<br>n.a.
1		7.197	14.100	5.06	n.a.
2		7.657	264.429	94.94	n.a.
**Total:**			**278.529**	**100.00**	

Supplementary figure 209. HPLC chromatogram for compound **3ba**

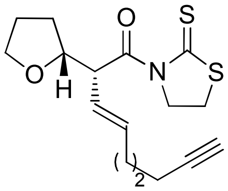

3ba’

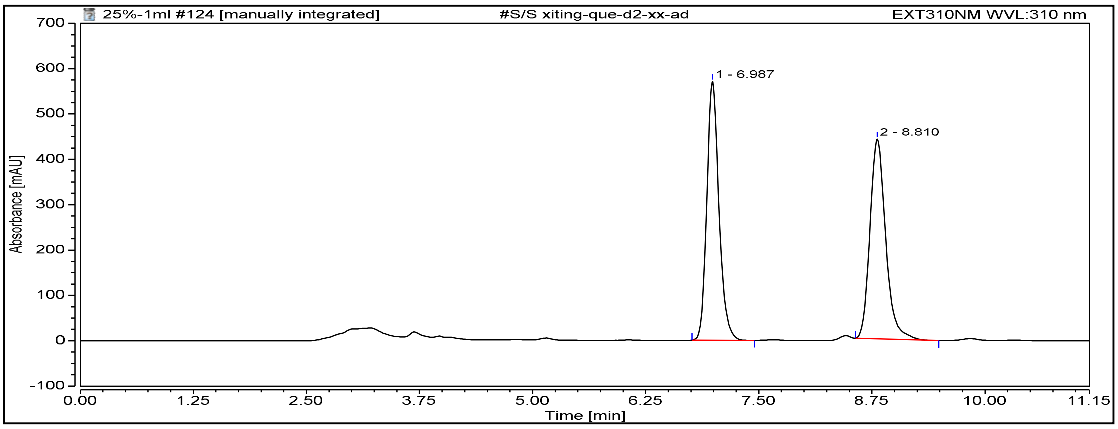

Integration Results					
No.	Peak Name	Retention Time<br>min	Area<br>mAU*min	Relative Area<br>%	Amount<br>n.a.
1		6.987	89.122	50.12	n.a.
2		8.810	88.698	49.88	n.a.
**Total:**			**177.820**	**100.00**	

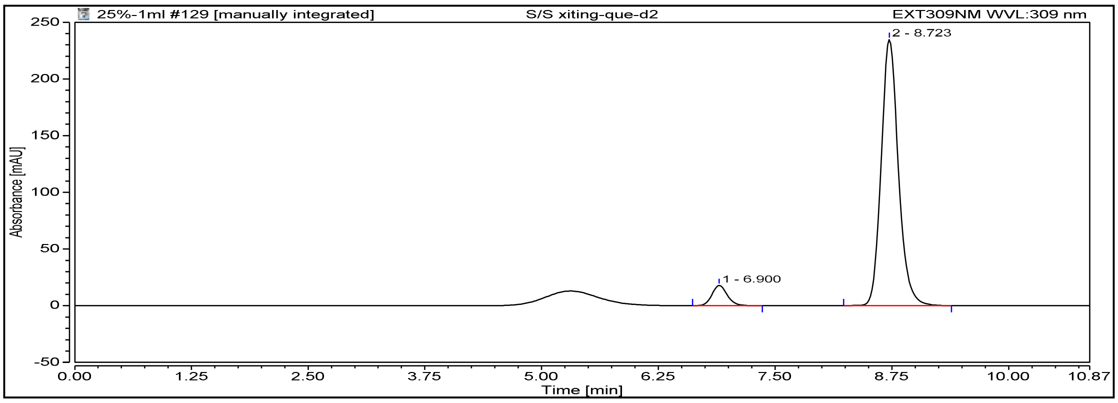

Integration Results					
No.	Peak Name	Retention Time<br>min	Area<br>mAU*min	Relative Area<br>%	Amount<br>n.a.
1		6.900	3.382	6.32	n.a.
2		8.723	50.160	93.68	n.a.
**Total:**			**53.542**	**100.00**	

Supplementary figure 210. HPLC chromatogram for compound **3ba’**

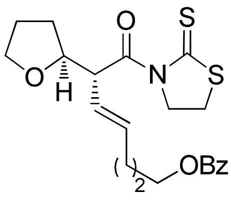

3bb

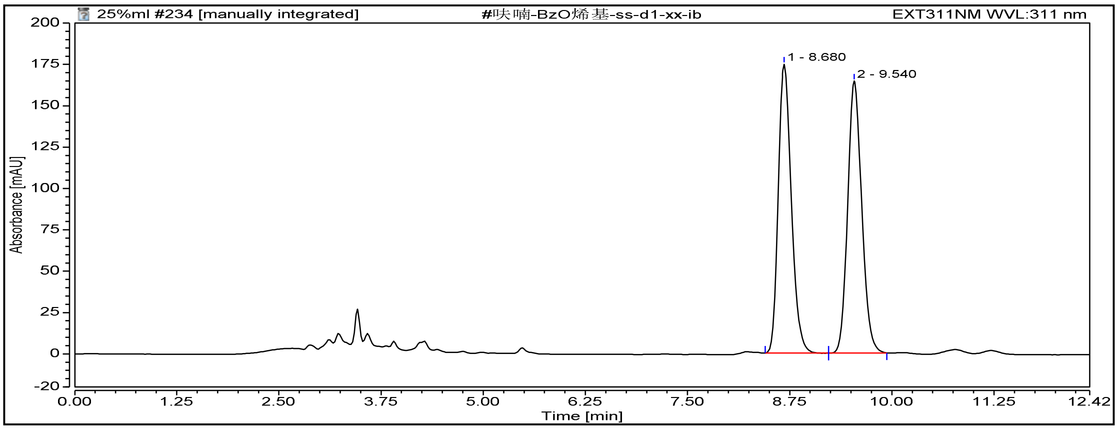

Integration Results					
No.	Peak Name	Retention Time<br>min	Area<br>mAU*min	Relative Area<br>%	Amount<br>n.a.
1		8.680	32.331	49.58	n.a.
2		9.540	32.874	50.42	n.a.
**Total:**			**65.205**	**100.00**	

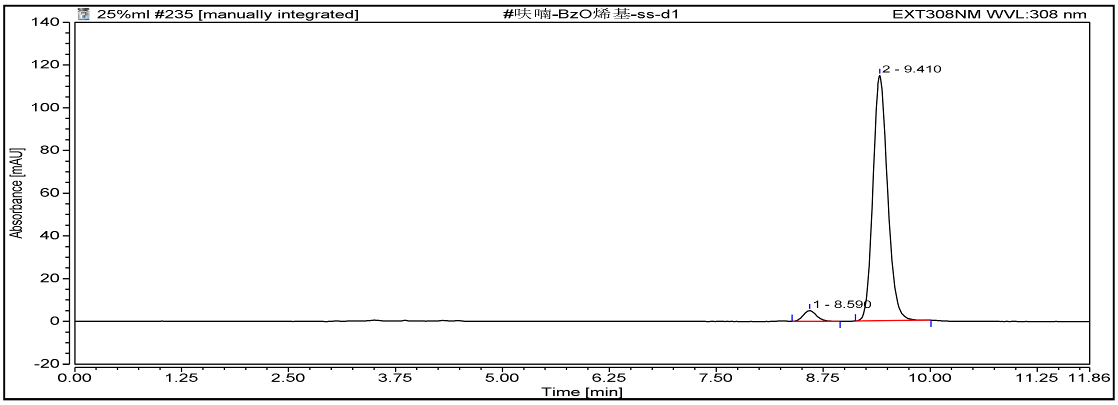

Integration Results					
No.	Peak Name	Retention Time<br>min	Area<br>mAU*min	Relative Area<br>%	Amount<br>n.a.
1		8.590	0.895	3.78	n.a.
2		9.410	22.759	96.22	n.a.
**Total:**			**23.654**	**100.00**	

Supplementary figure 211. HPLC chromatogram for compound **3bb**

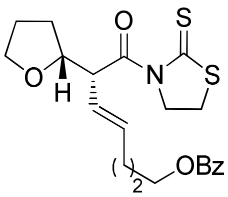

3bb’

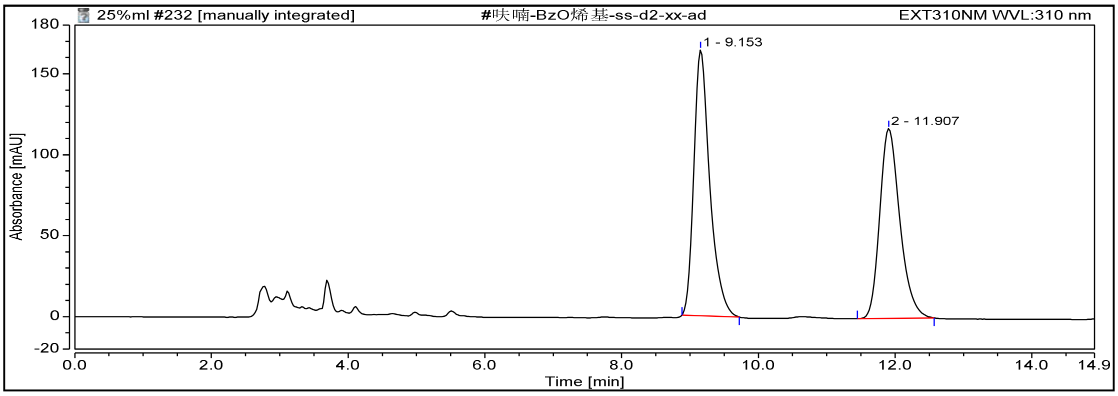

Integration Results					
No.	Peak Name	Retention Time<br>min	Area<br>mAU*min	Relative Area<br>%	Amount<br>n.a.
1		9.153	44.311	52.67	n.a.
2		11.907	39.816	47.33	n.a.
**Total:**			**84.127**	**100.00**	

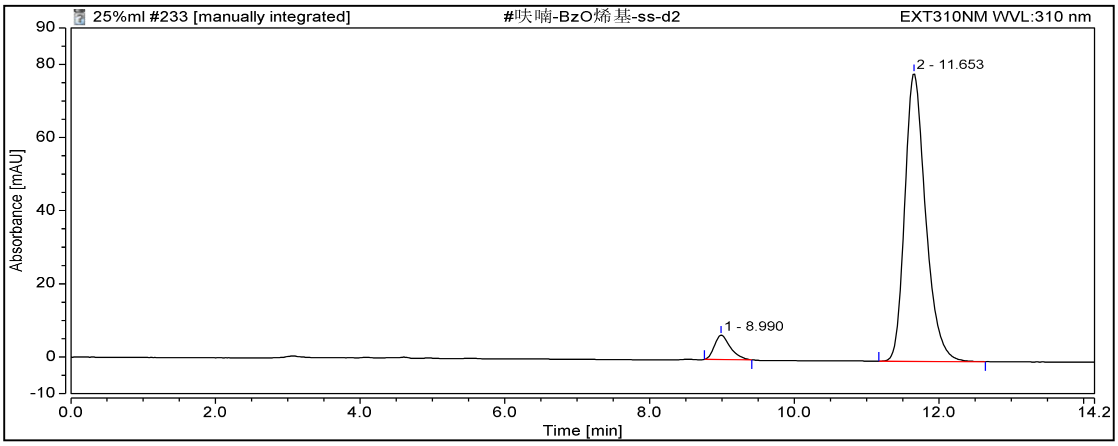

Integration Results					
No.	Peak Name	Retention Time<br>min	Area<br>mAU*min	Relative Area<br>%	Amount<br>n.a.
1		8.990	1.686	6.03	n.a.
2		11.653	26.279	93.97	n.a.
**Total:**			**27.966**	**100.00**	

Supplementary figure 212. HPLC chromatogram for compound **3bb’**

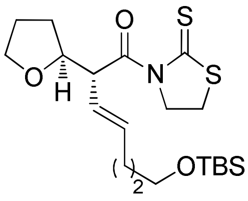

3bc

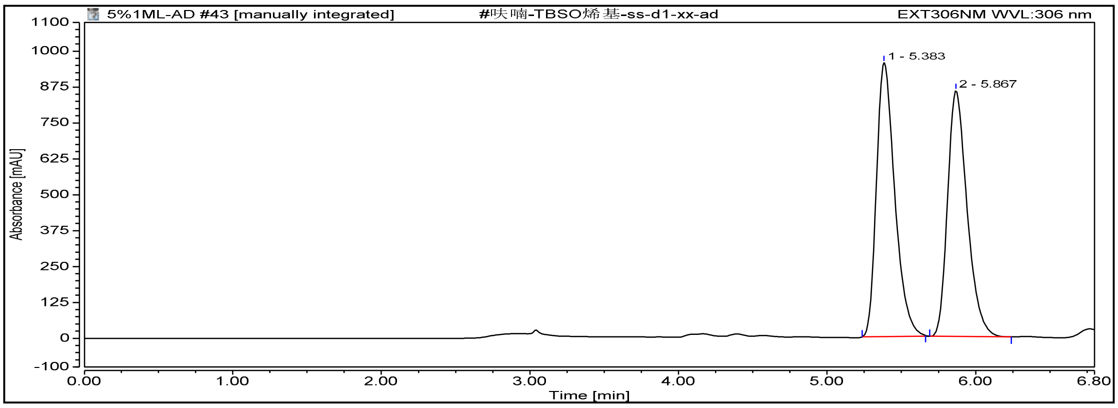

Integration Results					
No.	Peak Name	Retention Time<br>min	Area<br>mAU*min	Relative Area<br>%	Amount<br>n.a.
1		5.383	136.550	51.11	n.a.
2		5.867	130.616	48.89	n.a.
**Total:**			**267.166**	**100.00**	

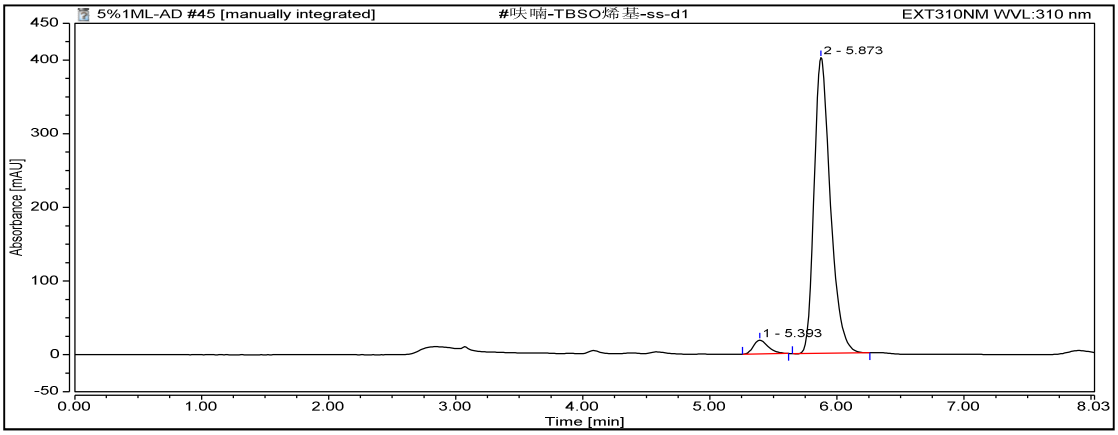

Integration Results					
No.	Peak Name	Retention Time<br>min	Area<br>mAU*min	Relative Area<br>%	Amount<br>n.a.
1		5.393	2.549	4.10	n.a.
2		5.873	59.573	95.90	n.a.
**Total:**			**62.122**	**100.00**	

Supplementary figure 213. HPLC chromatogram for compound **3bc**

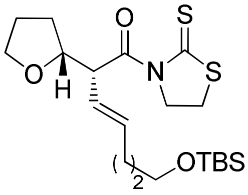

3bc'

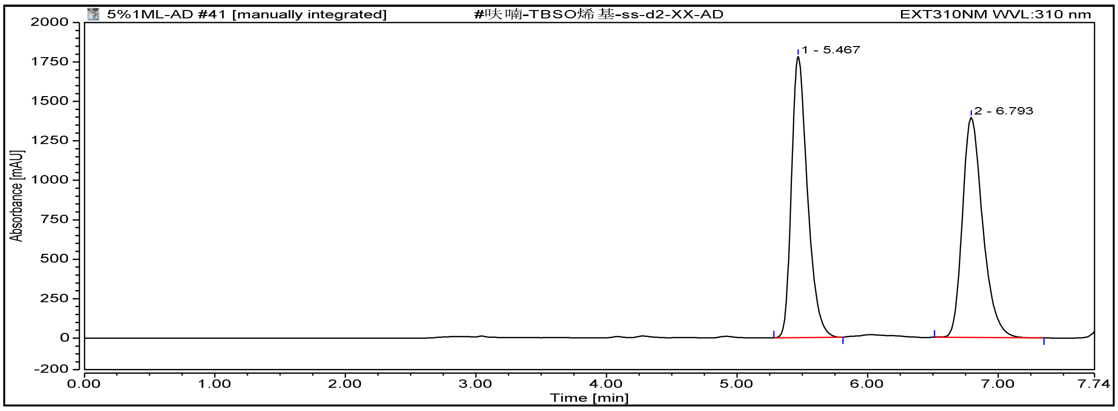

Integration Results					
No.	Peak Name	Retention Time<br>min	Area<br>mAU*min	Relative Area<br>%	Amount<br>n.a.
1		5.467	252.729	50.08	n.a.
2		6.793	251.907	49.92	n.a.
**Total:**			**504.637**	**100.00**	

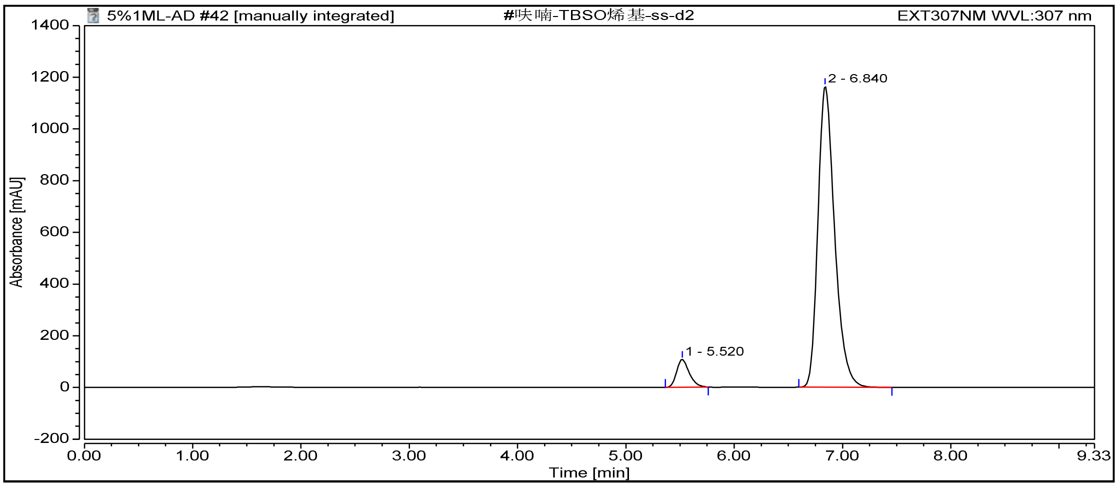

Integration Results					
No.	Peak Name	Retention Time<br>min	Area<br>mAU*min	Relative Area<br>%	Amount<br>n.a.
1		5.520	14.565	6.57	n.a.
2		6.840	207.295	93.43	n.a.
**Total:**			**221.861**	**100.00**	

Supplementary figure 214. HPLC chromatogram for compound **3bc'**

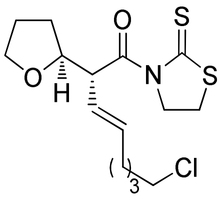

3bd

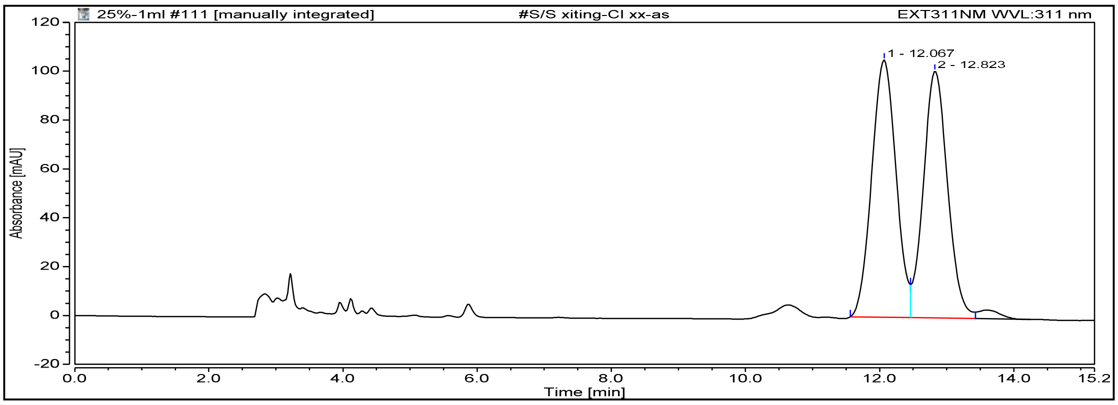

Integration Results					
No.	Peak Name	Retention Time<br>min	Area<br>mAU*min	Relative Area<br>%	Amount<br>n.a.
1		12.067	44.118	51.17	n.a.
2		12.823	42.093	48.83	n.a.
**Total:**			**86.211**	**100.00**	

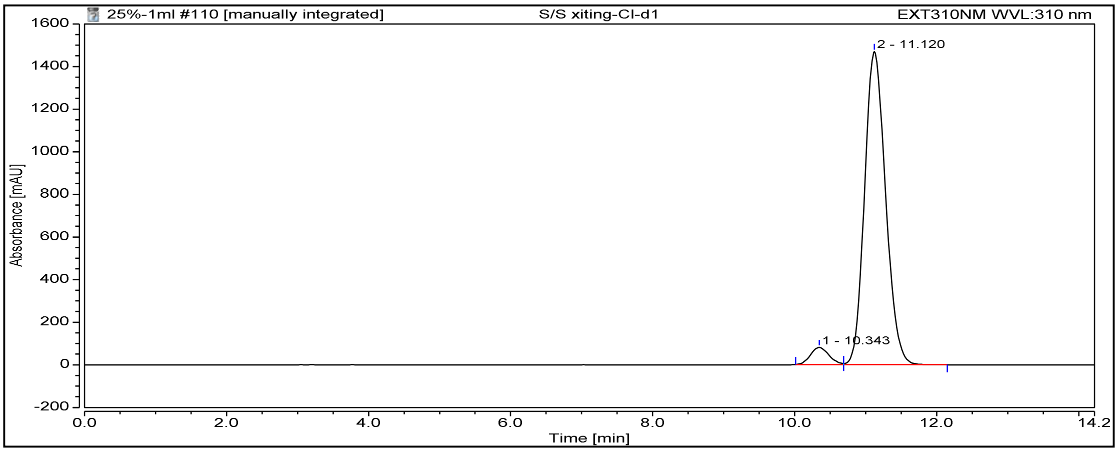

Integration Results					
No.	Peak Name	Retention Time<br>min	Area<br>mAU*min	Relative Area<br>%	Amount<br>n.a.
1		10.343	24.383	4.61	n.a.
2		11.120	504.805	95.39	n.a.
**Total:**			**529.188**	**100.00**	

Supplementary figure 215. HPLC chromatogram for compound **3bd**

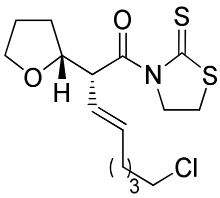

3bd’

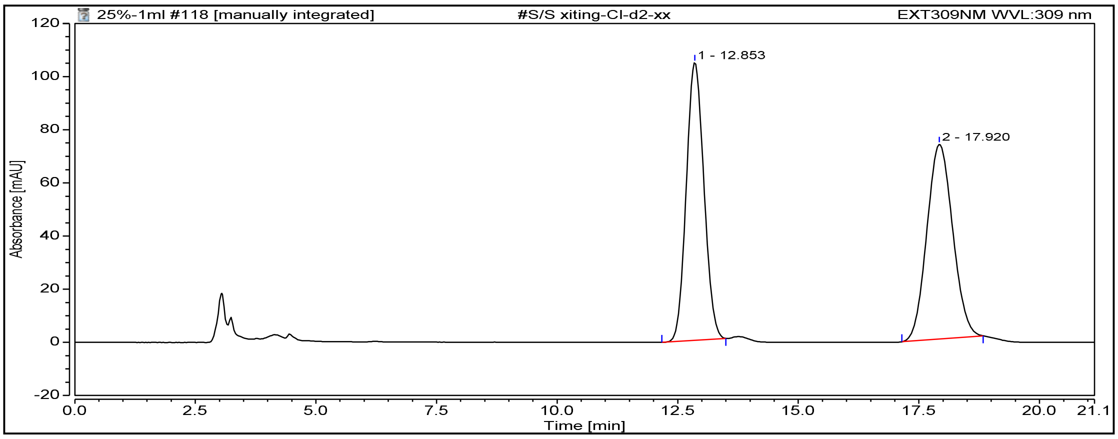

Integration Results					
No.	Peak Name	Retention Time<br>min	Area<br>mAU*min	Relative Area<br>%	Amount<br>n.a.
1		12.853	44.320	49.34	n.a.
2		17.920	45.503	50.66	n.a.
**Total:**			**89.823**	**100.00**	

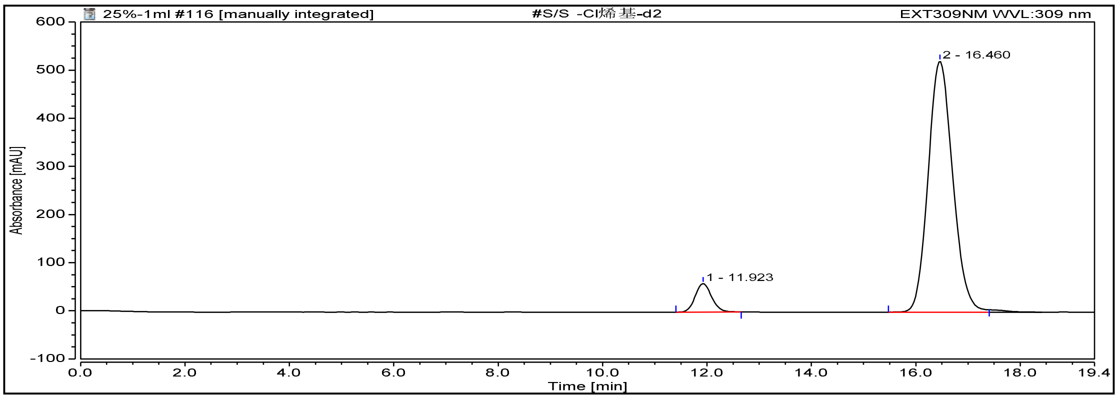

Integration Results					
No.	Peak Name	Retention Time<br>min	Area<br>mAU*min	Relative Area<br>%	Amount<br>n.a.
1		11.923	21.423	7.01	n.a.
2		16.460	284.270	92.99	n.a.
**Total:**			**305.693**	**100.00**	

Supplementary figure 216. HPLC chromatogram for compound **3bd’**

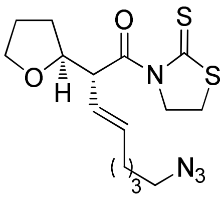

3be

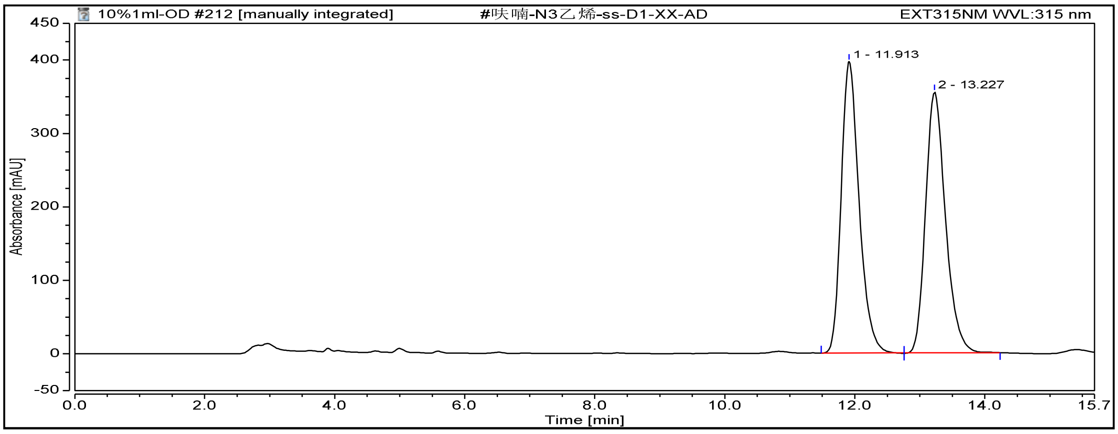

**Integration Results**					
No.	Peak Name	Retention Time<br>min	Area<br>mAU*min	Relative Area<br>%	Amount<br>n.a.
1		11.913	126.501	50.18	n.a.
2		13.227	125.600	49.82	n.a.
**Total:**			**252.100**	**100.00**	

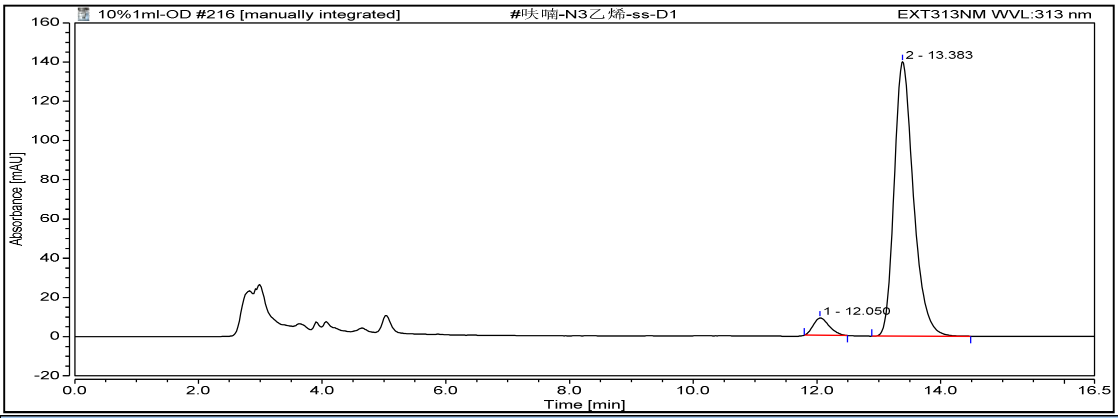

**Integration Results**					
No.	Peak Name	Retention Time<br>min	Area<br>mAU*min	Relative Area<br>%	Amount<br>n.a.
1		12.050	2.650	4.98	n.a.
2		13.383	50.612	95.02	n.a.
**Total:**			**53.262**	**100.00**	

Supplementary figure 217. HPLC chromatogram for compound **3be**

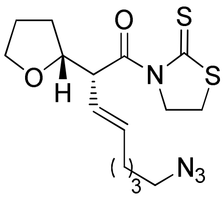

3be’

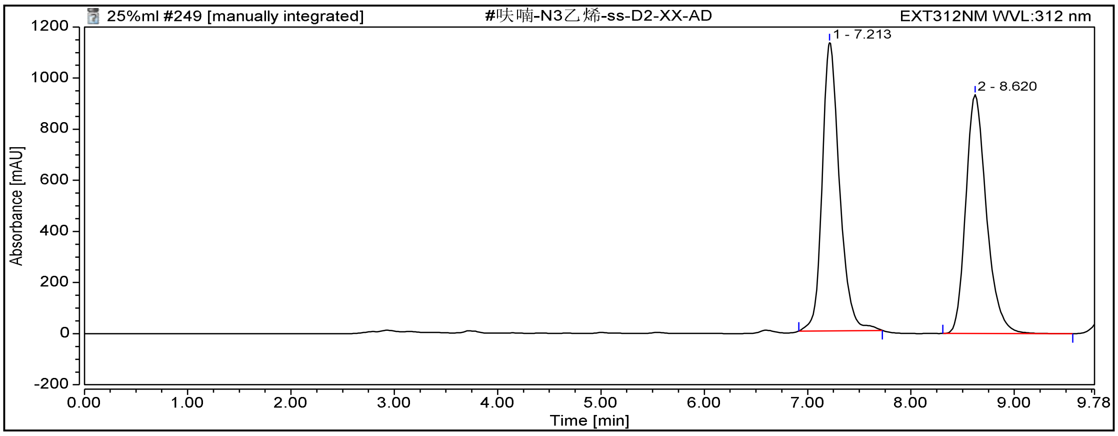

Integration Results					
No.	Peak Name	Retention Time<br>min	Area<br>mAU*min	Relative Area<br>%	Amount<br>n.a.
1		7.213	219.892	50.40	n.a.
2		8.620	216.391	49.60	n.a.
**Total:**			**436.283**	**100.00**	

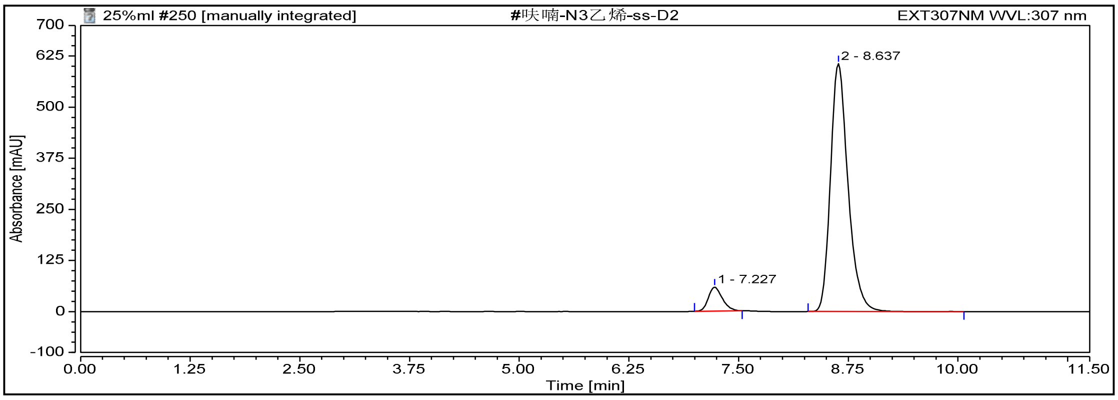

Integration Results					
No.	Peak Name	Retention Time<br>min	Area<br>mAU*min	Relative Area<br>%	Amount<br>n.a.
1		7.227	10.795	7.14	n.a.
2		8.637	140.469	92.86	n.a.
**Total:**			**151.264**	**100.00**	

Supplementary figure 218. HPLC chromatogram for compound **3be’**

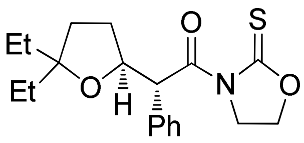

4a

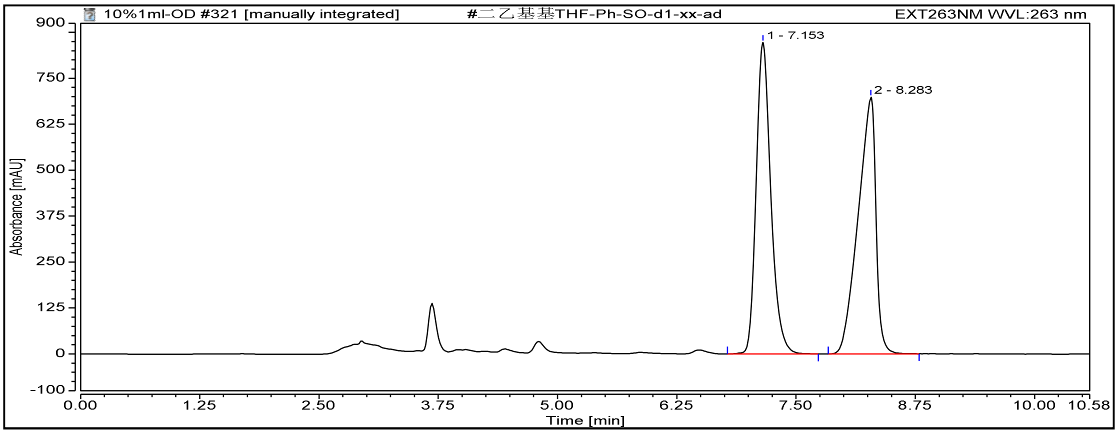

Integration Results					
No.	Peak Name	Retention Time<br>min	Area<br>mAU*min	Relative Area<br>%	Amount<br>n.a.
1		7.153	150.214	50.03	n.a.
2		8.283	150.011	49.97	n.a.
**Total:**			**300.226**	**100.00**	

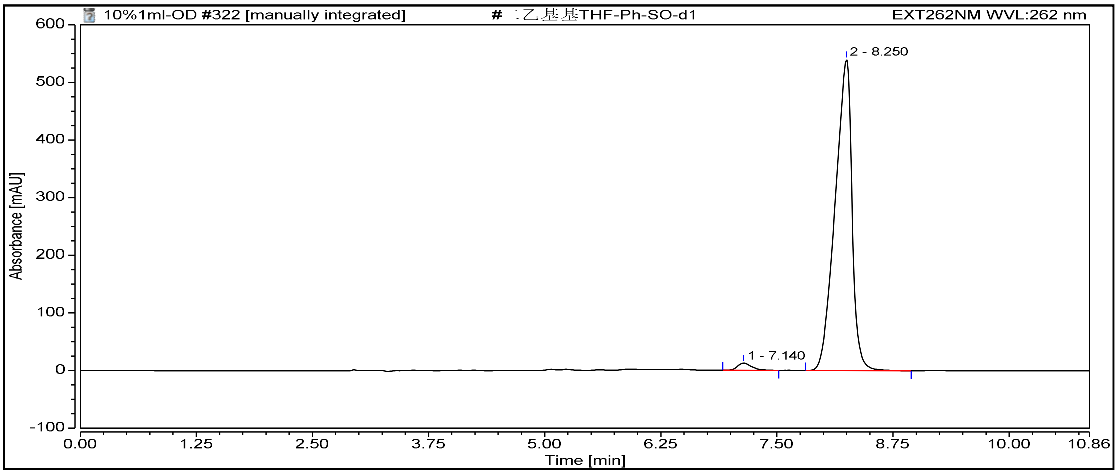

Integration Results					
No.	Peak Name	Retention Time<br>min	Area<br>mAU*min	Relative Area<br>%	Amount<br>n.a.
1		7.140	2.227	1.91	n.a.
2		8.250	114.204	98.09	n.a.
**Total:**			**116.431**	**100.00**	

Supplementary figure 219. HPLC chromatogram for compound **4a**

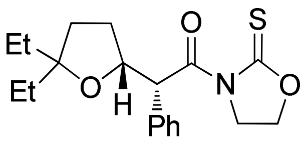

4a'

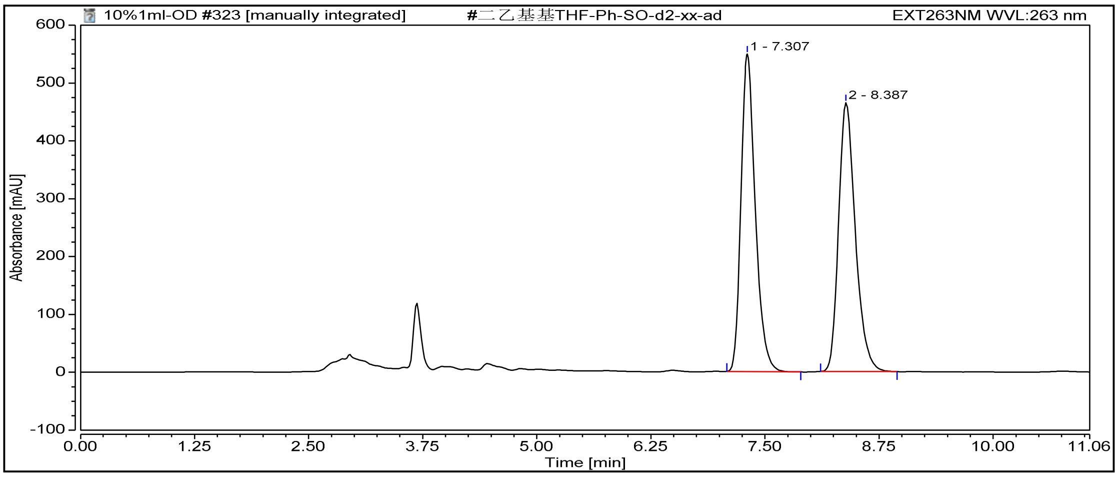

Integration Results					
No.	Peak Name	Retention Time min	Area mAU*min	Relative Area %	Amount n.a.
1		7.307	99.243	50.06	n.a.
2		8.387	98.991	49.94	n.a.
**Total:**			**198.233**	**100.00**	

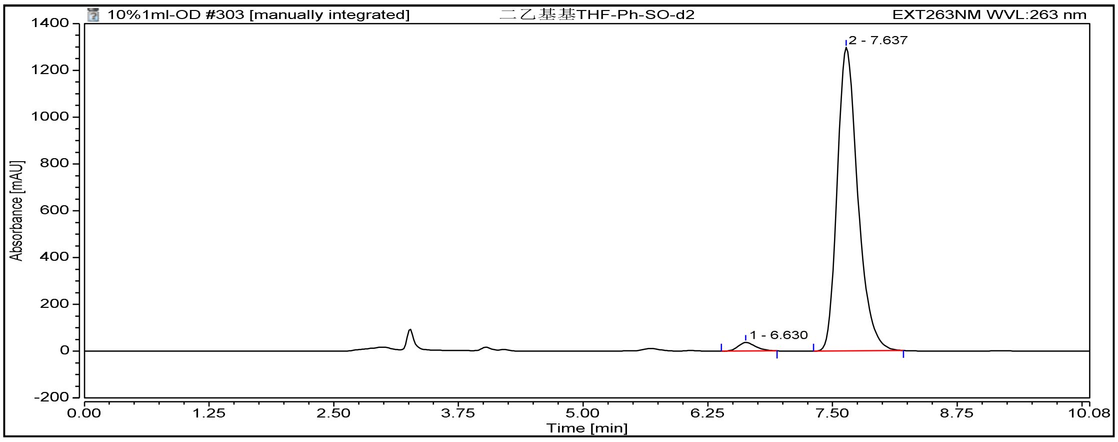

Integration Results					
No.	Peak Name	Retention Time min	Area mAU*min	Relative Area %	Amount n.a.
1		6.630	7.494	2.35	n.a.
2		7.637	312.004	97.65	n.a.
**Total:**			**319.498**	**100.00**	

Supplementary figure 220. HPLC chromatogram for compound **4a'**

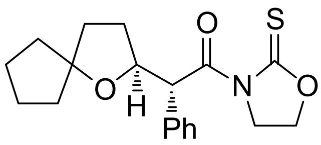

4b

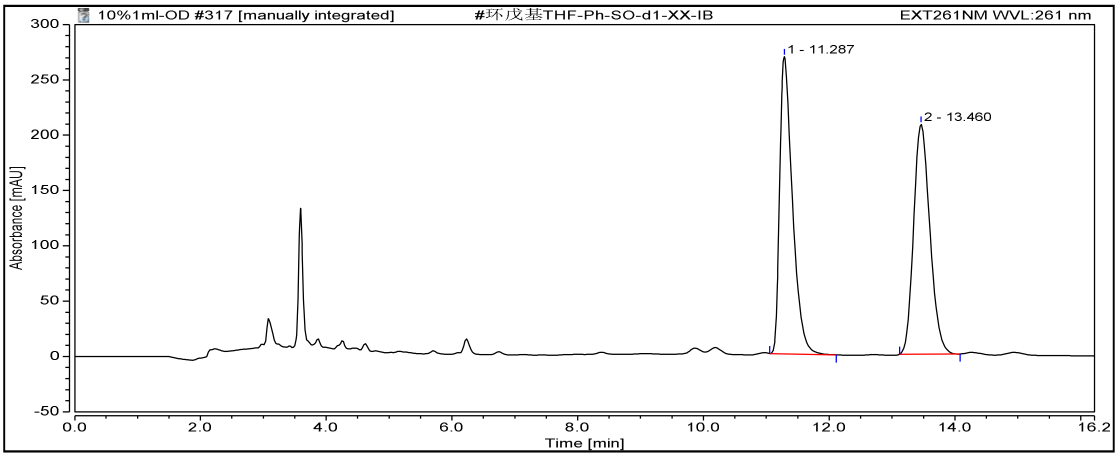

Integration Results					
No.	Peak Name	Retention Time min	Area mAU*min	Relative Area %	Amount n.a.
1		11.287	63.078	50.25	n.a.
2		13.460	62.448	49.75	n.a.
**Total:**			**125.526**	**100.00**	

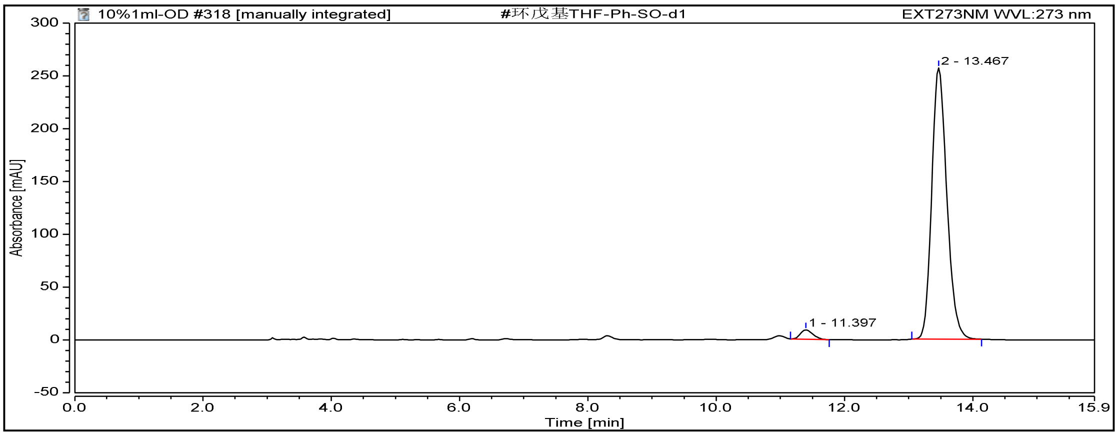

Integration Results					
No.	Peak Name	Retention Time min	Area mAU*min	Relative Area %	Amount n.a.
1		11.397	2.003	2.77	n.a.
2		13.467	70.201	97.23	n.a.
**Total:**			**72.203**	**100.00**	

Supplementary figure 221. HPLC chromatogram for compound **4b**

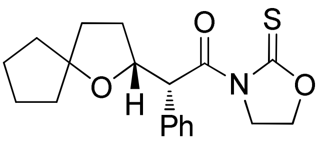

4b'

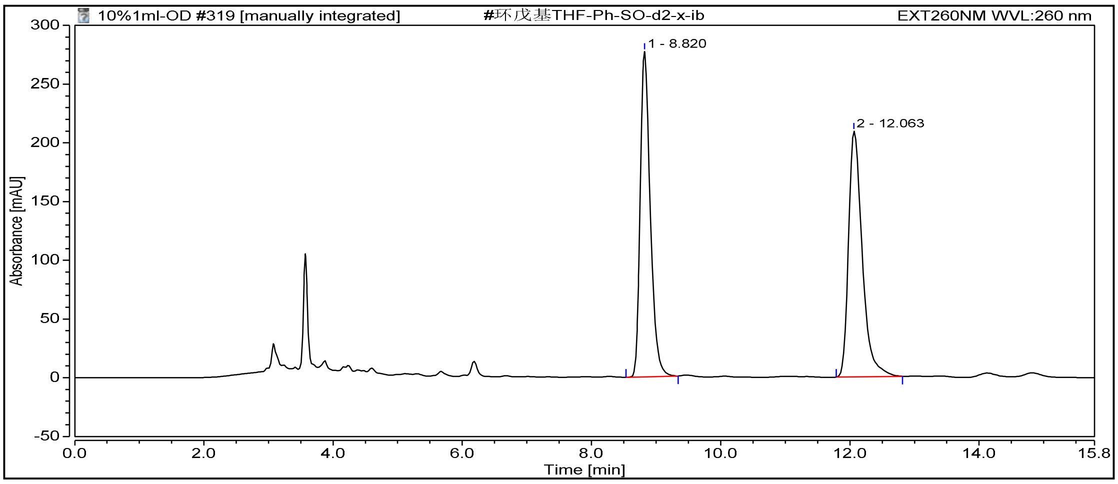

Integration Results					
No.	Peak Name	Retention Time<br>min	Area<br>mAU*min	Relative Area<br>%	Amount<br>n.a.
1		8.820	50.046	49.27	n.a.
2		12.063	51.520	50.73	n.a.
**Total:**			**101.566**	**100.00**	

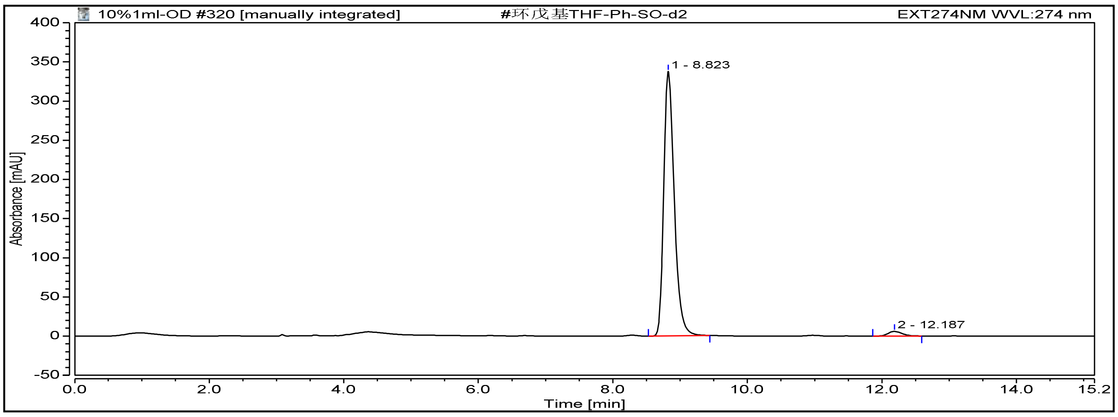

Integration Results					
No.	Peak Name	Retention Time<br>min	Area<br>mAU*min	Relative Area<br>%	Amount<br>n.a.
1		8.823	61.061	97.66	n.a.
2		12.187	1.463	2.34	n.a.
**Total:**			**62.524**	**100.00**	

Supplementary figure 222. HPLC chromatogram for compound **4b'**

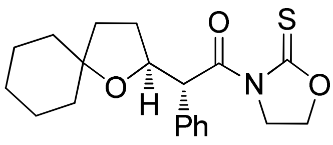

4c

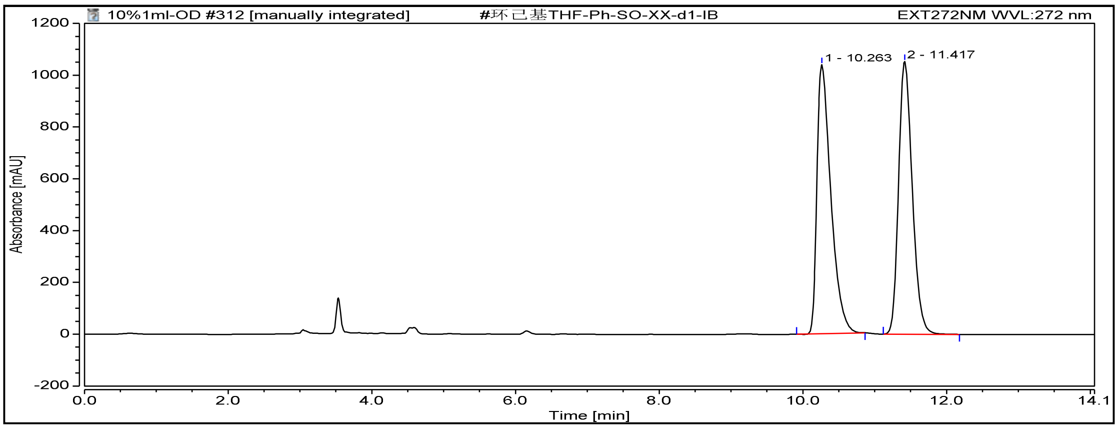

Integration Results					
No.	Peak Name	Retention Time<br>min	Area<br>mAU*min	Relative Area<br>%	Amount<br>n.a.
1		10.263	232.725	50.03	n.a.
2		11.417	232.484	49.97	n.a.
**Total:**			**465.209**	**100.00**	

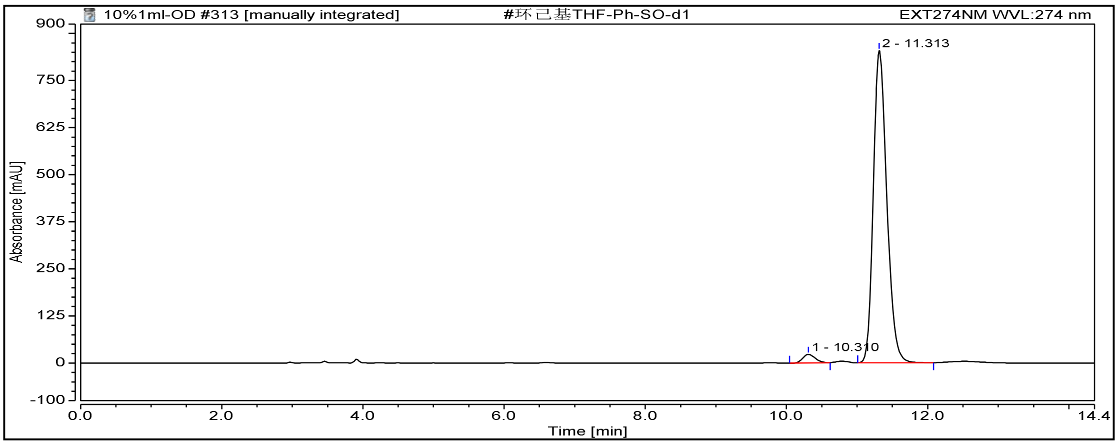

Integration Results					
No.	Peak Name	Retention Time<br>min	Area<br>mAU*min	Relative Area<br>%	Amount<br>n.a.
1		10.310	4.553	2.42	n.a.
2		11.313	183.293	97.58	n.a.
**Total:**			**187.846**	**100.00**	

Supplementary figure 223. HPLC chromatogram for compound **4c**

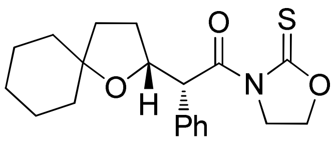

4c'

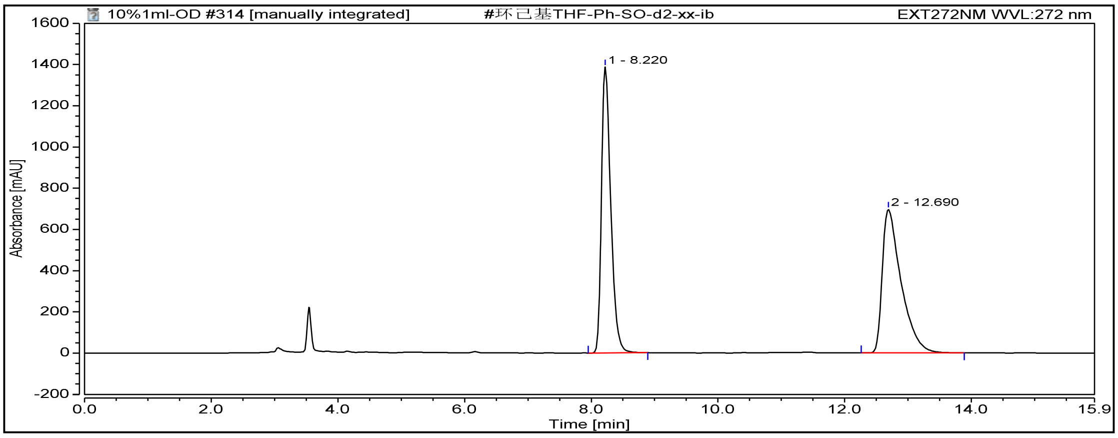

Integration Results					
No.	Peak Name	Retention Time<br>min	Area<br>mAU*min	Relative Area<br>%	Amount<br>n.a.
1		8.220	235.710	49.64	n.a.
2		12.690	239.124	50.36	n.a.
**Total:**			**474.834**	**100.00**	

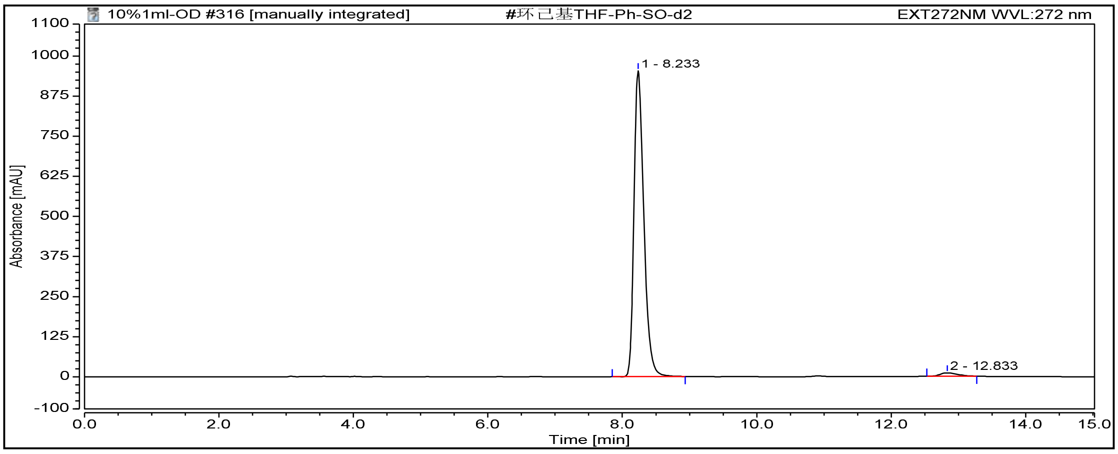

Integration Results					
No.	Peak Name	Retention Time<br>min	Area<br>mAU*min	Relative Area<br>%	Amount<br>n.a.
1		8.233	160.934	98.06	n.a.
2		12.833	3.185	1.94	n.a.
**Total:**			**164.119**	**100.00**	

Supplementary figure 224. HPLC chromatogram for compound **4c'**

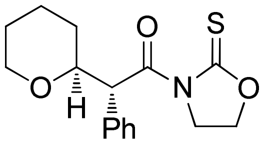

4d

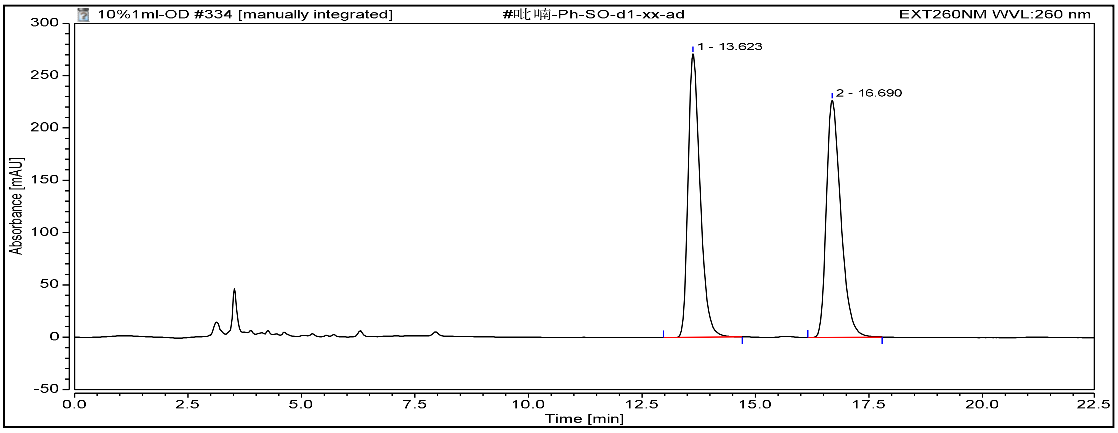

Integration Results					
No.	Peak Name	Retention Time<br>min	Area<br>mAU*min	Relative Area<br>%	Amount<br>n.a.
1		13.623	83.493	49.95	n.a.
2		16.690	83.652	50.05	n.a.
**Total:**			**167.145**	**100.00**	

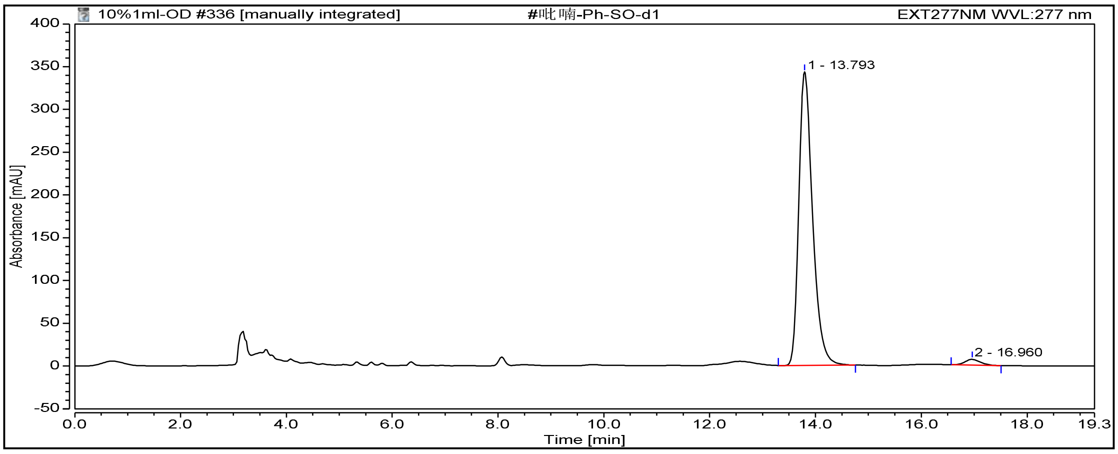

Integration Results					
No.	Peak Name	Retention Time<br>min	Area<br>mAU*min	Relative Area<br>%	Amount<br>n.a.
1		13.793	104.246	97.81	n.a.
2		16.960	2.329	2.19	n.a.
**Total:**			**106.575**	**100.00**	

Supplementary figure 225. HPLC chromatogram for compound **4d**

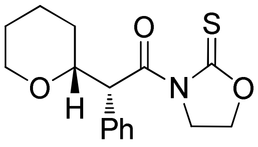

4d’

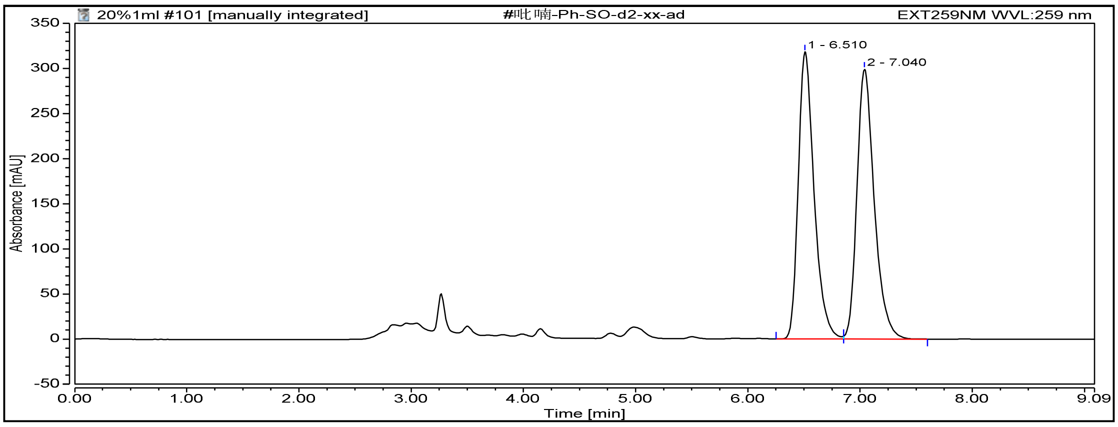

Integration Results					
No.	Peak Name	Retention Time<br>min	Area<br>mAU*min	Relative Area<br>%	Amount<br>n.a.
1		6.510	52.302	49.35	n.a.
2		7.040	53.677	50.65	n.a.
**Total:**			**105.979**	**100.00**	

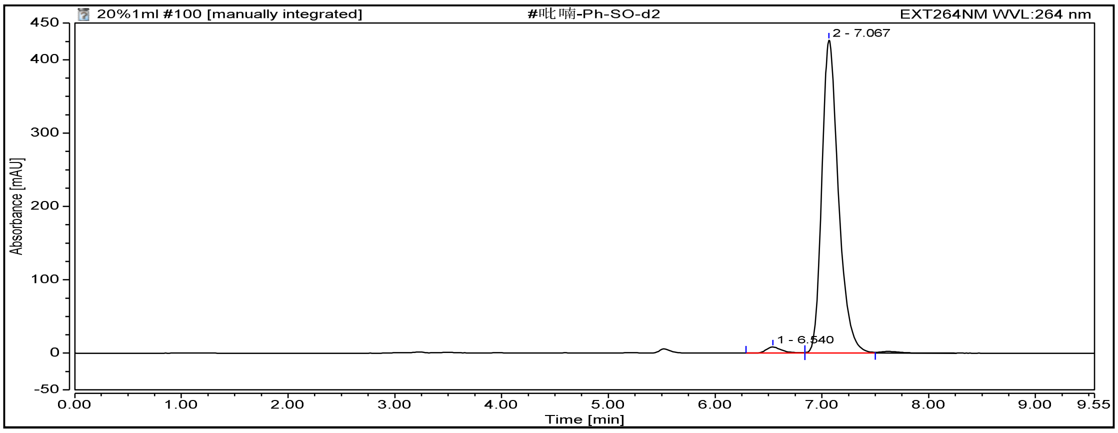

Integration Results					
No.	Peak Name	Retention Time<br>min	Area<br>mAU*min	Relative Area<br>%	Amount<br>n.a.
1		6.540	1.437	1.83	n.a.
2		7.067	77.069	98.17	n.a.
**Total:**			**78.506**	**100.00**	

Supplementary figure 226. HPLC chromatogram for compound **4d’**

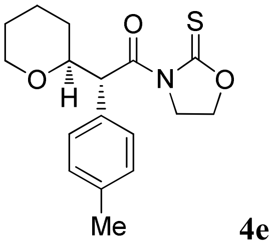

4e

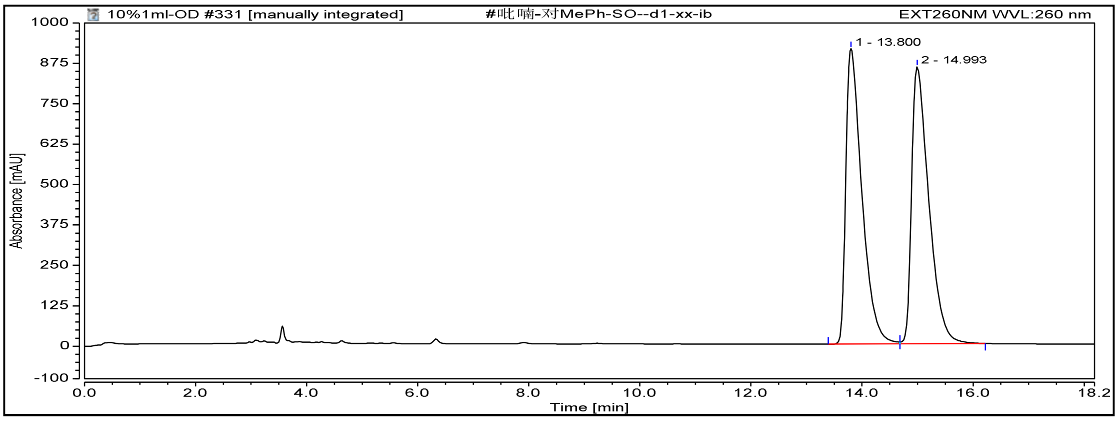

Integration Results					
No.	Peak Name	Retention Time<br>min	Area<br>mAU*min	Relative Area<br>%	Amount<br>n.a.
1		13.800	305.375	49.86	n.a.
2		14.993	307.098	50.14	n.a.
**Total:**			**612.473**	**100.00**	

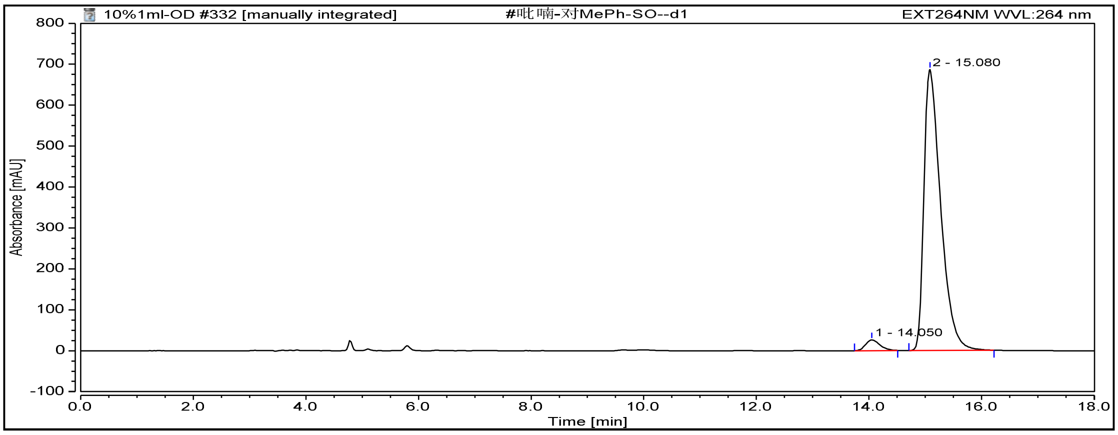

Integration Results					
No.	Peak Name	Retention Time<br>min	Area<br>mAU*min	Relative Area<br>%	Amount<br>n.a.
1		14.050	7.665	3.14	n.a.
2		15.080	236.644	96.86	n.a.
**Total:**			**244.310**	**100.00**	

Supplementary figure 227. HPLC chromatogram for compound **4e**

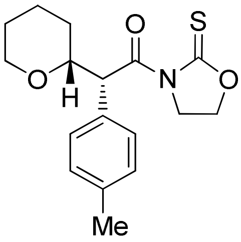

4e'

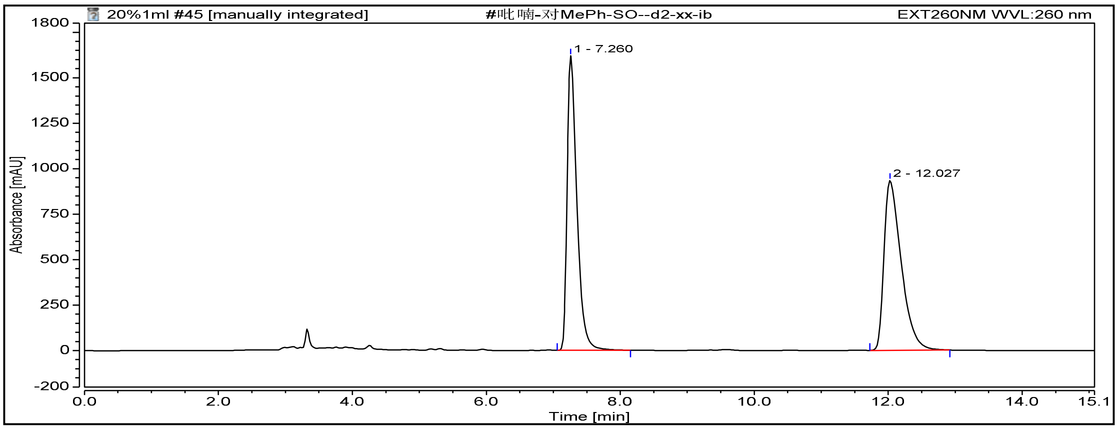

Integration Results					
No.	Peak Name	Retention Time<br>min	Area<br>mAU*min	Relative Area<br>%	Amount<br>n.a.
1		7.260	274.396	49.69	n.a.
2		12.027	277.818	50.31	n.a.
**Total:**			**552.214**	**100.00**	

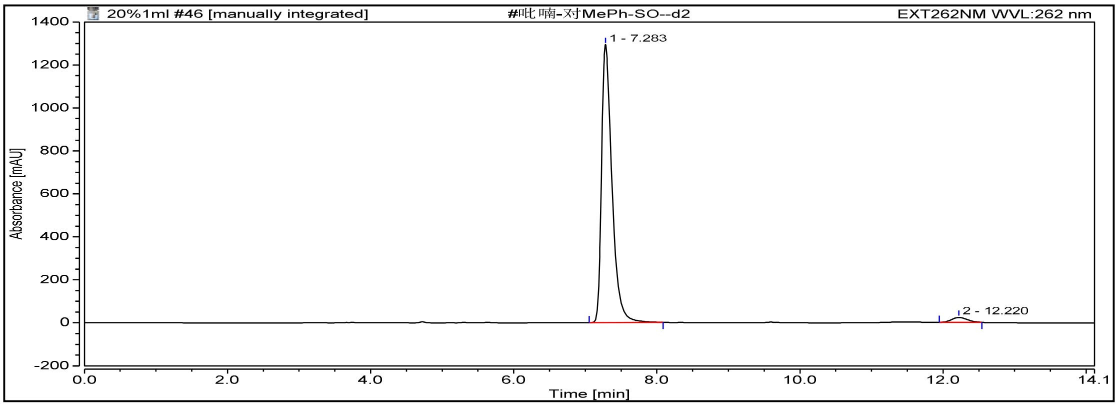

Integration Results					
No.	Peak Name	Retention Time<br>min	Area<br>mAU*min	Relative Area<br>%	Amount<br>n.a.
1		7.283	218.000	97.40	n.a.
2		12.220	5.825	2.60	n.a.
**Total:**			**223.825**	**100.00**	

Supplementary figure 228. HPLC chromatogram for compound **4e'**

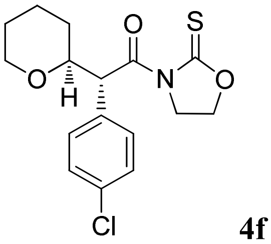

4f

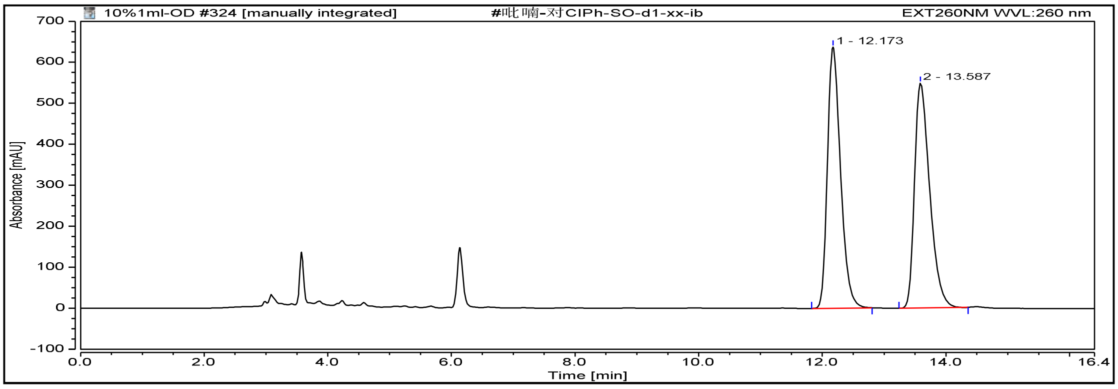

Integration Results					
No.	Peak Name	Retention Time<br>min	Area<br>mAU*min	Relative Area<br>%	Amount<br>n.a.
1		12.173	156.826	50.09	n.a.
2		13.587	156.259	49.91	n.a.
**Total:**			**313.085**	**100.00**	

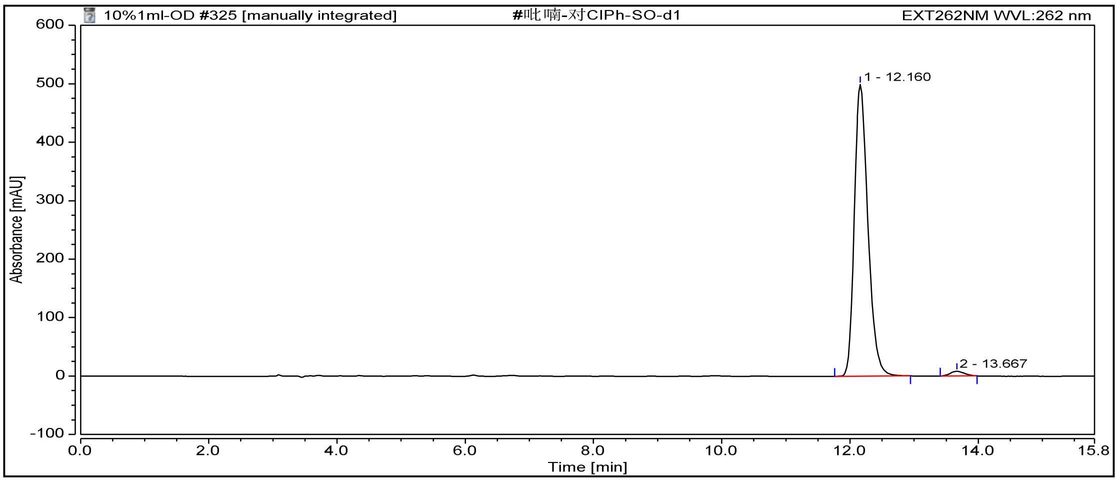

Integration Results					
No.	Peak Name	Retention Time<br>min	Area<br>mAU*min	Relative Area<br>%	Amount<br>n.a.
1		12.160	122.491	98.35	n.a.
2		13.667	2.055	1.65	n.a.
**Total:**			**124.546**	**100.00**	

Supplementary figure 229. HPLC chromatogram for compound **4f**

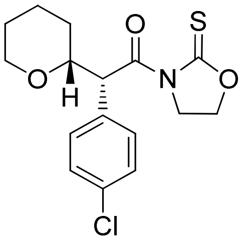

4f’

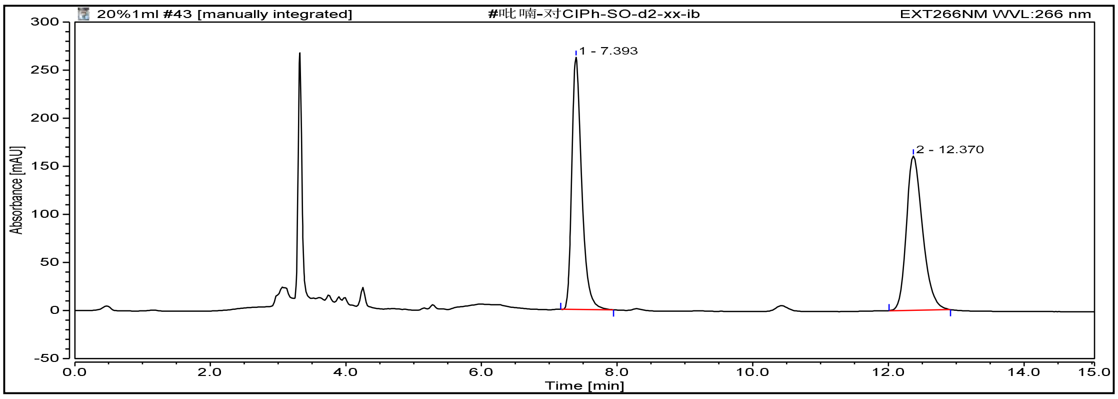

Integration Results					
No.	Peak Name	Retention Time min	Area mAU*min	Relative Area %	Amount n.a.
1		7.393	44.243	49.81	n.a.
2		12.370	44.576	50.19	n.a.
**Total:**			**88.819**	**100.00**	

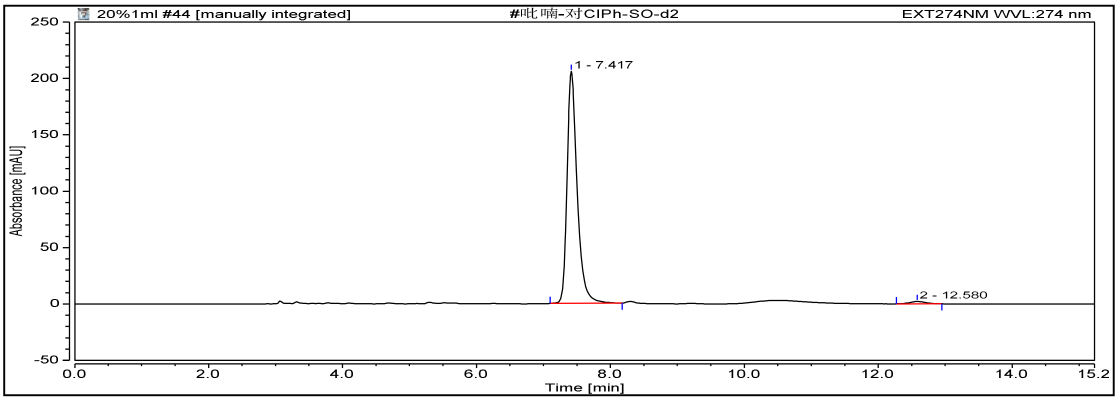

Integration Results					
No.	Peak Name	Retention Time min	Area mAU*min	Relative Area %	Amount n.a.
1		7.417	36.003	98.40	n.a.
2		12.580	0.584	1.60	n.a.
**Total:**			**36.587**	**100.00**	

Supplementary figure 230. HPLC chromatogram for compound **4f’**

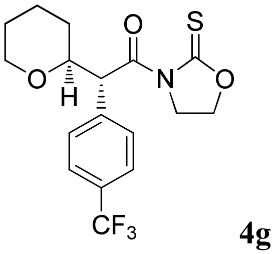

4g

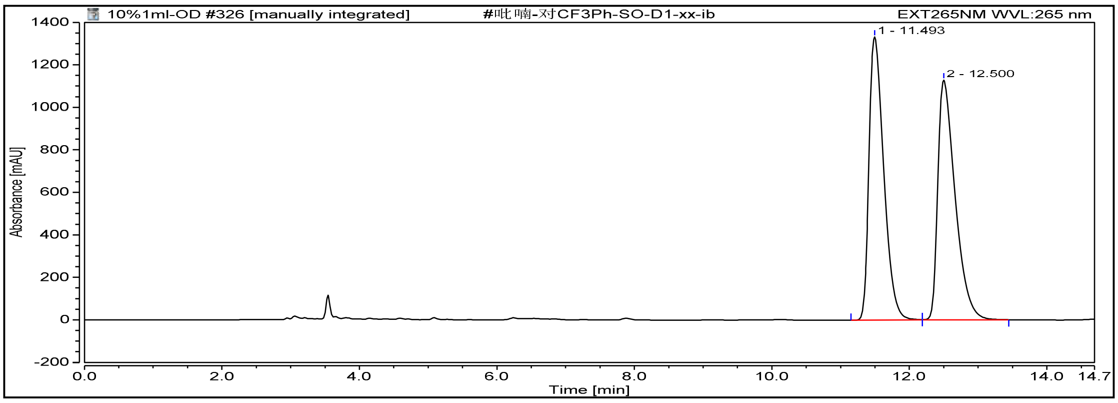

Integration Results					
No.	Peak Name	Retention Time<br>min	Area<br>mAU*min	Relative Area<br>%	Amount<br>n.a.
1		11.493	327.516	49.79	n.a.
2		12.500	330.258	50.21	n.a.
**Total:**			**657.774**	**100.00**	

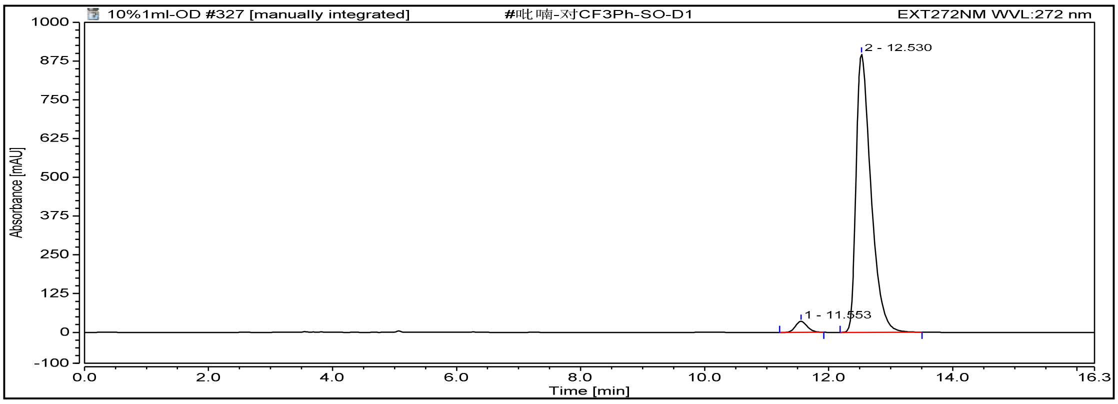

Integration Results					
No.	Peak Name	Retention Time<br>min	Area<br>mAU*min	Relative Area<br>%	Amount<br>n.a.
1		11.553	8.029	3.11	n.a.
2		12.530	250.568	96.89	n.a.
**Total:**			**258.598**	**100.00**	

Supplementary figure 231. HPLC chromatogram for compound **4g**

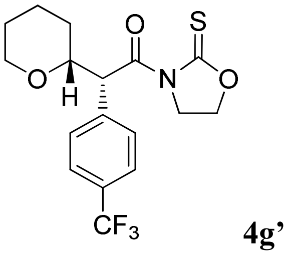

4g'

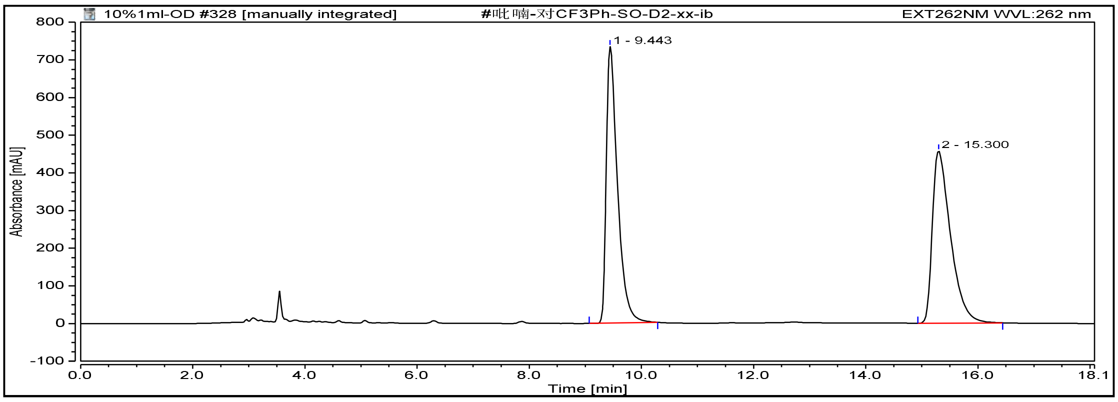

Integration Results					
No.	Peak Name	Retention Time<br>min	Area<br>mAU*min	Relative Area<br>%	Amount<br>n.a.
1		9.443	169.996	49.76	n.a.
2		15.300	171.653	50.24	n.a.
**Total:**			**341.649**	**100.00**	

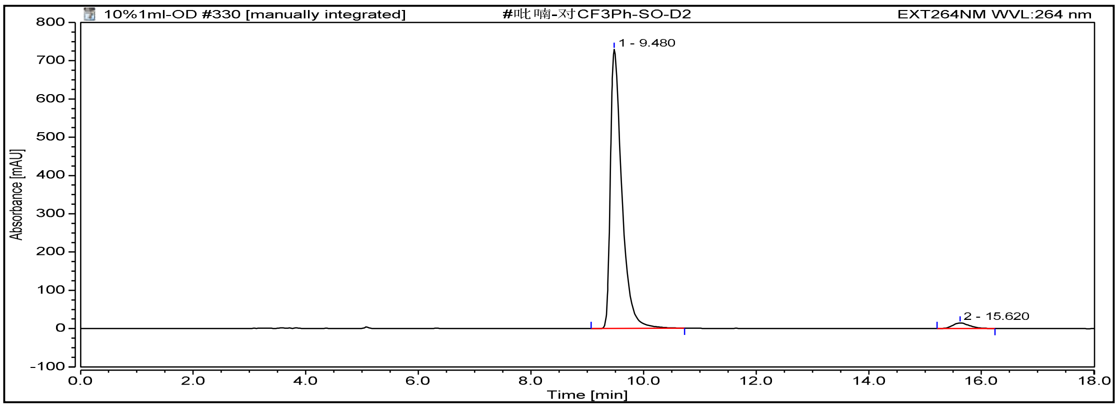

Integration Results					
No.	Peak Name	Retention Time<br>min	Area<br>mAU*min	Relative Area<br>%	Amount<br>n.a.
1		9.480	172.286	97.10	n.a.
2		15.620	5.141	2.90	n.a.
**Total:**			**177.427**	**100.00**	

Supplementary figure 232. HPLC chromatogram for compound **4g'**

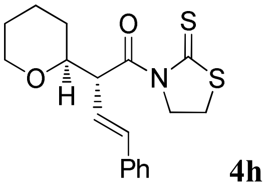

4h

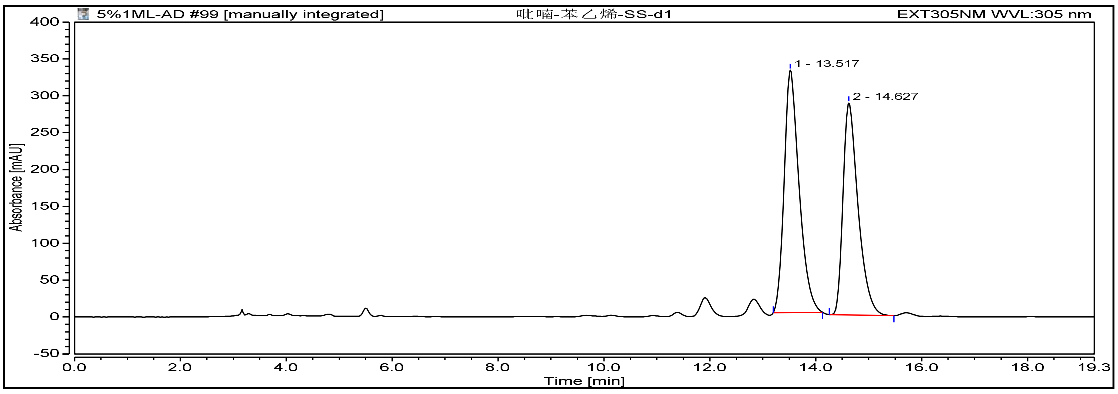

Integration Results					
No.	Peak Name	Retention Time min	Area mAU*min	Relative Area %	Amount n.a.
1		13.517	108.849	53.53	n.a.
2		14.627	94.490	46.47	n.a.
**Total:**			**203.338**	**100.00**	

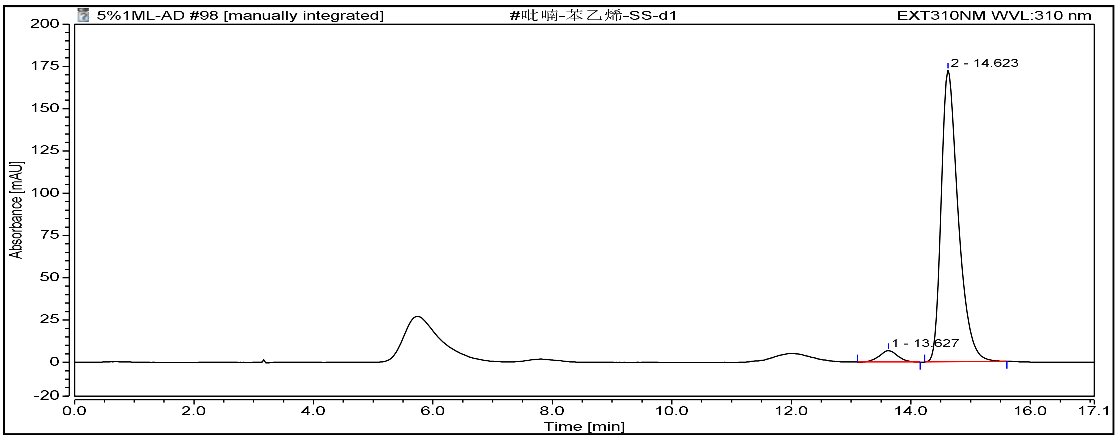

Integration Results					
No.	Peak Name	Retention Time min	Area mAU*min	Relative Area %	Amount n.a.
1		13.627	2.401	4.06	n.a.
2		14.623	56.683	95.94	n.a.
**Total:**			**59.084**	**100.00**	

Supplementary figure 233. HPLC chromatogram for compound **4h**

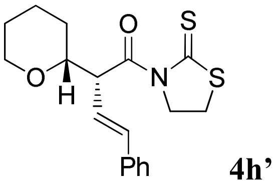

4h'

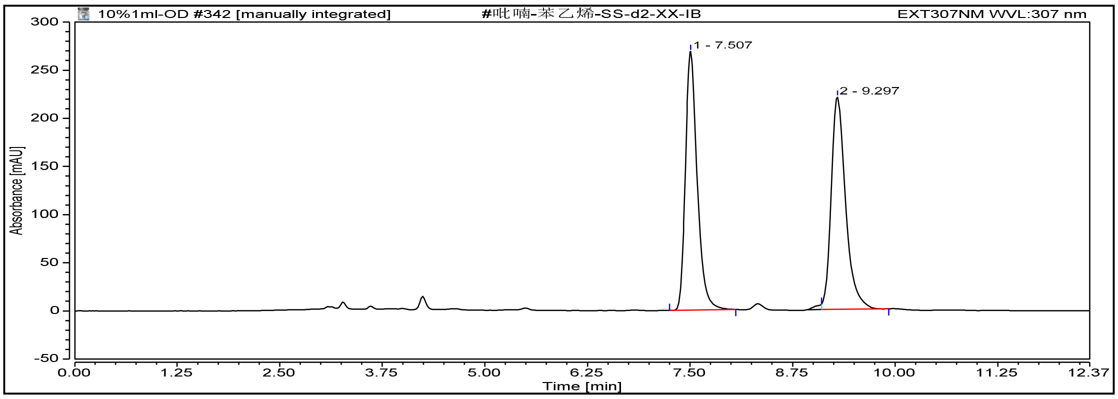

Integration Results					
No.	Peak Name	Retention Time<br>min	Area<br>mAU*min	Relative Area<br>%	Amount<br>n.a.
1		7.507	44.783	49.85	n.a.
2		9.297	45.043	50.15	n.a.
**Total:**			**89.826**	**100.00**	

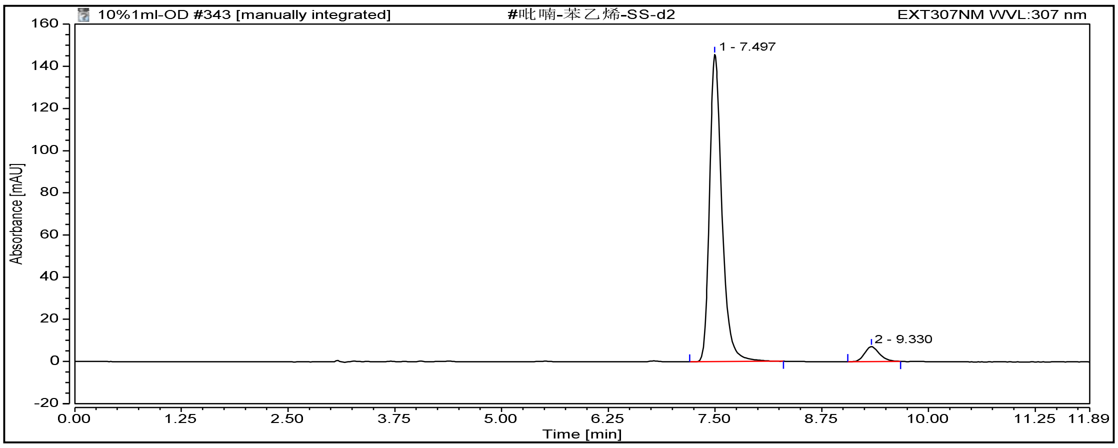

Integration Results					
No.	Peak Name	Retention Time<br>min	Area<br>mAU*min	Relative Area<br>%	Amount<br>n.a.
1		7.497	24.547	94.45	n.a.
2		9.330	1.442	5.55	n.a.
**Total:**			**25.989**	**100.00**	

Supplementary figure 234. HPLC chromatogram for compound **4h'**

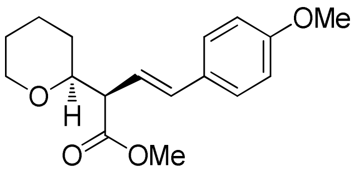

4i-a

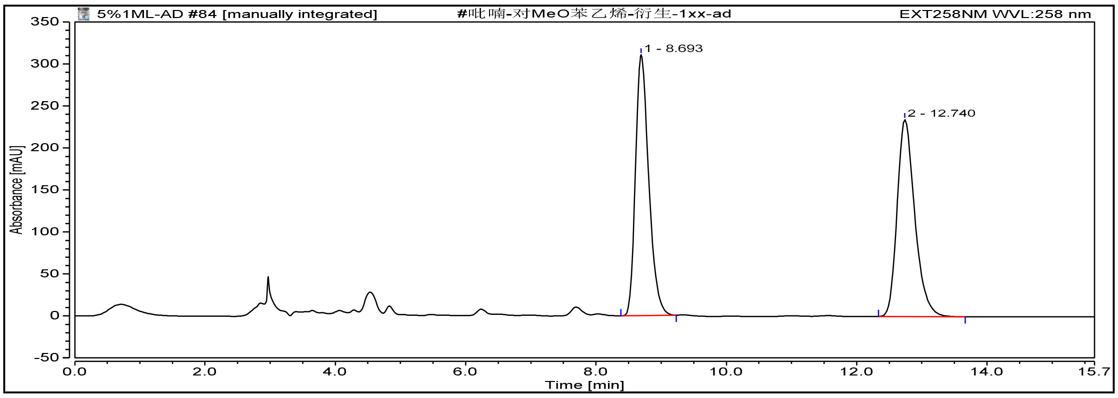

Integration Results					
No.	Peak Name	Retention Time min	Area mAU*min	Relative Area %	Amount n.a.
1		8.693	73.422	49.89	n.a.
2		12.740	73.749	50.11	n.a.
**Total:**			**147.172**	**100.00**	

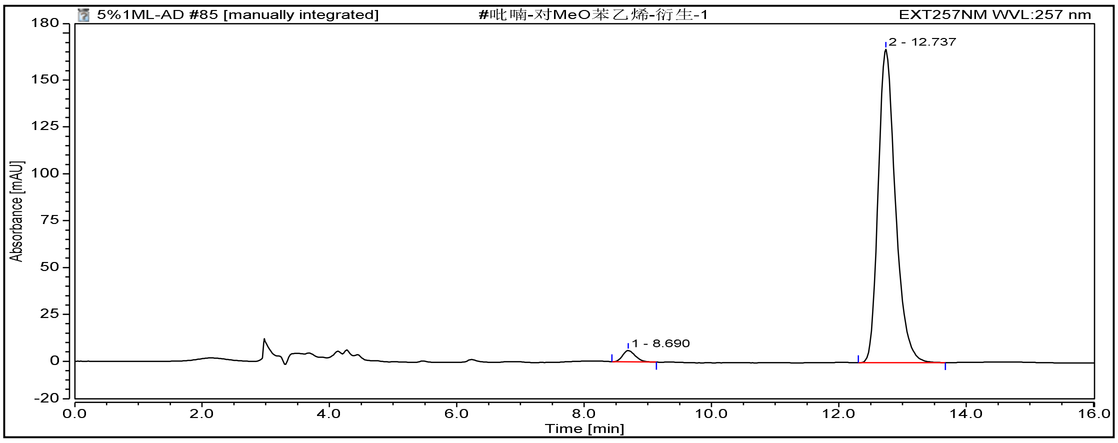

Integration Results					
No.	Peak Name	Retention Time min	Area mAU*min	Relative Area %	Amount n.a.
1		8.690	1.389	2.58	n.a.
2		12.737	52.482	97.42	n.a.
**Total:**			**53.870**	**100.00**	

Supplementary figure 235. HPLC chromatogram for compound **4i-a**

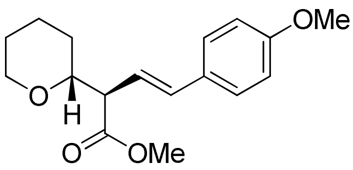

4i’-a

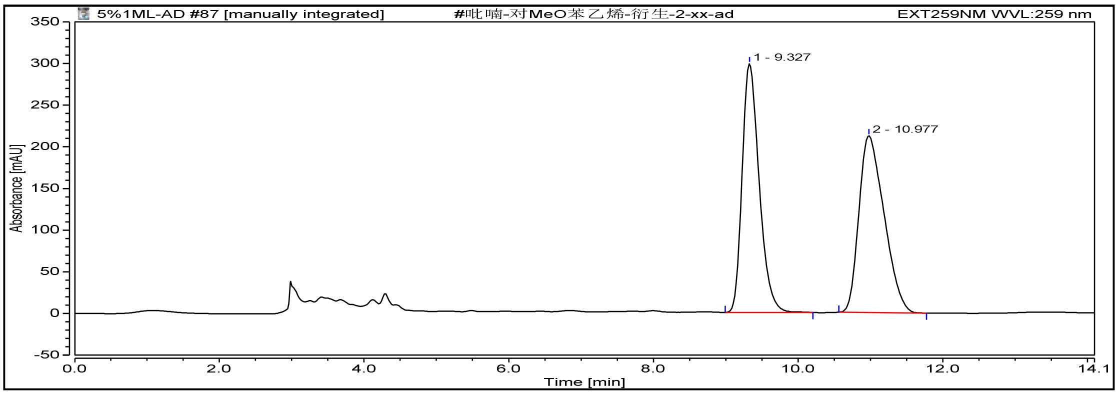

Integration Results					
No.	Peak Name	Retention Time<br>min	Area<br>mAU*min	Relative Area<br>%	Amount<br>n.a.
1		9.327	81.460	48.81	n.a.
2		10.977	85.432	51.19	n.a.
**Total:**			**166.891**	**100.00**	

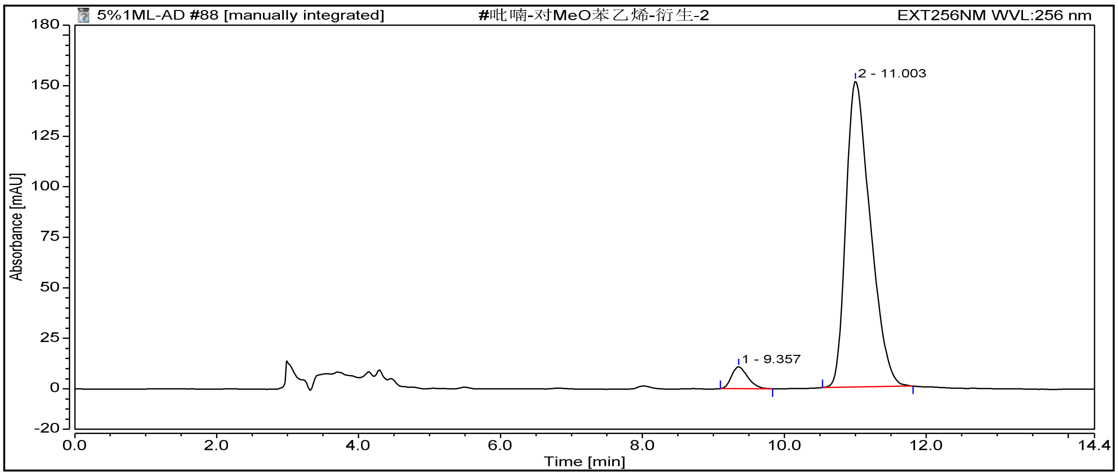

Integration Results					
No.	Peak Name	Retention Time<br>min	Area<br>mAU*min	Relative Area<br>%	Amount<br>n.a.
1		9.357	2.878	4.57	n.a.
2		11.003	60.151	95.43	n.a.
**Total:**			**63.029**	**100.00**	

Supplementary figure 236. HPLC chromatogram for compound **4i’-a**

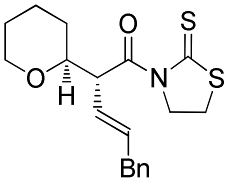

4j

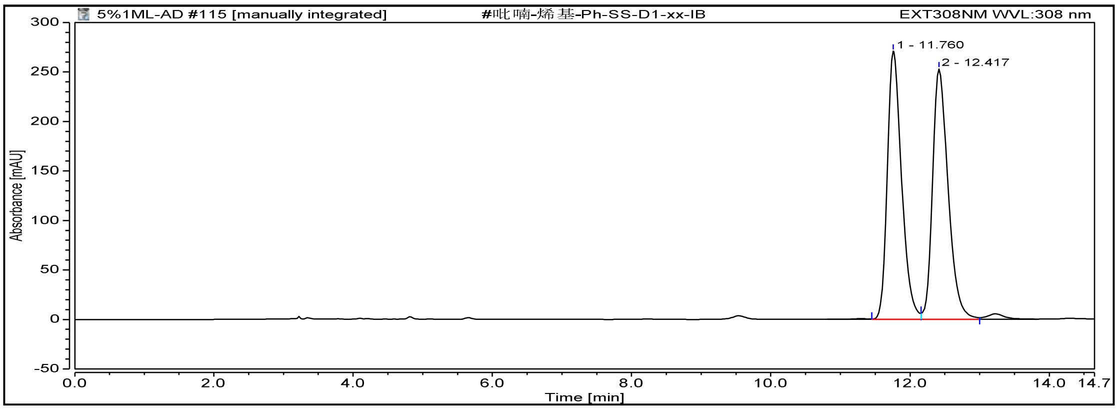

Integration Results					
No.	Peak Name	Retention Time<br>min	Area<br>mAU*min	Relative Area<br>%	Amount<br>n.a.
1		11.760	64.653	49.60	n.a.
2		12.417	65.696	50.40	n.a.
**Total:**			**130.349**	**100.00**	

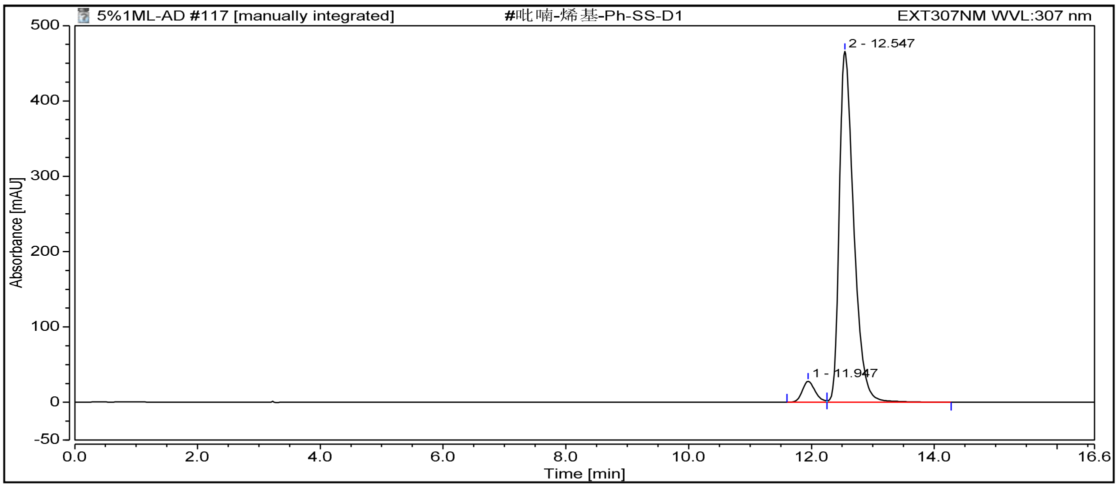

Integration Results					
No.	Peak Name	Retention Time<br>min	Area<br>mAU*min	Relative Area<br>%	Amount<br>n.a.
1		11.947	6.788	5.08	n.a.
2		12.547	126.778	94.92	n.a.
**Total:**			**133.567**	**100.00**	

Supplementary figure 237. HPLC chromatogram for compound **4j**

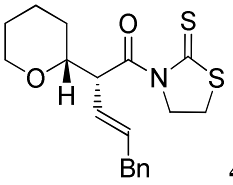

4j’

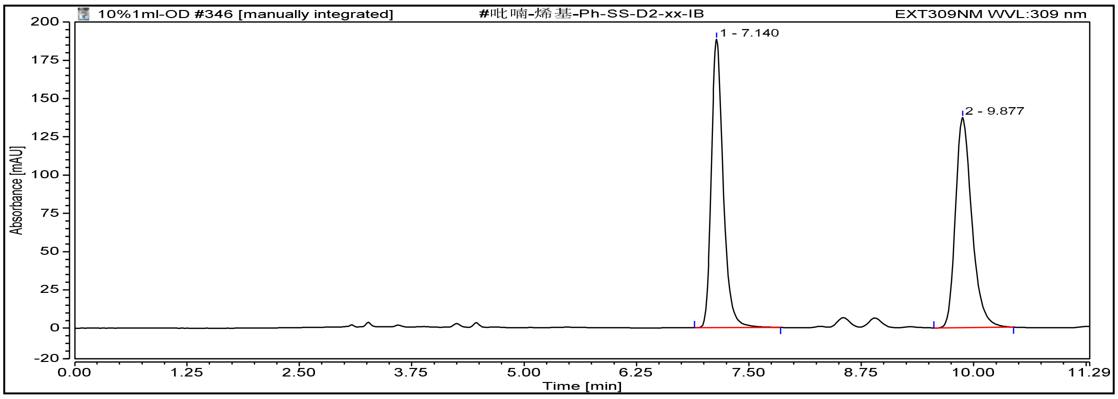

Integration Results					
No.	Peak Name	Retention Time<br>min	Area<br>mAU*min	Relative Area<br>%	Amount<br>n.a.
1		7.140	28.799	49.77	n.a.
2		9.877	29.061	50.23	n.a.
**Total:**			**57.859**	**100.00**	

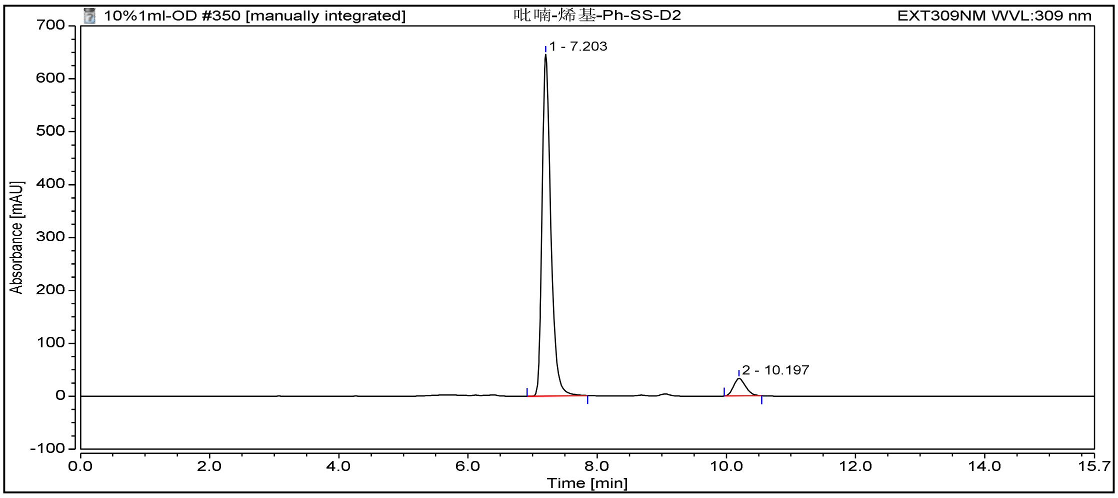

Integration Results					
No.	Peak Name	Retention Time<br>min	Area<br>mAU*min	Relative Area<br>%	Amount<br>n.a.
1		7.203	101.674	93.42	n.a.
2		10.197	7.161	6.58	n.a.
**Total:**			**108.835**	**100.00**	

Supplementary figure 238. HPLC chromatogram for compound **4j’**

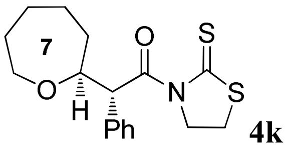

4k

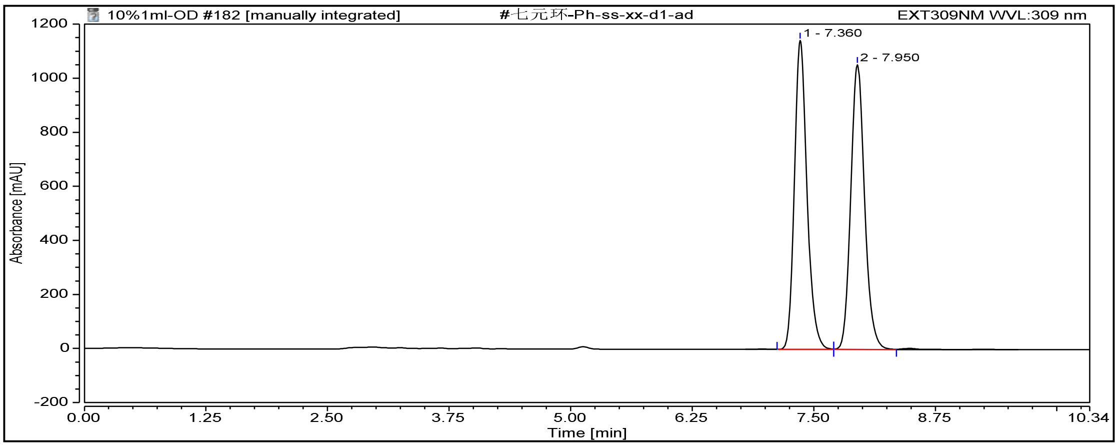

Integration Results					
No.	Peak Name	Retention Time min	Area mAU*min	Relative Area %	Amount n.a.
1		7.360	172.677	49.95	n.a.
2		7.950	172.998	50.05	n.a.
**Total:**			**345.675**	**100.00**	

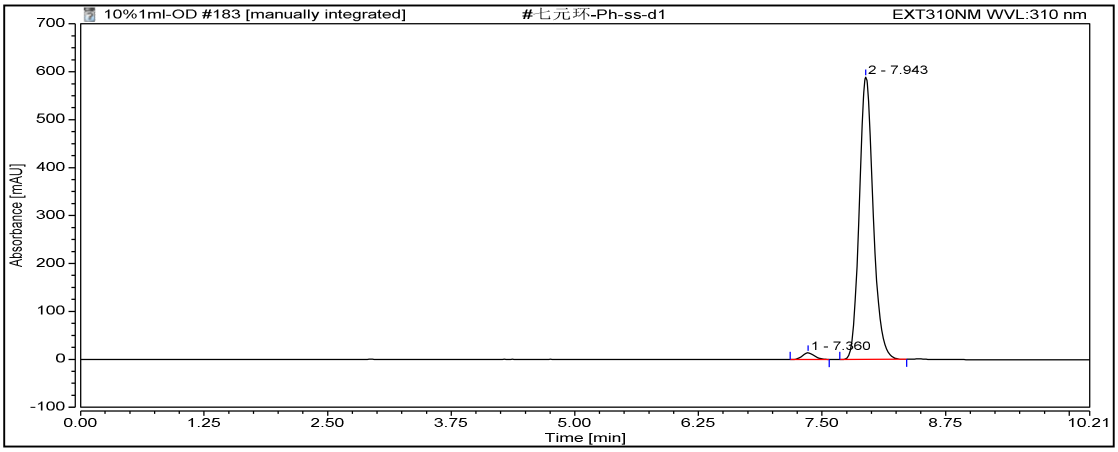

Integration Results					
No.	Peak Name	Retention Time min	Area mAU*min	Relative Area %	Amount n.a.
1		7.360	1.996	2.03	n.a.
2		7.943	96.354	97.97	n.a.
**Total:**			**98.350**	**100.00**	

Supplementary figure 239. HPLC chromatogram for compound **4k**

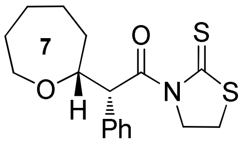

4k’

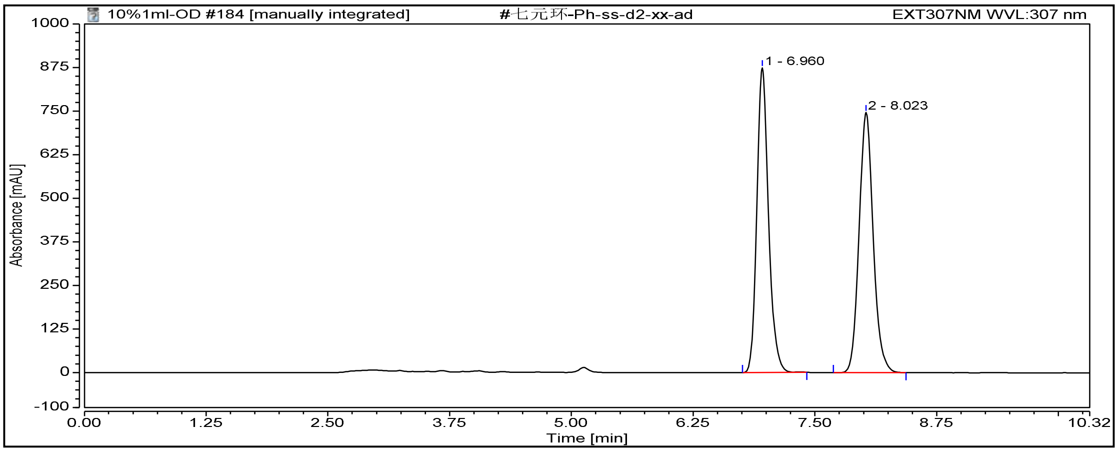

Integration Results					
No.	Peak Name	Retention Time<br>min	Area<br>mAU*min	Relative Area<br>%	Amount<br>n.a.
1		6.960	122.204	49.43	n.a.
2		8.023	125.043	50.57	n.a.
**Total:**			**247.247**	**100.00**	

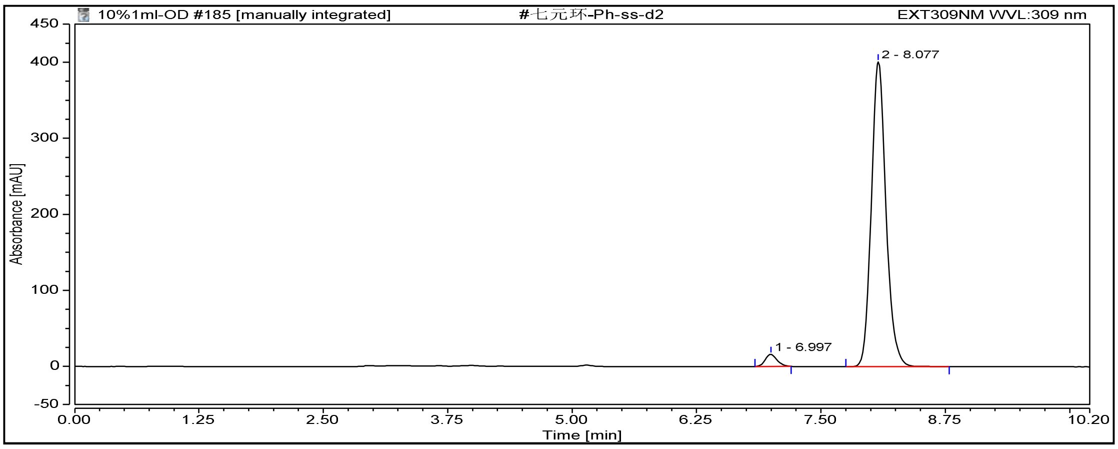

Integration Results					
No.	Peak Name	Retention Time<br>min	Area<br>mAU*min	Relative Area<br>%	Amount<br>n.a.
1		6.997	2.187	3.09	n.a.
2		8.077	68.493	96.91	n.a.
**Total:**			**70.680**	**100.00**	

Supplementary figure 240. HPLC chromatogram for compound **4k’**

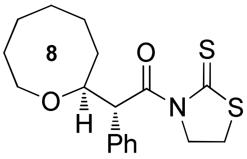

4l

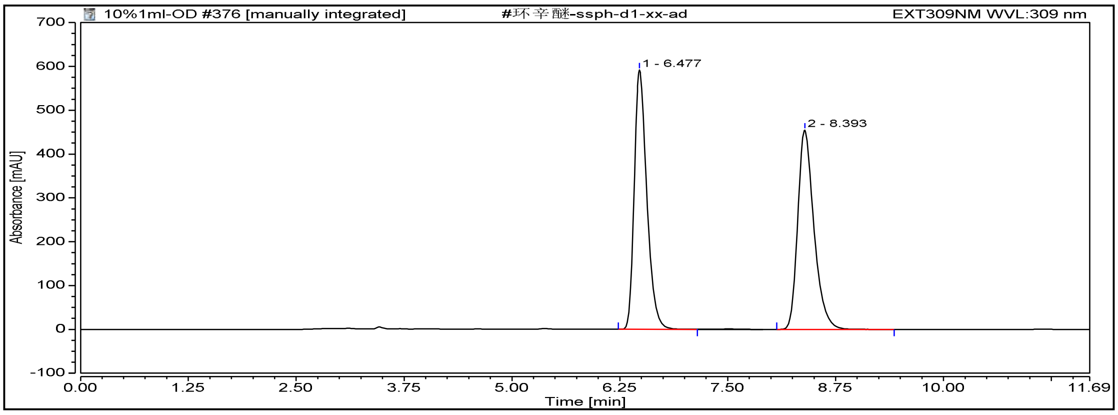

**Integration Results**					
No.	Peak Name	Retention Time<br>min	Area<br>mAU*min	Relative Area<br>%	Amount<br>n.a.
1		6.477	99.738	50.00	n.a.
2		8.393	99.737	50.00	n.a.
**Total:**			**199.475**	**100.00**	

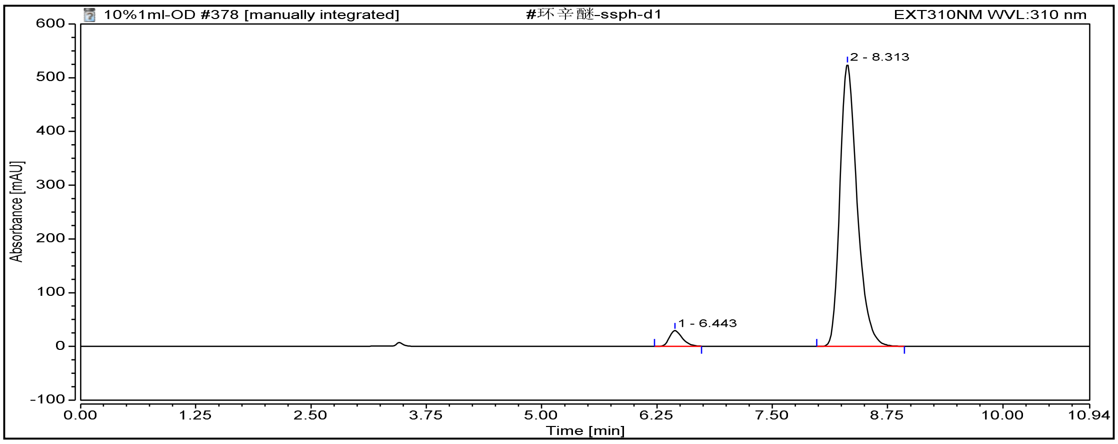

**Integration Results**					
No.	Peak Name	Retention Time<br>min	Area<br>mAU*min	Relative Area<br>%	Amount<br>n.a.
1		6.443	4.780	4.04	n.a.
2		8.313	113.480	95.96	n.a.
**Total:**			**118.259**	**100.00**	

Supplementary figure 241. HPLC chromatogram for compound **4l**

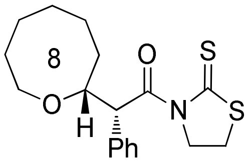

4l'

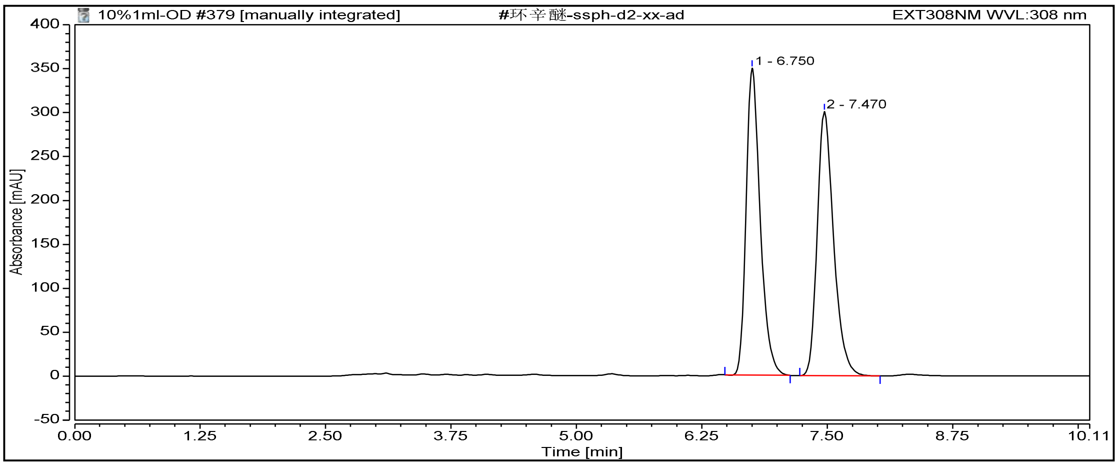

Integration Results					
No.	Peak Name	Retention Time<br>min	Area<br>mAU*min	Relative Area<br>%	Amount<br>n.a.
1		6.750	56.776	49.83	n.a.
2		7.470	57.158	50.17	n.a.
**Total:**			**113.934**	**100.00**	

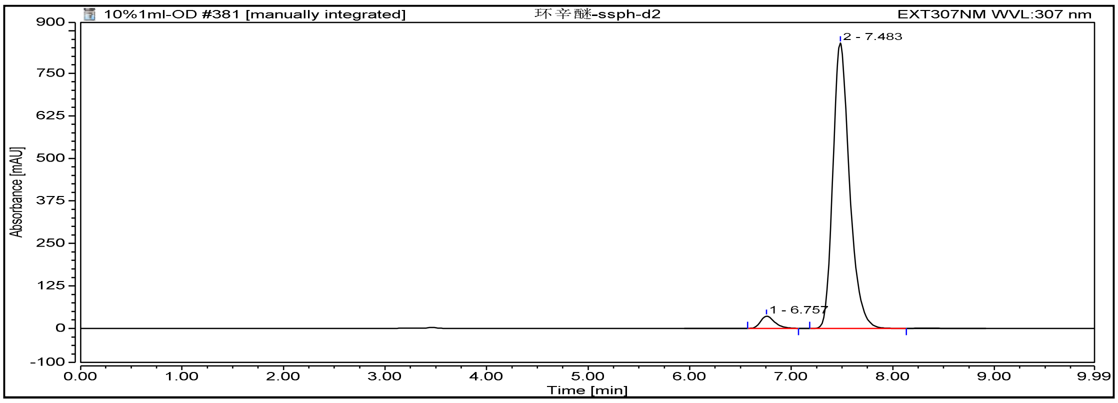

Integration Results					
No.	Peak Name	Retention Time<br>min	Area<br>mAU*min	Relative Area<br>%	Amount<br>n.a.
1		6.757	5.984	3.61	n.a.
2		7.483	159.762	96.39	n.a.
**Total:**			**165.746**	**100.00**	

Supplementary figure 242. HPLC chromatogram for compound **4l'**

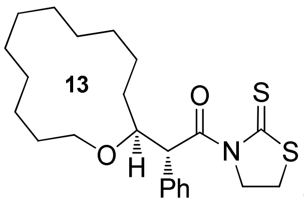

4m

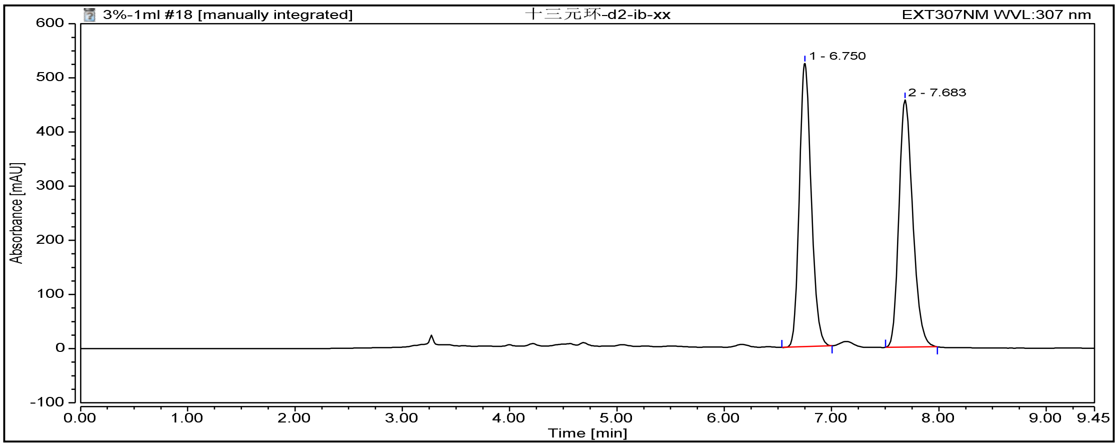

Integration Results					
No.	Peak Name	Retention Time<br>min	Area<br>mAU*min	Relative Area<br>%	Amount<br>n.a.
1		6.750	67.313	49.65	n.a.
2		7.683	68.250	50.35	n.a.
**Total:**			**135.563**	**100.00**	

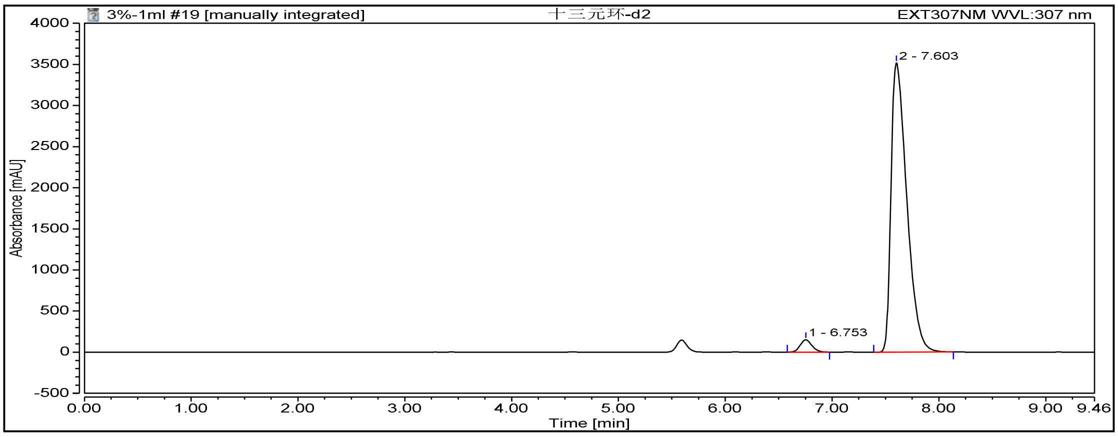

Integration Results					
No.	Peak Name	Retention Time<br>min	Area<br>mAU*min	Relative Area<br>%	Amount<br>n.a.
1		6.753	19.160	3.10	n.a.
2		7.603	599.888	96.90	n.a.
**Total:**			**619.048**	**100.00**	

Supplementary figure 243. HPLC chromatogram for compound **4m**

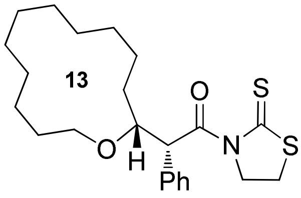

4m’

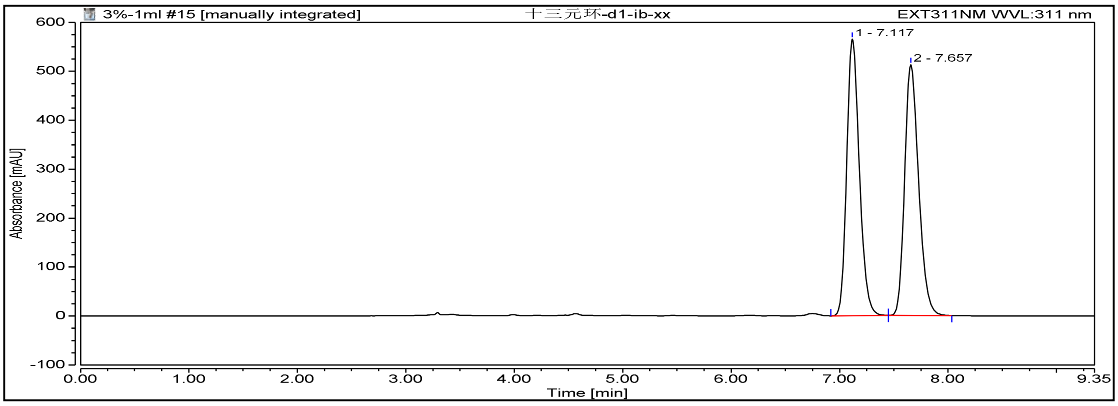

Integration Results					
No.	Peak Name	Retention Time min	Area mAU*min	Relative Area %	Amount n.a.
1		7.117	76.568	49.94	n.a.
2		7.657	76.758	50.06	n.a.
**Total:**			**153.326**	**100.00**	

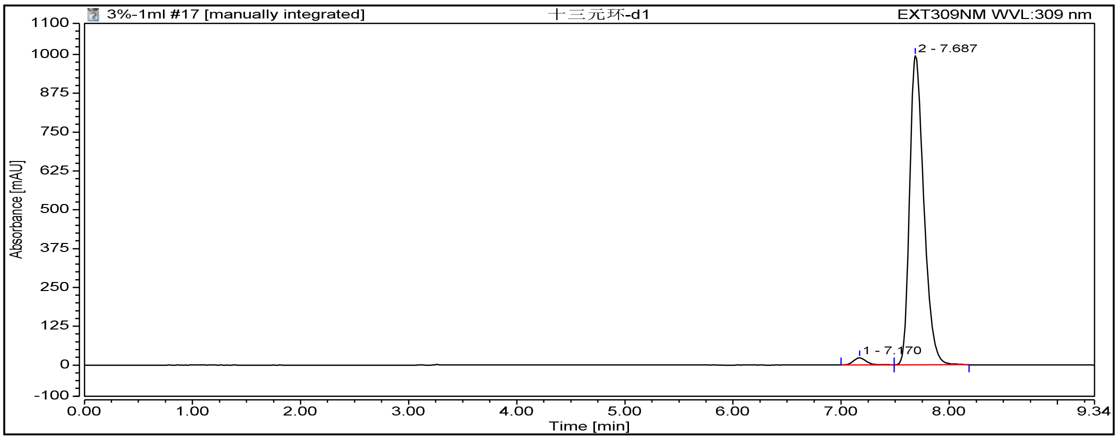

Integration Results					
No.	Peak Name	Retention Time min	Area mAU*min	Relative Area %	Amount n.a.
1		7.170	3.223	2.08	n.a.
2		7.687	151.811	97.92	n.a.
**Total:**			**155.033**	**100.00**	

Supplementary figure 244. HPLC chromatogram for compound **4m’**

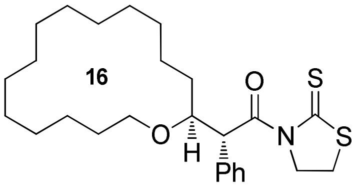

4n

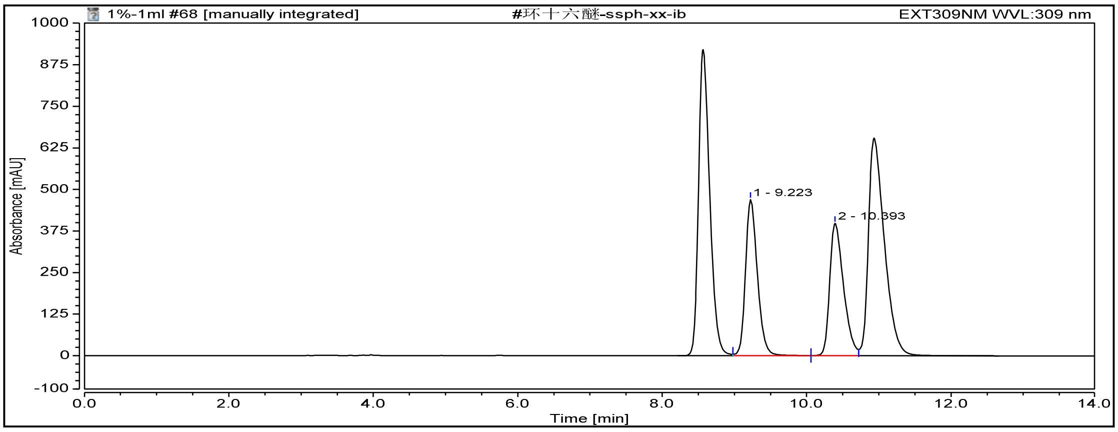

Integration Results					
No.	Peak Name	Retention Time min	Area mAU*min	Relative Area %	Amount n.a.
1		9.223	90.183	50.55	n.a.
2		10.393	88.207	49.45	n.a.
**Total:**			**178.390**	**100.00**	

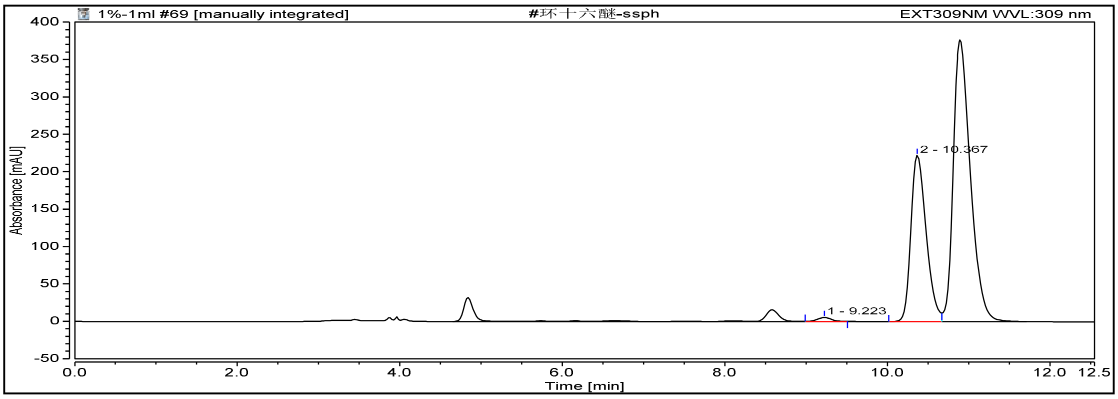

Integration Results					
No.	Peak Name	Retention Time min	Area mAU*min	Relative Area %	Amount n.a.
1		9.223	1.153	2.33	n.a.
2		10.367	48.369	97.67	n.a.
**Total:**			**49.522**	**100.00**	

Supplementary figure 245. HPLC chromatogram for compound **4n**

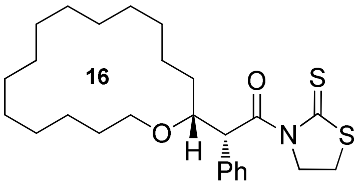

4n’

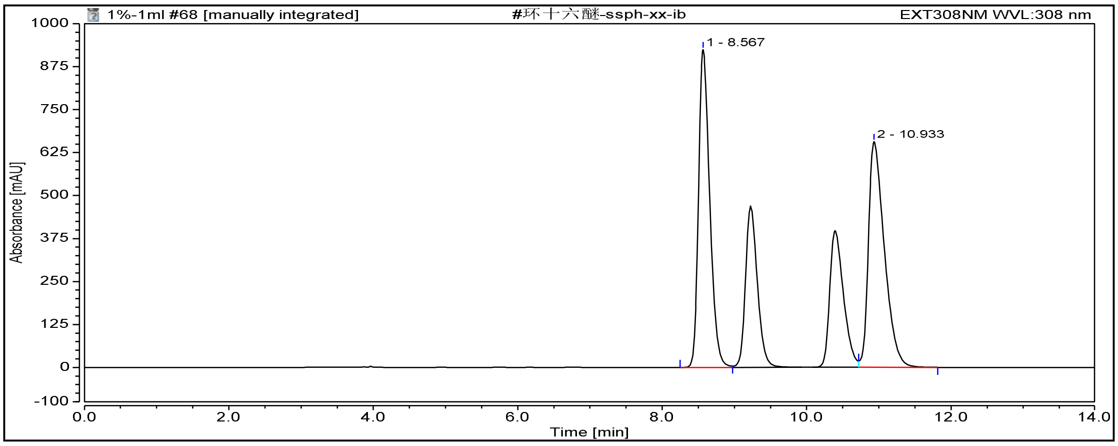

Integration Results					
No.	Peak Name	Retention Time min	Area mAU*min	Relative Area %	Amount n.a.
1		8.567	166.547	49.73	n.a.
2		10.933	168.375	50.27	n.a.
**Total:**			**334.922**	**100.00**	

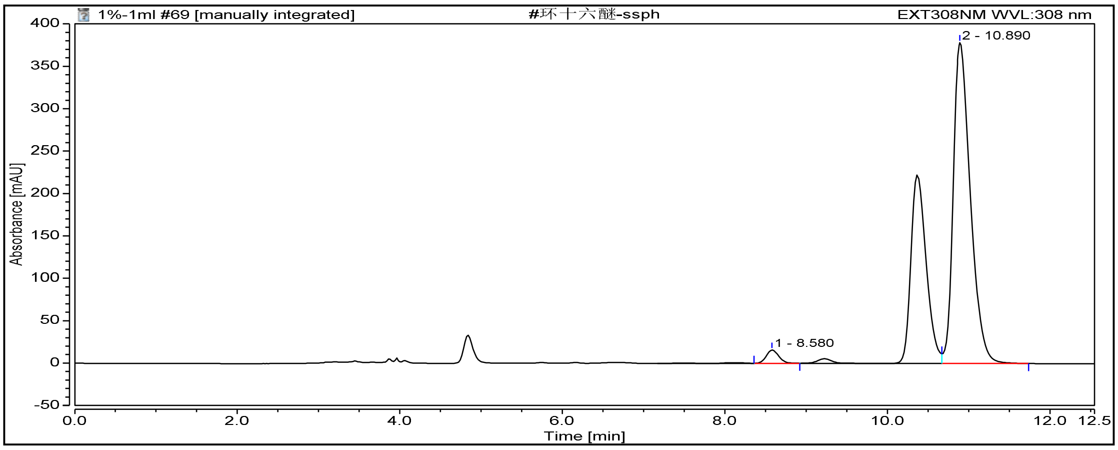

Integration Results					
No.	Peak Name	Retention Time min	Area mAU*min	Relative Area %	Amount n.a.
1		8.580	2.889	3.05	n.a.
2		10.890	91.902	96.95	n.a.
**Total:**			**94.791**	**100.00**	

Supplementary figure 246. HPLC chromatogram for compound **4n’**

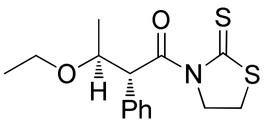

6a

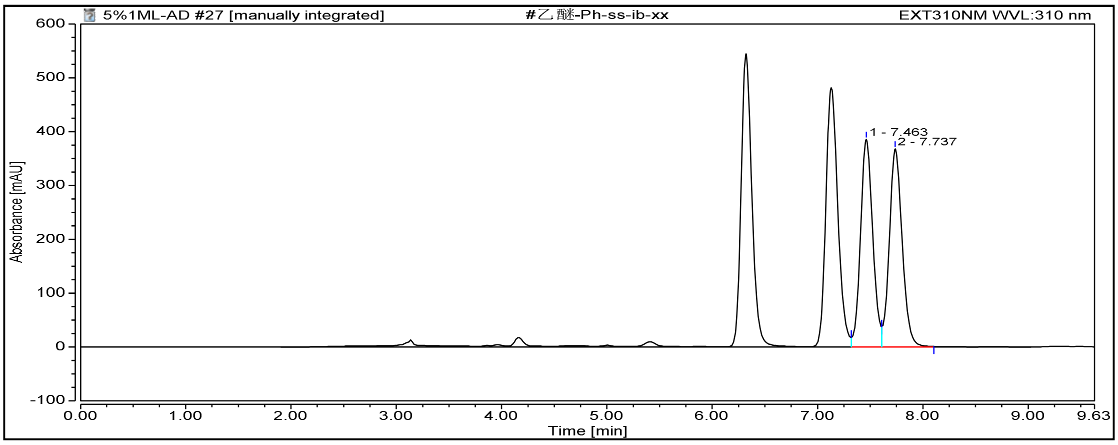

Integration Results					
No.	Peak Name	Retention Time<br>min	Area<br>mAU*min	Relative Area<br>%	Amount<br>n.a.
1		7.463	51.608	49.58	n.a.
2		7.737	52.485	50.42	n.a.
**Total:**			**104.093**	**100.00**	

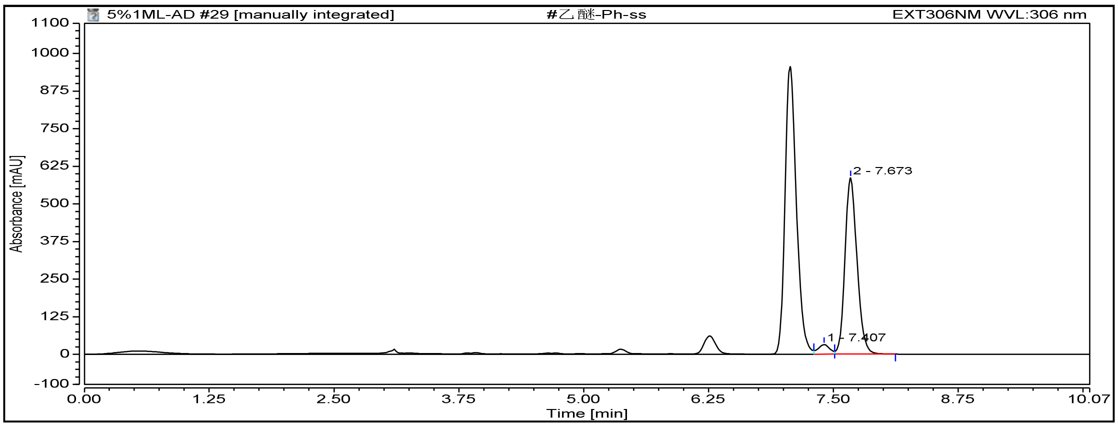

Integration Results					
No.	Peak Name	Retention Time<br>min	Area<br>mAU*min	Relative Area<br>%	Amount<br>n.a.
1		7.407	4.283	4.97	n.a.
2		7.673	81.809	95.03	n.a.
**Total:**			**86.091**	**100.00**	

Supplementary figure 247. HPLC chromatogram for compound **6a**

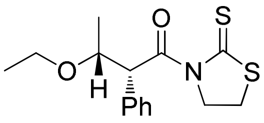

6a'

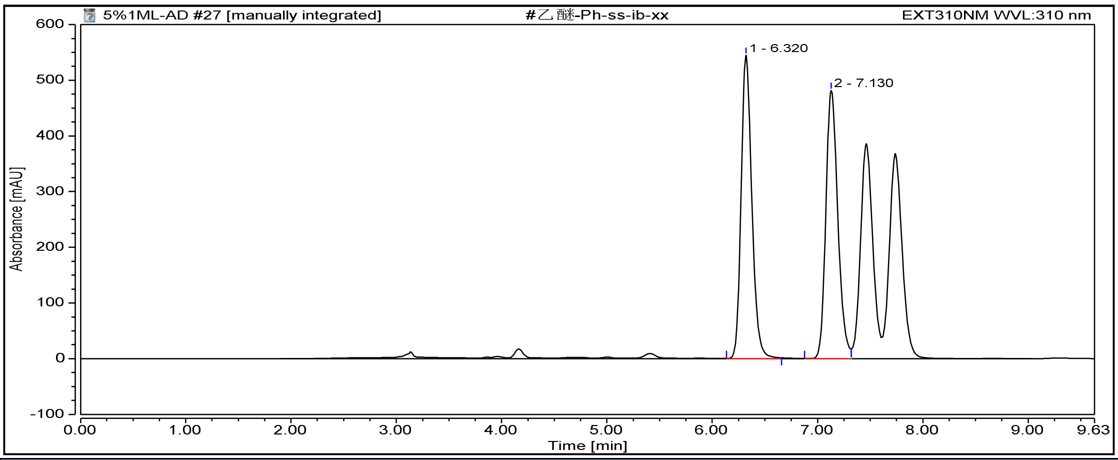

Integration Results					
No.	Peak Name	Retention Time min	Area mAU*min	Relative Area %	Amount n.a.
1		6.320	64.297	50.22	n.a.
2		7.130	63.741	49.78	n.a.
**Total:**			**128.038**	**100.00**	

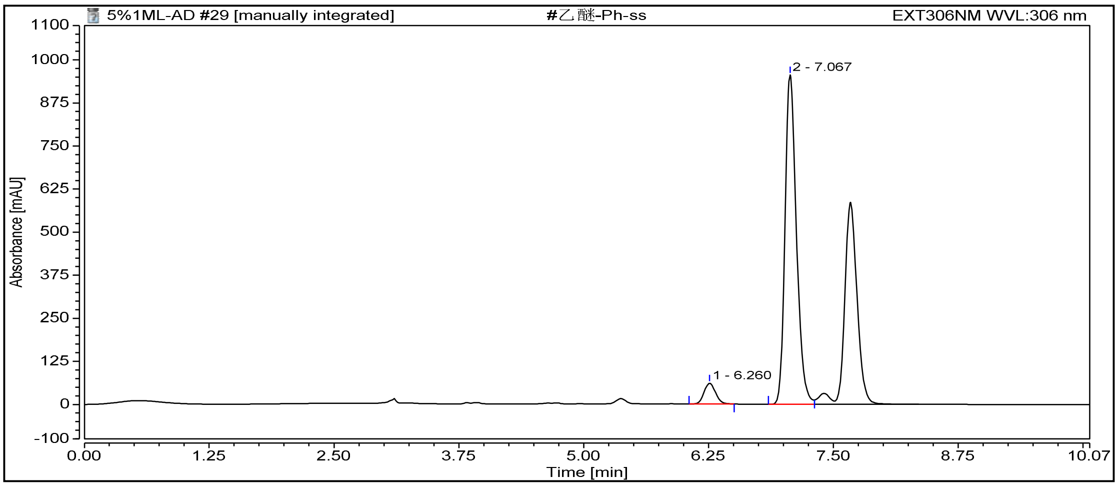

Integration Results					
No.	Peak Name	Retention Time min	Area mAU*min	Relative Area %	Amount n.a.
1		6.260	8.132	6.04	n.a.
2		7.067	126.512	93.96	n.a.
**Total:**			**134.644**	**100.00**	

Supplementary figure 248. HPLC chromatogram for compound **6a'**

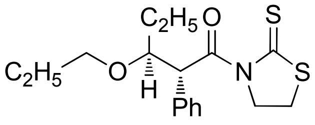

6b

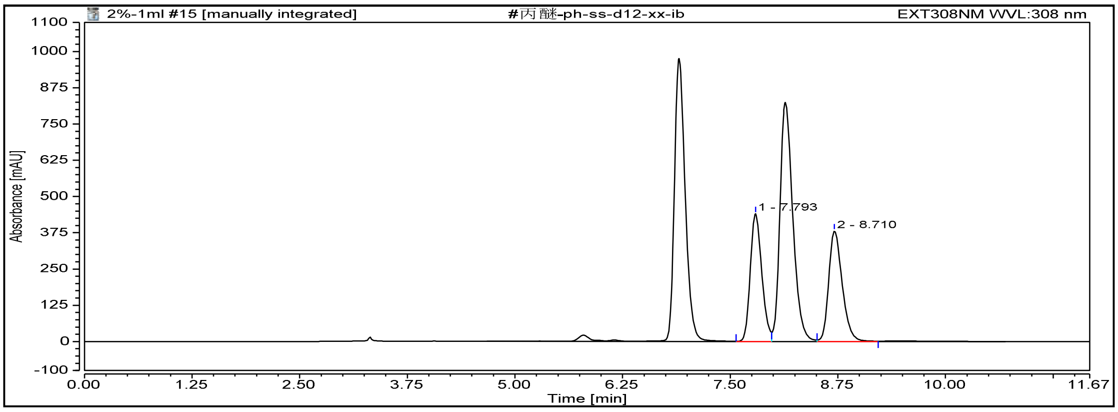

Integration Results

No.	Peak Name	Retention Time min	Area mAU*min	Relative Area %	Amount n.a.
1		7.793	68.058	49.41	n.a.
2		8.710	69.689	50.59	n.a.
**Total:**			**137.747**	**100.00**	

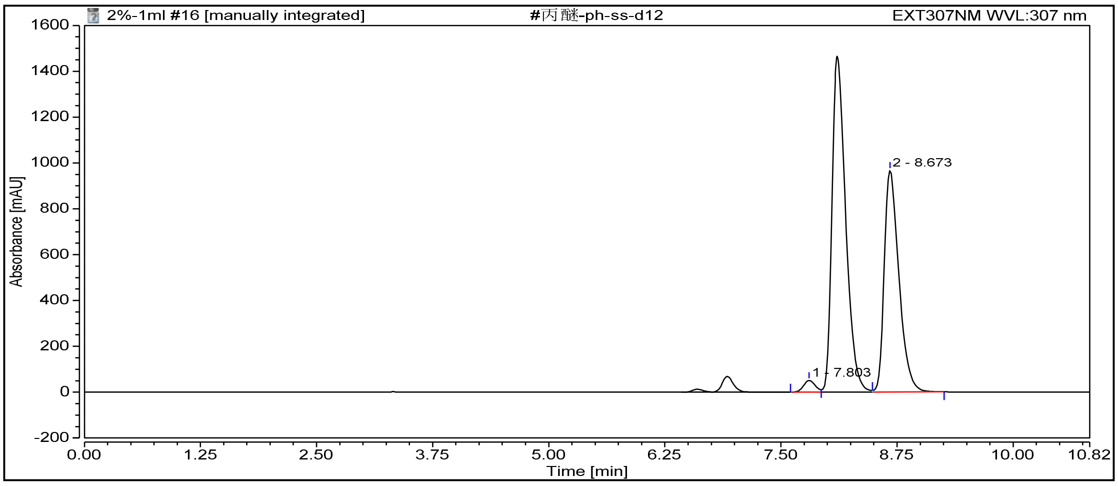

Integration Results

No.	Peak Name	Retention Time min	Area mAU*min	Relative Area %	Amount n.a.
1		7.803	7.302	3.96	n.a.
2		8.673	176.864	96.04	n.a.
**Total:**			**184.165**	**100.00**	

Supplementary figure 249. HPLC chromatogram for compound **6b**

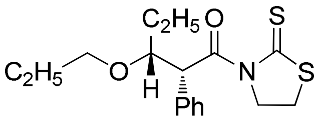

6b'

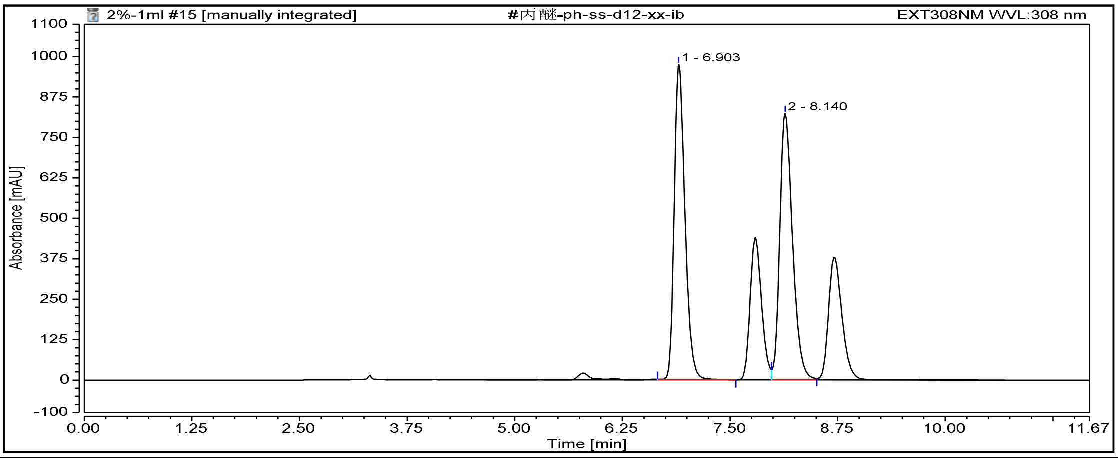

Integration Results

No.	Peak Name	Retention Time<br>min	Area<br>mAU*min	Relative Area<br>%	Amount<br>n.a.
1		6.903	141.429	49.74	n.a.
2		8.140	142.922	50.26	n.a.
**Total:**			**284.351**	**100.00**	

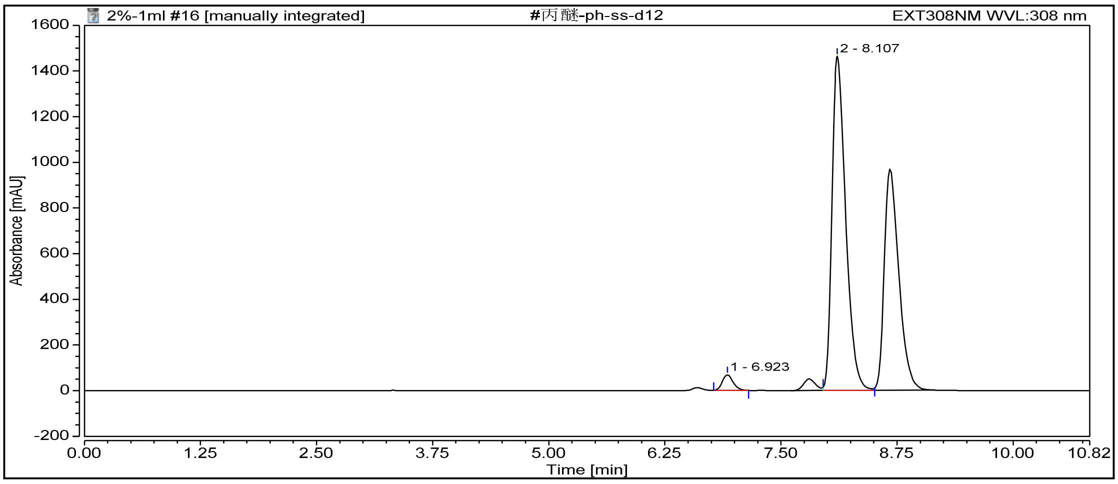

Integration Results

No.	Peak Name	Retention Time<br>min	Area<br>mAU*min	Relative Area<br>%	Amount<br>n.a.
1		6.923	9.264	3.58	n.a.
2		8.107	249.374	96.42	n.a.
**Total:**			**258.638**	**100.00**	

Supplementary figure 250. HPLC chromatogram for compound **6b'**

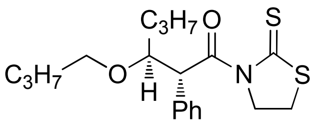

6c

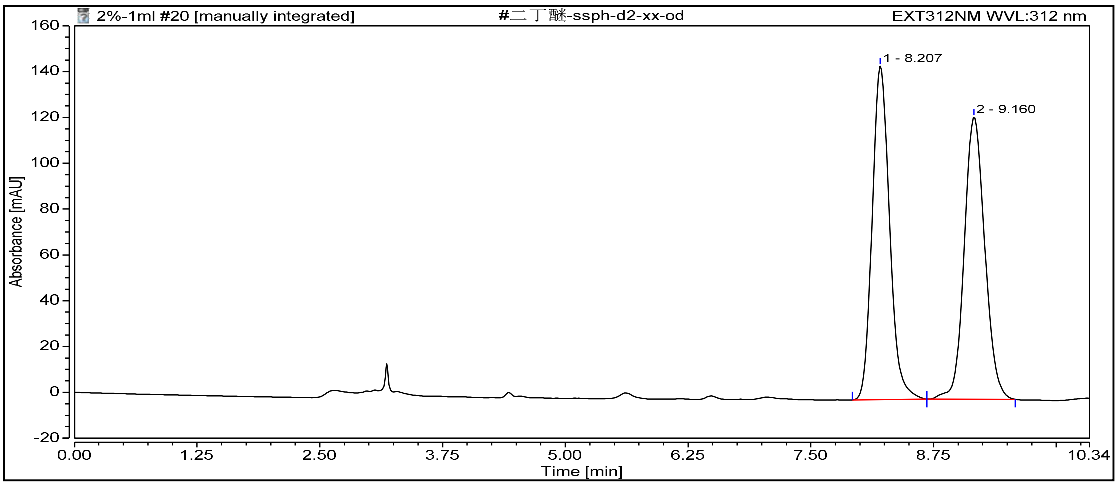

Integration Results					
No.	Peak Name	Retention Time<br>min	Area<br>mAU*min	Relative Area<br>%	Amount<br>n.a.
1		8.207	29.933	49.98	n.a.
2		9.160	29.959	50.02	n.a.
**Total:**			**59.892**	**100.00**	

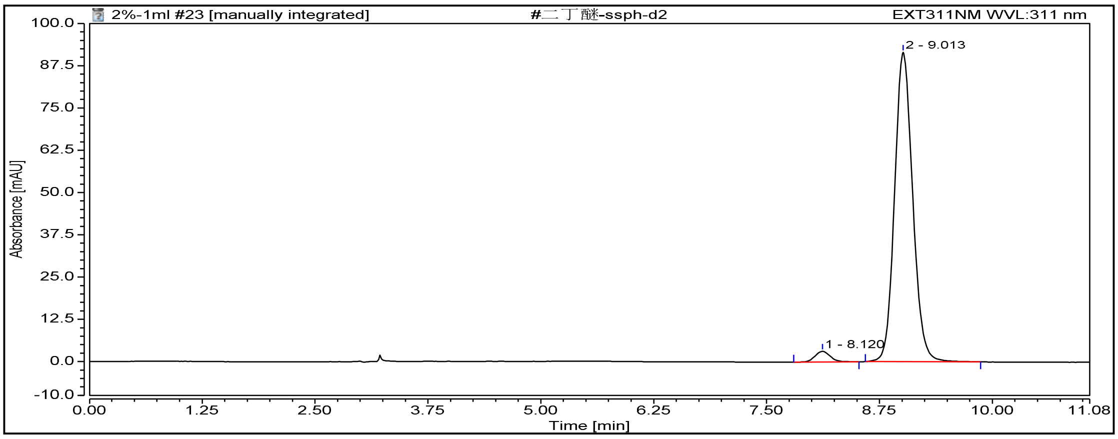

Integration Results					
No.	Peak Name	Retention Time<br>min	Area<br>mAU*min	Relative Area<br>%	Amount<br>n.a.
1		8.120	0.614	2.81	n.a.
2		9.013	21.271	97.19	n.a.
**Total:**			**21.886**	**100.00**	

Supplementary figure 251. HPLC chromatogram for compound **6c**

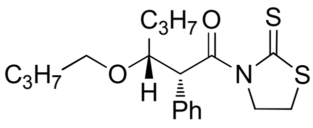

6c'

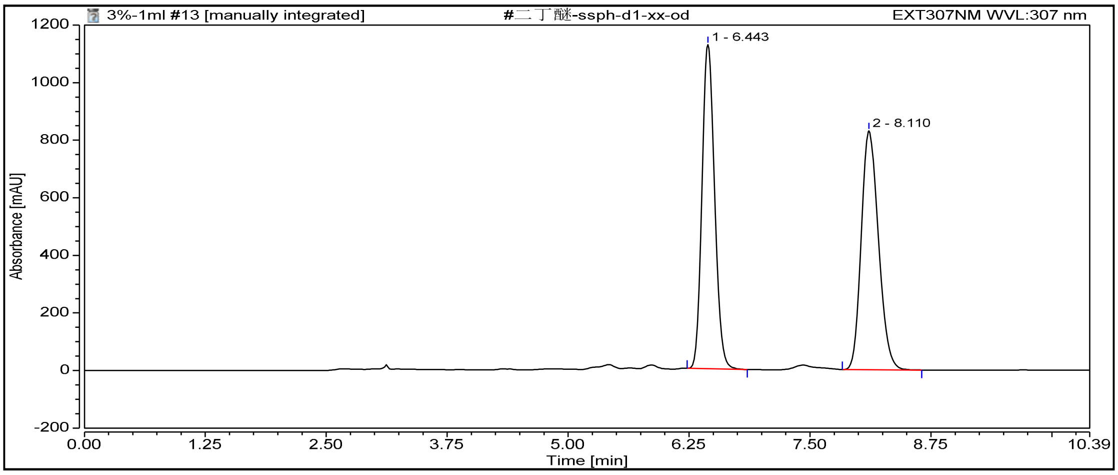

Integration Results					
No.	Peak Name	Retention Time min	Area mAU*min	Relative Area %	Amount n.a.
1		6.443	167.975	49.67	n.a.
2		8.110	170.177	50.33	n.a.
**Total:**			**338.152**	**100.00**	

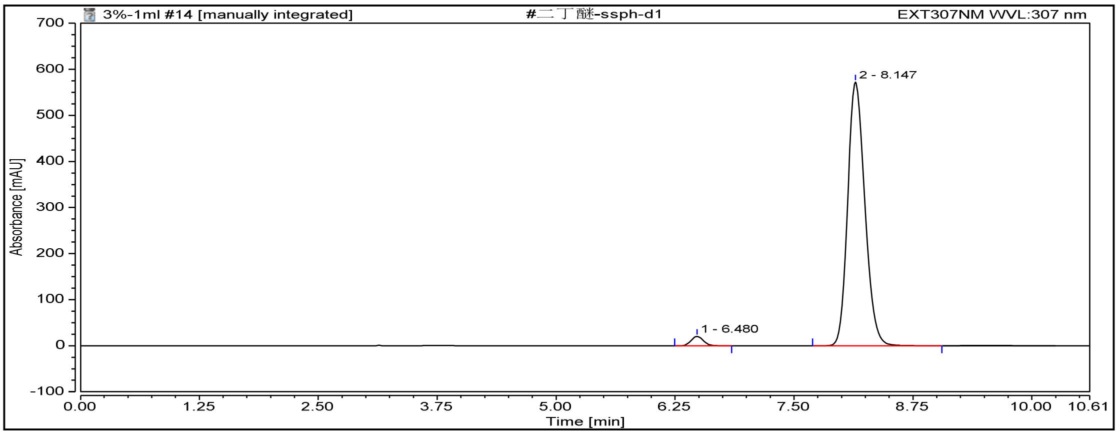

Integration Results					
No.	Peak Name	Retention Time min	Area mAU*min	Relative Area %	Amount n.a.
1		6.480	3.118	2.53	n.a.
2		8.147	120.193	97.47	n.a.
**Total:**			**123.311**	**100.00**	

Supplementary figure 252. HPLC chromatogram for compound **6c'**

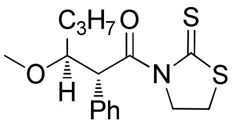

6d

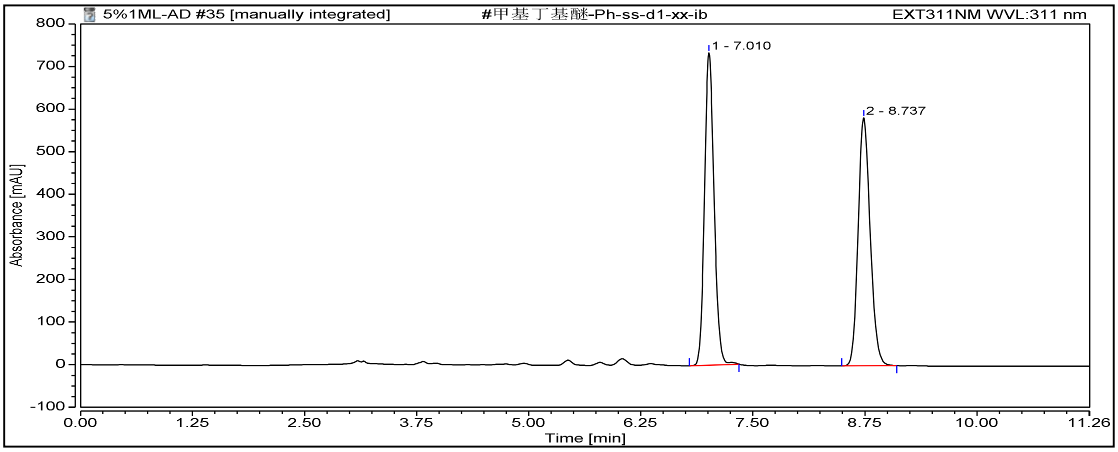

Integration Results					
No.	Peak Name	Retention Time<br>min	Area<br>mAU*min	Relative Area<br>%	Amount<br>n.a.
1		7.010	90.592	49.97	n.a.
2		8.737	90.689	50.03	n.a.
**Total:**			**181.281**	**100.00**	

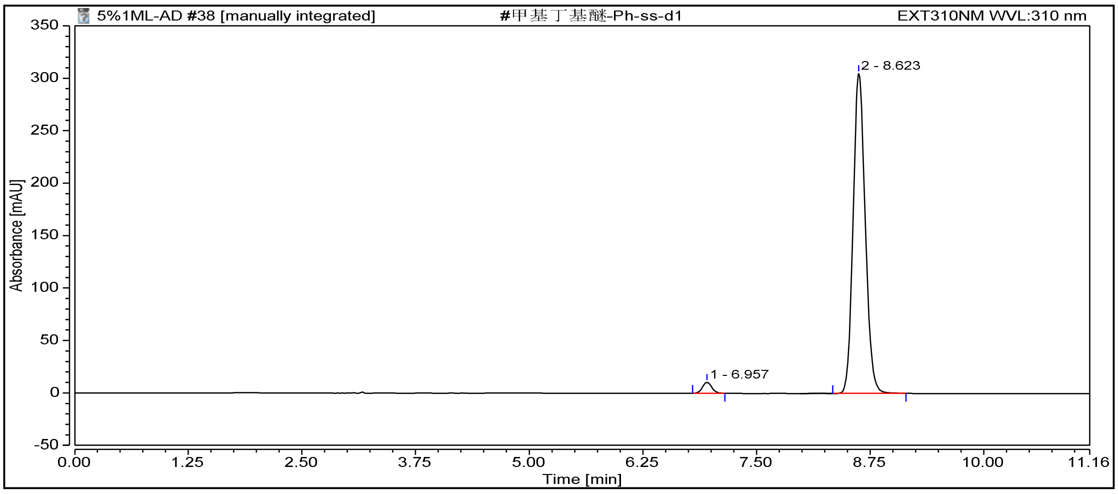

Integration Results					
No.	Peak Name	Retention Time<br>min	Area<br>mAU*min	Relative Area<br>%	Amount<br>n.a.
1		6.957	1.264	2.62	n.a.
2		8.623	47.024	97.38	n.a.
**Total:**			**48.288**	**100.00**	

Supplementary figure 253. HPLC chromatogram for compound **6d**

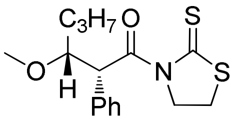

6d’

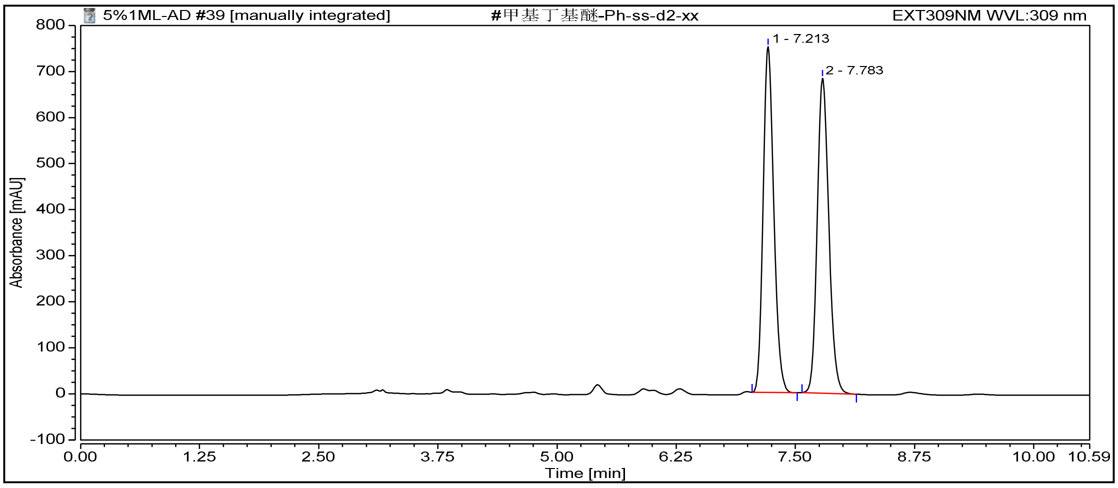

Integration Results					
No.	Peak Name	Retention Time<br>min	Area<br>mAU*min	Relative Area<br>%	Amount<br>n.a.
1		7.213	96.772	49.89	n.a.
2		7.783	97.198	50.11	n.a.
**Total:**			**193.970**	**100.00**	

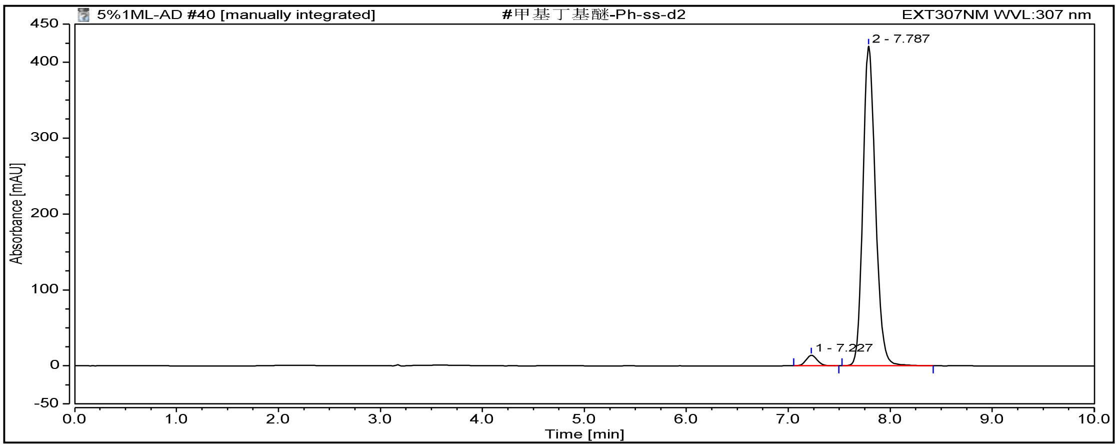

Integration Results					
No.	Peak Name	Retention Time<br>min	Area<br>mAU*min	Relative Area<br>%	Amount<br>n.a.
1		7.227	1.781	2.88	n.a.
2		7.787	60.000	97.12	n.a.
**Total:**			**61.781**	**100.00**	

Supplementary figure 254. HPLC chromatogram for compound **6d’**

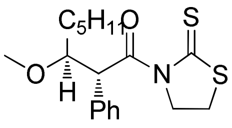

6e

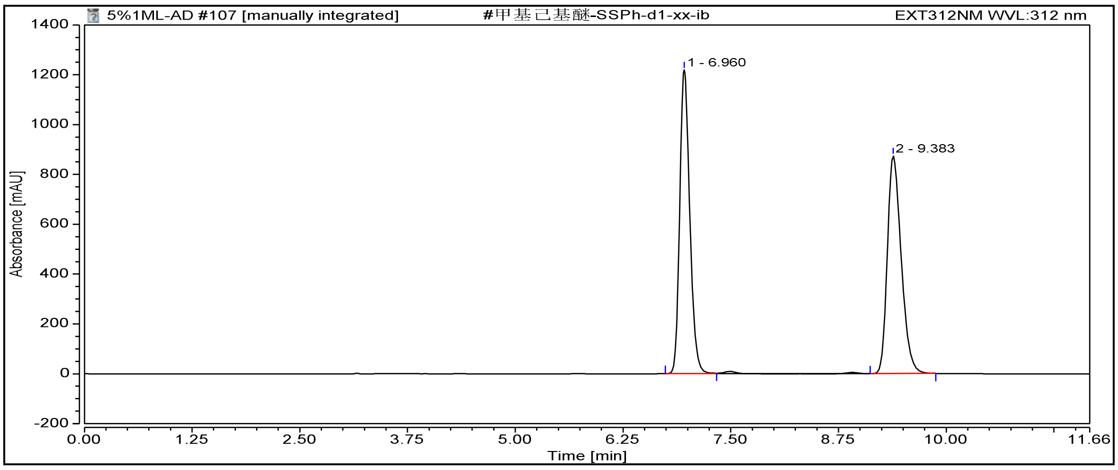

Integration Results					
No.	Peak Name	Retention Time<br>min	Area<br>mAU*min	Relative Area<br>%	Amount<br>n.a.
1		6.960	163.972	50.20	n.a.
2		9.383	162.695	49.80	n.a.
**Total:**			**326.667**	**100.00**	

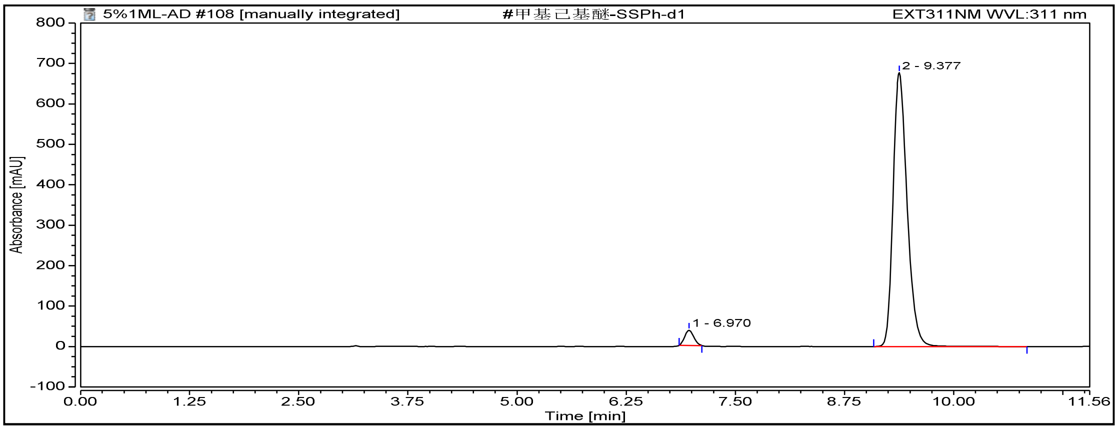

Integration Results					
No.	Peak Name	Retention Time<br>min	Area<br>mAU*min	Relative Area<br>%	Amount<br>n.a.
1		6.970	4.537	3.48	n.a.
2		9.377	125.695	96.52	n.a.
**Total:**			**130.232**	**100.00**	

Supplementary figure 255. HPLC chromatogram for compound **6e**

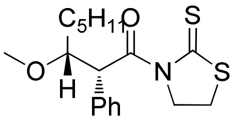

6e’

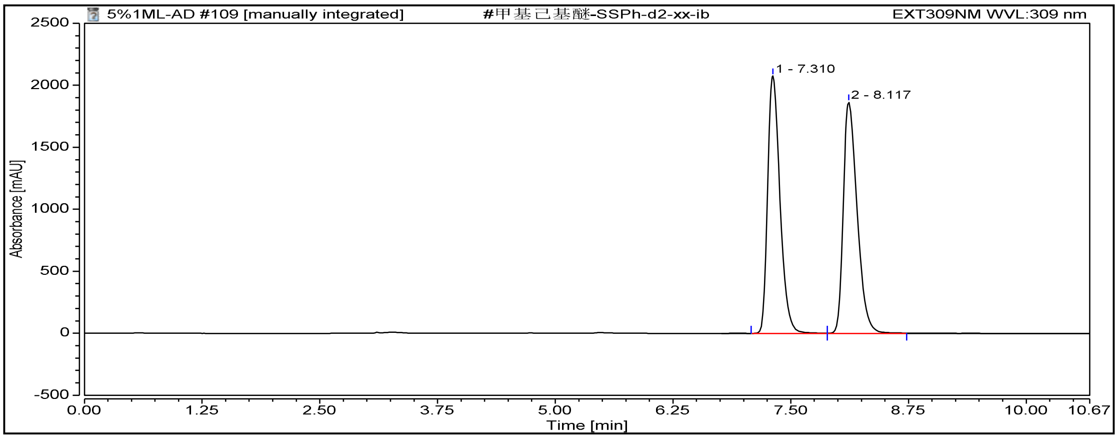

Integration Results					
No.	Peak Name	Retention Time<br>min	Area<br>mAU*min	Relative Area<br>%	Amount<br>n.a.
1		7.310	314.584	49.67	n.a.
2		8.117	318.820	50.33	n.a.
**Total:**			**633.404**	**100.00**	

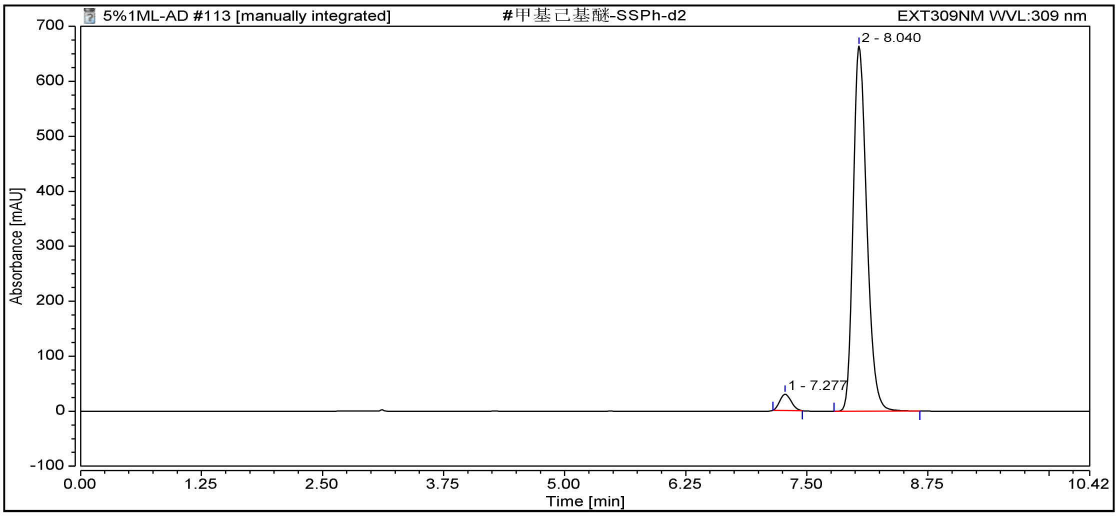

Integration Results					
No.	Peak Name	Retention Time<br>min	Area<br>mAU*min	Relative Area<br>%	Amount<br>n.a.
1		7.277	3.992	3.60	n.a.
2		8.040	107.015	96.40	n.a.
**Total:**			**111.008**	**100.00**	

Supplementary figure 256. HPLC chromatogram for compound **6e’**

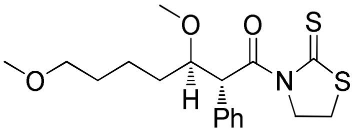

6f

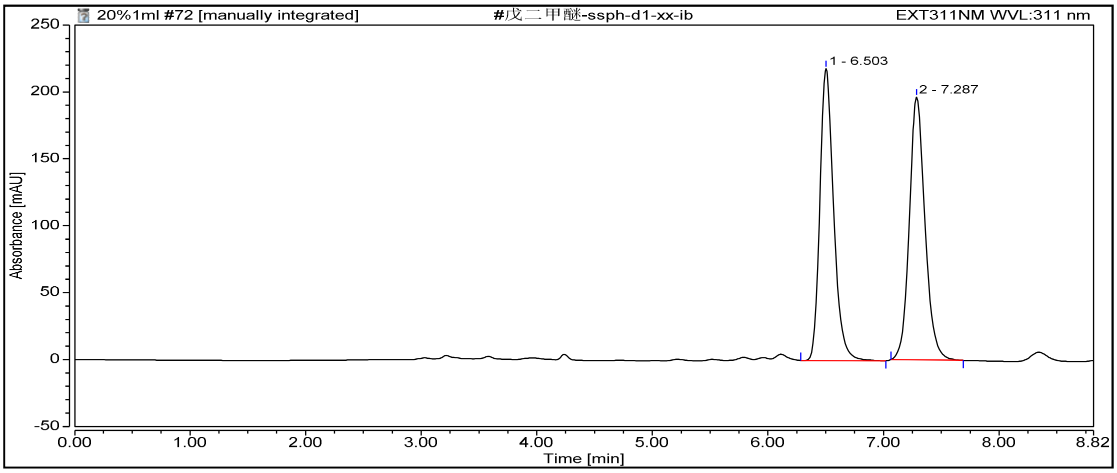

Integration Results					
No.	Peak Name	Retention Time<br>min	Area<br>mAU*min	Relative Area<br>%	Amount<br>n.a.
1		6.503	30.172	49.39	n.a.
2		7.287	30.918	50.61	n.a.
**Total:**			**61.090**	**100.00**	

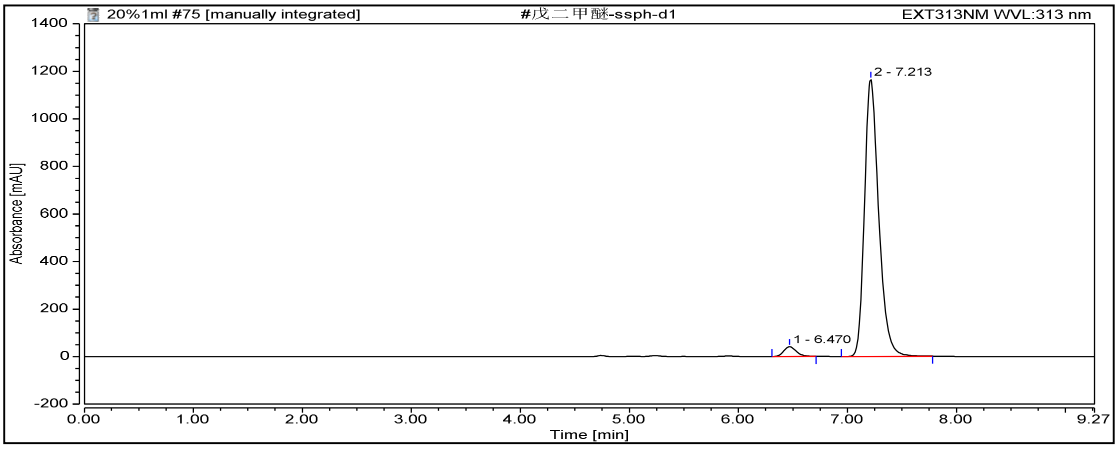

Integration Results					
No.	Peak Name	Retention Time<br>min	Area<br>mAU*min	Relative Area<br>%	Amount<br>n.a.
1		6.470	5.444	3.00	n.a.
2		7.213	176.260	97.00	n.a.
**Total:**			**181.704**	**100.00**	

Supplementary figure 257. HPLC chromatogram for compound **6f**

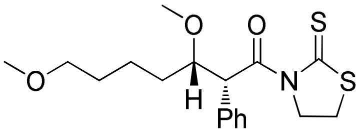

6f'

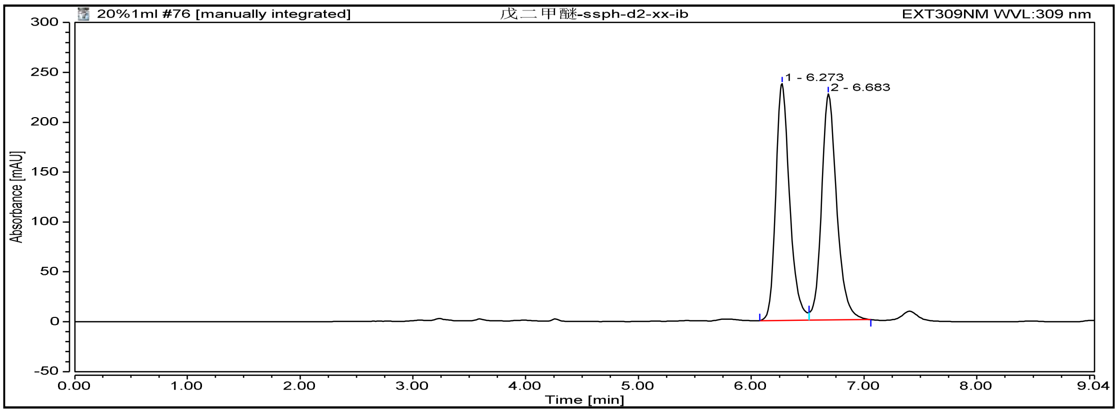

Integration Results					
No.	Peak Name	Retention Time min	Area mAU*min	Relative Area %	Amount n.a.
1		6.273	33.831	48.87	n.a.
2		6.683	35.398	51.13	n.a.
**Total:**			**69.229**	**100.00**	

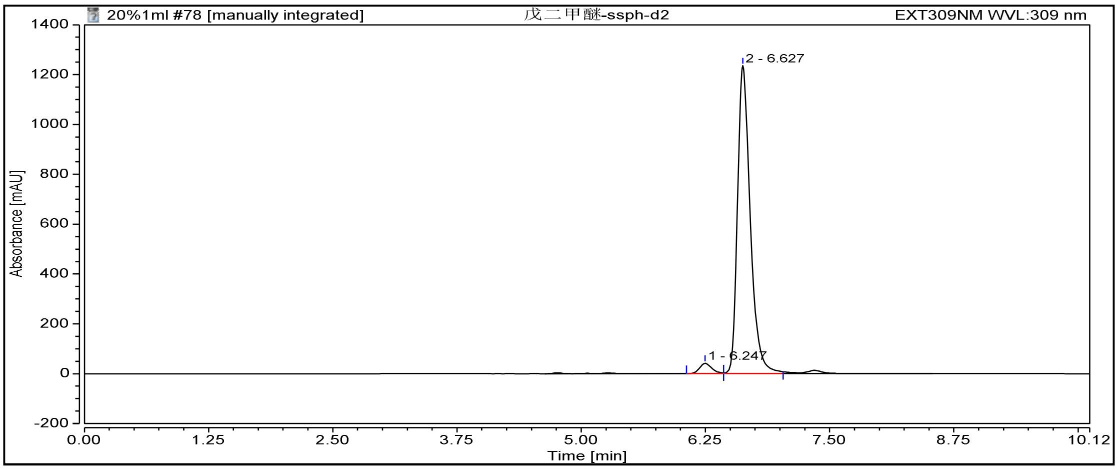

Integration Results					
No.	Peak Name	Retention Time min	Area mAU*min	Relative Area %	Amount n.a.
1		6.247	5.975	3.15	n.a.
2		6.627	183.763	96.85	n.a.
**Total:**			**189.738**	**100.00**	

Supplementary figure 258. HPLC chromatogram for compound **6f'**

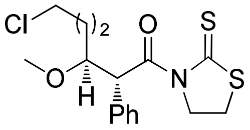

6g

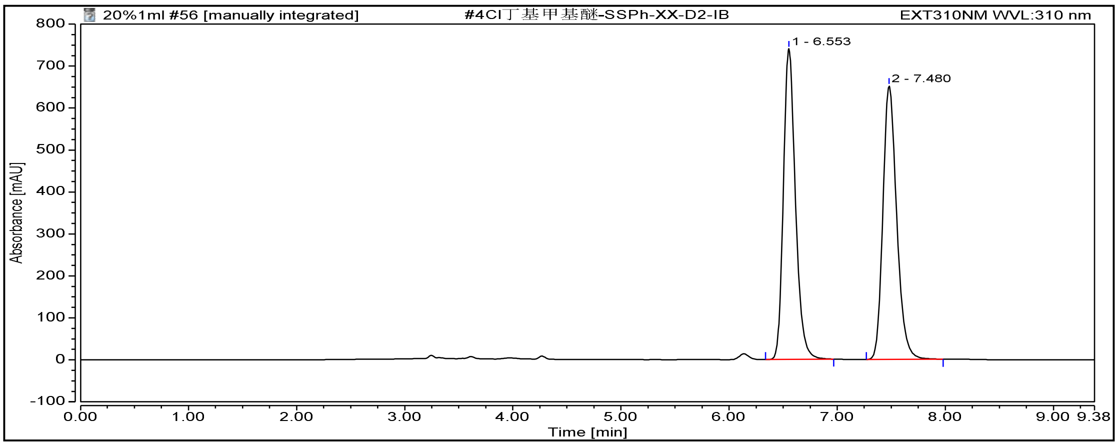

Integration Results					
No.	Peak Name	Retention Time<br>min	Area<br>mAU*min	Relative Area<br>%	Amount<br>n.a.
1		6.553	95.827	49.80	n.a.
2		7.480	96.594	50.20	n.a.
**Total:**			**192.420**	**100.00**	

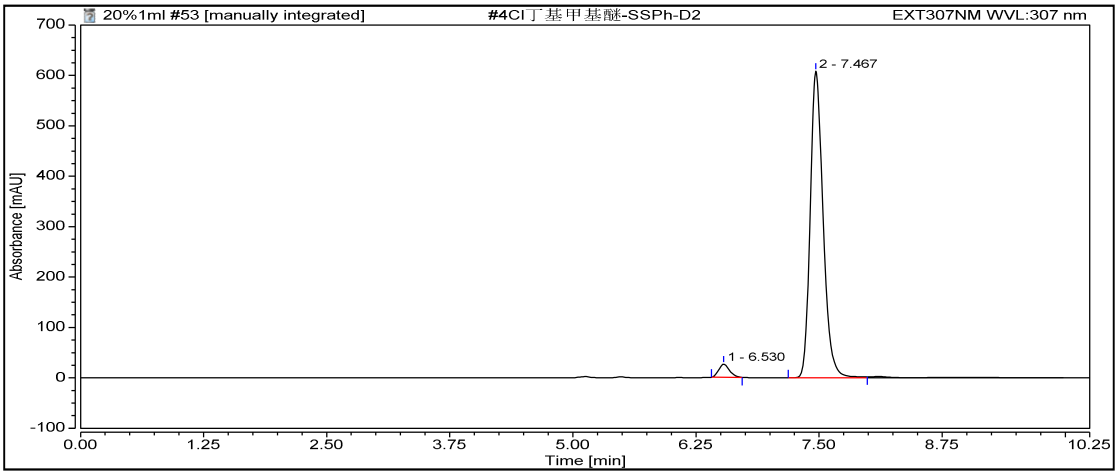

Integration Results					
No.	Peak Name	Retention Time<br>min	Area<br>mAU*min	Relative Area<br>%	Amount<br>n.a.
1		6.530	3.310	3.45	n.a.
2		7.467	92.626	96.55	n.a.
**Total:**			**95.936**	**100.00**	

Supplementary figure 259. HPLC chromatogram for compound **6g**

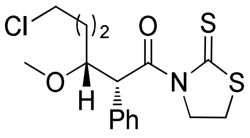

6g’

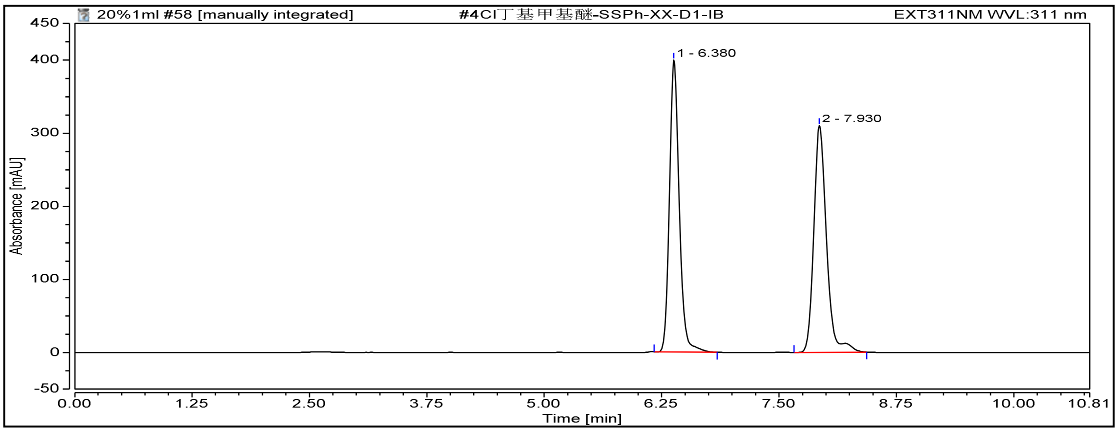

Integration Results					
No.	Peak Name	Retention Time min	Area mAU*min	Relative Area %	Amount n.a.
1		6.380	48.458	49.91	n.a.
2		7.930	48.638	50.09	n.a.
**Total:**			**97.096**	**100.00**	

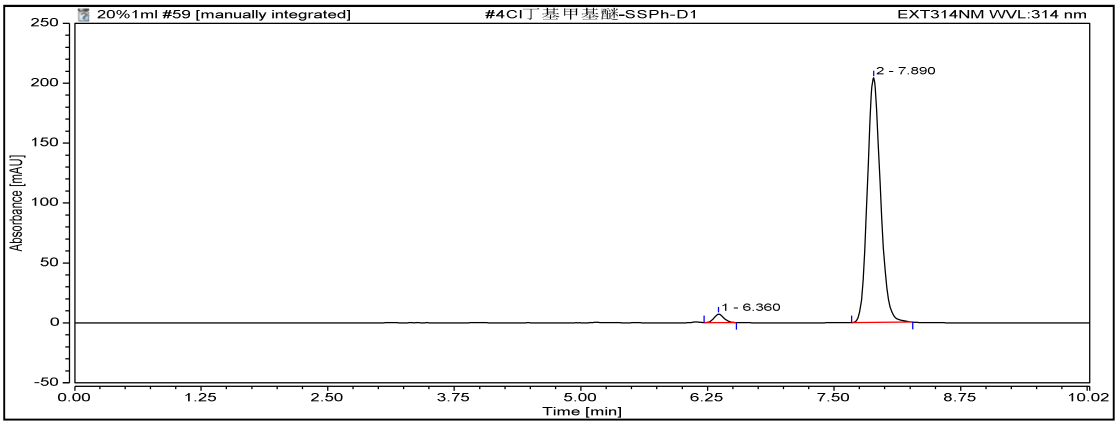

Integration Results					
No.	Peak Name	Retention Time min	Height mAU	Relative Area %	Amount n.a.
1		6.360	6.980	2.52	n.a.
2		7.890	204.123	97.48	n.a.
**Total:**			**211.103**	**100.00**	

Supplementary figure 260. HPLC chromatogram for compound **6g’**

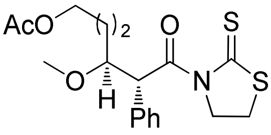

6h

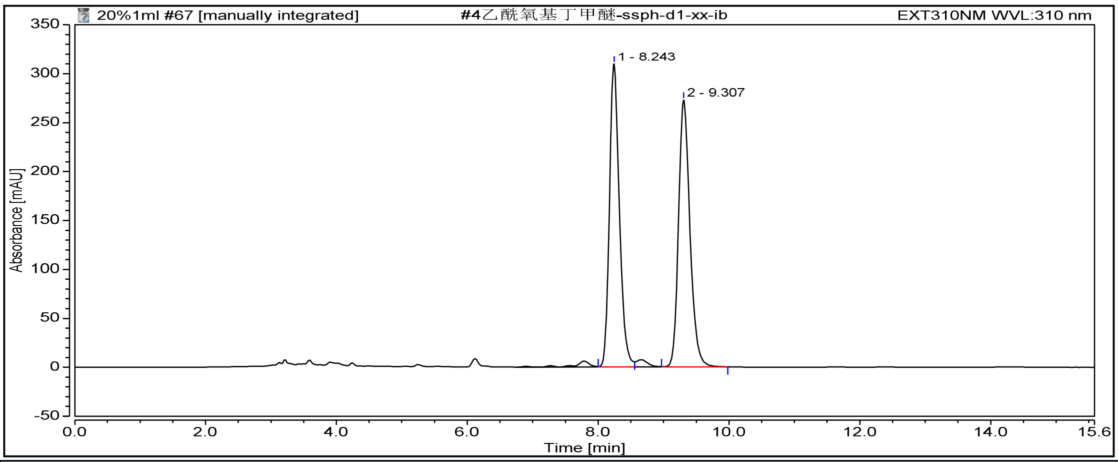

Integration Results					
No.	Peak Name	Retention Time<br>min	Area<br>mAU*min	Relative Area<br>%	Amount<br>n.a.
1		8.243	54.527	49.69	n.a.
2		9.307	55.210	50.31	n.a.
**Total:**			**109.737**	**100.00**	

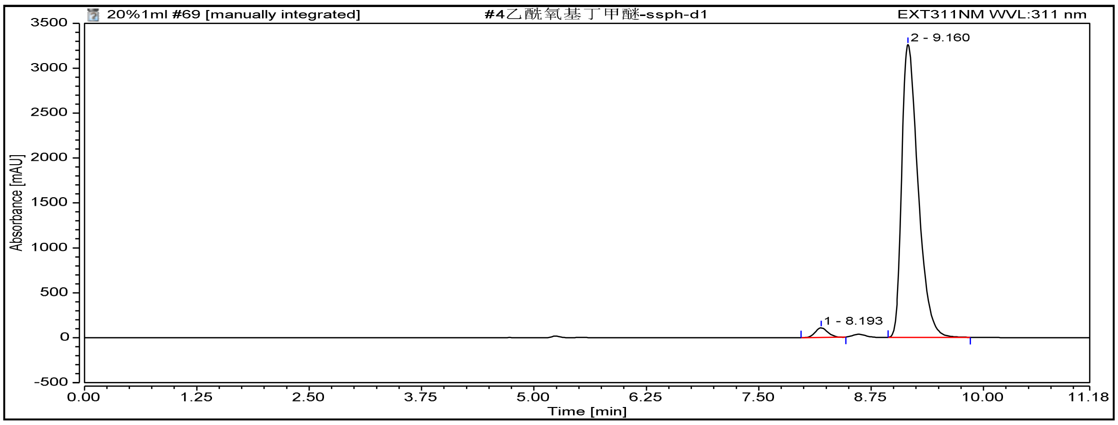

Integration Results					
No.	Peak Name	Retention Time<br>min	Area<br>mAU*min	Relative Area<br>%	Amount<br>n.a.
1		8.193	17.252	2.45	n.a.
2		9.160	688.343	97.55	n.a.
**Total:**			**705.595**	**100.00**	

Supplementary figure 261. HPLC chromatogram for compound **6h**

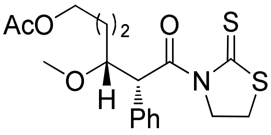

6h’

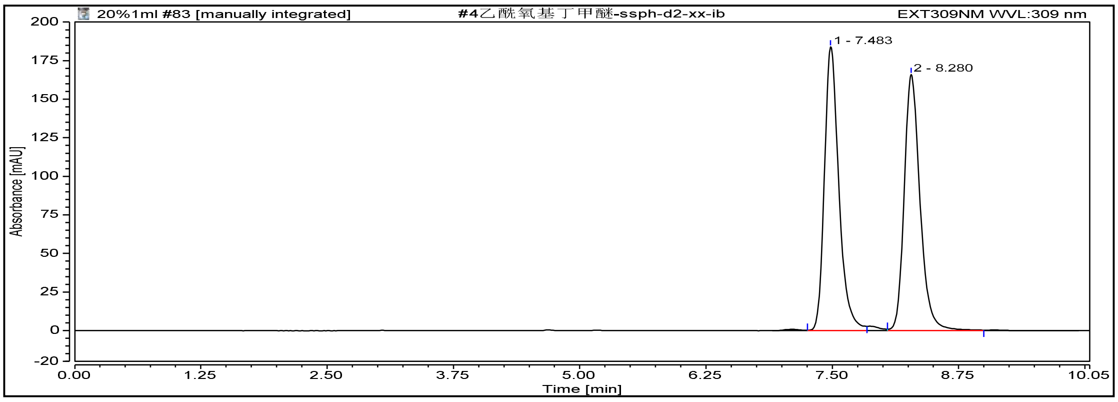

Integration Results

No.	Peak Name	Retention Time<br>min	Area<br>mAU*min	Relative Area<br>%	Amount<br>n.a.
1		7.483	30.504	50.18	n.a.
2		8.280	30.283	49.82	n.a.
**Total:**			**60.787**	**100.00**	

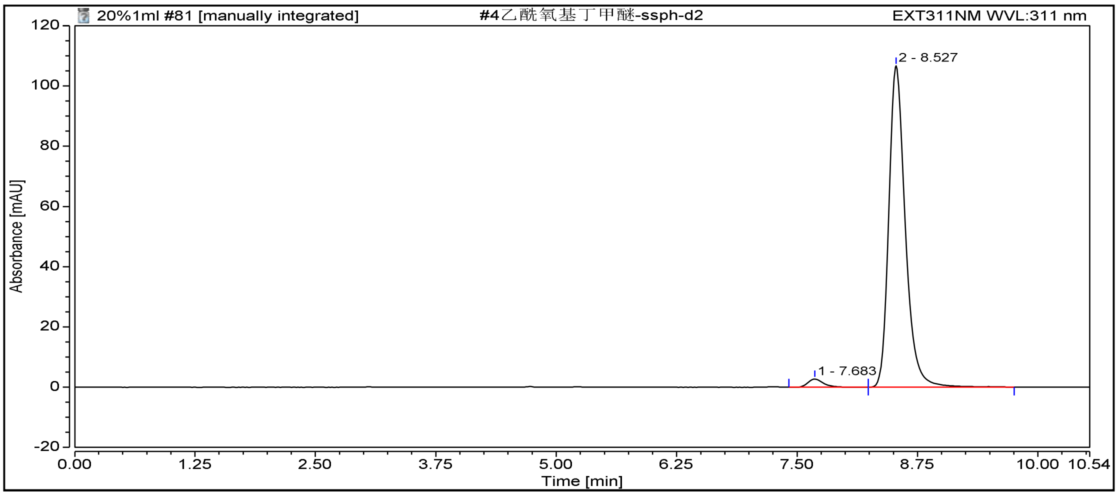

Integration Results

No.	Peak Name	Retention Time<br>min	Area<br>mAU*min	Relative Area<br>%	Amount<br>n.a.
1		7.683	0.512	2.35	n.a.
2		8.527	21.251	97.65	n.a.
**Total:**			**21.763**	**100.00**	

Supplementary figure 262. HPLC chromatogram for compound **6h’**

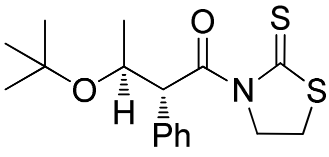

6i

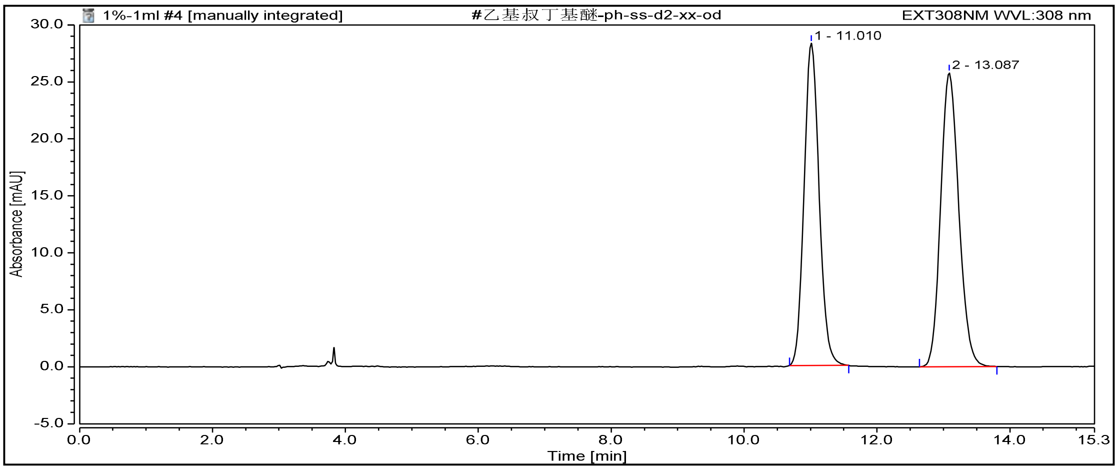

Integration Results					
No.	Peak Name	Retention Time<br>min	Area<br>mAU*min	Relative Area<br>%	Amount<br>n.a.
1		11.010	7.420	47.57	n.a.
2		13.087	8.176	52.43	n.a.
**Total:**			**15.596**	**100.00**	

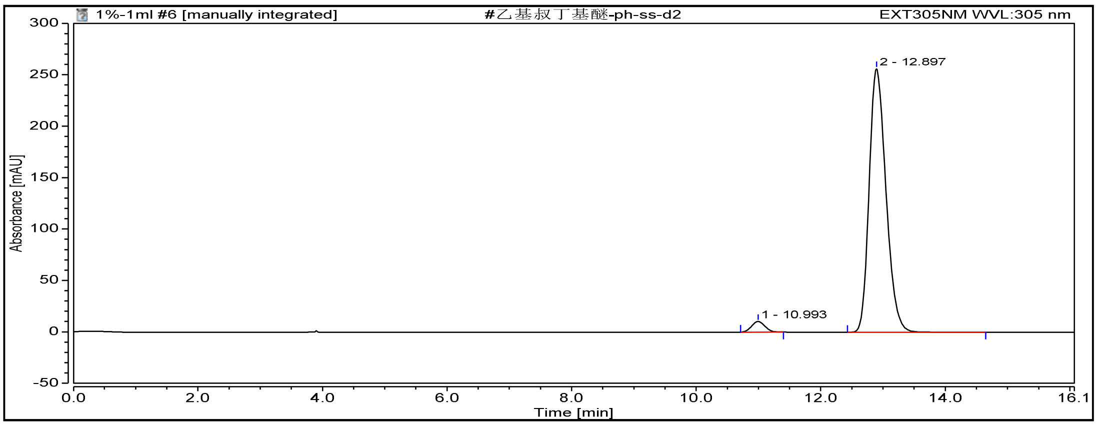

Integration Results					
No.	Peak Name	Retention Time<br>min	Area<br>mAU*min	Relative Area<br>%	Amount<br>n.a.
1		10.993	2.569	3.10	n.a.
2		12.897	80.236	96.90	n.a.
**Total:**			**82.805**	**100.00**	

Supplementary figure 263. HPLC chromatogram for compound **6i**

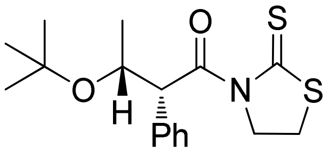

6i’

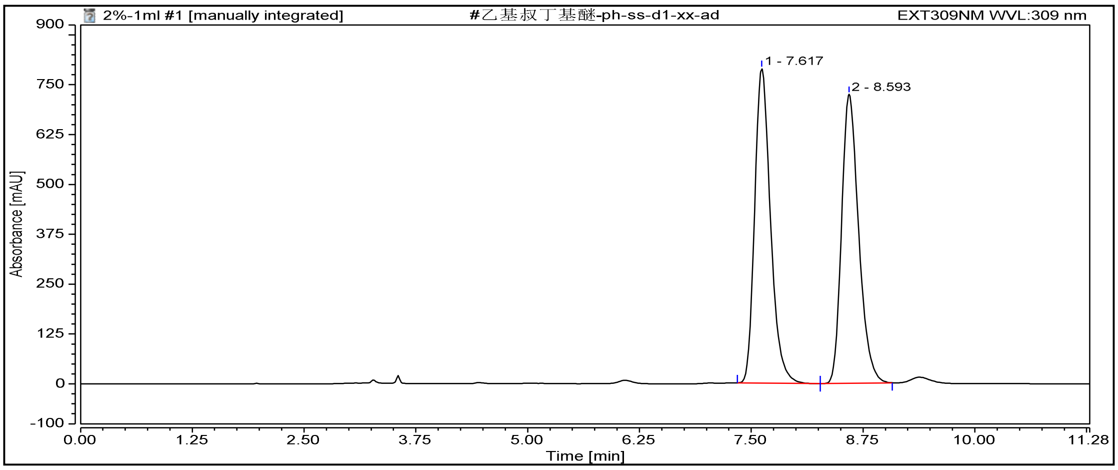

Integration Results					
No.	Peak Name	Retention Time<br>min	Area<br>mAU*min	Relative Area<br>%	Amount<br>n.a.
1		7.617	162.159	50.32	n.a.
2		8.593	160.079	49.68	n.a.
**Total:**			**322.238**	**100.00**	

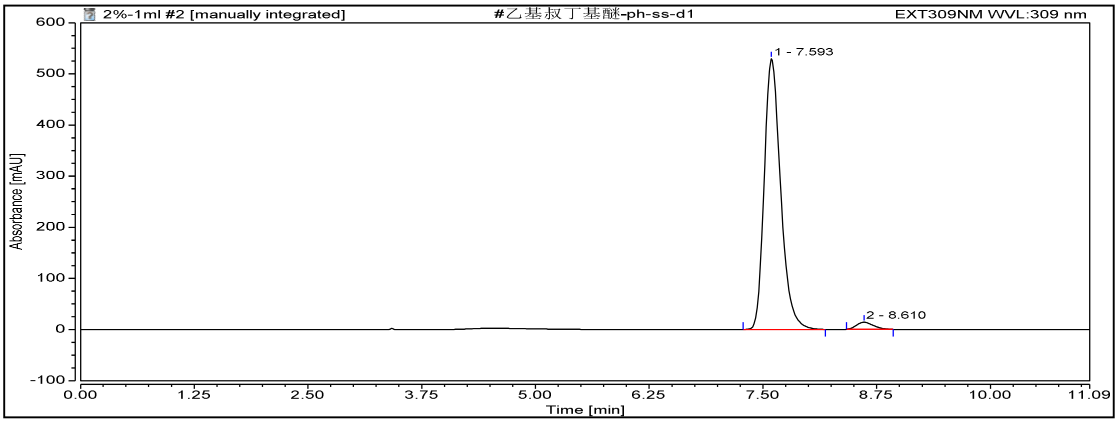

Integration Results					
No.	Peak Name	Retention Time<br>min	Area<br>mAU*min	Relative Area<br>%	Amount<br>n.a.
1		7.593	111.064	97.39	n.a.
2		8.610	2.981	2.61	n.a.
**Total:**			**114.046**	**100.00**	

Supplementary figure 264. HPLC chromatogram for compound **6i’**

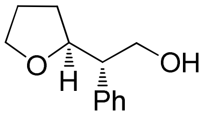

7

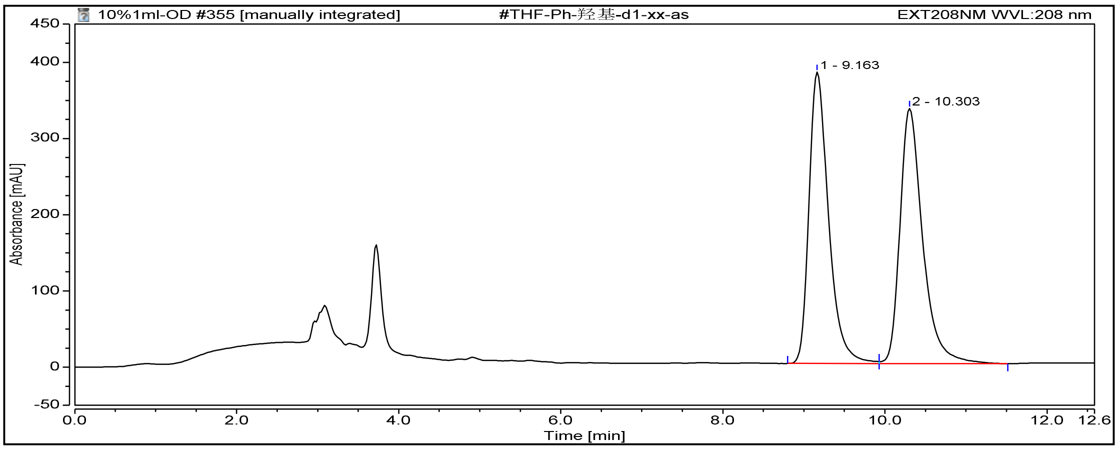

Integration Results					
No.	Peak Name	Retention Time min	Area mAU*min	Relative Area %	Amount n.a.
1		9.163	107.106	49.63	n.a.
2		10.303	108.682	50.37	n.a.
**Total:**			**215.788**	**100.00**	

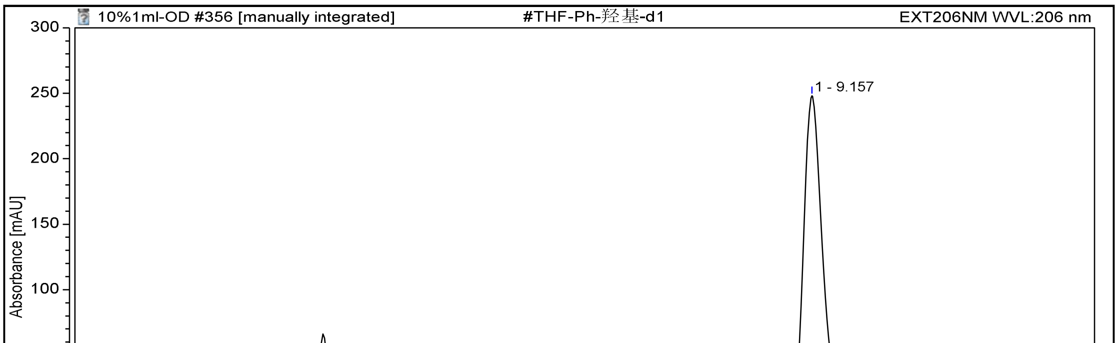

Integration Results					
No.	Peak Name	Retention Time min	Area mAU*min	Relative Area %	Amount n.a.
1		9.157	69.439	98.41	n.a.
2		10.300	1.119	1.59	n.a.
**Total:**			**70.558**	**100.00**	

Supplementary figure 265. HPLC chromatogram for compound **7**

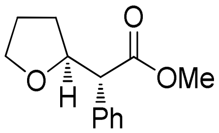

8

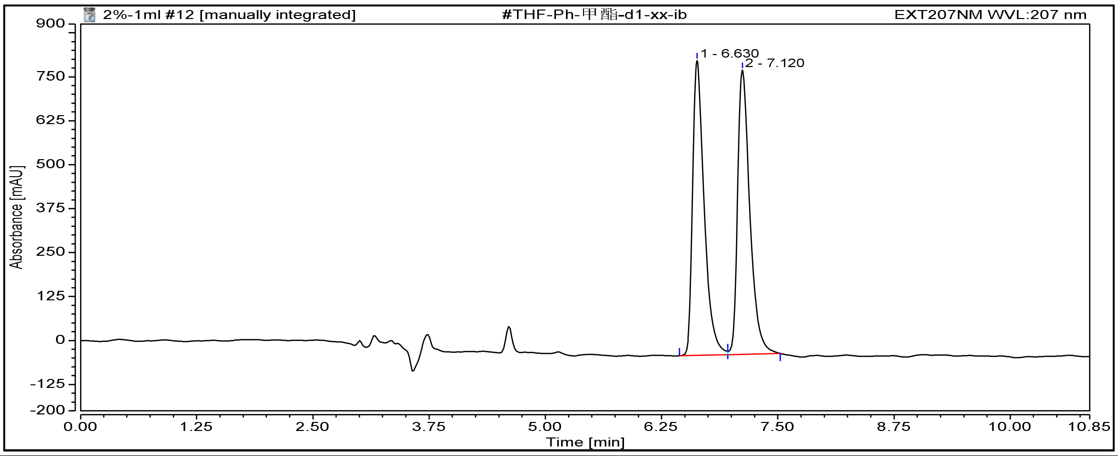

Integration Results					
No.	Peak Name	Retention Time<br>min	Area<br>mAU*min	Relative Area<br>%	Amount<br>n.a.
1		6.630	121.421	49.64	n.a.
2		7.120	123.170	50.36	n.a.
**Total:**			**244.590**	**100.00**	

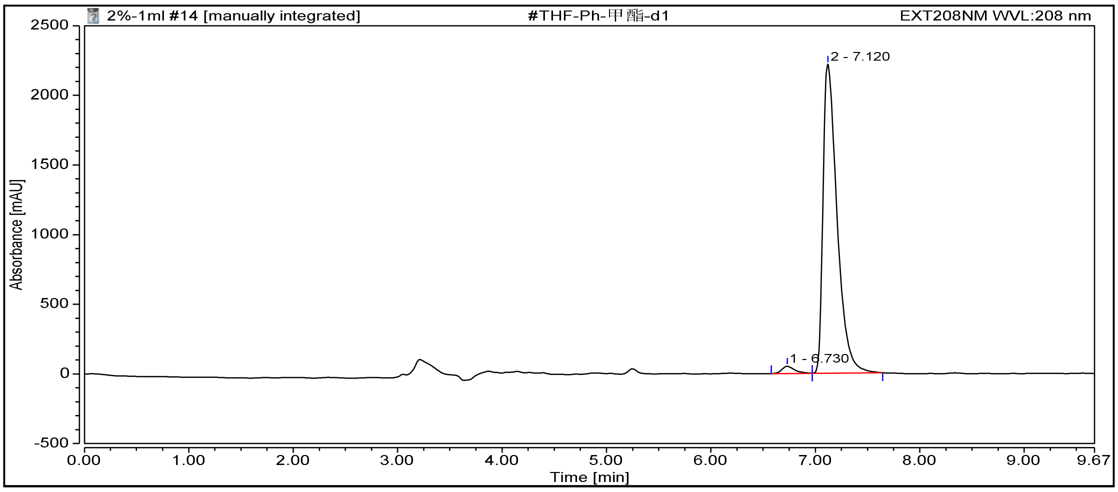

Integration Results					
No.	Peak Name	Retention Time<br>min	Area<br>mAU*min	Relative Area<br>%	Amount<br>n.a.
1		6.730	7.335	2.07	n.a.
2		7.120	346.421	97.93	n.a.
**Total:**			**353.756**	**100.00**	

Supplementary figure 266. HPLC chromatogram for compound **8**

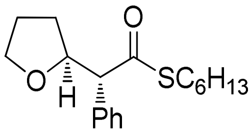

9

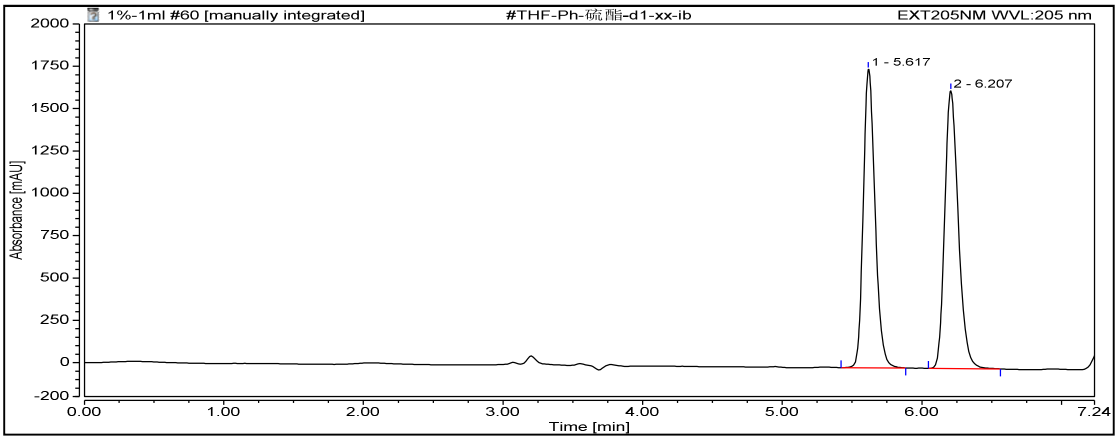

Integration Results					
No.	Peak Name	Retention Time min	Area mAU*min	Relative Area %	Amount n.a.
1		5.617	172.251	48.19	n.a.
2		6.207	185.198	51.81	n.a.
**Total:**			**357.449**	**100.00**	

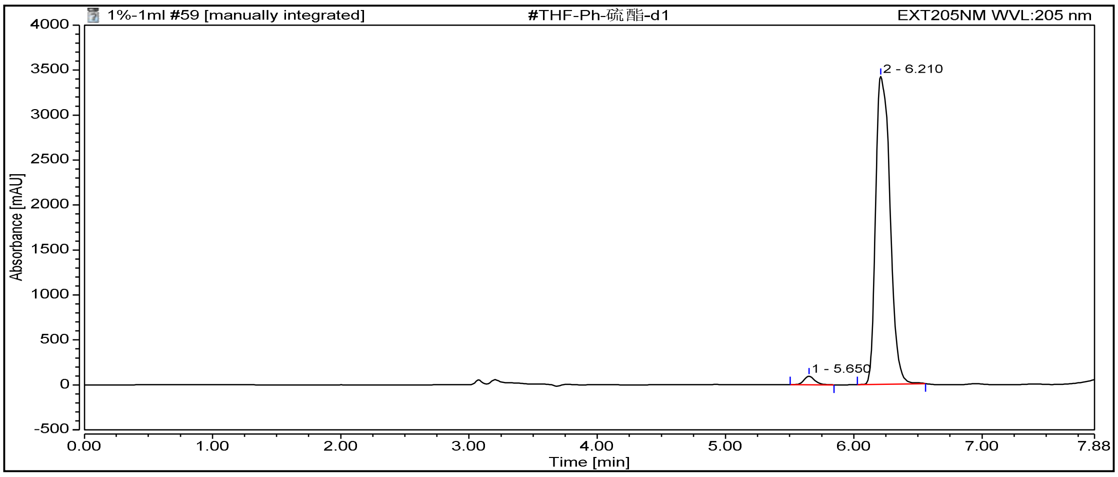

Integration Results					
No.	Peak Name	Retention Time min	Area mAU*min	Relative Area %	Amount n.a.
1		5.650	9.539	2.09	n.a.
2		6.210	446.088	97.91	n.a.
**Total:**			**455.627**	**100.00**	

Supplementary figure 267. HPLC chromatogram for compound **9**

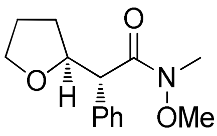

10

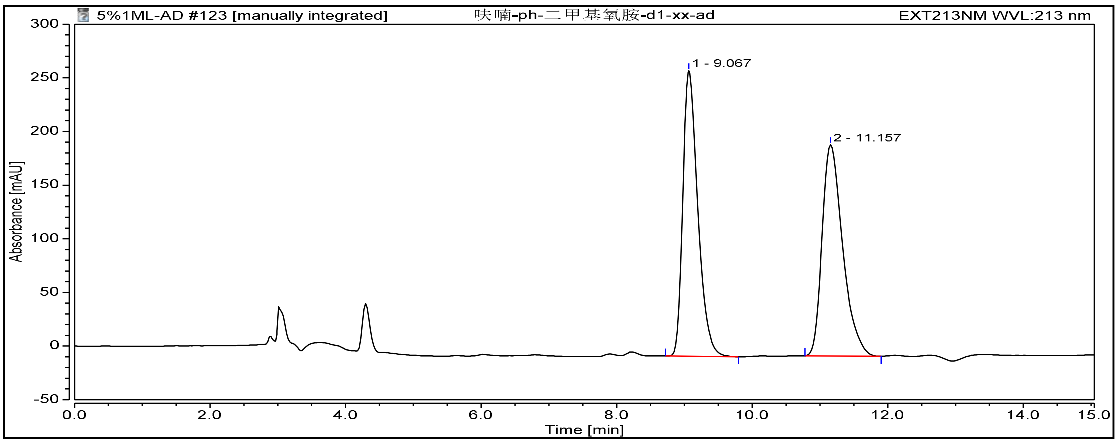

Integration Results

No.	Peak Name	Retention Time min	Area mAU*min	Relative Area %	Amount n.a.
1		9.067	68.405	49.74	n.a.
2		11.157	69.130	50.26	n.a.
**Total:**			**137.535**	**100.00**	

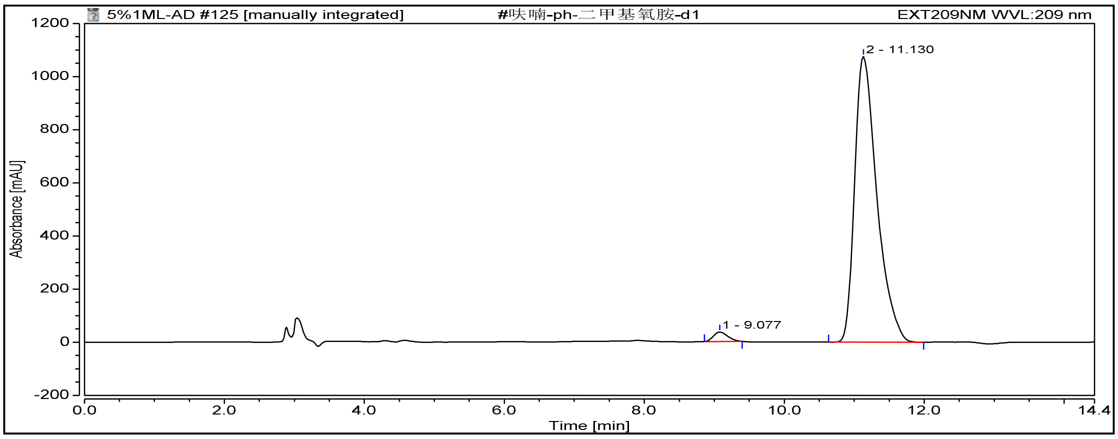

Integration Results

No.	Peak Name	Retention Time min	Area mAU*min	Relative Area %	Amount n.a.
1		9.077	8.453	2.09	n.a.
2		11.130	396.138	97.91	n.a.
**Total:**			**404.591**	**100.00**	

Supplementary figure 268. HPLC chromatogram for compound **10**

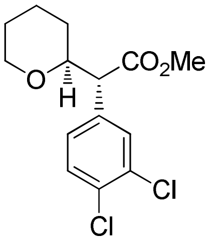

12a

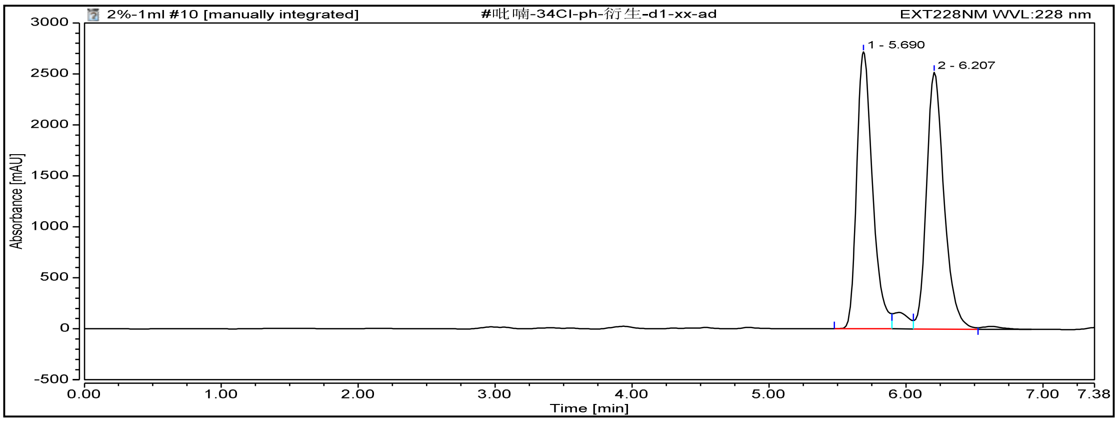

Integration Results					
No.	Peak Name	Retention Time<br>min	Area<br>mAU*min	Relative Area<br>%	Amount<br>n.a.
1		5.690	364.579	49.34	n.a.
2		6.207	374.380	50.66	n.a.
**Total:**			**738.959**	**100.00**	

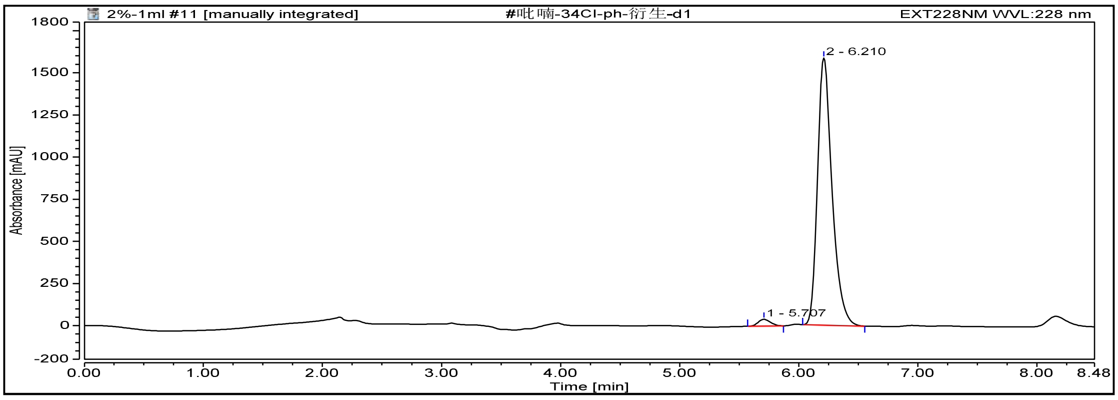

Integration Results					
No.	Peak Name	Retention Time<br>min	Area<br>mAU*min	Relative Area<br>%	Amount<br>n.a.
1		5.707	4.798	2.08	n.a.
2		6.210	225.877	97.92	n.a.
**Total:**			**230.676**	**100.00**	

Supplementary figure 269. HPLC chromatogram for compound **12a**

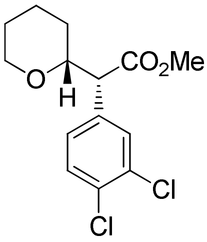

12b

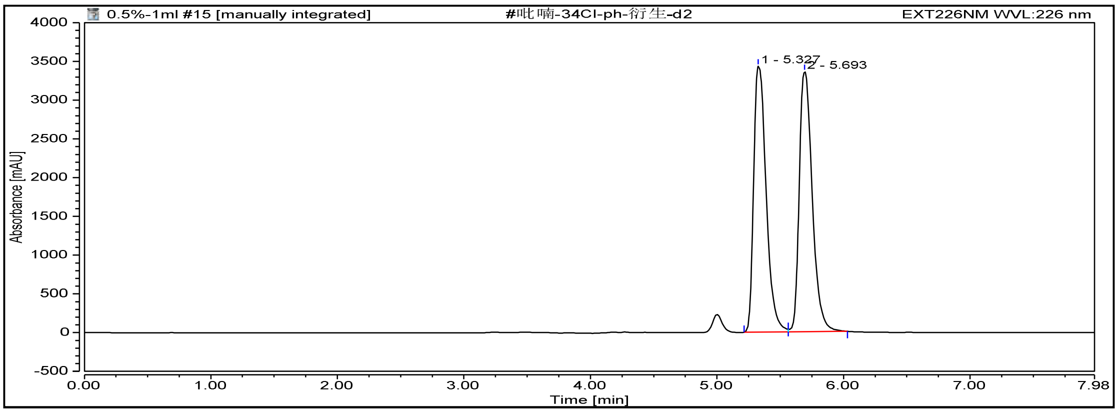

Integration Results					
No.	Peak Name	Retention Time min	Area mAU*min	Relative Area %	Amount n.a.
1		5.327	309.315	97.51	n.a.
2		5.723	7.884	2.49	n.a.
**Total:**			**317.198**	**100.00**	

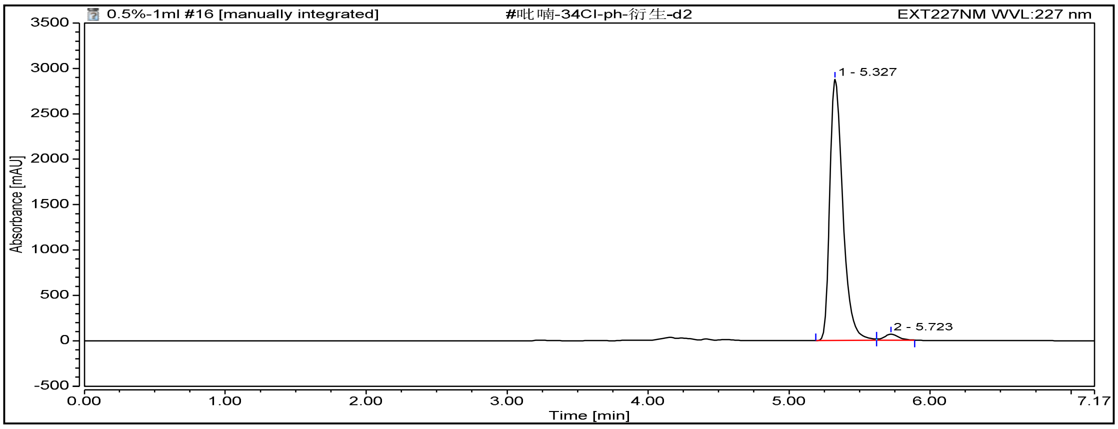

Integration Results					
No.	Peak Name	Retention Time min	Area mAU*min	Relative Area %	Amount n.a.
1		5.327	309.315	97.51	n.a.
2		5.723	7.884	2.49	n.a.
**Total:**			**317.198**	**100.00**	

Supplementary figure 270. HPLC chromatogram for compound **12b**

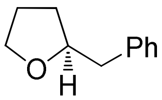

13

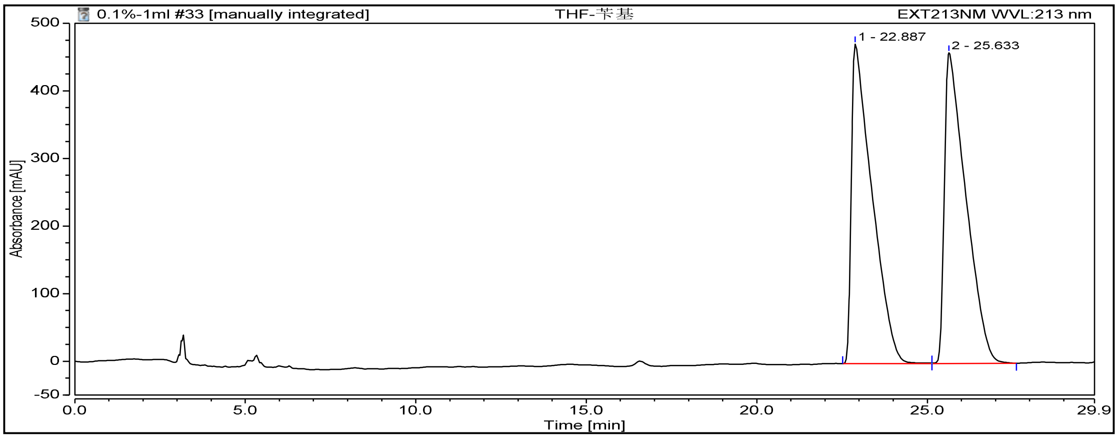

Integration Results					
No.	Peak Name	Retention Time<br>min	Area<br>mAU*min	Relative Area<br>%	Amount<br>n.a.
1		22.887	331.442	49.74	n.a.
2		25.633	334.940	50.26	n.a.
**Total:**			**666.382**	**100.00**	

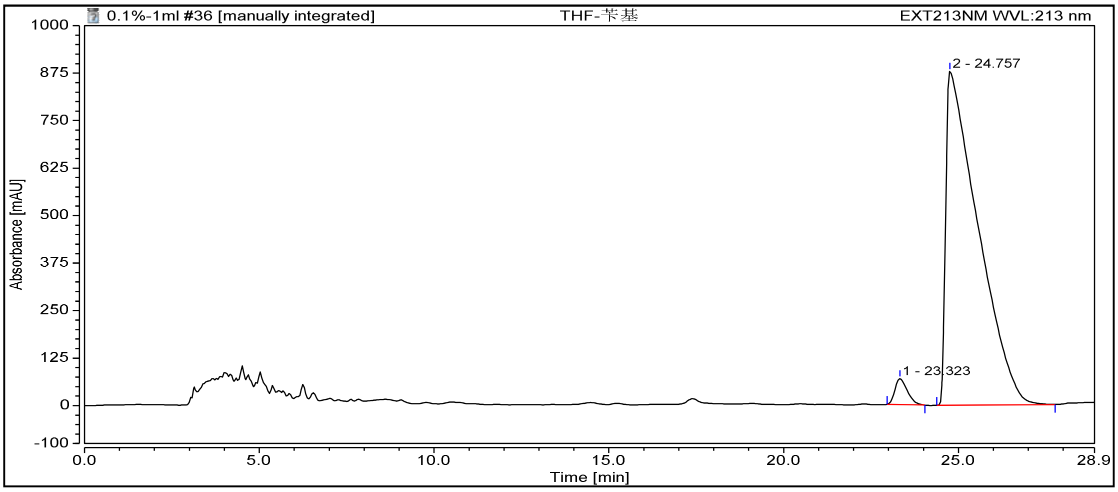

Integration Results					
No.	Peak Name	Retention Time<br>min	Area<br>mAU*min	Relative Area<br>%	Amount<br>n.a.
1		23.328	35.327	2.69	n.a.
2		24.757	1279.778	97.31	n.a.
**Total:**			**1315.104**	**100.00**	

Supplementary figure 271. HPLC chromatogram for compound **13**

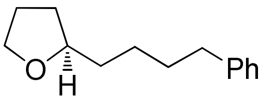

14

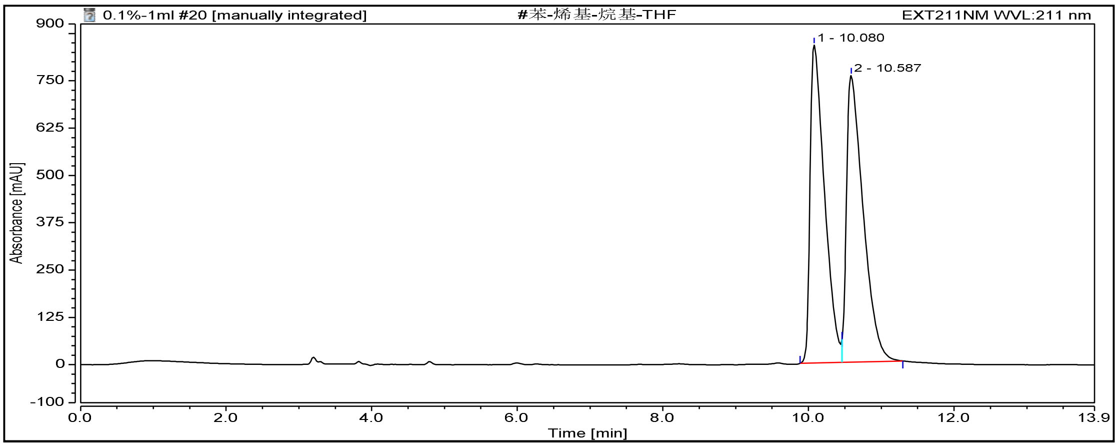

Integration Results					
No.	Peak Name	Retention Time<br>min	Area<br>mAU*min	Relative Area<br>%	Amount<br>n.a.
1		10.080	181.288	49.01	n.a.
2		10.587	188.601	50.99	n.a.
**Total:**			**369.889**	**100.00**	

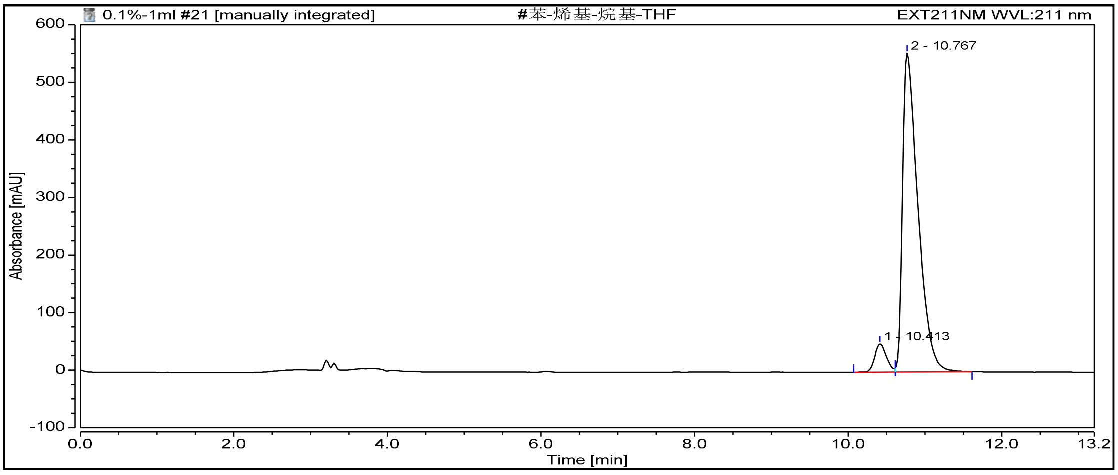

Integration Results					
No.	Peak Name	Retention Time<br>min	Area<br>mAU*min	Relative Area<br>%	Amount<br>n.a.
1		10.413	8.680	6.30	n.a.
2		10.767	129.164	93.70	n.a.
**Total:**			**137.844**	**100.00**	

Supplementary figure 272. HPLC chromatogram for compound **14**

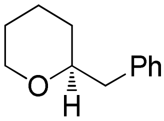

15

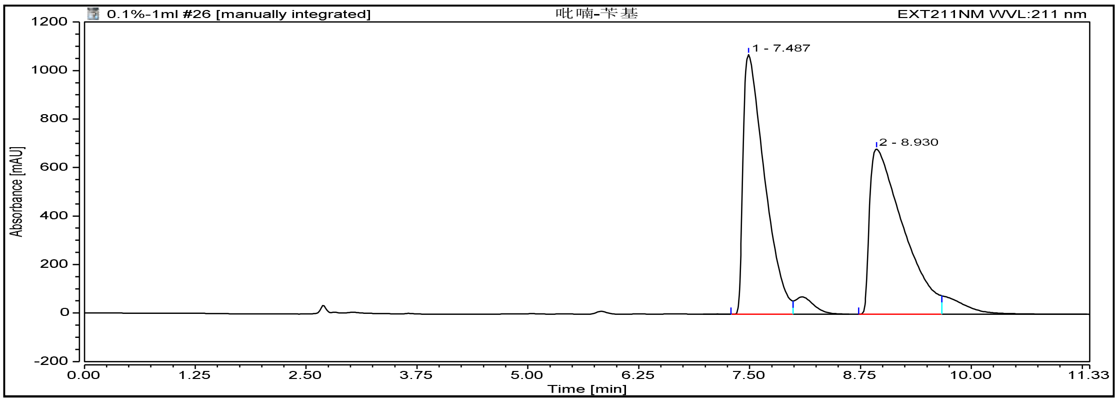

Integration Results

No.	Peak Name	Retention Time<br>min	Area<br>mAU*min	Relative Area<br>%	Amount<br>n.a.
1		7.487	301.604	50.11	n.a.
2		8.930	300.222	49.89	n.a.
**Total:**			**601.826**	**100.00**	

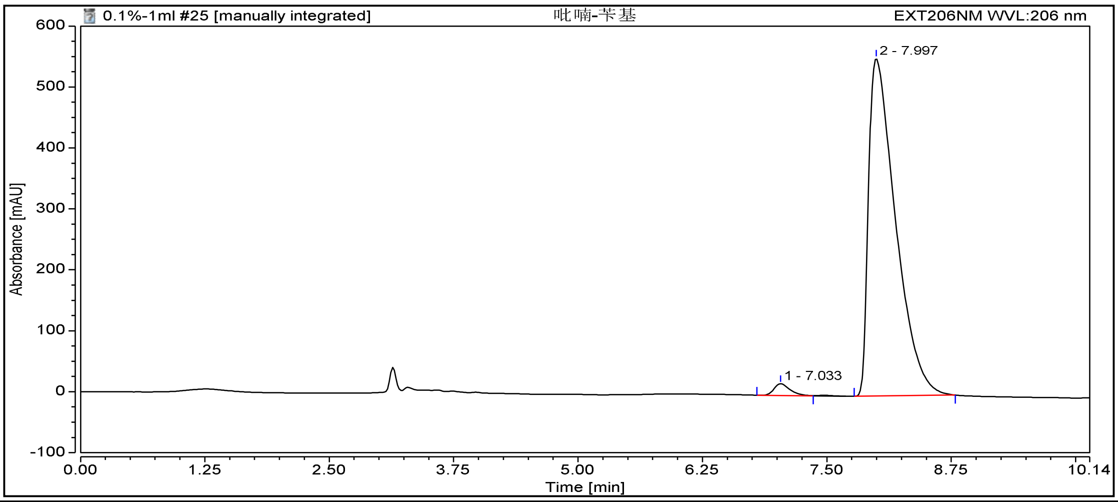

Integration Results

No.	Peak Name	Retention Time<br>min	Area<br>mAU*min	Relative Area<br>%	Amount<br>n.a.
1		7.033	3.631	2.04	n.a.
2		7.997	174.277	97.96	n.a.
**Total:**			**177.908**	**100.00**	

Supplementary figure 273. HPLC chromatogram for compound **15**

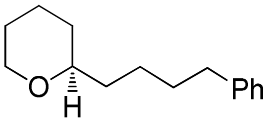

16

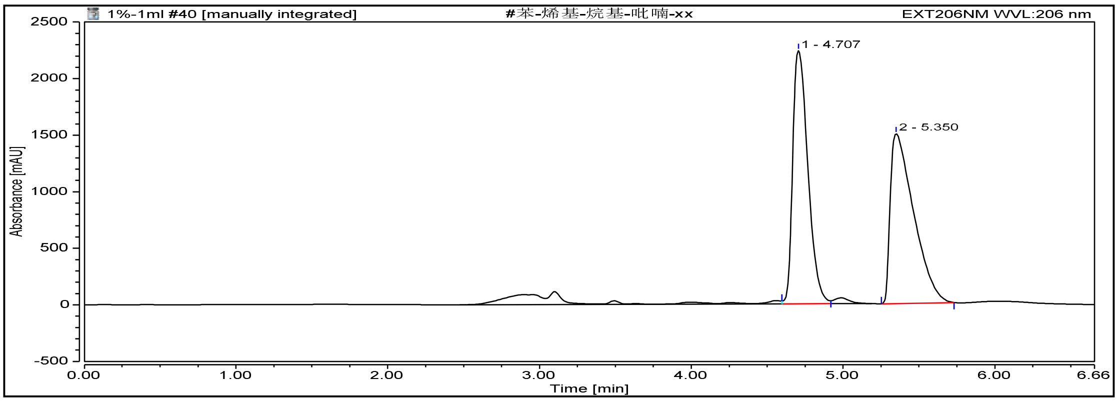

Integration Results					
No.	Peak Name	Retention Time min	Area mAU*min	Relative Area %	Amount n.a.
1		4.707	259.338	49.23	n.a.
2		5.350	267.414	50.77	n.a.
**Total:**			**526.752**	**100.00**	

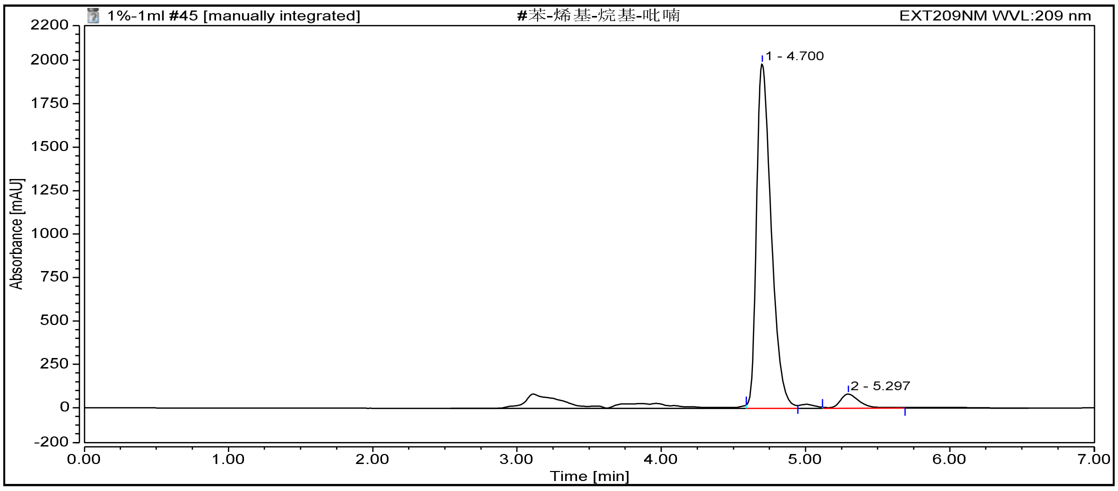

Integration Results					
No.	Peak Name	Retention Time min	Area mAU*min	Relative Area %	Amount n.a.
1		4.700	235.116	94.69	n.a.
2		5.297	13.194	5.31	n.a.
**Total:**			**248.310**	**100.00**	

Supplementary figure 274. HPLC chromatogram for compound **16**

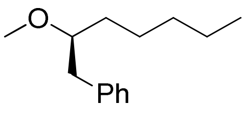

17

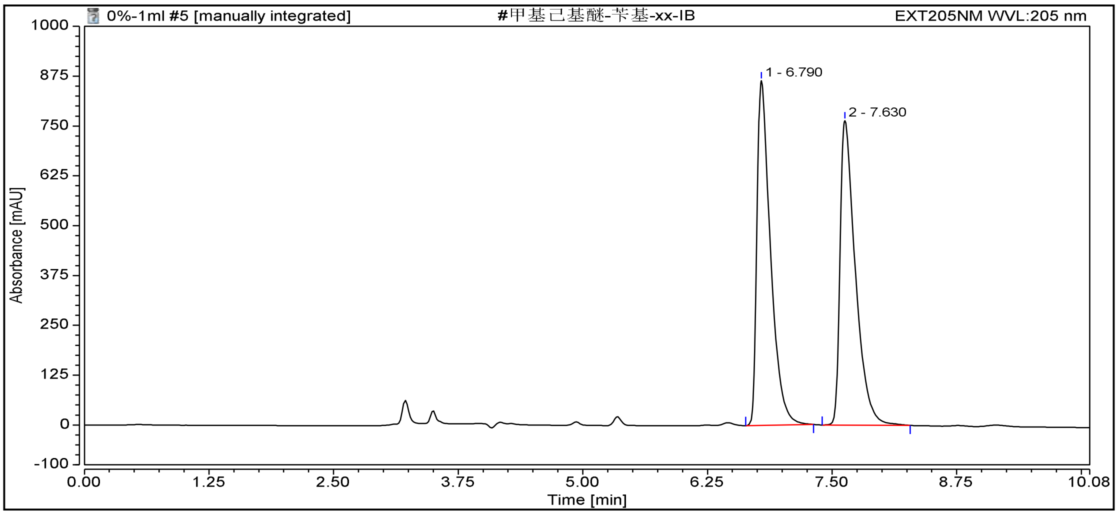

Integration Results					
No.	Peak Name	Retention Time<br>min	Area<br>mAU*min	Relative Area<br>%	Amount<br>n.a.
1		6.790	136.929	50.05	n.a.
2		7.630	136.658	49.95	n.a.
**Total:**			**273.586**	**100.00**	

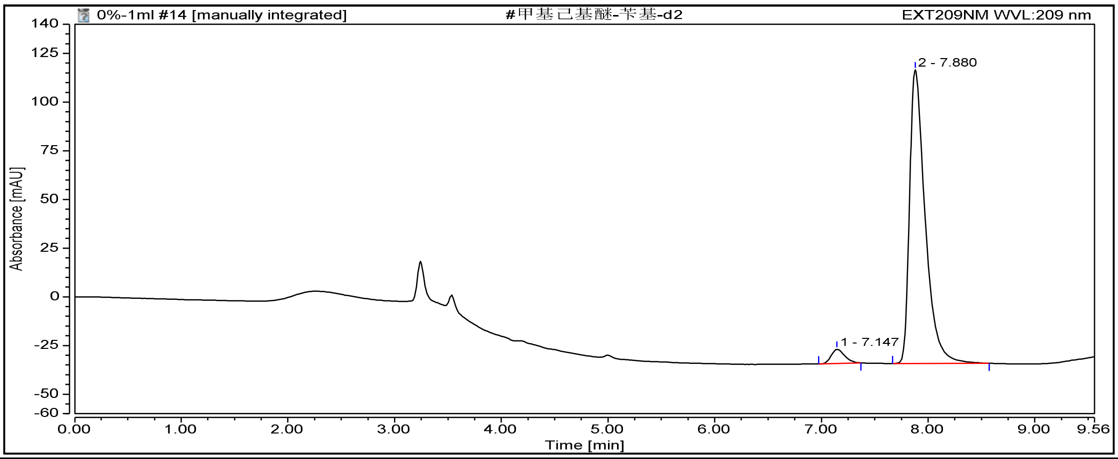

Integration Results					
No.	Peak Name	Retention Time<br>min	Area<br>mAU*min	Relative Area<br>%	Amount<br>n.a.
1		7.147	1.069	4.02	n.a.
2		7.880	25.501	95.98	n.a.
**Total:**			**26.570**	**100.00**	

Supplementary figure 275. HPLC chromatogram for compound **17**

Supplementary References

1. Cacho, R. A.; Thuss, J.; Xu, W.; Sanichar, R.; Gao, Z.; Nguyen, A.; Vederas, J. C. & Tang, Y. Understanding programming of fungal iterative polyketide synthases: The biochemical basis for regioselectivity by the methyltransferase domain in the lovastatin megasynthase. *J. Am. Chem. Soc.* **137**, 15688-15691 (2015).
2. Meltzer, P.; Wang, P.; Blundell, P. & Madras, B. K. Synthesis and evaluation of dopamine and serotonin transporter inhibition by oxacyclic and carbacyclic analogues of methylphenidate. *J. Med. Chem.* **46**, 1538-1545 (2003).
3. Chen, L.; Shi, E.; Liu, Z.; Chen, S.; Wei, W.; Li, H.; Xu, K. & Wan. X. Bu_4NI-catalyzed C–O bond formation by using a cross-dehydrogenative coupling (CDC) reaction. *Chem. – Eur. J.* **17**, 4085-4089 (2011).
4. Crimmins, M. T.; King, B. W.; Tabet, E. A. & Chaudhary, K. Asymmetric aldol additions: use of titanium tetrachloride and (-)-Sparteine for the soft enolization of *N*-acyl oxazolidinones, oxazolidinethiones, and thiazolidinethiones. *J. Org. Chem*. **66**, 894-902 (2001).
